# Catalytic asymmetric synthesis of cannabinoids and menthol from neral

**DOI:** 10.1038/s41586-023-05747-9

**Published:** 2023-03-01

**Authors:** Joyce A. A. Grimm, Hui Zhou, Roberta Properzi, Markus Leutzsch, Giovanni Bistoni, Johanna Nienhaus, Benjamin List

**Affiliations:** 1 Max-Planck-Institut für Kohlenforschung, Mülheim an der Ruhr, Germany; 2 Department of Chemistry, Biology and Biotechnology, University of Perugia, Perugia, Italy

**Keywords:** Asymmetric catalysis, Organocatalysis

## Abstract

The selective conversion of natural or synthetic neral to (1*R*,6*S*)-*trans*-isopiperitenol would enable and expedite sustainable routes to menthol^1,2^ and cannabinoids^3–5^. However, this reaction has been considered impossible because its product is more reactive to the required acid catalysts than its starting material, resulting in several side products^6–9^. We now show that an unsymmetric, strong and confined chiral acid, a highly fluorinated imino-imidodiphosphate, catalyses this process with excellent efficiency and selectivity. Expanding the method to other α,β-unsaturated aldehydes could enable access to new cannabinoids and menthol derivatives not readily accessible previously. Mechanistic studies suggest that the confined catalyst accomplishes this reaction by binding the product in an unreactive conformation, thereby preventing its decomposition. We also show how (1*R*,6*S*)-*trans*-isopiperitenol can be readily converted to pharmaceutically useful cannabinoids and menthol, each in the shortest and most atom-economic routes so far.

## Abstract (web summary)

An unsymmetric, strong and confined chiral acid, a highly fluorinated imino-imidodiphosphate, catalyses the selective conversion of neral to (1*R*,6*S*)-*trans*-isopiperitenol, enabling sustainable routes to menthol and cannabinoids.

## Main

The asymmetric cyclization of neral to isopiperitenol constitutes an equally attractive and challenging problem for chemists. Although (1*S*,6*R*)-*trans*-isopiperitenol occurs in nature, access to its enantiomer the non-natural (1*R*,6*S*)-*trans*-isopiperitenol would enable extremely short routes to (–)-menthol^1,2^ and several cannabinoids^3–5^. The growing market volume for synthetic (–)-menthol, which is used in a multitude of consumer products, encompasses hundreds of millions of US dollars. It is used primarily as a cooling and refreshing agent, because menthol is a chemical agonist of the cold-sensitive TRPM8 channel^10^. Similarly, several cannabinoids, such as Δ^9^-tetrahydrocannabinol (Δ^9^-THC) and cannabidiol (CBD), are approved for the treatment of side effects in cancer therapy and show promise against several ailments^11,12^. Cannabinoids target the two G-protein-coupled cannabinoid receptors CB1 and CB2 (refs. ^13,14^). There is increasing demand for cannabinoids, accompanying the current global trend of legalization for medical and recreational use. Consequently, ever more efficient, industrially applicable routes towards their synthesis are sought, ideally circumventing tedious extraction processes from plants. An elegant and high-yielding approach to cannabinoids uses enantioenriched monoterpene derivatives such as *p*-mentha-2,8-dien-1-ol^15^ or *trans*-isopiperitenol^3,4^. However, access to these terpene building blocks, especially isopiperitenol, requires several steps starting from already enantioenriched natural products^3,16,17^.

**Fig. 1:**
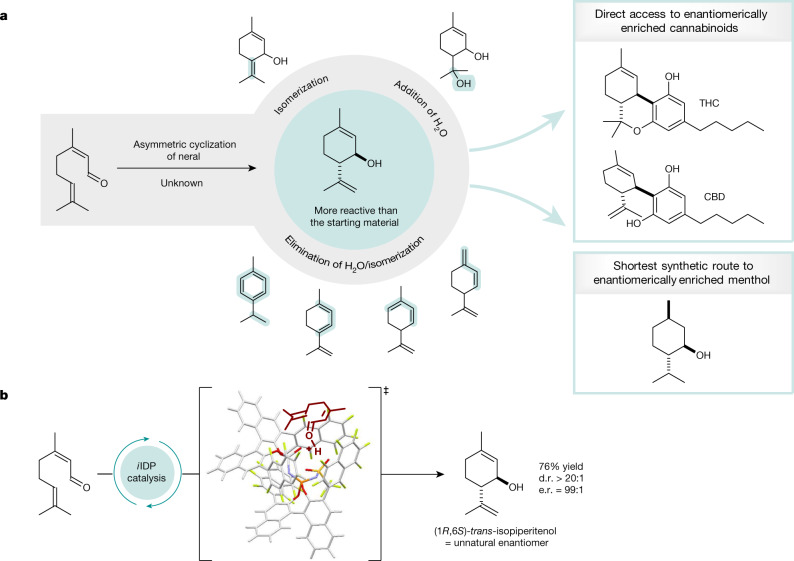
Brønsted acid-catalysed cyclization of neral to (1*R*,6*S*)-*trans*-isopiperitenol. **a**, Selectivity problems in the cyclization of neral named the ‘neral to isopiperitenol dilemma’ and isopiperitenol as valuable chiral pool material in the synthesis of cannabinoids and menthol. **b**, The first asymmetric, selective cyclization of neral to isopiperitenol under confined Brønsted acid catalysis.

Despite early efforts in the late nineteenth century on the conversion of neral to isopiperitenol from Verley^18^ and Semmler^19^, it took nearly 100 years to fully uncover its complexity^6–9^. Under acidic conditions, the cyclization of neral follows a stepwise Prins-like mechanism and initially gives rise to isopiperitenol. However, isopiperitenol, a cyclic allylic alcohol, although stable under neutral conditions, is only metastable even under weakly acidic conditions and is prone to elimination of water, giving complex mixtures of cyclic trienes and aromatic compounds (Fig. 1). Further side reactions that have been characterized include double-bond isomerizations and, in aqueous media, the re-addition of water furnishing different alcohols. Even though a variety of reaction conditions has previously been evaluated in depth, isopiperitenol can be isolated in only low yields making it merely a reaction intermediate. In the meantime, significant progress has been made towards catalytic asymmetric intramolecular carbonyl-ene and Prins cyclizations of olefinic carbonyl compounds^20–23^.

For example, we have contributed a highly enantioselective Brønsted acid-catalysed carbonyl-ene cyclization^21^, proceeding via a Prins mechanism, to give five-membered ring products. However, the reactivity towards six-membered rings is insufficient and α,β-unsaturated aldehydes are not investigated in this study. Very recently, the Jacobsen group reported intramolecular Prins-type cyclizations of α,β-unsaturated aldehydes applying cooperative hydrogen-bond-donor catalysis^23^. This system seems to be limited to aryl-substituted substrates, and β,β-dialkyl aldehydes such as neral are absent in this report. Inspired by a long-standing interest in the chemistry of citral and encouraged by our previous studies on confinement-controlled reactivity, for example in single aldolizations of acetaldehyde enolates^24^ and conformationally induced Nazarov cyclizations^25^, we became interested in the ‘neral to isopiperitenol dilemma’ (Fig. 1). We expected a member of our readily tunable confined Brønsted acid catalyst portfolio to provide sufficient reactivity and enantioselectivity, enabling the smooth asymmetric cyclization of the small and unengineered neral substrate, while simultaneously hoping that enzyme-like confinement effects could prevent product decomposition. Here, we report the development of a new type of unsymmetric and highly fluorinated confined acid that converts neral to (1*R*,6*S*)-*trans*-isopiperitenol, providing asymmetric access to five useful cannabinoids, as well as to menthol and piperitol, each in the shortest and most atom-economic route reported so far.

At the onset, several mineral and organic achiral Brønsted acids covering a broad p*K*_a_ range were tested in the targeted reaction (Supplementary Information). Consistent with previous reports^6–9^, activation of neral (**1**) depends strongly on the acidity of the catalyst. Whereas mild acids barely show conversion, stronger acids readily activate the substrate. However, this reactivity is usually accompanied by the fast decomposition of isopiperitenol (**2**) leading to an unselective reaction.

**Fig. 2:**
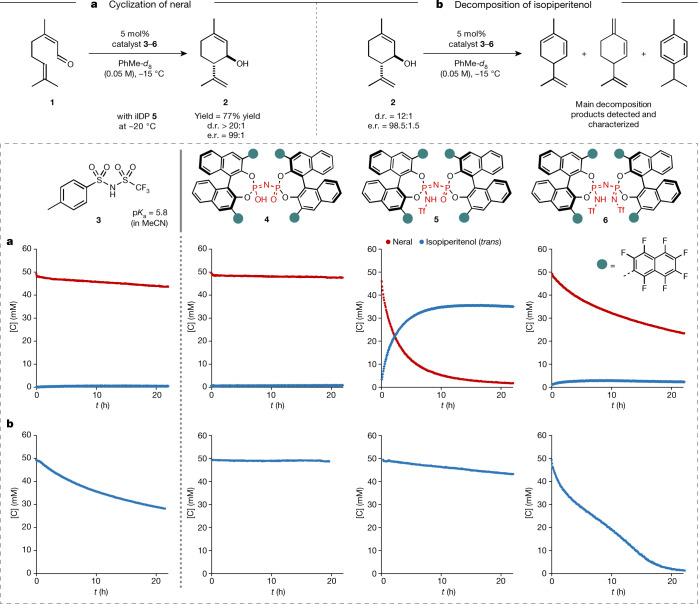
Comparison of different achiral and chiral Brønsted acids 3–6 in the formation and decomposition of isopiperitenol (2). **a**,**b**, Four different catalysts were used for ^1^H NMR reaction monitoring of the cyclization of neral (**a**) and the reaction of isopiperitenol to undesired side products (**b**) under optimized reaction conditions. d.r. and e.r. were determined by gas chromatography (GC) analysis. The shown decomposition products only represent the main side products characterized by ^1^H NMR spectroscopy.

The ideal acid catalyst required for this transformation therefore needs to reach a certain p*K*_a_ threshold to activate aldehyde **1**, while suppressing the decomposition of **2**. We exemplarily confirmed the higher reactivity of isopiperitenol versus neral with achiral acid **3** (p*K*_a_ = 5.8, MeCN) using ^1^H nuclear magnetic resonance (NMR) reaction monitoring (Fig. 2). Using acid **3**, neral (**1**) remained mostly unreacted (conversion less than 10%) and isopiperitenol (**2**) was decomposed by almost half in 20 h, notably illustrating the challenge at hand. Comparing the readily tunable confined Brønsted acid catalysts **4**–**6** shed light on the significance of confinement as well as the inner-core architecture towards catalysing the targeted cyclization (Fig. 2). Indeed, we find that not only is a certain acidity required to achieve a balance between the activation of aldehyde **1** and the decomposition of allylic alcohol **2**, but so too is a non-C_2_-symmetric inner core. Accordingly, imino-imidodiphosphates (iIDP), featuring a bifunctional inner-core system with an acidic P=NHTf moiety and a basic P=O moiety, combine excellent reactivity and selectivity for the cyclization of neral (**1**) to isopiperitenol (**2**). We were intrigued to find that best results were obtained with highly fluorinated iIDP catalyst **5**, which furnishes (1*R*,6*S*)-*trans*-isopiperitenol in good yield (77%) and excellent diastereo- and enantioselectivity (d.r. > 20:1; e.r. = 99:1) (Fig. 2).

**Fig. 3:**
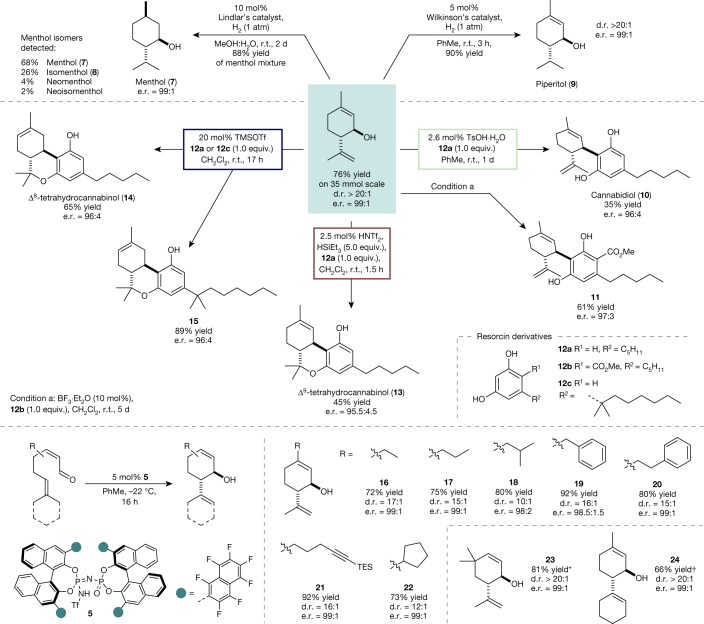
Isopiperitenol as a valuable, chiral material in the syntheses of menthol, piperitol and cannabinoids. Hydrogenation reactions to **7** and **9** were performed on 1.0 mmol scale. Reactions to the cannabinoids were performed using an excess of isopiperitenol (1.1–1.5 equiv.). Isolated yields after chromatographic purification. d.r. and e.r. were determined by high-performance liquid chromatography or GC analysis. For the substrate scope of the asymmetric catalytic cyclization of α,β-unsaturated aldehydes, reactions were performed on a 0.25 mmol scale with iIDP **5**. Only the main *trans*-diastereoisomer is depicted. d.r. and e.r. were determined by GC analysis. TMSOTf, trimethylsilyl triflate. *Reaction was performed on a 0.025 mmol scale and yield was determined by ^1^H NMR spectroscopy using mesitylene as internal standard. ^†^Reaction stirred for 24 h. TMSOTf.

The cyclization of neral can be performed easily on a multigram scale (>4 g, 35 mmol) without any loss of selectivity or yield (Fig. 3). It is noteworthy that catalyst **5** can be recovered in excellent yield (95%) and re-used in further cyclization reactions (Supplementary Information).

Enantiomerically enriched (1*R*,6*S*)-*trans*-isopiperitenol (**2**) enables direct access to menthol, piperitol and cannabinoids. Indeed, exhaustive hydrogenation of (1*R*,6*S*)-*trans*-isopiperitenol (**2**) using 10 mol% of Lindlar’s catalyst provides 88% yield of a mixture of enantioenriched menthol isomers, in which (–)-menthol (**7**) (68%) and (–)-isomenthol (**8**) (26%) are the main products. Although separation of the isomeric menthol mixtures on a laboratory scale is tedious, purification on an industrial scale routinely involves distillation and subsequent crystallization^26,27^. It should be noted that despite the long history of technical menthol syntheses, which has provided several highly efficient routes to the stereochemically pure product^26^, our two-step synthesis starting from easily accessible neral (**1**) is perfectly atom-economic and presents the shortest route reported so far. Moreover, we found that selective hydrogenation of the external double bond using Wilkinson’s catalyst furnishes piperitol (**9**) in excellent yield. Piperitol has potential as a surfactant and odorant because of its medium strength herbal scent and antimicrobial activity^28^.

Early reports from Dethe^3^ and Mechoulam^4^ demonstrated the promising applicability of isopiperitenol in Lewis acid-catalysed Friedel–Crafts reactions with resorcinols furnishing cannabinoids. However, the commonly used Lewis acid BF_3_·Et_2_O, in our hands, although showing high reactivity, typically gives uncertain and unselective reaction profiles resulting in tedious purifications and low yields. Focusing our effort on more reliable and selective methods, we investigated catalysts of varying acidity to selectively obtain different types of cannabinoid. Remarkably, direct access to CBD (**10**) from isopiperitenol (**2**) and olivetol (**12a**) was provided in 35% yield under mild conditions using TsOH·H_2_O as catalyst (Fig. 3). It is noteworthy that no further reaction of product **10** to the corresponding tetrahydrocannabinols was observed. These mild conditions were not suitable for the synthesis of ester **11** because resorcinol **12b** proved to be insufficiently reactive in the Friedel–Crafts reaction. Fortunately, using BF_3_·Et_2_O as a catalyst readily delivered the desired CBD derivative **11** in 61% yield. The most famous Δ^9^-isomer of THC (**13**) could reliably be obtained in 45% yield by employing triflimide as the catalyst in the presence of an excess of triethylsilane. Under these Lewis acidic conditions, isomerization to the thermodynamically more stable Δ^8^-THC (**14**) product was observed only after significantly longer reaction times. Indeed, Δ^8^-THC (**14**) and its pharmaceutically relevant derivative **15** could be obtained from the reaction of isopiperitenol (**2**) and either olivetol (**12a**) or 5-(1,1-dimethylheptyl)resorcinol (**12c**) at ambient temperature, by employing 20 mol% trimethylsilyl triflate as the catalyst. To explore the generality of our new method, we investigated the cyclization of further α,β-unsaturated aldehydes. Novel cyclic, allylic alcohols **16**–**24** were obtained in good to excellent yield using optimal iIDP catalyst **5** with similarly superb enantioselectivity (e.r. > 98:2). Although elongation of the β-side chain is well tolerated, increased steric demand leads to a loss of diastereoselectivity (**18**, d.r. = 10:1). Noteworthy, the cyclohexyl group in product **24**, which is located in close proximity to the active side of the catalyst and actively engages in the reaction, slows the reaction but excellent levels of enantio- and diastereoselectivity are maintained.

**Fig. 4:**
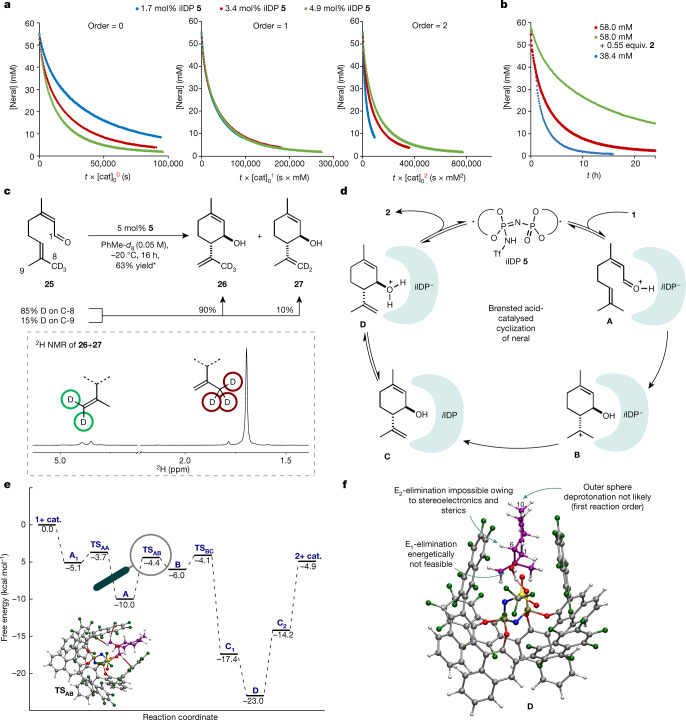
Mechanistic studies. **a**, ^1^H NMR reaction monitoring to determine the reaction order in catalyst following Burés’ method^29^. **b**, Temporal ^1^H NMR concentration profiles^31^ of the reaction under different conditions. The rate differences show strong product inhibition in the cyclization reaction. **c**, Top, deuterium-labelled experiments with 85% C-8 labelled substrate **25**. *The yield is determined by ^1^H NMR spectroscopy using mesitylene as an internal standard and is calculated on the basis of neral present in the sample (*Z*:*E* ratio = 70:30). Bottom, ^2^H NMR of the product mixture. **d**, Proposed catalytic cycle of the Brønsted acid-catalysed cyclization of neral. **e**, Free-energy profile for the cyclization reaction and the diastereo- and enantio-determining transition state TS_AB_. The strongest stereocontrolling interactions, C−H···F, are highlighted. **f**, Computed structure **D**, adduct of protonated product and catalyst **5**.

To elucidate the mechanism of the cyclization of neral to isopiperitenol and the reasons for the remarkably high selectivity exerted by privileged iIDP catalyst **5**, NMR investigations including deuterium-labelling, as well as computational studies were conducted. First, time normalization analysis following the report from Burés^29,30^ confirmed a first-order reaction in iIDP catalyst **5** for both the catalytic cyclization of neral (**1**) (Fig. 4a) and the decomposition of product **2** (Supplementary Information). Notably, the reaction order studies showed a decrease in reaction rate over time, suggesting either catalyst decomposition or product inhibition. Two different NMR experiments^31^, independently confirm the stability of the catalyst under the reaction conditions and show a strong inhibition of the reaction by the product (Fig. 4b), reminiscent of competitive inhibition observed with enzymes. At this point, we speculated that the strong interaction of confined catalyst **5** with product **2** might contribute to the peculiar selectivity of our reaction. Indeed, subsequent ^13^C NMR investigations of equimolar mixtures of neral or isopiperitenol with catalyst **5** showed significant shifts of aldehyde and alcohol peaks, suggesting interactions of both moieties with the catalyst (Supplementary Information). Second, we found that in contrast to neral, its double-bond isomer geranial is unreactive under the reaction conditions.

In addition, no notable double-bond isomerization occurred and yield and selectivity remain excellent from neral/geranial mixtures (Supplementary Information). Third, in contrast to our previous report^21^, covalent adducts were not observed by NMR or by mass spectrometry. Last, a deuterium-labelling experiment was conducted to gain insights into the nature of the cyclization reaction (stepwise versus concerted). Accordingly, deuterium-labelled neral **25** was synthesized and deuterium incorporation was found to be 85% at C-8 and 15% at C-9. In case of a concerted carbonyl-ene mechanism, abstraction of a deuterium from C-8 would be required. By contrast, a stepwise process, proceeding via a tertiary cation, could lead to abstraction of either a proton or a deuterium, potentially affected by a kinetic isotope effect. Performing the cyclization reaction using substrate **25**, the corresponding allylic alcohols **26** and **27** were obtained in 63% NMR yield at a ratio of 90:10. On the basis of these findings, a concerted mechanism seemed less probable (Fig. 4c). Because the ratio of products **26** and **27** reflects the starting material composition, both a stepwise and a highly asynchronous concerted pathway are plausible. On the basis of these studies and previous reports from our group^21,32^, the following catalytic cycle is proposed (Fig. 4d). After initial protonation of the substrate, ion pair **A** is formed. Diastereo- and enantioselectivity determining C–C–bond formation then takes place in a stepwise fashion via structure **B**, which after deprotonation leads to catalyst/product complex **C**. Either this complex or ion pair **D** seem to be the resting state of the catalytic cycle and responsible for the observed strong product inhibition. Finally, decomplexation should provide product **2** and regenerate the catalyst.

To validate our proposed catalytic cycle and to clarify the nature of the reaction mechanism (stepwise versus concerted) as well as the origin of selectivity, computational studies were conducted. The reaction profile at the M062X/def2-TZVP +C-PCM-(toluene) level of theory is shown in Fig. 4e. Interaction of the substrate with the catalyst in reactant complex **A**_**1**_ leads to facile protonation of **1** via transition state TS_AA_. An ion pair structure consisting of the protonated substrate and iIDP^−^ (**A**) is then formed. Subsequently, the reaction follows a stepwise cyclization (TS_AB_)/deprotonation (TS_BC_) pathway, proceeding through a highly reactive carbocationic intermediate (**B**) and finally delivering product complex **C**. The product catalyst complexes C_1_ (OH-acid) and C_2_ (NH-acid) differ only in the position of the acidic proton of iIDP, respectively (Supplementary Information). Interaction of the product with the catalyst in this complex leads to the formation of a stable ion pair between the alcohol protonated product and the iIDP anion (**D**). This computational result is consistent with the experimentally observed product inhibition. Concerning the selectivity of the transformation, the energy difference of the competing cyclization transition states (TS_AB_) for each of the *trans*-products (ΔΔG^‡^) is 3.6 kcal mol^−1^, which is consistent with the high degree of selectivity observed experimentally. We also carried out an analysis of key non-covalent interactions operating in these transitions states to elucidate the stereocontrolling factors of this transformation, using dispersion-corrected density functional theory in conjunction with the NCI tool (Supplementary Information). Our analysis showed that the most stable transition state is highly stabilized by dispersion forces. In particular, TS_AB_ features three close C−H···F contacts (Fig. 4e), which contribute significantly to its stability.

A fascinating explanation for the unusual preference of our confined acid catalyst to selectively process neral rather than its normally more reactive reaction product arises. By non-covalently binding, but not rapidly converting isopiperitenol further, its typically observed acid-mediated decomposition is prevented. In other words, isopiperitenol is a ‘substrate’ for our enzyme-like catalyst but only towards its protonation and not its decomposition. Computed structure **D** (Fig. 4f), showing the protonated product in the cavity of the catalyst anion, also shows how this is accomplished. A potential E2-elimination by abstracting proton H-6 in this lowest energy conformation is stereoelectronically hampered because of an unfavourable orbital alignment and the inaccessibility of this proton to the active site of the catalyst. Furthermore, an alternative E1-pathway is energetically challenged as suggested by initial calculations (Supplementary Information). A hypothetical outer sphere deprotonation of H-10 by another catalyst also seems to improbable because the decomposition follows first-order reaction kinetics. Therefore, we believe that the confined active site of our catalyst protects product **2** by stereoelectronically, sterically and energetically disfavouring its decomposition. The methods presented here may facilitate the synthesis of menthol and aid the production of cannabinoids starting from cheap and readily available non-chiral materials. We anticipate use of the concepts advanced here in the development of other challenging transformations.

## Online content

Any methods, additional references, Nature Portfolio reporting summaries, source data, extended data, supplementary information, acknowledgements, peer review information; details of author contributions and competing interests; and statements of data and code availability are available at 10.1038/s41586-023-05747-9.

## Supplementary information

Supplementary Information

Supplementary data XYZ coordinates.

Supplementary data XYZ coordinates.

## Data Availability

The experimental procedures and analytical data supporting the findings of this study are available in the manuscript and its Supplementary Information file. Raw and unprocessed NMR data are available from the corresponding author on reasonable request.
