# Supplementary material for: Catalytic asymmetric synthesis of cannabinoids and menthol from neral
Source: Nature. 2023 Mar 1;615(7953):634–9. doi: 10.1038/s41586-023-05747-9 (PMC10033408; doi:10.1038/s41586-023-05747-9)
Supplement: Supplementary file 1 — Supplementary Information [file 41586_2023_5747_MOESM1_ESM.pdf]

nature portfolio

https://doi.org/10.1038/s41586-023-05747-9

Supplementary information

Catalytic asymmetric synthesis of cannabinoids and menthol from neral

In the format provided by the authors and unedited

Supplementary Information

Catalytic Asymmetric Synthesis of Cannabinoids and Menthol from Neral

Joyce A. A. Grimm, Hui Zhou, Roberta Properzi, Markus Leutzsch, Giovanni Bistoni, Johanna Nienhaus, Benjamin List*

Max-Planck-Institut für Kohlenforschung, Kaiser-Wilhelm-Platz 1, 45470 Mülheim an der Ruhr

*list@kofo.mpg.de

Table of contents

1. Materials and Methods 3
   1.1. General Information 3
   1.2. Cyclization of neral/citral to isopiperitenol 7
      1.2.1. Evaluation of several Brønsted acid catalysts in the targeted reaction 7
      1.2.2. Optimization of reaction conditions 8
      1.2.3. Large-scale reaction of the cyclization of neral 12
   1.3. Supplementary methods 13
      1.3.1. Synthesis of 3,3'-substituted binaphthyl diols 13
      1.3.2. Synthesis of chiral Brønsted acids 16
      1.3.3. Synthesis of racemic and enantiomerically pure isopiperitenol 27
      1.3.4. Hydrogenation of Isopiperitenol 28
      1.3.5. Synthesis of Cannabinoids 30
      1.3.6. Synthesis of α,β-unsaturated aldehydes 35
      1.3.7. Cyclization of α,β-unsaturated aldehydes 51
      1.3.8. Synthesis of deuterium-labelled substrate analogue **25** 57
   1.4. Supplementary data 61
      1.4.1. Copies of NMR spectra 61
      1.4.2. Copies of HPLC traces 134
      1.4.3. Copies of GC traces 141
2. Supplementary text 160
   2.1. NMR spectroscopic kinetic studies 160
   2.2. Rotameric mixtures of IDP, *i*IDP and IDPi-catalysts 167
   2.3. Computational methods 171
      2.3.1. Computational details 171
      2.3.2. Free Energy Profiles associated with the minium energy pathway – connecting the substrate with the enantiomeric products experimentally detected 173
      2.3.3. Proposed catalytic cycle of the Brønsted-acid catalyzed cyclization of neral 174
      2.3.4. NCI-Plot 175
      2.3.5. Initial studies on the E1-elimination pathway 176
      2.3.6. Alternative pathways 177
      2.3.7. Calculation of the KIE 177
      2.3.8. ORCA 5.0.3 input examples 178
      2.3.9. XYZ structures 179

References 305

1. Materials and Methods

1.1. General Information

Chemicals

Chemicals were purchased from commercial suppliers (Abcr, Acros Organics, Alfa Aesar, Apollo Scientific, Fisher Scientific, Fluorochem, Manchester Organics, Oxchem Corporation, Sigma-Aldrich, Strem Chemicals, TCI Deutschland) as reagent grade and used without further purification unless otherwise stated. Et_3N and pyridine were dried and stored over molecular sieves and under Ar. Citral and Neral were distilled prior to use and stored under Ar in Schlenk flasks in the freezer (–20 °C). The *E*/*Z*-ratio of Citral was determined by gas chromatography. The following compounds were synthesized from commercially available starting materials according to literature procedures: Neral[1], $P(NTf)Cl_3$ (**S18)** and IDPi **S5**[2], hexachlorobisphosphazonium hexachlorophosphate (HCPP) (**S16**)[3], diboronate **S11** and MOM-protected BINOL **S14**[4], diol **S17**[5], IDP **S4**[6], *i*IDP **S6** and *i*IDP **S8**[7] and achiral acid **3**[8] (subsequent acidification following the general procedure *GP–2*).

Solvents

Solvents (Et_2O, THF, 1,4-Dioxane, CH_2Cl_2, $CHCl_3$, PhMe) were dried by distillation from an appropriate drying agent in the technical department of the Max-Planck-Institut für Kohlenforschung and received in Schlenk flasks under Ar.[9] Additional solvents ($CHCl_3$, MeCN, EtOAc, MeOH) were purchased from commercial suppliers and dried over molecular sieves.

Inert gas

Dry argon (Ar) was purchased from Air Liquide with >99.5% purity.

Thin-layer chromatography

Thin-layer chromatography (TLC) was performed using silica gel pre-coated polyester sheets (POLYGRAM® SIL G/UV_{254}, 0.2 mm silica gel 60 with fluorescent indicator; Macherey-Nagel) and aluminium oxide pre-coated polyester sheets (POLYGRAM® ALOX N/UV_{254}, 0.2 mm aluminium oxide N with fluorescent indicator; Macherey-Nagel), which were visualized by irradiation with UV light ($\lambda = 254$ or 366 nm), basic $KMnO_4$, and/or phosphomolybdic acid (PMA). *$KMnO_4$ stain*: aq. NaOH (10 wt%, 1.25 mL), $KMnO_4$ (1.5 g), K_2CO_3 (10 g) in H_2O (200 mL); *PMA stain*: PMA (20 g) in EtOH (200 mL).

Preparative thin-layer chromatography was performed on silica gel pre-coated TLC plates SIL G-25 UV_{254}, 0.25 mm silica gel with fluorescent indicator (Macherey-Nagel).

Column Chromatography

Flash column chromatography (FCC) was carried out using Merck (60 Å, 230−400 mesh, particle size 0.040−0.063 mm), VWR (40−63 µm) silica gel or Thermo Scientific™ (aluminium oxide (ALOX), basic, Brockmann I, 40 to 300 µm, 60 Å) activated with 20% water using technical grade solvents. Elution was accelerated using compressed air. Automated column chromatography was conducted on a Biotage® IsoleraTM ISO–4SW instrument, using SNAP Ultra HP-Sphere™ 25 µm chromatography cartridges. All fractions containing a desired substance were combined and concentrated under reduced pressure, then redissolved in an appropriate solvent and filtered through cotton to remove silica residues. All reported yields refer to chromatographically and spectroscopically pure compounds unless otherwise stated.

Nomenclature

Nomenclature follows the suggestions proposed by the computer program ChemDraw Professional (20.1.1.125) of PerkinElmer®.

Melting Points

Melting points (m. p.) were measured on a Büchi Melting Point M-565 apparatus in open glass capillaries and are uncorrected.

IR Spectroscopy

IR spectra were recorded on a Perkin Elmer Spectrum Two FT-IR spectrometer. The spectra were measured in the neat state unless otherwise stated on a UATR Two crystal plate. Selected absorption bands are reported in wavenumbers (cm^{-1}). Intensities are reported as follows: br (broad), w (weak), m (medium) and s (strong).

Nuclear Magnetic Resonance Spectroscopy

^{1}H, ^{2}H, ^{13}C, ^{19}F, ^{31}P nuclear magnetic resonance (NMR) spectra were recorded on a Bruker Avance NEO 600 or AvanceIII 500 MHz NMR or AV-300 spectrometer in a suitable deuterated solvent. The solvent employed and respective measuring frequencies (reported in MHz) are indicated for each experiment. Chemical shifts are reported with tetramethylsilane (TMS) serving as universal reference of all nuclides and with two or one digits after the comma. The resonance multiplicity is described as s (singlet), d (doublet), t (triplet), q (quadruplet), p

(pentet), hept (heptet), m (multiplet), and b (broad) and combinations thereof. All spectra were recorded at 298 K unless otherwise noted and processed with MestreNova 14.2.3. Multiplicity and coupling constants are reported as observed. The residual solvent signal relative to tetramethylsilane was used as the internal reference in ^{1}H NMR, ^{2}H NMR and ^{13}C NMR spectra (e.g. $CHCl_3$ = 7.26 ppm, $CDHCl_2$= 5.32 ppm)[10,11], which are reported as follows: chemical shift δ in ppm (multiplicity, coupling constant *J* in Hz, number of protons). ^{19}F spectra and ^{31}P NMR spectra were referenced according to Ξ-values (IUPAC recommendations 2008)[12] relative to the internal references set in ^{1}H NMR spectra (e.g. ^{19}F: CCl_3F, ^{31}P: H_3PO_4 each 0.00 ppm). All spectra are broadband decoupled unless otherwise noted.

Mass Spectrometry

Electron impact (EI, 70 eV) mass spectrometry was performed on a Thermo Fisher Scientific Q Exactive GC Orbitrap GC-MS/MS system (LRMS and HRMS). Chemical ionization (CI) mass spectrometry was performed on a Thermo Fisher Scientific Q. Exactive GC Orbitrap GC-MS/MS system (LRMS and HRMS).Electrospray ionization (ESI) was performed on a Thermo Fisher Scientific Q Exactive Plus Orbitrap (LRMS and HRMS). The ionization method and mode of detection employed is indicated for the respective experiment and all masses are reported in atomic mass units divided by elementary charge number (m/z).

Specific Rotations

Specific rotations $[\alpha]_D^T$ were measured on a Rudolph RA AUTOPOL® IV Automatic Polarimeter at the indicated temperature with a sodium lamp (sodium D line, λ = 589 nm). Measurements were performed in an acid resistant 1 mL cell (50 mm length) with concentrations (g/(100 mL)) reported in the corresponding solvent.

High Performance Liquid Chromatography

High performance liquid chromatography (HPLC) was performed on a Shimadzu LC-40AD (SIL-40C XS autosampler, DGU-405 degasser, CTO-40C column oven, SPD-M40 PDA detector, SCL-40 controller, LC-40D XS pump) or Prominence LC-2030C (LC-2030 autosampler, LC-2030/2040 degasser, LC-2030 column oven, LC-2030 PDA detector, LC-2030 pump) using CHIRALPAK® and CHIRALCEL® columns. All solvents used were HPLC-grade solvents purchased from Sigma-Aldrich. The specific column employed and respective solvent mixtures are indicated for each experiment.

Preparative High Performance Liquid Chromatography

Preparative High Performance Liquid Chromatography (prep-HPLC) was performed on a Shimadzu LC-20AP (SIL-20A HT autosampler, DGU-20A 3R degasser, CTO-20AC column over, SPD-20A UV/Vis detector, FRC-10A fraction collector, CBM-20A controller, LC-20AP pump). All solvents used were HPLC-grade solvents purchased from Sigma-Aldrich.

Liquid Chromatography-Mass Spectrometry

Liquid Chromatography-Mass Spectrometry (LC-MS) was performed on a Shimadzu LC-20AD. All solvents used were HPLC-grade solvents purchased from Sigma-Aldrich. The column employed, the respective solvent mixture, and the MS parameters are indicated for each experiment.

Gas Chromatography

Gas chromatography (GC) analyses were performed on an Agilent Technologies 6890N, Agilent Technologies 6890, Agilent Technologies 7890A, Agilent Technologies 7890B (split-mode capillary injection system, flame ionization detector (FID)). The conditions for achiral and chiral separation employed are described in detail for the individual experiments.

Gas Chromatography-Mass spectrometry

Gas Chromatography-Mass Spectrometry analyses were performed on a Thermo Fisher Scientific Q Exactive Orbitrap / Thermo Scientific Trace 1310. The columns and conditions employed are described in detail for the individual experiments.

1.2. Cyclization of neral/citral to isopiperitenol

1.2.1. Evaluation of several Brønsted acid catalysts in the targeted reaction

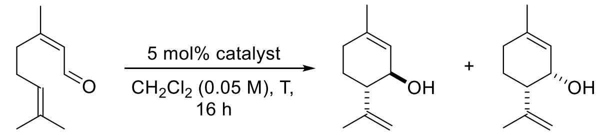

1 *trans*-**2** *cis*-**2**

entry	catalyst	T [°C]	conversion* [%]	yield* [%]	d.r.[†] (*trans*:*cis*)	e.r.[†] (*trans*)	e.r.[†] (*cis*)
1[§]	**HCl**	23	n.d.	<5	-	-	-
2	**TsOH**	23	n.d.	<5	-	-	-
3	**S1**	23	>95	<5	-	-	-
4	**S2**	23	>95	<5	-	-	-
5	**3**	23	>95	<5	-	-	-
6[#]	**S1**	–15	12	<5	-	-	-
7[#]	**S2**	–15	20	<5	-	-	-
8[#]	**3**	–15	12	<5	-	-	-
9	**S3**	23	10	<5	-	-	-
10	**S4**	23	10	<5	-	-	-
11[‡]	**S5**	–40	>95	<5	-	-	-
12[**]	**S6**	–40	36	35	16:1	52:48	42:58
13[**]	**S7**	–40	<5	<5	-	-	-
14[**]	**S8**	–40	52	49	>20:1	14.5:85.5	44:56
15[**]	**S9**	–40	30	13	8:1	38:62	47:53
16[**]	**5**	–40	71	70	>20:1	99.5:0.5	71.5:28.5

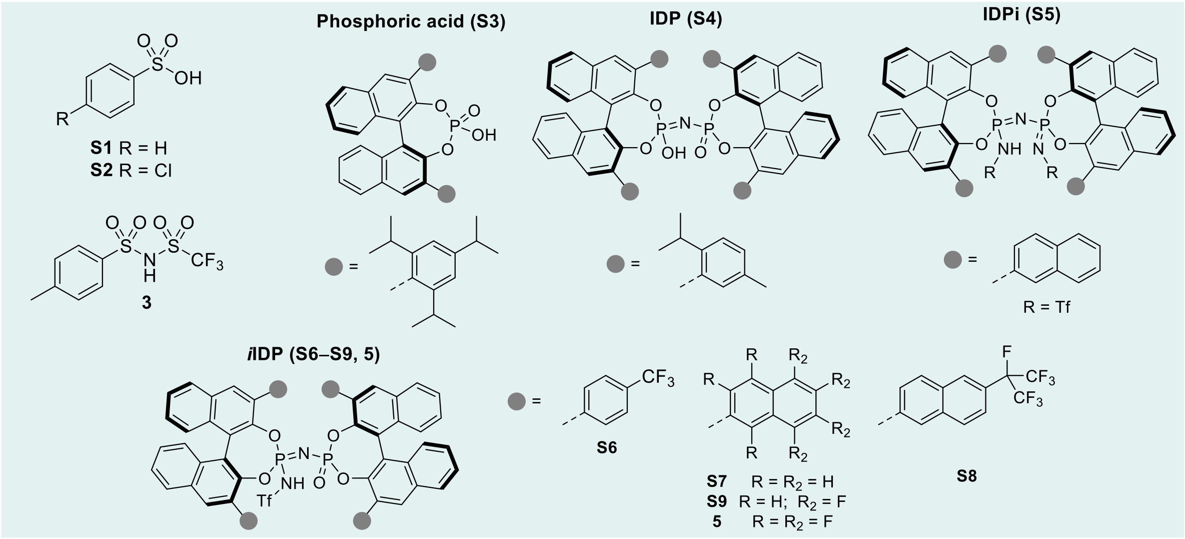

Figure S1: Performance of selected achiral Brønsted acids (BA) and chiral, confined BA catalysts of varying acidity in the targeted transformation. All reactions were performed on a 0.029 mmol scale of neral (Z:E>95:5) with 5 mol% of catalyst.[*]Conversion and yield were determined by ^{1}H NMR spectroscopy using mesitylene as an internal standard. [†]Diastereomeric and enantiomeric ratios were determined by gas chromatography. [§]10 mol% catalyst used. [#]PhMe as solvent. [‡]Lowering the reaction temperature was not beneficial for the formation of the desired product.**Concentration of the reaction is 0.005 M.

1.2.2. Optimization of reaction conditions

*Note: In the following subchapters A–C, the reaction parameters (concentration, catalyst loading, solvent, temperature) were optimized using iIDP catalyst **5** and in the subchapter D–E, the Z:E-ratio of the starting material and the dependence of the catalyst loading on the decomposition of isopiperitenol was investigated.*

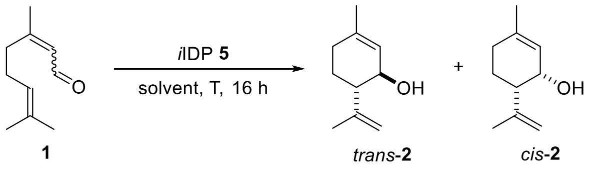

General reaction procedure for the cyclization of neral (GP–1): In an oven-dried vial, *i*IDP catalyst **5** was dissolved and if desired cooled down to the desired reaction temperature. *α,β*-Unsaturated aldehyde **1** (5.0 μL, 0.029 mmol, 1.0 equiv., *Z*:*E*-ratio >95:5 unless stated otherwise) was added in one portion and the reaction was stirred for 16 h. The reaction was quenched with two drops of triethylamine. Mesitylene was added as internal standard and the yield was determined by ^{1}H NMR spectroscopy. After evaporation of the solvent, purification of the crude mixture by preparative TLC (silica gel, 10:1 hexane:EtOAc, partially stained with $KMnO_4$) afforded isopiperitenol (**2**) as an inseparable mixture of diastereoisomers.

A – Concentration

Table S1. Evaluation of different concentrations using optimal *i*IDP catalyst 5 in the targeted transformation. The optimal concentration is highlighted in green. All reactions were performed following *GP–1* using 5 mol% *i*IDP **5** in PhMe at –20 °C.*Yields were determined by ^{1}H NMR spectroscopy using mesitylene as internal standard. †Diastereomeric and enantiomeric ratios were determined by gas chromatography.

concentration [M]	yield* [%]	d.r.† (*trans*:*cis*)	e.r.† (*trans*)	e.r.† (*cis*)
0.025	78	20:1	99:1	62:38
0.05	77	>20:1	99:1	62:38
0.1	37	19:1	98.5:1.5	61.5:38.5
0.25	34	20:1	98.5:1.5	60:40
0.5	19	19:1	98.5:1.5	57.5:42.5

B – Solvent and temperature

Table S2. Evaluation of selected solvents at different temperatures (–60 to 20 °C) using optimal *i*IDP catalyst 5 in the targeted transformation. Optimal solvent/temperature conditions are highlighted in green. All reactions were performed following *GP–1* using 5 mol% *i*IDP **5** and 0.6 mL of solvent.[*]Yields were determined by ^{1}H NMR spectroscopy using mesitylene as internal standard. [†]Diastereomeric and enantiomeric ratios were determined by gas chromatography. [§]*i*IDP catalyst **5** is not fully soluble.

solvent / T [°C]	yield* [%]<br>d.r. (*trans*:*cis*)[†]<br>e.r. (*trans*)[†]				
	CH_2Cl_2	$CHCl_3$	PhMe	Pentane[§]	Et_2O
**–60**	6	<5	<5	-	-
**–50**	43<br>>20:1<br>99.5:0.5	14<br>17:1<br>99:1	9	5	<5
**–40**	67<br>18:1<br>99.5:0.5	35<br>17:1<br>99.5:0.5	21<br>>20:1<br>99:1	12<br>13:1<br>97.5:2.5	<5
**–30**	70<br>16:1<br>99.5:0.5	66<br>17:1<br>99.5:0.5	35<br>>20:1<br>99:1	29<br>12:1<br>97.5:2.5	6
**–20**	40<br>6:1<br>98.5:1.5	73<br>13:1<br>99:1	77<br>>20:1<br>99:1	44<br>15:1<br>98.5:1.5	21<br>10:1<br>96.5:3.5
**–15**	18<br>3:1<br>96:4	63<br>8:1<br>99:1	80<br>17:1<br>99:1	63<br>14:1<br>98.5:1.5	32<br>17:1<br>99.5:0.5
**–10**	8	40<br>5:1<br>98.5:1.5	69<br>14:1<br>99:1	77<br>12:1<br>98.5:1.5	47<br>17:1<br>99:1
**0**	-	8	26<br>5:1<br>97.5:2.5	40<br>7:1<br>97:3	58<br>19:1<br>99:1
**10**	-	<5	-	-	44<br>13:1<br>98.5:1.5
**20**	-	-	-	-	17<br>8:1<br>97.5:2.5

C – Catalyst loading

Table S3. Evaluation of the catalyst loading using optimal *i*IDP catalyst 5 in the targeted transformation. Optimal catalyst loading is highlighted in green. All reactions were performed following *GP–1* using PhMe (0.6 mL) at –20 °C.*Yields were determined by ^{1}H NMR spectroscopy using mesitylene as internal standard. †Diastereomeric and enantiomeric ratios were determined by gas chromatography.

catalyst loading [mol%]	yield* [%]	d.r.† (*trans*:*cis*)	e.r.† (*trans*)	e.r.† (*cis*)
**1**	34	19:1	99.5:0.5	67.5:32.5
**2.5**	63	>20:1	99:1	59.5:40.5
**5**	77	>20:1	99:1	62:38
**10**	84	19:1	99:1	56.5:43.5

D – *Z*:*E*-ratio of the starting material

Table S4. Evaluation of different *Z*:*E*-ratios of the starting material (Neral/Geranial) using optimal *i*IDP catalyst 5 in the targeted transformation. All reactions were performed following *GP–1* using 5 mol% *i*IDP **5** and PhMe (0.05 M) at –20 °C.*Yields were determined by ^{1}H NMR spectroscopy using mesitylene as internal standard. †Diastereomeric and enantiomeric ratios were determined by gas chromatography. §*Z*:*E*-ratios of the starting materials were analyzed by gas chromatography prior to use[1]. The different *Z*:*E*-mixtures were obtained by mixing Neral and Citral.

Z:*E*-ratio§ [%]	yield* [%]		d.r.† (*trans*:*cis*)	e.r.† (*trans*)	*Z*:*E*-ratio§ [%]	yield* [%]		d.r.† (*trans*:*cis*)	e.r.† (*trans*)
	based on SM	based on Neral				based on SM	based on Neral		
**99.9:0.1**	77	77	>20:1	99:1	**66.4:33.6**	50	76	>20:1	99:1
**93.7:6.3**	74	79	20:1	99:1	**61.5:38.5**	47	77	>20:1	99:1
**88.3:11.7**	70	79	>20:1	99:1	**58.8:41.2**	44	75	>20:1	99:1
**84.3:15.7**	67	79	20:1	99:1	**55.4:44.6**	40	72	>20:1	99:1
**80.9:19.1**	61	75	>20:1	99:1	**49.9:50.1**	38	77	>20:1	99:1
**77.5:22.5**	59	76	20:1	99:1	**43.8:56.2**	34	78	>20:1	99:1
**71.7:28.3**	55	76	-	-					

[1] GC-conditions: *Column*: 24.3 m, RTX-5 0.25/0.5df; *Temperature program*: 220/ 50 5/min 150 12/min 320; *Gas*: 0.40 bar (H_2). t_R (Neral) = 15.17 min; t_R (Geranial) = 16.04 min.

E – Dependence of the catalyst loading on the decomposition of isopiperitenol

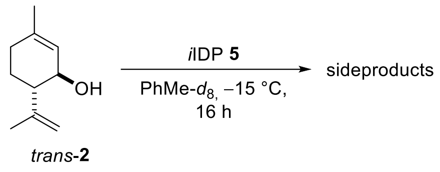

In an oven-dried vial, *i*IDP catalyst **5** was dissolved in PhMe-d_8 (0.6 mL) and cooled down to −15 °C. Isopiperitenol **2** (5.0 µL, 0.031 mmol, 1.0 equiv., d.r. (*trans*:*cis*) = 10:1) was added in one portion and the reaction was stirred for 16 h. The reaction was quenched with two drops of triethylamine. Mesitylene was added as internal standard and the conversion to the sideproducts was determined by ^{1}H NMR spectroscopy.

Table S5. Evaluation of the product decomposition using different catalyst loadings of optimal *i*IDP catalyst 5. *Conversions were determined by ^{1}H NMR spectroscopy using mesitylene as internal standard.

catalyst loading [mol%]	conversion* [%]
0	0
2.5	3.6
5	9.2
10	19
20	30

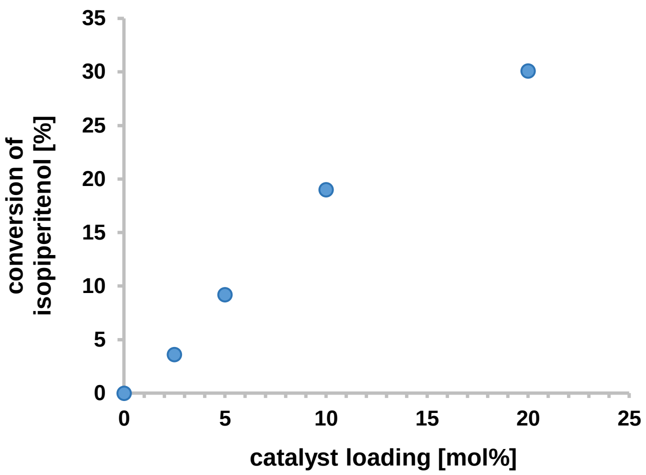

1.2.3. Large-scale reaction of the cyclization of neral

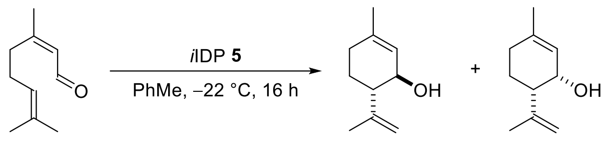

In a flame-dried round-bottom flask under Ar, *i*IDP catalyst **5** (3.1 g, 1.7 mmol, 5 mol%) was dissolved in PhMe (700 mL) and cooled to –22 °C. Neral (**1**) (6.0 mL, 35.0 mmol, 1.0 equiv.) was slowly added and the reaction was stirred for 16 h. The reaction was quenched with trimethylamine (10 mol%) and warmed up to room temperature. The solvent was removed under reduced pressure (40 °C water bath, 40 mbar). Purification by FCC (spherical silica gel, gradient 20:1 to 10:1 pentane:Et_2O) yielded the desired product **2** as colorless oil (4.05 g, 76% yield) and as mixture of diastereoisomers (d.r. (*trans*:*cis*) > 20:1, e.r. (*trans*) = 99:1), $[\alpha]_D^{25} = -19.4$ (c = 0.65, $CHCl_3$)).

Table S6. Selected results from the experiments on larger scale using *i*IDP catalyst 5. All reported yields refer to isolated, chromatographically and spectroscopically pure compounds. † Yield was determined by ^{1}H NMR spectroscopy using mesitylene as internal standard. §*i*IDP catalyst **5** was recovered after the reaction in 95% yield (2.5 g) after purification by FCC and subsequent acidification following *GP–2*.

entry	solvent	scale [mmol]	T [°C]*	cat. load. (mol%)	yield [%]	d.r. (*trans*:*cis*)	e.r. (*trans*)	e.r. (*cis*)
1§	PhMe	29	–18	5	70	19:1	99:1	55:45
2	PhMe	35	–22	5	76	>20:1	99:1	59:41
3	pentane	58	0	0.5	33 (38†)	12:1	98.5:1.5	68.5:31.5

1.3. Supplementary methods

1.3.1. Synthesis of 3,3'-substituted binaphthyl diols

(*S*)-3,3'-bis(2-(5,6,7,8,-tetrafluoro)naphthyl)-[1,1'-binaphthalene]-2,2'-diol (**S10**)

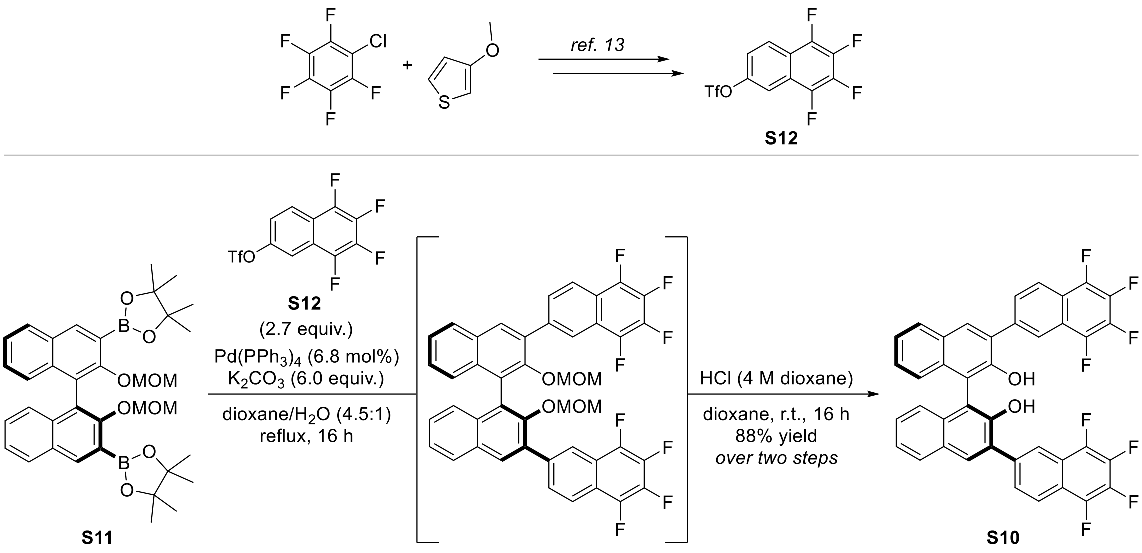

In a flame-dried flask under Ar, (*S*)-2,2'-(2,2'-bis(methoxymethoxy)-[1,1'-binaphthalene]-3,3'-diyl)bis(4,4,5,5-tetramethyl-1,3,2-dioxaborolane) (**S11**) (975 mg, 1.56 mmol, 1.0 equiv.), potassium carbonate (1.3 g, 9.4 mmol, 6.0 equiv.) and 5,6,7,8-tetrafluoronaphthalen-2-yl trifluoromethanesulfonate (**S12**)[13] (1.5 g, 4.2 mmol, 2.7 equiv.) were dissolved in 1,4-dioxane (7 mL) and water (1.6 mL). After degassing the reaction mixture, $Pd(PPh_3)_4$ (123 mg, 0.106 mmol, 6.8 mol%) was subsequently added and the reaction was sealed and was heated to reflux for 16 h. The reaction mixture was cooled to room temperature, exposed to air, diluted with H_2O (15 mL) and CH_2Cl_2 (15 mL). The layers were separated and the aqueous layer was further extracted with CH_2Cl_2 (3x15 mL). The combined organic layers were washed with brine, dried over Na_2SO_4, filtered and concentrated under reduced pressure. The crude reaction product was submitted to MOM-deprotection without prior purification.

A flame-dried flask was charged with crude MOM-protected 3,3'-substituted BINOL and HCl (4 M in dioxane, 2.3 mL, 6.0 equiv.) was added at room temperature. The reaction was stirred until full conversion of the starting material. The solvents were removed under reduced pressure. Purification by automated CC (spherical silica gel, 20:1 hexane:EtOAc) afforded compound **S10** (930 mg, 88% yield) as a pale-yellow solid.

R*f* = 0.50 (10:1 hexane:EtOAc).

m.p. = 257 °C.

^{1}H NMR (501 MHz, CD_2Cl_2) δ_H = 8.44 (s, 2H), 8.20 (s, 2H), 8.17 (d, *J* = 9.0 Hz, 2H), 8.06 (dd, *J* = 8.8, 1.6 Hz, 2H), 8.02 (d, *J* = 8.1 Hz, 2H), 7.47 (ddd, *J* = 8.2, 6.8, 1.2 Hz, 2H), 7.40 (ddd, *J* = 8.2, 6.8, 1.3 Hz, 2H), 7.26 (d, *J* = 8.6 Hz, 2H), 5.51 (s, 2xOH) ppm.

^{13}C NMR (126 MHz, CD_2Cl_2) δ_C = 150.78, 143.82, 143.63, 141.81, 141.64, 139.60, 139.47, 139.34, 139.21, 137.72, 137.59, 137.46, 137.33, 137.17, 133.73, 132.76, 130.07, 130.04, 129.84, 129.17, 128.39, 125.18, 124.45, 120.84, 120.82, 120.80, 120.78, 120.75, 120.34, 120.31, 120.29, 120.27, 120.24, 120.19, 120.14, 120.09, 119.26, 119.22, 119.14, 119.11, 112.53 ppm (no further signals detected or observed).

^{19}F NMR (471 MHz, CD_2Cl_2) δ_F = –151.01 (t, *J* = 16.0 Hz), –151.12 (t, *J* = 16.2 Hz), –159.59 – –159.96 (m) ppm.

HRMS (ESI) m/z calculated for $C_{40}H_{17}F_8O_2$ $[M–H]^-$: 681.1106, found: 681.1105.

IR (neat) = 3517 (br), 1668 (w), 1617 (m), 1494 (s), 1462 (m), 1364 (m), 1235 (m), 1126 (m), 1033 (s), 961 (m), 889 (m), 822 (m), 749 (s), 713 (m), 673 (m) cm^{-1}.

$[\boldsymbol{\alpha}]_D^{25}$ = 22.1 (c = 0.65, $CHCl_3$).

(*S*)-3,3'-bis-(1,3,4,5,6,7,8-heptafluoronaphthalene)-[1,1'-binaphthalene]-2,2'-diol (**S13**)

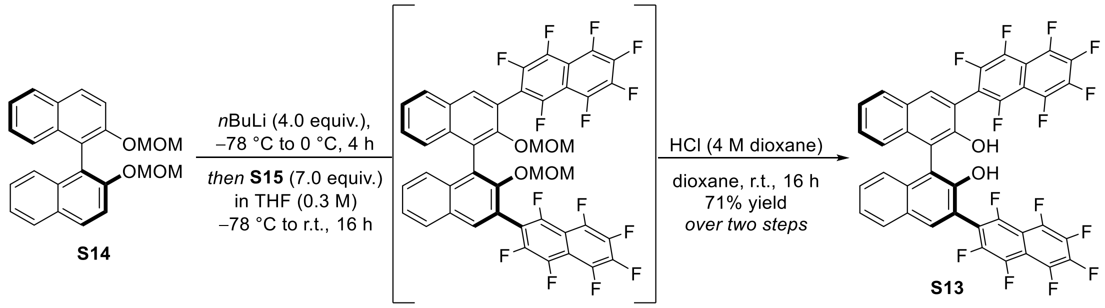

The compound **S13** was prepared according to a modified literature procedure.[14]

In a flame dried round-bottom flask under Ar, MOM-protected (*S*)-BINOL **S14** (5.0 g, 13. mmol, 1.0 equiv.) was dissolved in dry THF (90 mL) and the reaction was cooled to –78 °C. *n*BuLi (2.5 M in hexane, 21.4 mL, 53.4 mmol, 4.0 equiv.) was added dropwise and the reaction mixture was warmed up to 0 °C and was stirred for 4 h. After cooling to –78 °C, a solution of octofluoronaphtalene (**S15**) in THF (25.4 g, 93.5 mmol, 0.3 M in THF, 7.0 equiv.) was slowly added to the reaction mixture. After stirring for 2 h at –78 °C, the reaction mixture was warmed up to room temperature and was stirred overnight until full conversion of the starting material. The reaction mixture was quenched with sat. aq. NH_4Cl and was diluted with Et_2O. The phases

were separated[2] and the aqueous layer was extracted with Et_2O (3x200 mL). The combined organic layers were washed with brine, dried over Na_2SO_4, filtered and concentrated under reduced pressure. The excess of octafluoronaphtalene was carefully removed by sublimation (30 mbar, ~40 °C) and the resulting crude reaction mixture was submitted to MOM-deprotection without further purification.

A flask was charged with crude product and HCl (4 M in dioxane, 20.0 mL, 6.0 equiv.) was added at room temperature. The reaction stirred at room temperature until full conversion of the starting material. The solvents were removed under reduced pressure. Purification by automated CC (spherical silica gel, gradient 20:1 to 1:1 hexane:EtOAc) afforded compound **S13** (7.5 g, 71% yield) as a pale-yellow solid.

R_f = 0.21 (3:1 hexane:CH_2Cl_2);
m.p. = 171 °C;
^{1}H NMR (501 MHz, CD_2Cl_2) δ_H = 8.18 (s, 2H), 8.05 – 7.98 (m, 2H), 7.55 – 7.45 (m, 4H), 7.36 – 7.27 (m, 2H), 5.48 – 5.45 (m, 2xOH) ppm;
^{13}C NMR (126 MHz, CD_2Cl_2) δ_C = 151.42, 151.04, 151.03, 151.01, 149.37, 147.47, 145.43, 143.26, 142.79, 142.23, 142.07, 141.42, 141.30, 141.18, 140.80, 140.64, 140.30, 140.19, 140.06, 139.38, 139.26, 139.14, 138.29, 138.18, 138.04, 134.58, 134.36, 134.33, 134.29, 129.57, 129.36, 129.30, 129.28, 125.46, 125.35, 124.42, 124.40, 124.36, 124.34, 117.04, 117.03, 117.02, 116.44, 116.38, 116.26, 116.21, 116.10, 116.04, 111.99, 111.98, 111.97, 111.96, 111.90, 111.79, 111.68, 108.60, 108.46, 108.36 ppm (no further signals detected or observed);
^{19}F NMR (471 MHz, CD_2Cl_2) δ_F = –118.53 – –118.83 (m), –119.13 (dd, J = 68.7, 18.0 Hz), –135.16 – –135.48 (m), –144.46 – –144.95 (m), –146.80 – –147.18 (m), –149.96 – –150.49 (m), –154.29 (t, J = 18.7 Hz), –156.56 – –156.80 (m) ppm;
HRMS (ESI) m/z calculated for $C_{40}H_{11}F_{14}O_2$ $[M–H]^-$: 789.0541, found: 789.0543;
IR (neat) = 3538 (br), 1651 (m), 1601 (w), 1522 (w), 1494 (m), 1463 (m) 1403 (s), 1213 (m), 1115 (s), 951 (s), 900 (m), 753 (s), 682 (m) cm^{-1};
$[\alpha]_D^{25}$ = –6.48 (c = 0.68, $CHCl_3$).
The analytical and spectroscopic data are in accordance with those reported.[14]

[2] If necessary, the biphasic mixture was filtered through glass wool to accelerate the separation of the layers.

1.3.2. Synthesis of chiral Brønsted acids

General reaction procedure for the acidification of chiral Brønsted acids (GP–2): DOWEX® 50WX8 (hydrogen form, 50–100 mesh) was activated/regenerated prior to use by stirring with 0.15 M aq. H_2SO_4 for 30–60 min. The regenerated resin was packed into a column and rinsed with water (3 CV), MeOH (3 CV) and CH_2Cl_2 (3–5 CV) applying nitrogen pressure. The purified catalyst salt was dissolved in CH_2Cl_2 and loaded onto the column, which was subsequently rinsed with CH_2Cl_2 until no further UV-active material was released. The solvent was evaporated under reduced pressure to furnish the acidified catalysts. The acidified catalysts were dried in high vacuum overnight prior to use. Due to the high confinement of the catalyst and stabilization effects of the inner core structure, especially for the bifunctional *i*IDP catalysts, solvent residues and/or water can remain in the pocket of the catalyst.

(*S,S*)-IDP **4**

(2*S*)-4-(((2*S*)-4-hydroxy-2,6-bis(perfluoronaphthalen-2-yl)-4λ^5-dinaphtho[2,1-d:1',2'-*f*][1,3,2]dioxaphosphepin-4-ylidene)amino)-2,6-bis(perfluoronaphthalen-2-yl)dinaphtho[2,1-d:1',2'-*f*][1,3,2]dioxaphosphepine 4-oxide (**4**)

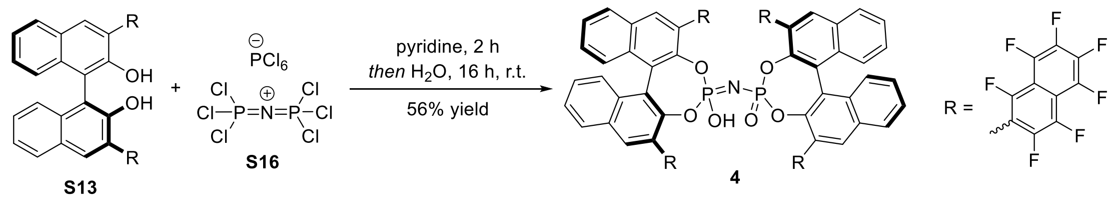

The synthesis is a modified literature procedure for a related compound.[3]

In a flame-dried Schlenk flask under Ar, HCPP (**S16**) (112 mg, 0.210 mmol, 1.0 equiv.) and (*S*)-3,3'-bis-(1,3,4,5,6,7,8-heptafluoro-naphthalene)-[1,1'-binaphthalene]-2,2'-diol (**S13**) (333 mg, 0.421 mmol, 2.0 equiv.) were dissolved in dry pyridine (2 mL). The clear solution slowly formed a precipitate. After 2 h water (0.4 mL) was added to the reaction and the reaction was stirred overnight. The reaction was treated with excess of aq. HCl (10 %) to remove the pyridine. The layers were separated and the organic layer was extracted with CH_2Cl_2 (3x20 mL). The combined organic layers were washed with brine, dried over Na_2SO_4, filtered and concentrated under reduced pressure. The crude was purified by automated CC (spherical silica gel, gradient 20:1 to 3:1 hexane:EtOAc) and the desired catalyst was acidified with

DOWEX® 50WX8 (hydrogen form, 50–100 mesh) following *GP–2*. The acidified catalyst **4** was obtained after evaporation of the solvent and drying in high vacuum overnight as colorless solid (197 mg, 56% yield) and as inseparable mixture of rotamers.

R*f* = 0.44 (3:1 hexane:EtOAc).
m.p. = 297 °C (decomposition).
^{1}H NMR (600 MHz, CD_2Cl_2) δ_H = 8.19 – 7.92 (m, 6H), 7.91 – 7.53 (m, 10H), 7.52 – 7.37 (m, 4H), 6.25 (br. s, OH) ppm.
^{13}C NMR (151 MHz, CD_2Cl_2) δ_C = 151.00, 150.92, 149.31, 149.21, 146.98, 146.90, 146.40, 146.34, 145.35, 145.20, 145.17, 145.15, 145.12, 145.07, 145.00, 144.98, 144.95, 144.92, 144.88, 144.86, 144.74, 144.70, 144.66, 143.99, 143.86, 142.73, 142.34, 141.85, 141.14, 141.05, 140.93, 140.67, 140.14, 139.94, 139.84, 139.45, 139.36, 139.27, 138.25, 138.15, 134.80, 134.41, 134.19, 134.04, 133.89, 133.71, 133.67, 133.53, 133.49, 133.37, 133.34, 133.31, 133.19, 133.07, 132.99, 131.89, 131.71, 131.64, 131.61, 131.38, 129.34, 129.18, 129.04, 129.01, 128.87, 128.82, 128.78, 128.66, 128.56, 128.36, 128.20, 128.04, 127.81, 127.76, 127.56, 127.52, 127.50, 127.39, 127.23, 127.02, 126.96, 126.84, 126.71, 126.61, 123.67, 123.62, 123.53, 123.50, 122.87, 122.80, 122.61, 122.56, 120.00, 119.85, 119.75, 119.67, 119.60, 119.51, 118.01, 115.61, 115.47, 115.25, 111.85, 111.71, 111.53, 111.43, 108.35, 108.27, 107.72, 107.66, 107.57, 107.50 ppm (no further signals observed).
^{19}F NMR (565 MHz, CD_2Cl_2) δ_F = –115.95, –116.07, –116.40, –116.53, –116.66, –117.27, –117.76, –117.79, –117.87, –117.90, –118.00, –118.03, –118.09, –118.12, –118.15, –118.19, –118.22, –118.40, –118.42, –118.43, –118.45, –118.52, –118.55, –118.58, –118.64, –118.67, –118.71, –118.76, –118.79, –119.05, –119.18, –119.29, –119.41, –119.53, –119.74, –119.88, –119.91, –119.94, –119.99, –120.03, –120.06, –120.11, –120.14, –120.20, –120.30, –120.33, –120.52, –120.62, –120.65, –120.74, –120.78, –120.81, –121.06, –121.10, –121.12, –121.19, –121.22, –121.25, –133.76, –133.88, –134.40, –134.73, –134.77, –135.09, –135.12, –135.16, –135.19, –135.27, –135.29, –135.30, –135.32, –135.36, –135.43, –135.60, –135.75, –135.78, –135.81, –135.99, –136.01, –136.03, –136.04, –136.14, –136.16, –136.27, –136.29, –136.35, –136.38, –136.44, –136.60, –136.68, –136.82, –136.84, –136.93, –137.28, –137.57, –137.66, –137.94, –138.07, –138.30, –138.82, –139.04, –139.36, –140.03, –140.31, –142.64, –142.77, –142.98, –143.01, –143.08, –143.17, –143.63, –143.73, –143.75, –143.79, –143.86, –143.89, –143.90, –143.93, –143.99, –144.02, –144.06, –144.09, –144.12, –144.19, –144.22, –144.25, –144.35, –144.36, –144.47, –144.49, –144.76, –144.79, –144.88, –144.91, –144.94, –144.98, –145.00, –145.03, –145.06, –145.10, –145.13, –145.18, –145.33, –145.44, –145.47, –145.51, –145.54, –145.56, –145.59, –145.63, –145.66, –145.74, –145.77, –145.83, –145.86, –145.90,

–145.93, –145.96, –145.99, –146.01, –146.04, –146.09, –146.11, –146.21, –146.31, –146.34, –146.38, –146.45, –146.48, –146.70, –146.81, –146.98, –147.05, –147.08, –147.11, –147.13, –147.15, –147.18, –147.21, –147.24, –147.26, –147.29, –147.31, –147.33, –147.36, –147.39, –147.41, –147.44, –147.46, –147.49, –147.67, –147.69, –147.76, –147.78, –147.81, –147.83, –147.86, –147.89, –147.91, –147.94, –147.96, –148.03, –148.06, –148.13, –148.16, –148.20, –148.23, –148.27, –148.34, –148.37, –148.45, –148.75, –148.78, –148.81, –148.84, –148.88, –148.91, –148.94, –148.97, –149.05, –149.25, –149.34, –149.41, –149.44, –149.48, –149.51, –149.55, –149.58, –150.18, –150.21, –150.24, –150.31, –150.34, –150.36, –150.40, –150.44, –150.47, –151.04, –151.16, –151.22, –151.33, –152.15, –152.19, –152.21, –152.25, –152.28, –152.31, –152.35, –152.38, –152.41, –153.64, –153.68, –153.77, –153.80, –153.92, –154.02, –154.12, –154.21, –154.30, –155.41, –155.68, –155.71, –155.74, –155.78, –155.81, –155.91, –155.97, –156.01, –156.03, –156.06, –156.10, –156.17, –156.20, –156.24, –156.29, –156.32, –156.35, –156.36, –156.47, –156.51, –156.61, –156.78, –157.41, –157.79, –157.82, –157.86, –158.04, –158.09, –158.13, –158.16, –158.34, –158.41, –158.44, –158.48, –158.51, –158.54, –158.80, –158.86, –159.14 ppm (no further signals detected or observed).

^{31}P NMR (243 MHz, CD_2Cl_2) δ_P = 10.41, 10.17, 9.92, 9.69, 9.28, 5.73, 5.36, 5.23, 4.86, 4.25, 4.16, 3.87, 3.78, 3.63, 3.49, 3.34, 3.25, 3.06, 2.91, 2.88, 2.78, 2.69, 2.66, 2.51, 1.86, –1.09, –1.37, –1.60, –1.82 ppm (no further signals detected or observed).

HRMS (ESI) m/z calculated for $C_{80}H_{20}F_{28}N_1O_6P_2$ [M–H]$^-$: 1684.0324, found: 1684.0334.

IR (neat) = 1652 (m), 1603 (w), 1524 (w), 1495 (m), 1463 (m), 1404 (s), 1325 (m), 1188 (m), 1116 (m), 949 (s), 750 (s), 665 (m), 573 (m) cm^{-1}.

$[\boldsymbol{\alpha}]_{\boldsymbol{D}}^{\mathbf{25}}$ = 123.72 (c = 0.61, $CHCl_3$).

LC-MS (50 mm Zorbax 300SB-C8, 4.6 mm i.d., MeCN / 1% TFA = 75:25, 1.0 mL/min, 5.9 MPa, 308 K, UV 254 nm): t_R = 5.65 (99.60%); m/z = 1684 ([M–H]$^-$).

(*S*,*S*)-*i*IDP **S7**

N-(4-((2,6-di(naphthalen-2-yl)-4-oxidodinaphtho[2,1-d:1',2'-*f*][1,3,2]dioxaphosphepin-4-yl)imino)-2,6-di(naphthalen-2-yl)-4λ^5-dinaphtho[2,1-d:1',2'-*f*][1,3,2]dioxaphosphepin-4-yl)-1,1,1-trifluoromethanesulfonamide (**S7**)

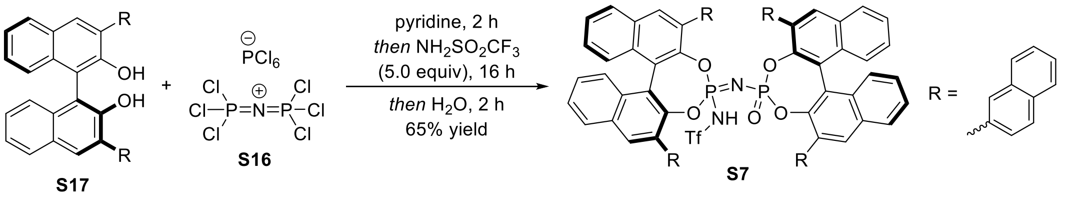

The synthesis is a modified literature procedure for a related compound.[3]

In a flame-dried Schlenk flask under Ar, HCPP (**S16**) (197 mg, 0.37 mmol, 1.0 equiv.) and (*S*)-[2,3':1',1'':3'',2'''-quaternaphthalene]-2',2''-diol (**S17**) (398 mg, 0.74 mmol, 2.0 equiv.) were dissolved in dry pyridine (4 mL). The clear solution slowly formed a precipitate and after 2 h trifluoromethane sulfonamide (275 mg, 1.85 mmol, 5.0 equiv) was added to the reaction. After 16 h, water (0.4 mL) was added to the reaction and was stirred for further 2 h. The reaction was treated with excess of aq. HCl (10 %) to remove the pyridine. The layers were separated and the organic layer was extracted with CH_2Cl_2 (3x30 mL). The combined organic layers were washed with brine, dried over Na_2SO_4, filtered and concentrated under reduced pressure. The crude was purified by FCC (silica gel, 4:1:1 hexane:EtOAc:CH_2Cl_2) and the desired catalyst was acidified with DOWEX® 50WX8 (hydrogen form, 50–100 mesh) following *GP–2*. The acidified catalyst **S7** was obtained after evaporation of the solvent and drying in high vacuum overnight as a pale yellow solid (318 mg, 65% yield).

$\mathbf{R_f}$ = 0.42 (2:1:1 hexane:CH_2Cl_2:EtOAc).
m.p. = 290 °C (decomposition).
^{1}**H NMR** (501 MHz, CD_2Cl_2) δ_H = 8.21 (d, *J* = 8.2 Hz, 2H), 8.16 (d, *J* = 11.1 Hz, 2H), 8.10 (t, *J* = 7.6 Hz, 2H), 8.06 (dd, *J* = 11.1, 8.6 Hz, 2H), 7.96 – 7.86 (m, 3H), 7.85 – 7.77 (m, 2H), 7.76 – 7.72 (m, 3H), 7.69 – 7.54 (m, 11H), 7.51 (d, *J* = 9.6 Hz, 2H), 7.48 – 7.31 (m, 16H), 6.80 (dd, *J* = 8.6, 1.8 Hz, 1H), 6.75 (dd, *J* = 8.5, 1.8 Hz, 1H), 6.42 (dd, *J* = 8.5, 1.8 Hz, 1H), 6.32 (dd, *J* = 8.5, 1.8 Hz, 1H) ppm.
^{13}**C NMR** (126 MHz, CD_2Cl_2) δ_C = 144.86, 144.78, 144.53, 144.50, 144.45, 144.43, 143.30, 143.23, 134.27, 134.25, 134.17, 134.15, 133.98, 133.95, 133.92, 133.89, 133.87, 133.72, 133.63, 133.59, 133.46, 133.35, 133.19, 133.16, 132.91, 132.74, 132.37, 132.33, 132.28, 132.21, 132.15, 131.86, 131.63, 131.24, 129.61, 129.55, 129.16, 129.12, 129.10, 129.07,

128.88, 128.75, 128.67, 128.50, 128.38, 128.06, 128.04, 127.96, 127.91, 127.86, 127.76, 127.73, 127.69, 127.65, 127.56, 127.49, 127.44, 127.36, 127.28, 127.22, 127.15, 127.12, 126.96, 126.92, 126.89, 126.77, 126.64, 126.58, 126.43, 126.35, 126.25, 126.13, 125.89, 124.17, 124.15, 123.85, 123.83, 123.07, 123.04, 122.74, 122.72, 122.27, 122.25, 120.52, 120.49, 117.98, 117.95, 115.43, 115.40 ppm (no further signals detected or observed).

^{19}F NMR (471 MHz, CD_2Cl_2) δ_F = –80.41 ppm.

^{31}P NMR (203 MHz, CD_2Cl_2) δ_P = –5.77 (d, J = 127.2 Hz), –10.92 (d, J = 126.5 Hz) ppm.

HRMS (ESI) m/z calculated for $C_{81}H_{48}F_3N_2O_7P_2S_1$ $[M–H]^-$: 1311.2615, found: 1311.2615;

IR (neat) = 3053 (br), 1596 (w), 1496 (w), 1441 (w), 1398 (m), 1321 (m), 1197 (s), 1185 (s), 1149 (m), 982 (m), 953 (m), 889 (m), 854 (m), 816 (m), 745 (s), 474 (s) cm^{-1}.

$[\boldsymbol{\alpha}]_D^{25}$ = 428.89 (c = 0.57, $CHCl_3$).

LC-MS (50 mm Zorbax SB300-C8, 3.5 µm, 4.6 mm i.d., MeCN / 1% TFA = 70:30, 1.0 mL/min, 7.5 MPa, 308 K, UV 254 nm): t_R = 5.62 (97.78%), m/z = 1311 ($[M–H]^-$).

(*S*,*S*)-*i*IDP **S9**

1,1,1-trifluoro-*N*-(4-((4-oxido-2,6-bis(5,6,7,8-tetrafluoronaphthalen-2-yl)dinaphtho[2,1-d:1',2'-*f*][1,3,2]dioxaphosphepin-4-yl)imino)-2,6-bis(5,6,7,8-tetrafluoronaphthalen-2-yl)-4λ^5-dinaphtho[2,1-d:1',2'-*f*][1,3,2]dioxaphosphepin-4-yl)methanesulfonamide (**S9**)

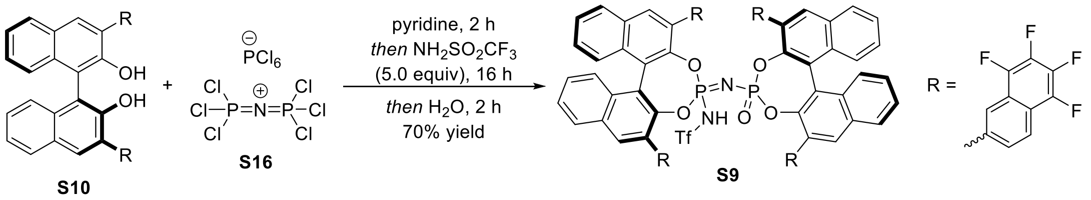

The synthesis is a modified literature procedure for a related compound.[3]

In a flame-dried Schlenk flask under Ar, HCPP (**S16**) (60.4 mg, 0.114 mmol, 1.0 equiv.) and (*S*)-3,3'-bis(2-(5,6,7,8,-tetrafluoro)naphthyl)-[1,1'-binaphthalene]-2,2'-diol (**S10**) (155 mg, 0.227 mmol, 2.0 equiv.) were dissolved in dry pyridine (1.25 mL). The clear solution slowly formed a precipitate and after 2 h trifluoromethane sulfonamide (84.6 mg, 0.567 mmol, 5.0 equiv.) was added to the reaction. After 16 h, water (0.15 mL) was added to the reaction and stirred for further 2 h. The reaction was treated with excess of aq. HCl (10 %) to remove the pyridine. The layers were separated and the organic layer was extracted with CH_2Cl_2 (3x20 mL). The combined organic layers were washed with brine, dried over Na_2SO_4, filtered and concentrated under reduced pressure. The crude was purified by automated CC (spherical

silica gel, gradient 10:1 to 3:1 hexane:EtOAc) and the desired catalyst was acidified with DOWEX® 50WX8 (hydrogen form, 50–100 mesh) following *GP–2*. The acidified catalyst **S9** was obtained after evaporation of the solvent and drying in high vacuum overnight as a colorless solid (128 mg, 70% yield).

R*f* = 0.42 (3:1 hexane:EtOAc).
m.p. = 230 °C (decomposition).
^{1}H NMR (501 MHz, CD_2Cl_2) δ_H = 8.43 (br. s, NH), 8.28 (s, 1H), 8.20 (s, 1H), 8.16 – 8.10 (m, 2H), 8.08 (dd, *J* = 8.3, 5.2 Hz, 2H), 7.90 (q, *J* = 8.0 Hz, 2H), 7.88 – 7.82 (m, 4H), 7.80 – 7.73 (m, 4H), 7.71 (d, *J* = 8.9 Hz, 1H), 7.69 – 7.58 (m, 4H), 7.50 (d, *J* = 8.8 Hz, 1H), 7.49 – 7.42 (m, 3H), 7.40 (d, *J* = 8.5 Hz, 1H), 7.35 (s, 1H), 7.28 (s, 1H), 6.81 (d, *J* = 8.8 Hz, 1H), 6.73 (d, *J* = 8.8 Hz, 1H), 6.63 (dd, *J* = 8.6, 3.3 Hz, 2H) ppm.
^{13}C NMR (126 MHz, CD_2Cl_2) δ_C = 143.87, 143.65, 142.47, 141.36, 139.28, 137.29, 135.82, 135.81, 135.59, 135.57, 135.52, 135.51, 135.24, 135.22, 135.19, 133.09, 132.69, 132.68, 132.57, 132.53, 132.48, 132.46, 132.45, 132.43, 132.16, 132.13, 132.09, 131.93, 131.88, 131.60, 131.51, 131.49, 129.86, 129.76, 129.71, 129.52, 129.44, 129.36, 129.31, 128.79, 128.69, 128.47, 128.41, 128.39, 128.37, 128.14, 127.93, 127.76, 127.47, 127.38, 127.23, 127.13, 124.15, 124.12, 123.83, 123.81, 122.91, 122.89, 122.34, 122.33, 120.96, 120.89, 120.79, 120.60, 120.56, 120.12, 120.04, 119.97, 119.76, 119.74, 119.65, 119.62, 119.60, 119.04, 119.01, 118.92 ppm (no further signals detected or observed).
^{19}F NMR (471 MHz, CD_2Cl_2) δ_F = –80.95, –149.80 – –150.13 (m), –150.81 – –151.14 (m), –151.34 (t, *J* = 17.0 Hz), –151.45 (t, *J* = 16.7 Hz), –158.71 (t, *J* = 18.5 Hz), –159.12 – –159.31 (m), –159.31 – –159.51 (m), –159.68 – –159.86 (m) ppm (no further signals detected or observed).
^{31}P NMR (203 MHz, CD_2Cl_2) δ_P = –4.57 (d, *J* = 123.8 Hz), –10.61 (d, *J* = 123.9 Hz) ppm.
HRMS (ESI) m/z calculated for $C_{81}H_{32}F_{19}N_2O_7P_2S_1$ $[M–H]^-$: 1599.1108, found: 1599.1126.
IR (neat) = 1668 (w), 1618 (m), 1515 (m), 1495 (s), 1462 (m), 1403 (m), 1362 (m), 1325 (m), 1242 (w), 1187 (s), 1125 (m), 1035 (s), 955 (s), 888 (m), 823 (m), 750 (m), 562 (m) cm^{-1}.
$[\alpha]_D^{25}$ = 333.39 (c = 0.64, $CHCl_3$).

(*S*,*S*)-*i*IDP **5**

1,1,1-trifluoro-N-((2*S*)-4-(((2*R*)-4-oxido-2,6-bis(perfluoronaphthalen-2-yl)dinaphtho[2,1-*d*:1',2'-*f*][1,3,2]dioxaphosphepin-4-yl)imino)-2,6-bis(perfluoronaphthalen-2-yl)-4λ^5-dinaphtho[2,1-*d*:1',2'-*f*][1,3,2]dioxaphosphepin-4-yl)methanesulfonamide (**5**)

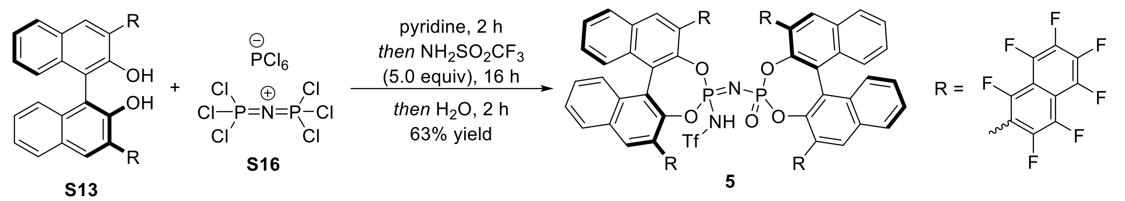

The synthesis is a modified literature procedure for a related compound.[3]

In a flame-dried Schlenk flask under Ar, HCPP (**S16**) (286 mg, 0.537 mmol, 1.0 equiv.) and (*S*)-3,3'-bis-(1,3,4,5,6,7,8-heptafluoro-naphthalene)-[1,1'-binaphthalene]-2,2'-diol (**S13**) (851 mg, 1.08 mmol, 2.0 equiv.) were dissolved in dry pyridine (5.5 mL). The clear solution slowly formed a precipitate and after 2 h trifluoromethane sulfonamide (437 mg, 2.93 mmol, 5.5 equiv) was added to the reaction. After 16 h, water (0.5 mL) was added to the reaction and stirred for further 2 h. The reaction was treated with excess of aq. HCl (10 %) to remove the pyridine. The layers were separated and the organic layer was extracted with CH_2Cl_2 (3x100 mL). The combined organic layers were washed with brine, dried over Na_2SO_4, filtered and concentrated under reduced pressure. The crude was purified by FCC (silica gel, hexane:EtOAc:CH_2Cl_2, 3:1:1) and the desired catalyst was acidified with DOWEX® 50WX8 (hydrogen form, 50–100 mesh) following *GP–2*. The acidified catalyst **5** was obtained after evaporation of the solvent and drying in high vacuum overnight as a brown-yellow solid (618 mg, 63% yield) and as an inseparable mixture of rotameric compounds.

R*f* = 0.37 (3:1 hexane:EtOAc).

m.p. = 277 °C (decomposition).

^{1}H NMR (500 MHz, CD_2Cl_2) δ_H = 8.21 – 7.97 (m, 6H), 7.92 – 7.58 (m, 11H), 7.53 (q, *J* = 8.2 Hz, 1H), 7.50 – 7.37 (m, 3H) ppm.

^{13}C NMR (151 MHz, CD_2Cl_2) δ_C = 151.25, 150.52, 149.22, 146.34, 146.25, 146.07, 144.71, 144.68, 144.66, 144.64, 144.60, 144.54, 144.51, 144.48, 144.45, 144.43, 144.40, 144.38, 144.31, 144.22, 144.21, 144.14, 144.06, 144.05, 144.03, 143.95, 143.92, 143.89, 143.09, 143.03, 142.92, 142.72, 142.65, 142.04, 141.41, 141.29, 141.04, 140.92, 140.35, 140.24, 140.16, 140.03, 139.38, 139.26, 138.15, 135.11, 134.98, 134.95, 134.69, 134.63, 134.47, 134.19, 134.12, 133.94, 133.81, 133.72, 133.64, 133.57, 133.51, 133.26, 133.17, 133.12, 132.18, 132.06, 131.99, 131.93, 129.45, 129.41, 129.38, 129.08, 129.01, 128.96, 128.94, 128.79, 128.73, 128.61, 128.56, 128.51, 128.46, 128.37, 128.33, 128.18, 128.11, 128.03, 127.94, 127.88, 127.78, 127.72, 127.66, 127.60, 127.42, 127.39, 127.34, 127.30, 127.27, 127.18, 127.12, 127.09, 127.02, 123.31, 123.29, 123.23, 123.13, 123.10, 123.08, 123.06, 123.03, 123.01, 122.97, 122.94, 122.90, 122.88, 122.84, 122.75, 122.74, 122.68, 122.65,

122.38, 120.39, 119.57, 119.52, 119.45, 119.22, 119.10, 119.02, 114.39, 114.18, 114.01, 112.37, 112.26, 112.16, 112.03, 108.90, 108.81, 108.69, 107.91, 107.82, 107.68 ppm (no further signals detected or observed).

^{19}F NMR (471 MHz, CD_2Cl_2) δ_F = –80.76, –80.79, –80.82, –80.84, –80.88, –80.95, –80.96, –81.02, –81.03, –81.10, –81.11, –81.17, –81.22, –81.25, –81.26, –81.30, –116.31, –116.35, –116.46, –116.50, –116.63, –116.70, –116.74, –116.79, –116.85, –116.89, –116.93, –117.31, –117.40, –117.44, –117.55, –117.59, –117.64, –117.67, –117.71, –117.74, –117.78, –117.83, –117.87, –117.93, –117.98, –118.02, –118.08, –118.11, –118.16, –118.22, –118.26, –118.31, –118.36, –118.40, –118.71, –118.86, –118.99, –119.11, –119.46, –119.61, –119.65, –119.70, –119.75, –119.79, –120.79, –120.93, –121.02, –121.16, –121.32, –121.37, –121.42, –121.47, –121.52, –121.56, –121.69, –121.73, –121.83, –121.87, –122.08, –122.23, –122.39, –122.55, –126.56, –126.75, –127.68, –127.88, –128.71, –128.88, –133.97, –134.01, –134.07, –134.11, –134.18, –134.20, –134.33, –134.47, –134.82, –135.00, –135.04, –135.31, –135.39, –135.43, –135.54, –135.82, –135.85, –135.95, –136.02, –136.06, –136.17, –136.39, –136.42, –136.70, –137.16, –137.29, –137.33, –137.38, –137.56, –137.69, –137.73, –137.88, –138.07, –138.11, –138.39, –138.43, –138.53, –138.56, –138.71, –138.90, –139.12, –139.95, –140.15, –140.20, –142.54, –142.74, –142.78, –142.94, –143.08, –143.26, –143.48, –143.52, –143.55, –143.58, –143.63, –143.66, –143.70, –143.73, –143.77, –143.80, –143.84, –143.87, –143.94, –143.97, –144.41, –144.45, –144.48, –144.56, –144.59, –144.63, –144.72, –144.75, –144.79, –144.87, –144.90, –144.99, –145.02, –145.06, –145.10, –145.14, –145.17, –145.21, –145.24, –145.35, –145.38, –145.41, –145.45, –145.49, –145.53, –145.56, –145.60, –145.73, –145.77, –145.80, –145.84, –145.88, –145.91, –145.95, –145.99, –146.16, –146.20, –146.24, –146.31, –146.37, –146.41, –146.44, –146.49, –146.53, –146.57, –146.89, –146.99, –147.02, –147.05, –147.08, –147.11, –147.15, –147.19, –147.23, –147.27, –147.31, –147.34, –147.40, –147.49, –147.53, –147.56, –147.69, –147.73, –147.76, –147.85, –147.89, –147.96, –148.00, –148.04, –148.08, –148.09, –148.14, –148.18, –148.22, –148.26, –148.30, –148.37, –148.41, –148.45, –148.50, –148.54, –148.58, –148.62, –148.67, –148.70, –148.83, –148.99, –149.12, –149.28, –149.32, –149.36, –149.40, –149.44, –149.48, –149.53, –149.77, –149.83, –149.90, –150.06, –150.18, –150.73, –150.88, –150.91, –150.95, –151.04, –151.07, –151.28, –152.10, –152.14, –152.18, –152.22, –152.26, –152.30, –152.34, –152.38, –153.27, –153.30, –153.38, –153.42, –153.45, –153.50, –153.54, –153.57, –153.64, –153.68, –153.72, –153.77, –153.81, –153.88, –153.93, –153.97, –154.00, –154.19, –154.39, –154.45, –154.53, –154.75, –154.79, –154.83, –154.87, –154.93, –154.96, –155.00, –155.29, –155.43, –155.47, –155.52, –155.56, –155.79, –155.83, –155.88, –155.98, –156.03, –156.07, –156.13, –156.26, –156.30, –156.35, –156.42, –156.53,

–156.57, –156.61, –156.66, –156.80, –157.03, –157.07, –157.11, –157.17, –157.37, –157.47, –157.51, –157.62, –157.74, –157.86, –158.29 ppm (no further signals detected or observed).
^{31}P NMR (203 MHz, CD_2Cl_2) δ_P = 4.34, 4.14, 4.03, 3.94, 3.86, 3.70, 3.62, 3.52, 3.44, 3.39, 3.25, 3.19, 3.12, 3.04, 2.90, 2.83, 2.76, 2.68, 2.62, 2.56, 2.34, 2.20, 2.08, 1.88, 1.56, 1.41, 0.94, 0.89, 0.41, 0.34, –0.34, –0.96, –1.45, –1.71, –2.02, –2.20, –2.47, –2.80, –2.95, –3.21, –3.29, –3.44, –3.61, –3.70, –3.91, –4.02, –4.10, –4.40, –4.73, –4.93, –4.98, –5.24, –5.50, –5.90, –6.11 ppm (no further signals detected or observed).
HRMS (ESI) m/z calculated for $C_{81}H_{20}F_{31}N_2O_7P_2S_1$ $[M–H]^-$: 1814.9977, found: 1814.9977.
IR (neat) = 1652 (m), 1605 (w), 1525 (w), 1495 (m), 1464 (m), 1406 (s), 1323 (m), 1231 (w), 1187 (m), 1116 (m), 1068 (m), 951 (s), 895 (m), 750 (m), 572 (s) cm^{-1}.
$[\boldsymbol{\alpha}]_{\boldsymbol{D}}^{\mathbf{25}}$(*S*,*S*)= +109.95 (c = 0.61, $CHCl_3$), $[\boldsymbol{\alpha}]_{\boldsymbol{D}}^{\mathbf{25}}$(*R*,*R*) = –107.06 (c = 0.61, $CHCl_3$).
LC-MS (50 mm Zorbax 300SB-C8, 3.5 μm, 4.6 mm i.d.): MeCN / 1% TFA = 80:20, 1.0 mL/min, 5.2 MPa, 308 K, UV 220 nm): t_{Rot1} = 2.80 (29.90%), t_{Rot2} = 2.91 (69.45%), m/z = 1816 ($[M–H]^-$), purity = 99.4%.

(*S*,*S*)-IDPi **6**

N-((2*S*)-2,6-bis(perfluoronaphthalen-2-yl)-4-((2-((*S*)-perfluoronaphthalen-2-yl)-6-(perfluoronaphthalen-2-yl)-4-(((trifluoromethyl)sulfonyl)imino)-4λ^5-dinaphtho[2,1-*d*:1',2'-*f*][1,3,2]dioxaphosphepin-4-yl)imino)-4λ^5-dinaphtho[2,1-*d*:1',2'-*f*][1,3,2]dioxaphosphepin-4-yl)-1,1,1-trifluoromethanesulfonamide

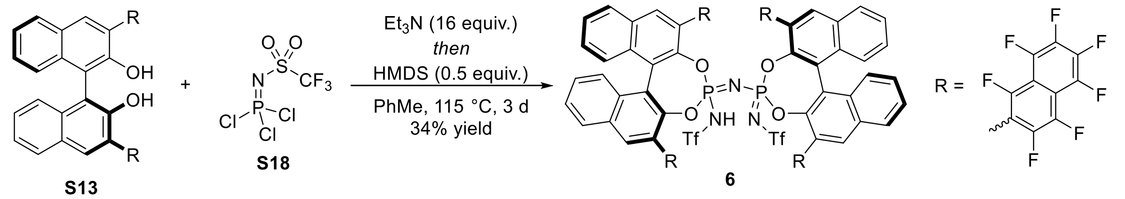

The synthesis is a modified literature procedure for a related compound.[15]

In a flame-dried Schlenk flask under Ar, phosphazene reagent (**S18**) (151 mg, 0.532 mmol, 1.05 equiv.) and (*S*)-3,3'-bis-(1,3,4,5,6,7,8-heptafluoro-naphthalene)-[1,1'-binaphthalene]-2,2'-diol (**S13**) (400 mg, 0.506 mmol, 1.0 equiv.) were dissolved in PhMe (3 mL). Triethylamine (1.1 ml, 8.10 mmol, 16 equiv.) was subsequently added. After 1 h, HMDS (52.8 μL, 0.253 mmol, 0.5 equiv.) was added. The reaction was sealed and was heated to 120 °C over three days. After cooling down to room temperate, the reaction was treated with excess of aq. HCl (10 %). The layers were separated and the organic layer was extracted with CH_2Cl_2

(3x30 mL). The combined organic layers were washed with brine, dried over Na_2SO_4, filtered and concentrated under reduced pressure. The crude was purified by automated CC (spherical silica gel, gradient 10:1 to 1:1 hexane:EtOAc) and the desired catalyst was acidified with DOWEX® 50WX8 (hydrogen form, 50–100 mesh) following *GP–2*. The acidified catalyst **6** was obtained after evaporation of the solvent and drying in high vacuum overnight as orange-brown solid (170 mg, 34% yield) and as inseparable mixture of rotamers.

R*f1* = 0.26 (2:1 hexane:EtOAc), **R*f2*** = 0.12 (2:1 hexane:EtOAc).
m.p. = 278 °C (decomposition).
^{1}H NMR (501 MHz, CD_2Cl_2) δ_H = 8.23 – 8.00 (m, 6H), 7.97 – 7.56 (m, 10H), 7.54 – 7.40 (m, 4H), 7.36 (br. s., NH) ppm.
^{13}C NMR (126 MHz, CD_2Cl_2) δ_C = 151.40, 149.36, 149.18, 145.91, 145.06, 144.54, 144.20, 143.96, 143.14, 141.93, 141.05, 139.87, 139.30, 137.94, 135.17, 135.14, 135.12, 134.94, 134.85, 134.35, 134.23, 133.99, 133.80, 133.71, 133.59, 133.52, 133.46, 133.31, 133.10, 133.06, 133.01, 132.26, 132.21, 132.18, 132.09, 132.06, 132.01, 131.82, 129.43, 129.39, 129.37, 129.28, 129.22, 129.17, 129.11, 129.08, 128.99, 128.94, 128.90, 128.80, 128.75, 128.57, 128.50, 128.48, 128.42, 128.29, 128.18, 128.12, 127.86, 127.80, 127.76, 127.67, 127.65, 127.61, 127.56, 127.49, 127.45, 127.36, 127.32, 127.25, 126.98, 123.14, 123.04, 123.00, 122.82, 122.67, 122.60, 122.28, 122.22, 120.43, 120.12, 119.77, 119.44, 119.37, 119.20, 119.03, 118.98, 117.89, 117.64, 117.57, 117.49, 114.22, 112.24, 112.01, 111.83, 111.57, 108.78, 108.65, 108.43, 108.09, 107.98, 107.89 ppm (no further signals detected or observed).
^{19}F NMR (471 MHz, CD_2Cl_2) δ_F = –78.94, –79.02, –79.04, –79.17, –79.22, –79.23, –79.32, –79.37, –79.40, –79.50, –79.53, –79.55, –79.64, –79.70, –79.73, –79.80, –79.82, –79.86, –79.91, –79.94, –80.11, –116.28, –116.38, –116.43, –116.47, –116.52, –116.58, –116.62, –117.46, -117.51, –117.56, –117.60, –117.66, –117.70, –118.44, –118.59, –118.72, –118.75, –118.88, -119.22, –119.26, –119.36, –119.40, –119.51, –119.66, –119.79, –119.98, –120.02, –120.13, -120.17, –120.33, –120.37, –120.48, –120.52, –120.57, –120.73, –120.76, –120.91, –121.16, -121.31, –121.91, –121.95, –122.05, –122.09, –122.20, –122.23, –122.34, –122.38, –122.51, -122.66, –123.13, –123.16, –123.23, –123.27, –123.32, –123.38, –123.41, –128.26, –128.40, –129.49, –134.66, –134.77, –134.84, –135.32, –135.56, –135.75, –135.93, –136.16, –136.27, –136.69, –136.85, –137.03, –137.06, –137.16, –137.33, –137.52, –137.59, –137.63, –137.82, –137.93, –137.97, –138.52, –139.01, –139.28, –139.38, –139.41, –139.57, –139.59, –140.03, –140.06, –140.32, –143.17, –143.35, –143.44, –143.47, –143.51, –143.57, –143.61, –143.65, –143.68, –143.83, –144.01, –144.05, –144.08, –144.12, –144.15, –144.19, –144.23,

–144.27, –144.46, –144.49, –144.53, –144.61, –144.65, –144.68, –144.79, –144.83, –144.86, –145.24, –145.28, –145.31, –145.48, –145.51, –145.55, –145.62, –145.66, –145.70, –145.74, –145.77, –145.85, –145.89, –145.92, –146.22, –146.26, –146.29, –146.37, –146.40, –146.44, –146.66, –146.69, –146.73, –146.80, –146.84, –146.87, –146.91, –146.95, –146.98, –147.05, –147.09, –147.12, –147.33, –147.36, –147.42, –147.46, –147.50, –147.54, –147.57, –147.60, –147.64, –147.67, –147.73, –147.76, –147.79, –147.91, –147.95, –147.98, –148.01, –148.04, –148.10, –148.13, –148.26, –148.28, –148.31, –148.33, –148.37, –148.40, –148.43, –148.46, –148.74, –148.84, –148.88, –148.92, –148.96, –149.00, –149.04, –149.24, –149.28, –149.32, –149.36, –149.40, –149.44, –149.47, –149.53, –149.57, –149.61, –149.69, –149.73, –149.82, –150.22, –150.35, –150.39, –150.53, –150.69, –150.82, –151.29, –151.43, –151.57, –151.84, –151.88, –151.92, –151.96, –152.00, –152.04, –152.08, –152.12, –152.16, –154.00, –154.04, –154.08, –154.13, –154.51, –154.82, –154.93, –154.97, –155.02, –155.07, –155.10, –155.14, –155.18, –155.22, –155.26, –155.30, –155.34, –155.40, –155.43, –155.48, –155.61, –155.65, –155.68, –155.84, –155.88, –155.91, –156.01, –156.04, –156.08, –156.45, –156.77, –157.18, –157.32, –157.35, –157.39, –157.48, –157.51, –157.73, –157.93, –158.18, –158.33, –158.60, –158.97, –159.46, –159.60, –159.65, –159.69, –160.09 ppm (no further signals detected or observed).

^{31}P NMR (203 MHz, CD_2Cl_2) δ_P = 3.52, 3.23, 1.16, –0.89, –1.87, –2.06, –2.19, –2.43, –3.49, –3.74, –4.05, –4.53 ppm.

HRMS (ESI) m/z calculated for $C_{82}H_{20}F_{34}N_3O_8P_2S_2$ [M–H]$^-$: 1945.9630, found: 1945.9639; **IR (neat)** = 1653 (m), 1606 (w), 1526 (w), 1496 (m), 1465 (m), 1406 (s), 1300 (m), 1186 (m), 1152 (m), 1115 (m), 952 (s), 895 (m), 750 (m), 611 (m), 572 (s), 502 (m) cm^{-1}.

$[\boldsymbol{\alpha}]_{\boldsymbol{D}}^{\mathbf{25}}$ = 124.08 (c = 0.49, $CHCl_3$).

LC-MS (50 mm Zorbax 300SB-C8, 4.8 mm i.d. MeCN / 1% TFA = 80:20, 1.0 mL/min, 5.4 MPa, 308 K, UV 254 nm): t_{Rot1} = 2.39 (11.78%), t_{Rot2} = 2.53 (47.71%), t_{Rot3} = 2.78 (35.78%), m/z = 1946 ([M–H]$^-$), purity = 95.3%.

1.3.3. Synthesis of racemic and enantiomerically pure isopiperitenol

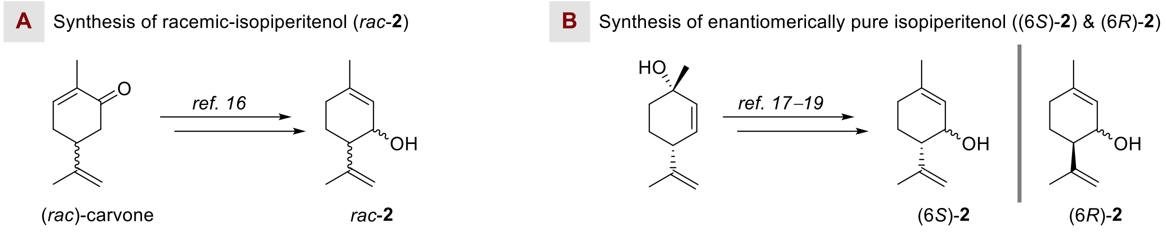

rac-isopiperitenol (*rac*-**2**)

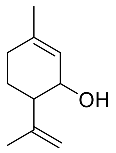

The compound *rac*-**2** was prepared according to the literature procedure.[16] The desired product (*rac*)-**2** was obtained as a colorless liquid (970 mg, 20% yield over four steps) and as inseparable mixture of diastereoisomers (d.r. (*trans*:*cis*) = 1.3:1; e.r. (*trans*) = 51:49; e.r. (*cis*) = 51:49).

R*f* = 0.20 (10:1 hexane:EtOAc);

^{1}H NMR (600 MHz, CD_2Cl_2, *trans* and *cis*) δ_H = 5.63 (ddq, *J* = 5.0, 2.8, 1.4 Hz, 1H, H_{cis}), 5.40 (tp, *J* = 2.7, 1.4 Hz, 1H, H_{trans}), 4.95 (dq, *J* = 2.2, 1.1 Hz, 1H, H_{cis}), 4.86 (dt, *J* = 2.9, 1.5 Hz, 1H, H_{trans}), 4.82 (dt, *J* = 2.3, 0.8 Hz, 1H, H_{trans}), 4.77 (dq, *J* = 1.9, 0.9 Hz, 1H, H_{cis}), 4.08 (ddtd, *J* = 10.8, 5.3, 3.6, 1.6 Hz, 2H, H_{cis}+H_{trans}), 2.13–1.88 (m, 6H, H_{cis}+H_{trans}), 1.82–1.81 (m, 3H, H_{cis}), 1.74–1.67 (m, 12H, H_{cis}+H_{trans}), 1.65–1.54 (m, 2H, H_{trans}), 1.43 (s, OH) ppm.

^{13}C NMR (151 MHz, CD_2Cl_2) δ_C = 147.3, 147.3, 139.6, 136.8, 125.1, 123.2, 112.1, 111.5, 69.1, 64.4, 51.4, 46.6, 31.5, 30.6, 26.8, 23.5, 23.2, 22.7, 21.3, 19.6 ppm.

HRMS (GC-EI) m/z calculated for $C_{10}H_{15}O_1$ $[M–H]^-$: 151.1117, found: 151.1117.

GC (achiral) (HP-5 MS 0.25/0.5df; 220/50, 5/min 150 12/min 320, 5 min iso/350, 0.5 bar H_2): t_{trans} = 17.76 min, t_{cis} = 18.33 min.

GC (chiral) (Hydrodex-beta-TBDAc-CD 0.25/?df; 220/95, 42 min iso 8/min, 3 min iso/350, 0.4 bar H_2): $t_{1S,6R}$ = 30.37 min, $t_{1R,6S}$ = 31.38 min, $t_{1R,6R}$ = 34.17 min, $t_{1S,6S}$ = 37.20 min.

The analytical and spectroscopic data are in accordance with those reported.[16,17]

(6S)-3-methyl-6-(prop-1-en-2-yl)cyclohex-2-en-1-ol ((6*S*)-**2**)

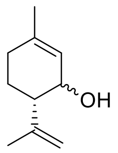

The compounds (6*S*)-**2** was prepared according to modified literature procedures.[17–19] The desired product (6*S*)-**2** was obtained as a colorless liquid (7.2 g, 73% yield over two steps) and as inseparable mixture of diastereoisomers (d.r. (*trans*:*cis*) = 1:4.9; e.r. (*trans*) = >99.9:0.1; e.r. (*cis*) = 99.8:0.2).

The analytical and spectroscopic data are in accordance with those reported.[16,17]

(6R)-3-methyl-6-(prop-1-en-2-yl)cyclohex-2-en-1-ol ((6*R*)-**2**)

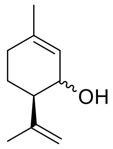

The compounds (6*R*)-**2** was prepared according to modified literature procedures.[17–19] The desired product (6*R*)-**2** was obtained as a colorless liquid (300 mg, 61% yield over two steps) and as inseparable mixture of diastereoisomers (d.r. (*trans*:*cis*) = 1:4.8; e.r. (*trans*) = 97:3; e.r. (*cis*) = 97:3).

The analytical and spectroscopic data are in accordance with those reported.[16,17]

1.3.4. Hydrogenation of Isopiperitenol

*Note: trans-Isopiperitenol (**2**) with shown diastereomeric and enantiomeric ratios was used for following hydrogenation reactions, respectively.*

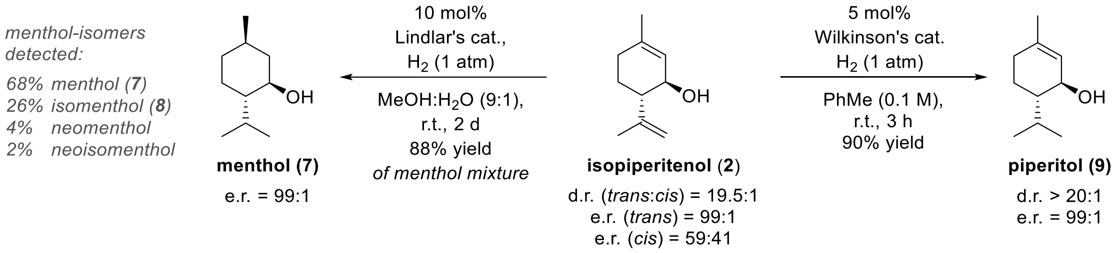

menthol (**7**)

The hydrogenation of isopiperitenol to menthol followed a modified literature procedure.[20] In a flame-dried round-bottom flask under Ar, isopiperitenol (**2**) (150 mg, 0.99 mmol, 1.0 equiv) was dissolved in MeOH:H_2O (9:1, 15 mL) and Lindlar's catalyst (209 mg, 0.09 mmol, 0.1 equiv) was subsequently added. The reaction was purged with hydrogen and was submitted to hydrogenative conditions using a H_2-filled balloon (~1 atm). After two days (full conversion of starting material indicated by TLC / $KMnO_4$-stain), the reaction was filtered over a short pad of Celite and the organic solvent was carefully removed under reduced pressure. The crude was diluted with CH_2Cl_2 and dried over Na_2SO_4. Without further purification, a mixture of four menthol isomers was obtained (135 mg, 88% yield; 95.9% GC purity). The observed ratios of the four isomers are as following: menthol (**7**) (68.12%; e.r. = 99:1), isomenthol (**8**) (26.36%, e.r. = 98.5:1.5), neomenthol (3.80%, e.r. = 86.5:13.5) and neoisomenthol (1.72%, e.r. = 77:23).

GC (achiral) (DB-1 0.25/0.25df; 220/ 50 2/min 110 12/min 320, 5 min iso/350, 0.6 bar H_2): $t_{neomenthol}$ = 17.48 min, $t_{menthol}$ = 17.99 min, $t_{neoisomenthol}$ = 18.51 min $t_{isomenthol}$ = 18.91 min.

GC (chiral) (BGB-176/BGB-15 0.25/0.25df; 220/ 80, iso/350, 0.6 bar H_2): $t_{2S\text{-neomenthol}}$ = 58.30 min, $t_{2R\text{-neomenthol}}$ = 65.32 min, $t_{2S\text{-neoisomenthol}}$ = 68.45 min, $t_{2R\text{-menthol}}$ = 70.39 min, $t_{2S\text{-menthol}}$ = 70.83 min, $t_{2S\text{-isomenthol}}$ = 74.92 min, $t_{2R\text{-isomenthol}}$ = 81.49 min, $t_{2R\text{-neoisomenthol}}$ = 83.47 min.

piperitol (**9**)

In a flame-dried Schlenk flask under Ar, isopiperitenol (**2**) (180 mg, 1.12 mmol, 1.0 equiv) was dissolved in PhMe (15.8 mL) and Wilkinson's catalyst (55 mg, 0.059 mmol, 5 mol%) was subsequently added. The reaction was purged with hydrogen and submitted to hydrogenative conditions using a H_2-filled balloon (~1 atm). After full conversion of the starting material (monitored by ^{1}H NMR spectroscopy, approx. ~3 h), the reaction was purged with Ar and the solvent was evaporated under reduced pressure. Purification by automated FCC (ALOX, pentane:Et_2O 20:1) afforded piperitol (**9**) as a colorless oil and as a mixture of diastereoisomers (165 mg, 90% yield, d.r. (*trans*:*cis*) = 20:1; e.r. (*trans*) = 99:1).

R_f = 0.31 (10:1 hexane:EtOAc, ALOX).
^{1}H NMR (501 MHz, CD_2Cl_2) (*major*) δ_H = 5.38 – 5.33 (m, 1H), 3.97 (s, 1H), 2.04 – 1.86 (m, 3H), 1.70 – 1.63 (m, 1H), 1.67 (s, 3H), 1.38 (br. s., OH), 1.35 – 1.24 (m, 1H), 1.24 – 1.17 (m, 1H), 0.96 (d, *J* = 6.9 Hz, 3H), 0.83 (d, *J* = 6.9Hz, 3H) ppm.
^{13}C NMR (126 MHz, CD_2Cl_2) (*major*) δ_C = 137.7, 126.0, 69.4, 48.4, 30.5, 27.0, 23.2, 21.4, 21.4, 17.5 ppm.
HRMS (GC-EI) m/z calculated for $C_{10}H_{18}O_1$ $[M]^{+\cdot}$: 154.1352, found: 154.1356.
GC (achiral) (DB-1 0.25/0.25df; 220/60, 3/min 120 12/min 320/350, 0.5 bar H_2): t_{cis} = 14.21 min, t_{trans} = 14.79 min.
GC (chiral) (Hydrodex-gamma-TBDAc-CD 0.25/?df; 220/90, 30 min iso 8/min 220/350, 0.5 bar H_2): $t_{1R,6R}$ = 21.05 min, $t_{1S,6S}$ = 24.10 min, e.r. = 99:1.

The analytical and spectroscopic data are in accordance with those reported.[21]

1.3.5. Synthesis of Cannabinoids

*Note: trans-isopiperitenol (**2**) with diastereomeric and enantiomeric ratios (d.r. (trans:cis) = 19.5:1, e.r. (trans) = 99:1, e.r. (cis) = 59:41)) was used for the syntheses of cannabinoids, respectively.*

Cannabidiol (CBD) (**10**)

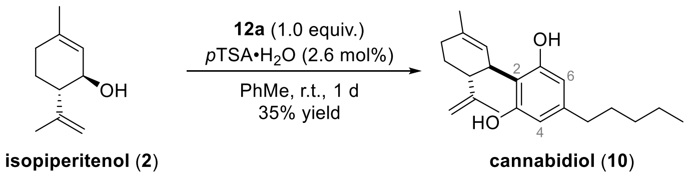

In a flame-dried Schlenk flask under Ar, olivetol (**12a**) (360 mg, 2.0 mmol, 1.0 equiv.), isopiperitenol (480 μL, 3.0 mmol, 1.5 equiv.) and *p*TSA•H_2O (10 mg, 2.6 mol%) were dissolved in PhMe (5 mL) at room temperature. The reaction flask was covered in aluminium-foil paper and stirred for 24 h. After full completion of the reaction (monitored by TLC), the reaction was quenched by five drops of triethylamine and the solvent was removed under reduced pressure. The crude reaction mixture constituted a ~1:1-mixture of the desired product cannabidiol (**10**) (C2-linkage) and its regioisomer, abnormal cannabidiol (C4- or C6-linkage). Purification by FCC (spherical silica gel, 20:1 hexane:EtOAc) afforded cannabidiol (**10**) (221 mg, 35% yield, e.r. = 96:4) as a pale-yellow oil.

$\mathbf{R}_f$ = 0.28 (19:1 hexane:EtOAc).

^{1}H NMR (600 MHz, $CDCl_3$) δ_H = 6.28 (d, *J* = 1.5 Hz, 1H), 6.17 – 6.14 (m, 2H), 5.56 (s, 1H), 5.12 (br. s., OH), 4.65 – 4.59 (m, 1H), 4.52 – 4.44 (m, 1H), 3.87 – 3.81 (m, 1H), 2.43 – 2.39 (m, 2H), 2.36 (td, *J* = 11.0, 3.6 Hz, 1H), 2.27 – 2.14 (m, 1H), 2.12 – 2.04 (m, 1H), 1.82 – 1.72 (m, 5H), 1.66 (s, 3H), 1.55 – 1.47 (m, 2H), 1.32 – 1.18 (m, 4H), 0.84 (t, *J* = 7.1 Hz, 3H) ppm.

^{13}C NMR (151 MHz, $CDCl_3$) δ_C = 155.7, 153.8, 149.5, 143.1, 140.7, 123.7, 113.5, 111.0, 109.5, 107.8, 46.1, 36.7, 35.5, 31.5, 31.0, 30.2, 28.1, 24.1, 22.7, 20.3, 14.3 ppm.

HRMS (GC-EI) m/z calculated for $C_{21}H_{30}O_2$ $[M]^+$: 314.2240, found: 314.2242.

HPLC (IG-3, heptane:*i*PrOH = 99:1, 1.0 mL/min, 298 K, 254 nm): t_{R1} = 15.1 min, t_{R2} = 19.7 min, e.r. = 96:4.

$[\boldsymbol{\alpha}]_{\boldsymbol{D}}^{\mathbf{25}}$ = –66.4 (c = 1.0, $CHCl_3$).

The analytical and spectroscopic data are in accordance with those reported.[22]

methyl (1'R,2'R)-2,6-dihydroxy-5'-methyl-4-pentyl-2'-(prop-1-en-2-yl)-1',2',3',4'-tetrahydro-[1,1'-biphenyl]-3-carboxylate (**11**)

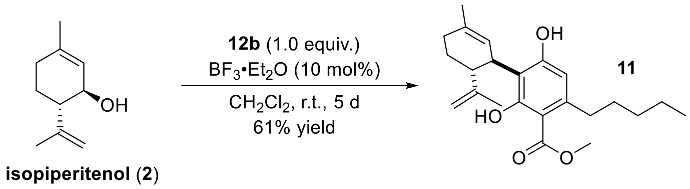

In a flame-dried Schlenk flask under Ar, methyl 2,4-dihydroxy-6-pentylbenzoate (**12b**) (95 mg, 0.4 mmol, 1.0 equiv.), isopiperitenol (72 μL, 0.44 mmol, 1.1 equiv.) and BF_3•Et_2O (5 μL, 10 mol%) were dissolved in CH_2Cl_2 (4 mL) at room temperature. The reaction flask was covered in aluminium-foil paper and was stirred for 5 d. After completion of the reaction (monitored by TLC), the reaction was quenched with five drops of triethylamine and the solvent was removed under reduced pressure. Purification by FCC (spherical silica gel, 20:1 to 10:1 hexane:EtOAc) afforded desired product **11** (92 mg, 61% yield, e.r. = 97:3) as a colorless oil.

$\mathbf{R_f}$ = 0.41 (19:1 hexane:EtOAc).

^{1}H NMR (600 MHz, $CDCl_3$) δ_H = 11.98 (s, 1H), 6.50 (s, 1H), 6.21 (s, 1H), 5.56 (s, 1H), 4.52 (dq, J = 2.3, 1.5 Hz, 1H), 4.38 (s, 1H), 4.10 (d, J = 9.1 Hz, 1H), 3.90 (s, 3H), 2.90 – 2.80 (m, 1H), 2.80 – 2.67 (m, 1H), 2.38 (q, J = 9.1 Hz, 1H), 2.31 – 2.19 (m, 1H), 2.16 –2.04 (m, 1H), 1.86 – 1.75 (m, 5H), 1.71 (s, 3H), 1.59 – 1.44 (m, 2H), 1.44 – 1.24 (m, 4H), 0.94 – 0.83 (m, 3H) ppm.

^{13}C NMR (151 MHz, $CDCl_3$) δ_C = 172.8, 163.3, 160.1, 147.4, 146.1, 140.4, 124.2, 114.5, 111.6, 111.4, 104.0, 51.9, 46.8, 37.0, 35.5, 32.2, 31.5, 30.4, 28.0, 23.9, 22.7, 19.0, 14.2 ppm.

HRMS (ESI) m/z calculated for $C_{23}H_{33}O_4$ $[M+H]^+$: 373.2373, found: 373.2374.

HPLC (AD-3, heptane:*i*PrOH = 99:1, 0.5 mL/min, 298 K, 254 nm): t_{R1} = 7.9 min, t_{R2} = 9.1 min, e.r. = 97:3.

$[\alpha]_D^{25}$ = 9.8 (c = 1.0, $CHCl_3$).

Δ⁹-Tetrahydrocannabinol (**13**)

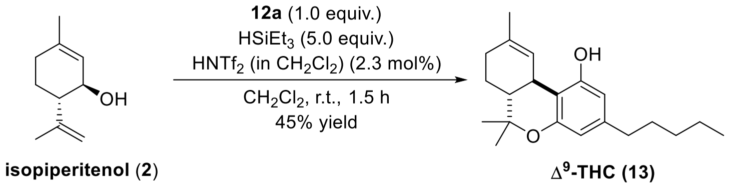

In a flame-dried Schlenk flask under Ar, olivetol (**12a**) (217 mg, 1.2 mmol, 1.0 equiv.) and triethylsilane (0.96 mL, 6.0 mmol, 5.0 equiv.) were dissolved in CH_2Cl_2 (24 mL) at room temperature. A freshly prepared solution of bistriflimide (0.425 M in $CHCl_3$, 64 µL, 0.027 mmol, 2.3 mol%) was added and the reaction stirred for 20 minutes before isopiperitenol (220 mg, 1.44 mmol, 1.2 equiv.) was subsequently added. The reaction flask was covered in aluminium-foil paper and was stirred for 1.5 h (reaction progress was monitored by GC-MS). The reaction was quenched by five drops of triethylamine and the solvent was removed under reduced pressure. Purification by FCC (spherical silica gel, gradient 100:1 to 50:1 pentane:Et_2O) afforded Δ^9-THC (**13**) (168 mg, 45% yield, e.r. = 95.5:4.5) as a colorless oil.

^{1}H NMR (600 MHz, $CDCl_3$) δ_H = 6.30 (hept, *J* = 1.8 Hz, 1H), 6.27 (d, *J* = 1.6 Hz, 1H), 6.14 (d, *J* = 1.6 Hz, 1H), 4.75 (s, 1H), 3.20 (dhept, *J* = 11.1, 2.1 Hz, 1H), 2.48 – 2.39 (m, 2H), 2.22 – 2.12 (m, 2H), 1.95 – 1.88 (m, 1H), 1.70 (td, *J* = 12.0, 11.1, 2.1 Hz, 1H), 1.70 – 1.66 (m, 3H), 1.56 (p, *J* = 7.5 Hz, 2H), 1.45 – 1.37 (m, 1H), 1.41 (s, 3H), 1.35 – 1.25 (m, 4H), 1.09 (s, 3H), 0.88 (t, *J* = 7.1 Hz, 3H) ppm.

^{13}C NMR (151 MHz, $CDCl_3$) δ_C = 154.9, 154.3, 143.0, 134.6, 123.9, 110.2, 109.2, 107.7, 77.4, 45.9, 35.6, 33.7, 31.7, 31.3, 30.8, 27.7, 25.2, 23.5, 22.7, 19.4, 14.2 ppm.

HRMS (GC-EI) m/z calculated for $C_{21}H_{30}O_2$ $[M]^{+\cdot}$: 314.2240, found: 314.2241.

HPLC (OJ–3R, $MeCN/H_2O/$ = 60:40, 1 mL/min, 298 K, 210 nm): t_{R1} = 10.6 min, t_{R2} = 11.9 min, e.r. = 95.5:4.5.

$[\boldsymbol{\alpha}]_{\boldsymbol{D}}^{\boldsymbol{25}}$ = –81.60 (c = 1.0, $CHCl_3$);

The analytical and spectroscopic data are in accordance with those reported.[19,23]

General reaction procedure for the synthesis of Δ⁸-THC and its derivative (GP–3): In a flame-dried Schlenk flask under Ar, resorcinol derivative (1.0 equiv.) was dissolved in CH_2Cl_2 (0.05 M) at room temperature. Subsequently TMSOTf (0.2 equiv.) and isopiperitenol (1.2 equiv.) were added. The reaction flask was covered in aluminium-foil paper and was stirred for 17 h (reaction progress was monitored by GC-MS). The reaction was quenched by five

drops of triethylamine and the solvent was removed under reduced pressure. Purification by FCC (spherical silica gel, pentane:Et_2O) afforded the desired compounds **14**–**15**.

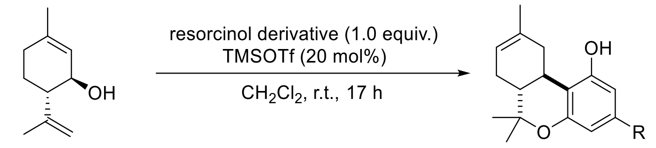

isopiperitenol (**2**)

Δ⁸-Tetrahydrocannbidiol (**14**):

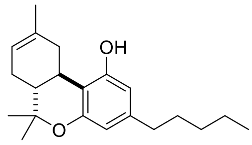

The title compound was prepared according to the general procedure *GP–3* with olivetol (**12a**) (360 mg, 2.0 mmol, 1.0 equiv.), TMSOTf (72 μL, 0.4 mmol, 0.2 equiv.) and isopiperitenol (**2**) (365 mg, 2.4 mmol, 1.2 equiv.). Purification by FCC (spherical silica gel, gradient 100:1 to 20:1 pentane:Et_2O) yielded the desired compound Δ^8-THC (**14**) (406 mg, 65% yield, e.r. = 96:4) as colorless oil.

^{1}H NMR (501 MHz, $CDCl_3$) δ_H = 6.28 (d, *J* = 1.5 Hz, 1H), 6.11 (d, *J* = 1.6 Hz, 1H), 5.43 (d, *J* = 4.3 Hz, 1H), 4.76 (s, 1H), 3.24 – 3.12 (m, 1H), 2.70 (td, *J* = 10.8, 4.7 Hz, 1H), 2.44 (td, *J* = 7.6, 3.5 Hz, 2H), 2.18 – 2.08 (m, 1H), 1.90 – 1.75 (m, 3H), 1.72 – 1.69 (m, 3H), 1.57 (p, *J* = 7.5 Hz, 2H), 1.38 (s, 3H), 1.36 – 1.25 (m, 4H), 1.11 (s, 3H), 0.89 (t, *J* = 6.9 Hz, 3H) ppm.
^{13}C NMR (126 MHz, $CDCl_3$) δ_C = 155.0, 154.9, 142.9, 134.9, 119.5, 110.7, 110.3, 107.8, 76.8, 45.0, 36.2, 35.6, 31.7, 31.7, 30.7, 28.0, 27.7, 23.6, 22.7, 18.6, 14.2 ppm.
HRMS (GC-EI) m/z calculated for $C_{21}H_{30}O_2$ $[M]^{+\cdot}$: 314.2240, found: 314.2243.
HPLC (AD-3, heptane:*i*PrOH = 98:2, 1 mL/min, 298 K, 254 nm): t_{R1} = 12.4 min, t_{R2} = 15.8 min, e.r. = 96:4.
$[\boldsymbol{\alpha}]_{\boldsymbol{D}}^{\mathbf{25}}$ = –216.28 (c = 0.41, $CHCl_3$).
The analytical and spectroscopic data are in accordance with those reported.[19]

(1R,6S)-3-methyl-6-(prop-1-en-2-yl)cyclohex-2-en-1-ol-(6aR,10aR)-6,6,9-trimethyl-3-(2-methyloctan-2-yl)-6a,7,10,10a-tetrahydro-6H-benzo[c]chromen-1-ol (**15**):

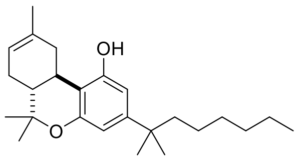

The title compound was prepared according to the general procedure *GP–3* with 5-(1,1-dimethylheptyl)-resorcinol (**12c**) (236 mg, 1.0 mmol, 1.0 equiv.), TMSOTf (36 µL, 0.2 mmol, 0.2 equiv.) and isopiperitenol (**2**) (183 mg, 1.2 mmol, 1.2 equiv.). Purification by FCC (spherical silica gel, gradient 100:1 to 20:1 pentane:Et_2O) yielded the desired compound **15** (330 mg, 89% yield, e.r. = 96:4) as colorless oil.

^{1}H NMR (600 MHz, $CDCl_3$) δ_H = 6.39 (d, *J* = 1.8 Hz, 1H), 6.23 (d, *J* = 1.8 Hz, 1H), 5.52 – 5.41 (m, 1H), 4.68 (s, 1H), 3.19 (dd, *J* = 16.8, 4.8 Hz, 1H), 2.70 (td, *J* = 11.0, 4.8 Hz, 1H), 2.23 –2.06 (m, 1H), 1.98 – 1.78 (m, 3H), 1.71 (s, 3H), 1.56 – 1.44 (m, 2H), 1.39 (s, 3H), 1.30 – 1.15 (m, 12H), 1.11 (s, 3H), 1.09 – 1.02 (m, 2H), 0.85 (t, *J* = 7.1 Hz, 3H) ppm.
^{13}C NMR (151 MHz, $CDCl_3$) δ_C = 154.7, 154.6, 150.2, 134.9, 119.5, 110.3, 108.2, 105.6, 76.8, 45.0, 44.6, 37.5, 36.1, 31.9, 31.7, 30.2, 28.9, 28.8, 28.0, 27.8, 24.8, 23.6, 22.8, 18.7, 14.2 ppm.
HRMS (ESI) m/z calculated for $C_{25}H_{39}O_2$ $[M+H]^+$: 371.2945, found: 371.2947.
HPLC (AD-3, heptane:*i*PrOH = 98:2, 1 mL/min, 298 K, 254 nm): t_{R1} = 6.1 min, t_{R2} = 6.8 min, e.r. = 96:4.
$[\alpha]_D^{25}$ = –204.71 (c = 0.59, $CHCl_3$).

1.3.6. Synthesis of α,β-unsaturated aldehydes

The synthesis of β-substituted α,β-unsaturated aldehydes (**S19–S25**) followed the shown general reaction scheme. The employed (*E*)-tosylate **S26** was prepared according to literature and the subsequent Negishi cross-coupling followed a respective literature procedure.[24]

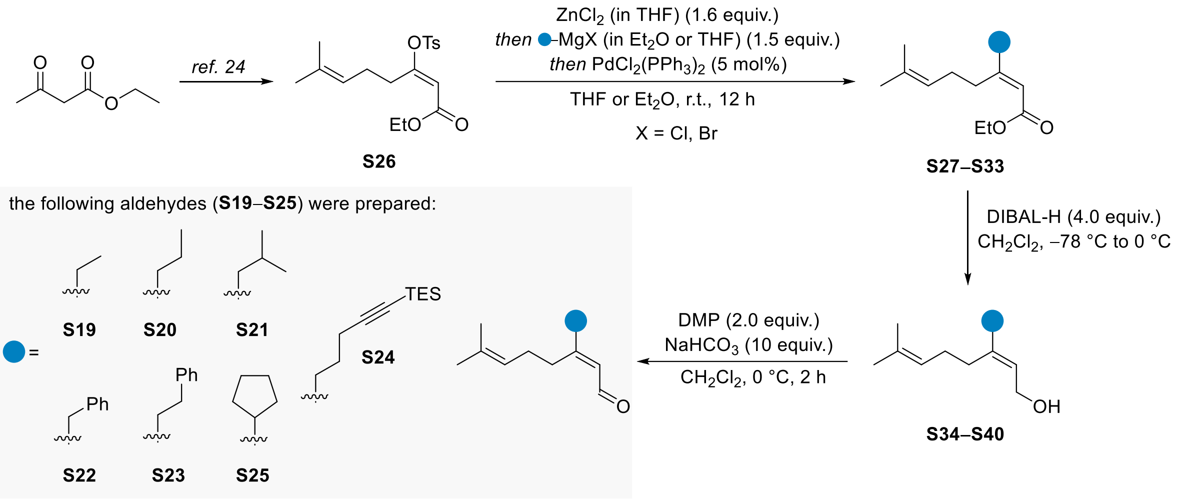

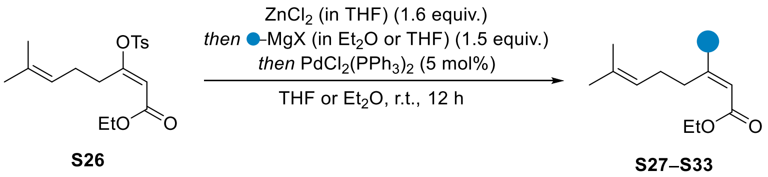

General procedure for the Negishi cross-coupling (GP–4): The synthesis followed a literature procedure.[24] A flame-dried flask under Ar was charged with a solution of $ZnCl_2$ (0.5 M in THF, 1.6 equiv.), to which was carefully added a solution of Grignard reagent (alkylmagnesium bromide or alkylmagenesium chloride in THF or Et_2O) at room temperature. The solution formed a precipitate after full addition of the Grignard reagent and the reaction was stirred for 45 minutes. A separate flame-dried flask under Ar was charged with (*E*)-tosylate **S26**[24], THF (0.9 M) and $PdCl_2(PPh_3)_2$ (5 mol%). The yellow suspension was carefully added to the reaction flask and the reaction was stirred until full consumption of the tosylate (monitored by TLC). The reaction was quenched with brine and diluted with Et_2O. The layers were separated and the aqueous layer was extracted with Et_2O. The combined organic layers were dried over Na_2SO_4 and the solvent was removed under reduced pressure. Purification by automated CC (spherical silica gel, gradient hexane:CH_2Cl_2) afforded the corresponding esters **S27–S33**.

ethyl (Z)-3-ethyl-7-methylocta-2,6-dienoate (**S27**)

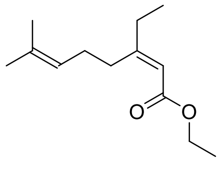

The title compound was prepared according to the general procedure *GP–4* with $ZnCl_2$ (0.5 M in THF, 13.5 mL, 6.75 mmol), EtMgBr (3 M in THF, 1.9 mL, 5.7 mmol), **S26** (1.5 g, 4.26 mmol) and $PdCl_2(PPh_3)_2$ (149 mg, 0.21 mmol). Purification by automated CC (spherical silica gel, gradient 10:1 to 5:1 hexane:CH_2Cl_2) yielded the desired compound **S27** (546 mg, 61% yield) as colorless oil.

R*f* = 0.33 (3:1 hexane:CH_2Cl_2).
^{1}H NMR (501 MHz, $CDCl_3$) δ_H = 5.61 (s, 1H), 5.15 (tp, *J* = 7.2, 1.4 Hz, 1H), 4.13 (q, *J* = 7.1 Hz, 2H), 2.64 – 2.57 (m, 2H), 2.21 – 2.09 (m, 4H), 1.69 – 1.65 (m, 3H), 1.60 (s, 3H), 1.26 (t, *J* = 7.1 Hz, 3H), 1.05 (t, *J* = 7.4 Hz, 3H) ppm.
^{13}C NMR (126 MHz, $CDCl_3$) δ_C = 166.7, 165.3, 132.1, 124.0, 114.5, 59.5, 32.6, 31.5, 27.4, 25.8, 17.7, 14.5, 12.1 ppm.
HRMS (GC-EI) m/z calculated for $C_{13}H_{22}O_2$ $[M]^+$: 210.1614, found: 210.1613.

ethyl (Z)-7-methyl-3-propylocta-2,6-dienoate (**S28**)

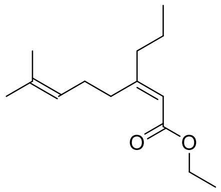

The title compound was prepared according to the general procedure *GP–4* with $ZnCl_2$ (0.5 M in THF, 9.0 mL, 4.5 mmol), *n*PrMgCl (2 M in Et_2O, 1.7 mL, 3.5 mmol), **S26** (1.0 g, 2.8 mmol) and $PdCl_2(PPh_3)_2$ (99.6 mg, 0.14 mmol). Purification by automated CC (spherical silica gel, 10:1 hexane:CH_2Cl_2) yielded the desired compound **S28** (555 mg, 87% yield) as colorless oil.

R*f* = 0.35 (3:1 hexane:CH_2Cl_2).
^{1}H NMR (501 MHz, $CDCl_3$) δ_H = 5.61 (s, 1H), 5.18 – 5.11 (m, 1H), 4.13 (q, *J* = 7.1 Hz, 2H), 2.62 – 2.56 (m, 2H), 2.17 – 2.08 (m, 4H), 1.67 (s, 3H), 1.61 (s, 3H), 1.49 (h, *J* = 7.4 Hz, 2H), 1.26 (t, *J* = 7.2 Hz, 3H), 0.91 (t, *J* = 7.4 Hz, 3H) ppm.
^{13}C NMR (126 MHz, $CDCl_3$) δ_C = 166.7, 163.9, 132.1, 124.0, 115.6, 59.6, 40.7, 32.3, 27.4, 25.8, 20.9, 17.7, 14.5, 13.9 ppm.
HRMS (GC-EI) m/z calculated for $C_{14}H_{24}O_2$ $[M]^+$: 224.1771, found: 224.1770.

ethyl (E)-3-isobutyl-7-methylocta-2,6-dienoate (**S29**)

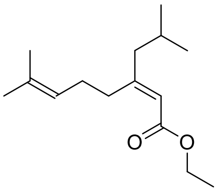

The title compound was prepared according to the general procedure *GP–4* with $ZnCl_2$ (0.5 M in THF, 9.0 mL, 4.5 mmol), *i*BuMgCl (2 M in THF, 1.8 mL, 3.6 mmol), **S26** (1.0 g, 2.8 mmol) and $PdCl_2(PPh_3)_2$ (99.6 mg, 0.14 mmol). Purification by automated CC (spherical silica gel, gradient 20:1 to 10:1 hexane:CH_2Cl_2) yielded the desired compound **S29** (392 mg, 58% yield) as colorless oil.

R*f* = 0.37 (3:1 hexane:CH_2Cl_2).

^{1}H NMR (501 MHz, CD_2Cl_2) δ_H = 5.59 (s, 1H), 5.15 (ddq, *J* = 8.7, 5.8, 1.5 Hz, 1H), 4.11 (q, *J* = 7.1 Hz, 2H), 2.62 – 2.54 (m, 2H), 2.17 – 2.09 (m, 2H), 2.02 (dd, *J* = 7.2, 1.1 Hz, 2H), 1.85 (dp, *J* = 13.7, 6.8 Hz, 1H), 1.68 (d, *J* = 0.8 Hz, 3H), 1.62 (s, 3H), 1.25 (t, *J* = 7.1 Hz, 3H), 0.90 (d, *J* = 6.6 Hz, 6H) ppm.

^{13}C NMR (126 MHz, CD_2Cl_2) δ_C = 166.6, 163.1, 132.4, 124.3, 117.1, 59.8, 48.4, 32.3, 27.6, 26.9, 25.8, 22.7, 17.8, 14.6 ppm.

HRMS (GC-EI) m/z calculated for $C_{15}H_{26}O_2$ $[M]^+$: 238.1927, found: 238.1928.

ethyl (E)-3-benzyl-7-methylocta-2,6-dienoate (**S30**)

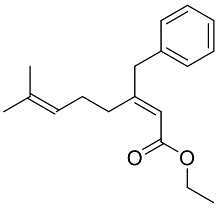

The title compound was prepared according to the general procedure *GP–4* with $ZnCl_2$ (0.5 M in THF, 9.0 mL, 4.5 mmol), BnMgCl (2 M in THF, 1.8 mL, 3.6 mmol), **S26** (1.0 g, 2.8 mmol) and $PdCl_2(PPh_3)_2$ (99.6 mg, 0.14 mmol). Purification by automated CC (spherical silica gel, gradient 10:1 to 4:1 hexane:CH_2Cl_2) yielded the desired compound **S30** (646 mg, 84% yield) as colorless oil.

R*f* = 0.29 (3:1 hexane:CH_2Cl_2).

^{1}H NMR (501 MHz, $CDCl_3$) δ_H = 7.33 – 7.28 (m, 2H), 7.26 – 7.21 (m, 1H), 7.19 – 7.15 (m, 2H), 5.59 (s, 1H), 5.15 (ddp, *J* = 7.2, 5.9, 1.4 Hz, 1H), 4.14 (q, *J* = 7.1 Hz, 2H), 3.47 (s, 2H), 2.66 – 2.55 (m, 2H), 2.16 (q, *J* = 7.7 Hz, 2H), 1.69 (s, 3H), 1.60 (s, 3H), 1.27 (t, *J* = 7.1 Hz, 3H) ppm.

^{13}C NMR (126 MHz, $CDCl_3$) δ_C = 166.5, 162.4, 138.0, 132.3, 129.5, 128.7, 126.8, 123.9, 117.6, 59.7, 45.1, 32.0, 27.4, 25.8, 17.8, 14.4 ppm.

HRMS (GC-EI) m/z calculated for $C_{18}H_{24}O_2$ $[M]^+$: 272.1771, found: 272.1773.

ethyl (E)-7-methyl-3-phenethylocta-2,6-dienoate (**S31**)

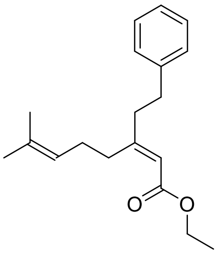

The title compound was prepared according to the general procedure *GP–4* with $ZnCl_2$ (0.5 M in THF, 9.0 mL, 4.5 mmol), 2-phenethylmagnesium chloride (1 M in THF, 3.5 mL, 3.5 mmol), **S26** (1.0 g, 2.8 mmol) and $PdCl_2(PPh_3)_2$ (99.6 mg, 0.14 mmol). Purification by automated CC (spherical silica gel, gradient 10:1 to 5:1 hexane:CH_2Cl_2) yielded the desired compound **S31** (656 mg, 81% yield) as colorless oil.

R*f* = 0.29 (3:1 hexane:CH_2Cl_2).

^{1}H NMR (501 MHz, $CDCl_3$) δ_H = 7.33 – 7.28 (m, 2H), 7.24 – 7.16 (m, 3H), 5.69 (s, 1H), 5.18 (ddq, *J* = 8.6, 5.8, 1.5 Hz, 1H), 4.16 (q, *J* = 7.1 Hz, 2H), 2.82 – 2.74 (m, 2H), 2.71 – 2.65 (m, 2H), 2.50 – 2.43 (m, 2H), 2.19 (q, *J* = 7.6 Hz, 2H), 1.71 – 1.68 (m, 3H), 1.63 (s, 3H), 1.29 (t, *J* = 7.1 Hz, 3H) ppm.

^{13}C NMR (126 MHz, $CDCl_3$) δ_C = 166.5, 162.9, 141.4, 132.3, 128.6, 128.4, 126.2, 123.8, 116.0, 59.7, 40.5, 34.3, 32.5, 27.4, 25.8, 17.8, 14.5 ppm.

HRMS (GC-CI) m/z calculated for $C_{19}H_{27}O_2$ $[M+H]^+$: 287.2006, found: 287.2008.

ethyl (Z)-7-methyl-3-(5-(triethylsilyl)pent-4-yn-1-yl)octa-2,6-dienoate (**S32**)

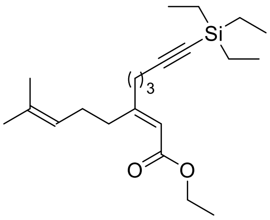

The title compound was prepared according to the general procedure *GP–4* with $ZnCl_2$ (0.5 M in THF, 9.0 mL, 4.5 mmol), (5-(triethylsilyl)pent-4-yn-1-yl)magnesium chloride[3] (~1 M in THF, 3.8 mL, 3.8 mmol), **S26** (1.0 g, 2.8 mmol) and $PdCl_2(PPh_3)_2$ (99.6 mg, 0.14 mmol). Purification by automated CC (spherical silica gel, gradient 20:1 to 10:1 hexane:CH_2Cl_2) yielded the desired compound **S32** (532 mg, 52% yield) as colorless oil.

R*f* = 0.31 (3:1 hexane:CH_2Cl_2).

^{1}H NMR (501 MHz, CD_2Cl_2) δ_H = 5.64 (s, 1H), 5.14 (tdd, *J* = 5.9, 2.7, 1.4 Hz, 1H), 4.11 (q, *J* = 7.1 Hz, 2H), 2.66 – 2.56 (m, 2H), 2.33 – 2.23 (m, 4H), 2.14 (q, *J* = 7.7 Hz, 2H), 1.73 – 1.64 (m, 2H), 1.68 (s, 3H), 1.62 (s, 3H), 1.25 (t, *J* = 7.1 Hz, 3H), 0.98 (t, *J* = 7.9 Hz, 9H), 0.57 (q, *J* = 7.9 Hz, 6H) ppm.

---

[3] The employed Grignard reagent was freshly prepared according to a modified literature procedure for a related compound[51] with (5-chloropent-1-yn-1-yl)triethylsilane (1.0 g, 4.6 mmol, 1.0 equiv.) and magnesium (224 mg, 9.22 mmol, 2.equiv.) and was diluted with THF to a concentration of 1 M.

^{13}C NMR (126 MHz, CD_2Cl_2) δ_C = 166.6, 163.1, 132.5, 124.1, 116.4, 108.1, 82.6, 59.8, 37.6, 32.5, 27.6, 27.2, 25.8, 19.8, 17.8, 14.5, 7.7, 4.9 ppm.

HRMS (GC-CI) m/z calculated for $C_{22}H_{38}O_2Si_1$ $[M]^+$: 362.2636, found: 362.2635.

ethyl (E)-3-cyclopentyl-7-methylocta-2,6-dienoate (**S33**)

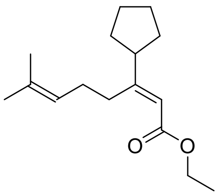

The title compound was prepared according to the general procedure *GP–4* with $ZnCl_2$ (0.5 M in THF, 9.0 mL, 4.5 mmol), cyclopentylmagnesium bromide (1 M in THF, 3.9 mL, 3.9 mmol), **S26** (1.0 g, 2.8 mmol) and $PdCl_2(PPh_3)_2$ (99.6 mg, 0.14 mmol). Purification by automated CC (spherical silica gel, 10:1 hexane:CH_2Cl_2) yielded the desired compound **S33** (393 mg, 55% yield) as colorless oil.

R$_f$ = 0.35 (3:1 hexane:CH_2Cl_2).

^{1}H NMR (501 MHz, $CDCl_3$) δ_H = 5.66 – 5.65 (m, 1H), 5.17 (tp, J = 7.2, 1.5 Hz, 1H), 4.14 (q, J = 7.1 Hz, 2H), 2.62 – 2.57 (m, 2H), 2.57 – 2.49 (m, 1H), 2.14 (q, J = 7.8 Hz, 2H), 1.89 – 1.79 (m, 2H), 1.76 – 1.66 (m, 5H), 1.65 – 1.54 (m, 2H), 1.62 (s, 3H), 1.48 – 1.37 (m, 2H), 1.27 (t, J = 7.1 Hz, 3H) ppm.

^{13}C NMR (126 MHz, $CDCl_3$) δ_C = 167.2, 166.8, 131.9, 124.1, 113.6, 59.4, 48.5, 32.3, 31.7, 27.9, 25.7, 25.0, 17.7, 14.3 ppm.

HRMS (GC-EI) m/z calculated for $C_{16}H_{26}O_2$ $[M]^+$: 250.1927, found: 250.1925.

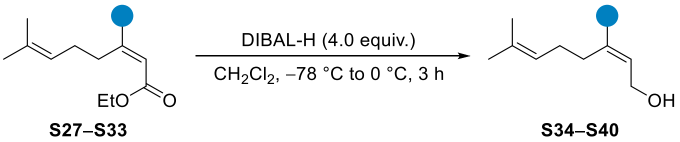

General procedure for the DIBAL–H reduction (GP–5): In a flame-dried flask under Ar, ester **S27**–**S33** (1.0 equiv.) was dissolved in CH_2Cl_2 (0.25 M) and was cooled to –78 °C. A solution of DIBAL–H (1 M in CH_2Cl_2, 4.0 equiv.) was added dropwise and the reaction stirred for 15 minutes at –78 °C. The reaction was warmed up to 0 °C and was stirred until full conversion of the ester (monitored by TLC). The reaction was quenched with a 1:1 mixture H_2O/MeOH, was warmed up to room temperature and was stirred for 2 h at room temperature. The resulting reaction mixture was filtered over a pad of Na_2SO_4/Celite, which was extensively washed with CH_2Cl_2. The organic layer was washed with brine, dried over Na_2SO_4 and the solvent was

removed under reduced pressure. Purification by FCC (spherical silica gel, hexane:EtOAc) afforded the corresponding alcohols **S34**–**S40**.

(Z)-3-ethyl-7-methylocta-2,6-dien-1-ol (**S34**)

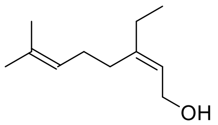

The title compound was prepared according to the general procedure *GP–5* with **S27** (526 mg, 2.50 mmol), DIBAL-H (1 M in CH_2Cl_2, 10 mL, 10 mmol). Purification by FCC (spherical silica gel, 10:1 hexane:EtOAc) yielded the desired compound **S34** (318 mg, 76% yield) as colorless oil.

R*f* = 0.30 (7:1 hexane:EtOAc).

^{1}H NMR (501 MHz, CD_2Cl_2) δ_H = 5.41 – 5.35 (m, 1H), 5.12 (tp, *J* = 7.1, 1.5 Hz, 1H), 4.13 – 4.06 (m, 2H), 2.13 – 2.01 (m, 6H), 1.70 – 1.66 (m, 3H), 1.60 (s, 3H), 1.36 (s, OH), 1.02 (t, *J* = 7.4 Hz, 3H) ppm.

^{13}C NMR (126 MHz, CD_2Cl_2) δ_C = 145.2, 132.5, 124.4, 123.3, 59.3, 30.9, 29.8, 27.6, 25.8, 17.7, 12.8 ppm.

HRMS (GC-EI) m/z calculated for $C_{11}H_{20}O_1$ $[M]^+$: 168.1509, found: 168.1508.

(Z)-7-methyl-3-propylocta-2,6-dien-1-ol (**S35**)

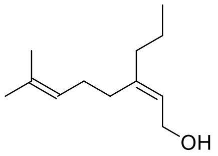

The title compound was prepared according to the general procedure *GP–5* with **S28** (260 mg, 1.16 mmol), DIBAL-H (1 M in CH_2Cl_2, 4.6 mL, 4.6 mmol). Purification by FCC (spherical silica gel, 10:1 hexane:EtOAc) yielded the desired compound **S35** (177 mg, 84% yield) as colourless oil.

R*f* = 0.31 (7:1 hexane:EtOAc).

^{1}H NMR (501 MHz, $CDCl_3$) δ_H = 5.41 (t, *J* = 6.9 Hz, 1H), 5.13 – 5.07 (m, 1H), 4.11 (d, *J* = 7.0 Hz, 2H), 2.11 – 1.97 (m, 6H), 1.68 (s, 3H), 1.59 (s, 3H), 1.51 – 1.39 (m, 2H+OH), 0.89 (t, *J* = 7.3 Hz, 3H) ppm.

^{13}C NMR (126 MHz, $CDCl_3$) δ_C = 143.7, 132.4, 124.1, 124.0, 59.2, 39.0, 30.5, 27.2, 25.8, 21.2, 17.8, 14.0 ppm.

HRMS (GC-EI) m/z calculated for $C_{12}H_{22}O_1$ $[M]^+$: 182.1665, found: 182.1666.

(E)-3-isobutyl-7-methylocta-2,6-dien-1-ol (**S36**)

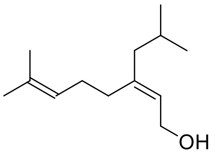

The title compound was prepared according to the general procedure *GP–5* with **S29** (392 mg, 1.75 mmol), DIBAL-H (1 M in CH_2Cl_2, 7 mL, 7 mmol). Purification by FCC (spherical silica gel, 10:1 hexane:EtOAc) yielded the desired compound **S36** (274 mg, 80% yield) as colorless oil.

$\mathbf{R}_f$ = 0.33 (7:1 hexane:EtOAc).

^{1}H NMR (501 MHz, $CDCl_3$) δ_H = 5.40 (t, *J* = 7.2 Hz, 1H), 5.13 – 5.07 (m, 1H), 4.13 (d, *J* = 7.1 Hz, 2H), 2.09 – 2.01 (m, 4H), 1.90 (d, *J* = 7.4 Hz, 2H), 1.82 – 1.69 (m, 1H), 1.69 (s, 3H), 1.60 (s, 3H), 1.32 (s, OH), 0.87 (d, *J* = 6.6 Hz, 6H) ppm.

^{13}C NMR (126 MHz, $CDCl_3$) δ_C = 142.8, 132.5, 125.4, 124.1, 59.2, 46.5, 30.3, 27.1, 26.3, 25.8, 22.7, 17.8 ppm.

HRMS (GC-CI) m/z calculated for $C_{13}H_{25}O_1$ $[M+H]^+$: 197.1900, found: 197.1900.

(E)-3-benzyl-7-methylocta-2,6-dien-1-ol (**S37**)

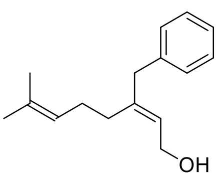

The title compound was prepared according to the general procedure *GP–5* with **S30** (624 mg, 2.29 mmol), DIBAL-H (1 M in CH_2Cl_2, 9.2 mL, 9.2 mmol). Purification by FCC (spherical silica gel, 10:1 hexane:EtOAc) yielded the desired compound **S37** (473 mg, 90% yield) as colorless oil.

$\mathbf{R}_f$ = 0.20 (7:1 hexane:EtOAc).

^{1}H NMR (501 MHz, $CDCl_3$) δ_H = 7.32 – 7.27 (m, 2H), 7.23 – 7.16 (m, 3H), 5.46 (t, *J* = 7.0 Hz, 1H), 5.13 – 5.06 (m, 1H), 4.15 (d, *J* = 7.0 Hz, 2H), 3.37 (s, 2H), 2.09 – 2.03 (m, 4H), 1.70 (s, 3H), 1.59 (s, 3H), 1.47 (s, OH) ppm.

^{13}C NMR (126 MHz, $CDCl_3$) δ_C = 142.8, 139.6, 132.6, 129.2, 128.4, 126.3, 126.3, 123.9, 59.2, 43.6, 30.2, 27.0, 25.8, 17.8 ppm.

HRMS (GC-EI) m/z calculated for $C_{16}H_{22}O_1$ $[M]^{+}$: 230.1665, found: 230.1665.

(E)-7-methyl-3-phenethylocta-2,6-dien-1-ol (**S38**)

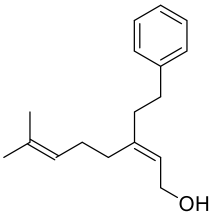

The title compound was prepared according to the general procedure *GP–5* with **S31** (650 mg, 2.27 mmol), DIBAL-H (1 M in CH_2Cl_2, 9.1 mL, 9.1 mmol). Purification by FCC (spherical silica gel, 10:1 hexane:EtOAc) yielded the desired compound **S38** (487 mg, 88% yield) as colourless oil.

R*f* = 0.20 (7:1 hexane:EtOAc).

^{1}H NMR (501 MHz, $CDCl_3$) δ_H = 7.31 – 7.27 (m, 2H), 7.22 – 7.17 (m, 3H), 5.46 (t, *J* = 7.1 Hz, 1H), 5.12 (ddt, *J* = 8.4, 5.4, 1.5 Hz, 1H), 4.13 (d, *J* = 7.0 Hz, 2H), 2.78 – 2.72 (m, 2H), 2.38 – 2.32 (m, 2H), 2.17 – 2.13 (m, 2H), 2.13 – 2.06 (m, 2H), 1.70 (s, 3H), 1.61 (s, 3H), 1.30 (s, OH) ppm.

^{13}C NMR (126 MHz, $CDCl_3$) δ_C = 143.1, 142.2, 132.6, 128.5, 128.5, 126.0, 124.5, 123.9, 59.2, 38.7, 34.7, 30.7, 27.2, 25.8, 17.8 ppm.

HRMS (GC-CI) m/z calculated for $C_{17}H_{25}O_1$ $[M+H]^+$: 245.1900, found: 245.1901.

(Z)-7-methyl-3-(5-(triethylsilyl)pent-4-yn-1-yl)octa-2,6-dien-1-ol (**S39**)

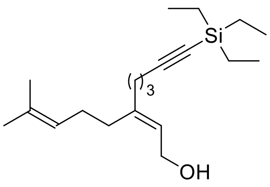

The title compound was prepared according to the general procedure *GP–5* with **S32** (530 mg, 1.46 mmol), DIBAL-H (1 M in CH_2Cl_2, 5.8 mL, 5.8 mmol). Purification by FCC (spherical silica gel, 10:1 hexane:EtOAc) yielded the desired compound **S39** (359 mg, 77% yield) as colorless oil.

R*f* = 0.27 (7:1 hexane:EtOAc).

^{1}H NMR (501 MHz, CD_2Cl_2) δ_H = 5.42 (t, *J* = 7.0 Hz, 1H), 5.14 – 5.08 (m, 1H), 4.09 (t, *J* = 6.1 Hz, 2H), 2.24 (t, *J* = 6.9 Hz, 2H), 2.20 – 2.12 (m, 2H), 2.11 – 2.03 (m, 4H), 1.68 (s, 3H), 1.67 – 1.62 (m, 2H), 1.60 (s, 3H), 1.24 (t, *J* = 5.5 Hz, OH), 0.98 (t, *J* = 7.9 Hz, 9H), 0.57 (q, *J* = 7.9 Hz, 6H) ppm.

^{13}C NMR (126 MHz, CD_2Cl_2) δ_C = 142.8, 132.6, 125.2, 124.3, 108.8, 82.1, 59.3, 35.9, 30.7, 27.7, 27.5, 25.8, 19.8, 17.8, 7.7, 4.9 ppm.

HRMS (GC-EI) m/z calculated for $C_{20}H_{36}O_1Si_1$ $[M]^+$: 320.2530, found: 320.2530.

(E)-3-cyclopentyl-7-methylocta-2,6-dien-1-ol (**S40**)

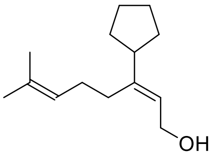

The title compound was prepared according to the general procedure *GP–5* with **S33** (390 mg, 1.56 mmol), DIBAL-H (1 M in CH_2Cl_2, 6.2 mL, 6.2 mmol). Purification by FCC (spherical silica gel, 10:1 hexane:EtOAc) yielded the desired compound **S40** (263 mg, 81% yield) as colourless oil.

R*f* = 0.29 (7:1 hexane:EtOAc).

^{1}H NMR (501 MHz, CD_2Cl_2) δ_H = 5.42 (td, *J* = 6.9, 1.3 Hz, 1H), 5.13 (ddq, *J* = 8.5, 5.7, 1.5 Hz, 1H), 4.11 (d, *J* = 6.9 Hz, 2H), 2.42 (dtt, *J* = 14.5, 7.6, 3.9 Hz, 1H), 2.14 – 1.99 (m, 4H), 1.84 – 1.75 (m, 2H), 1.73 – 1.63 (m, 2H), 1.68 (s, 3H), 1.63 – 1.54 (m, 2H), 1.61 (s, 3H), 1.42 – 1.32 (m, 2H), 1.29 (s, OH) ppm.

^{13}C NMR (126 MHz, CD_2Cl_2) δ_C = 146.7, 132.4, 124.5, 122.6, 59.6, 47.1, 32.1, 30.9, 28.5, 25.8, 25.3, 17.8 ppm.

HRMS (GC-EI) m/z calculated for $C_{14}H_{24}O_1$ $[M]^{+}$: 208.1822, found: 208.1821.

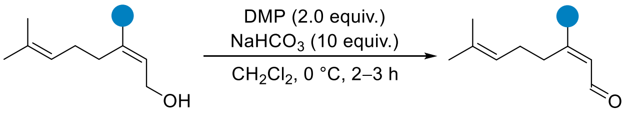

General procedure for the DMP–oxidation (GP–6): In a flame-dried flask under Ar, alcohol **S34–S40** (1.0 equiv.) was dissolved in CH_2Cl_2 (0.1 M). Solid $NaHCO_3$ (10 equiv.) was added and the reaction was cooled to 0 °C. DMP (2.0 equiv.) was added at 0 °C and the reaction was stirred until full conversion of the alcohol (monitored by TLC). The reaction was quenched with sat. aq. $Na_2S_2O_3$ and the reaction was diluted with CH_2Cl_2. The aqueous layer was extracted with CH_2Cl_2 and the combined organic layers were dried over Na_2SO_4. The solvent was removed under reduced pressure. Purification by FCC (spherical silica gel, gradient pentane:Et_2O) afforded the corresponding aldehydes **S19**–**S25**.

(Z)-3-ethyl-7-methylocta-2,6-dienal (**S19**)

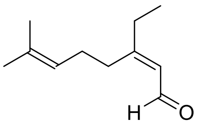

The title compound was prepared according to the general procedure *GP–6* with **S34** (286 mg, 1.70 mmol), $NaHCO_3$ (1.43 g, 17.0 mmol) and DMP (1.44 g, 3.40 mmol). Purification by FCC (spherical silica gel, 10:1 pentane:Et_2O) yielded the desired compound **S19** (212 mg, 75% yield) as colorless oil.

R$_f$ = 0.19 (20:1 hexane:Et_2O).

^{1}H NMR (501 MHz, CD_2Cl_2) δ_H = 9.92 (d, *J* = 8.1 Hz, 1H), 5.81 (d, *J* = 8.2 Hz, 1H), 5.13 (tp, *J* = 7.4, 1.4 Hz, 1H), 2.58 (t, *J* = 7.6 Hz, 2H), 2.27 (qd, *J* = 7.5, 1.4 Hz, 2H), 2.22 (q, *J* = 7.6 Hz, 2H), 1.67 (s, 3H), 1.59 (s, 3H), 1.09 (t, *J* = 7.4 Hz, 3H) ppm.

^{13}C NMR (126 MHz, CD_2Cl_2) δ_C = 191.3, 169.2, 133.8, 126.7, 123.0, 31.8, 31.2, 28.2, 25.7, 17.8, 12.0 ppm.

HRMS (GC-EI) m/z calculated for $C_{11}H_{18}O_1$ $[M]^+$: 166.1352, found: 166.1353.

(Z)-7-methyl-3-propylocta-2,6-dienal (**S20**)

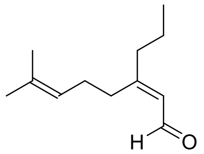

The title compound was prepared according to the general procedure *GP–6* with **S35** (248 mg, 1.36 mmol), $NaHCO_3$ (1.14 g, 13.6 mmol) and DMP (1.15 mg, 2.72 mmol). Purification by FCC (spherical silica gel, 10:1 pentane:Et_2O) yielded the desired compound **S20** (157 mg, 64% yield) as colorless oil.

R$_f$ = 0.19 (20:1 hexane:Et_2O).

^{1}H NMR (501 MHz, CD_2Cl_2) δ_H = 9.91 (d, *J* = 8.1 Hz, 1H), 5.81 (d, *J* = 8.3 Hz, 1H), 5.13 (tdt, *J* = 7.3, 2.9, 1.5 Hz, 1H), 2.57 (t, *J* = 7.6 Hz, 2H), 2.28 – 2.17 (m, 4H), 1.70 – 1.66 (m, 3H), 1.59 (s, 3H), 1.59 – 1.48 (m, 2H), 0.94 (t, *J* = 7.3 Hz, 3H) ppm.

^{13}C NMR (126 MHz, CD_2Cl_2) δ_C = 190.7, 167.3, 133.4, 127.4, 122.5, 40.0, 31.2, 27.7, 25.3, 20.7, 17.4, 13.5 ppm.

HRMS (GC-EI) m/z calculated for $C_{12}H_{20}O_1$ $[M]^+$: 180.1509, found: 180.1509.

(E)-3-isobutyl-7-methylocta-2,6-dienal (**S21**)

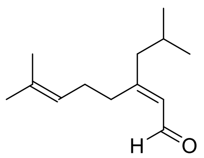

The title compound was prepared according to the general procedure *GP–6* with **S36** (259 mg, 1.32 mmol), $NaHCO_3$ (1.11 g, 13.2 mmol) and DMP (1.12 g, 2.64 mmol). Purification by FCC (spherical silica gel, 10:1 pentane:Et_2O) yielded the desired compound **S21** (179 mg, 70% yield) as colorless oil.

R$_f$ = 0.24 (20:1 hexane:Et_2O).

^{1}H NMR (501 MHz, CD_2Cl_2) δ_H = 9.92 (d, *J* = 8.2 Hz, 1H), 5.79 (d, *J* = 8.2 Hz, 1H), 5.13 (tp, *J* = 7.4, 1.5 Hz, 1H), 2.56 (t, *J* = 7.6 Hz, 2H), 2.26 – 2.17 (m, 2H), 2.10 (dd, *J* = 7.2, 1.1 Hz, 2H), 1.88 (dp, *J* = 13.6, 6.8 Hz, 1H), 1.68 (d, *J* = 0.8 Hz, 3H), 1.59 (s, 3H), 0.92 (d, *J* = 6.6 Hz, 6H) ppm.

^{13}C NMR (126 MHz, CD_2Cl_2) δ_C = 191.1, 166.8, 133.8, 129.1, 123.0, 47.8, 31.5, 28.1, 27.1, 25.8, 22.7, 17.9 ppm.

HRMS (GC-EI) m/z calculated for $C_{13}H_{22}O_1$ $[M]^+$: 194.1665, found: 194.1666.

(E)-3-benzyl-7-methylocta-2,6-dienal (**S22**)

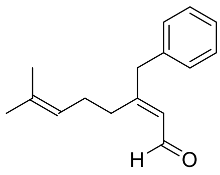

The title compound was prepared according to the general procedure *GP–6* with **S37** (408 mg, 1.77 mmol), $NaHCO_3$ (1.49 g, 17.7 mmol) and DMP (1.50 g, 3.54 mmol). Purification by FCC (spherical silica gel, 10:1 pentane:Et_2O) yielded the desired compound **S22** (346 mg, 86% yield) as colorless oil.

R$_f$ = 0.16 (20:1 hexane:Et_2O).

^{1}H NMR (501 MHz, CD_2Cl_2) δ_H = 9.92 (d, J = 8.1 Hz, 1H), 7.36 – 7.30 (m, 2H), 7.28 – 7.23 (m, 1H), 7.20 – 7.16 (m, 2H), 5.74 (d, J = 8.2 Hz, 1H), 5.13 (tp, J = 7.4, 1.5 Hz, 1H), 3.55 (s, 2H), 2.55 (t, J = 7.5 Hz, 2H), 2.24 (q, J = 7.5 Hz, 2H), 1.71 – 1.67 (m, 3H), 1.59 (s, 3H) ppm;

^{13}C NMR (126 MHz, CD_2Cl_2) δ_C = 191.1, 166.0, 137.9, 134.0, 129.7, 129.2, 129.0, 127.2, 122.8, 44.8, 31.2, 28.1, 25.8, 17.9 ppm.

HRMS (GC-EI) m/z calculated for $C_{16}H_{20}O_1$ $[M]^+$: 228.1509, found: 228.1509.

(E)-7-methyl-3-phenethylocta-2,6-dienal (**S23**)

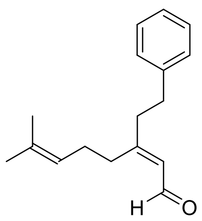

The title compound was prepared according to the general procedure *GP–6* with **S38** (463 mg, 1.89 mmol), $NaHCO_3$ (1.59 g, 18.9 mmol) and DMP (1.61 g, 3.79 mmol). Purification by FCC (spherical silica gel, 10:1 pentane:Et_2O) yielded the desired compound **S23** (340 mg, 74% yield) as colorless oil.

R$_f$ = 0.11 (20:1 hexane:Et_2O).

^{1}H NMR (501 MHz, CD_2Cl_2) δ_H = 9.93 (d, J = 8.1 Hz, 1H), 7.32 – 7.28 (m, 2H), 7.24 – 7.18 (m, 3H), 5.85 (d, J = 8.1 Hz, 1H), 5.14 (tp, J = 7.4, 1.5 Hz, 1H), 2.87 – 2.79 (m, 2H), 2.61 (t, J = 7.6 Hz, 2H), 2.58 – 2.52 (m, 2H), 2.25 (q, J = 7.5 Hz, 2H), 1.70 – 1.69 (m, 3H), 1.60 (s, 3H) ppm.

^{13}C NMR (126 MHz, CD_2Cl_2) δ_C = 191.1, 166.6, 141.5, 134.0, 128.9, 128.7, 128.1, 126.5,

122.8, 40.1, 34.2, 31.8, 28.1, 25.8, 17.9 ppm.

HRMS (GC-EI) m/z calculated for $C_{17}H_{22}O_1$ $[M]^+$: 242.1665, found: 242.1665.

(Z)-7-methyl-3-(5-(triethylsilyl)pent-4-yn-1-yl)octa-2,6-dienal (**S24**)

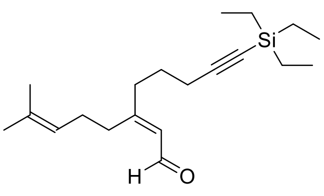

The title compound was prepared according to the general procedure *GP–6* with **S39** (335 mg, 1.05 mmol), $NaHCO_3$ (877 mg, 10.4 mmol) and DMP (886 g, 2.09 mmol). Purification by FCC (spherical silica gel, 10:1 pentane:Et_2O) yielded the desired compound **S25** (260 mg, 78% yield) as colorless oil.

R*f* = 0.15 (20:1 hexane:Et_2O).

^{1}H NMR (501 MHz, CD_2Cl_2) δ_H = 9.91 (d, *J* = 8.1 Hz, 1H), 5.83 (d, *J* = 8.1 Hz, 1H), 5.12 (ddq, *J* = 8.9, 5.9, 1.5 Hz, 1H), 2.58 (t, *J* = 7.6 Hz, 2H), 2.41 – 2.34 (m, 2H), 2.29 (t, *J* = 6.8 Hz, 2H), 2.23 (q, *J* = 7.6 Hz, 2H), 1.75 – 1.68 (m, 2H), 1.68 (s, 3H), 1.59 (s, 3H), 0.98 (t, *J* = 7.9 Hz, 9H), 0.57 (q, *J* = 7.9 Hz, 6H) ppm.

^{13}C NMR (126 MHz, CD_2Cl_2) δ_C = 191.0, 166.8, 133.9, 128.1, 122.8, 107.8, 82.8, 37.1, 31.7, 28.0, 27.1, 25.8, 19.8, 17.9, 7.7, 4.9 ppm.

HRMS (ESI) m/z calculated for $C_{20}H_{34}O_1Si_1Na_1$ $[M+Na]^+$: 341.2271, found: 341.2269.

(E)-3-cyclopentyl-7-methylocta-2,6-dienal (**S25**)

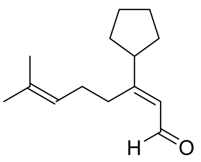

The title compound was prepared according to the general procedure *GP–6* with **S40** (238 mg, 1.14 mmol), $NaHCO_3$ (960 mg, 11.4 mmol) and DMP (969 g, 2.28 mmol). Purification by FCC (spherical silica gel, 10:1 pentane:Et_2O) yielded the desired compound **S25** (167 mg, 71% yield) as colorless oil.

R*f* = 0.20 (20:1 hexane:Et_2O).

^{1}H NMR (501 MHz, CD_2Cl_2) δ_H = 9.93 (d, *J* = 8.1 Hz, 1H), 5.85 (d, *J* = 8.1 Hz, 1H), 5.14 (tdt, *J* = 7.4, 2.9, 1.5 Hz, 1H), 2.65 – 2.54 (m, 3H), 2.23 (q, *J* = 7.7 Hz, 2H), 1.94 – 1.85 (m, 2H), 1.78 – 1.70 (m, 2H), 1.68 (s, 3H), 1.66 – 1.59 (m, 2H), 1.59 (s, 3H), 1.52 – 1.40 (m, 2H) ppm;

^{13}C NMR (126 MHz, CD_2Cl_2) δ_C = 191.5, 171.4, 133.7, 125.8, 123.1, 48.3, 32.3, 31.7, 29.1, 25.8, 25.5, 17.9 ppm.

HRMS (GC-EI) m/z calculated for $C_{14}H_{22}O_1$ $[M]^+$: 206.1665, found: 206.1666.

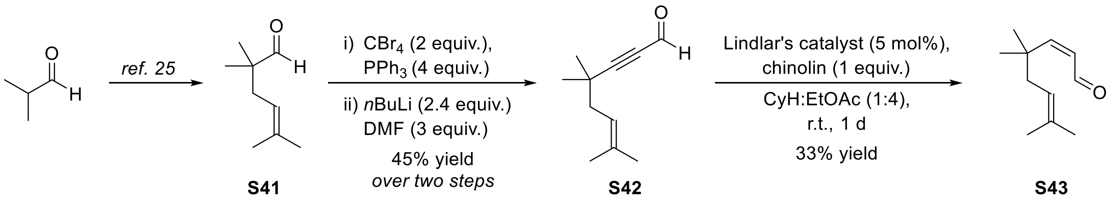

4,4,7-trimethyloct-6-en-2-ynal (**S42**)

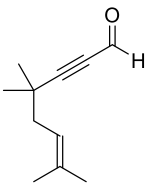

In a flame-dried round-bottom flask under Ar, CBr_4 (4.73 g, 14.3 mmol, 2.0 equiv.) was dissolved in CH_2Cl_2 (37.5 mL) and the reaction was cooled to 0 °C. Triphenylphosphine (7.48 g, 28.5 mmol, 4.0 equiv.) was added and the reaction was stirred for 15 minutes. A solution of aldehyde **S41**[25] (1.0 g, 7.1 mmol, 1.0 equiv) in CH_2Cl_2 (4 mL) was added and the reaction was stirred for 30 minutes at room temperature. The reaction was quenched with H_2O and the aqueous layer was extracted with CH_2Cl_2. The combined organic phases were washed with water, brine and dried over Na_2SO_4 and the solvent was removed under reduced pressure. The crude product was filtrated over a short pad of silica (pentane) and was subsequently used for the next step.

The crude product was dissolved in THF (37 mL) and *n*BuLi (2.5 M in hexane, 6.3 mL, 15.7 mmol, 2.2 equiv.) was added dropwise to the solution at –78 °C. After stirring for 1 hour, DMF (1.7 mL, 21.4 mmol, 3.0 equiv.) was carefully added and the reaction was stirred for 1.5 hours at 0 °C. After full conversion (monitored by TLC), the reaction was quenched with sat. aq. NH_4Cl solution and the aqueous layer was extracted with Et_2O. The combined organic layers were washed with brine, dried over Na_2SO_4 and the solvent was removed under reduced pressure. Purification by FCC (spherical silica gel, gradient pentane to 10:1 pentane:CH_2Cl_2) afforded compound **S42** (532 mg, 45% yield) as a colorless liquid. Due to the high volatility of the product, some solvent residue (CH_2Cl_2) is still present.

R_f = 0.29 (3:1 hexane:CH_2Cl_2).
^{1}H NMR (501 MHz, $CDCl_3$) δ_H = 9.19 (s, 1H), 5.23 (tdq, *J* = 7.6, 2.9, 1.4 Hz, 1H), 2.22 – 2.16 (m, 2H), 1.74 (d, *J* = 0.8 Hz, 3H), 1.62 (s, 3H), 1.25 (s, 6H) ppm;
^{13}C NMR (126 MHz, $CDCl_3$) δ_C = 177.5, 135.2, 119.6, 106.0, 81.5, 40.7, 32.5, 27.9, 26.1, 18.2 ppm.
HRMS (GC-EI) m/z calculated for $C_{11}H_{16}O_1$ $[M]^+$: 164.1196, found: 164.1196.

(Z)-4,4,7-trimethylocta-2,6-dienal (**S43**)

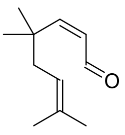

In a flame-dried round-bottom flask, aldehyde **S42** (180 mg, 1.10 mmol, 1.0 equiv.) was dissolved in CyH:EtOAc (1:4, 2 mL). Quinoline (0.13 ml, 1.1 mmol, 1.0 equiv.) and Lindlar's catalyst (116 mg, 0.055 mmol, 5 mol%) was added to the reaction. The reaction was purged with hydrogen and was submitted to hydrogenative conditions using a H_2-filled balloon (~1 atm). After full conversion (indicated by TLC), the reaction was filtered over a short pad of Celite, the pad was further washed with CH_2Cl_2. The organic solvent was carefully removed under reduced pressure. Purification by automated CC (spherical silica gel, gradient pentane to 10:1 pentane:CH_2Cl_2) afforded compound **S43** (60 mg, 33% yield) as a colorless liquid and as a mixture of *Z*:*E*–isomers (*Z*:*E* ~93:7).

$\mathbf{R}_f$ = 0.23 (5:1 hexane:CH_2Cl_2).

^{1}H NMR (501 MHz, $CDCl_3$, *major*) δ_H = 10.21 (d, J = 8.4 Hz, 1H), 6.43 (d, J = 12.6 Hz, 1H), 5.82 (dd, J = 12.5, 8.5 Hz, 1H), 5.13 (tp, J = 7.5, 1.5 Hz, 1H), 2.17 (d, J = 7.6 Hz, 2H), 1.71 (s, 3H), 1.59 (s, 3H), 1.28 (s, 6H) ppm.

^{13}C NMR (126 MHz, $CDCl_3$, *major*) δ_C = 192.3, 160.0, 135.0, 129.6, 119.9, 42.4, 40.2, 29.8, 26.1, 26.1, 18.1 ppm.

HRMS (GC-EI) m/z calculated for $C_{11}H_{18}O_1$ $[M]^+$: 166.1352, found: 166.1354.

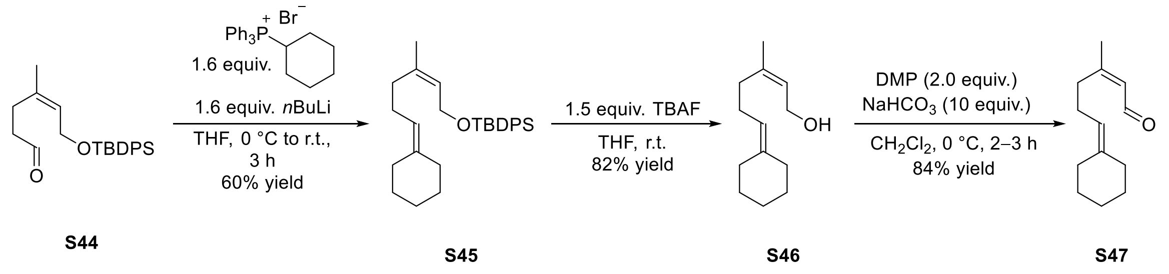

(Z)-tert-butyl((6-cyclohexylidene-3-methylhex-2-en-1-yl)oxy)diphenylsilane (**S45**)

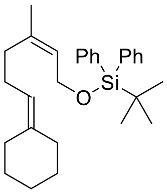

In a flame-dried round-bottom flask under Ar, cyclohexyltriphenylphosphonium bromide (1.86 g, 4.36 mmol, 1.6 equiv.) was dissolved in THF (4.2 mL) and the reaction was cooled to 0 °C. *n*BuLi (2.5 M in hexane, 1.75 mL, 4.36 mmol, 1.6 equiv.) was added dropwise to the reaction. The reaction was warmed up to room temperature and stirred for 30 minutes. A solution of aldehyde **S44**[26] (1.0 g, 2.7 mmol, 1.0 equiv) in THF (1.7 mL) was added and the reaction was stirred for 2 h at room temperature. After full conversion, the reaction was quenched with 10% aq. NH_4Cl. The layers were separated and the aqueous layer was extracted with Et_2O. The combined organic layers were

washed with water, brine and dried over Na_2SO_4 and the solvent was removed under reduced pressure. Purification by automated CC (spherical silica gel, 20:1 hexane:CH_2Cl_2) afforded compound **S45** (708 mg, 60% yield) as a colorless liquid.

R*f* = 0.19 (hexane).
^{1}H NMR (501 MHz, $CDCl_3$) δ_H = 7.72 – 7.66 (m, 4H), 7.45 – 7.35 (m, 6H), 5.40 (td, *J* = 6.4, 1.5 Hz, 1H), 4.96 – 4.91 (m, 1H), 4.19 (dd, *J* = 6.4, 1.3 Hz, 2H), 2.04 – 1.93 (m, 6H), 1.91 – 1.85 (m, 2H), 1.73 – 1.69 (m, 3H), 1.53 – 1.37 (m, 6H), 1.05 (s, 9H) ppm.
^{13}C NMR (126 MHz, $CDCl_3$) δ_C = 140.2, 137.5, 135.8, 135.0, 134.2, 129.6, 127.9, 127.7, 125.1, 120.6, 61.0, 37.2, 32.8, 28.7, 28.7, 28.0, 27.1, 27.0, 26.7, 25.8, 23.6, 19.3 ppm.
HRMS (ESI) m/z calculated for $C_{29}H_{40}O_1Si_1Na_1$ $[M+Na]^+$: 455.2741, found: 455.2739.

(Z)-6-cyclohexylidene-3-methylhex-2-en-1-ol (**S46**)

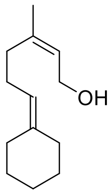

In a flame-dried round-bottom flask under Ar, protected alcohol **S45** (708 mg, 1.64 mmol, 1.0 equiv.) was dissolved in THF (6.8 mL) and TBAF (2.5 M in THF, 2.5 mL, 2.5 mmol, 1.5 equiv.) was added dropwise to the reaction at room temperature. The reaction was stirred until full conversion (monitored by TLC, $KMnO_4$) and was quenched with sat. aq. $NaHCO_3$. The layers were separated and the aqueous layer was extracted with CH_2Cl_2. The combined organic layers were washed with brine and dried over Na_2SO_4 and the solvent was removed under reduced pressure. Purification by FCC (spherical silica gel, 20:1 hexane:EtOAc) afforded compound **S46** (260 mg, 82% yield) as a colorless liquid.

R*f* = 0.21 (10:1 hexane:EtOAc).
^{1}H NMR (300 MHz, $CDCl_3$) δ_H = 5.50 – 5.40 (m, 1H), 5.09 – 5.00 (m, 1H), 4.13 – 4.05 (m, 2H), 2.17 – 2.01 (m, 8H), 1.78 – 1.62 (m, 3H), 1.59 – 1.42 (m, 6H), 1.22 – 1.10 (m, OH) ppm.
^{13}C NMR (75 MHz, $CDCl_3$) δ_C = 140.9, 140.0, 124.6, 120.5, 59.2, 37.2, 32.5, 28.8, 28.8, 28.0, 27.0, 25.7, 23.6 ppm.
HRMS (GC-EI) m/z calculated for $C_{13}H_{22}O_1$ $[M]^+$: 194.1665, found: 194.1664.

(Z)-6-cyclohexylidene-3-methylhex-2-enal (**S47**)

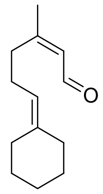

The title compound was prepared according to the general procedure *GP–6* with **S46** (115 mg, 0.592 mmol), $NaHCO_3$ (500 mg, 5.95 mmol) and DMP (518 mg, 2.06 mmol). Purification by FCC (spherical silica gel, 10:1 pentane:Et_2O) yielded the desired compound **S47** (96 mg, 84% yield) as a colorless oil.

R_f = 0.14 (20:1 hexane:Et_2O).

^{1}H NMR (501 MHz, CD_2Cl_2) δ_H = 9.88 (d, *J* = 8.3 Hz, 1H), 5.83 (d, *J* = 8.2 Hz, 1H), 5.06 (t, *J* = 7.5 Hz, 1H), 2.59 (t, *J* = 7.4 Hz, 2H), 2.25 (q, *J* = 7.4 Hz, 2H), 2.10 (t, *J* = 5.7 Hz, 2H), 2.05 (t, *J* = 5.9 Hz, 2H), 1.98 – 1.96 (m, 3H), 1.58 – 1.44 (m, 6H) ppm.

^{13}C NMR (126 MHz, CD_2Cl_2) δ_C = 191.0, 164.1, 142.1, 128.9, 119.4, 37.5, 33.3, 29.1, 28.9, 28.3, 27.2, 26.5, 25.2 ppm.

HRMS (GC-EI) m/z calculated for $C_{13}H_{20}O_1$ $[M]^+$: 192.1509, found: 192.1506.

1.3.7. Cyclization of α,β-unsaturated aldehydes

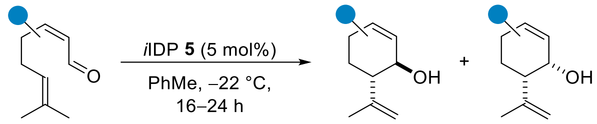

General procedure for the cyclization of α,β-unsaturated aldehydes (GP–7): In an oven-dried flask/vial, *i*IDP catalyst **5** was dissolved in PhMe (0.05 M) and was cooled to –22 °C. After 20 minutes the α,β-unsaturated aldehyde was dropwise added in one portion. The reaction was quenched with triethylamine and warmed up to room temperature. Unless otherwise stated, the solvent was removed (40 °C, 40 mbar) and purification of the crude mixture by FCC (silica gel, pentane:Et_2O) afforded the corresponding cyclic allylic alcohols. Reaction time, yields, diastereomeric and enantiomeric ratios are given for each individual reaction.

Racemate synthesis: The racemates were prepared according to general procedure *GP–7* using a racemic mixture of the *i*IDP catalyst **5**. The reactions were performed on 0.029 mmol scale. After evaporation of the solvent, purification of the crude mixture by preparative TLC (silica gel, 10:1 hexane:EtOAc, partially stained with $KMnO_4$) afforded racemic cyclic allylic alcohols.

3-ethyl-6-(prop-1-en-2-yl)cyclohex-2-en-1-ol (**16**)

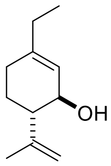

The title compound was prepared according to the general procedure *GP–7* with (*Z*)-3-ethyl-7-methylocta-2,6-dienal (**S19**) (44.5 mg, 0.268 mmol), *i*IDP catalyst **5** (24.6 mg, 5 mol%) in 16 h. Purification by automated FCC (spherical silica gel, pentane:Et_2O 10:1) afforded compound **16** as a colourless oil and as a mixture of diastereoisomers (31.9 mg, 72% yield, d.r. (*trans*:*cis*) = 17:1; e.r. (*trans*) = 99:1).

R_f = 0.29 (10:1 hexane:EtOAc).

^{1}H NMR (501 MHz, CD_2Cl_2, *major*) δ_H = 5.41 – 5.38 (m, 1H), 4.88 – 4.85 (m, 1H), 4.83 – 4.81 (m, 1H), 4.10 (d, *J* = 9.1 Hz, 1H), 2.13 – 1.92 (m, 5H), 1.76 – 1.69 (m, 5H), 1.65 – 1.55 (m, 1H), 1.02 (t, *J* = 7.5 Hz, 3H) ppm.

^{13}C NMR (126 MHz, CD_2Cl_2, *major*) δ_C = 147.4, 142.3, 123.3, 112.1, 69.3, 51.7, 30.0, 29.0, 26.9, 19.6, 12.3 ppm.

HRMS (GC-EI) m/z calculated for $C_{11}H_{18}O_1$ $[M]^+$: 166.1352, found: 166.1353.

GC (chiral) (Hydrodex-beta-TBDAc-CD 0.25/?df; 220/ 70, 420min iso 8/min 220/350, 0.5 bar H_2): t_{trans} = 235.45 min (93.25%), t_{trans} = 270.73 min (1.05%), t_{cis} = 282.91 min (3.83%),

t_{cis} = 364.91 min (1.86%).
$[\alpha]_D^{25}$ = –21.0 (c = 0.18, $CHCl_3$).

6-(prop-1-en-2-yl)-3-propylcyclohex-2-en-1-ol (**17**)

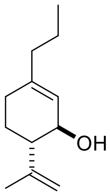

The title compound was prepared according to the general procedure *GP–7* with (Z)-7-methyl-3-propylocta-2,6-dienal (**S20**) (48.9 mg, 0.271 mmol), *i*IDP catalyst **5** (25.8 mg, 5 mol%) in 16 h. Purification by automated FCC (spherical silica gel, pentane:Et_2O 10:1) afforded compound **17** as a colourless oil and as mixture of diastereoisomers (36.9 mg, 75% yield, d.r. (*trans*:*cis*) = 15:1; e.r. (*trans*) = 99:1).

$\mathbf{R}_f$ = 0.34 (10:1 hexane:EtOAc).
^{1}H NMR (501 MHz, CD_2Cl_2, *major*) δ_H = 5.42 – 5.38 (m, 1H), 4.88 – 4.85 (m, 1H), 4.84 – 4.80 (m, 1H), 4.10 (d, *J* = 8.9 Hz, 1H), 2.12 – 2.01 (m, 2H), 1.99 – 1.91 (m, 3H), 1.75 – 1.67 (m, 5H), 1.64 – 1.54 (m, 1H), 1.44 (h, *J* = 7.4 Hz, 2H), 0.89 (t, *J* = 7.3 Hz, 3H) ppm.
^{13}C NMR (126 MHz, CD_2Cl_2, *major*) δ_C = 147.4, 140.6, 124.6, 112.1, 69.2, 51.6, 39.5, 28.8, 26.9, 21.1, 19.7, 14.0 ppm.
HRMS (GC-EI) m/z calculated for $C_{12}H_{20}O_1$ $[M]^+$: 180.1509, found: 180.1508;
GC (chiral) (BGB-174/BGB-1701 0.25/0.25df; 220/ 100, 76 min iso 8/min 240, 3 min iso/ 350, 0.6 bar H_2): t_{trans} = 57.90 min (92.77%), t_{trans} = 61.09 min (1.14%), t_{cis} = 65.01 min (3.86%), t_{cis} = 71.52 min (2.23%).
$[\alpha]_D^{25}$ = –18.7 (c = 0.23, $CHCl_3$).

3-isobutyl-6-(prop-1-en-2-yl)cyclohex-2-en-1-ol (**18**)

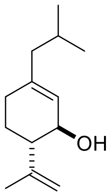

The title compound was prepared according to the general procedure *GP–7* with (*E*)-3-isobutyl-7-methylocta-2,6-dienal (**S21**) (53.1 mg, 0.273 mmol), *i*IDP catalyst **5** (23.7 mg, 5 mol%) in 16 h. Purification by automated FCC (spherical silica gel, pentane:Et_2O 10:1) afforded compound **18** as a colourless solid and as mixture of diastereoisomers (42.6 mg, 80% yield, d.r. (*trans*:*cis*) = 10:1; e.r. (*trans*) = 98:2).

$\mathbf{R}_f$ = 0.33 (10:1 hexane:EtOAc).
^{1}H NMR (501 MHz, CD_2Cl_2, *major*) δ_H = 5.41 – 5.36 (m, 1H), 4.89 – 4.85 (m, 1H), 4.83 – 4.80 (m, 1H), 4.11 (d, *J* = 9.1 Hz, 1H), 2.08 – 1.98 (m, 2H), 1.97 – 1.90 (m, 1H), 1.85 (dd, *J* = 7.4, 3.8 Hz, 2H), 1.80 – 1.66 (m, 6H), 1.64 – 1.54 (m, 1H), 0.86 (dd, *J* = 6.5, 5.4 Hz, 6H) ppm.

^{13}C NMR (126 MHz, CD_2Cl_2, *major*) δ_C = 147.4, 139.7, 125.9, 112.1, 69.2, 51.6, 47.1, 28.8, 26.9, 26.4, 22.7, 22.5, 19.7 ppm.

HRMS (GC-EI) m/z calculated for $C_{13}H_{22}O_1$ $[M]^+$: 194.1665, found: 194.1665.

GC (chiral) (IVADEX-1/PS086 0.25/0.15df; 220/ 100, 65 min iso 8/min 220/350, 0.5 bar H_2): t_{trans} = 50.02 min (1.61%), t_{cis} = 53.26 min (4.24%), t_{trans} = 54.52 min (88.74%), t_{cis} = 57.67 min (5.41%).

$[\alpha]_D^{25}$ = –14.4 (c = 0.32, $CHCl_3$).

3-benzyl-6-(prop-1-en-2-yl)cyclohex-2-en-1-ol (**19**)

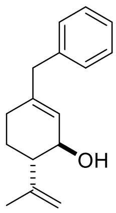

The title compound was prepared according to the general procedure *GP–7* with (E)-3-benzyl-7-methylocta-2,6-dienal (**S22**) (59.3 mg, 0.260 mmol), *i*IDP catalyst **5** (24.0 mg, 5 mol%) in 16 h. Purification by automated FCC (spherical silica gel, gradient 10:1 to 7:1 pentane:Et_2O) afforded compound **19** as a colourless solid and as mixture of diastereoisomers (54.8 mg, 92% yield, d.r. (*trans*:*cis*) = 16:1; e.r. (*trans*) = 98.5:1.5).

R*f* = 0.22 (10:1 hexane:EtOAc).

^{1}H NMR (501 MHz, CD_2Cl_2, *major*) δ_H = 7.31 – 7.26 (m, 2H), 7.22 – 7.17 (m, 3H), 5.49 – 5.45 (m, 1H), 4.88 – 4.85 (m, 1H), 4.83 – 4.81 (m, 1H), 4.17 – 4.10 (m, 1H), 3.32 (d, *J* = 14.6 Hz, 1H), 3.27 (d, *J* = 14.7 Hz, 1H), 2.05 (ddd, *J* = 12.2, 8.9, 3.0 Hz, 1H), 2.02 – 1.96 (m, 1H), 1.95 – 1.87 (m, 1H), 1.73 (d, *J* = 4.1 Hz, 1H), 1.72 – 1.71 (m, 3H), 1.71 – 1.66 (m, 1H), 1.62 – 1.52 (m, 1H) ppm.

^{13}C NMR (126 MHz, CD_2Cl_2, *major*) δ_C = 147.2, 140.1, 140.0, 129.3, 128.7, 126.5, 126.5, 112.2, 69.2, 51.5, 43.9, 28.6, 26.8, 19.6 ppm.

HRMS (GC-EI) m/z calculated for $C_{16}H_{20}O_1$ $[M]^+$: 228.1509, found: 228.1509.

GC (chiral) (BGB-174/BGB-1701 0.25/0.25df; 220/ 130, 400 min iso 8/min 240/350, 0.5 bar H_2): t_{trans} = 303.07 min (1.23%), t_{cis} = 327.12 min (1.89%), t_{trans} = 335.03 min (92.82%), t_{cis} = 369.23 min (4.05%).

$[\alpha]_D^{25}$ = –11.4 (c = 0.28, $CHCl_3$).

3-phenethyl-6-(prop-1-en-2-yl)cyclohex-2-en-1-ol (**20**)

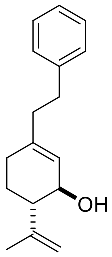

The title compound was prepared according to the general procedure *GP–7* with (E)-7-methyl-3-phenethylocta-2,6-dienal (**S23**) (65.4 mg, 0.270 mmol), *i*IDP catalyst **5** (24.5 mg, 5 mol%) in 16 h. Purification by automated FCC (spherical silica gel, 10:1 pentane:Et_2O) afforded compound **20** as a colourless liquid and as mixture of diastereoisomers (52.4 mg, 80% yield, d.r. (*trans*:*cis*) = 15:1; e.r. (*trans*) = 99:1).

R_f = 0.24 (10:1 hexane:EtOAc).

^{1}H NMR (501 MHz, CD_2Cl_2, *major*) δ_H = 7.30 – 7.25 (m, 2H), 7.23 – 7.15 (m, 3H), 5.46 – 5.43 (m, 1H), 4.89 – 4.86 (m, 1H), 4.84 – 4.82 (m, 1H), 4.10 (d, *J* = 9.1 Hz, 1H), 2.79 – 2.71 (m, 2H), 2.33 – 2.25 (m, 2H), 2.20 – 1.96 (m, 3H), 1.78 – 1.68 (m, 5H), 1.67 – 1.56 (m, 1H) ppm.

^{13}C NMR (126 MHz, CD_2Cl_2, *major*) δ_C = 147.3, 142.7, 140.1, 128.7, 128.6, 126.1, 125.1, 112.2, 69.2, 51.5, 39.2, 34.6, 29.1, 26.8, 19.7 ppm.

HRMS (GC-EI) m/z calculated for $C_{17}H_{22}O_1$ $[M]^+$: 242.1665, found: 242.1668.

GC (chiral) (IVADEX-3 0.25/0.25df; 220/ 120, 800 min iso 8 min 220/350, 0.5 bar H_2): t_{trans} = 545.51 min (93.58%), t_{cis} = 614.15 min (3.21%), t_{trans} = 657.64 min (0.94%), t_{cis} = 720.77 min (2.28%).

$[\alpha]_D^{25}$ = –10.4 (c = 0.21, $CHCl_3$).

6-(prop-1-en-2-yl)-3-(5-(triethylsilyl)pent-4-yn-1-yl)cyclohex-2-en-1-ol (**21**)

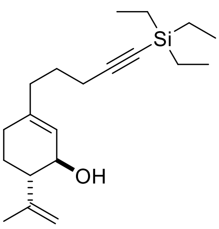

The title compound was prepared according to the general procedure *GP–7* with (Z)-7-methyl-3-(5-(triethylsilyl)pent-4-yn-1-yl)octa-2,6-dienal (**S25**) (82.5 mg, 0.259 mmol), *i*IDP catalyst **5** (22.7 mg, 5 mol%) in 16 h. Purification by automated FCC (spherical silica gel, 10:1 pentane:Et_2O) afforded compound **22** as a colourless liquid and as mixture of diastereoisomers (75.6 mg, 92% yield, d.r. (*trans*:*cis*) = 16:1; e.r. (*trans*) = 99:1).

R_f = 0.31 (10:1 hexane:EtOAc).

^{1}H NMR (501 MHz, CD_2Cl_2, *major*) δ_H = 5.45 – 5.41 (m, 1H), 4.88 – 4.85 (m, 1H), 4.84 – 4.81 (m, 1H), 4.15 – 4.04 (m, 1H), 2.23 (t, *J* = 7.0 Hz, 2H), 2.14 – 2.01 (m, 4H), 1.99 – 1.92 (m, 1H), 1.76 – 1.69 (m, 5H), 1.68 – 1.54 (m, 3H), 0.98 (t, *J* = 7.9 Hz, 9H), 0.57 (q, *J* = 7.9 Hz, 6H) ppm.

^{13}C NMR (126 MHz, CD_2Cl_2, *major*) δ_C = 147.3, 139.8, 125.2, 112.1, 108.8, 82.1, 69.2, 51.5, 36.2, 28.9, 27.3, 26.8, 19.8, 19.7, 7.7, 4.9 ppm.

HRMS (ESI) m/z calculated for $C_{20}H_{34}O_1Si_1Na_1$ $[M+Na]^+$: 341.2271, found: 341.2274.

GC (chiral) (BGB-175/BGB-1701 0.25/0.25df; 220/ 170, 195 min iso 8/min 240, 3 min iso/ 350, 0.5 bar H_2): t_{trans} = 162.72 min (0.82%), t_{trans} = 171.99 min (93.34%), t_{cis} = 176.00 min (2.59%), t_{cis} = 188.05 min (3.25%).

$[\alpha]_D^{25}$ = –12.2 (c = 0.39, $CHCl_3$).

3-cyclopentyl-6-(prop-1-en-2-yl)cyclohex-2-en-1-ol (**22**)

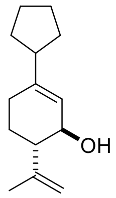

The title compound was prepared according to the general procedure *GP–7* with (E)-3-cyclopentyl-7-methylocta-2,6-dienal (**S24**) (55.4 mg, 0.269 mmol), *i*IDP catalyst **5** (24.0 mg, 5 mol%) in 16 h. Purification by automated FCC (spherical silica gel, 10:1 pentane:Et_2O) afforded compound **21** as a colourless liquid and as mixture of diastereoisomers (40.7 mg, 73% yield, d.r. (*trans*:*cis*) = 12:1; e.r. (*trans*) = 99:1).

$\mathbf{R_f}$ = 0.31 (10:1 hexane:EtOAc).

^{1}H NMR (501 MHz, CD_2Cl_2, *major*) δ_H = 5.45 – 5.41 (m, 1H), 4.87 – 4.84 (m, 1H), 4.84 – 4.81 (m, 1H), 4.13 – 4.08 (m, 1H), 2.35 (p, J = 8.6 Hz, 1H), 2.15 – 1.95 (m, 3H), 1.80 – 1.69 (m, 7H), 1.69 – 1.63 (m, 2H), 1.62 – 1.53 (m, 3H), 1.45 – 1.32 (m, 2H) ppm.

^{13}C NMR (126 MHz, CD_2Cl_2, *major*) δ_C = 147.4, 143.7, 122.6, 112.0, 69.4, 51.8, 47.0, 31.5, 31.2, 27.7, 27.0, 25.5, 19.6 ppm.

HRMS (GC-EI) m/z calculated for $C_{14}H_{22}O_1$ $[M]^+$: 206.1665, found: 206.1664.

GC (chiral) (BGB-175/BGB-1701 0.25/0.25df; 220/130, 120 min iso 8/min 240/350, 0.5 bar H_2): t_{trans} = 91.71 min (1.07%), t_{trans} = 96.79 min (91.46%), t_{cis} = 101.34 min (4.68%), t_{cis} = 111.57 min (2.79%).

$[\alpha]_D^{25}$ = –5.5 (c = 0.15, $CHCl_3$).

4,4-dimethyl-6-(prop-1-en-2-yl)cyclohex-2-en-1-ol (**23**)

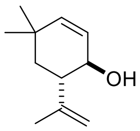

The title compound was prepared according to the general procedure *GP–7* with (*Z*)-4,4,7-trimethylocta-2,6-dienal (**S43**) (5 µL, 4.4 mg, 0.027 mmol), *i*IDP catalyst **5** (2.4 mg, 5 mol%) in 16 h. Mesitylene was added as internal standard to determine the yield by ^{1}H NMR spectroscopy (81% NMR yield). After evaporation of the solvent, purification of the crude mixture by preparative TLC (silica gel, 10:1 hexane:EtOAc, partially stained with $KMnO_4$) afforded compound **23** as an inseparable mixture of diastereoisomers (d.r. (*trans*:*cis*) > 20:1; e.r. (*trans*) = 99:1).

R$_f$ = 0.29 (10:1 hexane:EtOAc).

^{1}H NMR (501 MHz, $CDCl_3$, *major*) δ_H = 5.55 (dd, *J* = 10.0, 1.7 Hz, 1H), 5.44 (dt, *J* = 9.9, 1.6 Hz, 1H), 4.93 – 4.86 (m, 2H), 4.08 (dt, *J* = 9.3, 1.9 Hz, 1H), 2.27 (td, *J* = 9.7, 5.5 Hz, 1H), 1.84 (s, OH), 1.73 (t, *J* = 1.1 Hz, 3H), 1.51 – 1.44 (m, 2H), 1.04 (s, 3H), 1.01 (s, 3H) ppm.

^{13}C NMR (126 MHz, $CDCl_3$, *major*) δ_C = 146.5, 138.8, 127.5, 112.6, 69.0, 48.4, 40.9, 33.3, 31.2, 28.2, 19.4 ppm.

HRMS (GC-EI) m/z calculated for $C_{11}H_{18}O_1$ $[M]^+$: 166.1352, found: 166.1352.

GC (chiral) (BGB-176/BGB-15 0.25/0.25df; 220/ 60 iso/350, 0.6 bar H_2): t_{trans} = 165.36 min (0.70%), t_{trans} = 172.17 min (97.18%), t_{cis} = 188.75 min (1.57%), t_{cis} = 193.07 min (0.55%).

4-methyl-[1,1'-bi(cyclohexane)]-1',3-dien-2-ol (**24**)

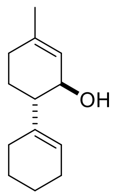

The title compound was prepared according to the general procedure *GP–7* with (*Z*)-6-cyclohexylidene-3-methylhex-2-enal (**S47**) (54.6 mg, 0.284 mmol), *i*IDP catalyst **5** (25.5 mg, 5 mol%) in 24 h. Purification by automated FCC (spherical silica gel, 10:1 pentane:Et_2O) afforded compound **24** as a colorless liquid and as mixture of diastereoisomers (36.2 mg, 66% yield, d.r. (*trans*:*cis*) > 20:1; e.r. (*trans*) = 99:1).

R$_f$ = 0.30 (10:1 hexane:EtOAc).

^{1}H NMR (501 MHz, CD_2Cl_2, *major*) δ_H = 5.55 (tt, *J* = 3.8, 1.8 Hz, 1H), 5.40 – 5.37 (m, 1H), 4.06 (d, *J* = 9.1 Hz, 1H), 2.12 – 1.99 (m, 3H), 1.96 – 1.84 (m, 4H), 1.74 (s, OH), 1.68 (s, 3H), 1.67 – 1.54 (m, 6H) ppm.

^{13}C NMR (126 MHz, CD_2Cl_2, *major*) δ_C = 138.9, 136.6, 125.2, 123.9, 68.9, 51.9, 30.8, 26.7, 25.7, 25.5, 23.4, 23.2, 23.1 ppm.

HRMS (GC-EI) m/z calculated for $C_{13}H_{20}O_1$ $[M]^+$: 192.1509, found: 192.1508.

GC (chiral) (IVADEX-3 0.25/0.25df; 220/ 110, 140 min iso 8/min 220, 3 min iso/ 350, 0.5 bar H_2): t_{trans} = 98.68 min (94.63%), t_{cis} = 112.34 min (3.11%), t_{trans} = 120.56 min (0.87%), t_{cis} = 130.97 min (1.39%).

$[\boldsymbol{\alpha}]_{\boldsymbol{D}}^{\mathbf{25}}$ = –18.9 (c = 0.21, $CHCl_3$).

1.3.8. Synthesis of deuterium-labelled substrate analogue **25**

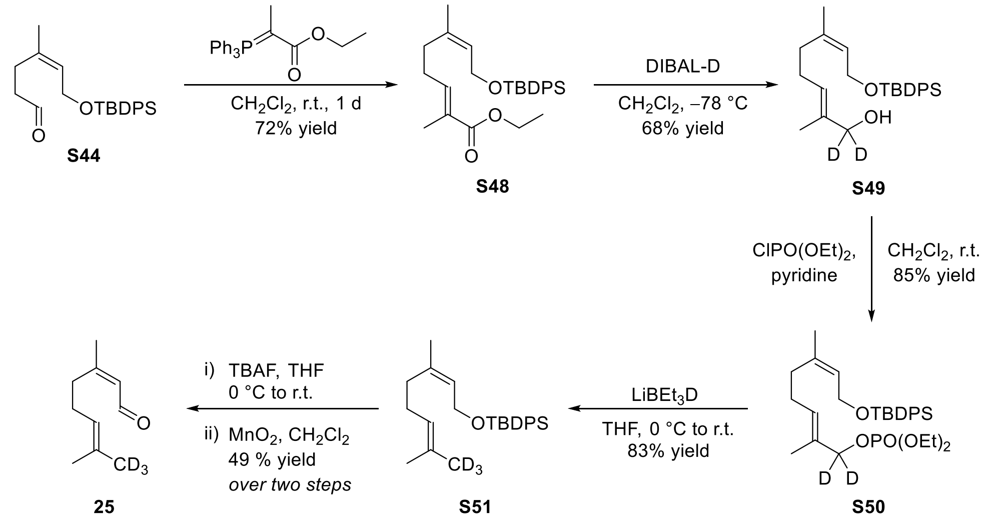

ethyl (2E,6Z)-8-((tert-butyldiphenylsilyl)oxy)-2,6-dimethylocta-2,6-dienoate (**S48**)

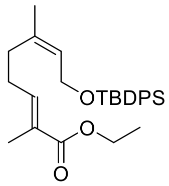

In a flame-dried round-bottom flask under Ar, aldehyde **S44**[26] (2.0 g, 5.5 mmol, 1.0 equiv) was dissolved in CH_2Cl_2 (12 mL) and ethyl-2-(triphenylphosphoranyliden)-propionate (4.0 g, 11 mmol, 2.0 equiv) was added at room temperature. The reaction was stirred until full conversion and was concentrated under reduced pressure. Purification by automated CC (spherical silica gel, 4:1 EtOAc:hexane) afforded compound **S48** (2.50 g, 72% yield) as a colorless liquid.

^{1}H NMR (501 MHz, $CDCl_3$) δ_H = 7.71 – 7.66 (m, 4H), 7.45 – 7.34 (m, 6H), 6.61 (tq, *J* = 7.4, 1.5 Hz, 1H), 5.43 (td, *J* = 6.5, 1.5 Hz, 1H), 4.18 (dd, *J* = 6.5, 1.2 Hz, 2H), 4.14 (q, *J* = 7.1 Hz, 2H), 2.13 (q, *J* = 7.7 Hz, 2H), 2.00 – 1.94 (m, 2H), 1.75 – 1.73 (m, 3H), 1.73 – 1.71 (m, 3H), 1.25 (t, *J* = 7.1 Hz, 3H), 1.04 (s, 9H) ppm.

^{13}C NMR (126 MHz, $CDCl_3$) δ_C = 168.2, 141.3, 136.6, 135.7, 134.1, 129.7, 128.2, 127.8, 127.7, 125.8, 60.7, 60.5, 31.0, 27.4, 27.0, 23.4, 19.3, 14.4, 12.4 ppm.

HRMS (ESI) m/z calculated for $C_{28}H_{38}O_3Si_1Na_1$ $[M+Na]^+$: 473.2482, found: 473.2486.

(2E,6Z)-8-((tert-butyldiphenylsilyl)oxy)-2,6-dimethylocta-2,6-dien-1,1-d_2-1-ol (**S49**)

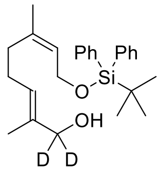

In a flame-dried round-bottom flask under Ar, ester **S48** (2.25 g, 4.99 mol, 1.0 equiv.) was dissolved in CH_2Cl_2 (20 mL) and cooled down to –78 °C. After stirring for 15 min, DIBAL–D (0.7 M in CH_2Cl_2, 9.99 mmol, 14.2 mL) was added dropwise and the reaction was stirred until full conversion of the starting material at –78 °C. The reaction was quenched with a 1:1 mixture H_2O/MeOH, was warmed up

to room temperature and was stirred for 2 h at room temperature. The resulting reaction mixture was filtered over a pad of Na_2SO_4/Celite, which was extensively washed with CH_2Cl_2. The organic layer was washed with brine, dried over Na_2SO_4 and the solvent was removed under reduced pressure. Purification by FCC (spherical silica gel, gradient from 4:1 to 1:1, Et_2O in pentane) afforded protected alcohol **S49** (1.39 g, 68% yield) as a colorless liquid.

^{1}H NMR (501 MHz, $CDCl_3$) δ_H = 7.72 – 7.66 (m, 4H), 7.47 – 7.35 (m, 6H), 5.40 (td, *J* = 6.4, 1.5 Hz, 1H), 5.28 (tq, *J* = 7.1, 1.5 Hz, 1H), 4.20 – 4.16 (dq, *J* = 6.3, 1.2 Hz, 2H), 2.07 – 1.98 (m, 2H), 1.96 – 1.92 (m, 2H), 1.72 – 1.70 (m, 3H), 1.59 – 1.57 (m, 3H), 1.19 (br. s., OH), 1.05 (s, 9H) ppm.

^{13}C NMR (126 MHz, $CDCl_3$) δ_C = 137.2, 135.8, 135.3, 134.1, 129.7, 127.7, 125.7, 125.3, 61.0, 31.9, 27.0, 26.2, 23.5, 19.3, 13.7 ppm.

HRMS (ESI) m/z calculated for $C_{26}H_{34}D_2O_2Si_1Na_1$ $[M+Na]^+$: 433.2502, found: 433.2501.

(2E,6Z)-8-((tert-butyldiphenylsilyl)oxy)-2,6-dimethylocta-2,6-dien-1-yl-1,1-d$_2$ diethyl phosphate (**S50**)

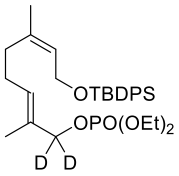

In a flame-dried round-bottom flask under Ar, alcohol **S49** (530 mg, 1.29 mol, 1.0 equiv.) and pyridine (188 µL, 2.32 mmol, 1.8 equiv.) were dissolved in CH_2Cl_2 (4 mL). Diethyl chlorophosphate (280 µL, 1.94 mmol, 1.5 equiv.) was slowly added at 0 °C and the reaction was stirred until full conversion. The reaction was treated with an aq. sat. NH_4Cl, the layers were separated and the organic layer was extracted three times with EtOAc. Purification by FCC (ALOX, gradient from 10:1 to 3:1, hexane:EtOAc) afforded compound **S50** (597 mg, 85% yield) as a colorless liquid.

^{1}H NMR (501 MHz, CD_2Cl_2) δ_H = 7.71 – 7.65 (m, 4H), 7.46 – 7.35 (m, 6H), 5.42 (td, *J* = 6.5, 1.5 Hz, 1H), 5.40 – 5.36 (m, 1H), 4.20 (dd, *J* = 6.5, 1.3 Hz, 2H), 4.09 – 3.99 (m, 4H), 2.09 – 2.01 (m, 2H), 1.97 – 1.90 (m, 2H), 1.74 – 1.71 (m, 3H), 1.61 – 1.58 (s, 3H), 1.29 (td, *J* = 7.1, 0.9 Hz, 6H), 1.03 (s, 9H) ppm.

^{13}C NMR (126 MHz, CD_2Cl_2) δ_C = 137.7, 136.0, 134.4, 131.4, 131.3, 130.0, 129.5, 128.0, 128.0, 125.5, 64.0, 63.9, 61.1, 31.9, 27.0, 26.7, 23.5, 19.4, 16.4, 16.3, 13.6 ppm.

^{31}P NMR (203 MHz, CD_2Cl_2) δ_P = –0.99 ppm.

HRMS (ESI) m/z calculated $C_{30}H_{43}D_2Na_1O_5P_1Si_1$ for $[M+Na]^+$: 569.2792, found: 569.2796.

tert-butyl(((2Z,6E)-3,7-dimethylocta-2,6-dien-1-yl-8,8,8-d_3)oxy)diphenylsilane (**S51**)

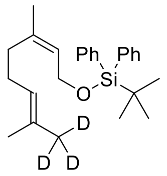

In a flame-dried round-bottom flask under Ar, phosphonate **S50** (250 mg, 0.46 mol, 1.0 equiv.) was dissolved in THF (2.5 mL). A freshly prepared solution of $LiBEt_3D$[27] (1 M in THF, 4.57 mL, 4.57 mmol, 10 equiv.) was added portionwise at 0 °C and the reaction was stirred until full conversion. The reaction was quenched by slow addition of water, layers were separated and the organic layer was extracted three times with hexane. The combined extracts were washed with 1M HCl, sat. aq. $NaHCO_3$, brine, dried over Na_2SO_4 and the solvent was removed under reduced pressure. Purification by FCC (silica gel, gradient from pentane to 10:1 Et_2O:pentane) afforded compound **S51** (150 mg, 83% yield) as a colorless liquid and as a mixture of double-bond isomers (~85:15).

^{1}H NMR (501 MHz, $CDCl_3$, *major*) δ_H = 7.71 – 7.66 (m, 4H), 7.46 – 7.35 (m, 6H), 5.44 – 5.37 (m, 1H), 5.03 – 4.97 (m, 1H), 4.20 (dd, J = 6.5, 1.2 Hz, 2H), 2.02 – 1.94 (m, 2H), 1.91 (ddd, J = 8.8, 6.5, 1.9 Hz, 2H), 1.72 (d, J = 1.3 Hz, 3H), 1.51–1.50 (m, 3H), 1.03 (s, 9H) ppm.
^{13}C NMR (126 MHz, $CDCl_3$) δ_C = 138.1, 136.0, 134.6, 134.5, 129.9, 128.0, 125.2, 124.3, 124.3, 61.5, 61.2, 39.9, 32.6, 30.5, 27.0, 27.0, 26.8, 23.5, 19.4, 17.6, 16.4 ppm.
HRMS (ESI) m/z calculated for $C_{26}H_{33}D_3Na_1O_1Si_1$ $[M+Na]^+$: 418.2616, found: 418.2618.

(2Z,6E)-3,7-dimethylocta-2,6-dienal-8,8,8-d_3 (**25**)

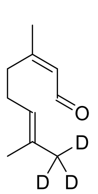

In a flame-dried round-bottom flask under Ar, protected alcohol **S55** (0.14 g, 0.35 mmol, 1.0 equiv.) was dissolved in THF (4 mL). TBAF (1 M in THF, 0.53 mL, 0.53 mmol, 1.5 equiv.) was added to the solution at 0 °C, which was subsequently allowed to warm up overnight. The reaction was diluted with Et_2O and washed three times with sat. aq. NH_4Cl solution. The combined aqueous layers were extracted with Et_2O. The combined organic layers were washed with additional sat. aq. NH_4Cl and brine, dried over Na_2SO_4, filtered and concentrated under reduced pressure. The crude was filtered through a short pad of silica and used in the next oxidation step without further purification.

The crude alcohol was dissolved in CH_2Cl_2 (1 mL) and manganese dioxide (309 mg, 3.56 mmol, 10 equiv.) were added at room temperature. After full conversion of the starting material the reaction suspension was filtered through a pad of celite and washed with CH_2Cl_2. Purification by FCC (spherical silica gel, pentane) afforded deuterated aldehyde **25** (27 mg, 49% yield) as a colorless liquid and as a mixture of Z:E-isomers (Z:E = 70:30).

^{1}H NMR (600 MHz, CD_2Cl_2, *major* (*Z*) and *minor* (*E*)) δ_H = 9.97 (d, *J* = 8.1 Hz, 0.3H, $1H_{min}$), 9.88 (d, *J* = 8.2 Hz, 0.7H, $1H_{maj}$), 5.86 – 5.79 (m, 1H, $1H_{maj}$+$1H_{min}$), 5.16 – 5.05 (m, 1H, $1H_{maj}$+$1H_{min}$), 2.58 (t, *J* = 7.5 Hz, 1.4H, $2H_{maj}$), 2.30 – 2.17 (m, 2.6H, $2H_{maj}$+$3H_{min}$), 2.15 (d, *J* = 1.3 Hz, 0.9H, $3H_{min}$), 1.97 (d, *J* = 1.3 Hz, 2.1H, $3H_{maj}$), 1.68 (dq, *J* = 3.7, 1.2 Hz, 0.5H, $1H_{min}$), 1.61 (dt, *J* = 1.4, 0.7 Hz, 0.8H, $3H_{min}$), 1.60 (dt, *J* = 1.4, 0.7 Hz, 1.8H, $3H_{maj}$) ppm.

^{2}H NMR (92 MHz, CD_2Cl_2) δ_D = 1.60 (s, 3D), 1.54 (s, 0.2D). 1.53 (s, 0.3D) ppm.

^{13}C NMR (151 MHz, CD_2Cl_2) δ_C = 191.4, 190.9, 164.2, 133.8, 133.1, 128.9, 127.7, 123.2, 123.1, 122.9, 122.9, 41.0, 32.9, 32.9, 27.5, 27.4, 26.2, 26.2, 25.7, 25.3, 25.2, 25.1, 25.0, 25.0, 25.0, 25.0, 24.9 (hept, *J* = 19.1 Hz), 24.6, 24.5, 17.8, 17.7 ppm.

HRMS (GC-EI) m/z calculated for $C_{10}H_{13}O_1D_3$ $[M]^{+\cdot}$: 155.1384, found: 155.1384.

1.4. Supplementary data

1.4.1. Copies of NMR spectra

(*S*)-3,3'-bis(2-(5,6,7,8,-tetrafluoro)naphthyl)-[1,1'-binaphthalene]-2,2'-diol (**S10**)

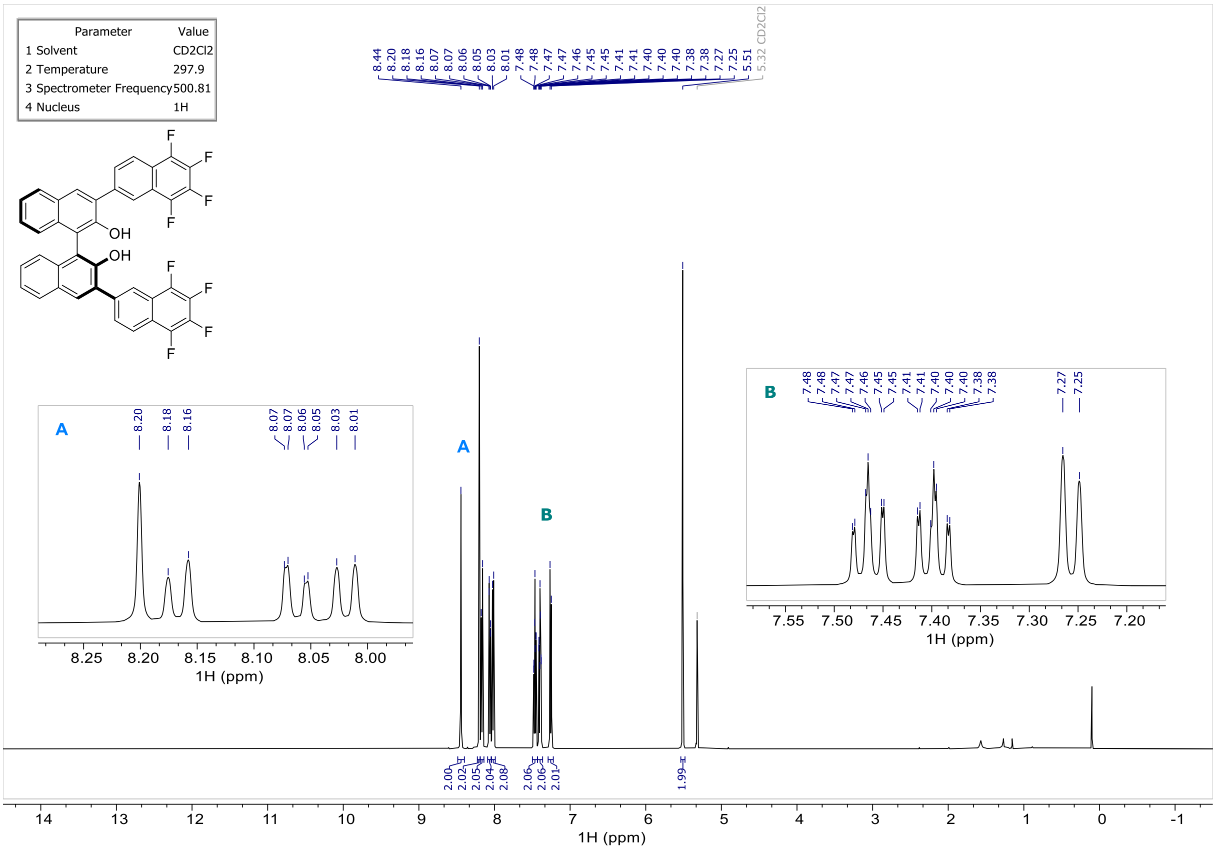

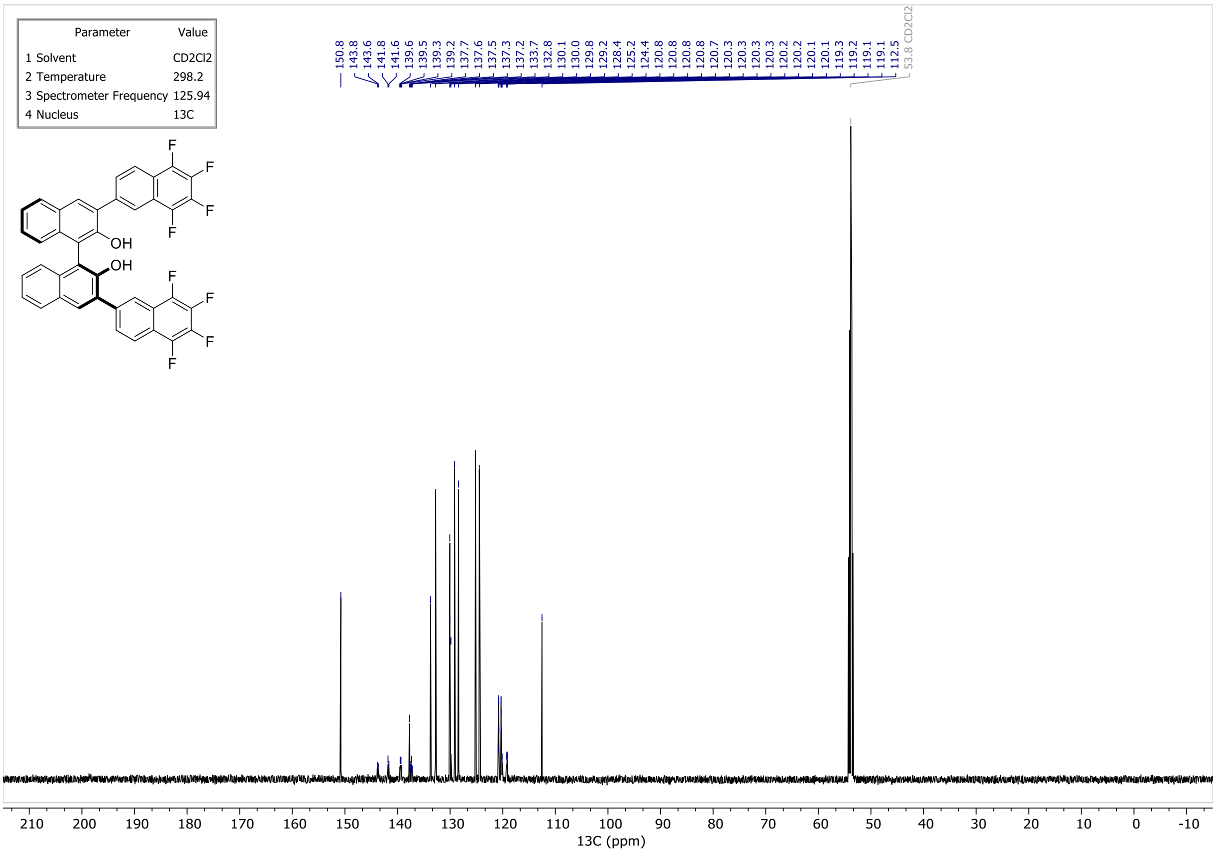
Parameter Value
1 Solvent CD2Cl2
2 Temperature 298.2
3 Spectrometer Frequency 125.94
4 Nucleus 13C
150.8 143.8 143.6 141.8 141.6 139.6 139.5 139.3 139.2 137.7 137.6 137.5 137.3 137.2 133.7 132.8 130.1 130.0 129.8 129.2 128.4 125.2 124.4 120.8 120.8 120.8 120.8 120.7 120.3 120.3 120.3 120.2 120.2 120.1 120.1 119.3 119.2 119.1 119.1 112.5
53.8 CD2Cl2
13C (ppm)

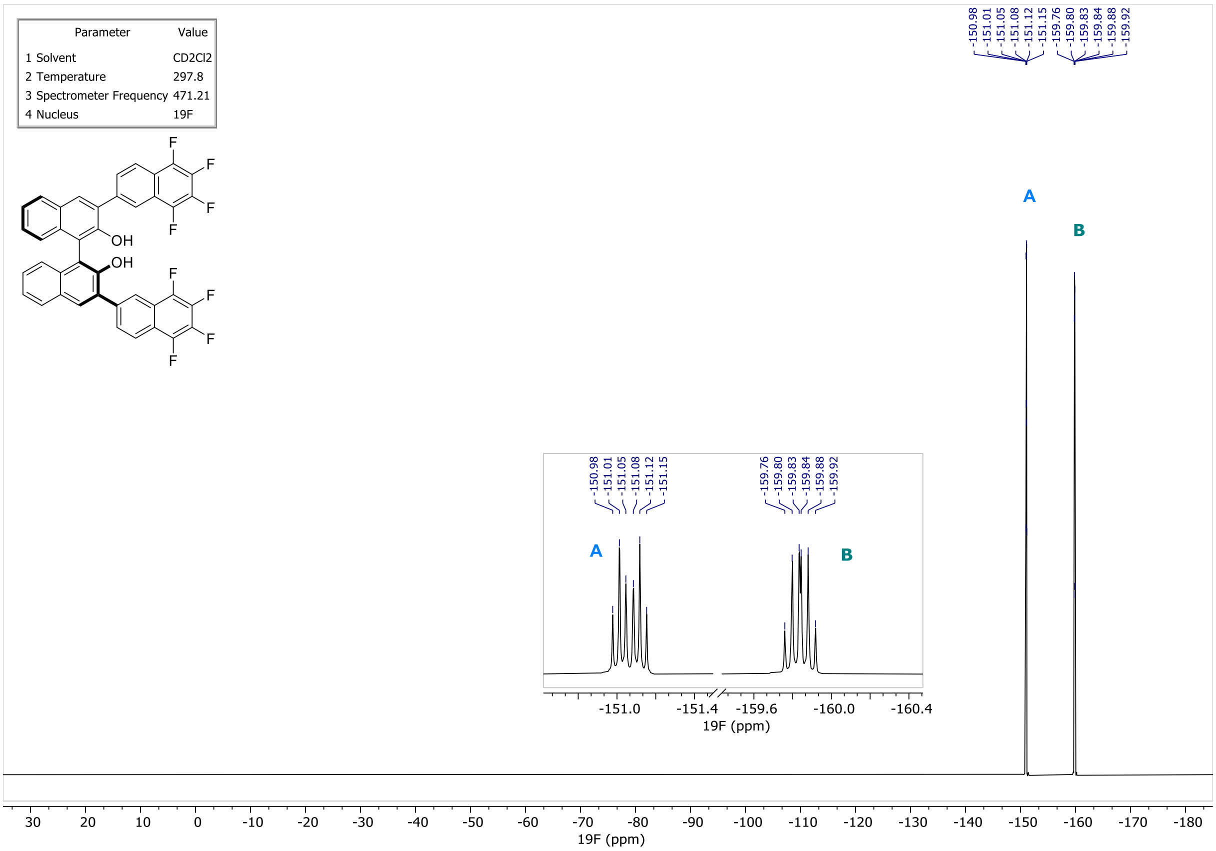
Parameter Value
1 Solvent CD2Cl2
2 Temperature 297.8
3 Spectrometer Frequency 471.21
4 Nucleus 19F
-150.98 -151.01 -151.05 -151.08 -151.12 -151.15 -159.76 -159.80 -159.83 -159.84 -159.88 -159.92
A
B
19F (ppm)

(*S*,*S*)-IDP **4**

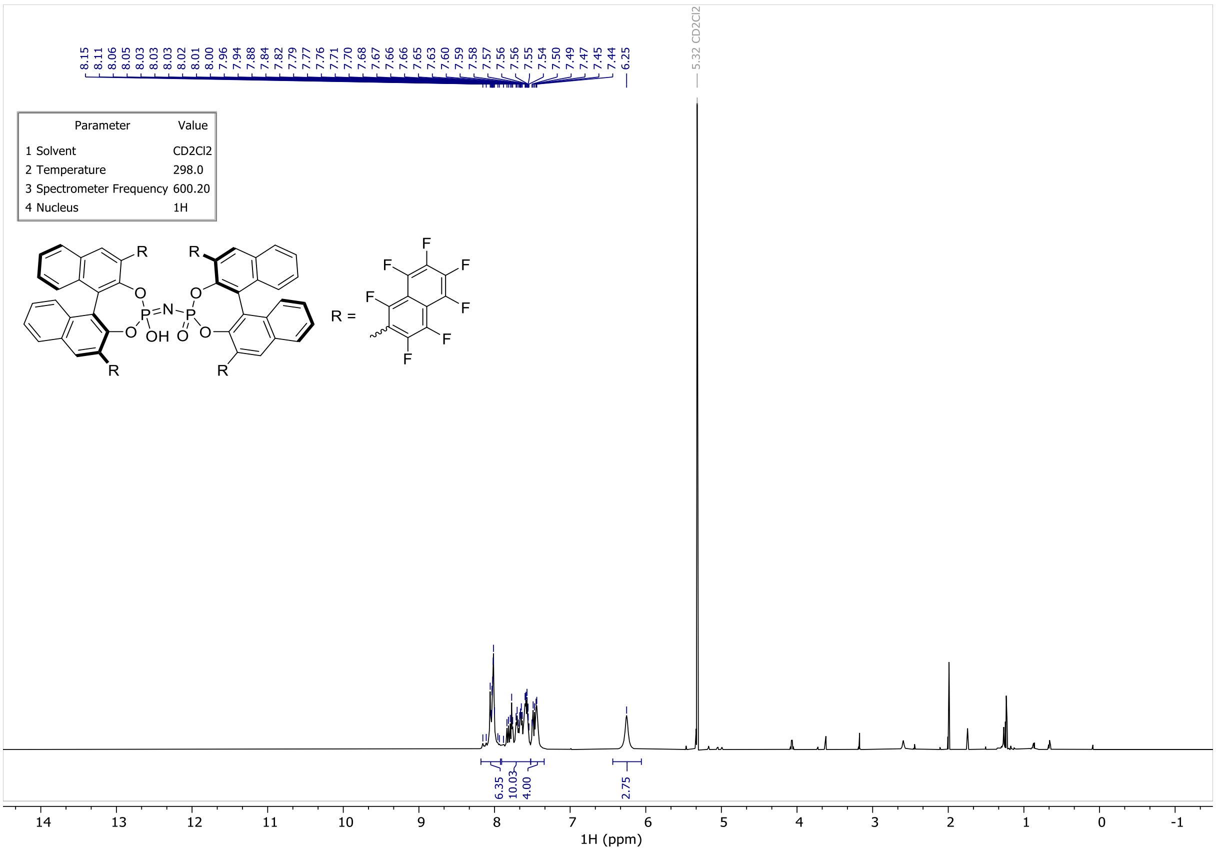
Parameter Value
1 Solvent CD2Cl2
2 Temperature 298.0
3 Spectrometer Frequency 600.20
4 Nucleus 1H
R =
1H (ppm)

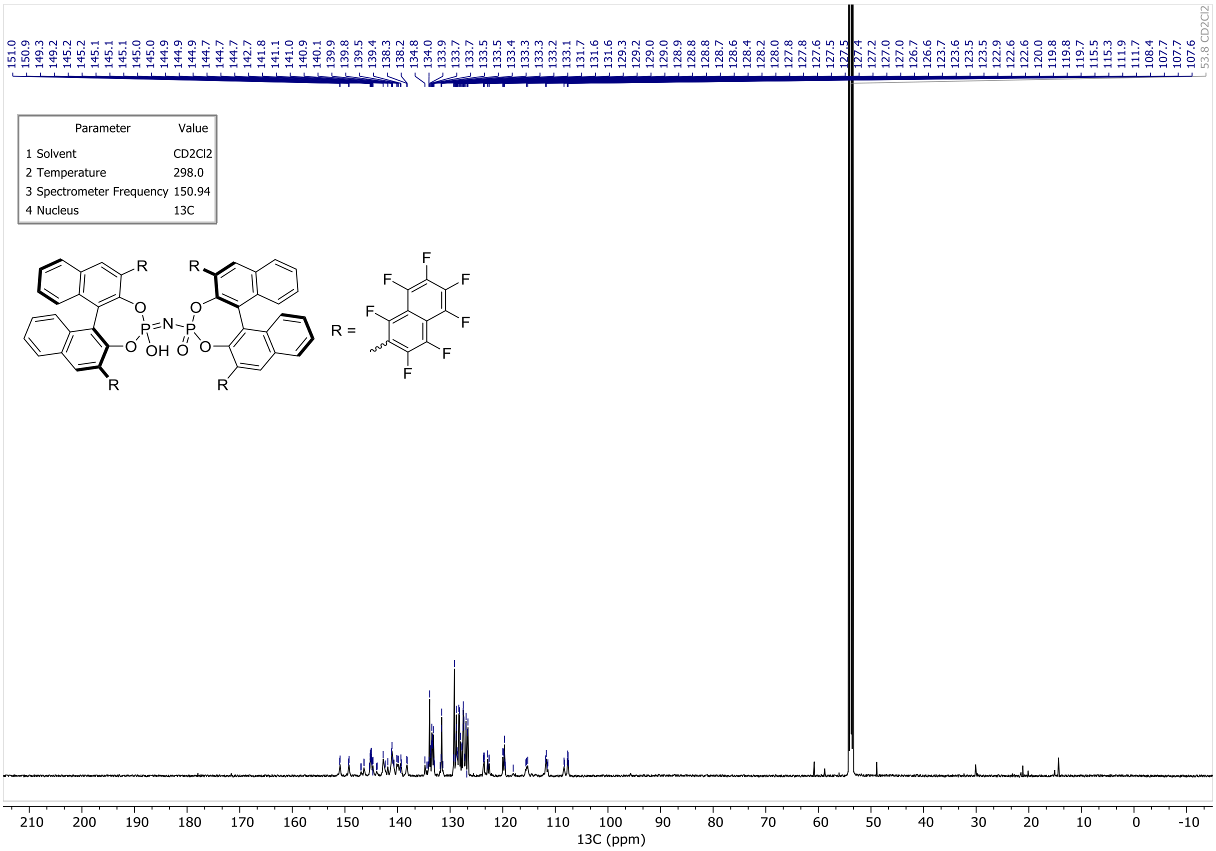
Parameter Value
1 Solvent CD2Cl2
2 Temperature 298.0
3 Spectrometer Frequency 150.94
4 Nucleus 13C
R =
13C (ppm)

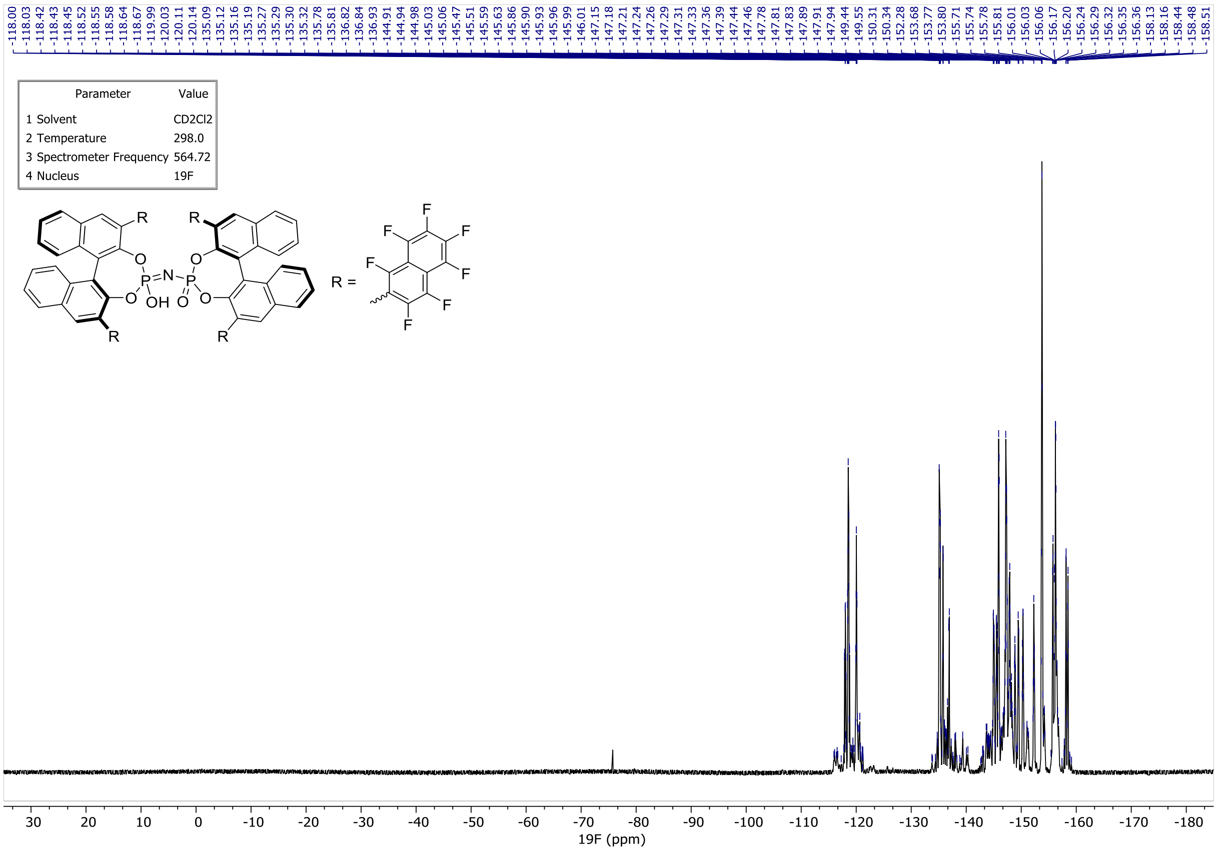

Parameter Value
1 Solvent CD2Cl2
2 Temperature 298.0
3 Spectrometer Frequency 564.72
4 Nucleus 19F
R =
19F (ppm)

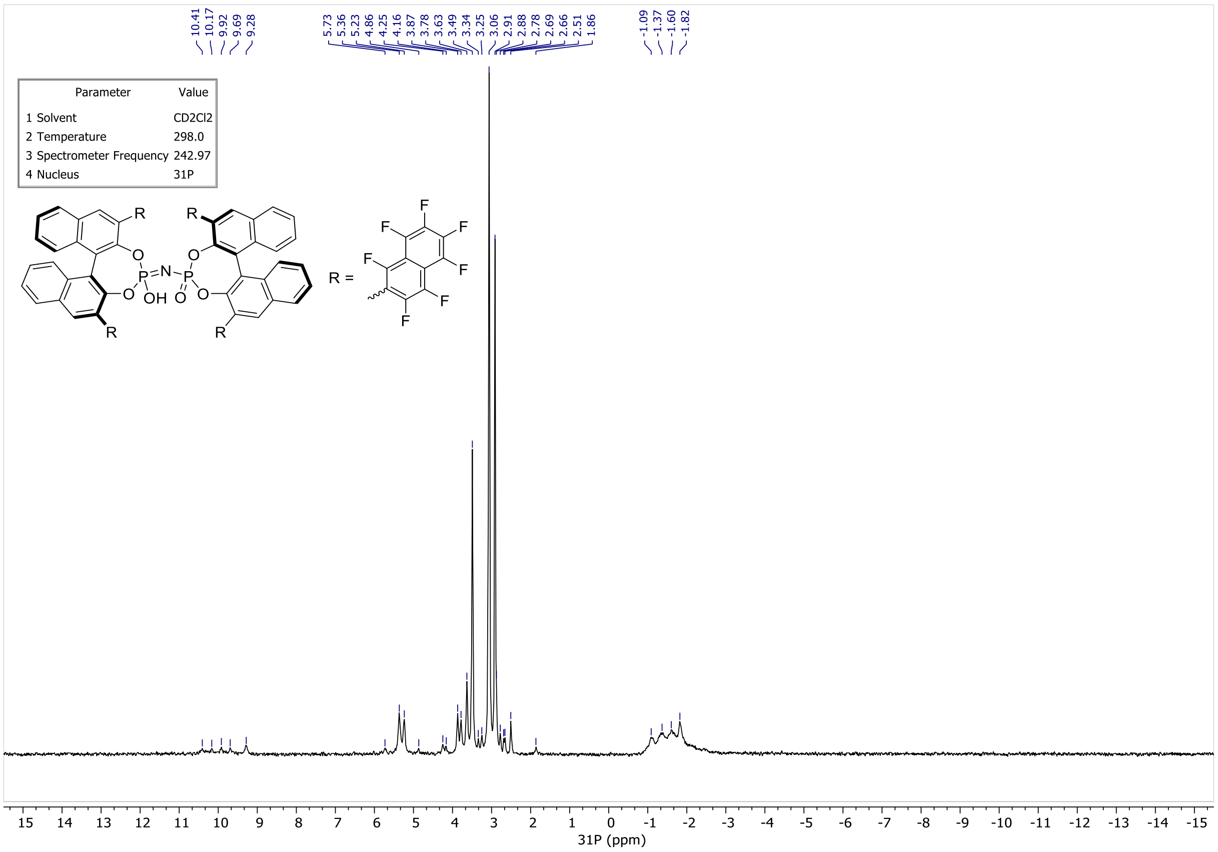

Parameter Value
1 Solvent CD2Cl2
2 Temperature 298.0
3 Spectrometer Frequency 242.97
4 Nucleus 31P
R =
31P (ppm)

(*S*,*S*)-*i*IDP **S7**

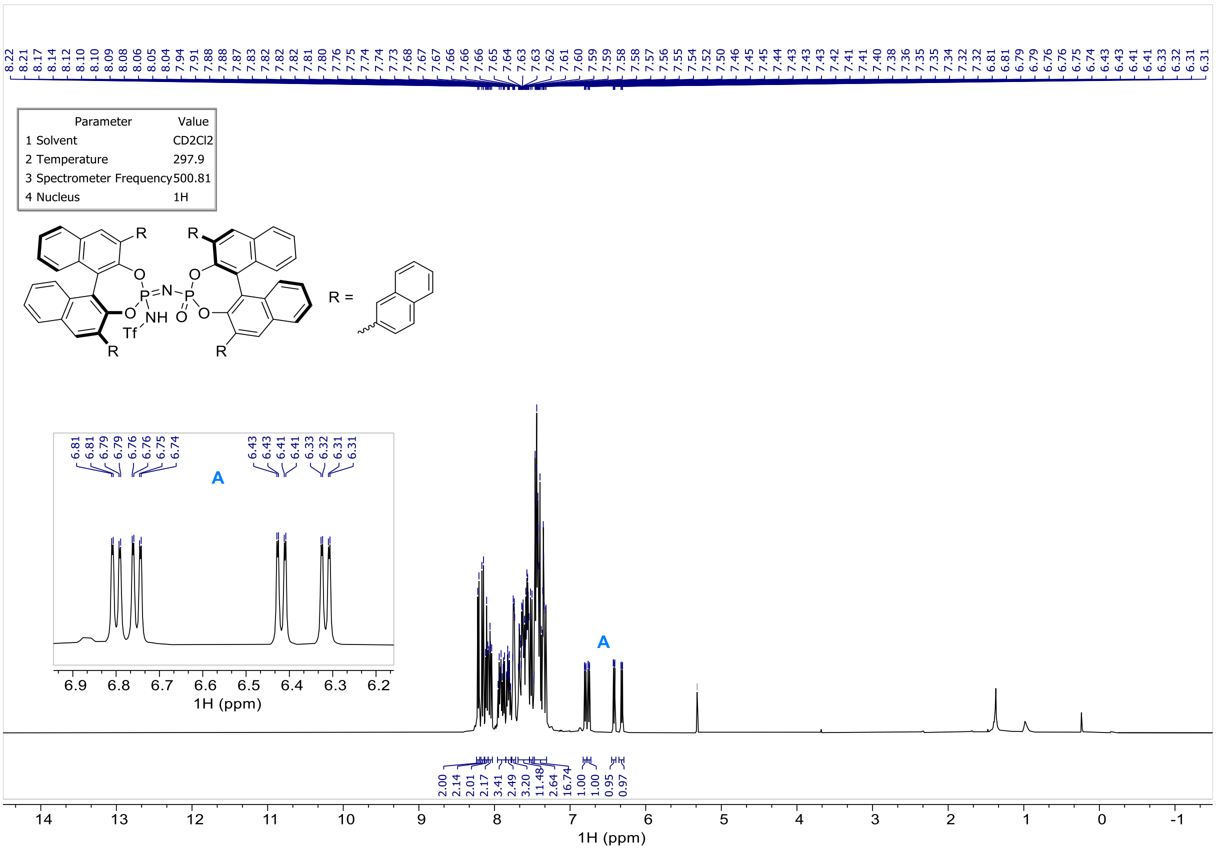
Parameter Value
1 Solvent CD2Cl2
2 Temperature 297.9
3 Spectrometer Frequency 500.81
4 Nucleus 1H
R =
Tf
NH
A
1H (ppm)

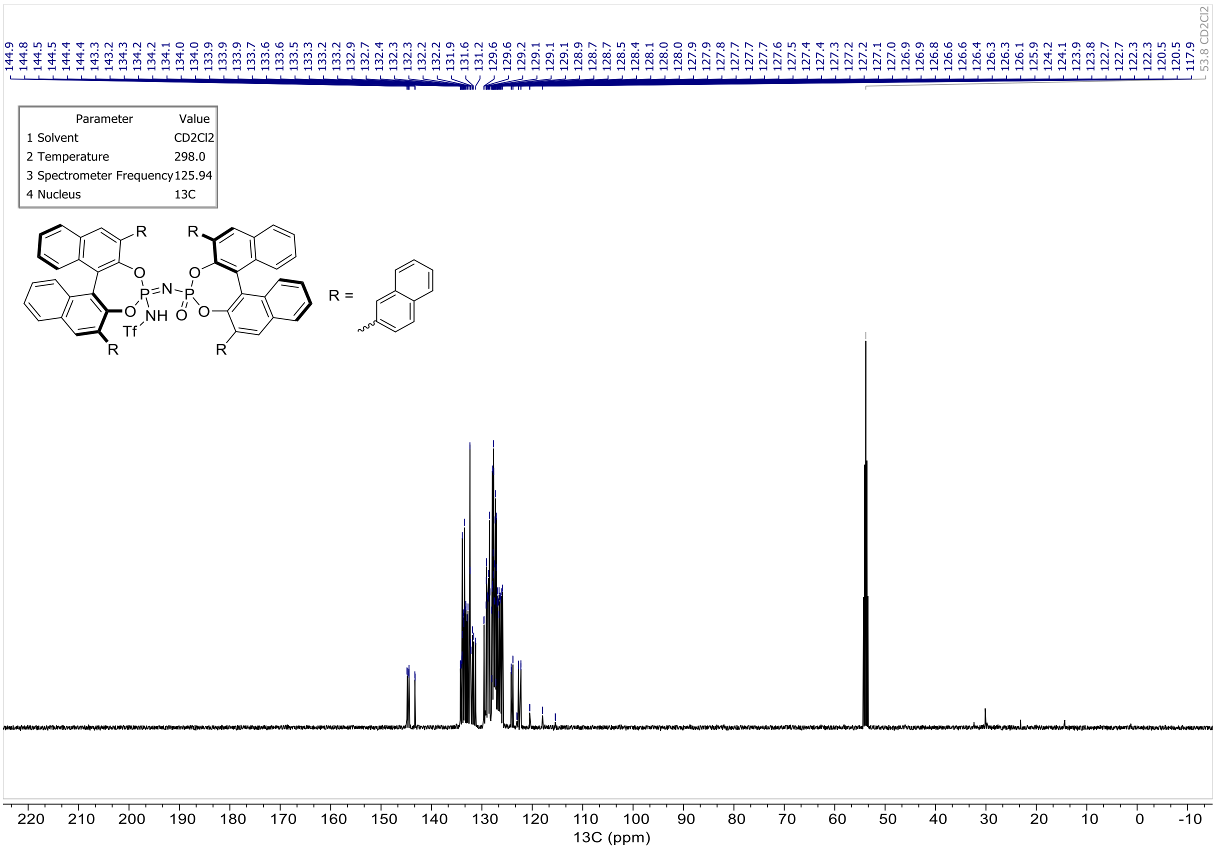
Parameter Value
1 Solvent CD2Cl2
2 Temperature 298.0
3 Spectrometer Frequency 125.94
4 Nucleus 13C
R =
Tf
NH
53.8 CD2Cl2
13C (ppm)

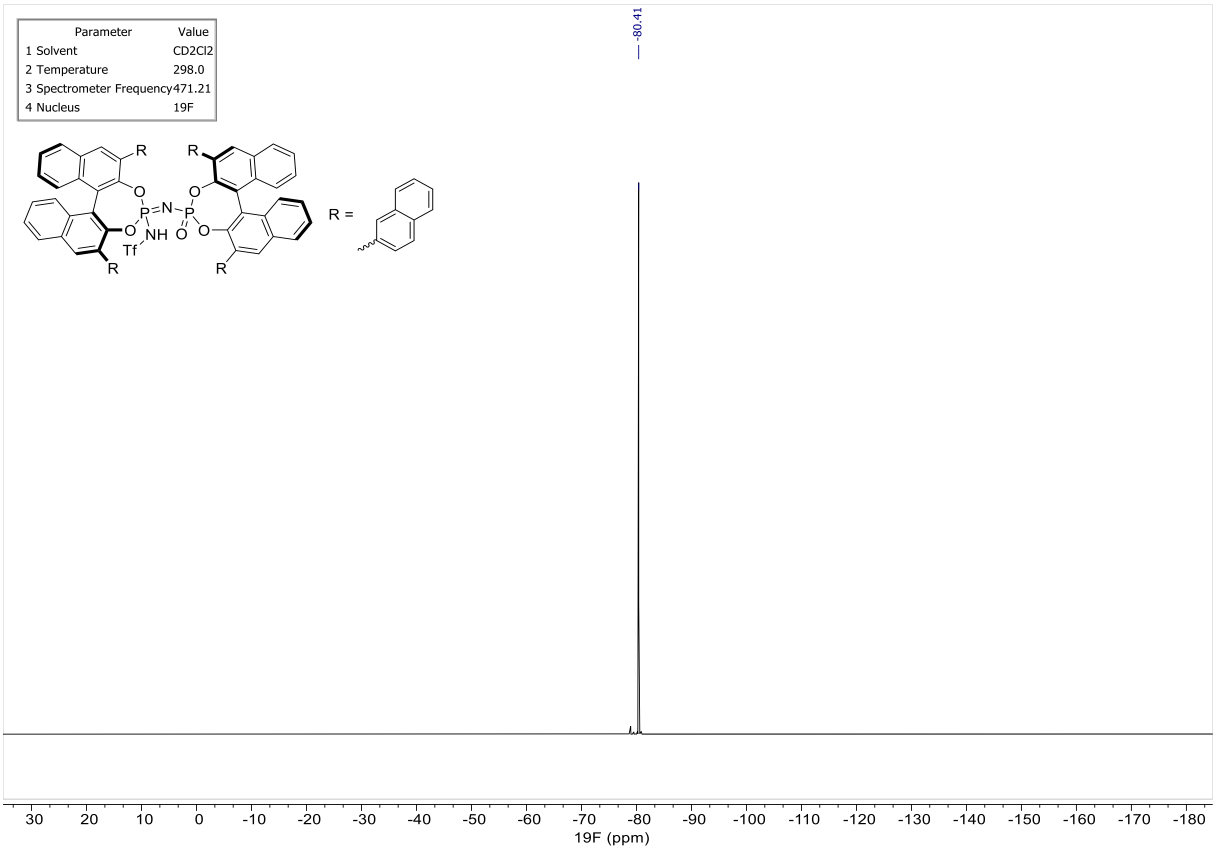
Parameter Value
1 Solvent CD2Cl2
2 Temperature 298.0
3 Spectrometer Frequency 471.21
4 Nucleus 19F
-80.41
R =
19F (ppm)

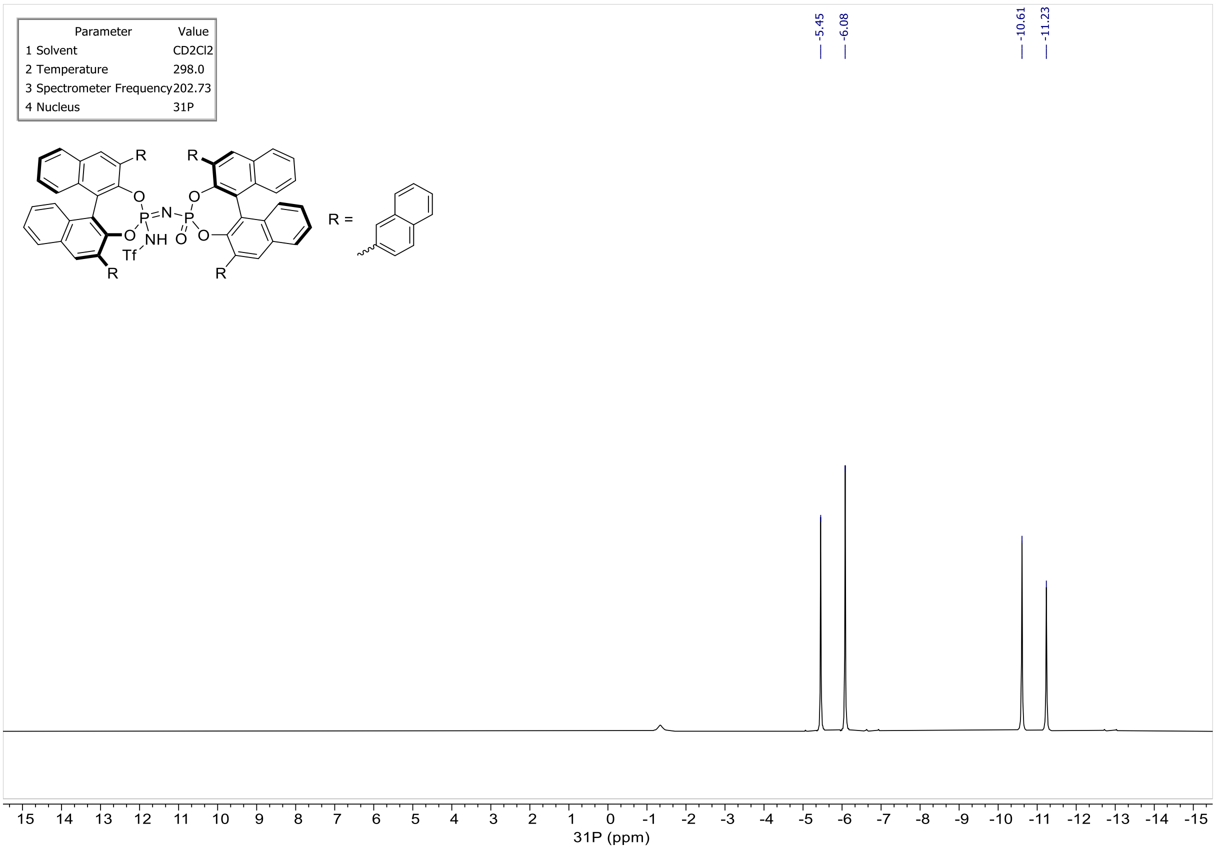
Parameter Value
1 Solvent CD2Cl2
2 Temperature 298.0
3 Spectrometer Frequency 202.73
4 Nucleus 31P
-5.45
-6.08
-10.61
-11.23
R =
31P (ppm)

(*S*,*S*)-*i*IDP **S9**

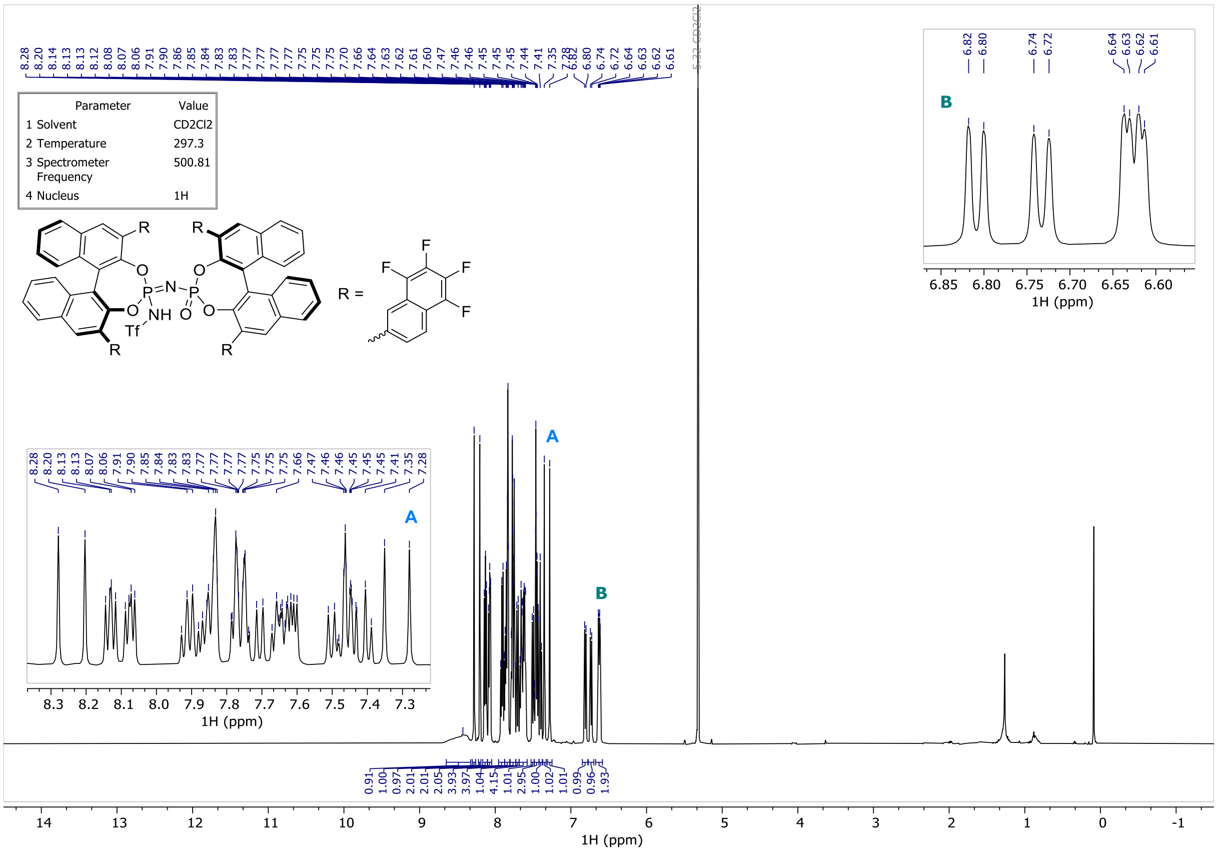

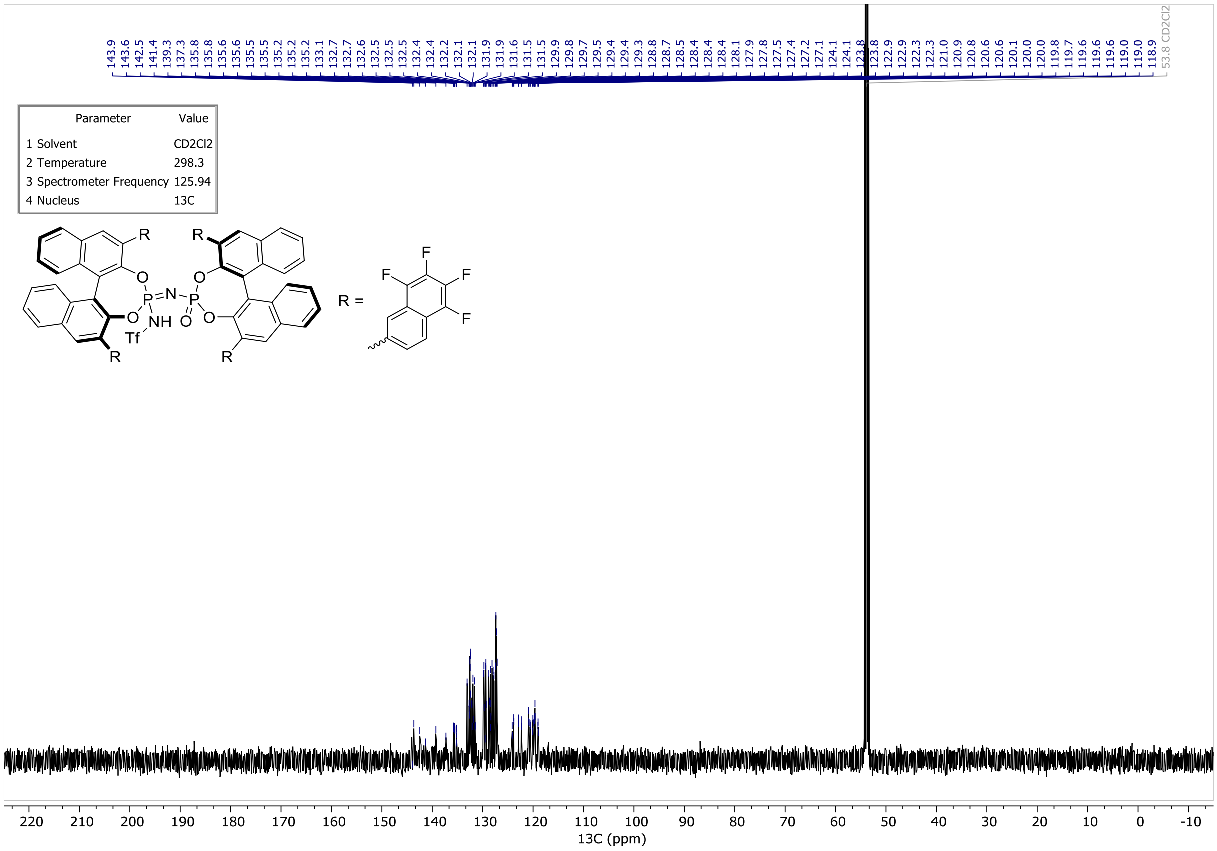

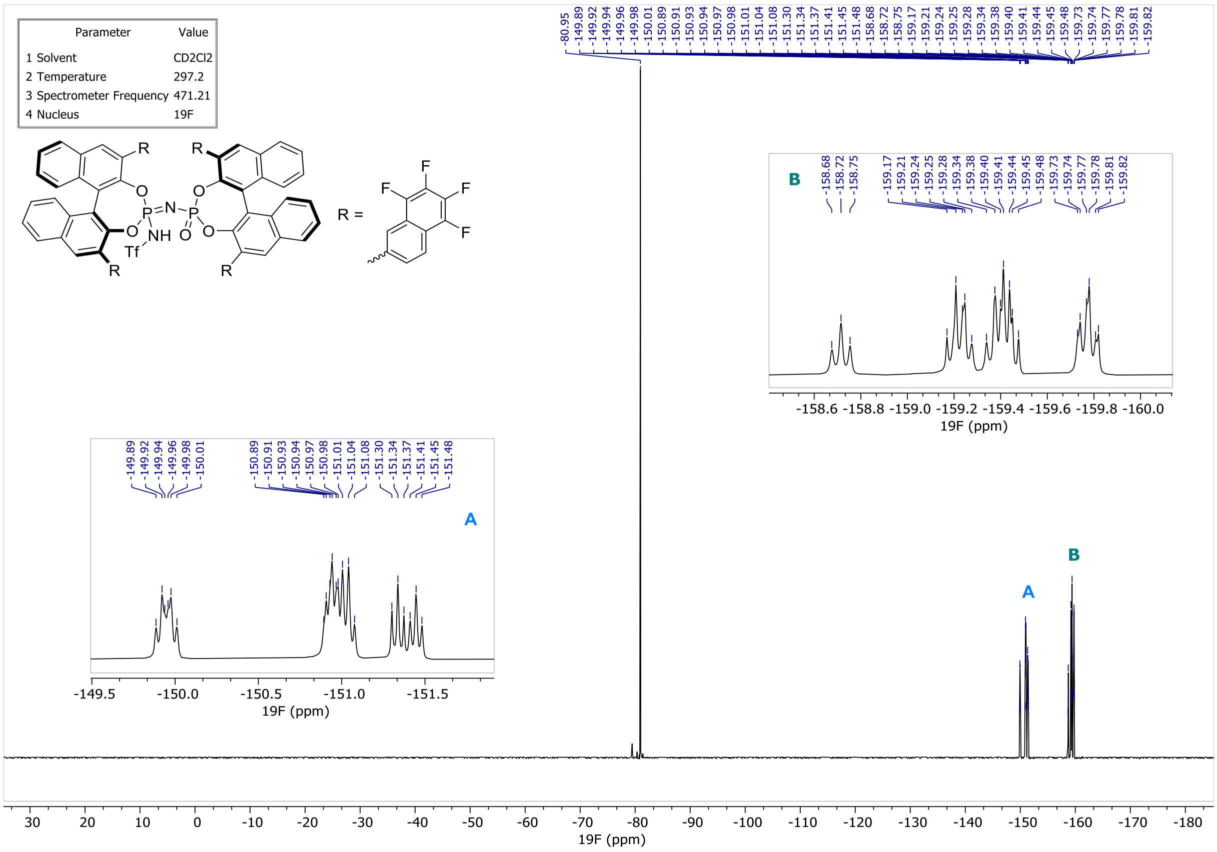
Parameter Value
1 Solvent CD2Cl2
2 Temperature 297.2
3 Spectrometer Frequency 471.21
4 Nucleus 19F
R =
A
B
19F (ppm)

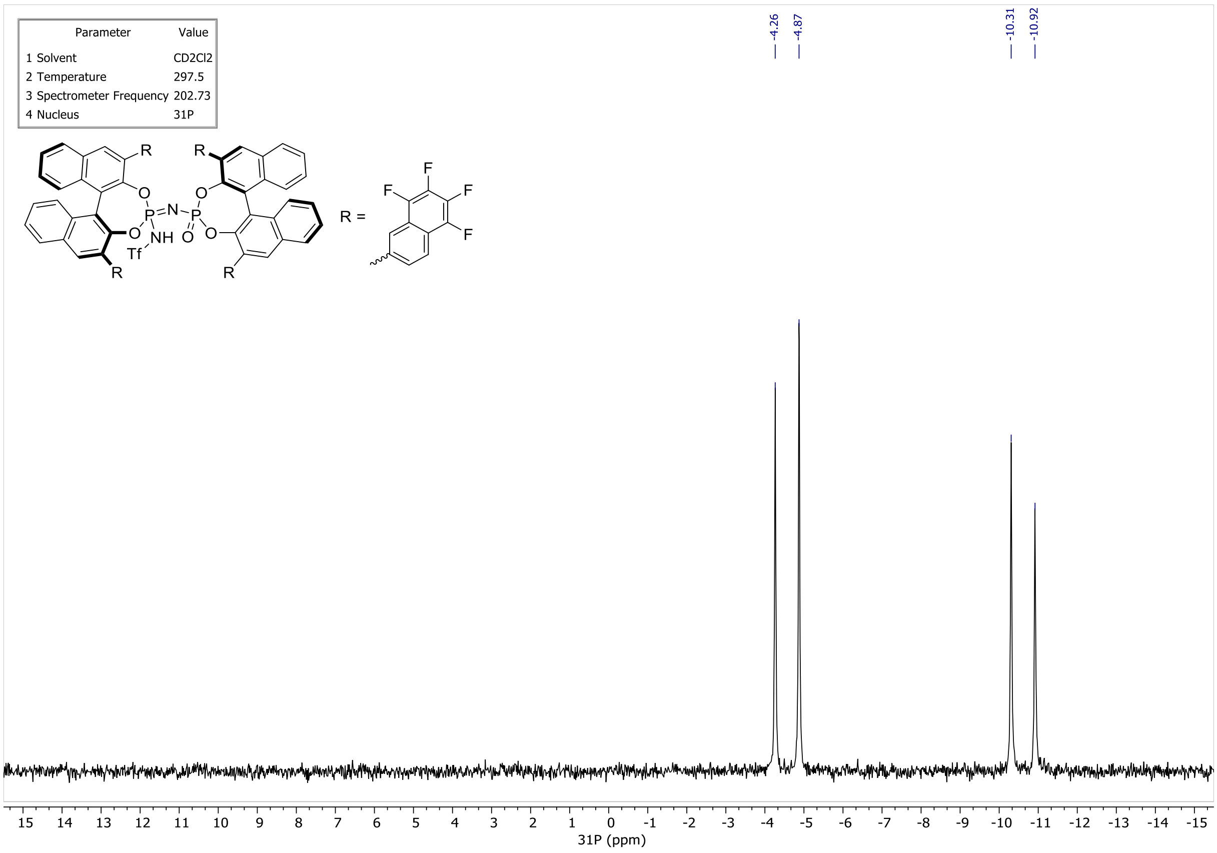
Parameter Value
1 Solvent CD2Cl2
2 Temperature 297.5
3 Spectrometer Frequency 202.73
4 Nucleus 31P
R =
-4.26
-4.87
-10.31
-10.92
31P (ppm)

(*S*,*S*)-*i*IDP **5**

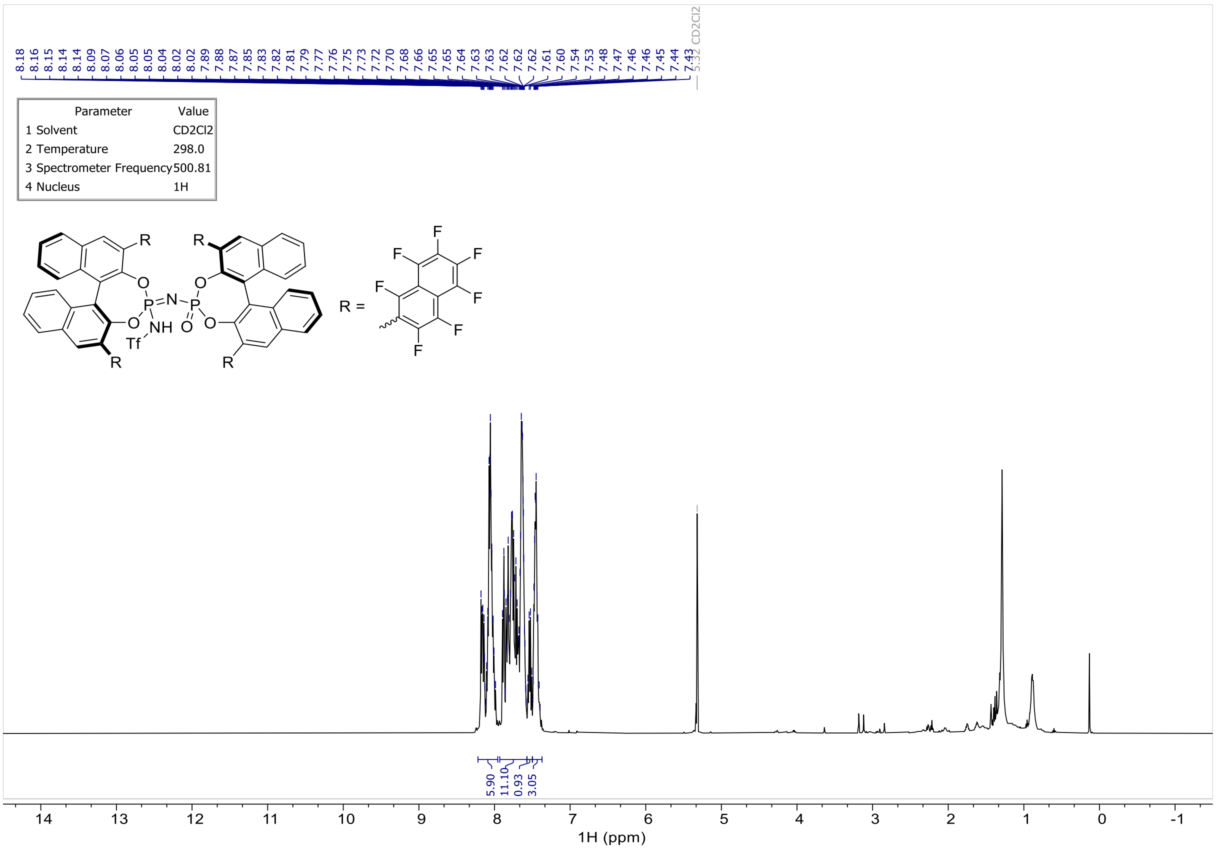

Parameter Value
1 Solvent CD2Cl2
2 Temperature 298.0
3 Spectrometer Frequency 500.81
4 Nucleus 1H
5.32 CD2Cl2
5.90
11.10
0.93
3.05
1H (ppm)

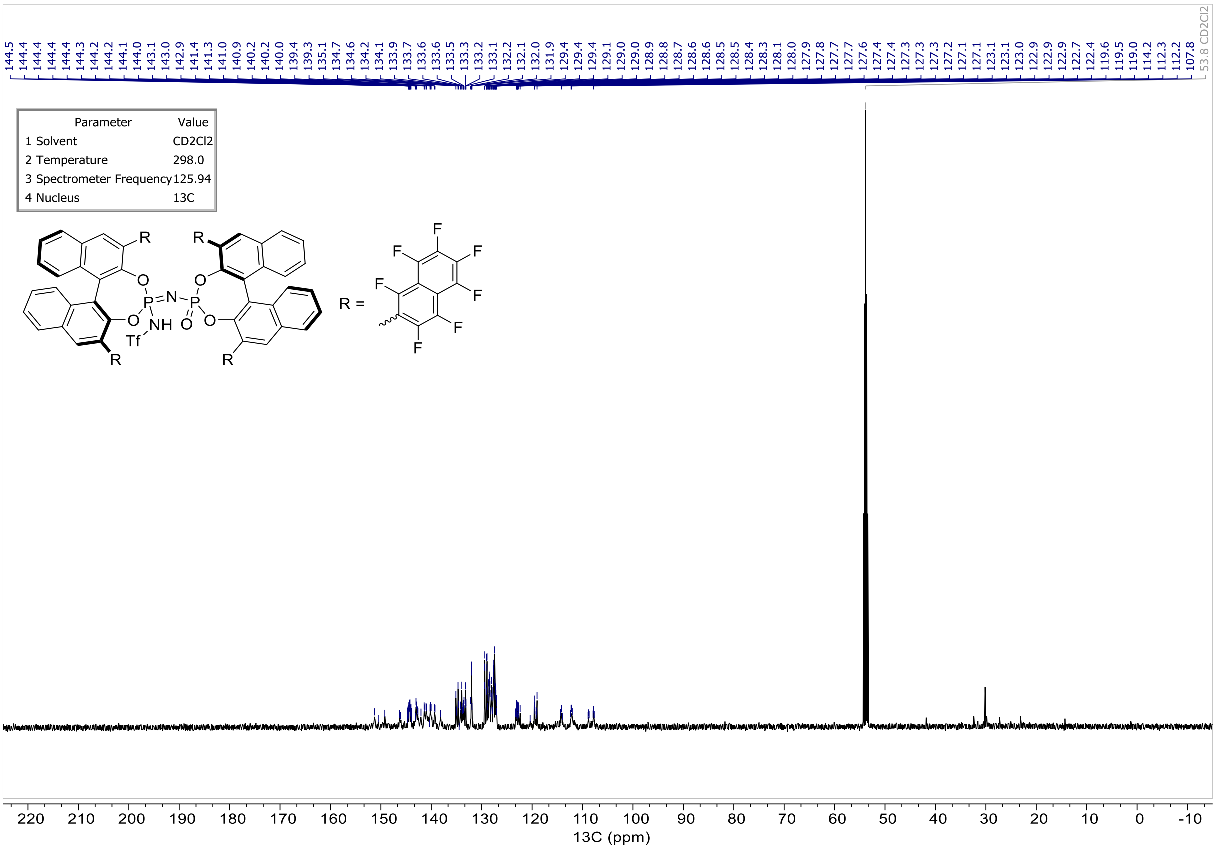

Parameter Value
1 Solvent CD2Cl2
2 Temperature 298.0
3 Spectrometer Frequency 125.94
4 Nucleus 13C
53.8 CD2Cl2
13C (ppm)

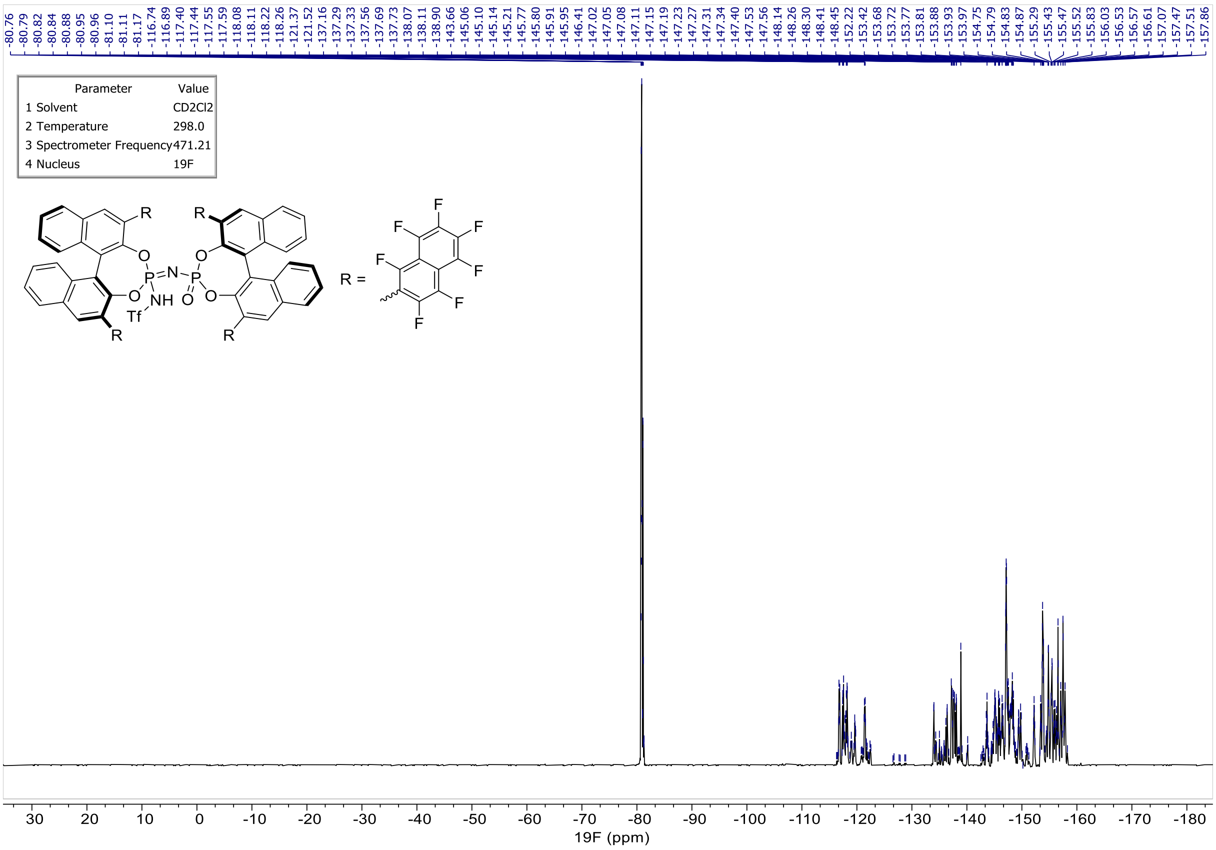
Parameter Value
1 Solvent CD2Cl2
2 Temperature 298.0
3 Spectrometer Frequency 471.21
4 Nucleus 19F
R =
19F (ppm)

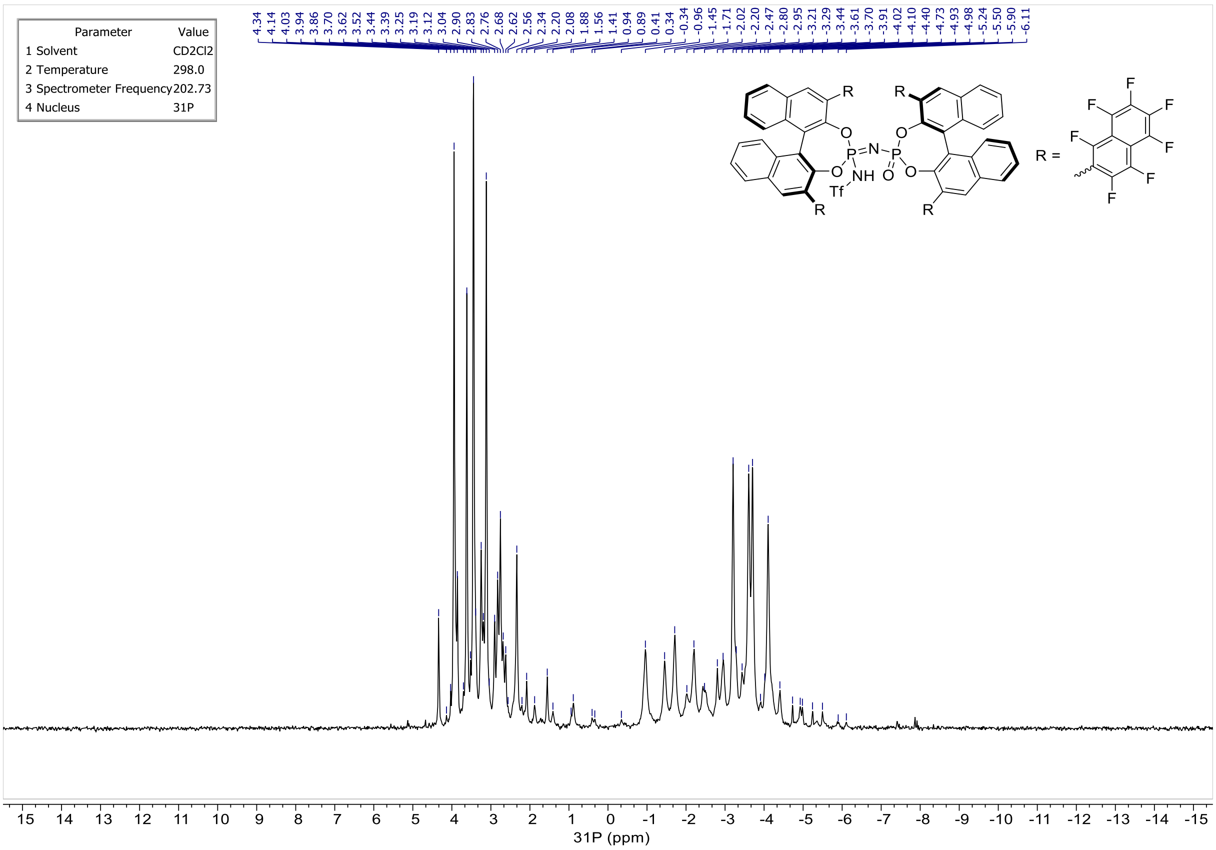
Parameter Value
1 Solvent CD2Cl2
2 Temperature 298.0
3 Spectrometer Frequency 202.73
4 Nucleus 31P
R =
31P (ppm)

(*S*,*S*)-IDPi **6**

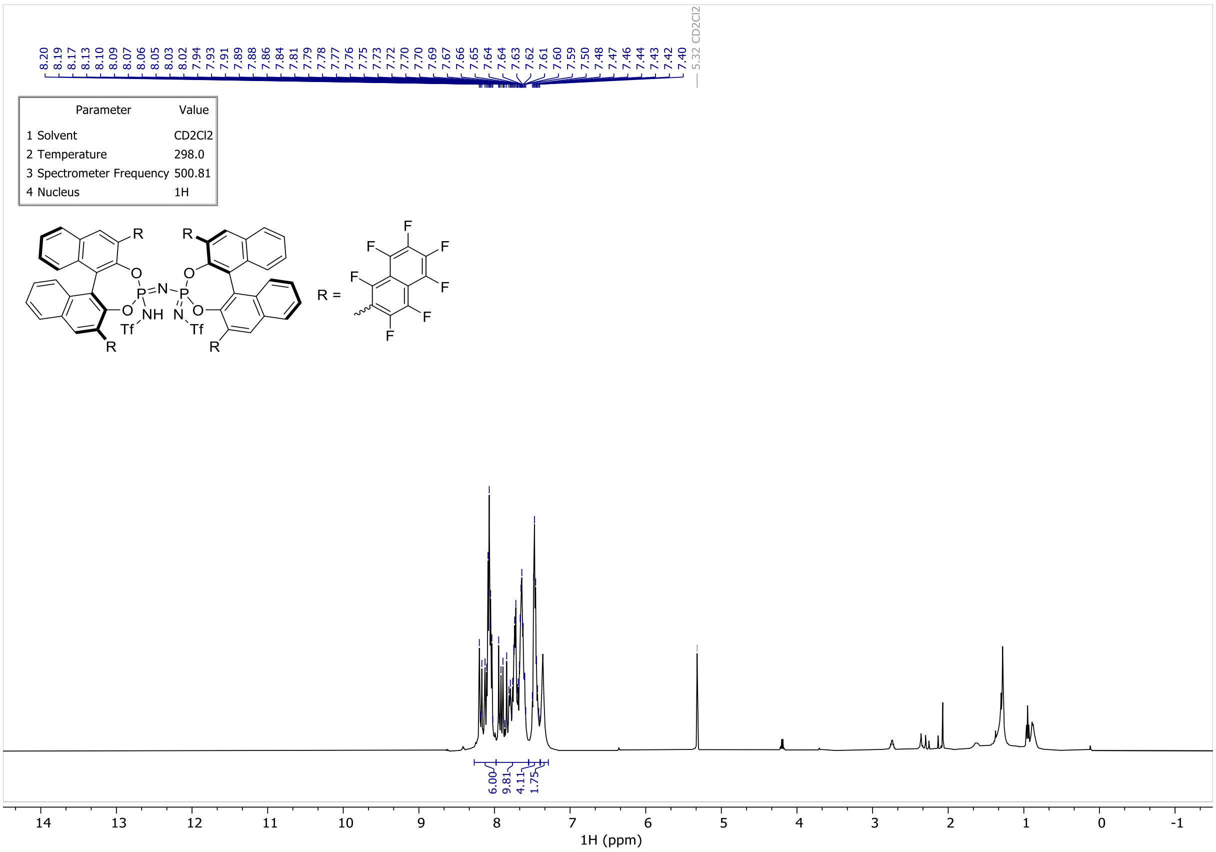
8.20 8.19 8.17 8.13 8.10 8.09 8.07 8.06 8.05 8.03 8.02 7.94 7.93 7.91 7.89 7.88 7.86 7.84 7.81 7.79 7.78 7.77 7.76 7.75 7.73 7.72 7.70 7.70 7.69 7.67 7.66 7.65 7.64 7.64 7.63 7.62 7.61 7.60 7.59 7.50 7.48 7.47 7.46 7.44 7.43 7.42 7.40
5.32 CD2Cl2
Parameter Value
1 Solvent CD2Cl2
2 Temperature 298.0
3 Spectrometer Frequency 500.81
4 Nucleus 1H
R =
6.00 9.81 4.11 1.75
1H (ppm)

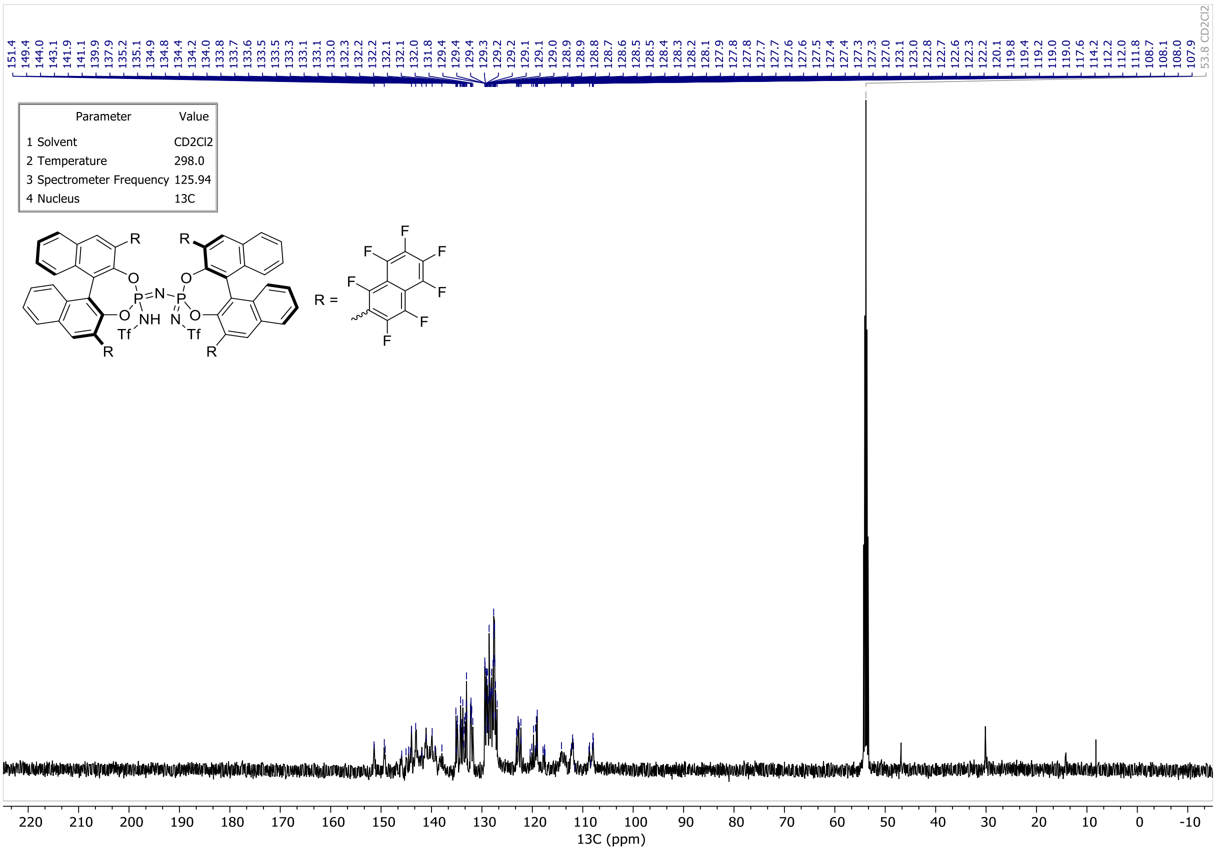
151.4 149.4 144.0 143.1 141.9 141.1 139.9 135.2 135.1 134.9 134.8 134.4 134.2 134.0 133.8 133.7 133.6 133.5 133.3 133.1 133.1 133.0 132.3 132.2 132.2 132.1 132.1 132.0 131.8 129.4 129.4 129.4 129.3 129.2 129.2 129.1 129.1 129.0 128.9 128.9 128.8 128.7 128.6 128.5 128.4 128.3 128.2 128.1 127.9 127.8 127.8 127.7 127.7 127.6 127.6 127.5 127.4 127.4 127.3 127.3 127.0 123.1 123.0 122.8 122.7 122.6 122.3 122.2 120.1 119.8 119.4 119.2 119.0 117.6 114.2 112.2 112.0 111.8 108.7 108.1 108.0 107.9
53.8 CD2Cl2
Parameter Value
1 Solvent CD2Cl2
2 Temperature 298.0
3 Spectrometer Frequency 125.94
4 Nucleus 13C
R =
13C (ppm)

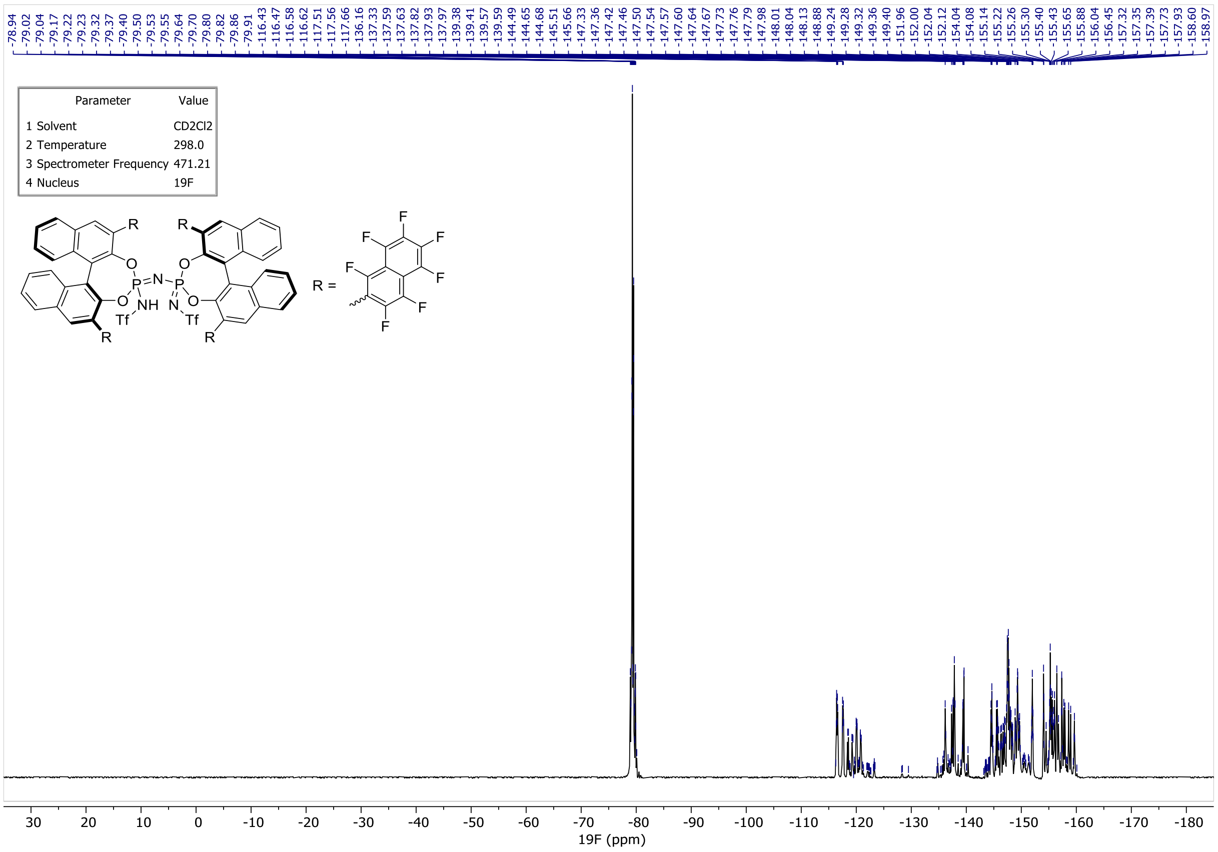

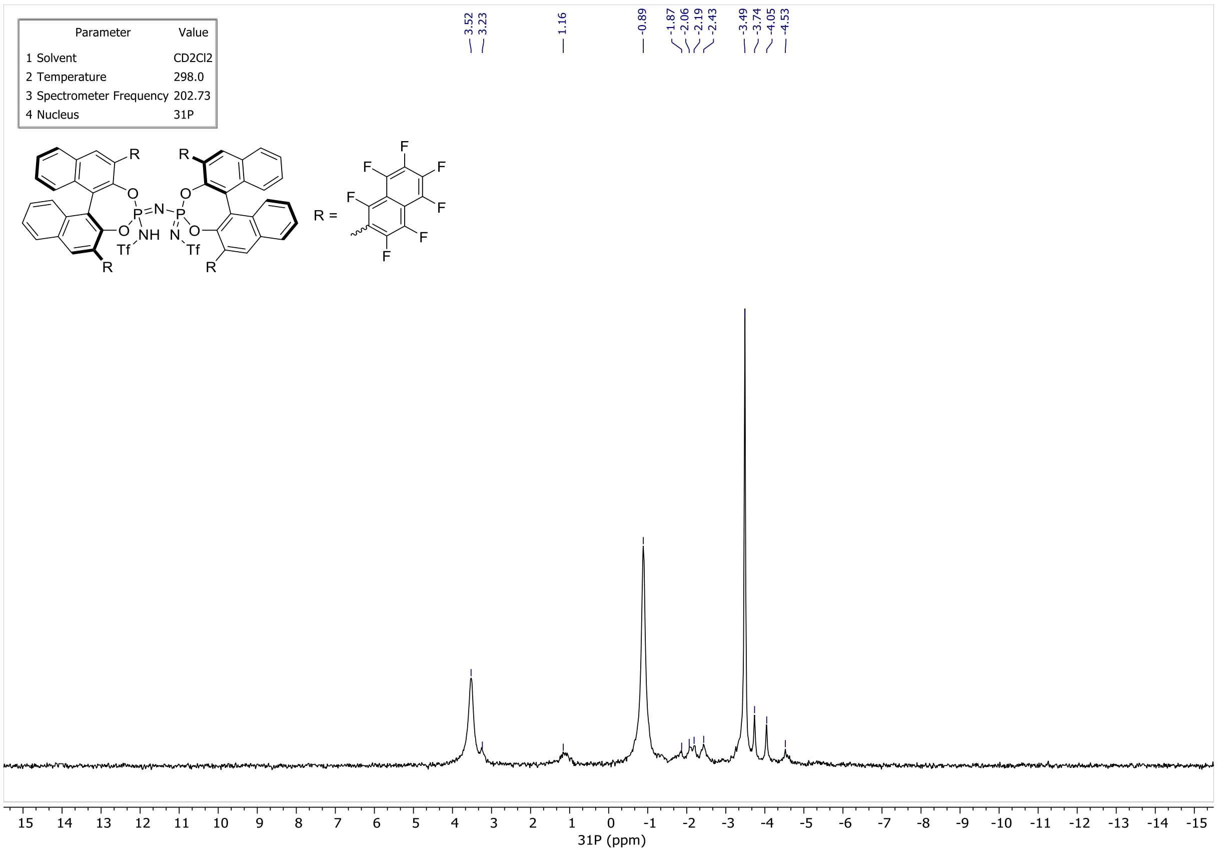

isopiperitenol (*rac*-**2**)

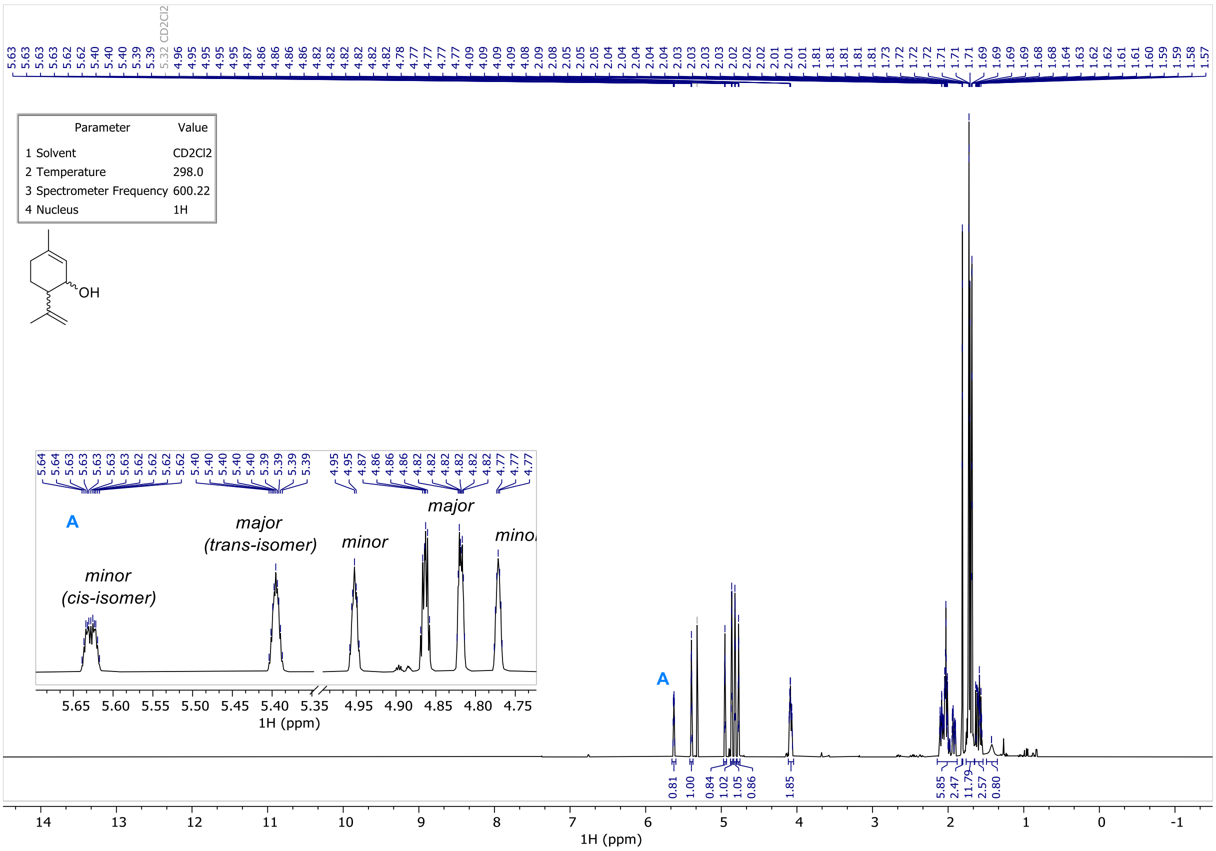

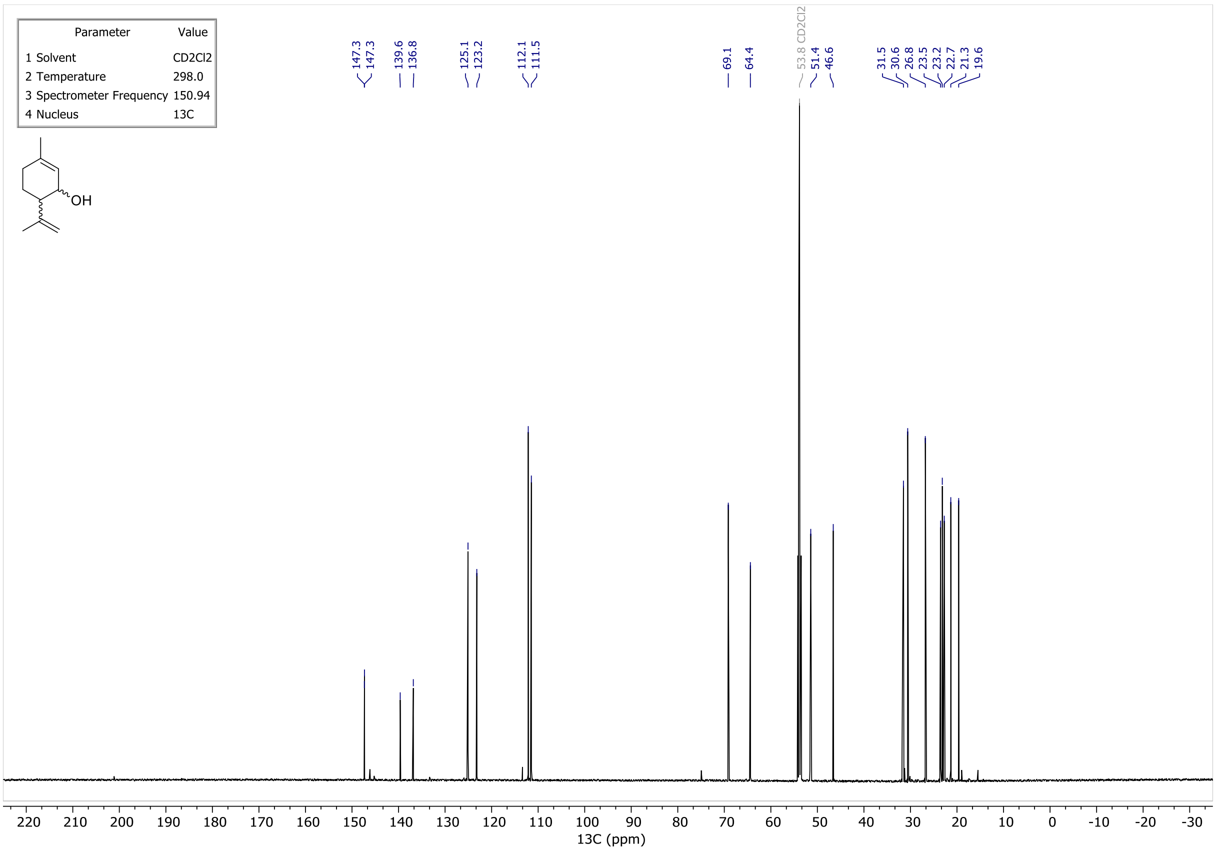

piperitol (**9**)

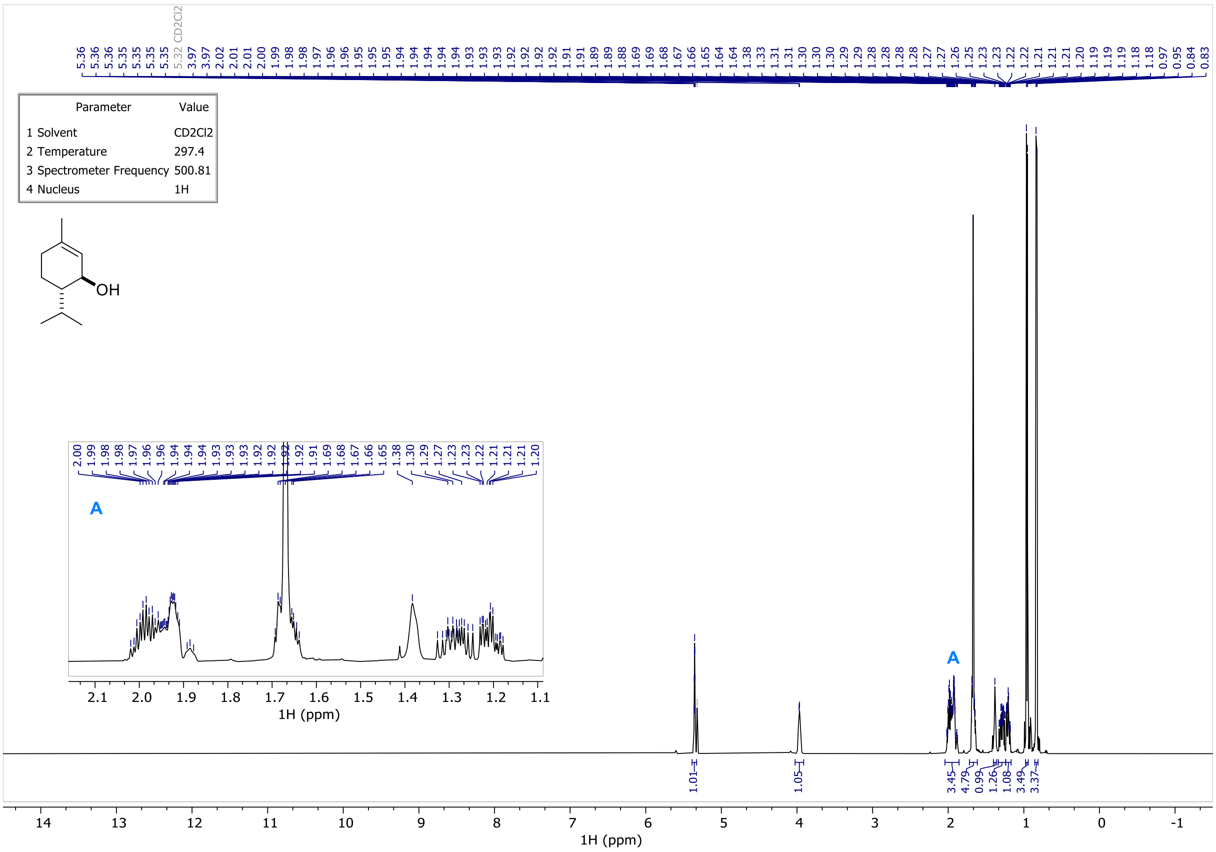

Parameter Value
1 Solvent CD2Cl2
2 Temperature 297.4
3 Spectrometer Frequency 500.81
4 Nucleus 1H
OH
A
1H (ppm)

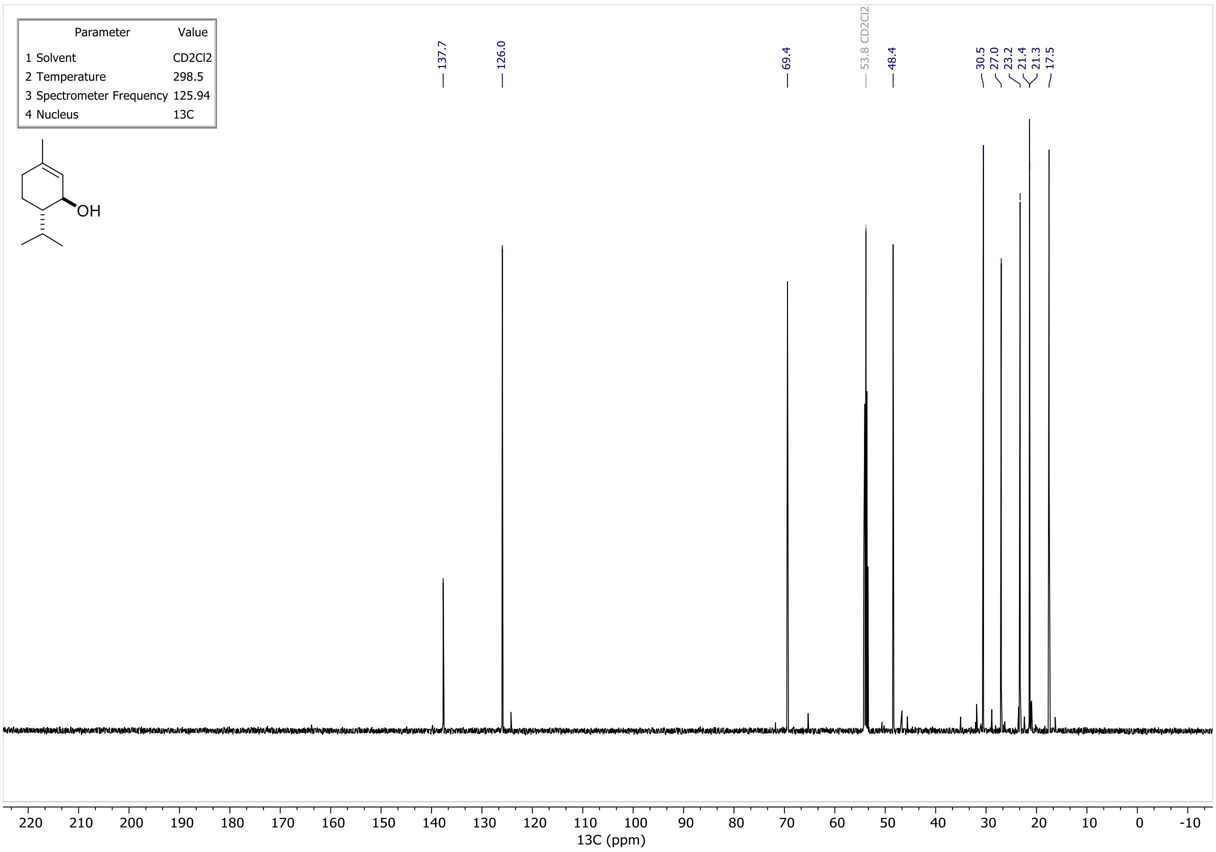

Parameter Value
1 Solvent CD2Cl2
2 Temperature 298.5
3 Spectrometer Frequency 125.94
4 Nucleus 13C
OH
137.7
126.0
69.4
53.8 CD2Cl2
48.4
30.5
27.0
23.2
21.4
21.3
17.5
13C (ppm)

Cannabidiol (**10**)

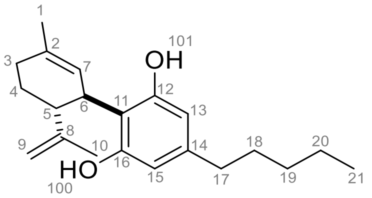

Atom	δ (ppm)	J	COSY	HSQC	HMBC	NOESY
1 C	24.068			1	7	
H3	1.774		6, 7	1	2, 3, 7	7
2 C	140.685				1, 3', 3"	
3 C	30.207			3', 3"	1, 4', 4", 5, 7	
H'	2.205		3', 4', 4"	3	2, 7	3", 4', 4"
H"	2.078		4', 4"	3	2, 7	3', 4', 4"
4 C	28.089			4', 4"	5	
H'	1.785	11.00(5)	3', 3", 5	4	3, 5, 6	3', 3", 5, 6, 9"
H"	1.785	3.60(5)	3', 3", 5	4	3, 5, 6	3', 3", 5, 6, 9"
5 C	46.145			5	4', 4", 7, 9', 9", 10	
H	2.356	11.00(6), 11.00(4'), 3.60(4")	4', 4", 6	5	3, 4, 6, 8, 9, 10, 11	4', 4", 9", 10, 100, 101
6 C	36.676			6	4', 4", 5, 7	
H	3.842	11.00(5)	1, 5, 7	6		4', 4", 7, 9", 10, 100, 101
7 C	123.677			7	1, 3', 3"	
H	5.556		1, 6	7	1, 3, 5, 6	1, 6, 100, 101
8 C	149.503				5, 10	
9 C	111.030			9', 9"	5, 10	
H'	4.623		9", 10	9	5, 10	9", 10
H"	4.495		9', 10	9	5, 10	4', 4", 5, 6, 9'
10 C	20.278			10	5, 9', 9"	
H3	1.659		9', 9"	10	5, 8, 9	5, 6, 9'
11 C	113.499				5, 13, 15, 101	

Atom	δ (ppm)	J	COSY	HSQC	HMBC	NOESY
12 C	153.781				13	
13 C	107.765			13	15, 17	
H	6.153	1.50(15)	15	13	11, 12, 14, 15, 17	17, 18
14 C	143.077				13, 17, 18	
15 C	109.454			15	13, 17, 101	
H	6.281	1.50(13)	13	15	11, 13, 16, 17	17, 18
16 C	155.723				15, 101	
17 C	35.528			17	13, 15, 18, 19	
H2	2.405		18	17	13, 14, 15, 18, 19	13, 15, 18, 19
18 C	31.025			18	17, 19, 20	
H2	1.505		17, 19	18	14, 17, 19, 20	13, 15, 17
19 C	31.547			19	17, 18, 20, 21'	
H2	1.226		18	19	17, 18, 20	17
20 C	22.721			20	18, 19, 21'	
H2	1.264		21'	20	18, 19, 21	
21 C	14.344			21', 21", 21'"	20	
H3	0.842	7.10(21"), 7.10(21'")	20	21	19, 20	
100 O						
H	5.115					5, 6, 7
101 O						
H	6.153				11, 15, 16	5, 6, 7

Remarks:

Due to broadened peaks at room temperature (caused by two dynamic processes: OH-exchange with water and the hindered rotation around the C6-C11 bond) the sample was fully characterized at –40 °C (233 K). The coupling constant between H6 and H5 is large, which support their *trans*-configuration in the 6-membered ring. The reported data at room temperature is in good agreement with literature[22]. However, the peaks of C16 and C12 are missing in the ^{13}C (likely due to the broadening). The ^{1}H NMR also shows the significant broadening of H15, H13, OH-100 and OH-101.

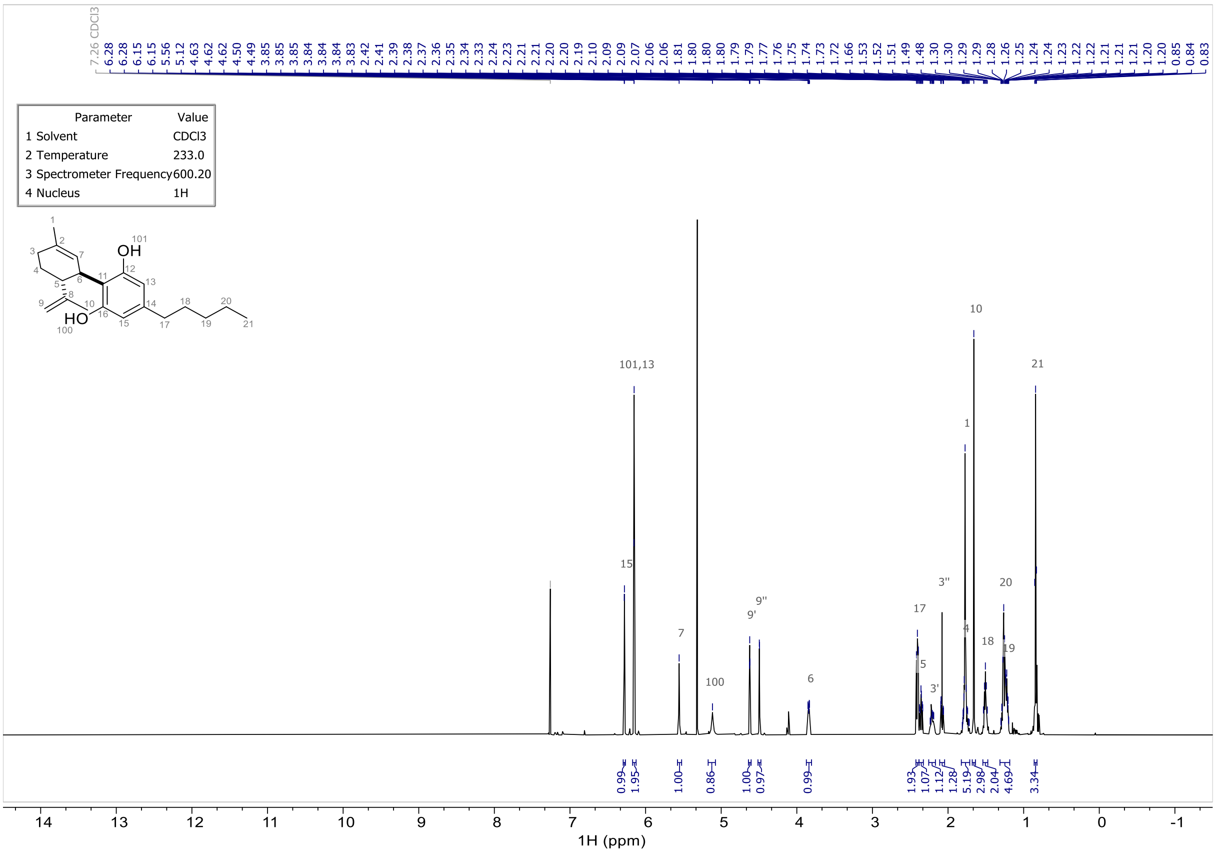
Parameter Value
1 Solvent CDCl3
2 Temperature 233.0
3 Spectrometer Frequency 600.20
4 Nucleus 1H
1H (ppm)

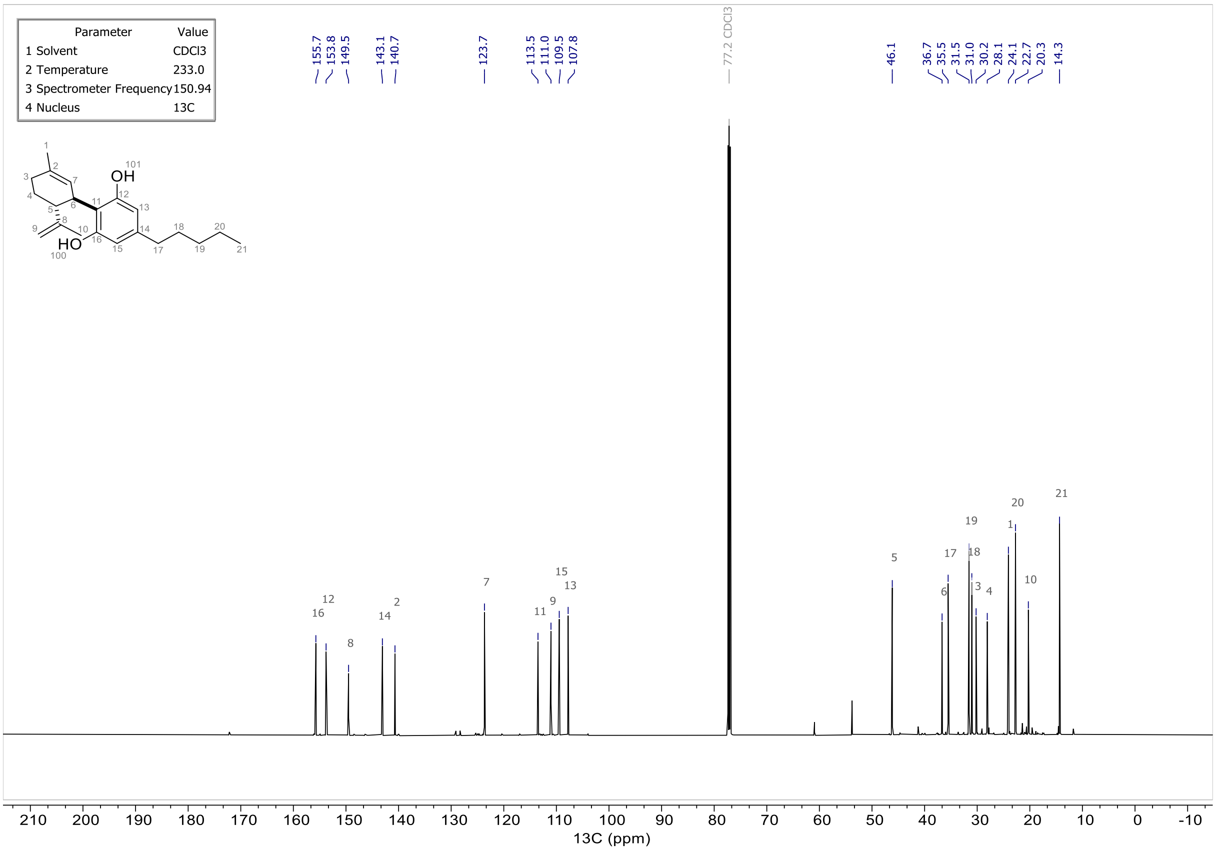
Parameter Value
1 Solvent CDCl3
2 Temperature 233.0
3 Spectrometer Frequency 150.94
4 Nucleus 13C
13C (ppm)

1H,13C-HSQC-EDITED

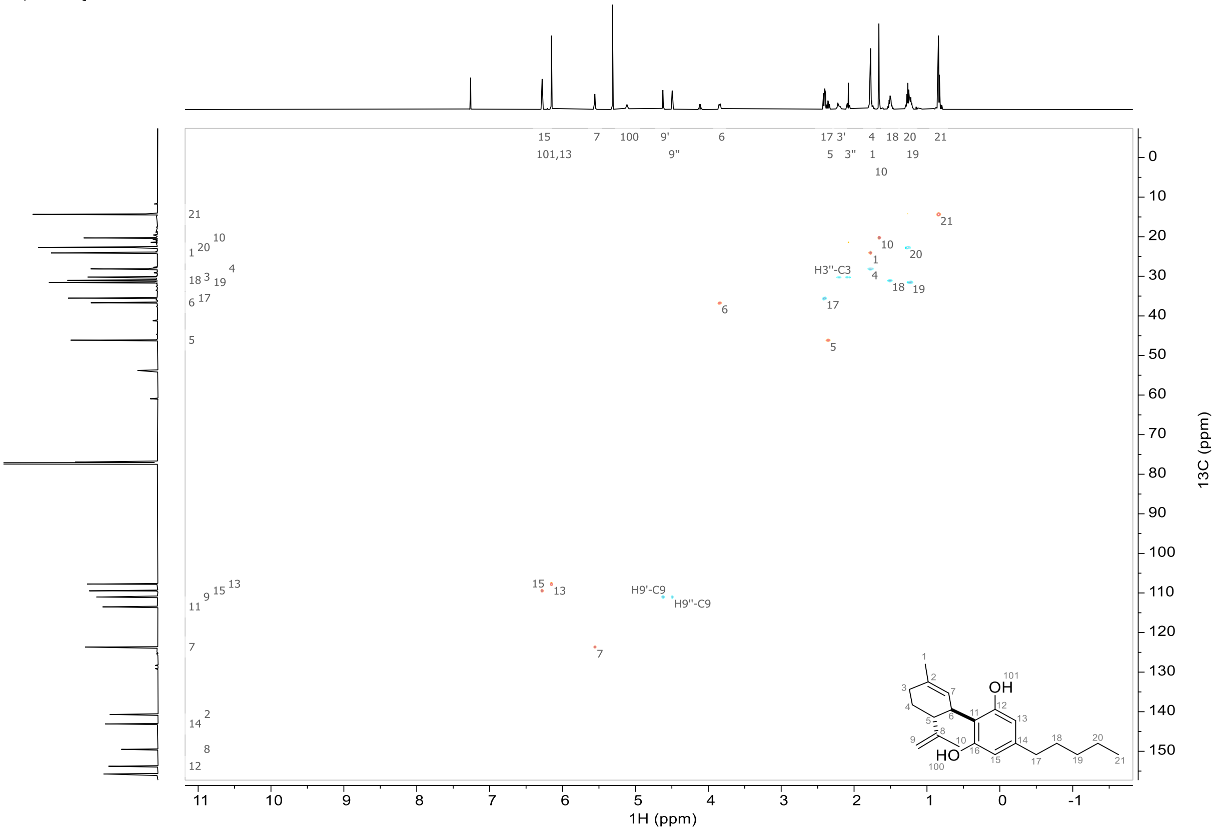

1H,13C-HMBC

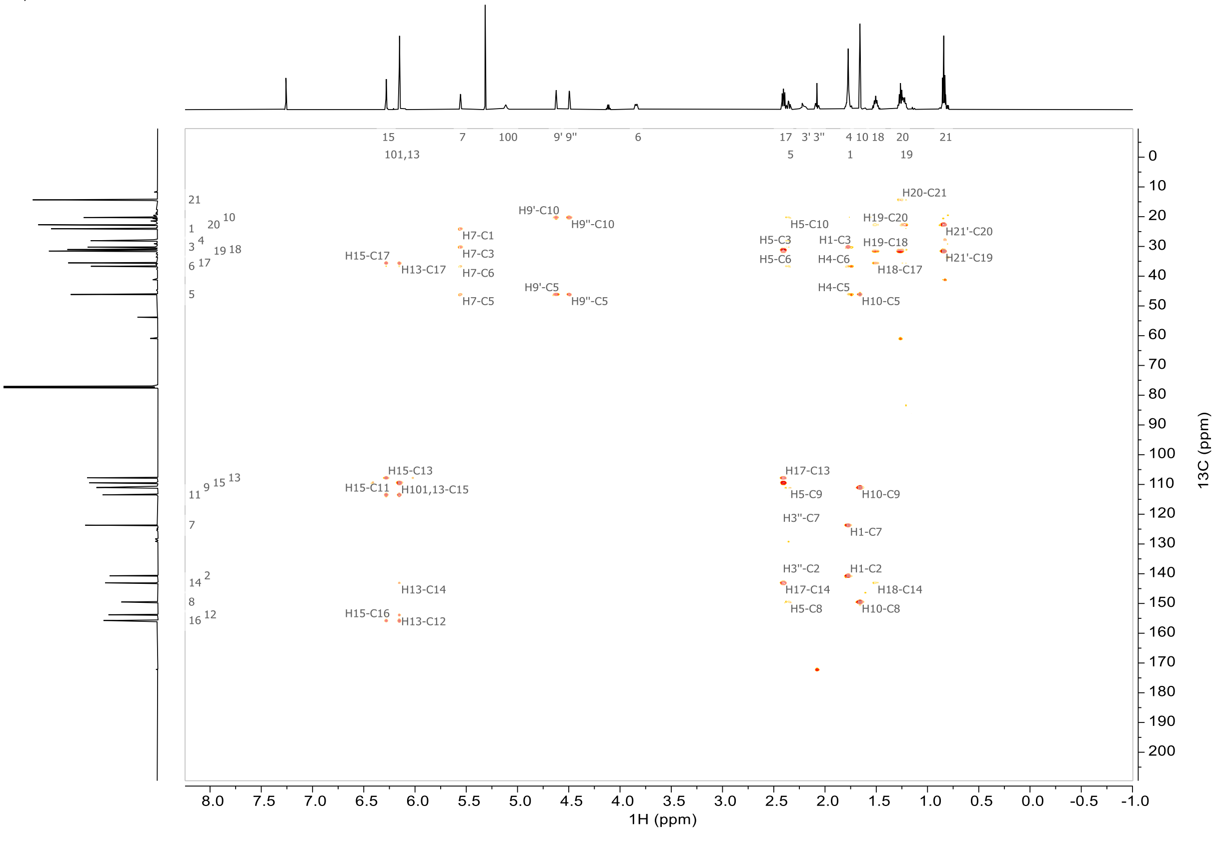

1H,1H-COSY

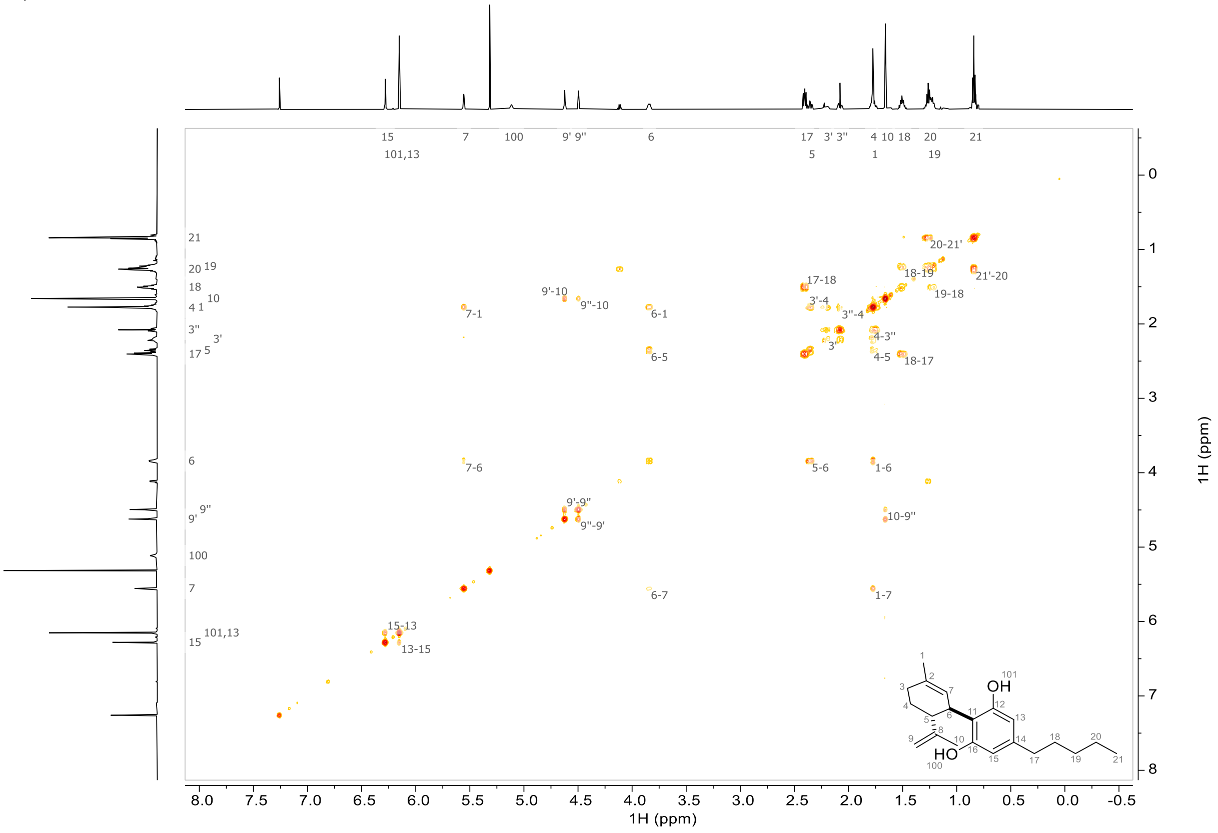

1H,1H-NOESY

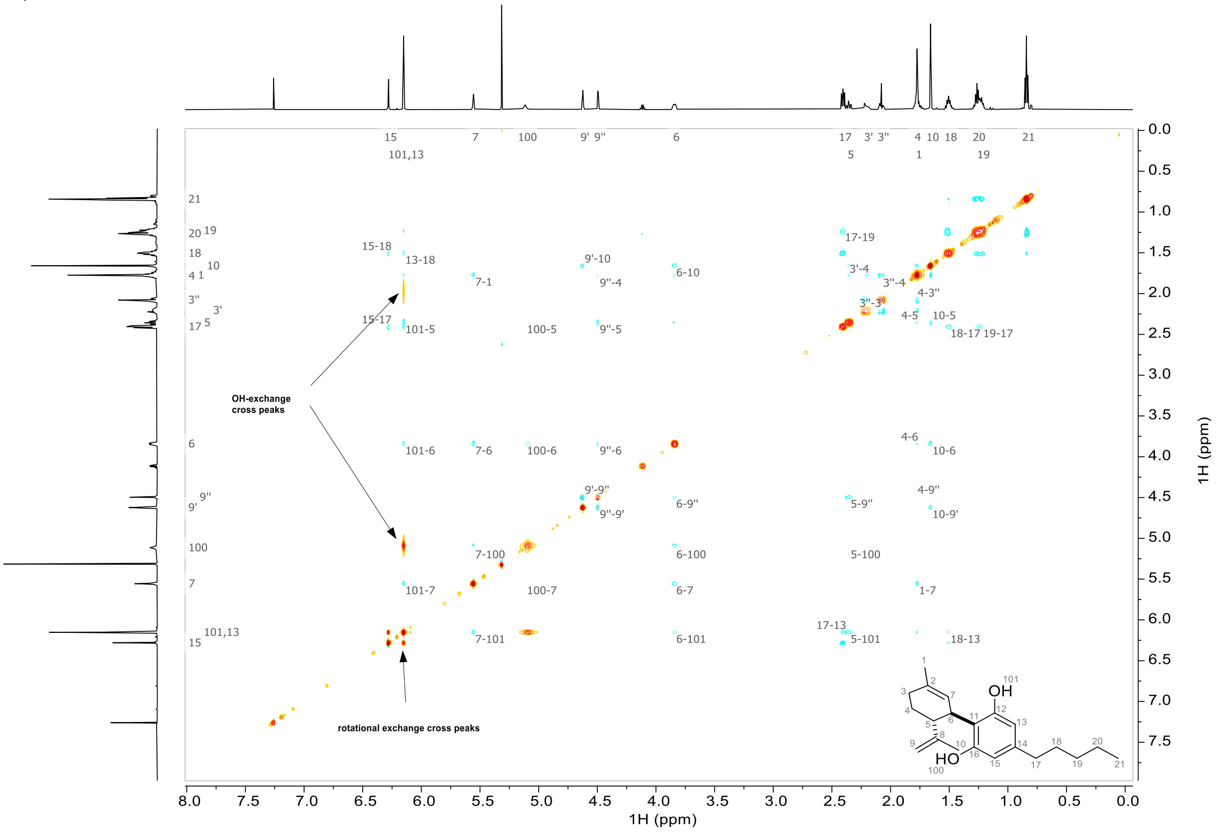

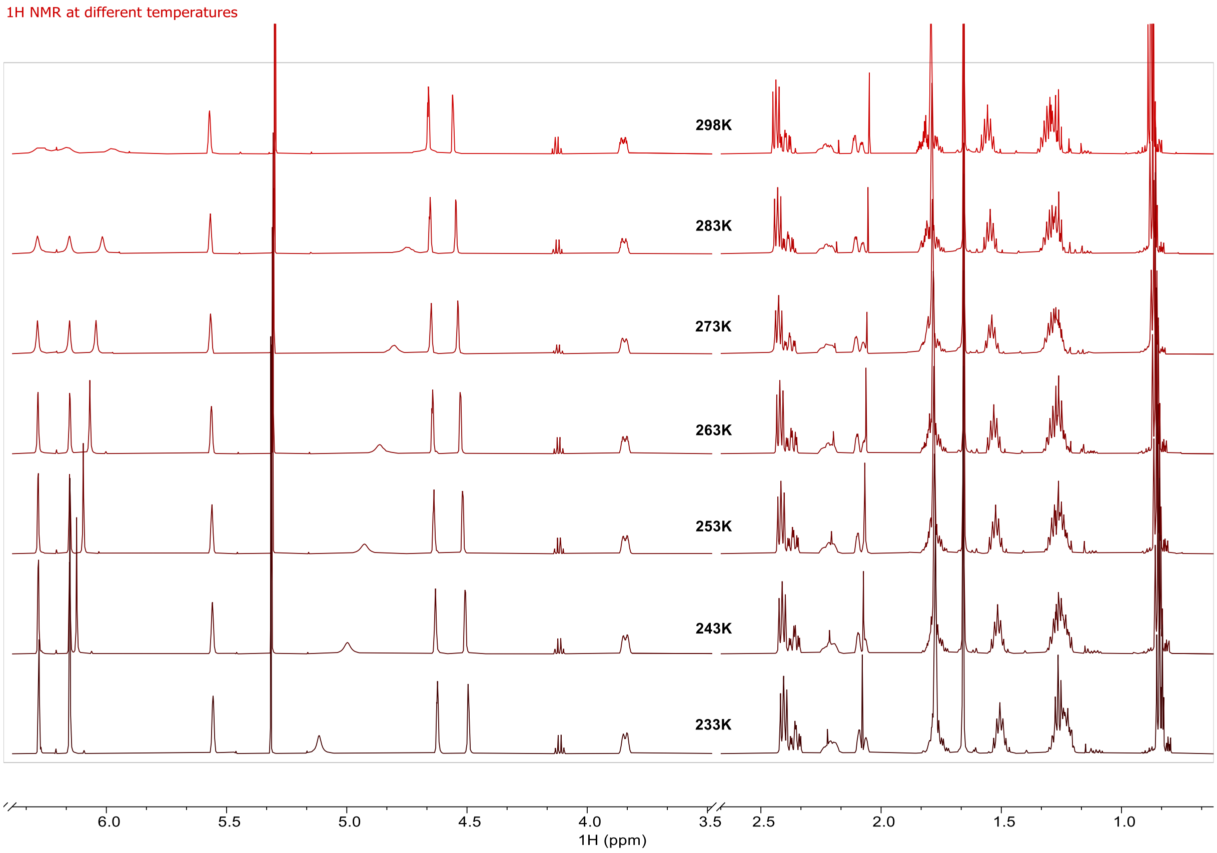
1H NMR at different temperatures
298K
283K
273K
263K
253K
243K
233K
1H (ppm)

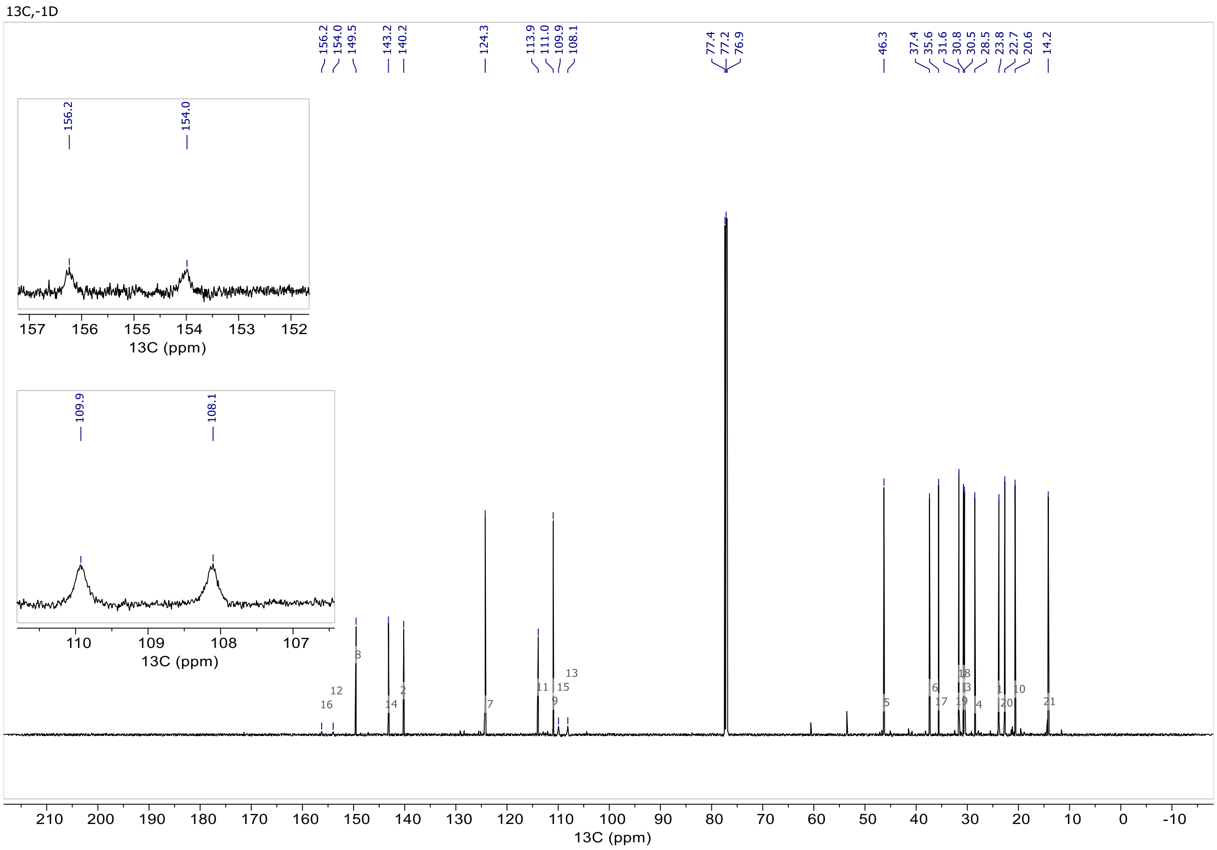
13C,-1D
156.2
154.0
149.5
143.2
140.2
124.3
113.9
111.0
109.9
108.1
77.4
77.2
76.9
46.3
37.4
35.6
31.6
30.8
30.5
28.5
23.8
22.7
20.6
14.2
13C (ppm)

Methyl (1'R,2'R)-2,6-dihydroxy-5'-methyl-4-pentyl-2'-(prop-1-en-2-yl)-1',2',3',4'-tetrahydro-[1,1'-biphenyl]-3-carboxylate (**11**)

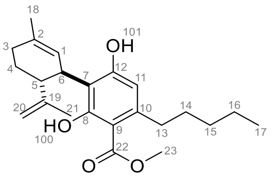

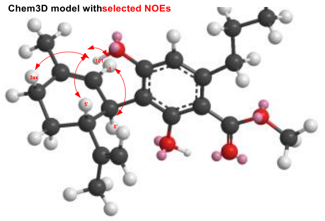

Atom	J	δ (ppm)	COSY	HSQC	HMBC	NOESY
1 C		124.187		1	3ax, 3eq, 6', 18	
H		5.556	3ax, 3eq, 6', 18	1	3, 5, 6, 7, 18	6', 18, 101
2 C		140.368			3ax, 3eq, 4, 18	
3 C		30.369		3ax, 3eq	1, 4, 5', 18	
Hax		2.223	1, 3eq, 4, 6'	3	1, 2, 4	4, 5', 101
Heq		2.099	1, 3ax, 4, 6'	3	1, 2, 4, 5, 18	4
4 C		27.956		4	3ax, 3eq, 5'	
H2		1.797	3ax, 3eq, 5'	4	2, 3, 5, 6, 19	3ax, 3eq, 5', 6', 20cis
5 C		46.804		5'	1, 3eq, 4, 20cis, 20trans, 21	
5' H	9.10(6')	2.384	4, 6'	5	3, 4, 6, 7, 19, 20, 21	3ax, 4, 20cis, 101
6 C		35.518		6'	1, 4, 5'	
6' H	9.10(5')	4.098	1, 3ax, 3eq, 5', 18	6	1, 7, 12	1, 4, 20cis, 21, 100
7 C		114.509			1, 5', 6', 11, 100, 101	
8 C		163.249			100	
9 C		104.038			11, 13', 13'', 100	
10 C		146.097			13', 13'', 14	
11 C		111.633		11	13', 13'', 101	
H		6.212		11	7, 9, 12, 13	13', 13'', 14, 15, 101
12 C		160.056			6', 11, 101	
13 C		36.956		13', 13''	11, 14, 15	
H'		2.838	14	13	9, 10, 11, 14, 15	11, 14, 15, 23
H''		2.738	14	13	9, 10, 11, 14, 15	11, 14, 15, 23
14 C		31.542		14	13', 13'', 15, 16	
H2		1.511	13', 13'', 15	14	10, 13, 15, 16	11, 13', 13'', 23

Atom	J	δ (ppm)	COSY	HSQC	HMBC	NOESY
15 C		32.228		15	13', 13'', 14, 16, 17	
H2		1.324	14	15	13, 14, 16, 17	11, 13', 13'', 23
16 C		22.681		16	14, 15, 17	
H2		1.317	17	16	14, 15, 17	
17 C		14.233		17	15, 16	
H3		0.897	16	17	15, 16	
18 C		23.851		18	1, 3eq	
H3		1.787	1, 6'	18	1, 2, 3	1, 101
19 C		147.372			4, 5', 20cis, 20trans, 21	
20 C		111.380		20cis, 20trans	5', 21	
Hcis		4.381	20trans, 21	20	5, 19, 21	4, 5', 6'
Htrans		4.519	20cis, 21	20	5, 19, 21	21
21 C		18.952		21	5', 20cis, 20trans	
H3		1.709	20cis, 20trans	21	5, 19, 20	6', 20trans, 100
22 C		172.771			23	
23 C		51.875		23		
H3		3.898		23	22	13', 13'', 14, 15, 100
100 O						
H		11.985			7, 8, 9	6', 21, 23
101 O						
H		6.500			7, 11, 12	1, 3ax, 5', 11, 18

Remarks:

The large $^3J_{H6'H5'}$ value (~9 Hz) supports their *trans*-configuration. OH-101 is exchanging with water in the sample, while OH-100 is not. The later one is also quite deshielding, which could be explained by H-bonding to the ester moiety. The molecule has an axial chirality along the C6-C7 bond, which does not seem to rotate fast based on the NOEs observed.

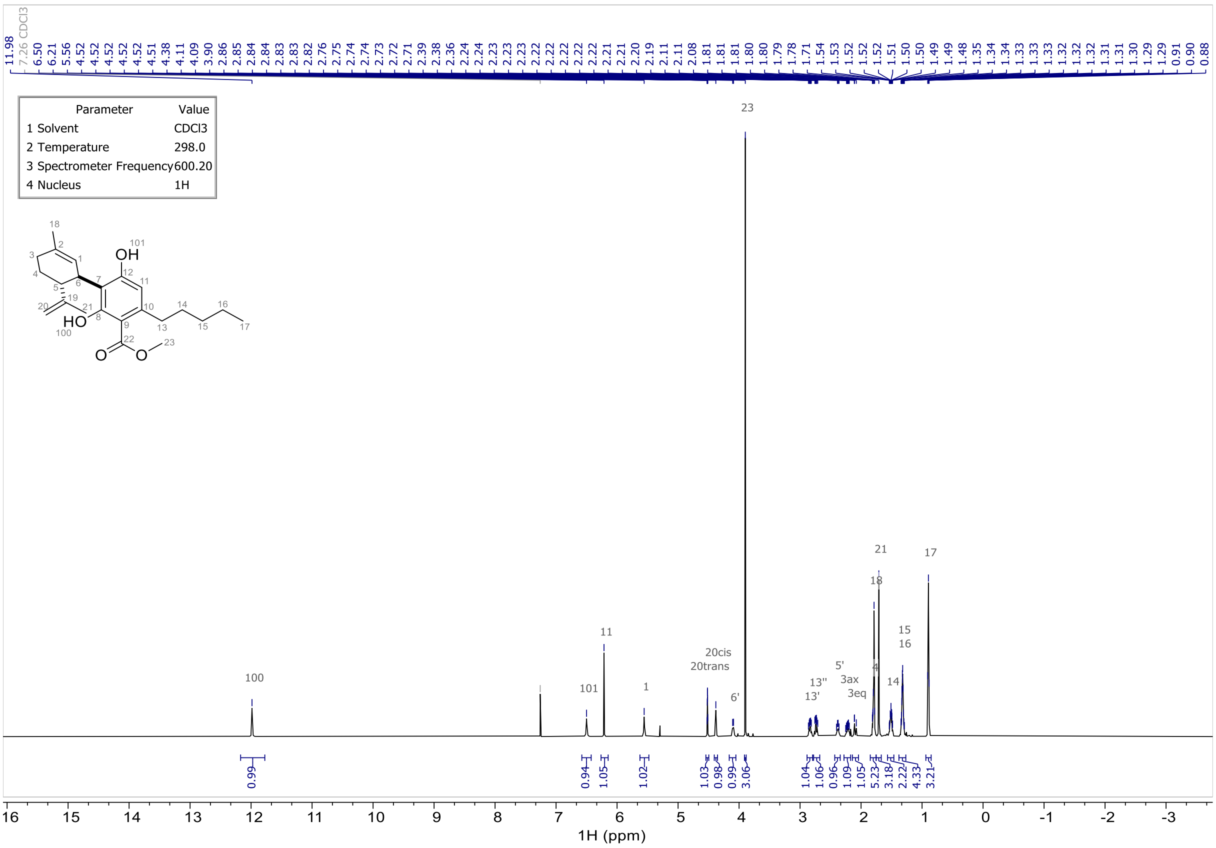
11.98
7.26 CDCl3
6.50
6.21
5.56
4.52
4.52
4.52
4.52
4.52
4.51
4.38
4.11
4.09
3.90
2.86
2.85
2.84
2.84
2.83
2.83
2.82
2.76
2.75
2.74
2.74
2.73
2.72
2.71
2.39
2.38
2.36
2.24
2.24
2.23
2.23
2.23
2.22
2.22
2.22
2.22
2.22
2.21
2.21
2.20
2.19
2.11
2.11
2.08
1.81
1.81
1.81
1.80
1.80
1.79
1.78
1.71
1.54
1.53
1.52
1.52
1.52
1.51
1.50
1.50
1.49
1.48
1.35
1.34
1.34
1.33
1.33
1.33
1.32
1.32
1.32
1.31
1.30
1.29
1.29
0.91
0.90
0.88
Parameter Value
1 Solvent CDCl3
2 Temperature 298.0
3 Spectrometer Frequency 600.20
4 Nucleus 1H
OH
HO
O
O
23
100
101
11
1
20cis
20trans
6'
13''
13'
5'
3ax
3eq
4
18
21
14
15
16
17
0.99
0.94
1.05
1.02
1.03
0.98
0.99
3.06
1.04
1.06
0.96
1.09
1.05
5.23
3.18
2.22
4.33
3.21
16 15 14 13 12 11 10 9 8 7 6 5 4 3 2 1 0 -1 -2 -3
1H (ppm)

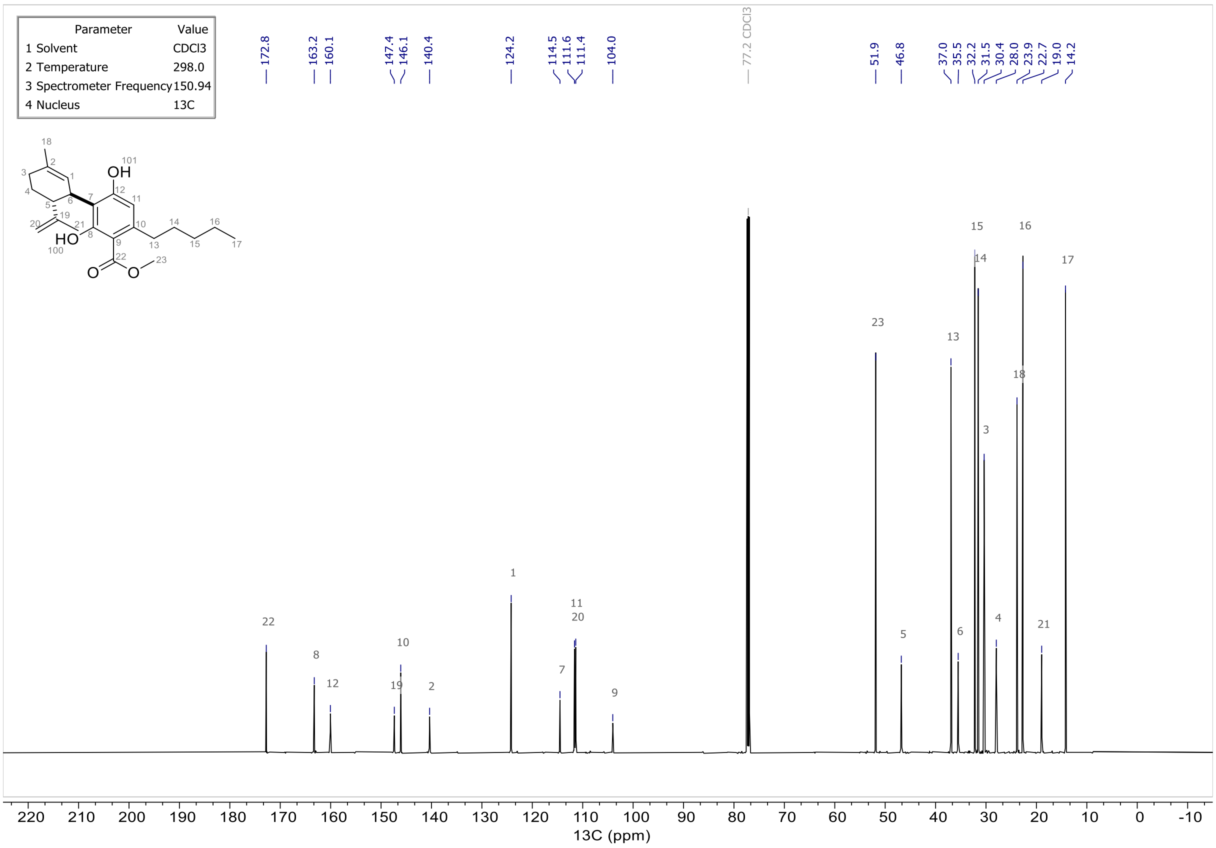
Parameter Value
1 Solvent CDCl3
2 Temperature 298.0
3 Spectrometer Frequency 150.94
4 Nucleus 13C
172.8
163.2
160.1
147.4
146.1
140.4
124.2
114.5
111.6
111.4
104.0
77.2 CDCl3
51.9
46.8
37.0
35.5
32.2
31.5
30.4
28.0
23.9
22.7
19.0
14.2
OH
HO
O
O
22
8
12
10
19
2
1
7
11
20
9
23
5
13
6
15
14
16
18
3
4
21
17
220 210 200 190 180 170 160 150 140 130 120 110 100 90 80 70 60 50 40 30 20 10 0 -10
13C (ppm)

1H{13C},HSQC-EDITED, 600.20 MHz,CDCl3,298.0K, pulse sequence: hsqcedetgpsisp2.3

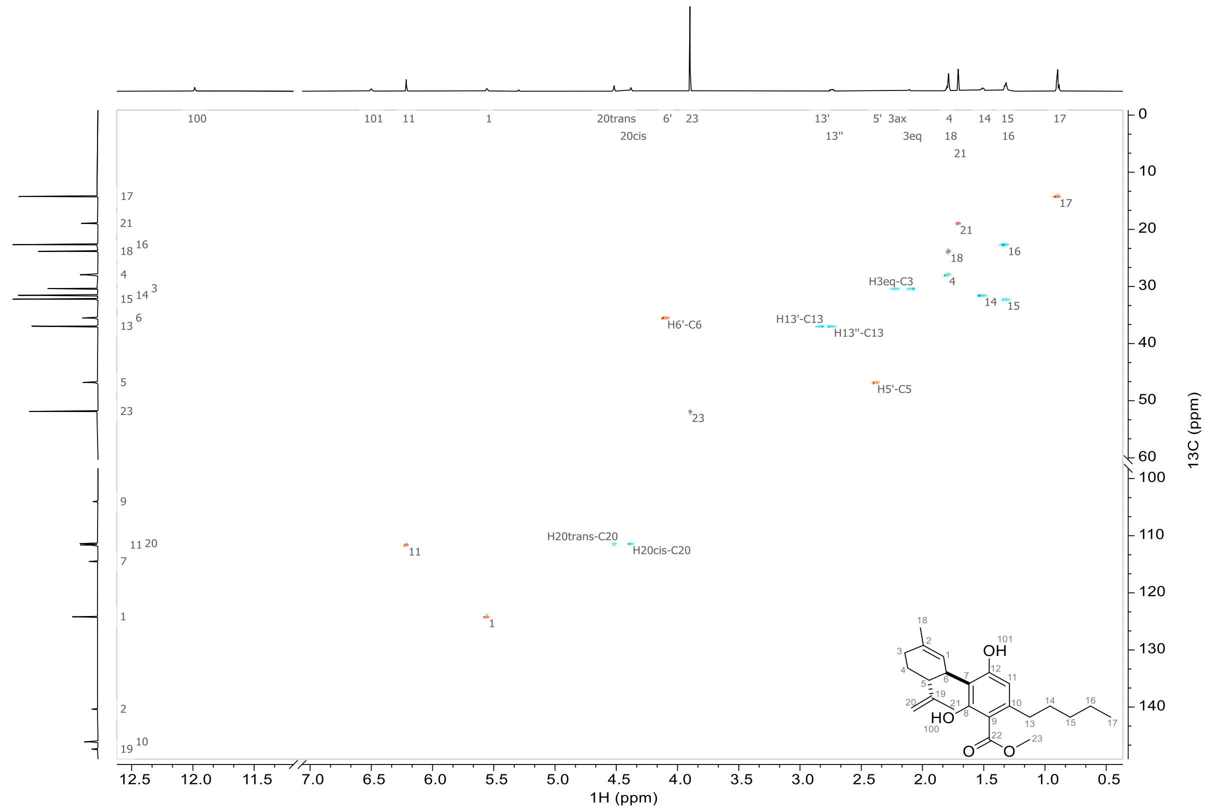

1H{13C},HMBC, 600.20 MHz,CDCl3,298.0K, pulse sequence: hmbcetgpl3nd

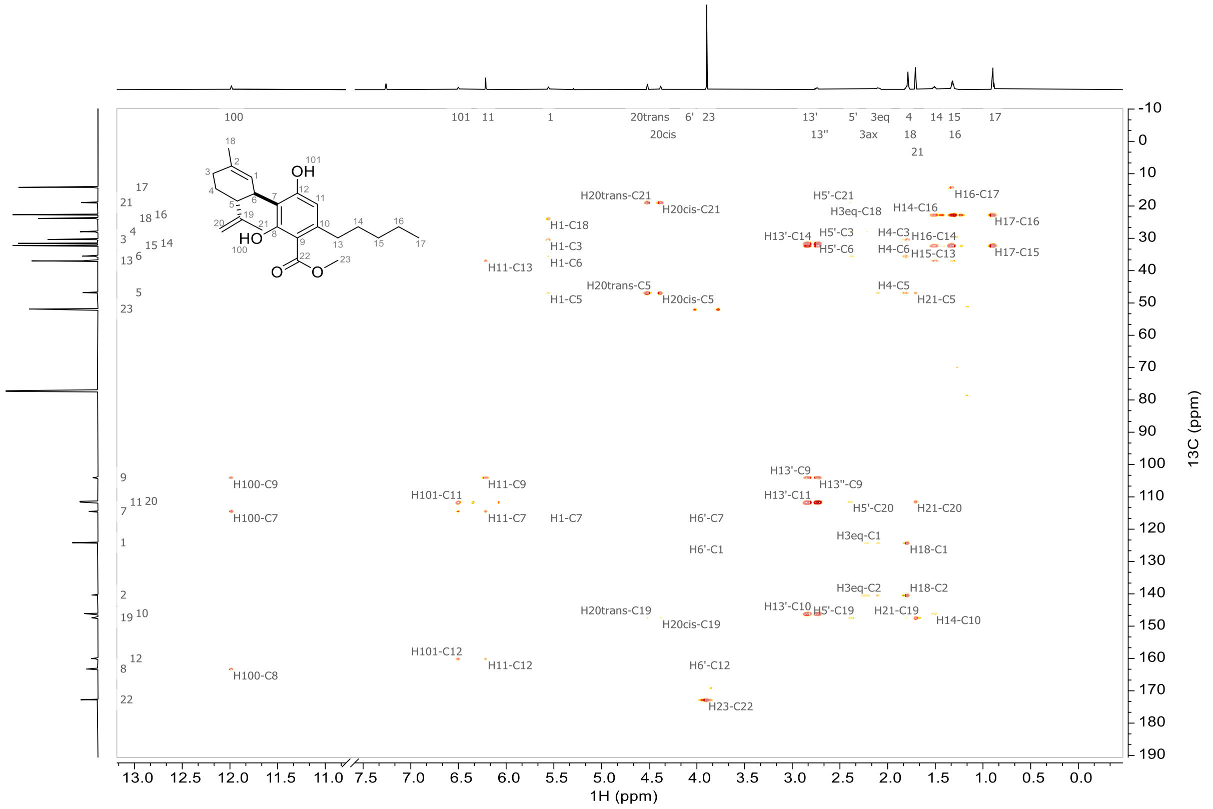

1H{off},COSY, 600.20 MHz,CDCl3,298.0K, pulse sequence: cosygpppqf

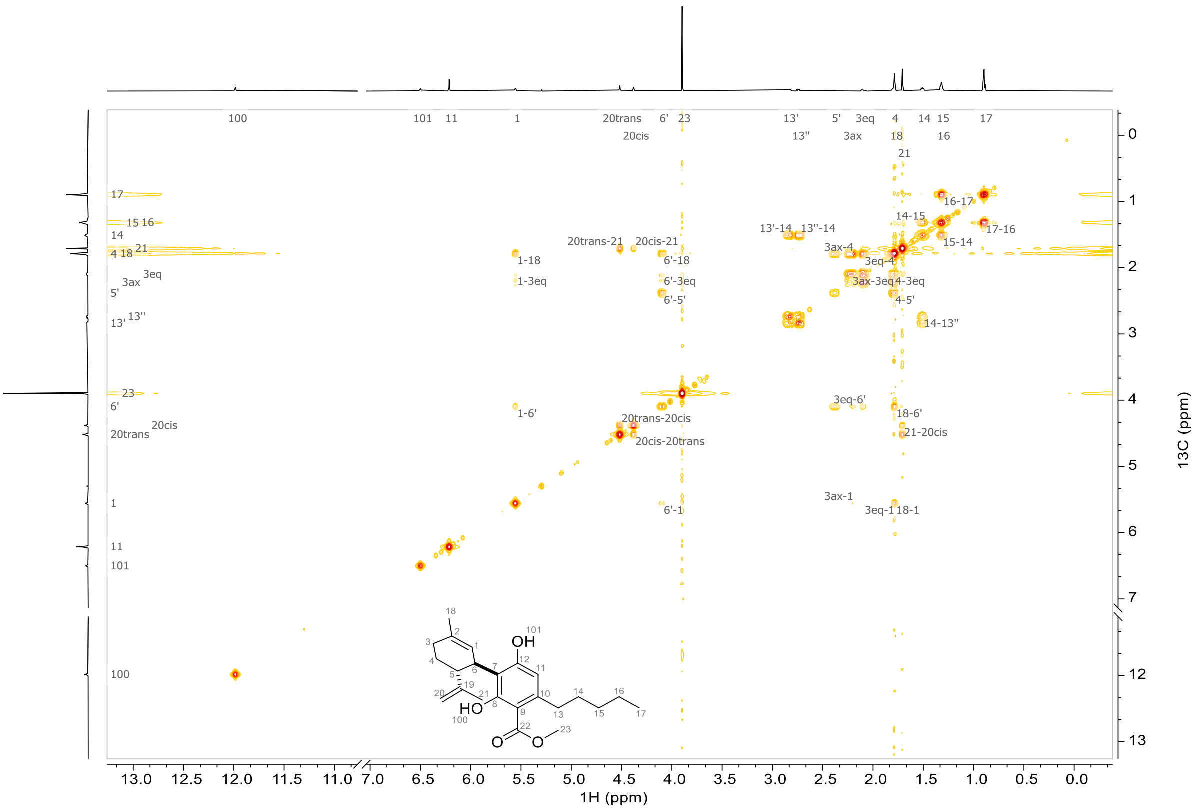

1H{off},NOESY, 600.20 MHz,CDCl3,298.0K, pulse sequence: noesygpphpp

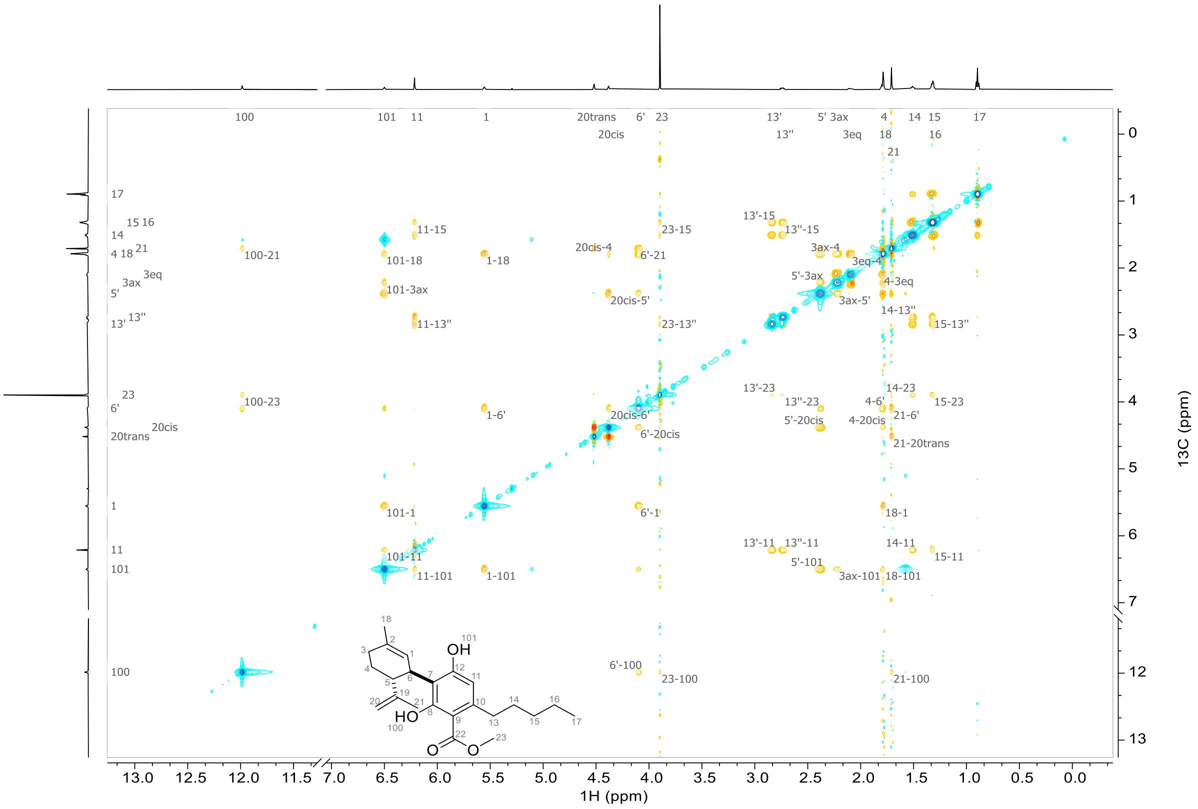

Δ^9-Tetrahydrocannabinol (**13**)

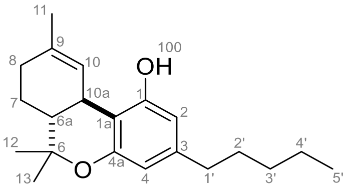

Atom	δ (ppm)	J	COSY	HSQC	HMBC	NOESY
1 C	154.286					
1' C	35.617			1'	2, 2', 3', 4	
H2	2.434		2'	1'	2, 2', 3, 3', 4	2, 2', 4
1a C	109.172				2, 4	
2 C	107.688			2	1', 4	
H	6.139	1.60(4)	4	2	1', 1a, 4	1', 2', 3'
2' C	30.792			2'	1', 3', 4'	
H2	1.556		1', 3'	2'	1', 3, 3', 4'	1', 2, 2', 3', 4
3 C	142.970				1', 2'	
3' C	31.656			3'	1', 2', 4', 5'	
H2	1.286		2', 4'	3'	1', 2', 4', 5'	2, 2', 4
4 C	110.246			4	1', 2	
H	6.272	1.60(2)	2	4	1', 1a, 2, 4a	1', 2', 3'
4' C	22.688			4'	2', 3', 5'	
H2	1.311	7.10(5')	3', 5'	4'	2', 3', 5'	
4a C	154.912				4, 12	
5' C	14.158			5'	3', 4'	
H3	0.879	7.10(4')	4'	5'	3', 4'	
6 C	77.358				6a, 12, 13	
6a C	45.946			6a	7ax, 7eq, 8, 10, 12, 13	
H	1.692	11.10(10a), 2.10(7ax)	10a	6a	6, 7, 10a, 12, 13	7eq, 8

Atom	δ (ppm)	J	COSY	HSQC	HMBC	NOESY
7 C	25.161			7ax, 7eq	6a, 8	
Hax	1.395	2.10(6a)	7eq, 8	7	6a	7eq, 8, 10a
Heq	1.914		7ax, 8	7	6a, 8, 9, 10a	6a, 7ax, 8
8 C	31.309			8	7eq, 10, 11	
H2	2.168		7ax, 7eq, 10, 10a	8	6a, 7, 9, 10	6a, 7ax, 7eq, 11
9 C	134.563				7eq, 8, 11	
10 C	123.853			10	8, 11	
H	6.303		8, 10a, 11	10	6a, 8, 10a, 11	10a, 11, 100
10a C	33.711			10a	6a, 7eq, 10	
H	3.201	11.10(6a)	6a, 8, 10	10a		7ax, 10, 13
11 C	23.514			11	10	
H3	1.681		10	11	8, 9, 10	8, 10
12 C	27.714			12	6a, 13	
H3	1.413		13	12	4a, 6, 6a	13
13 C	19.417			13	6a	
H3	1.093		12	13	6, 6a, 12	10a, 12
100 O						
H	4.755					10

Remarks:

The large $^3J_{H6a,H10a}$ value (11.1 Hz) supports their *trans*-configuration. Exchange of OH-100 and H2 is likely due to the keto-enol-tautomerism.

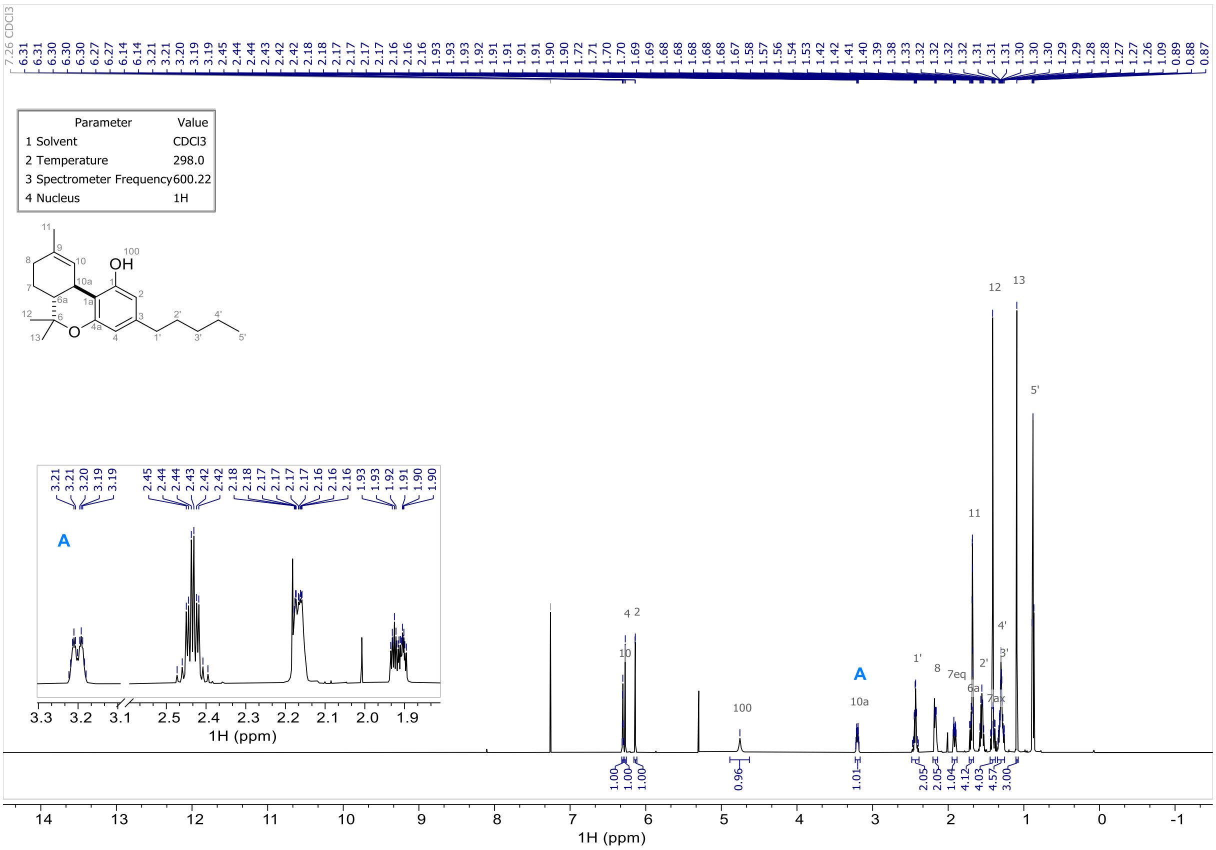

Parameter Value
1 Solvent CDCl3
2 Temperature 298.0
3 Spectrometer Frequency 600.22
4 Nucleus 1H
1H (ppm)

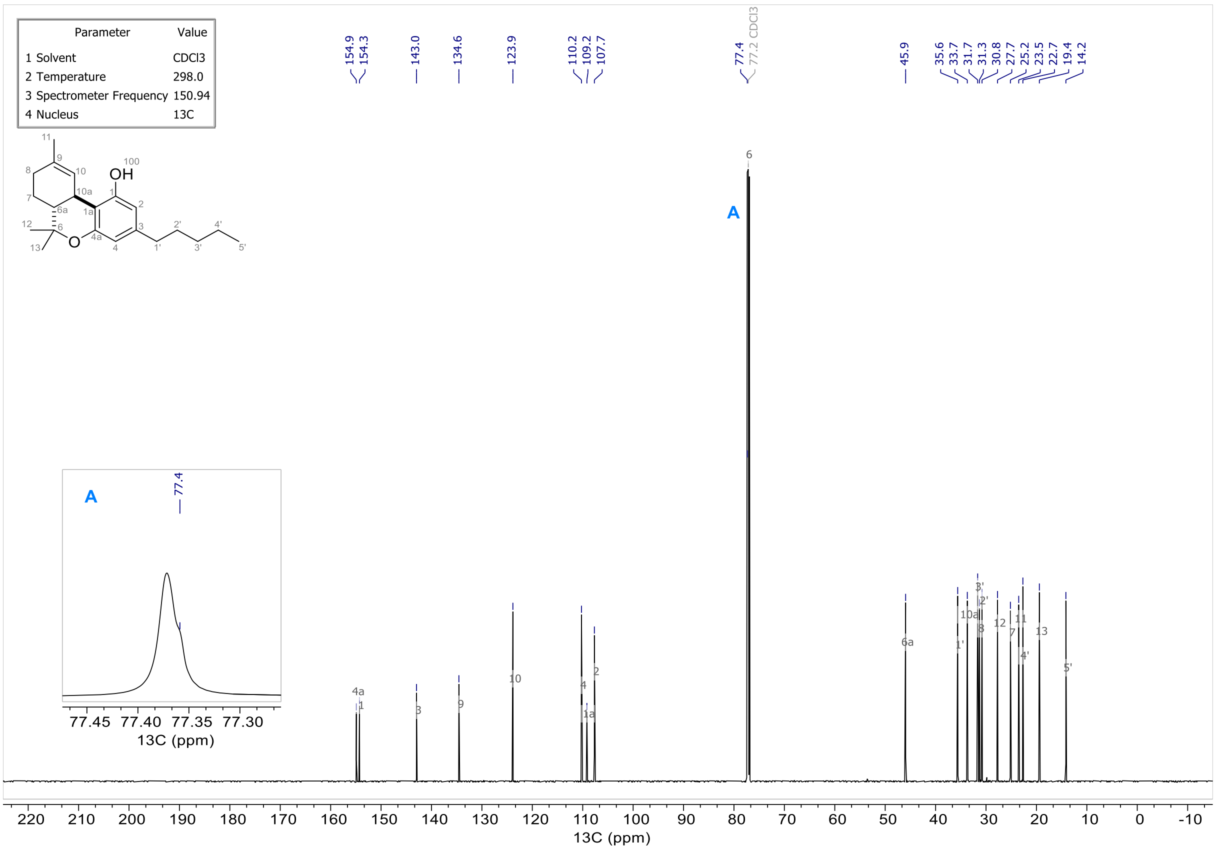

Parameter Value
1 Solvent CDCl3
2 Temperature 298.0
3 Spectrometer Frequency 150.94
4 Nucleus 13C
13C (ppm)

1H,1H-COSY

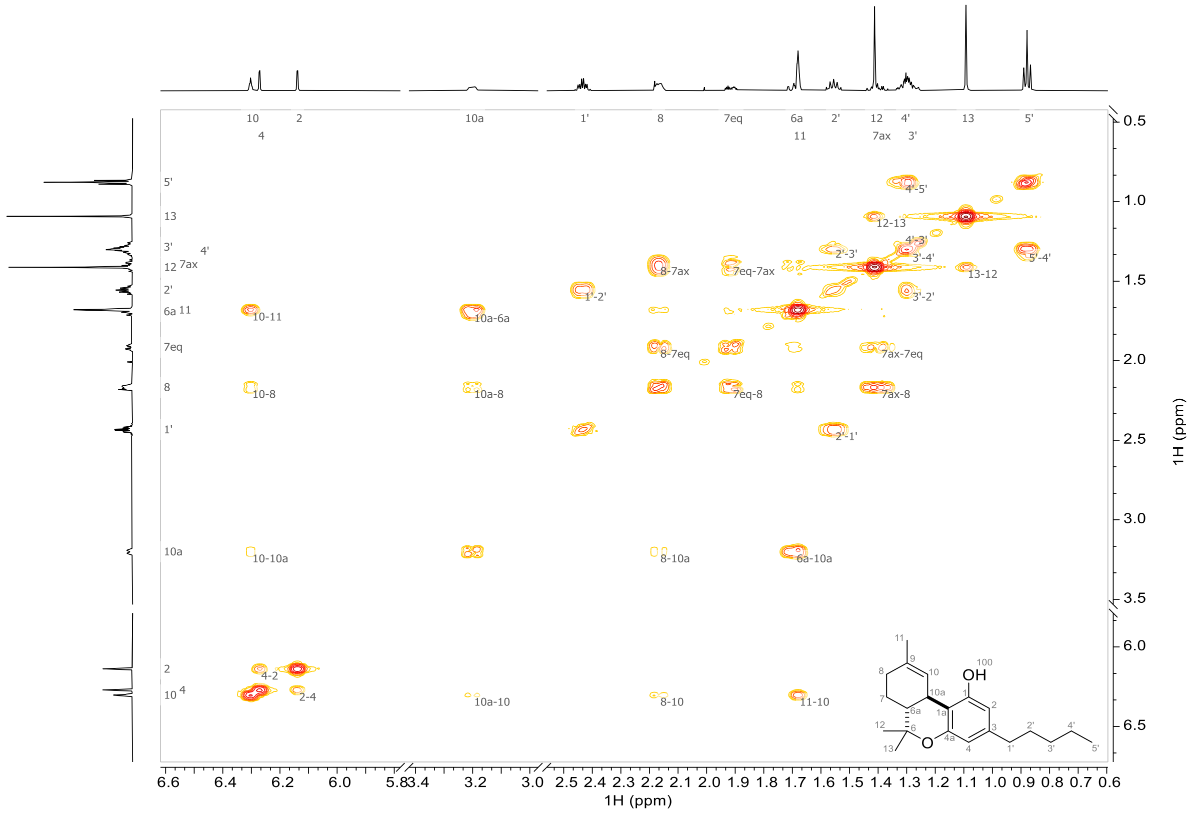

1H,13C-HSQC-EDITED

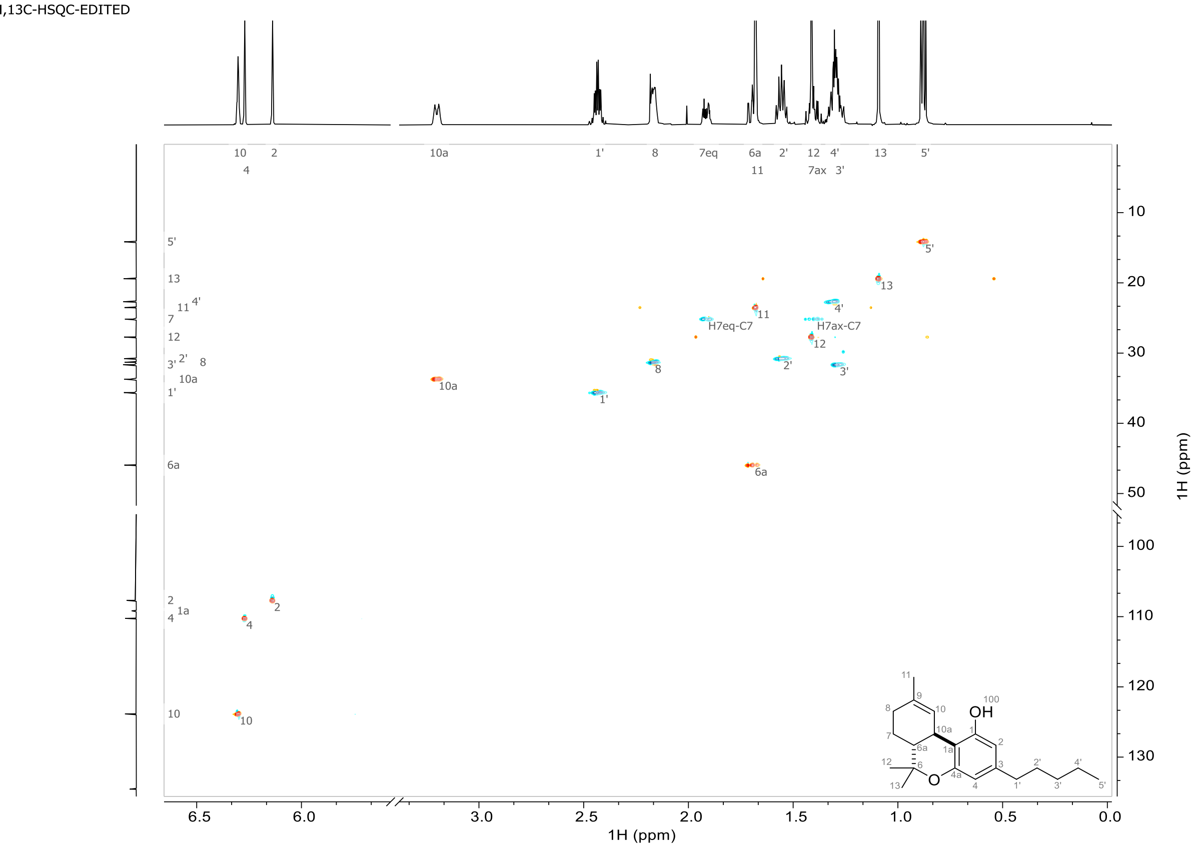

1H,13C-HMBC

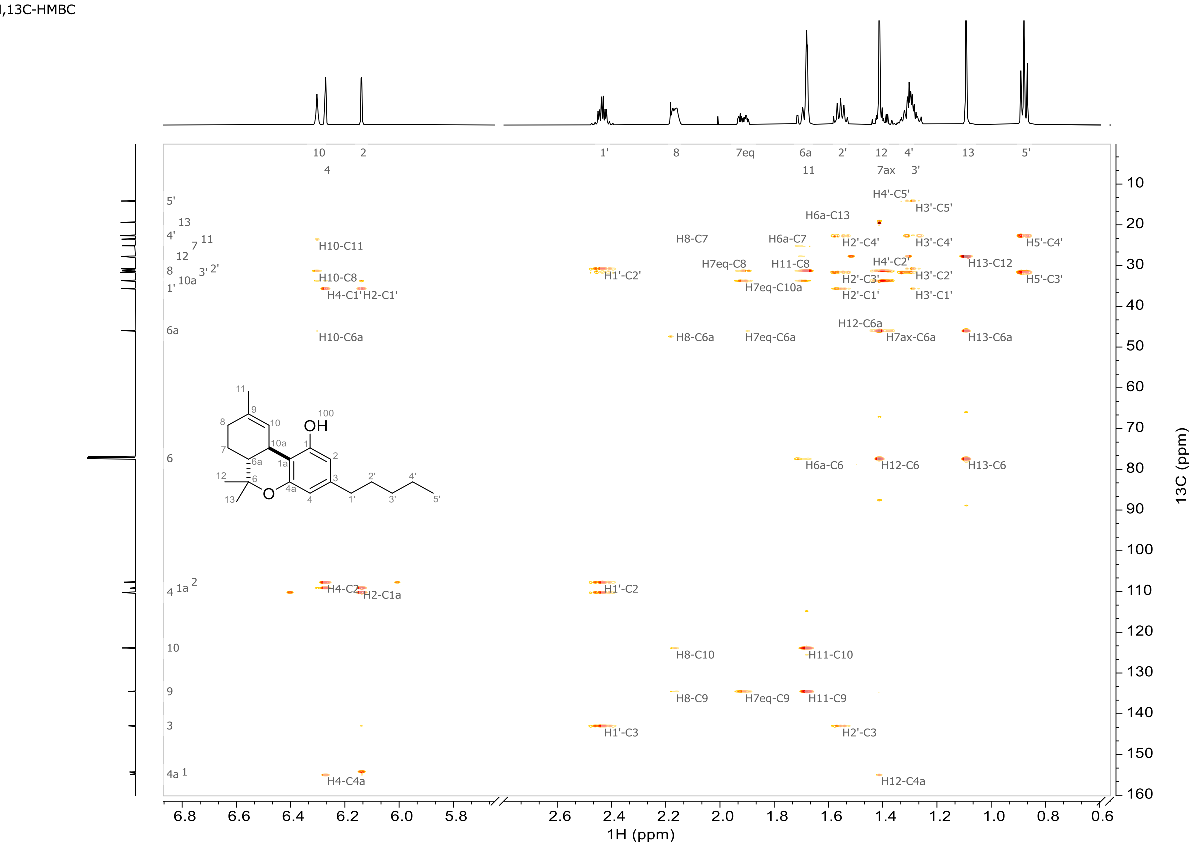

1H,1H-NOESY

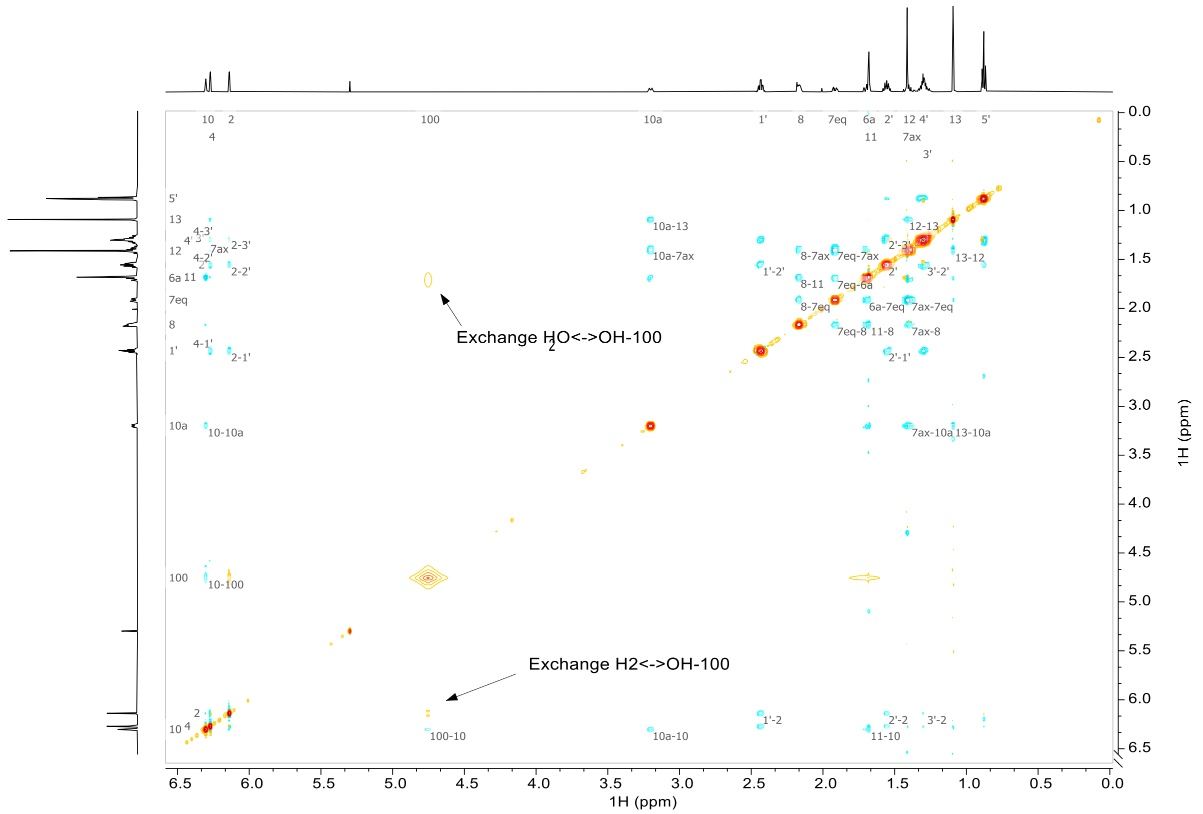

Δ^{8}-Tetrahydrocannabinol (**14**)

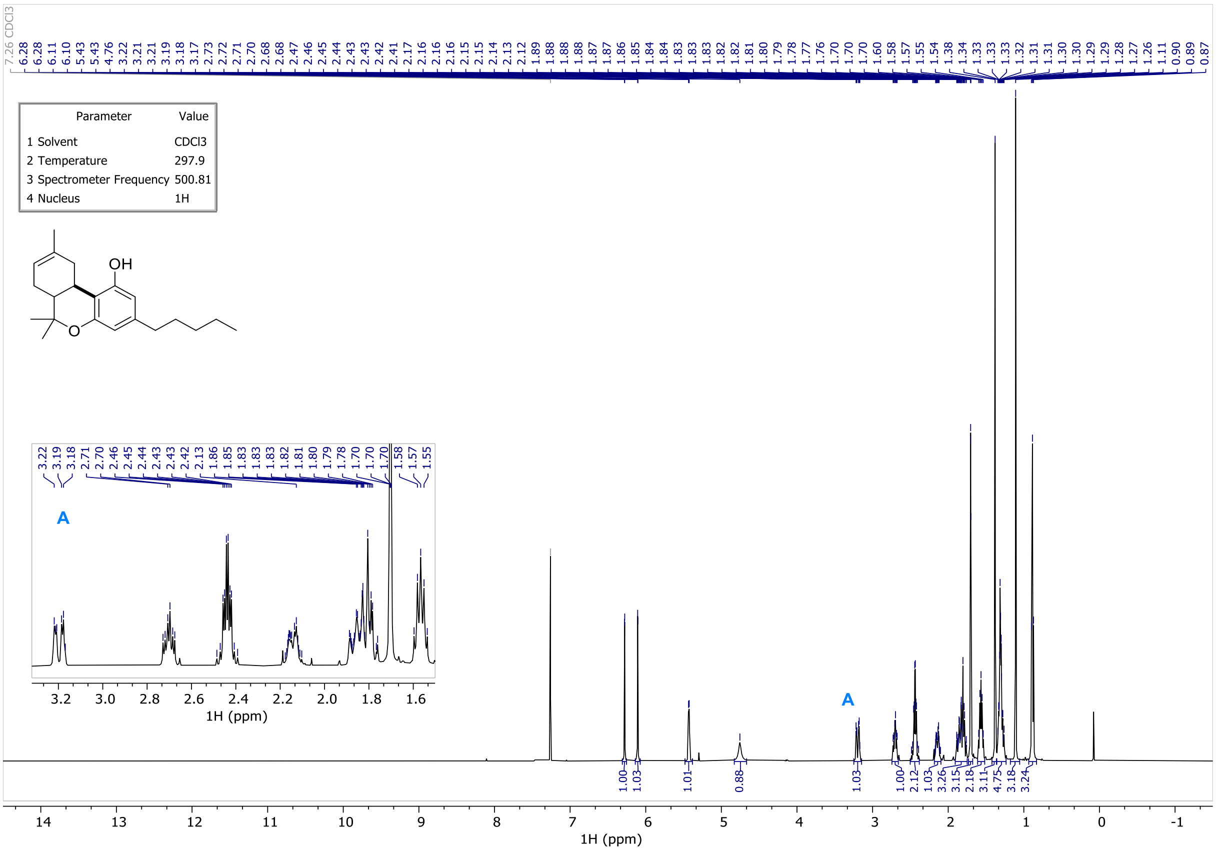

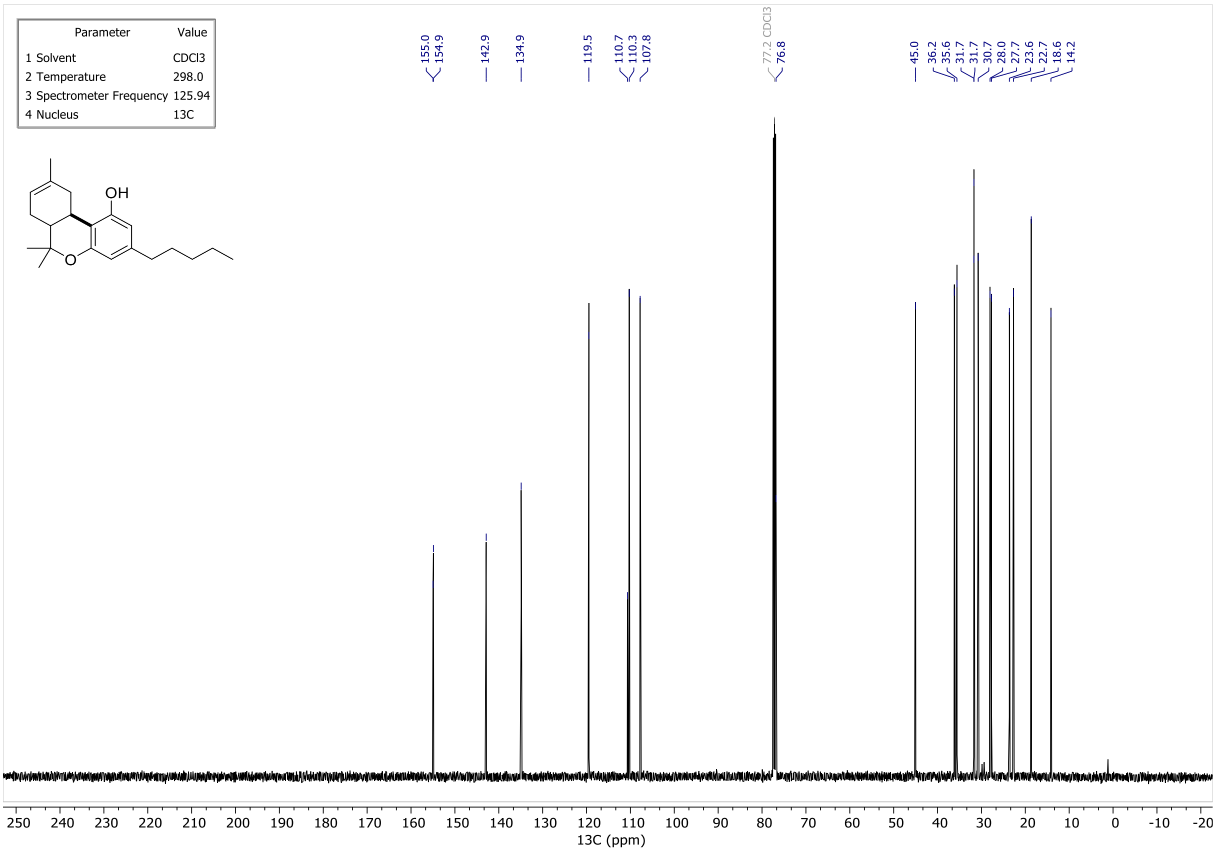

(10a*R*)-6,6,9-trimethyl-3-(2-methyloctan-2-yl)-6a,7,10,10a-tetrahydro-6*H*-benzo[c]chromen-1-ol (**15**)

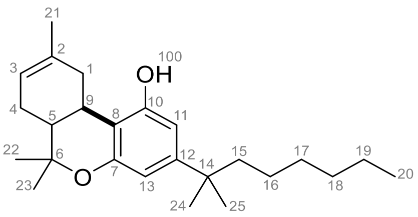

Chem3D model showing a few selected NOEs

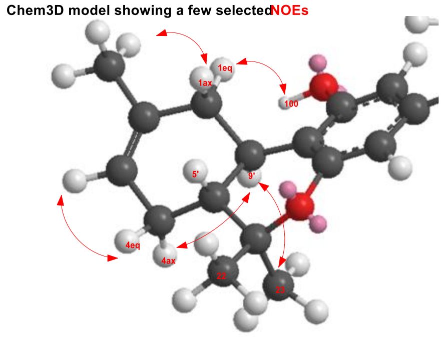

Atom	J	δ (ppm)	COSY	HSQC	HMBC	NOESY
1 C		36.177		1ax, 1eq	3, 9', 21	
Hax	16.80(1eq), 11.00(9')	1.875	1eq, 9', 21	1	2, 3, 5, 9	1eq, 21
Heq	16.80(1ax), 4.80(9')	3.195	1ax, 9', 21	1	2, 3, 5, 9, 21	1ax, 9', 21, 100
2 C		134.893			1ax, 1eq, 4ax, 4eq, 21	
3 C		119.480		3	1ax, 1eq, 4ax, 4eq, 21	
H		5.432	4eq, 21	3	1, 4, 5, 21	4ax, 4eq, 21
4 C		28.034		4ax, 4eq	3, 5', 9'	
Hax		1.827	4eq, 21	4	2, 3, 6, 9	3, 9', 23
Heq		2.148	3, 4ax, 5', 21	4	2, 3, 5, 9	3, 4eq, 5', 22
5 C		45.009		5'	1ax, 1eq, 3, 4eq, 9', 22, 23	
5' H	11.00(9')	1.796	4eq, 9'	5	4, 6	4eq, 22, 23
6 C		76.817			4ax, 5', 22, 23	
7 C		154.658			9', 13	
8 C		110.305			9', 11, 13, 100	
9 C		31.657		9'	1ax, 1eq, 4ax, 4eq	
9' H	11.00(1ax), 4.80(1eq), 11.00(5')	2.696	1ax, 1eq, 5'	9	1, 4, 5, 7, 8, 10	1eq, 4ax, 23
10 C		154.555			9', 11, 100	
11 C		105.556		11	13, 100	
H	1.80(13)	6.228	13	11	8, 10, 13, 14	15, 16, 24, 25, 100
12 C		150.175			15, 24, 25	
13 C		108.186		13	11	
H	1.80(11)	6.392	11	13	7, 8, 11, 14	15, 16, 24, 25
14 C		37.457			11, 13, 15, 24, 25	
15 C		44.622		15	16, 24, 25	
H2		1.497	16	15	12, 14, 16, 17, 24, 25	11, 13, 16, 17

Atom	J	δ (ppm)	COSY	HSQC	HMBC	NOESY
16 C		24.766		16	15	
H2		1.064	15, 17	16	15, 17, 18	11, 13, 15
17 C		30.175		17	15, 16, 19	
H2		1.174	16	17		15
18 C		31.934		18	16, 19, 20	
H2		1.204		18		
19 C		22.825		19	20	
H2		1.241	20	19	17, 18, 20	
20 C		14.239		20	19	
H3		0.848	19	20	18, 19	
21 C		23.640		21	1eq, 3	
H3		1.701	1ax, 1eq, 3, 4ax, 4eq	21	1, 2, 3	1ax, 1eq, 3
22 C		27.761		22	23	
H3		1.390	23	22	5, 6, 23	4eq, 5', 23
23 C		18.671		23	22	
H3		1.115	22	23	5, 6, 22	4ax, 5', 9', 22
24 C		28.885		24	15, 25	
H3		1.203		24	12, 14, 15, 25	11, 13
25 C		28.818		25	15, 24	
H3		1.203		25	12, 14, 15, 24	11, 13
100 O						
H		4.676			8, 10, 11	1eq, 11

Remarks:

The large $^3J_{H9'H5'}$ value (~11 Hz) supports their *trans*-configuration.

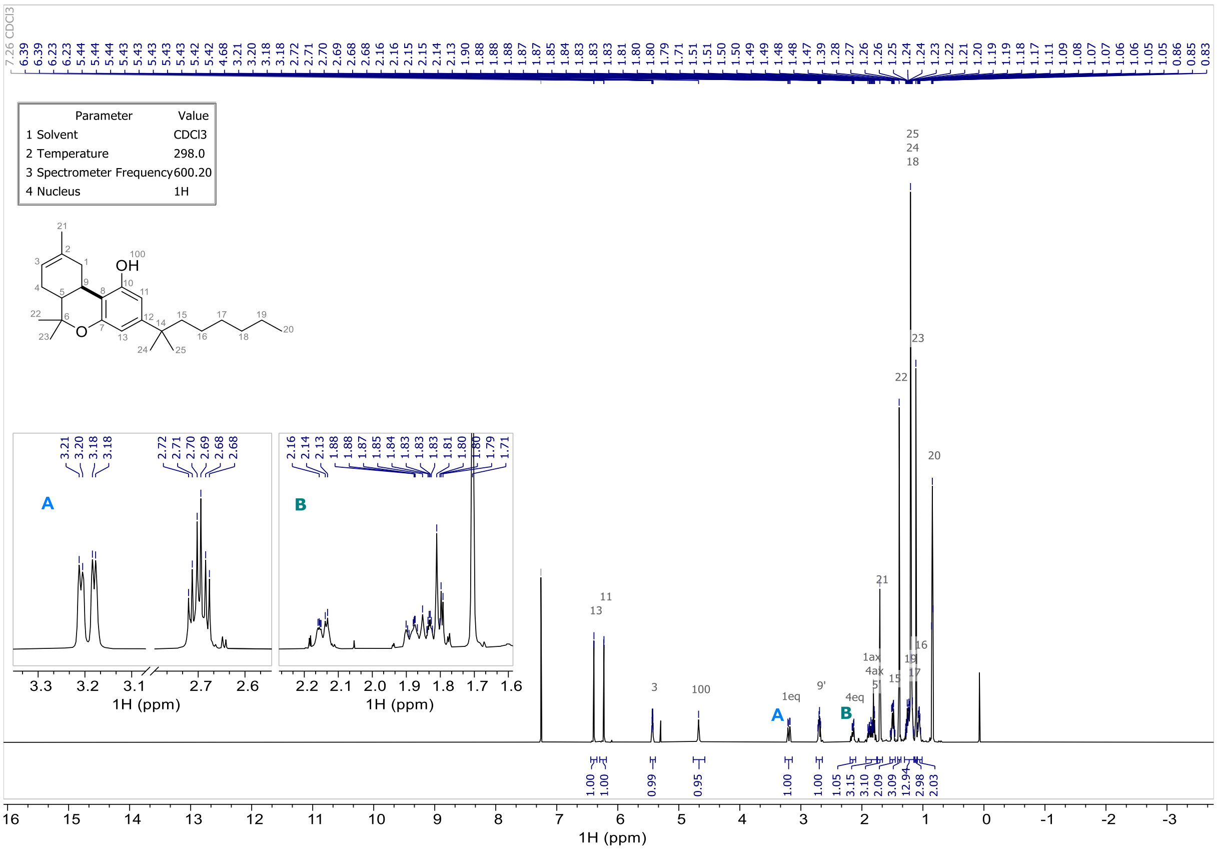

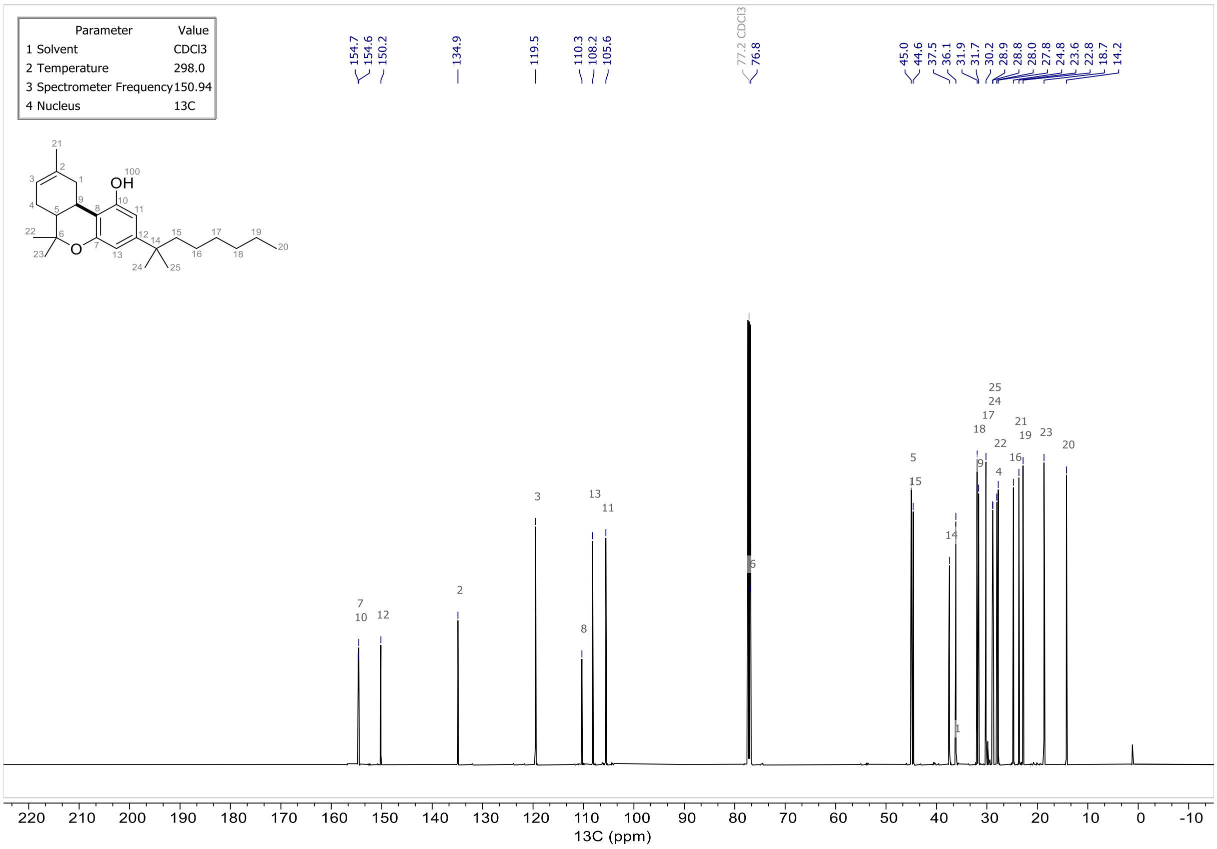

1H{13C},HSQC-EDITED, 600.20 MHz,CDCl3,298.0K, pulse sequence: hsqcedetgpsisp2.3

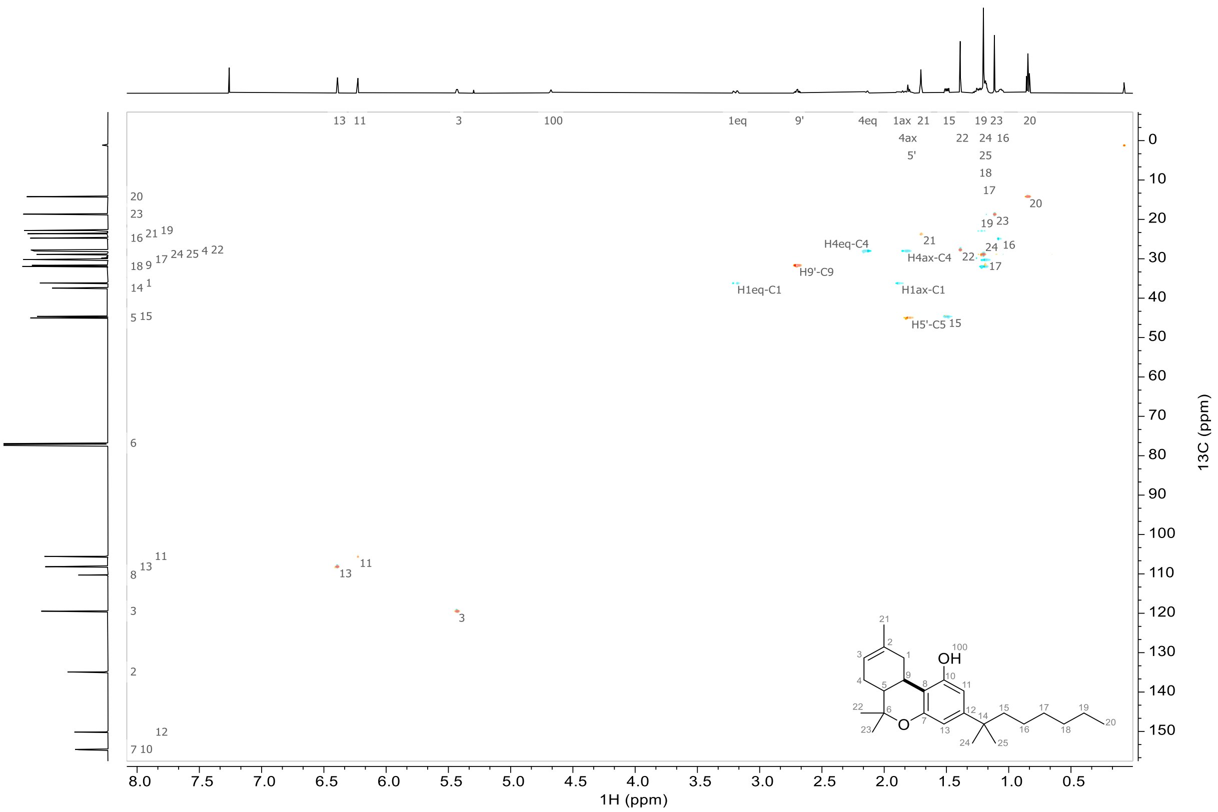

1H{13C},HMBC, 600.20 MHz,CDCl3,298.0K, pulse sequence: hmbcetgpl3nd

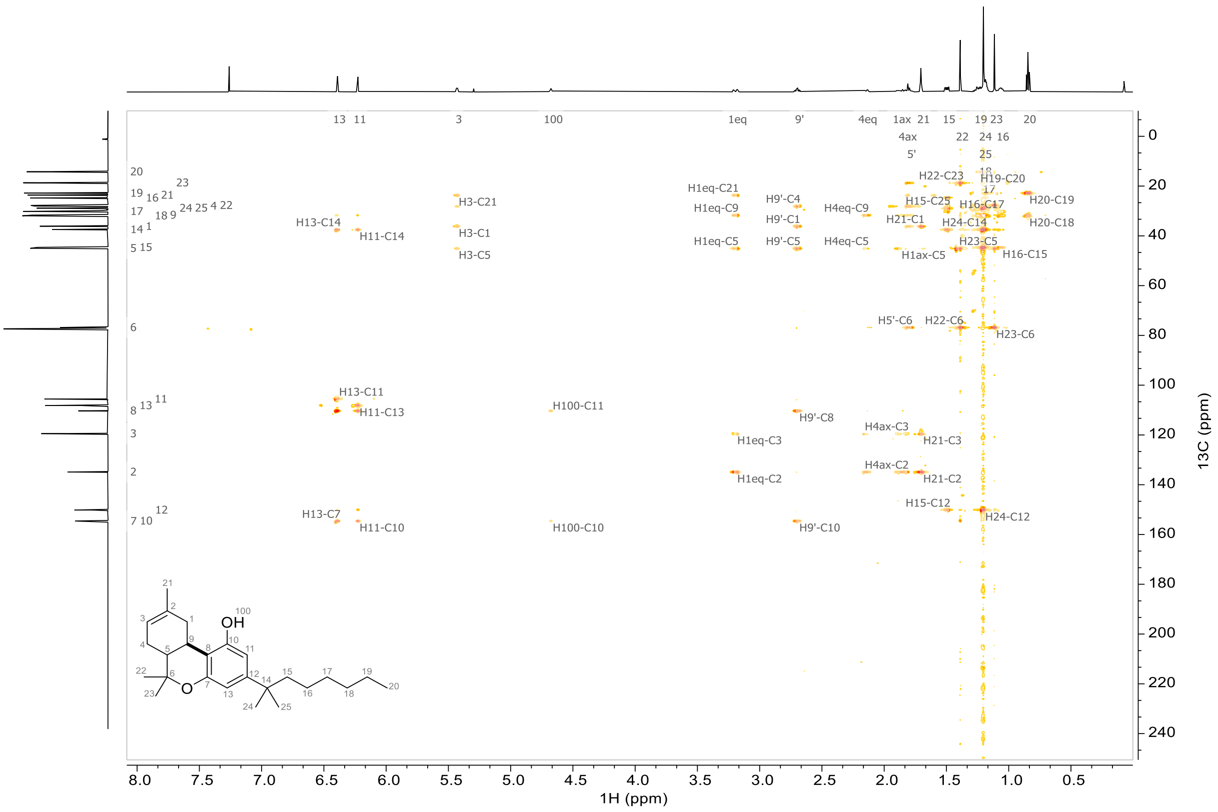

1H{off},COSY, 600.20 MHz,CDCl3,298.0K, pulse sequence: cosygpppqf

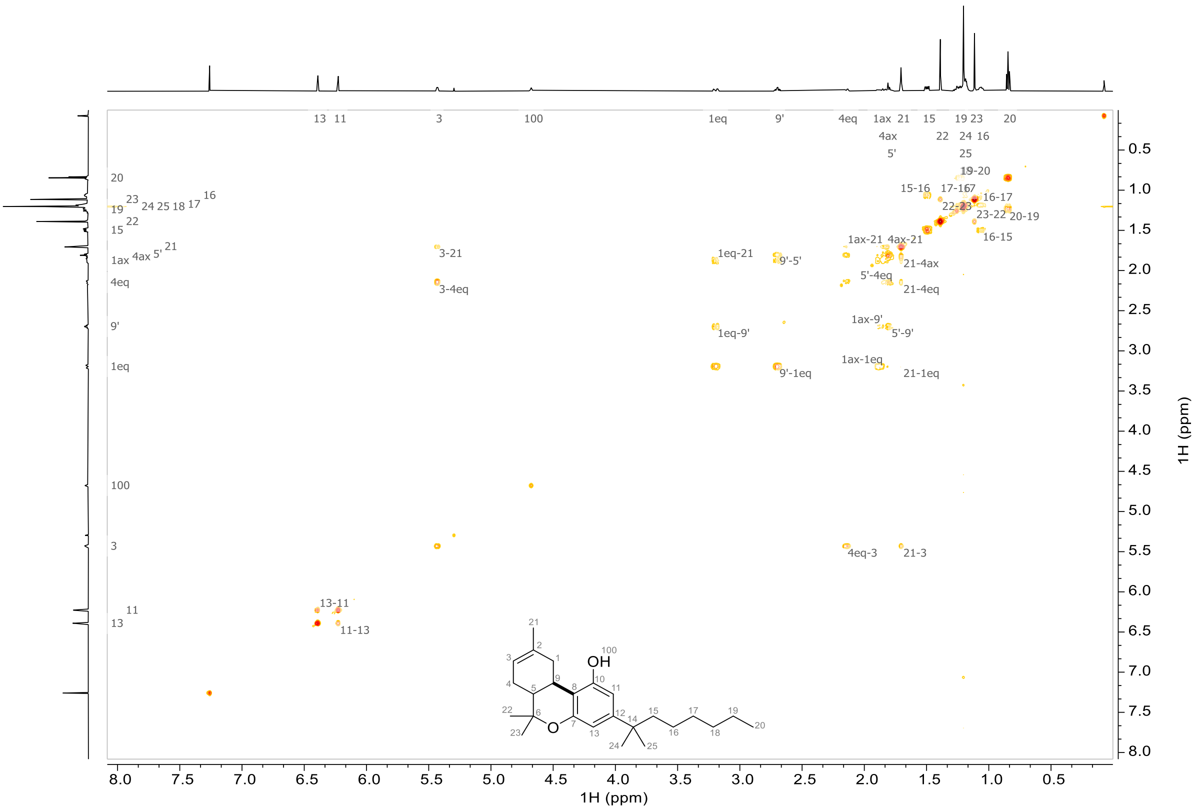

1H{off},NOESY, 600.20 MHz,CDCl3,298.0K, pulse sequence: noesygpphpp

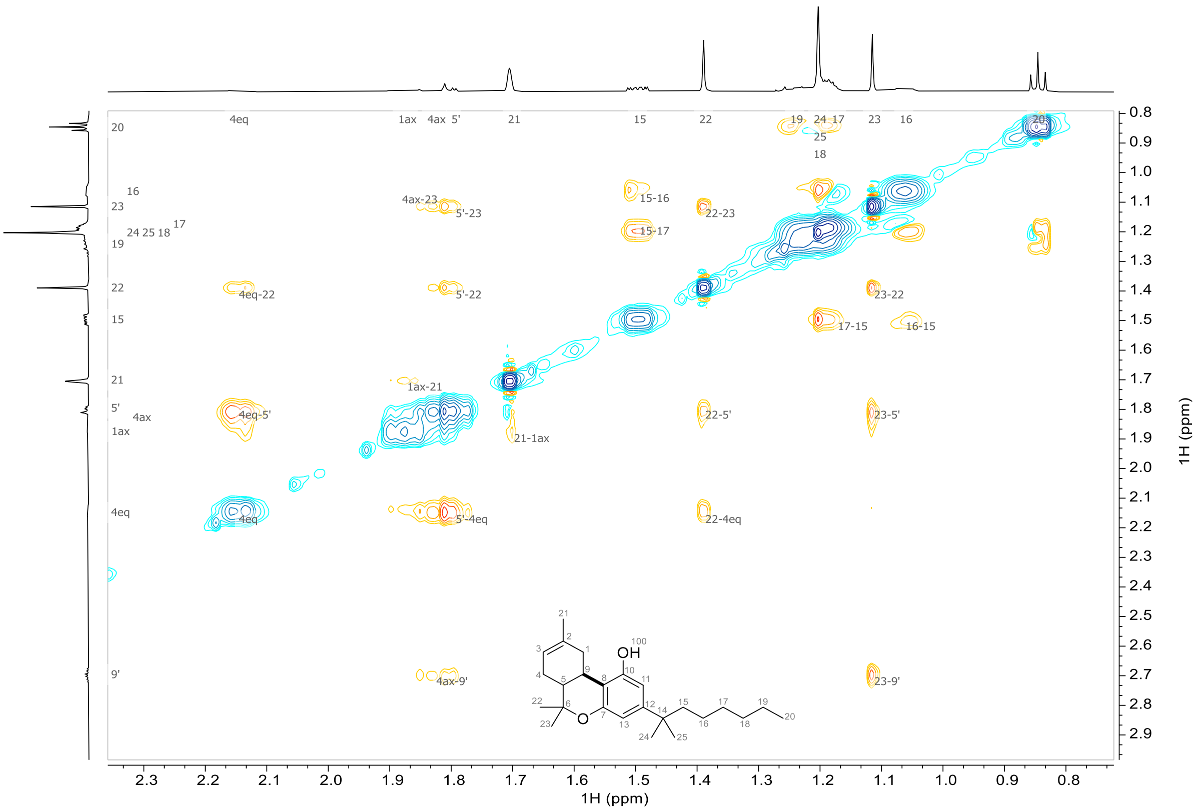

ethyl (*Z*)-3-ethyl-7-methylocta-2,6-dienoate (**S27**)

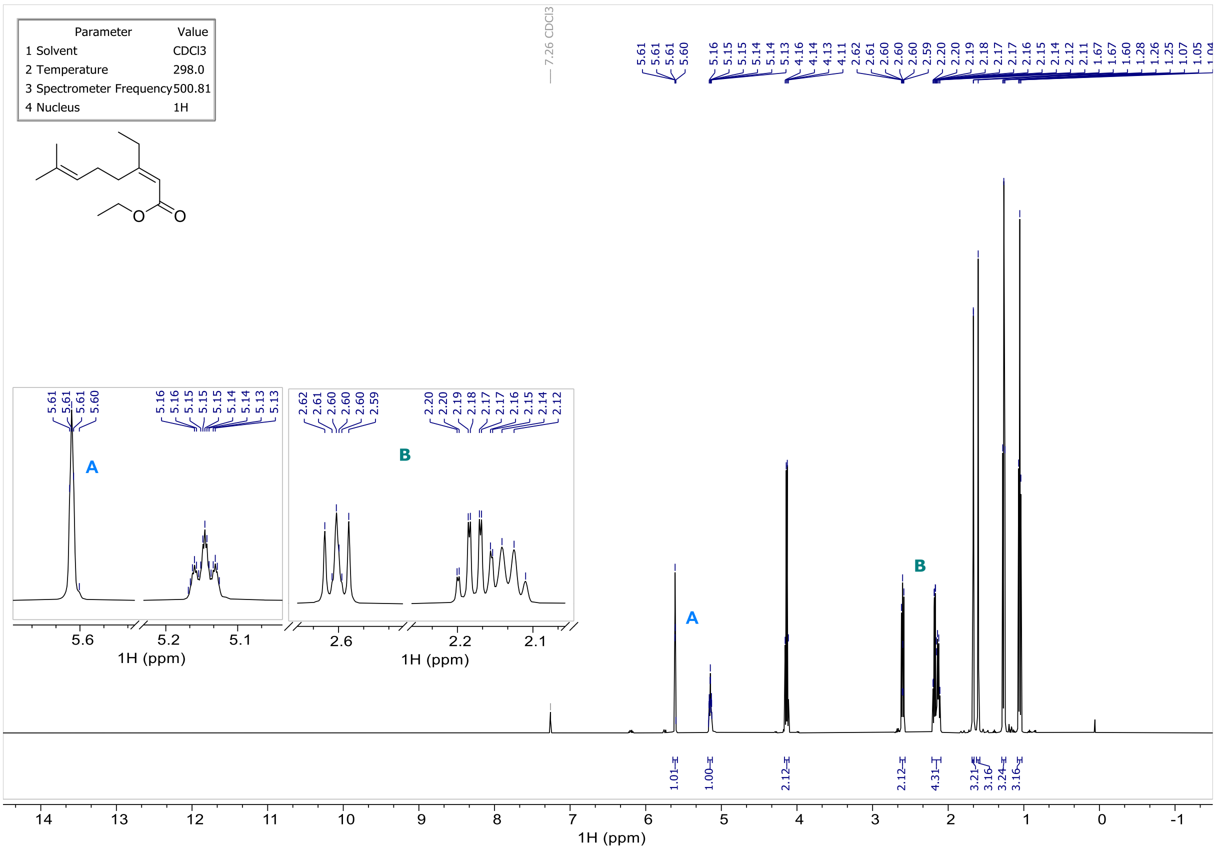

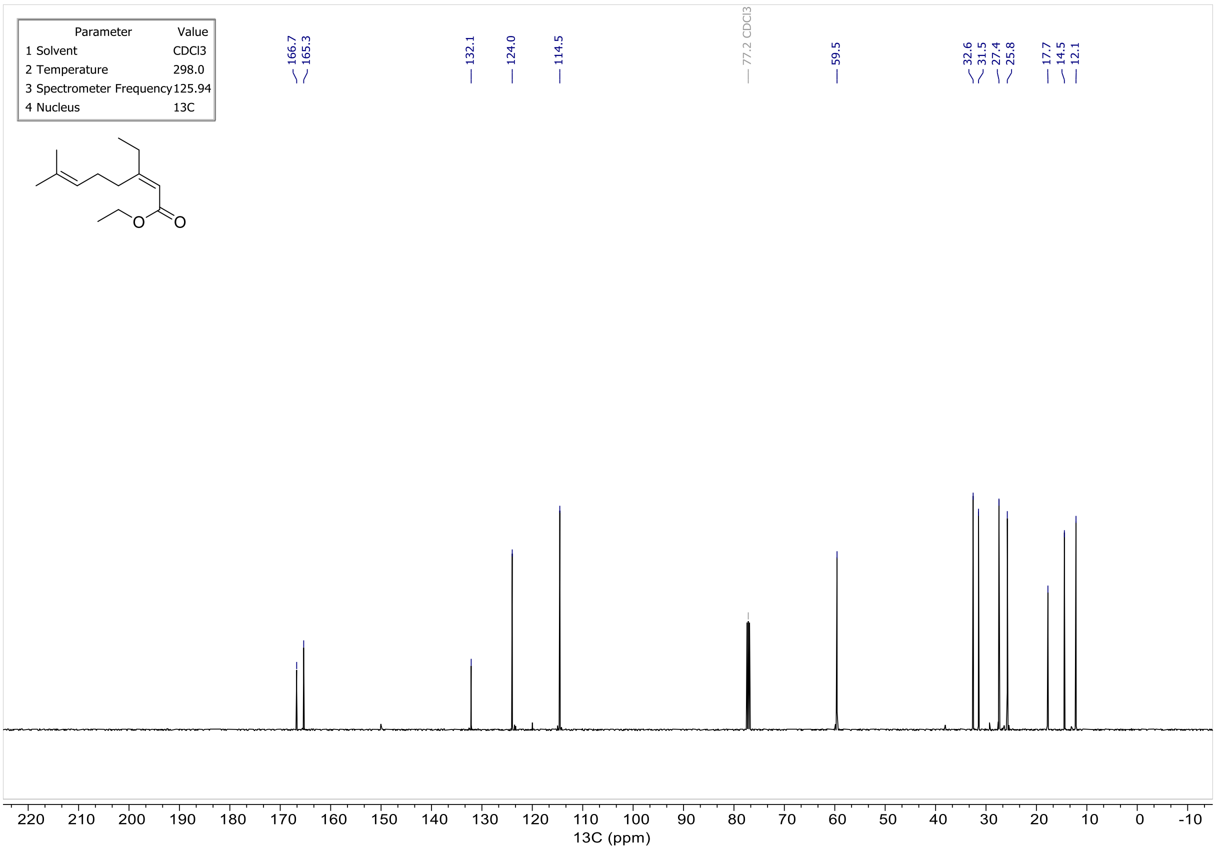

ethyl (*Z*)-7-methyl-3-propylocta-2,6-dienoate (**S28**)

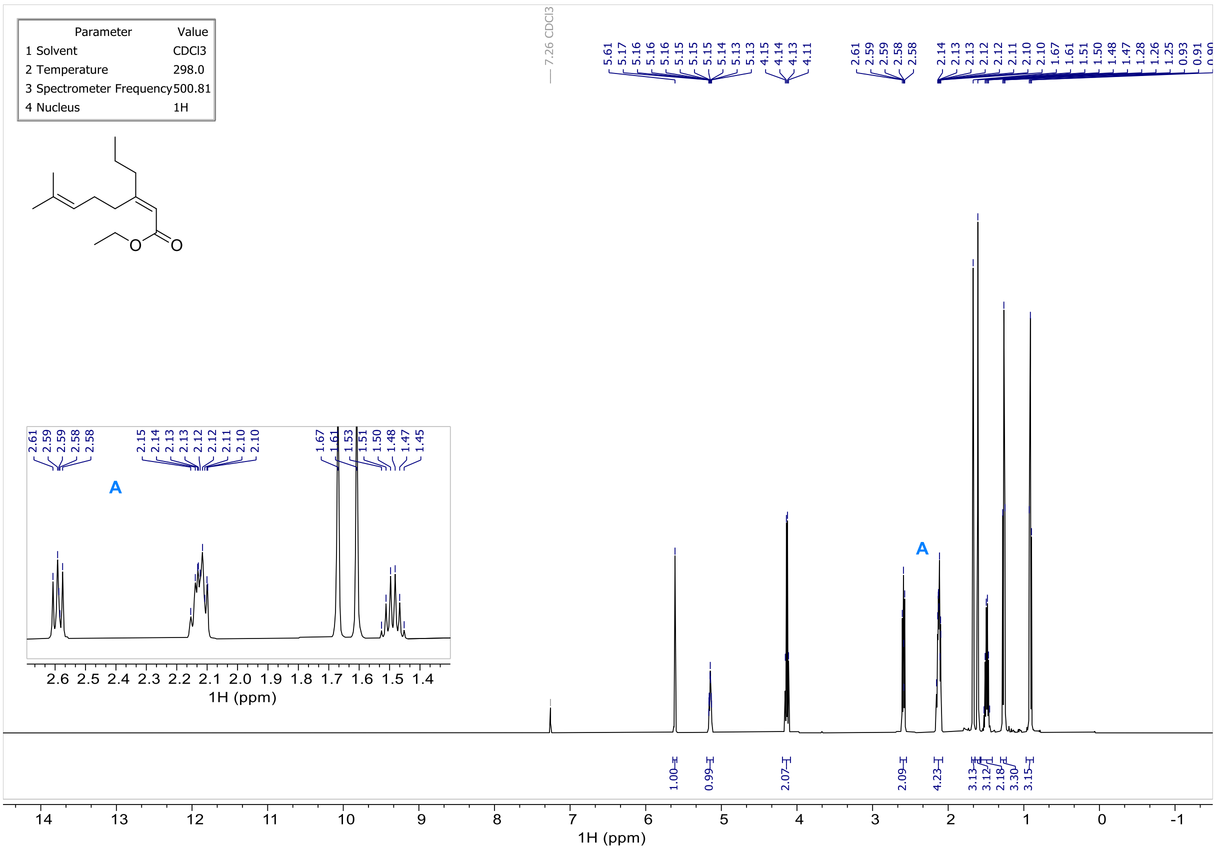

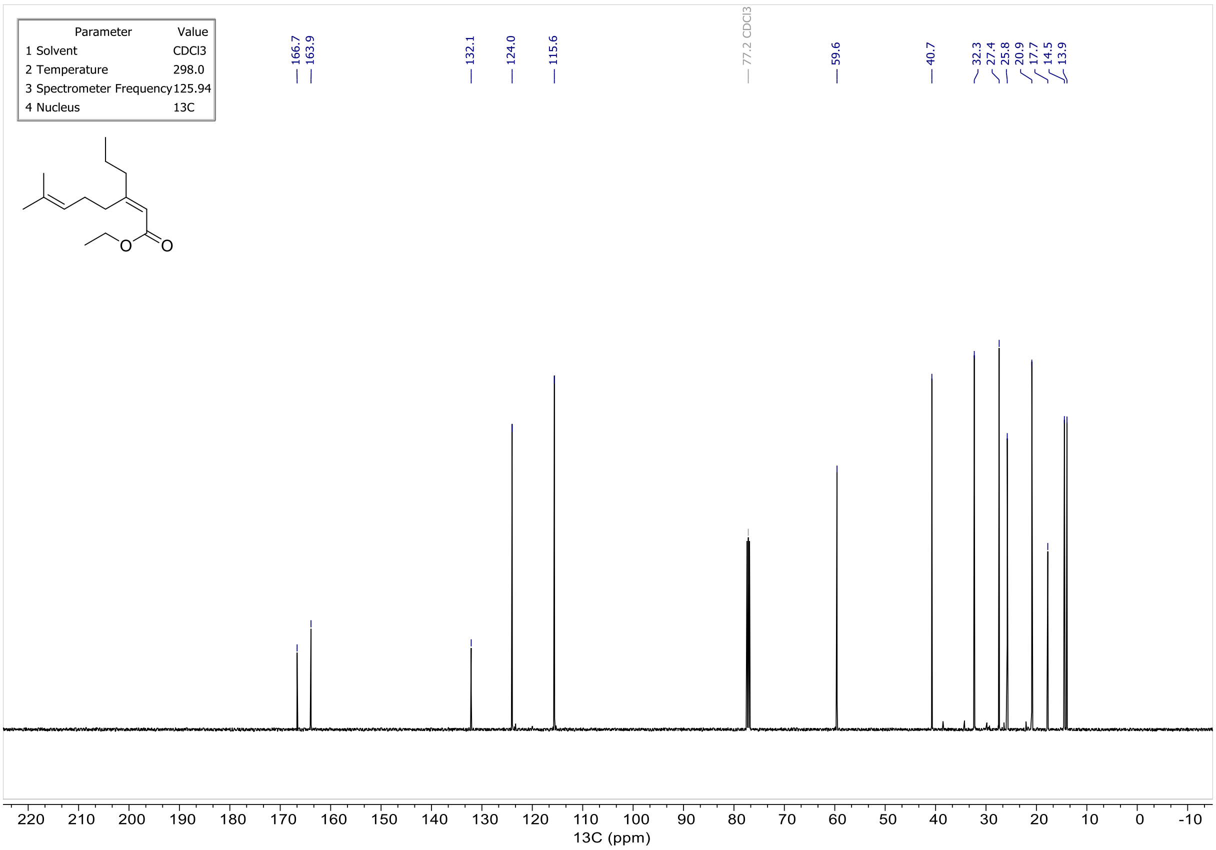

ethyl (*E*)-3-isobutyl-7-methylocta-2,6-dienoate (**S29**)

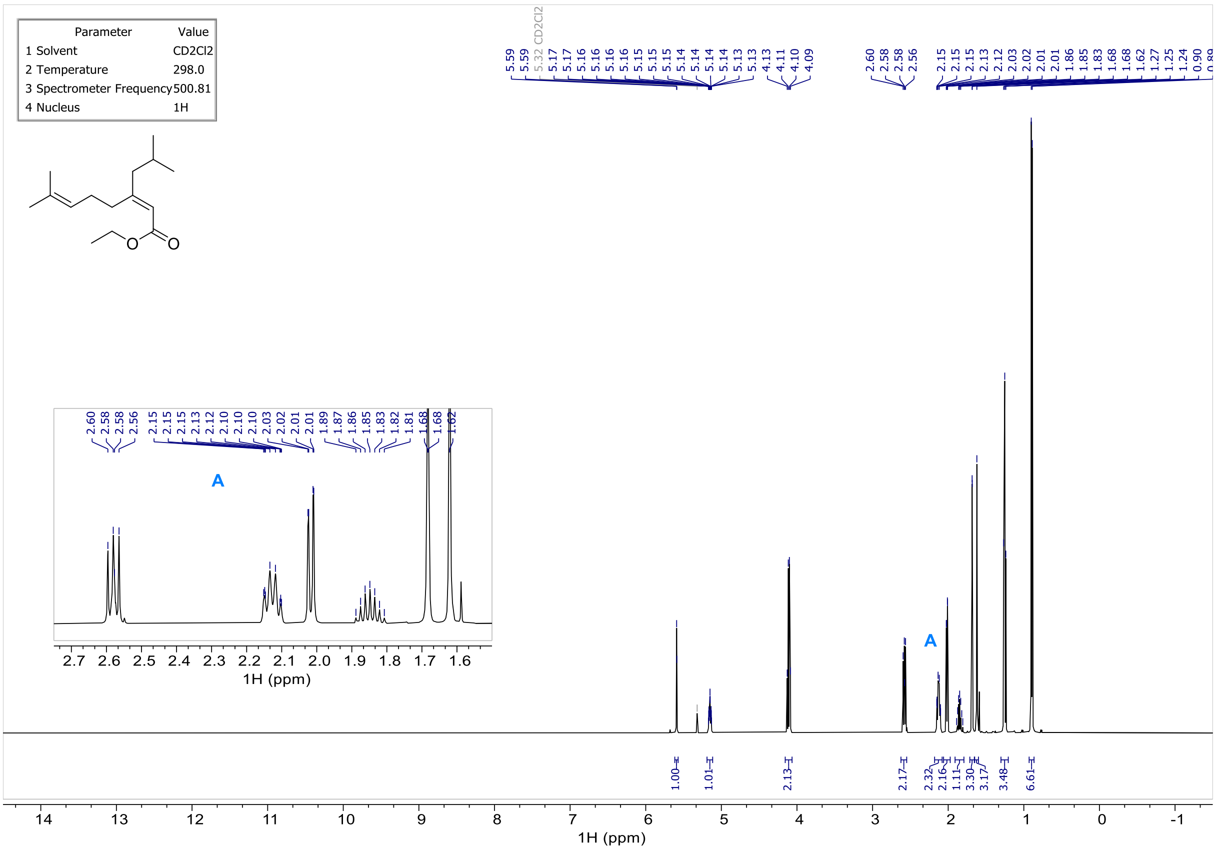

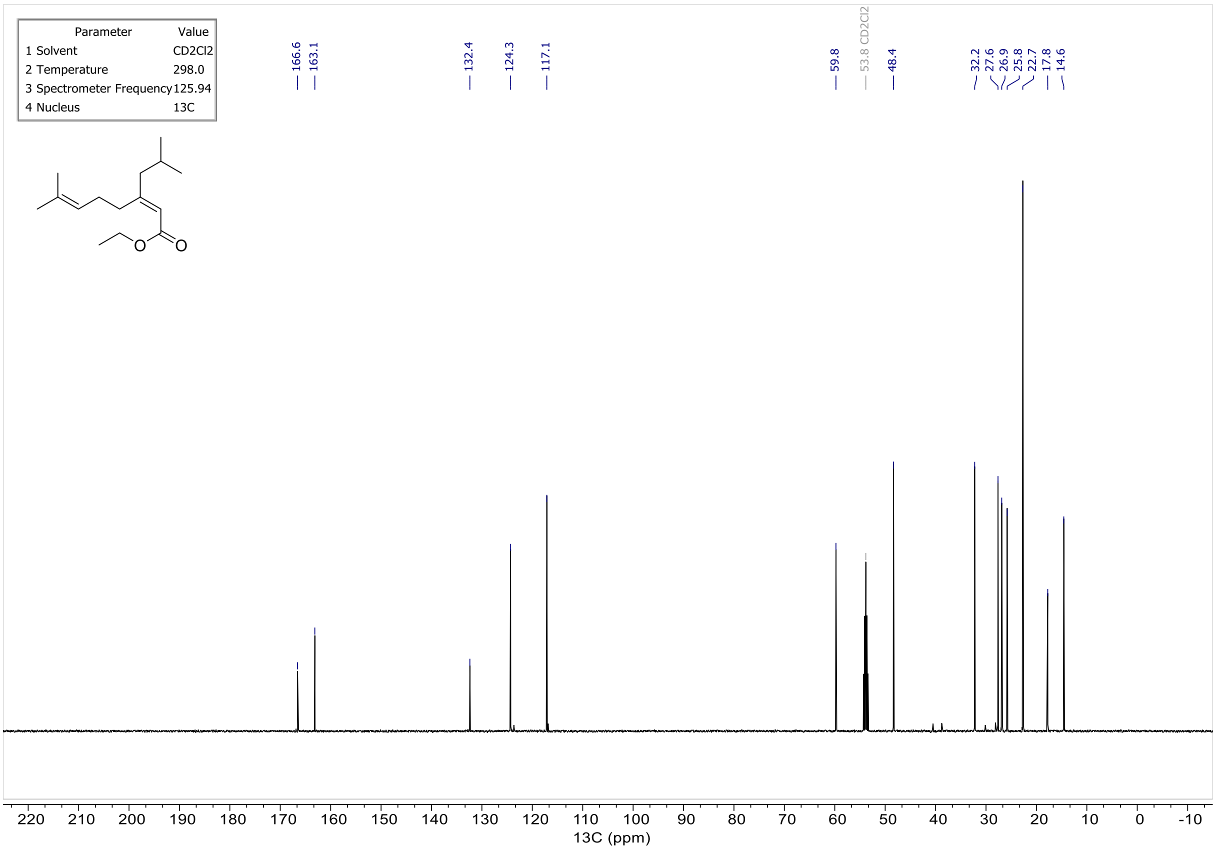

ethyl (*E*)-3-benzyl-7-methylocta-2,6-dienoate (**S30**)

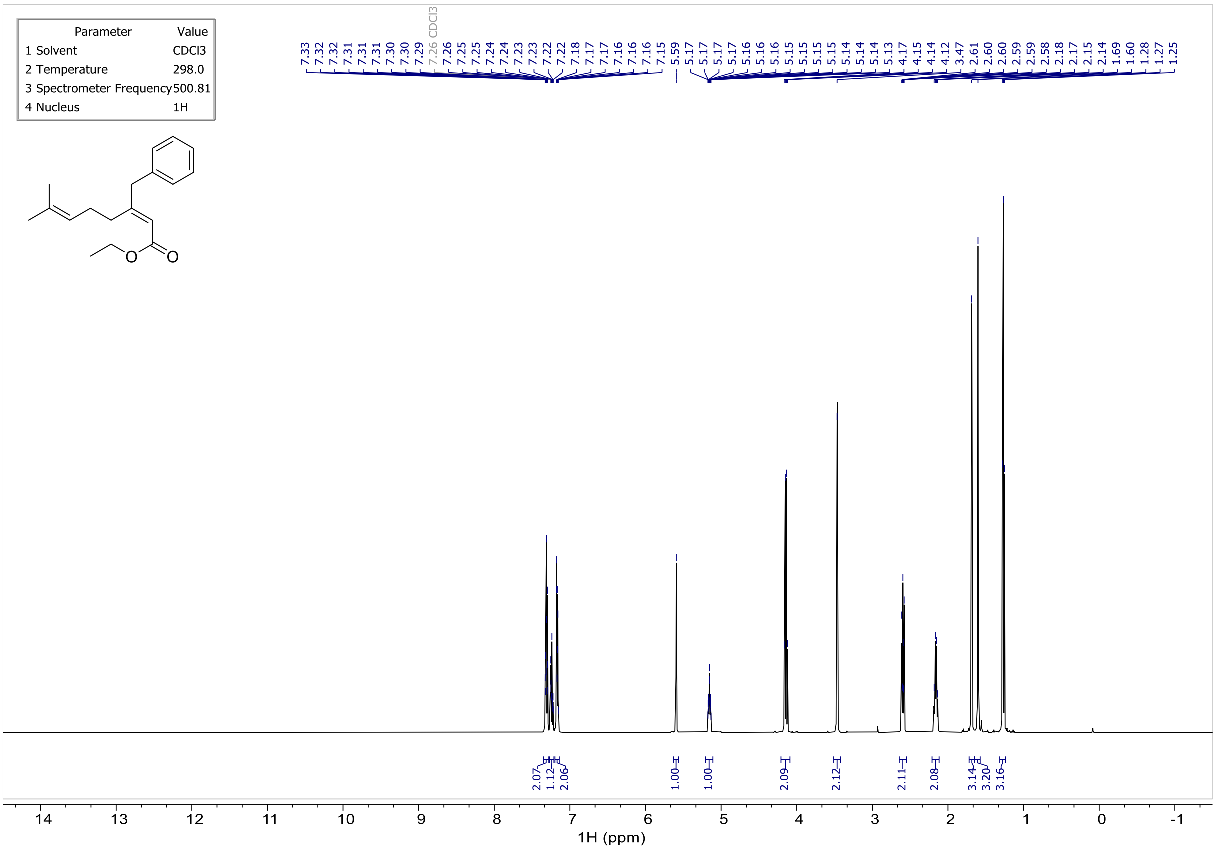

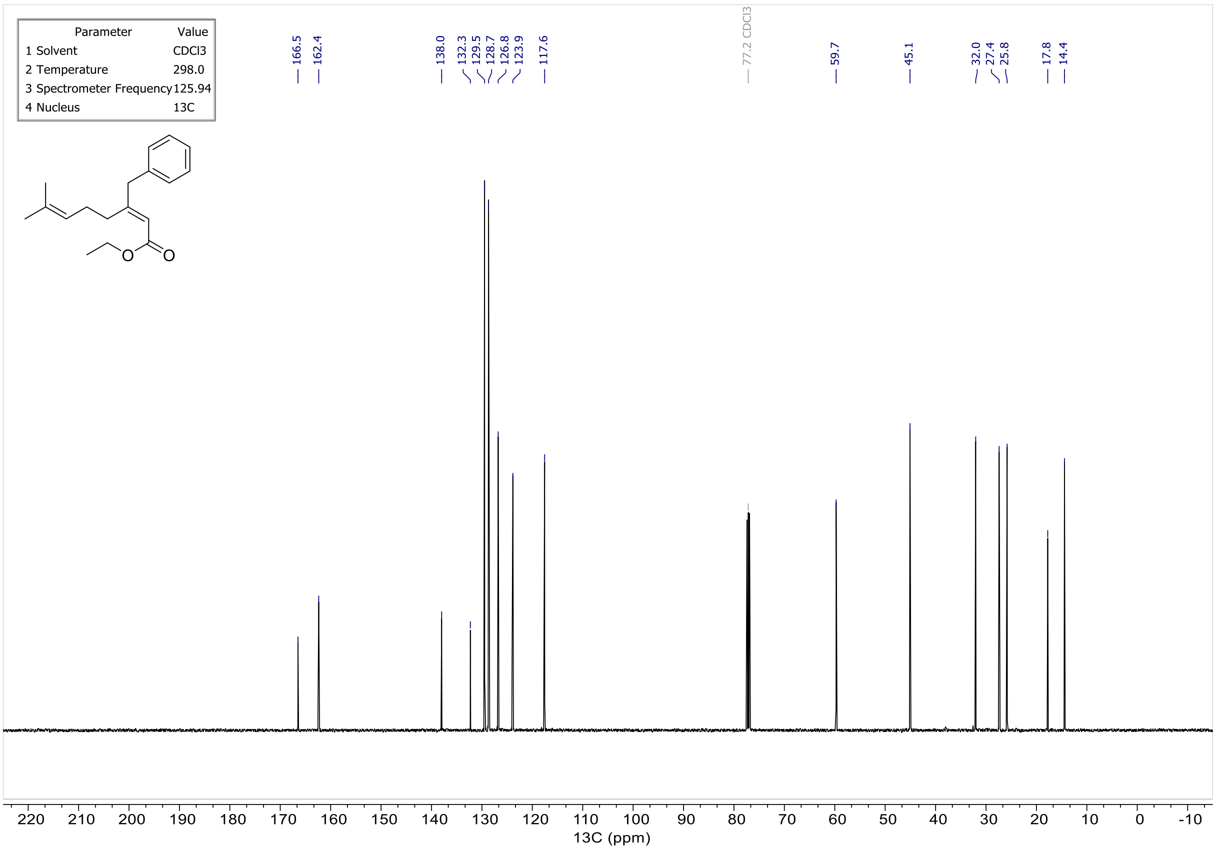

ethyl (*E*)-7-methyl-3-phenethylocta-2,6-dienoate (**S31**)

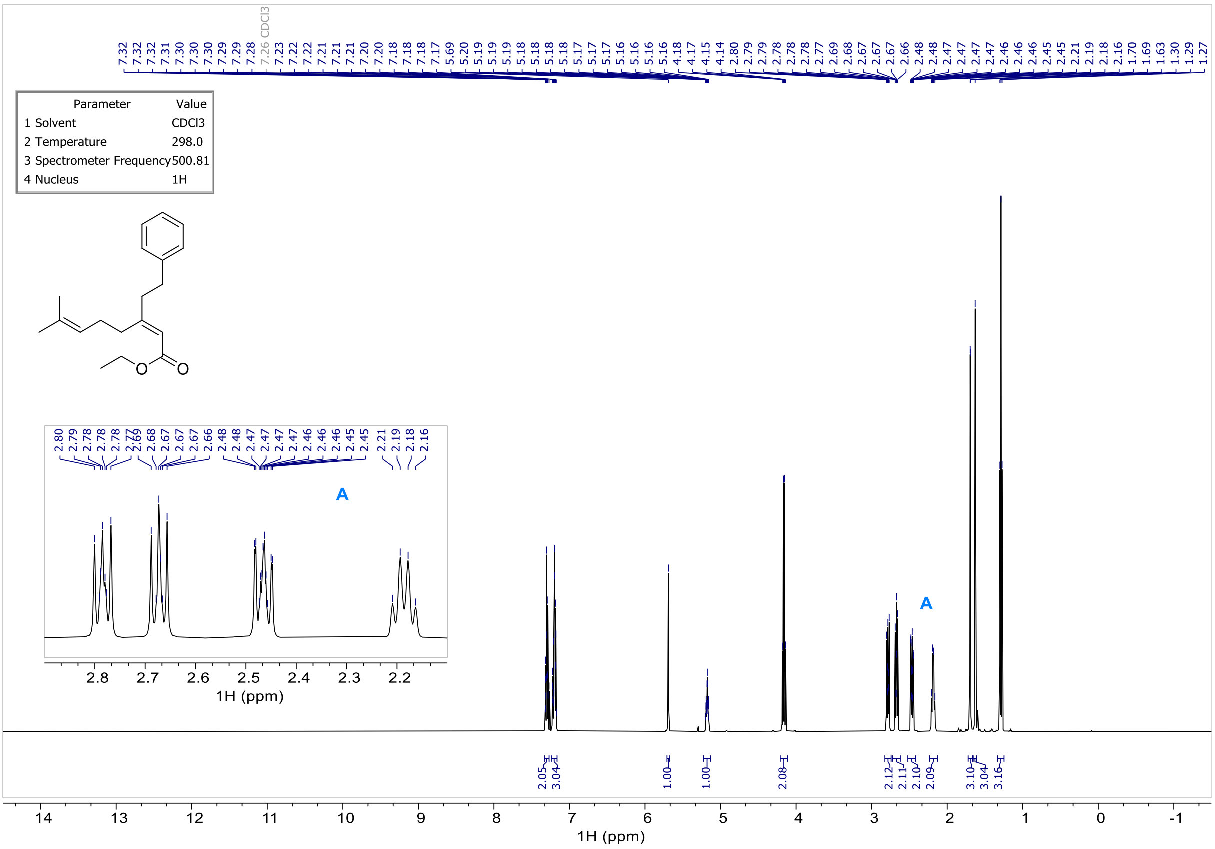

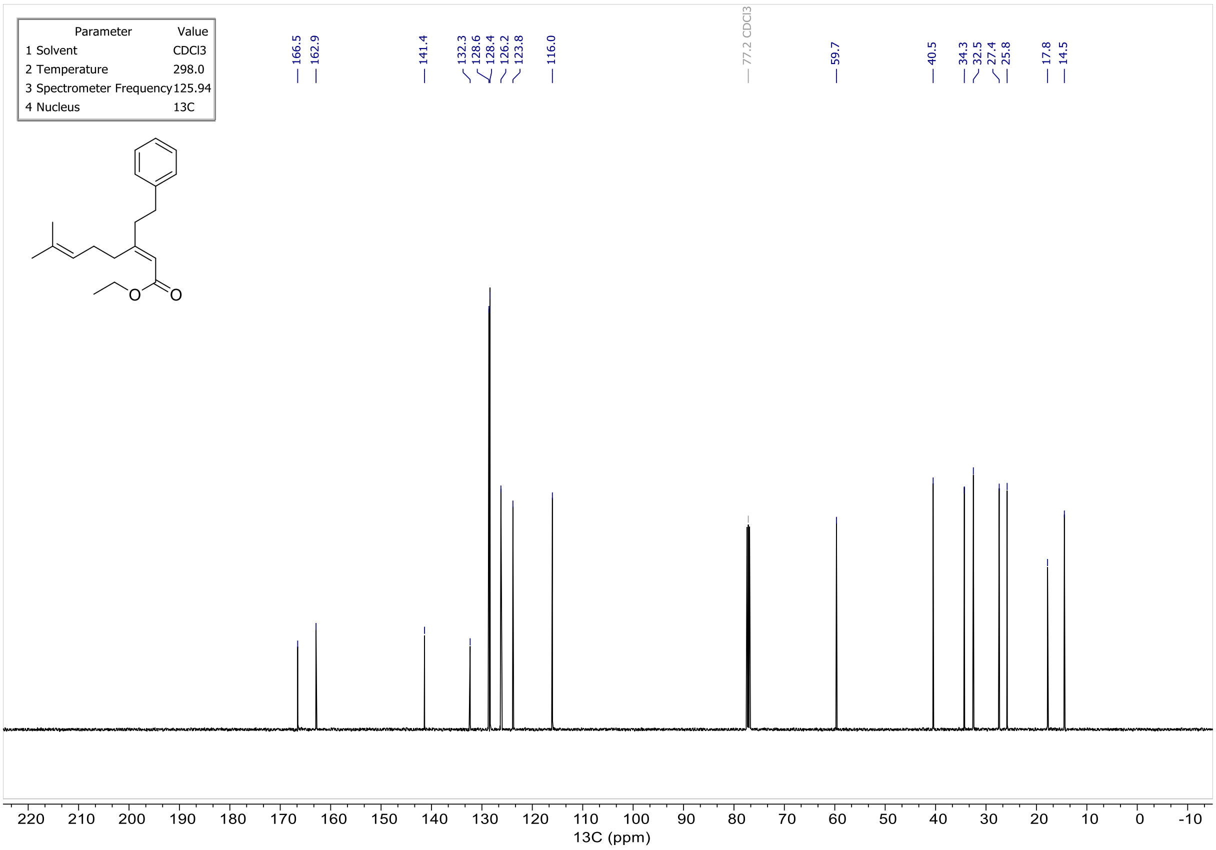

ethyl (*Z*)-7-methyl-3-(5-(triethylsilyl)pent-4-yn-1-yl)octa-2,6-dienoate (**S32**)

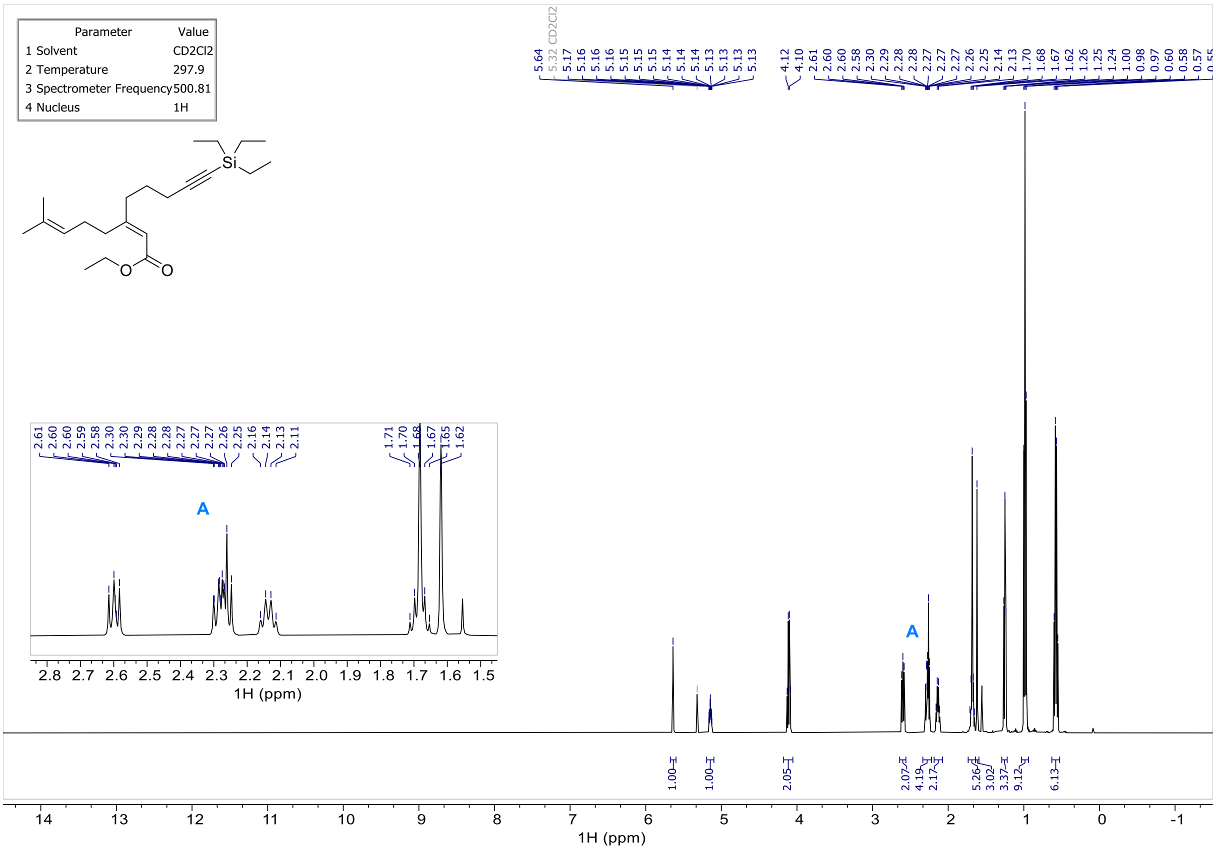

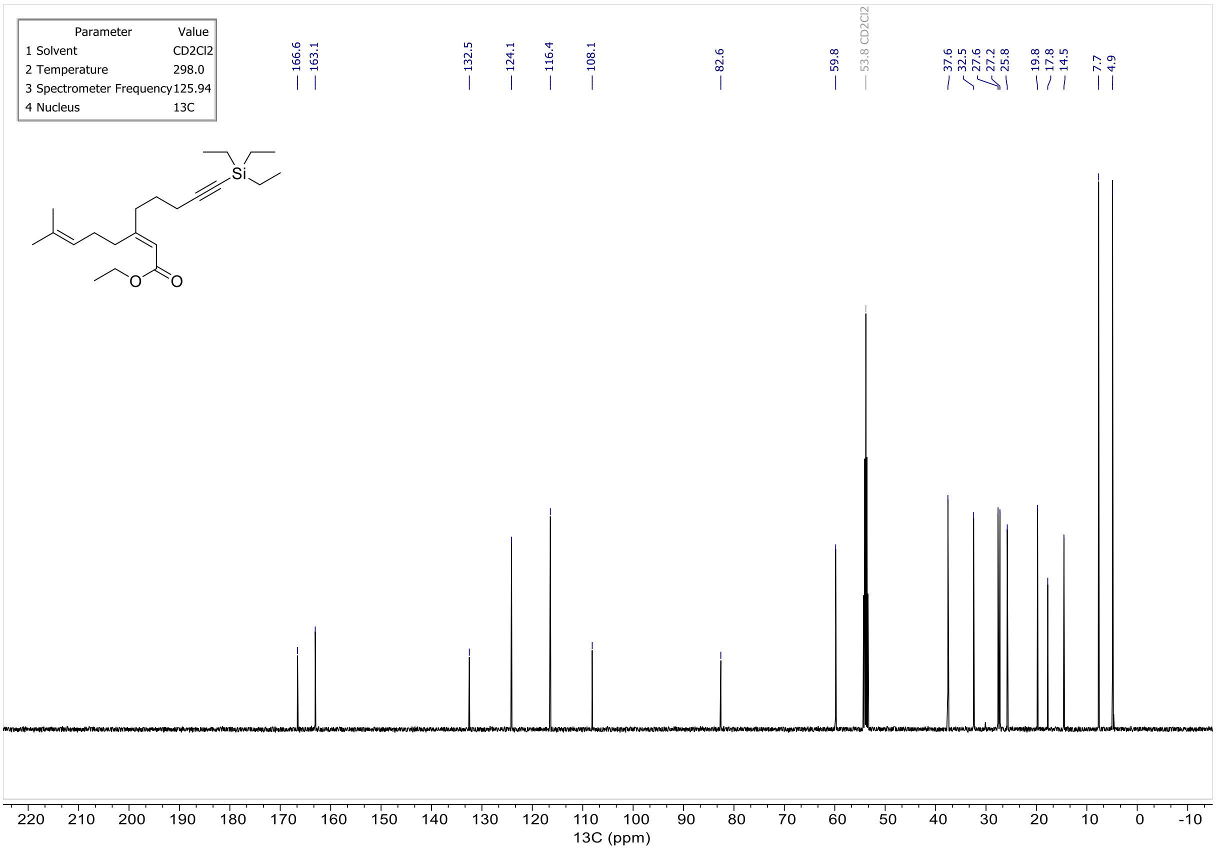

ethyl (*E*)-3-cyclopentyl-7-methylocta-2,6-dienoate (**S33**)

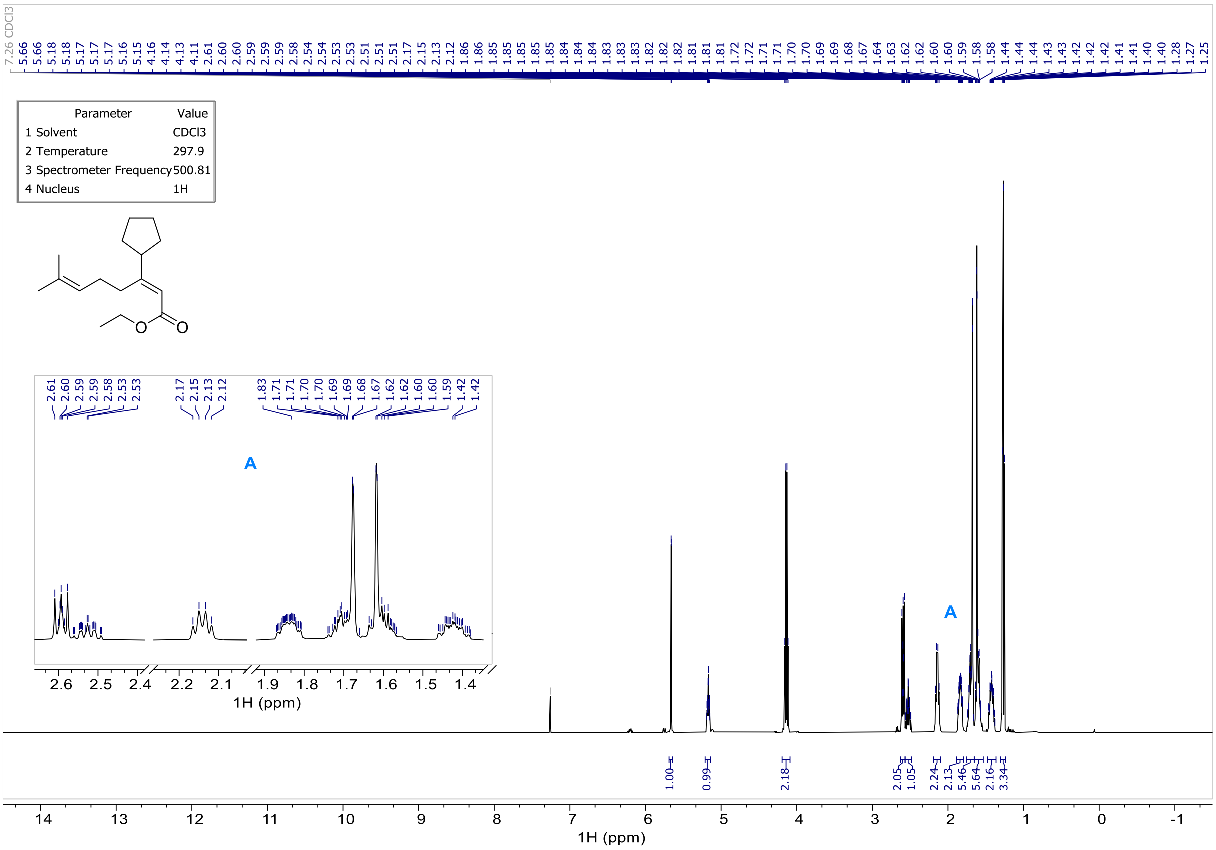

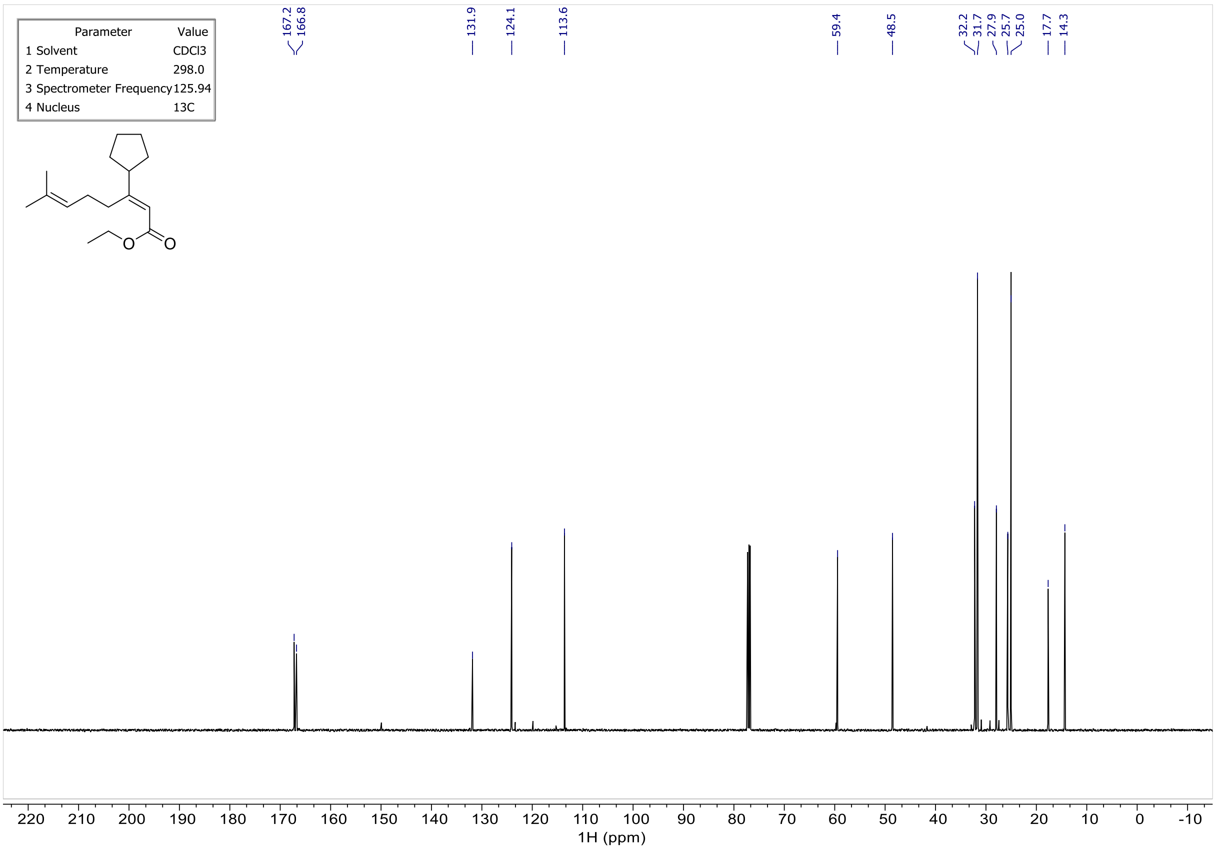

(*Z*)-3-ethyl-7-methylocta-2,6-dien-1-ol (**S34**)

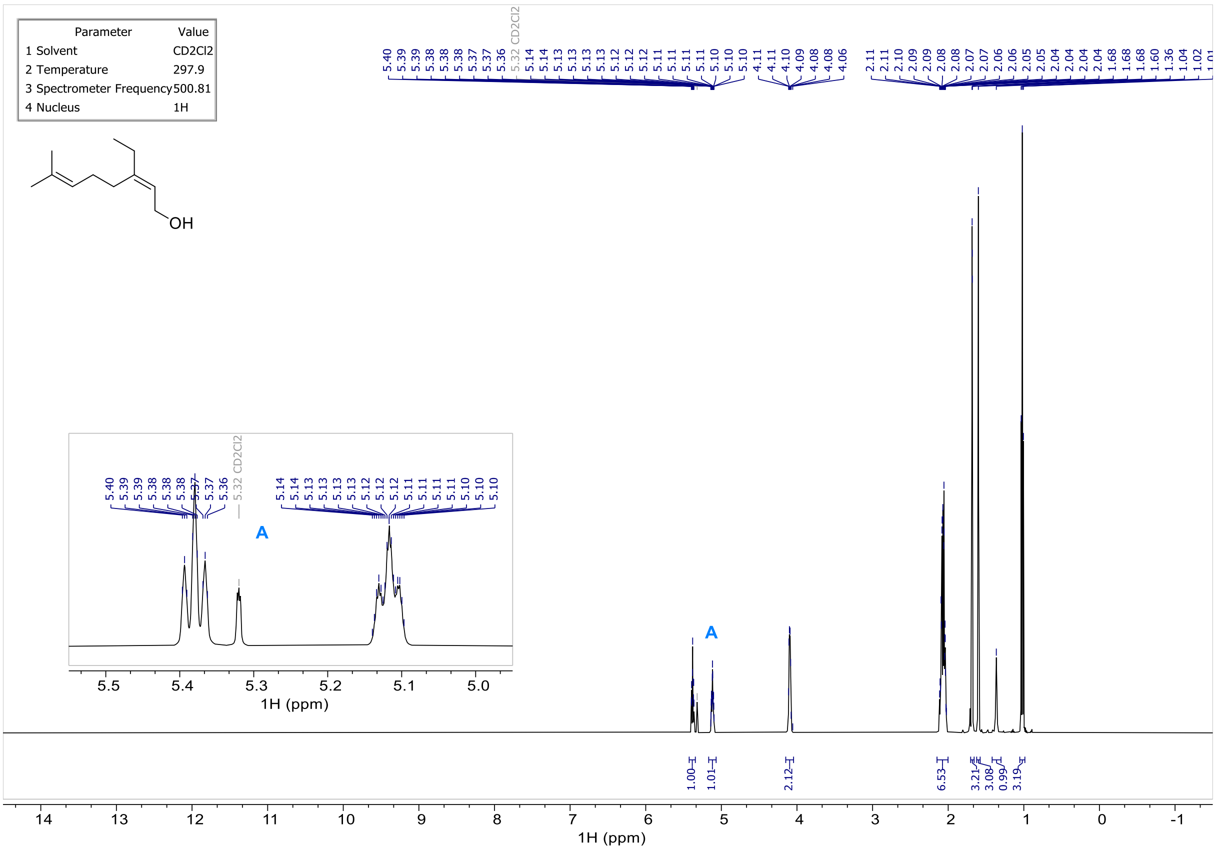

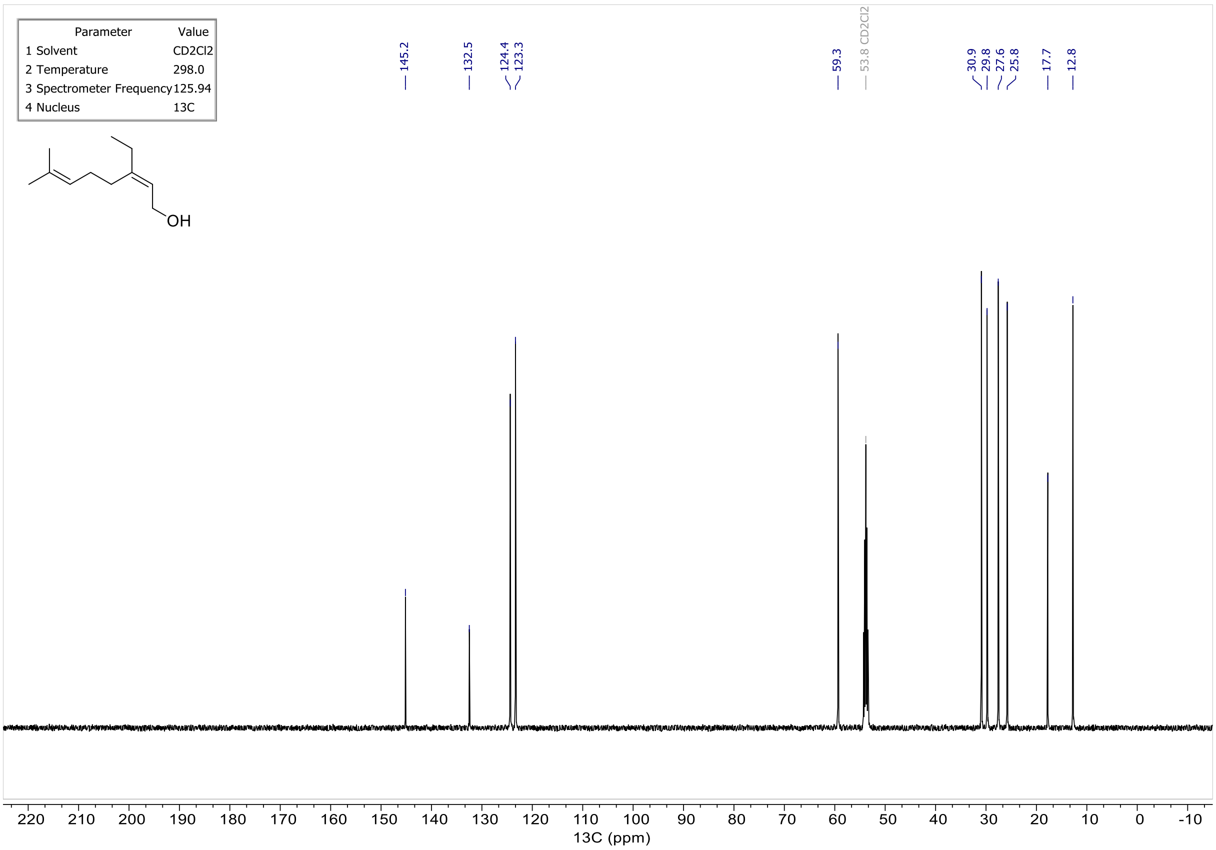

(*Z*)-7-methyl-3-propylocta-2,6-dien-1-ol (**S35**)

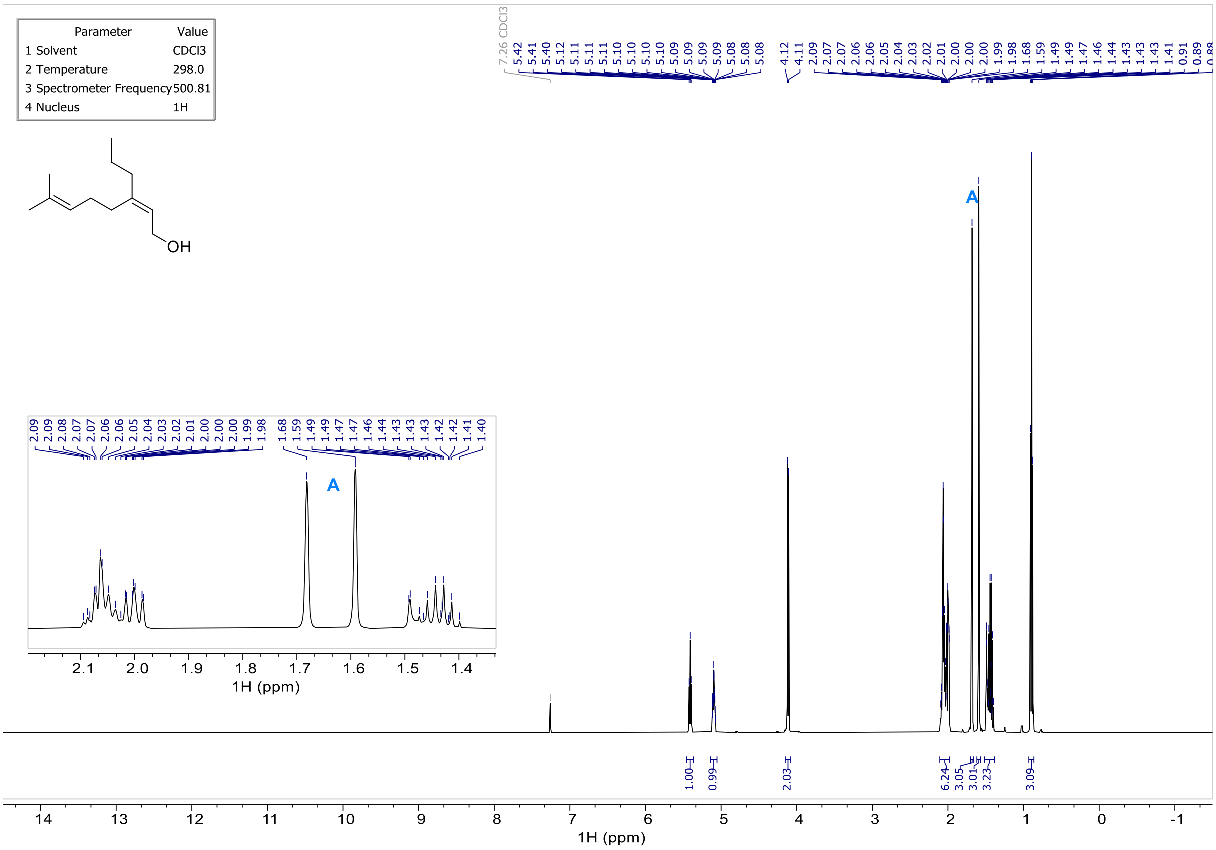

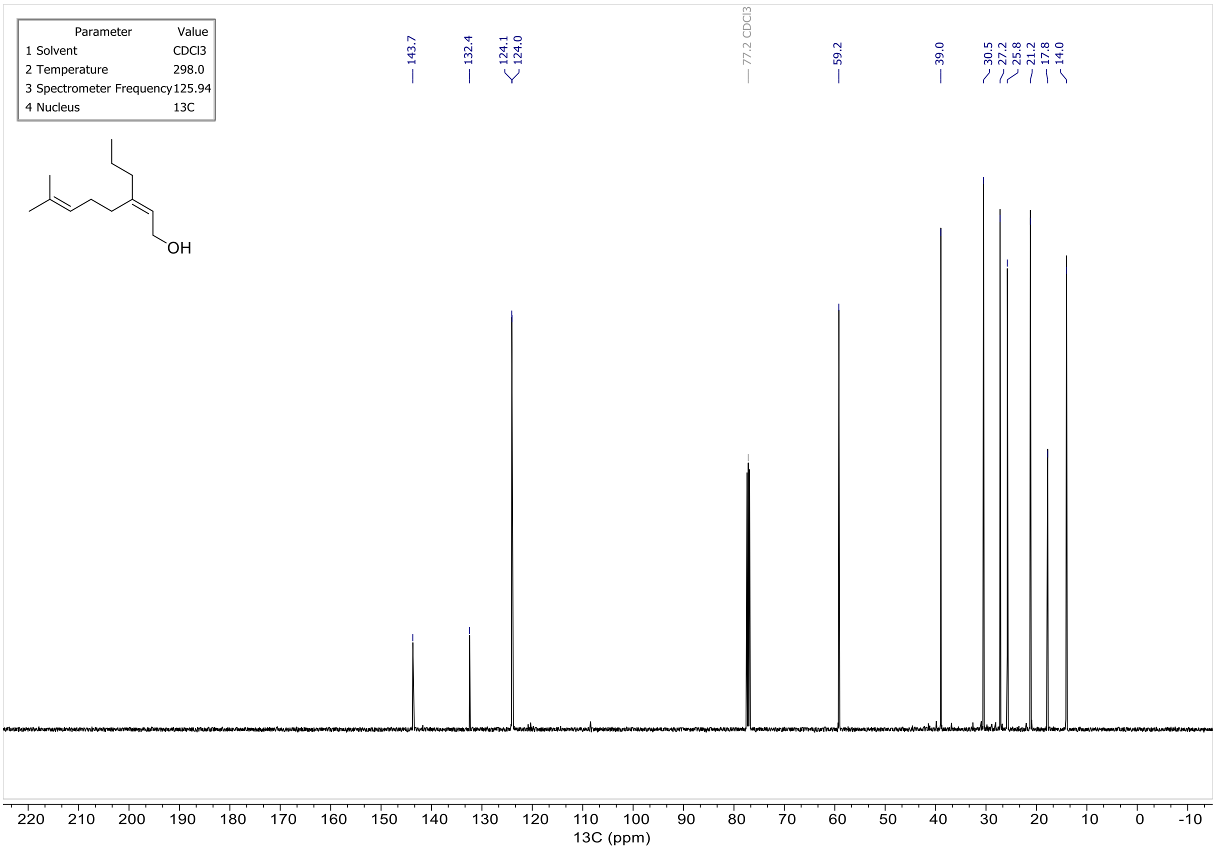

(*E*)-3-isobutyl-7-methylocta-2,6-dien-1-ol (**S36**)

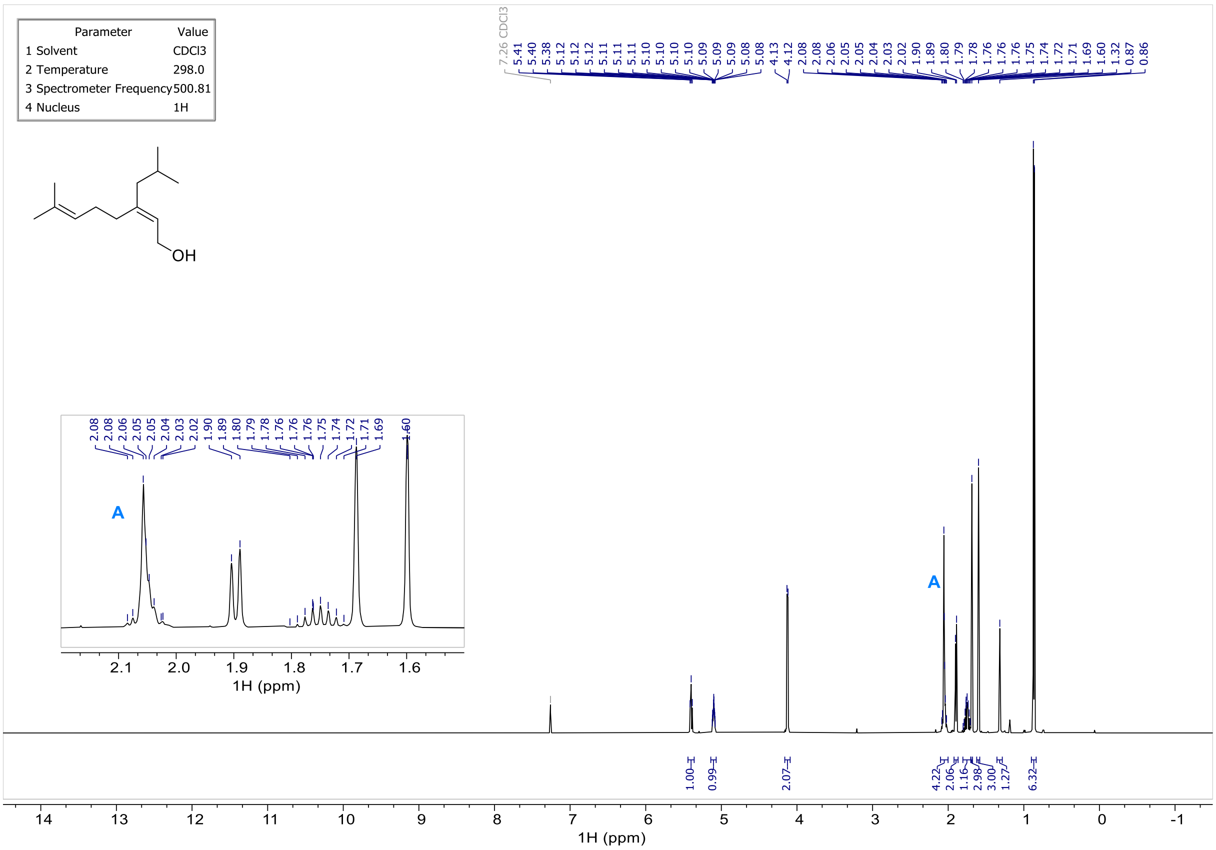

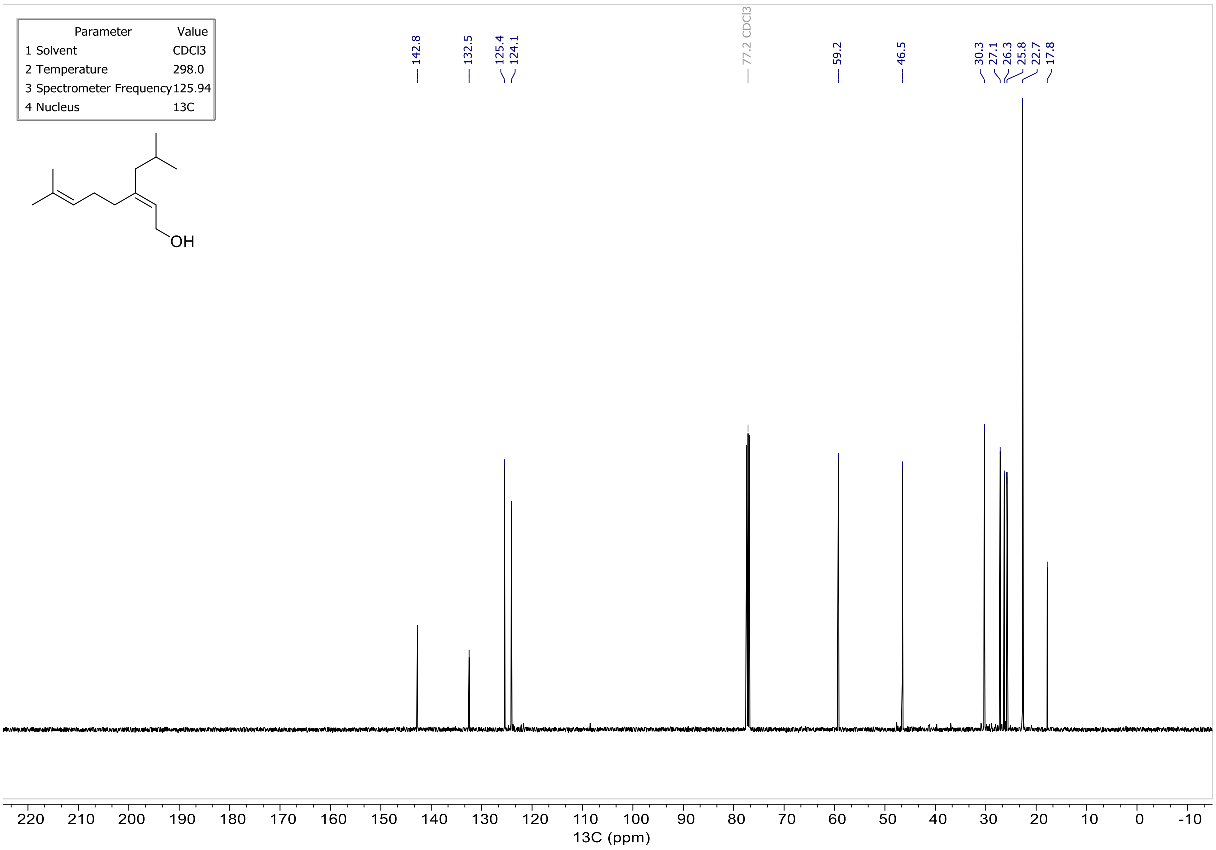

(*E*)-3-benzyl-7-methylocta-2,6-dien-1-ol (**S37**)

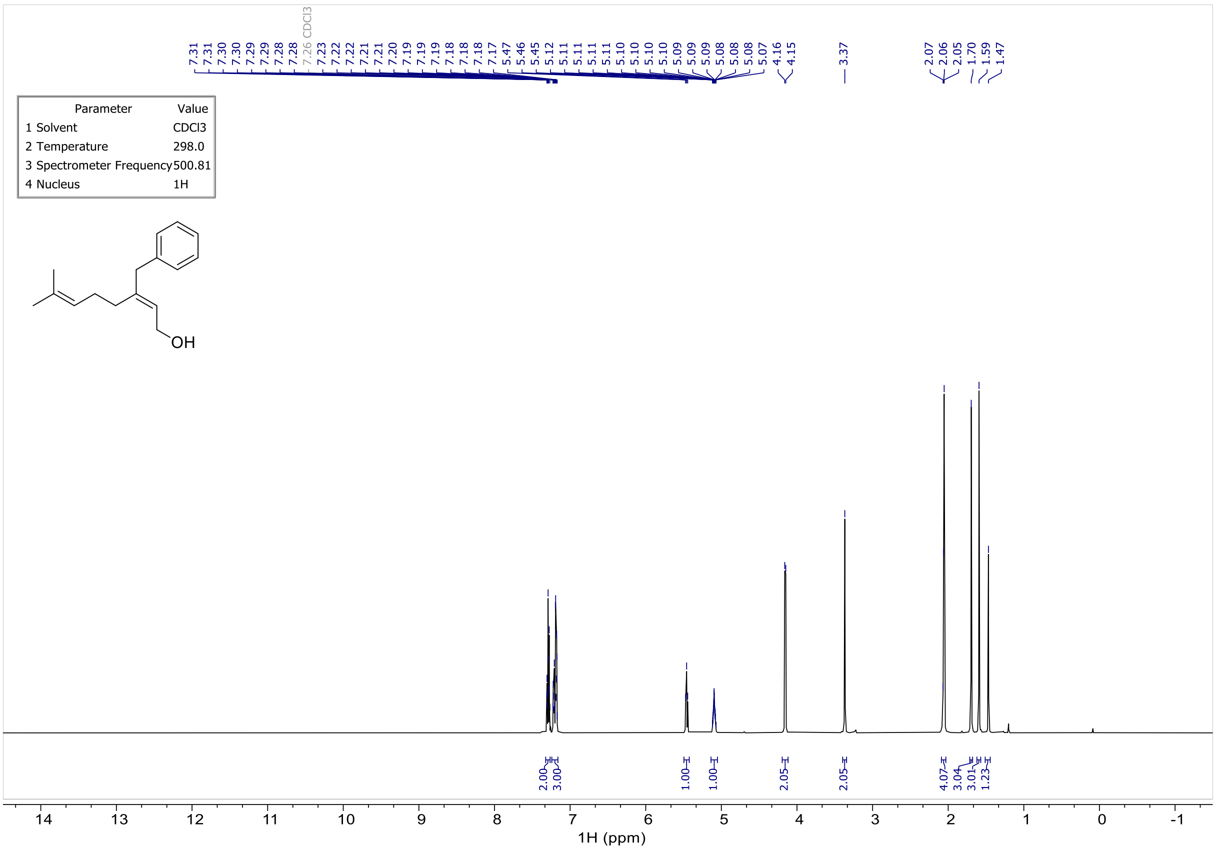

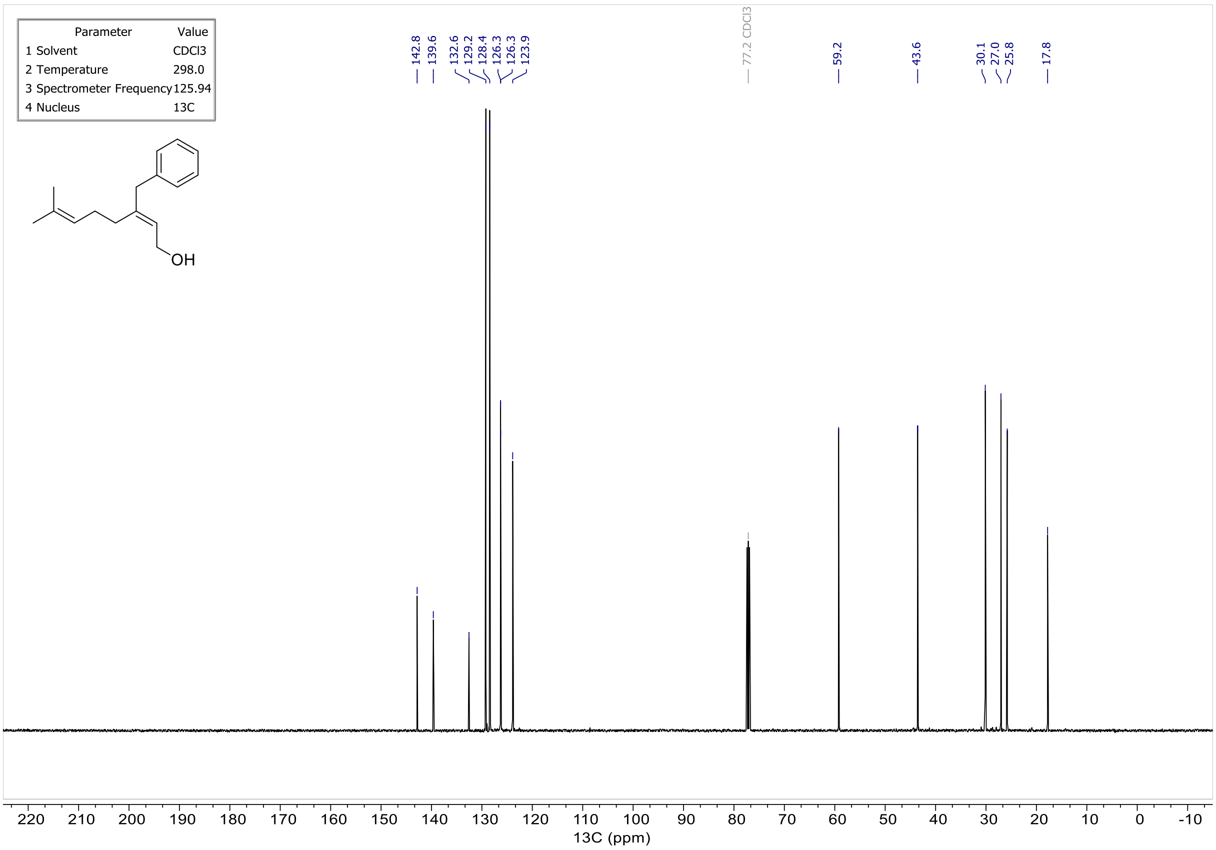

(*E*)-7-methyl-3-phenethylocta-2,6-dien-1-ol (**S38**)

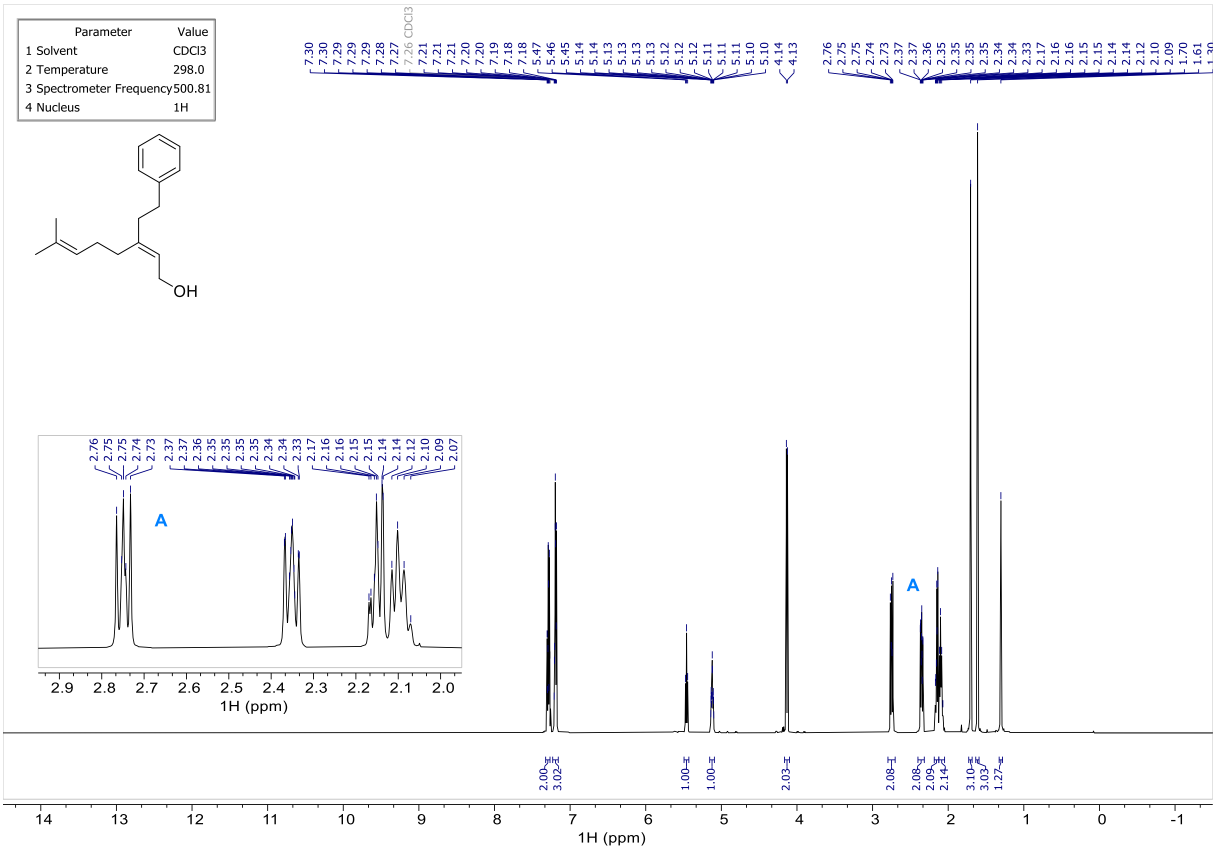

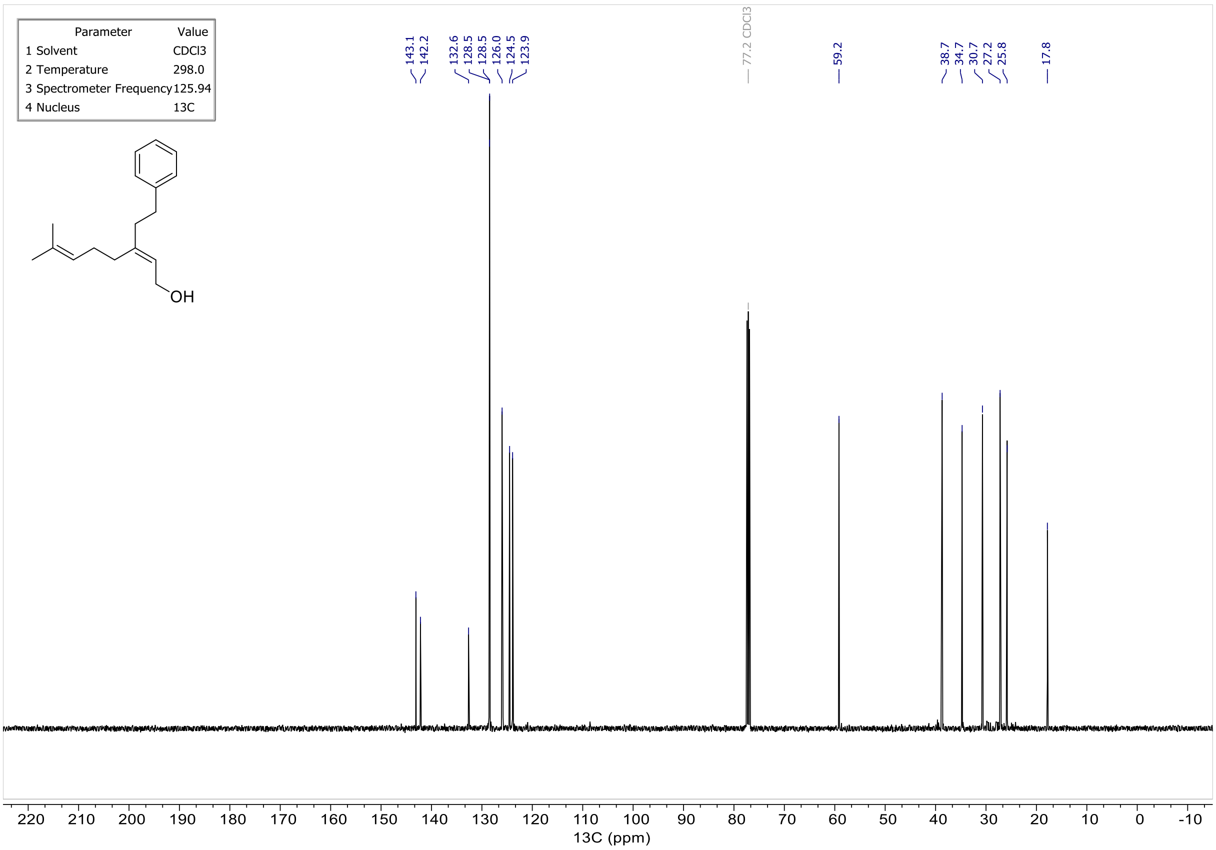

(*Z*)-7-methyl-3-(5-(triethylsilyl)pent-4-yn-1-yl)octa-2,6-dien-1-ol (**S39**)

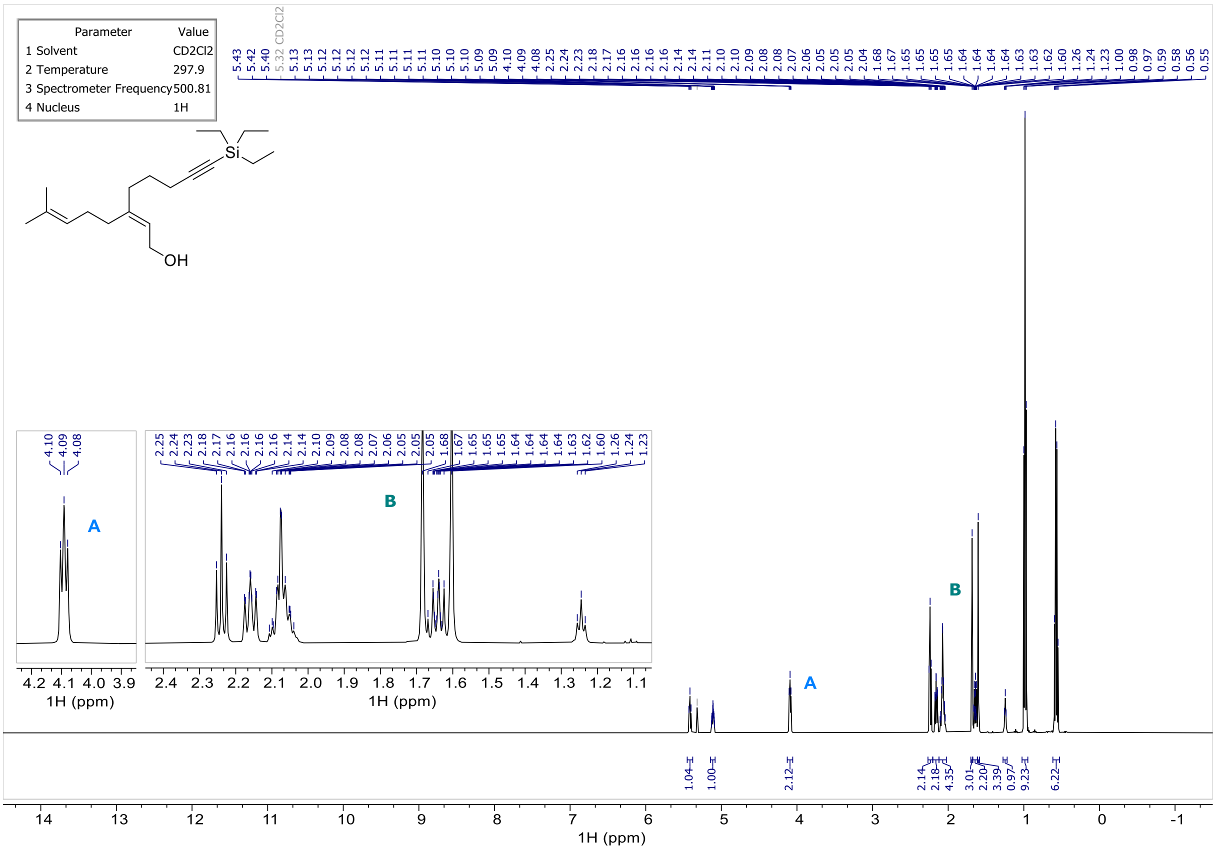

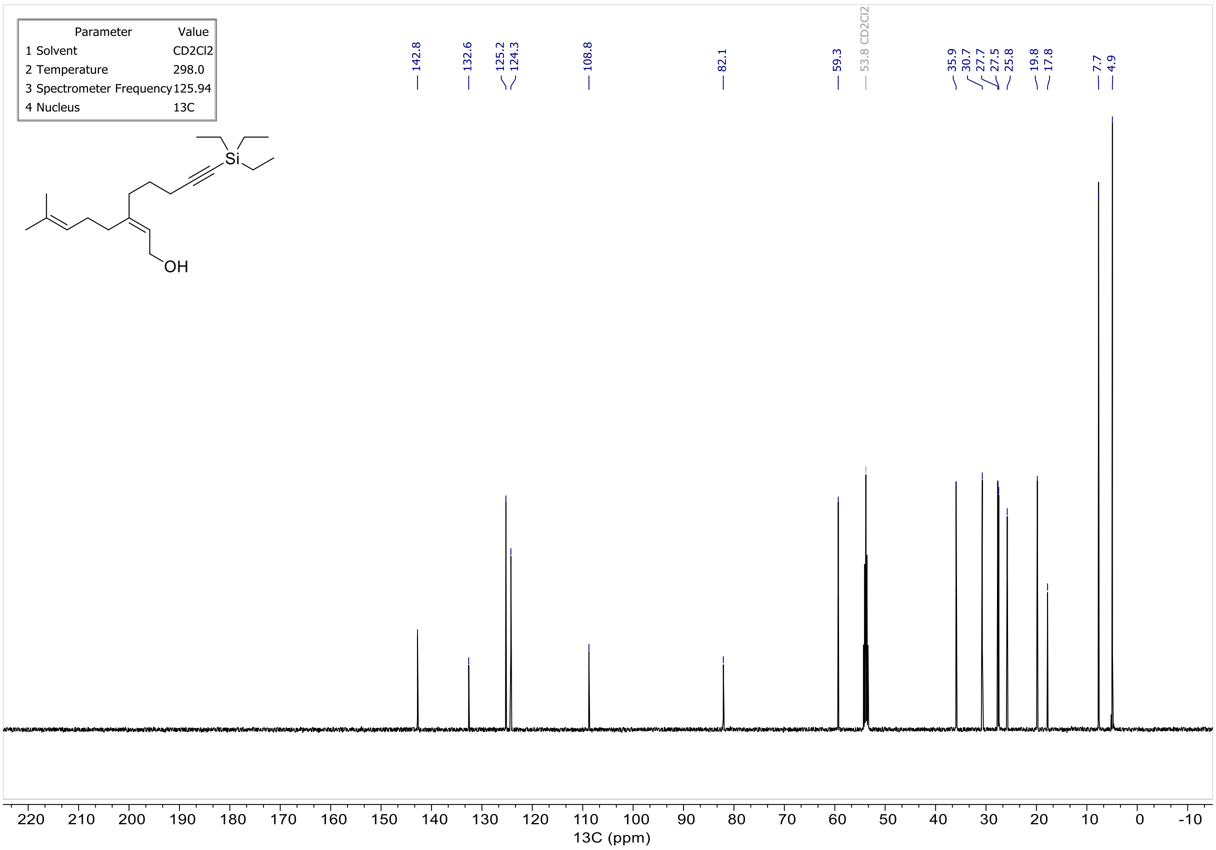

(*E*)-3-cyclopentyl-7-methylocta-2,6-dien-1-ol (**S40**)

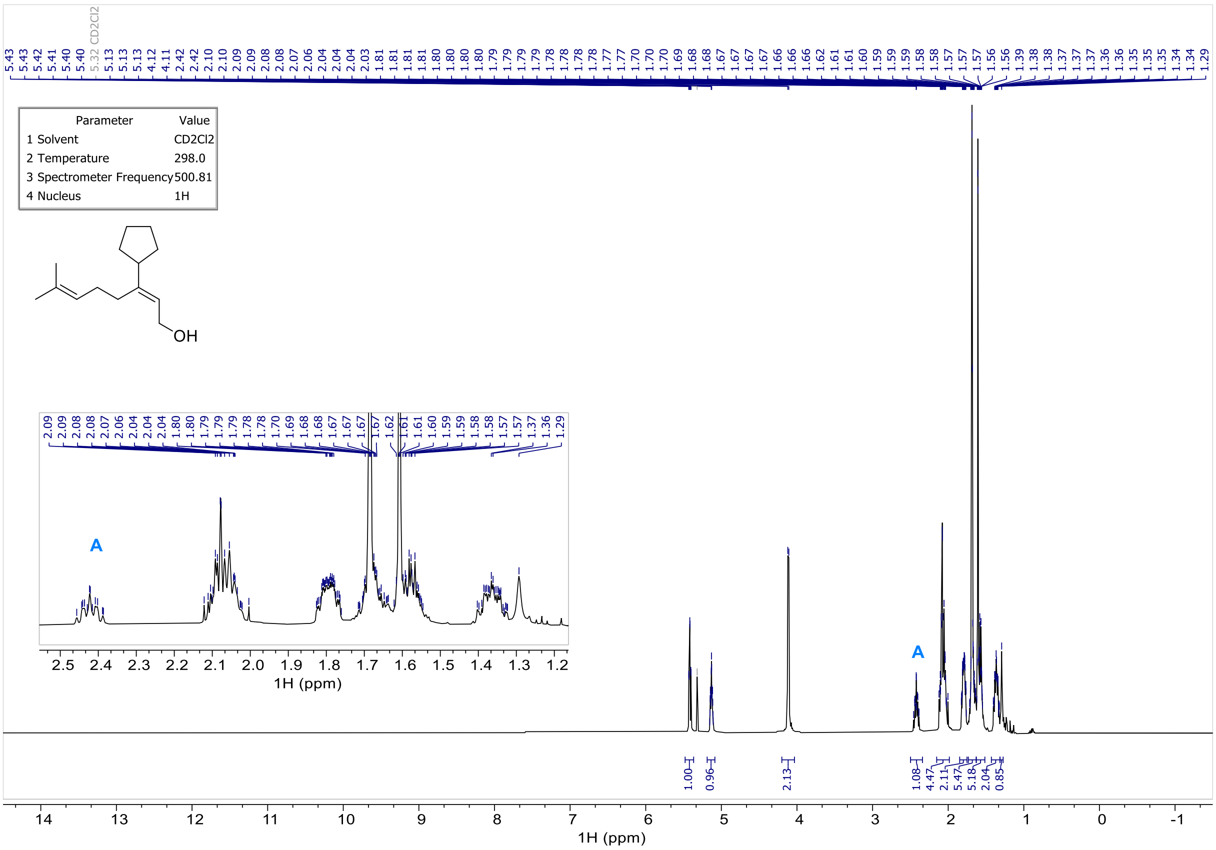

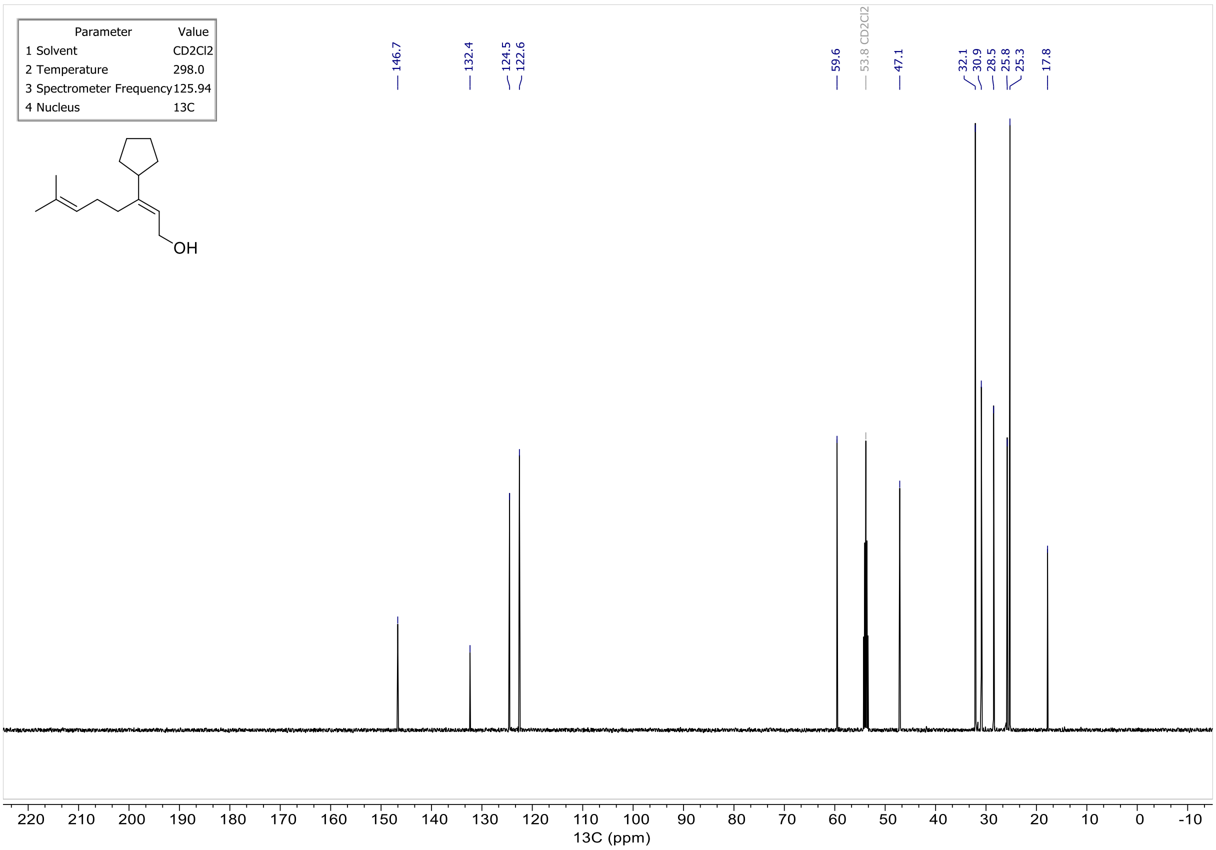

(*Z*)-3-ethyl-7-methylocta-2,6-dienal (**S19**)

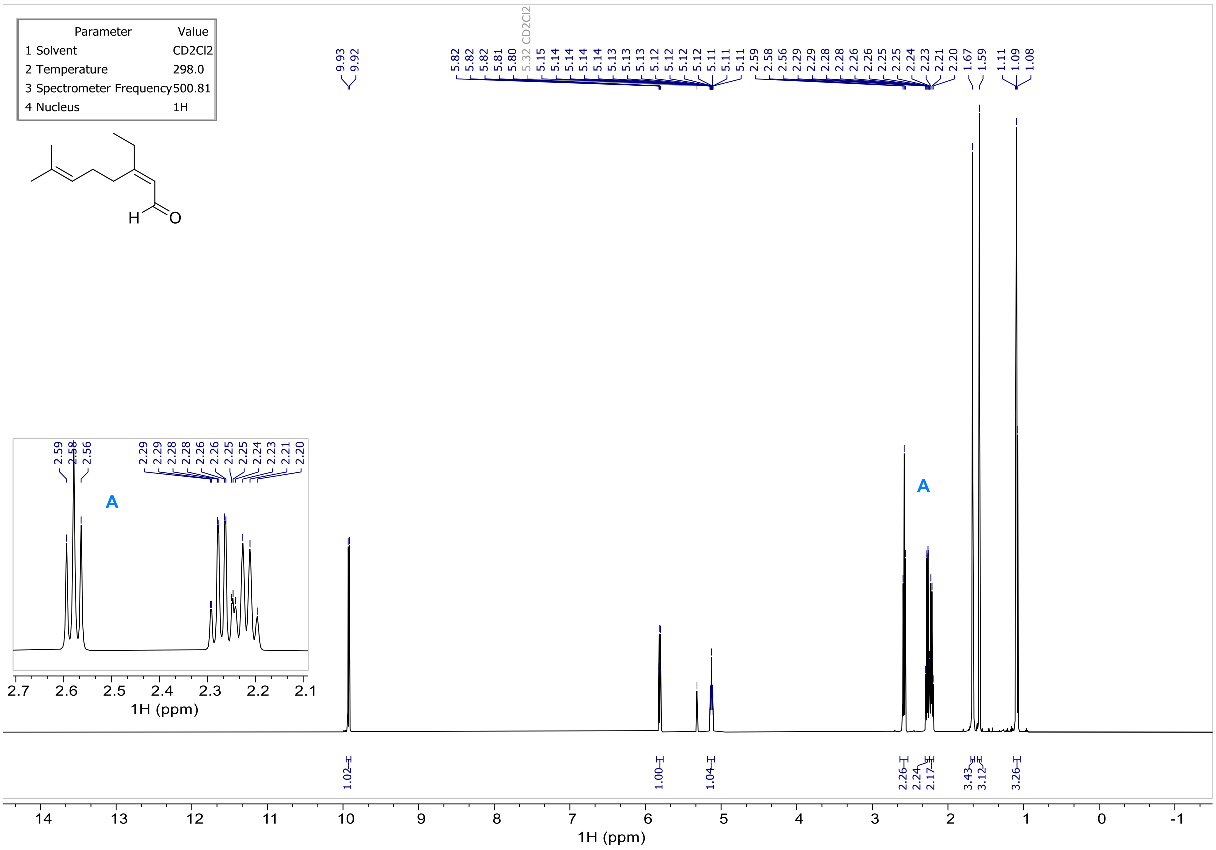

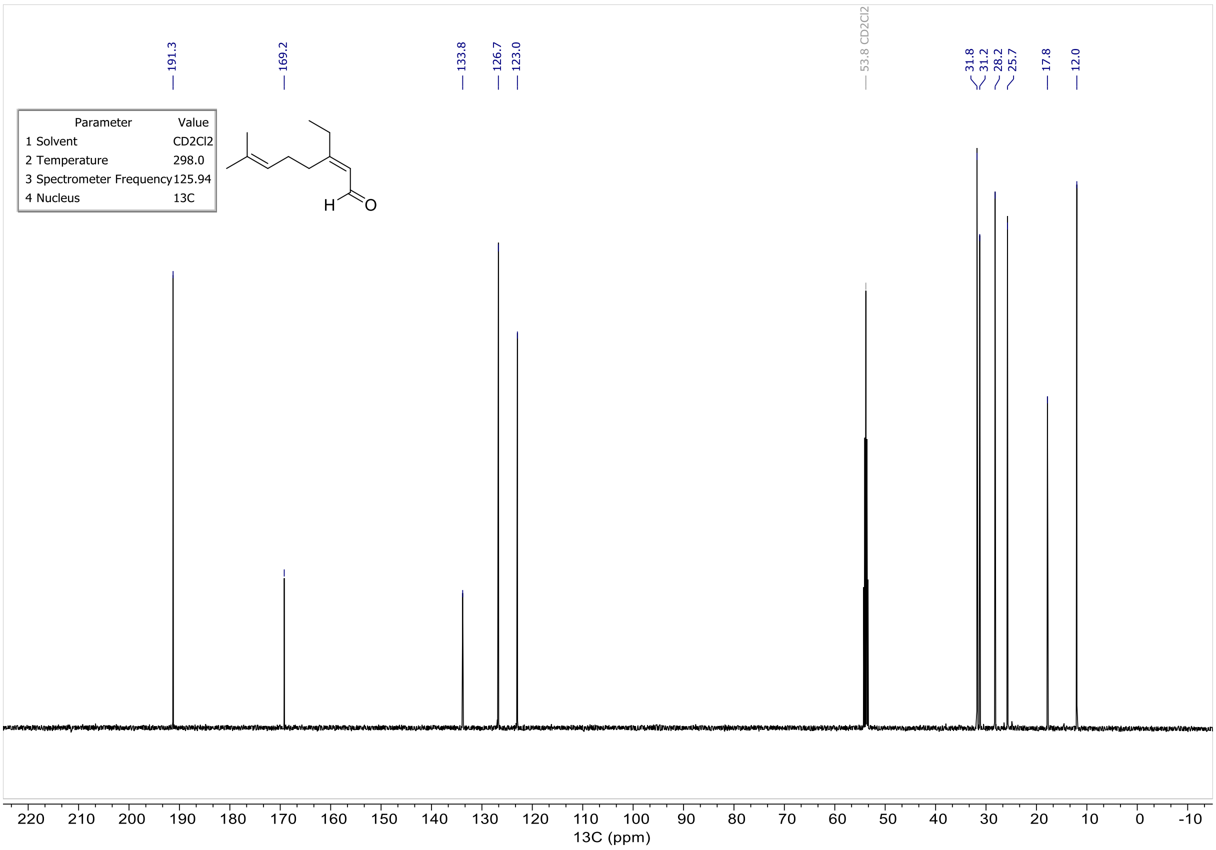

(*Z*)-7-methyl-3-propylocta-2,6-dienal (**S20**)

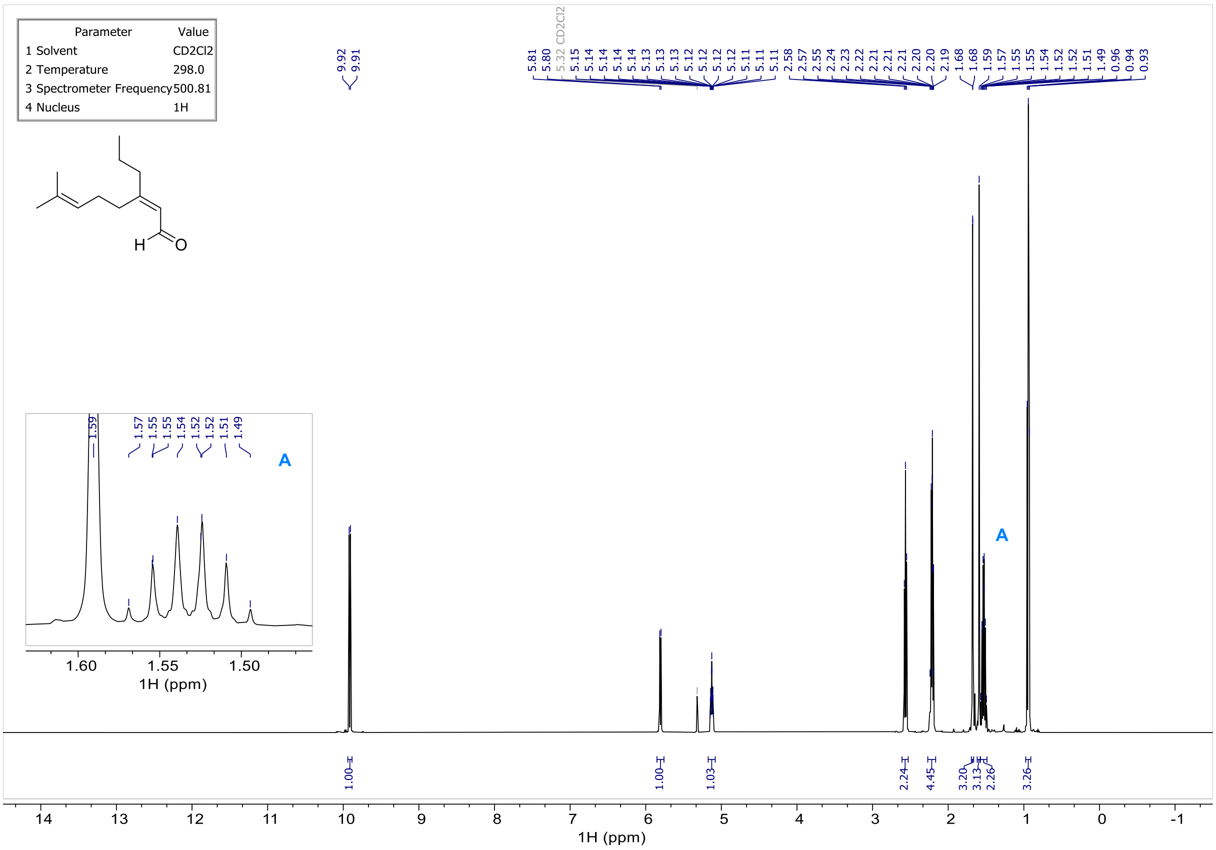

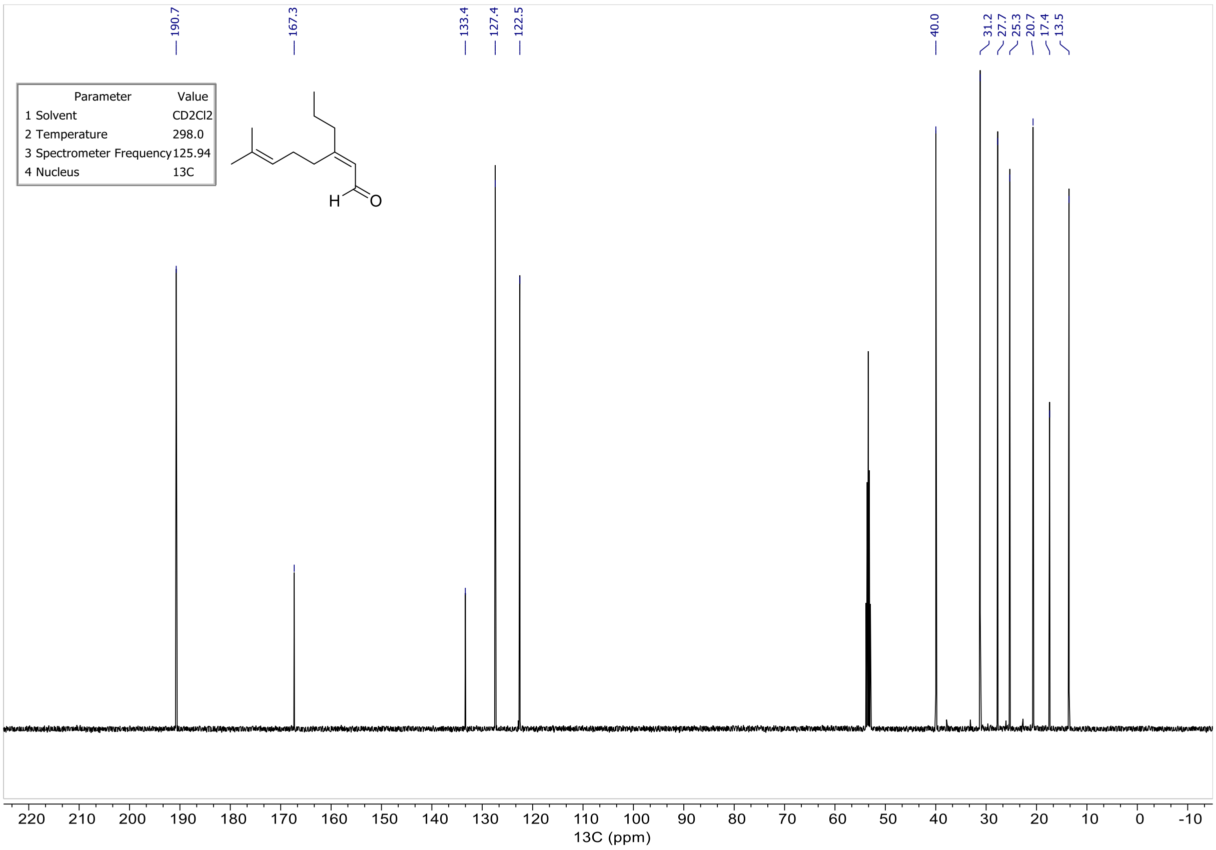

(*E*)-3-isobutyl-7-methylocta-2,6-dienal (**S21**)

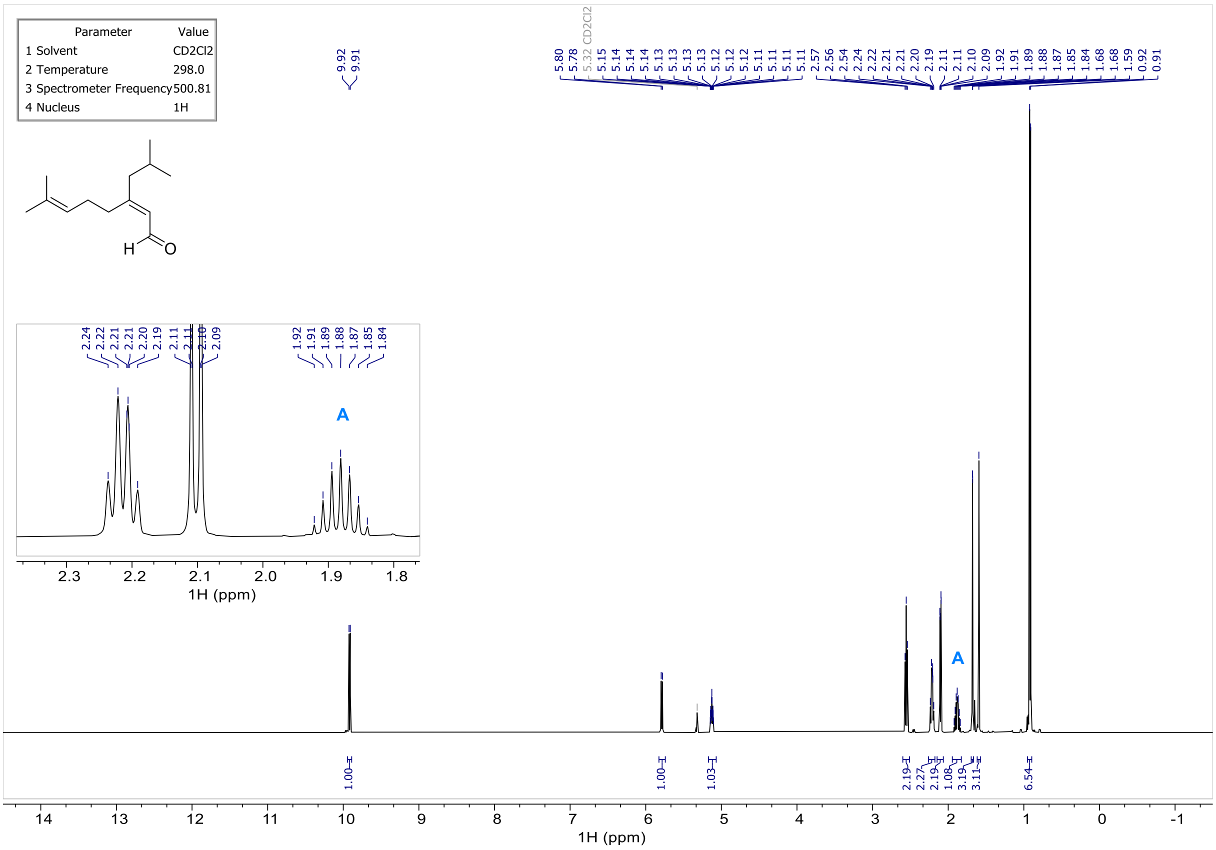

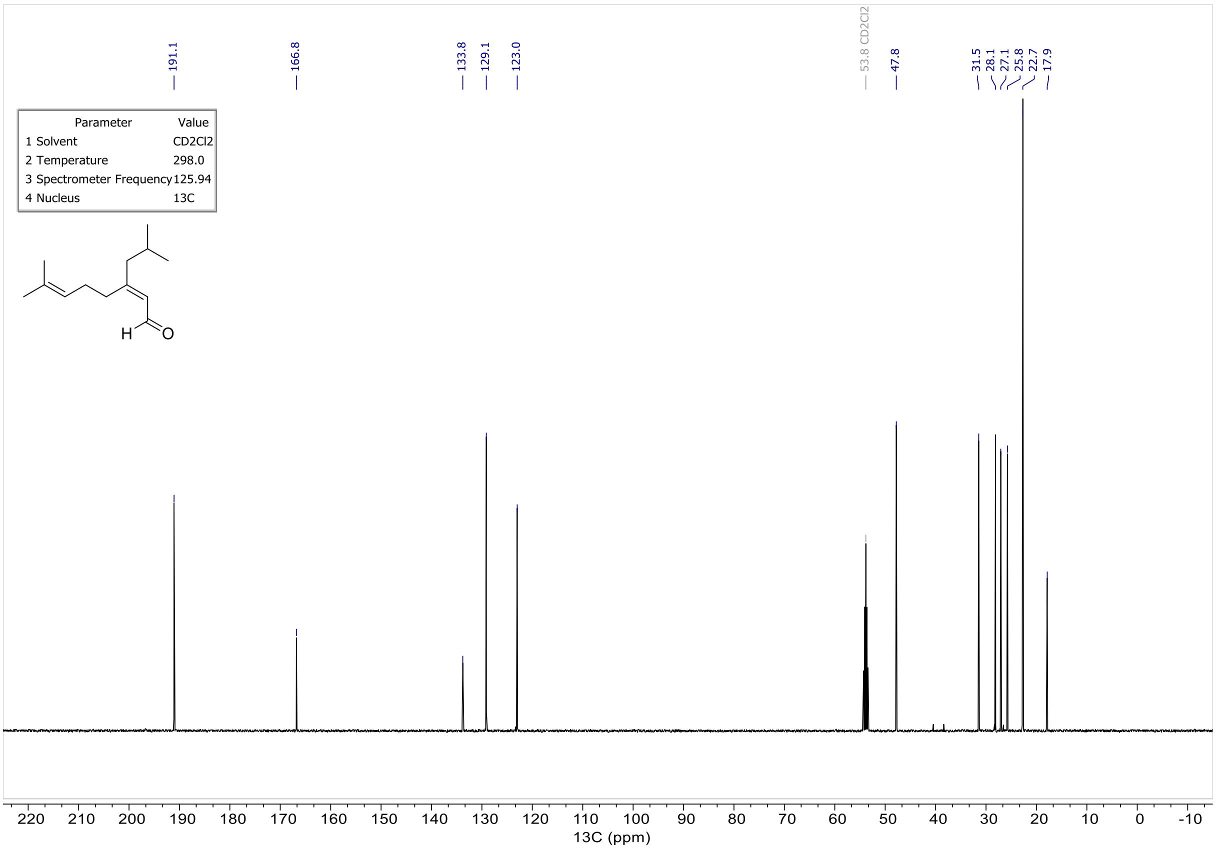

(*E*)-3-benzyl-7-methylocta-2,6-dienal (**S22**)

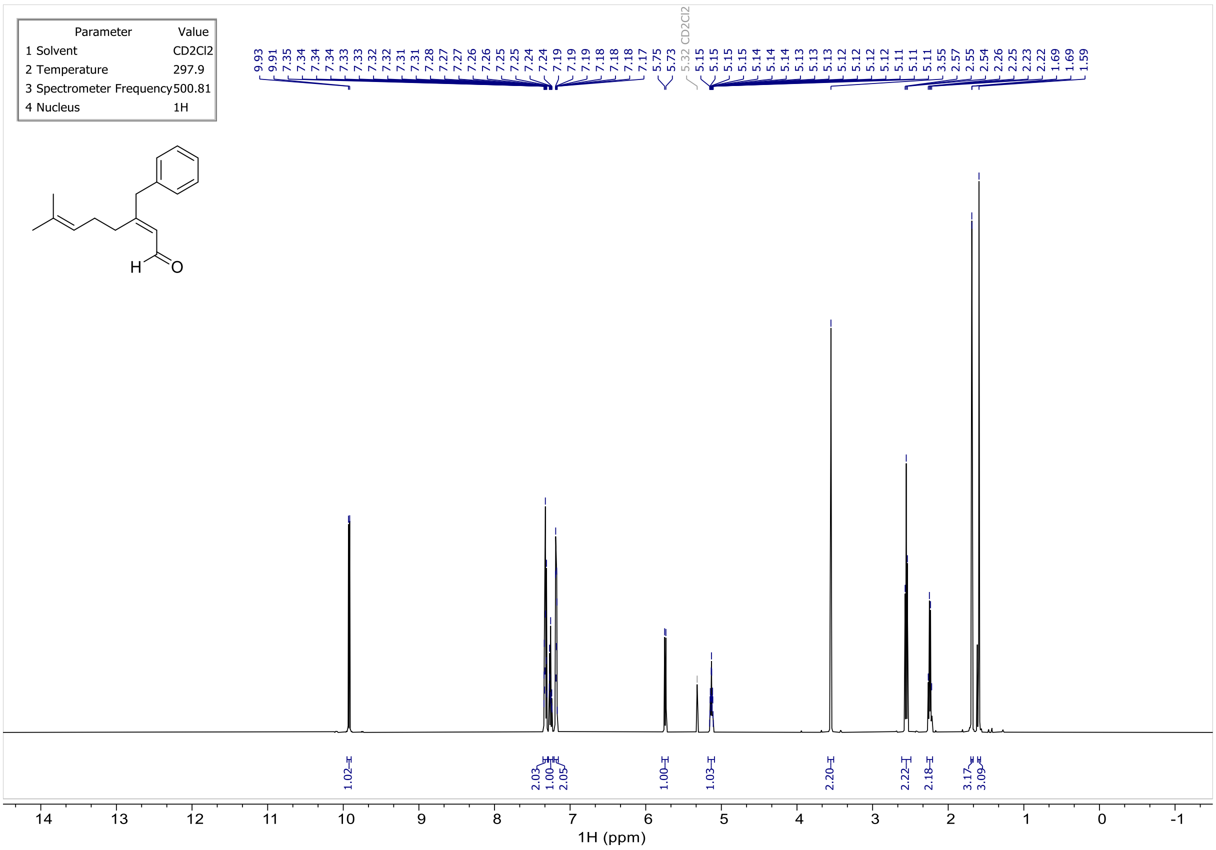

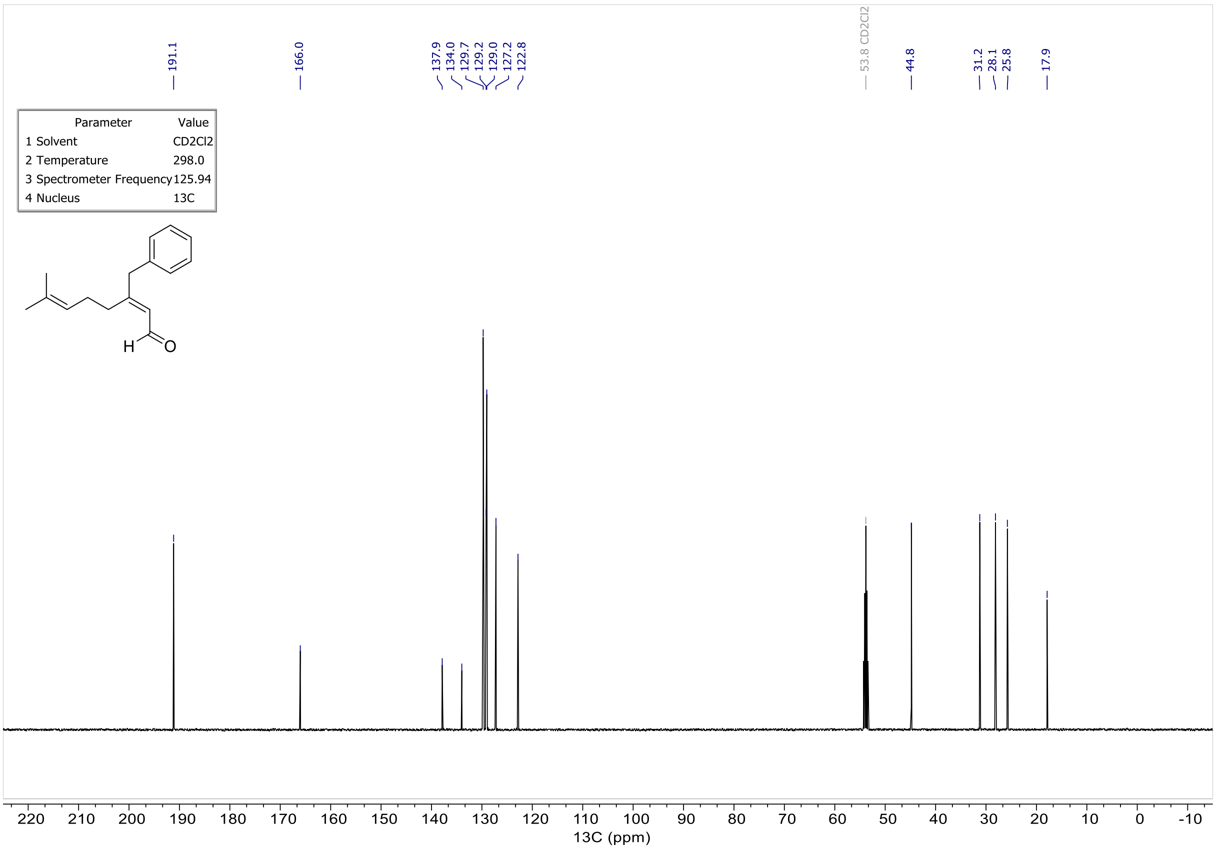

(*E*)-7-methyl-3-phenethylocta-2,6-dienal (**S23**)

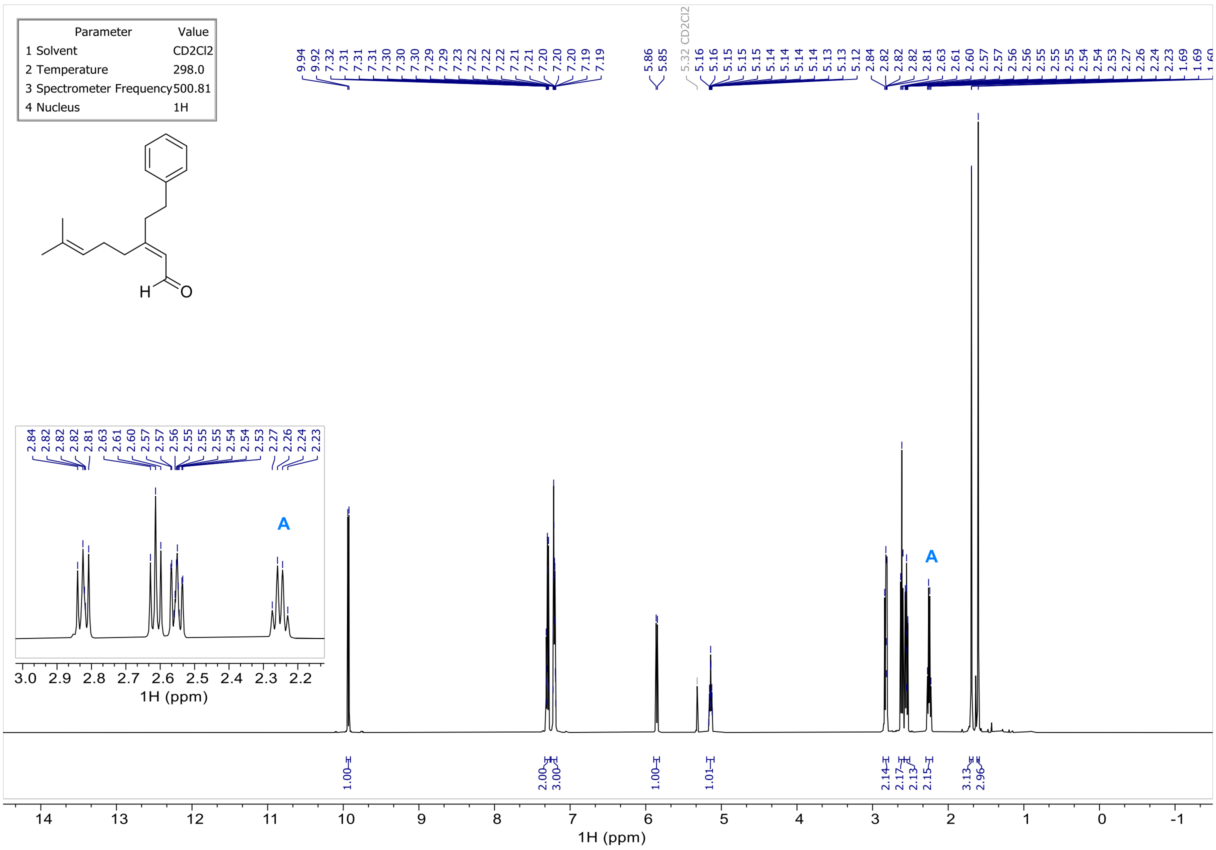

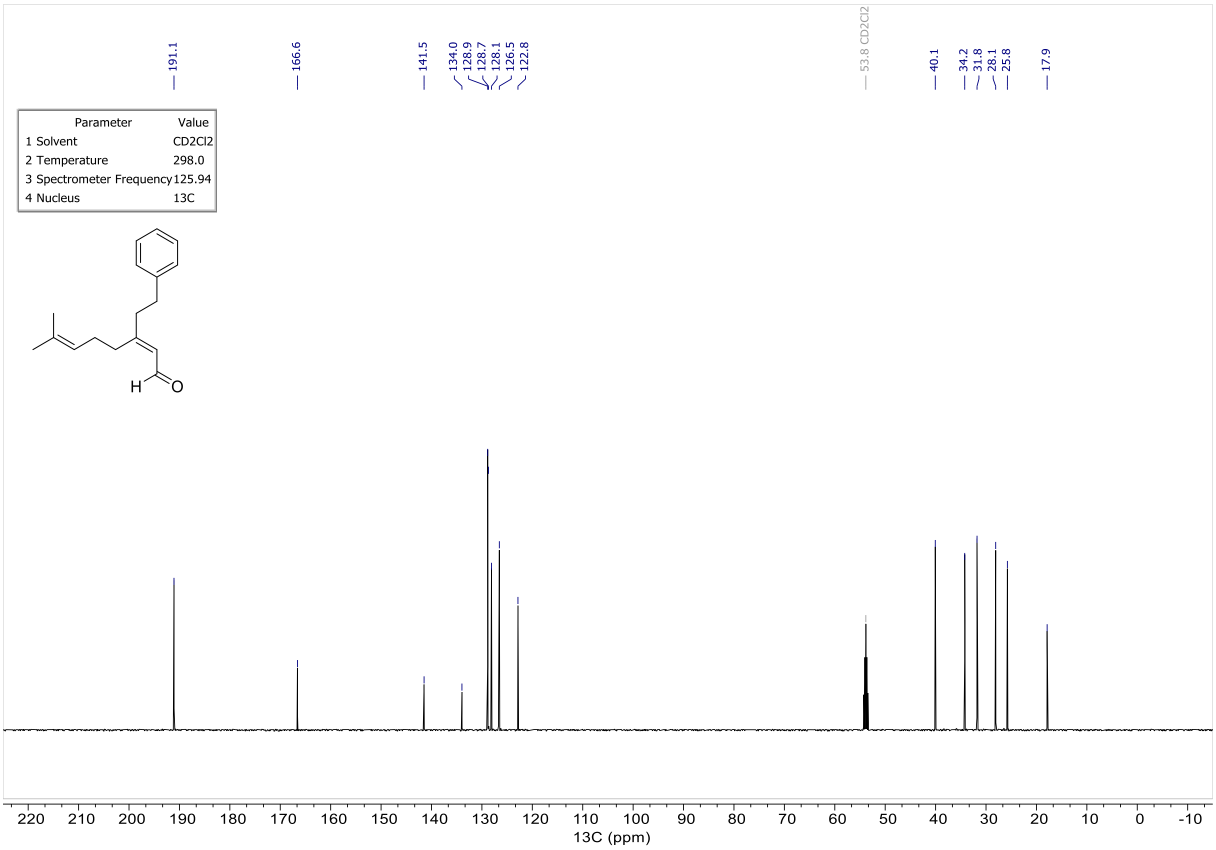

(*Z*)-7-methyl-3-(5-(triethylsilyl)pent-4-yn-1-yl)octa-2,6-dienal (**S24**)

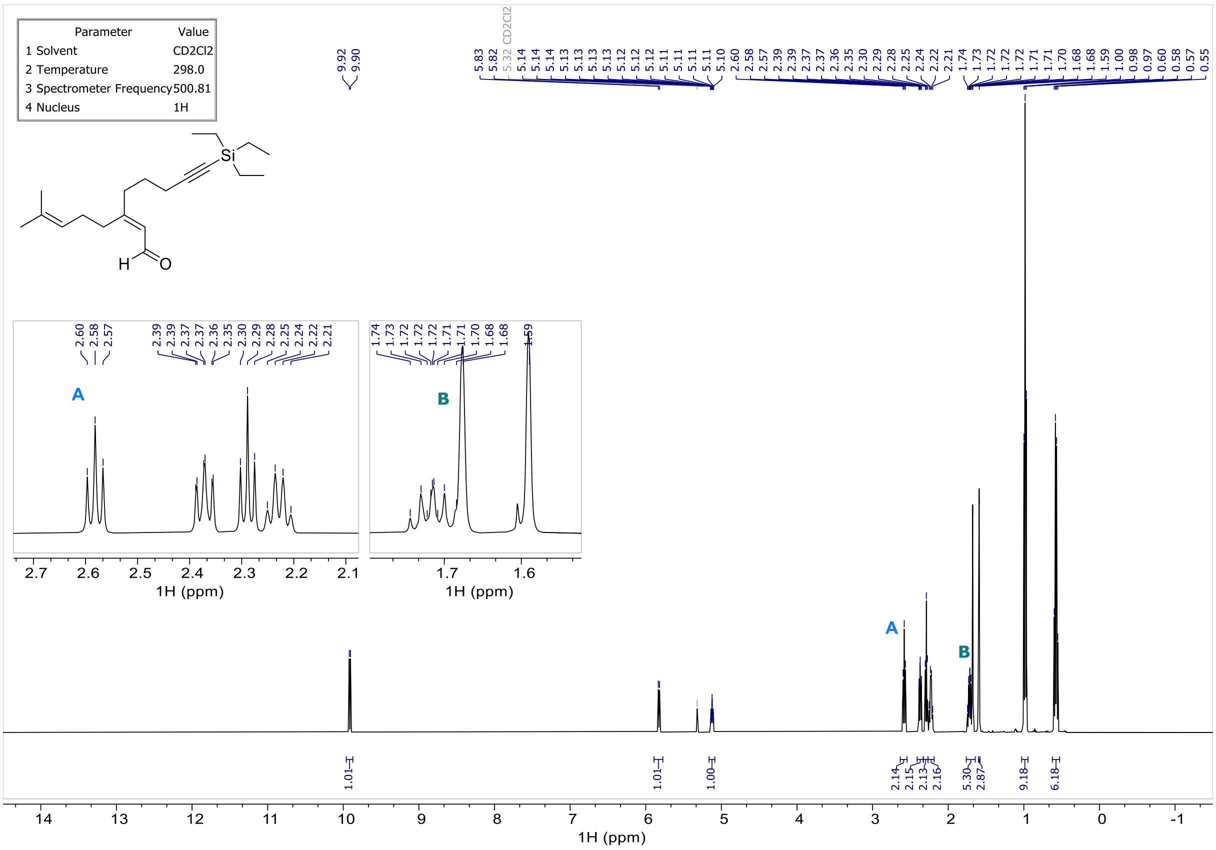

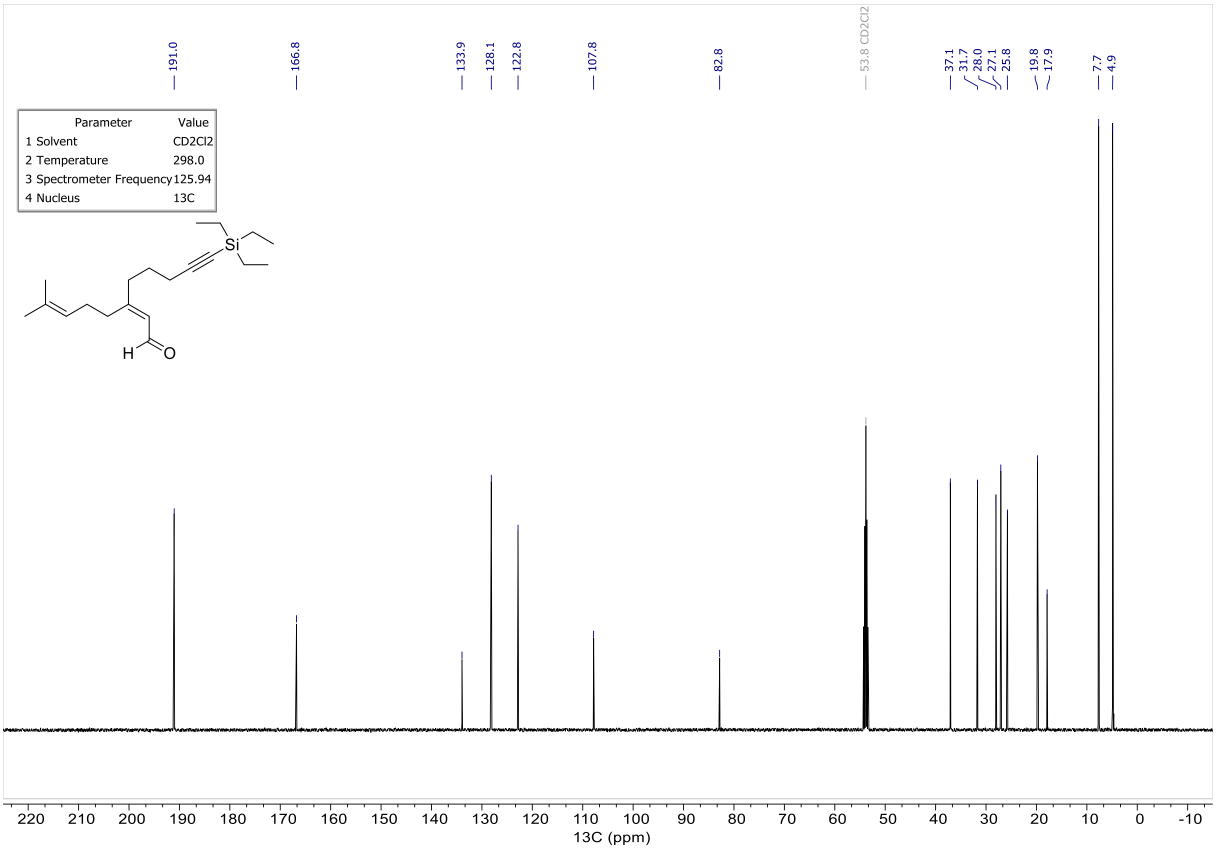

(*E*)-3-cyclopentyl-7-methylocta-2,6-dienal (**S25**)

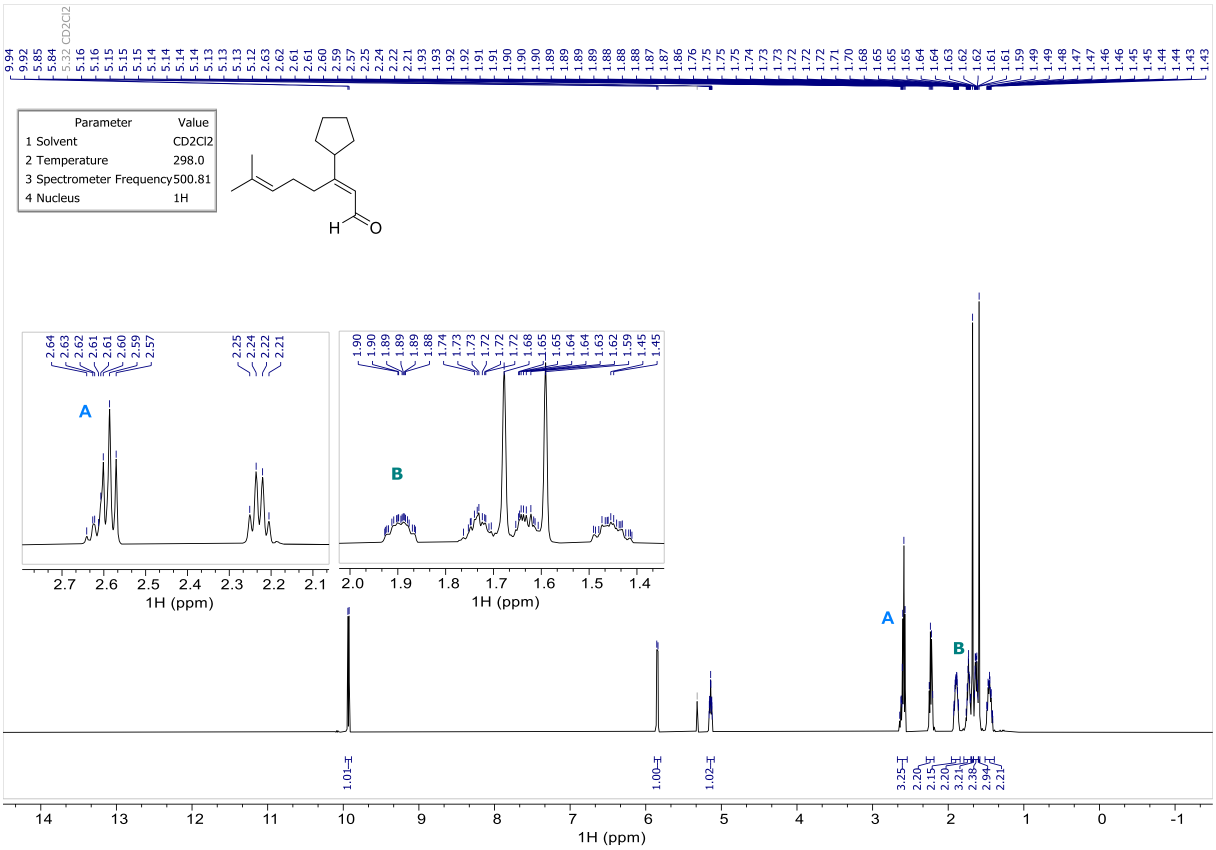

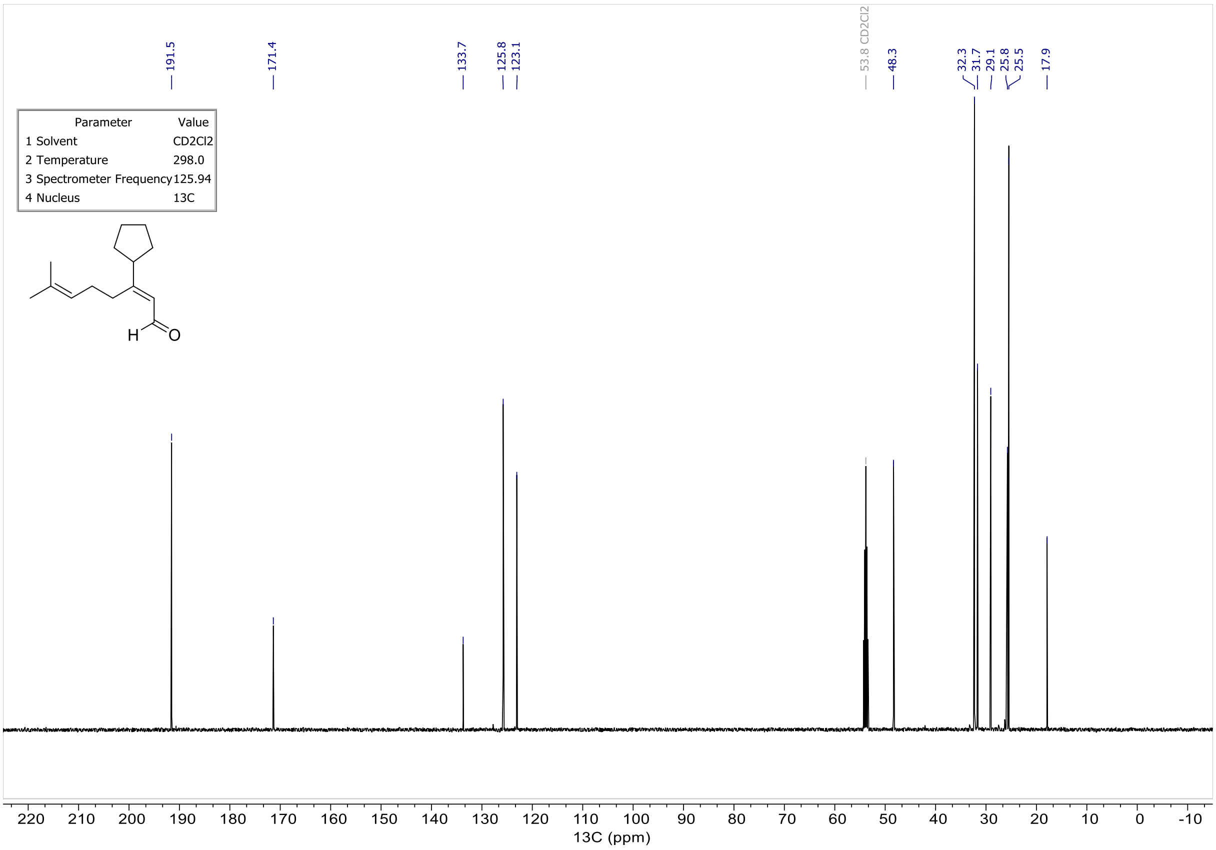

4,4,7-trimethyloct-6-en-2-ynal (**S42**)

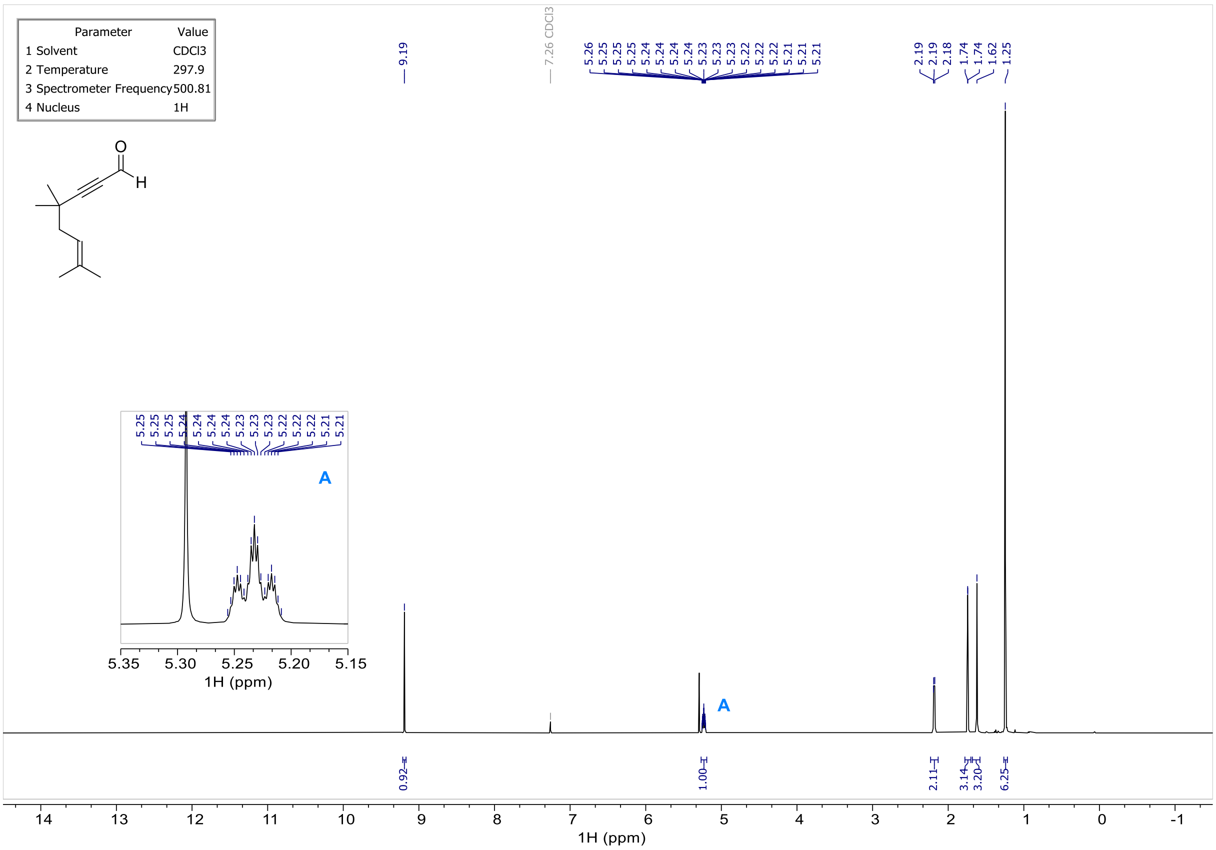

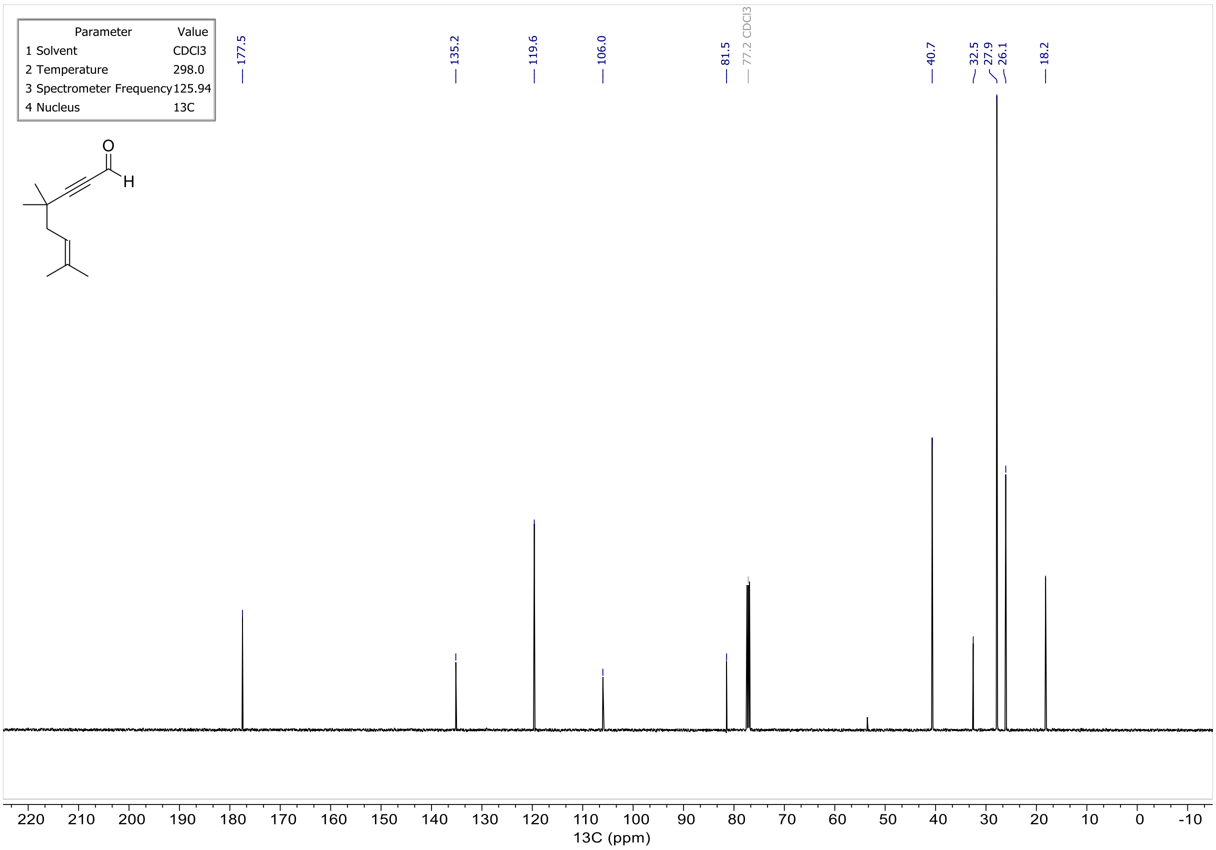

(*Z*)-4,4,7-trimethylocta-2,6-dienal (**S43**)

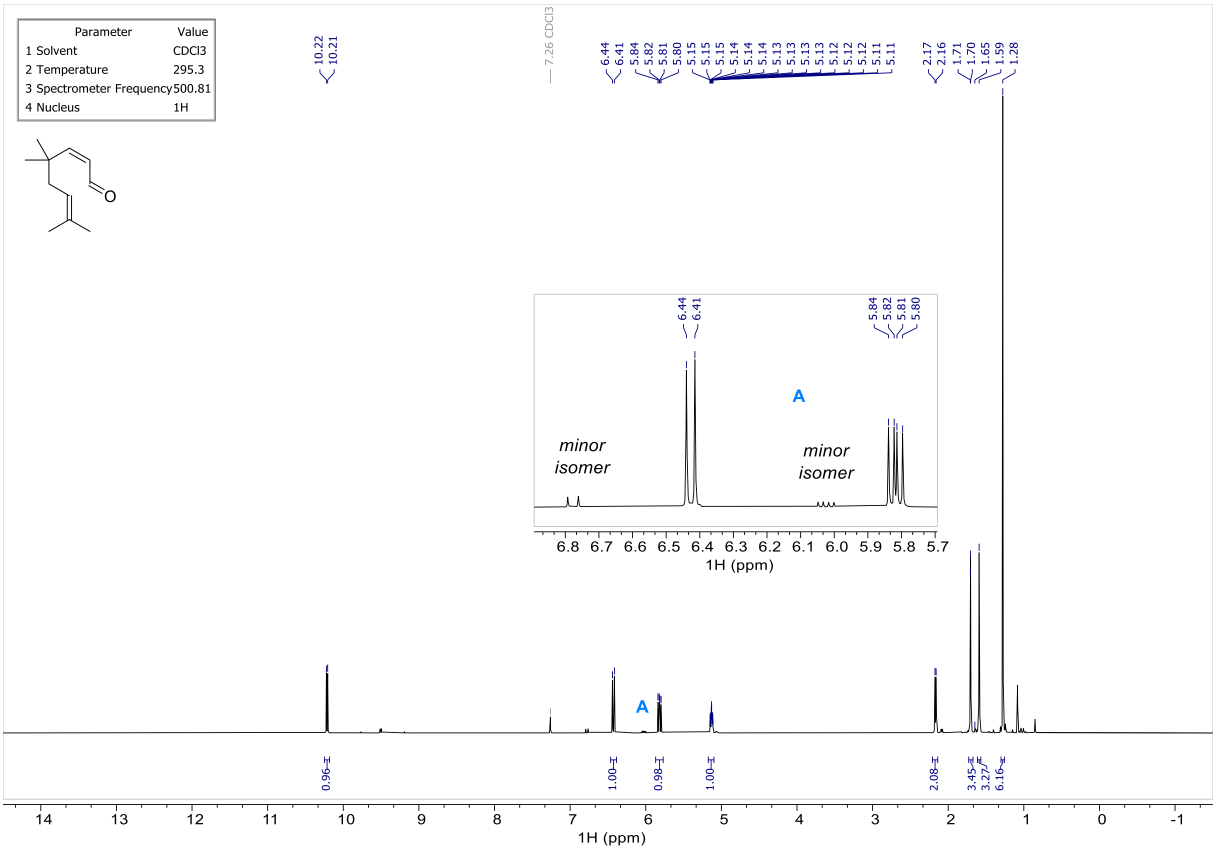

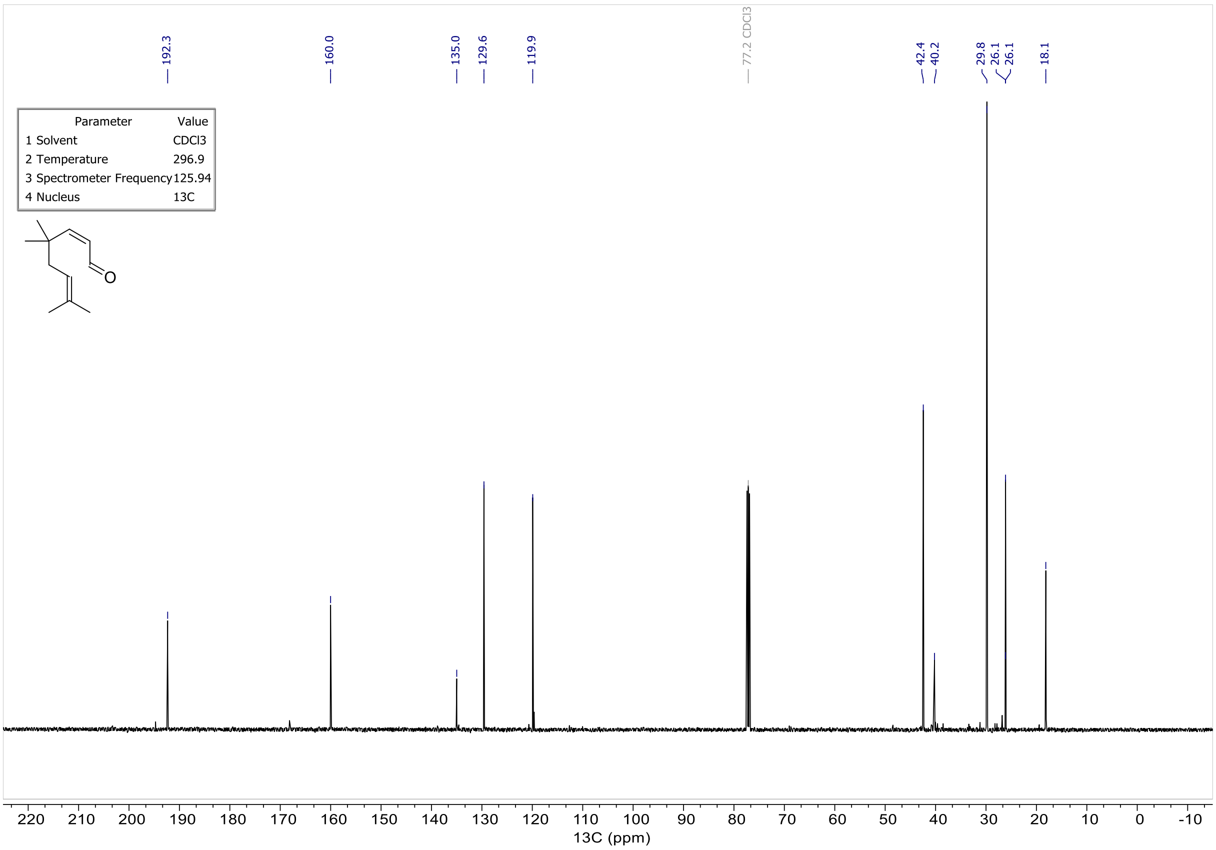

(*Z*)-*tert*-butyl((6-cyclohexylidene-3-methylhex-2-en-1-yl)oxy)diphenylsilane (**S45**)

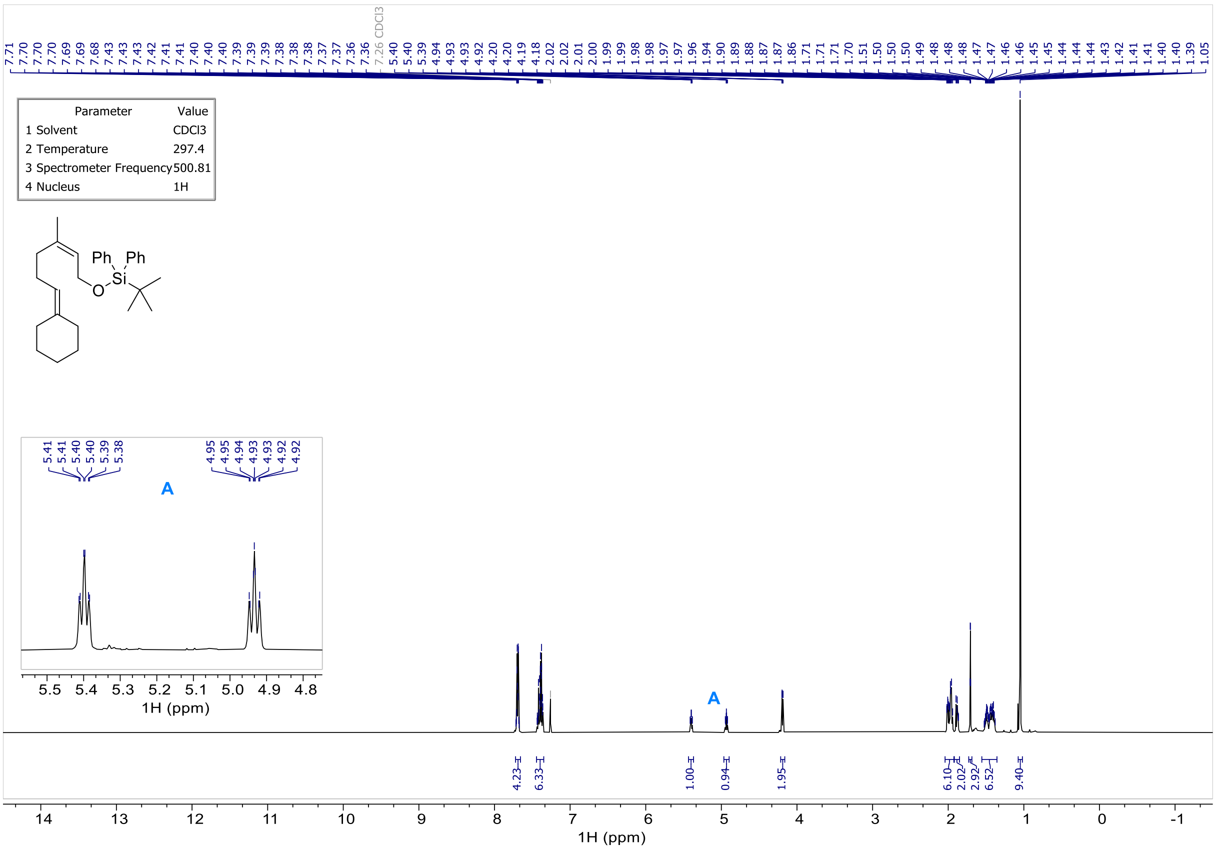

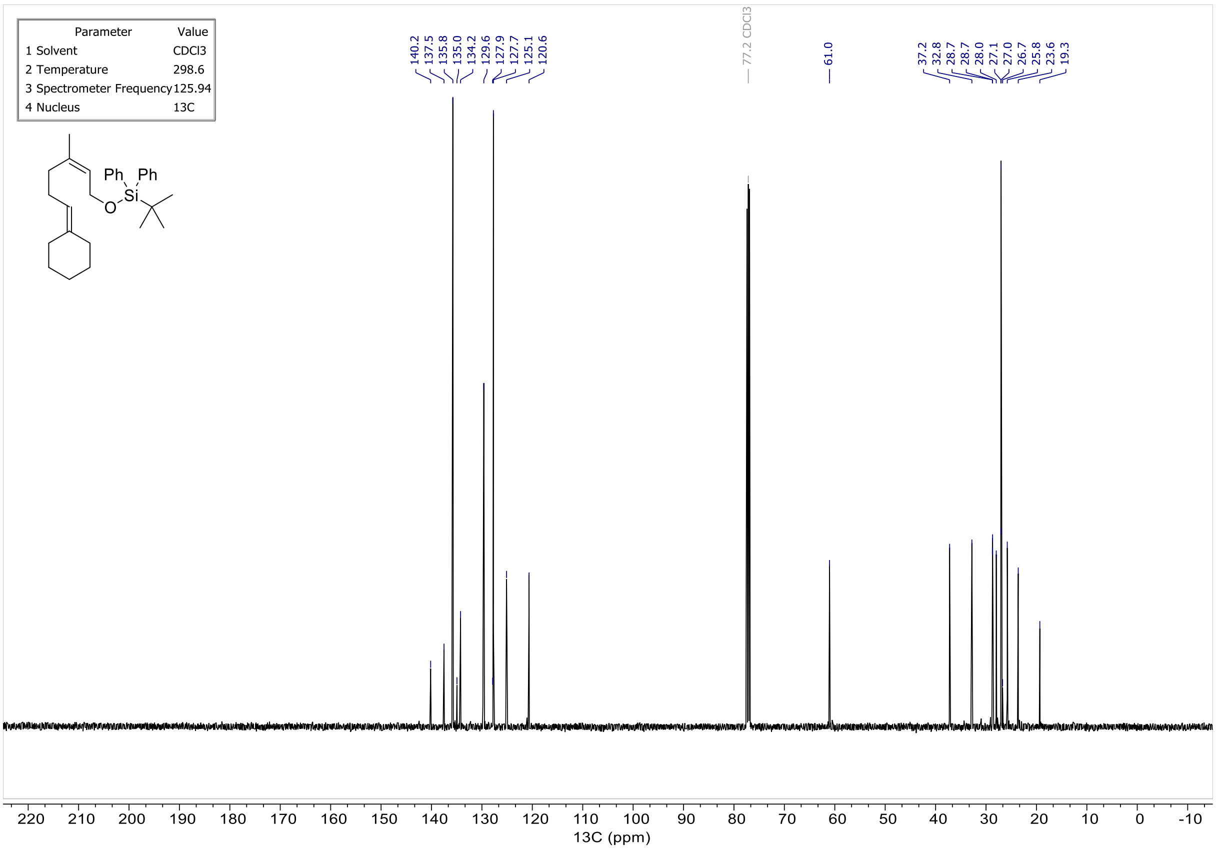

(*Z*)-6-cyclohexylidene-3-methylhex-2-en-1-ol (**S46**)

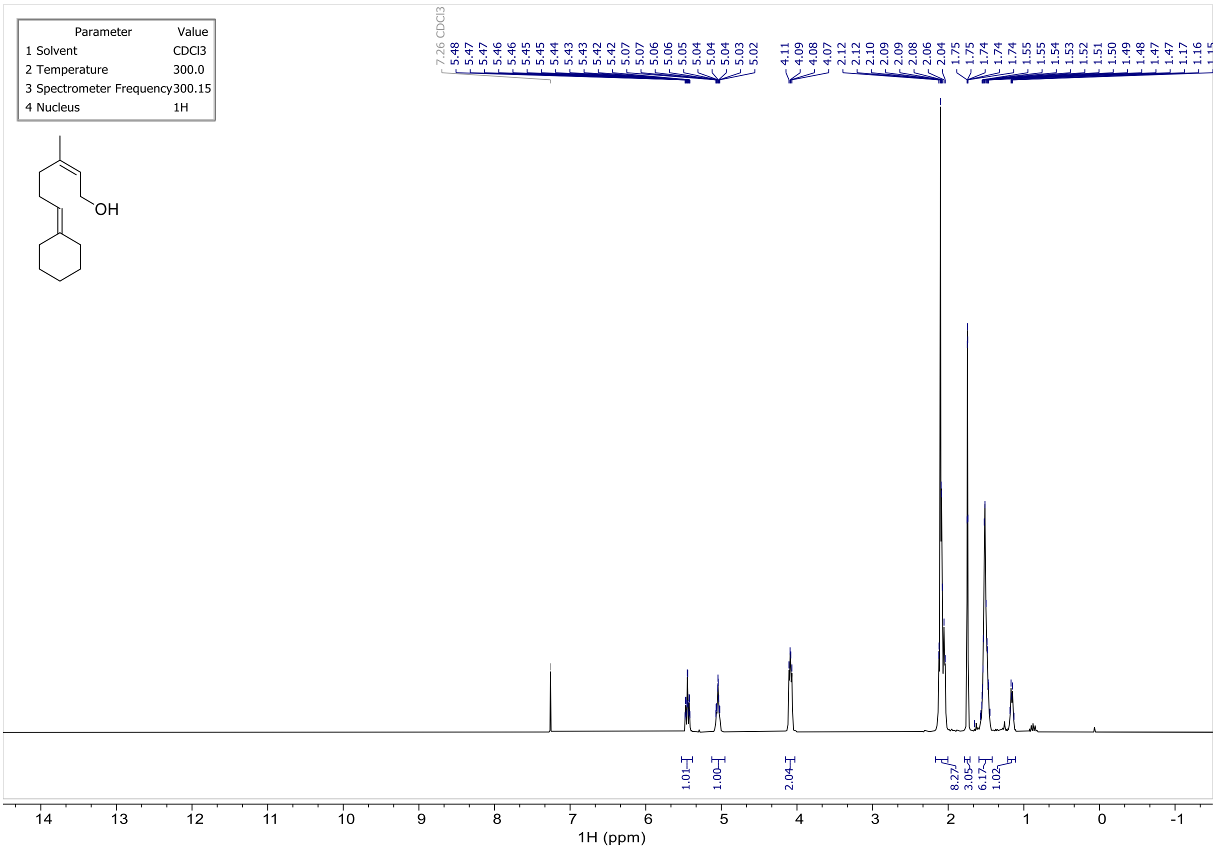

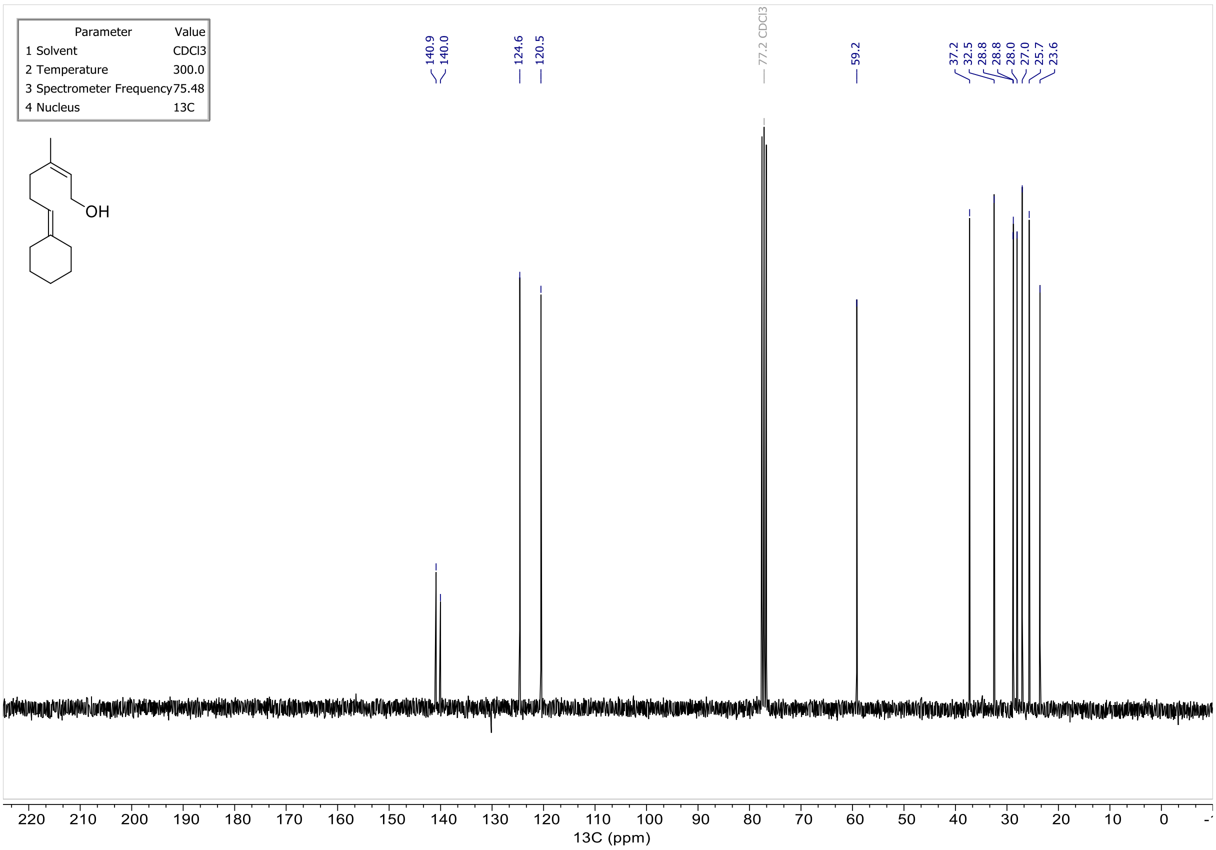

(*Z*)-6-cyclohexylidene-3-methylhex-2-enal (**S47**)

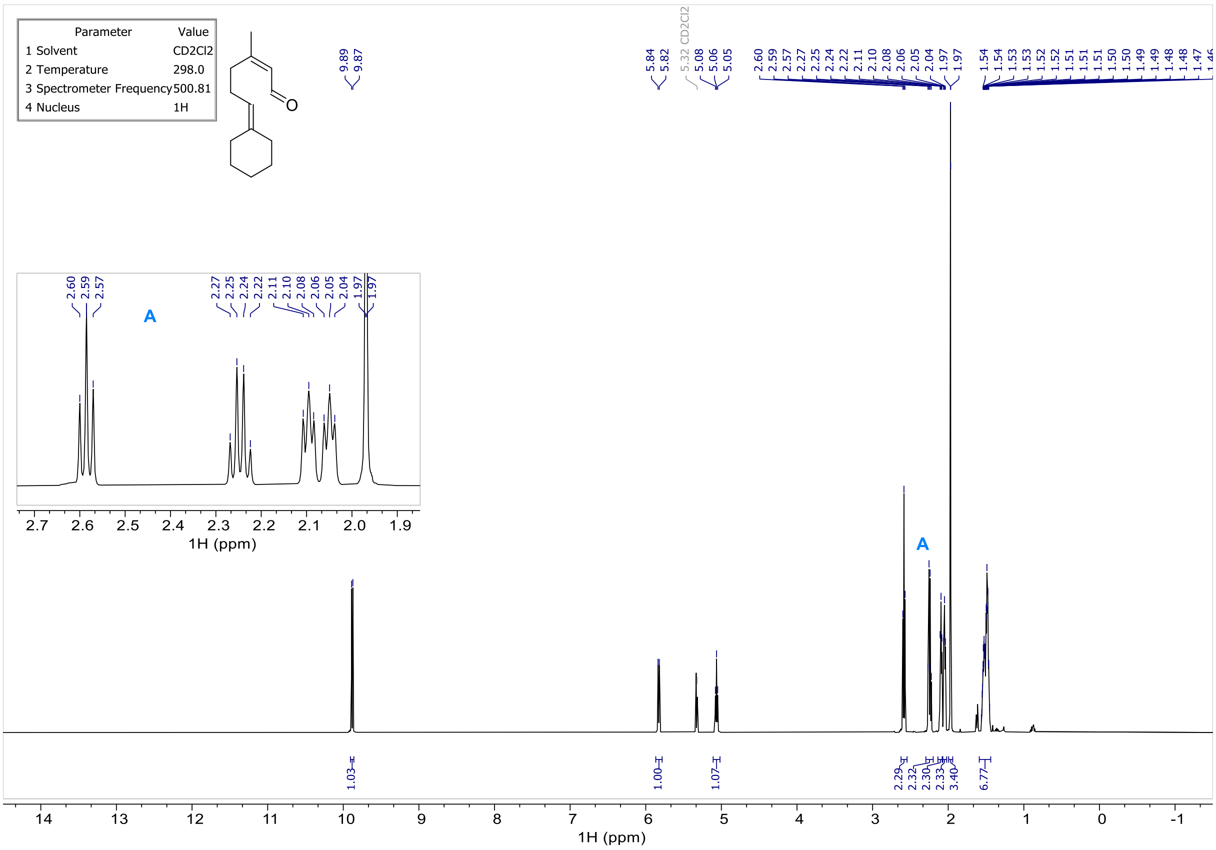

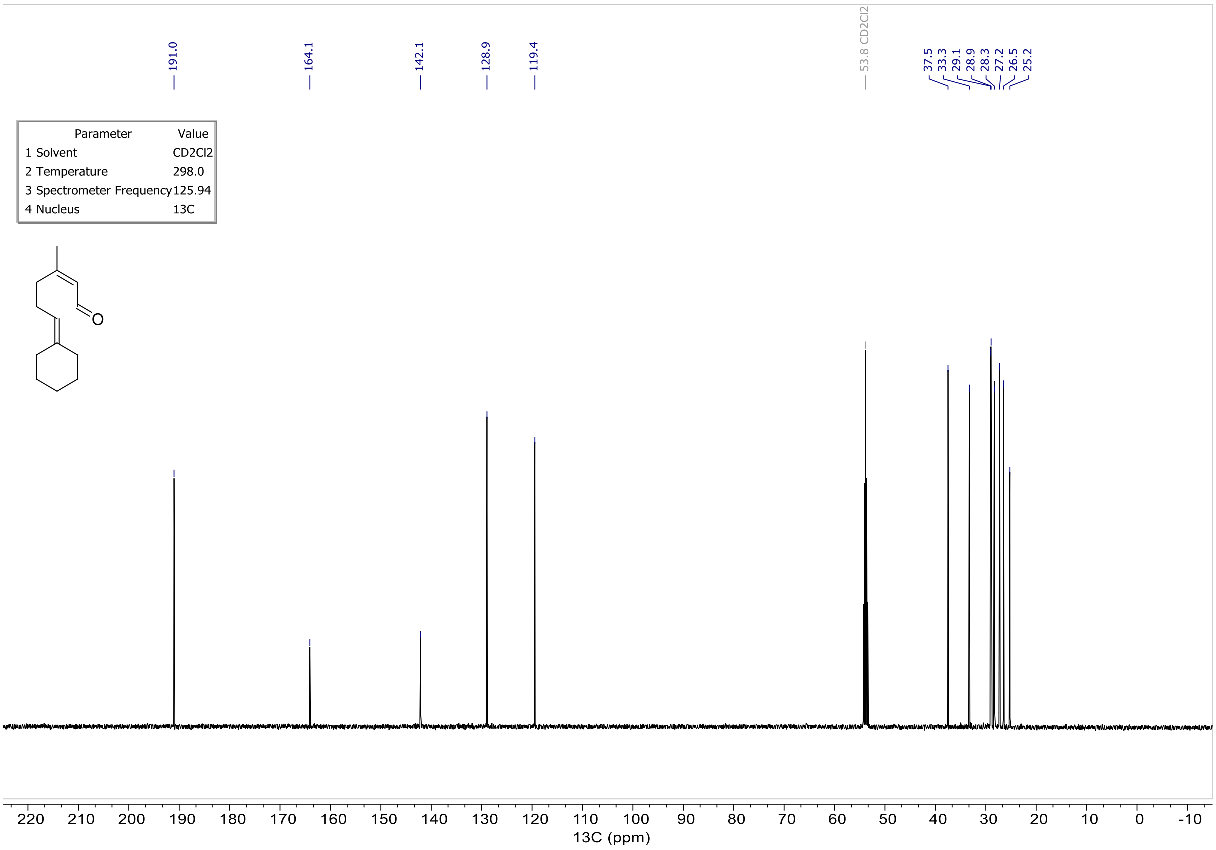

3-ethyl-6-(prop-1-en-2-yl)cyclohex-2-en-1-ol (**16**)

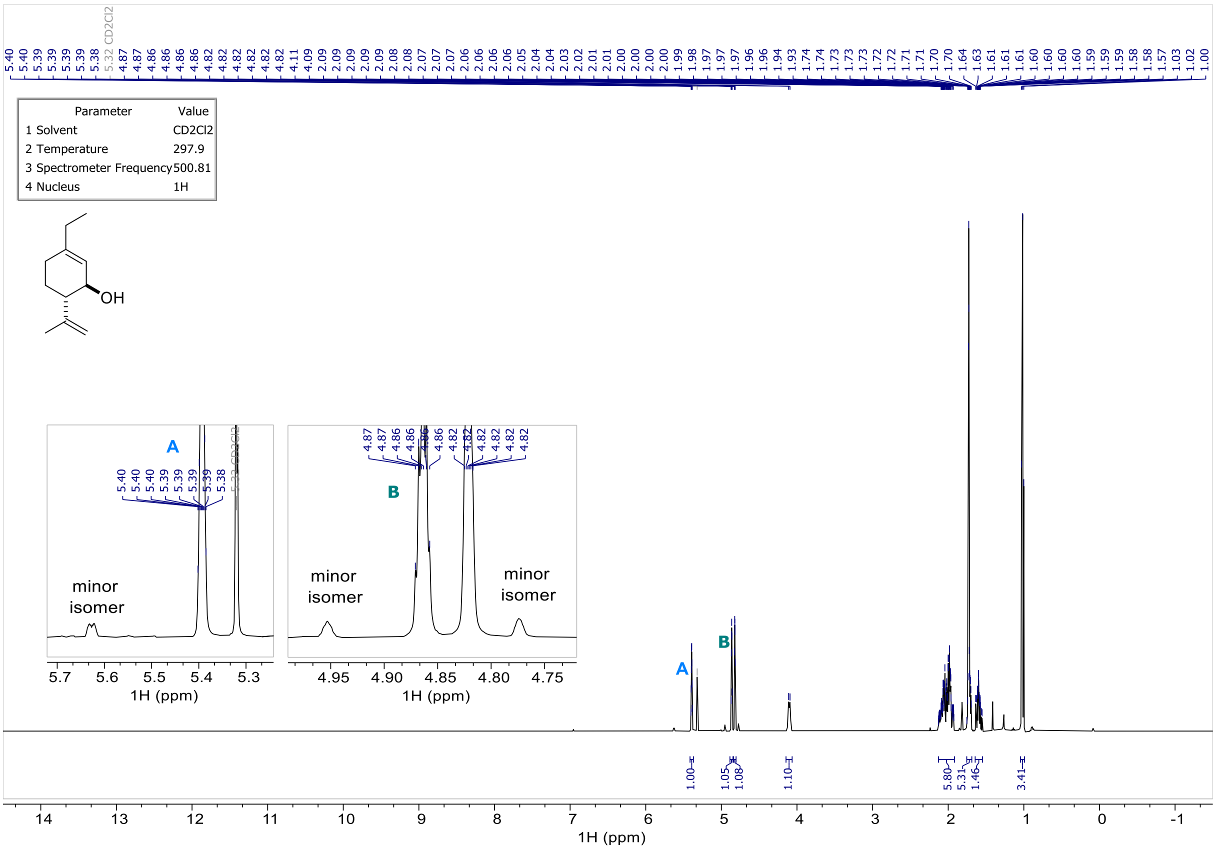

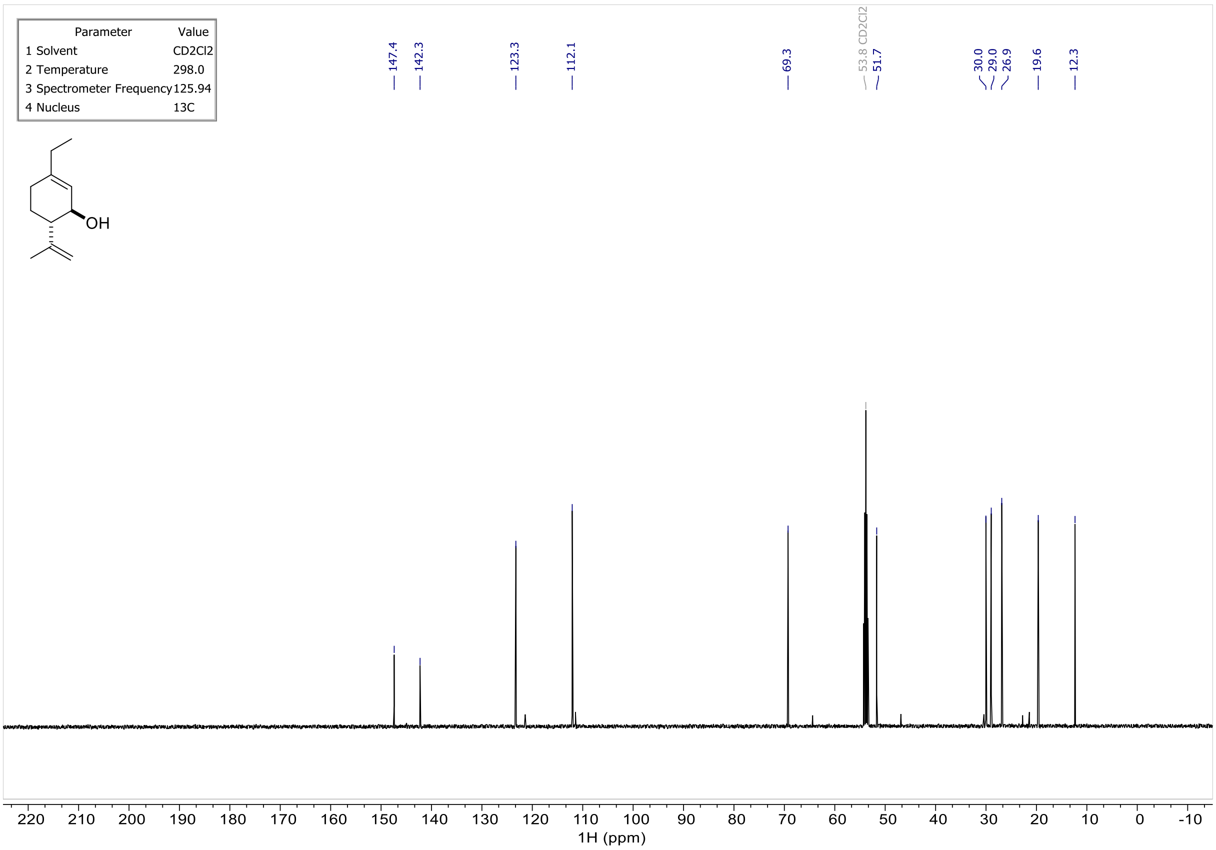

6-(prop-1-en-2-yl)-3-propylcyclohex-2-en-1-ol (**17**)

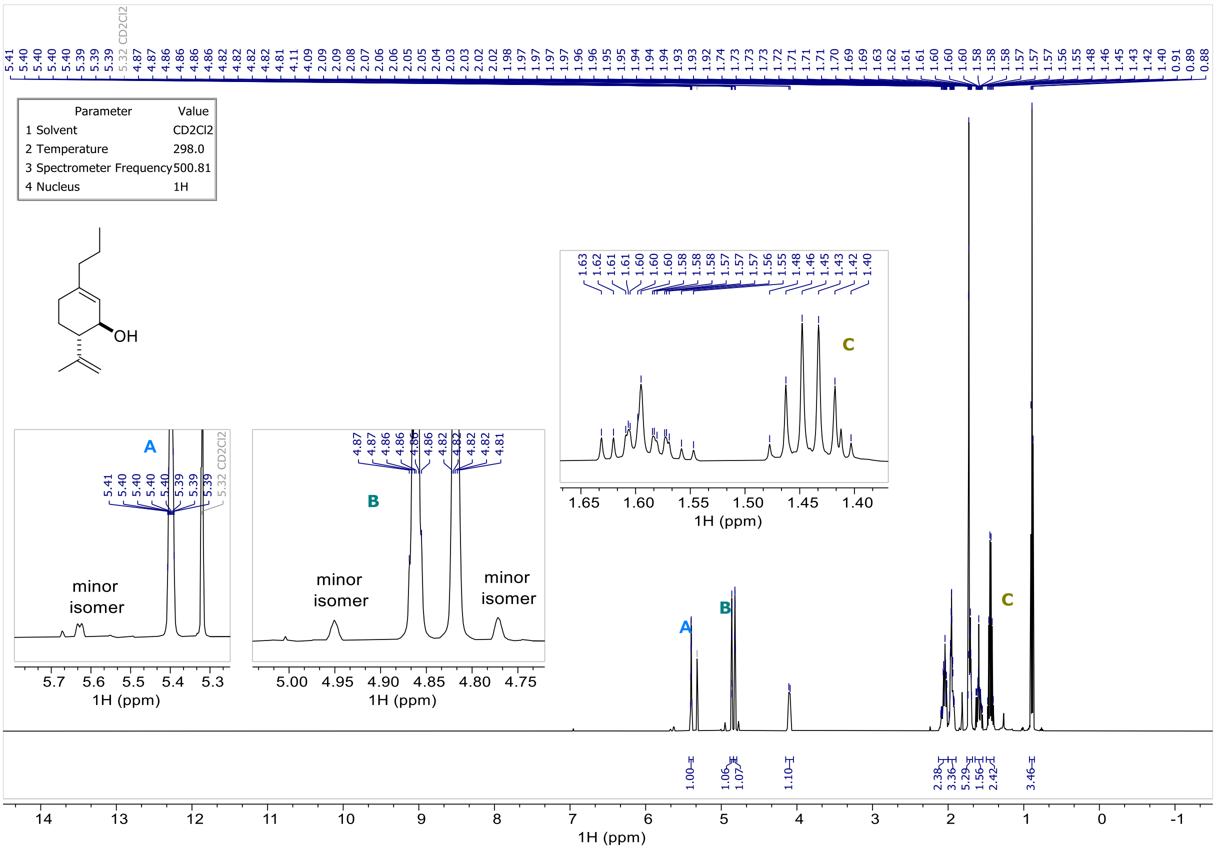

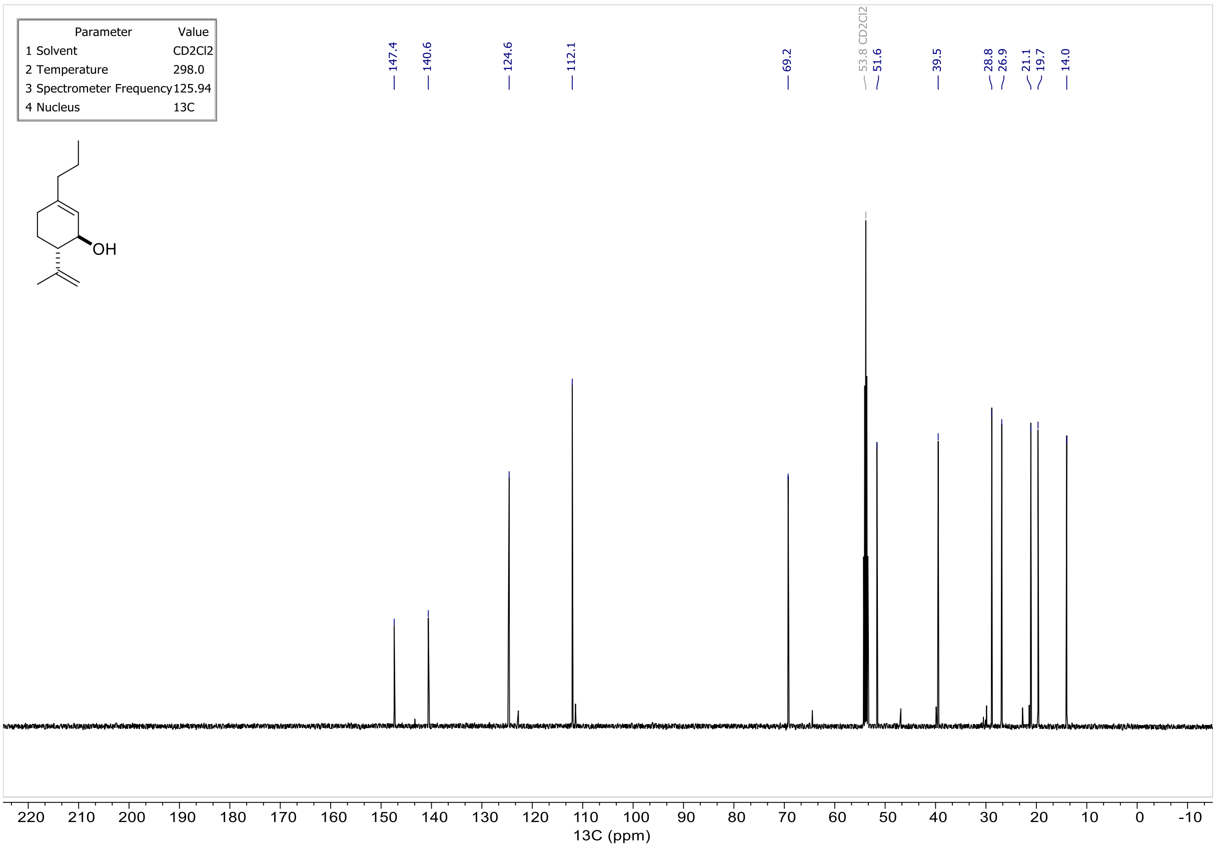

3-isobutyl-6-(prop-1-en-2-yl)cyclohex-2-en-1-ol (**18**)

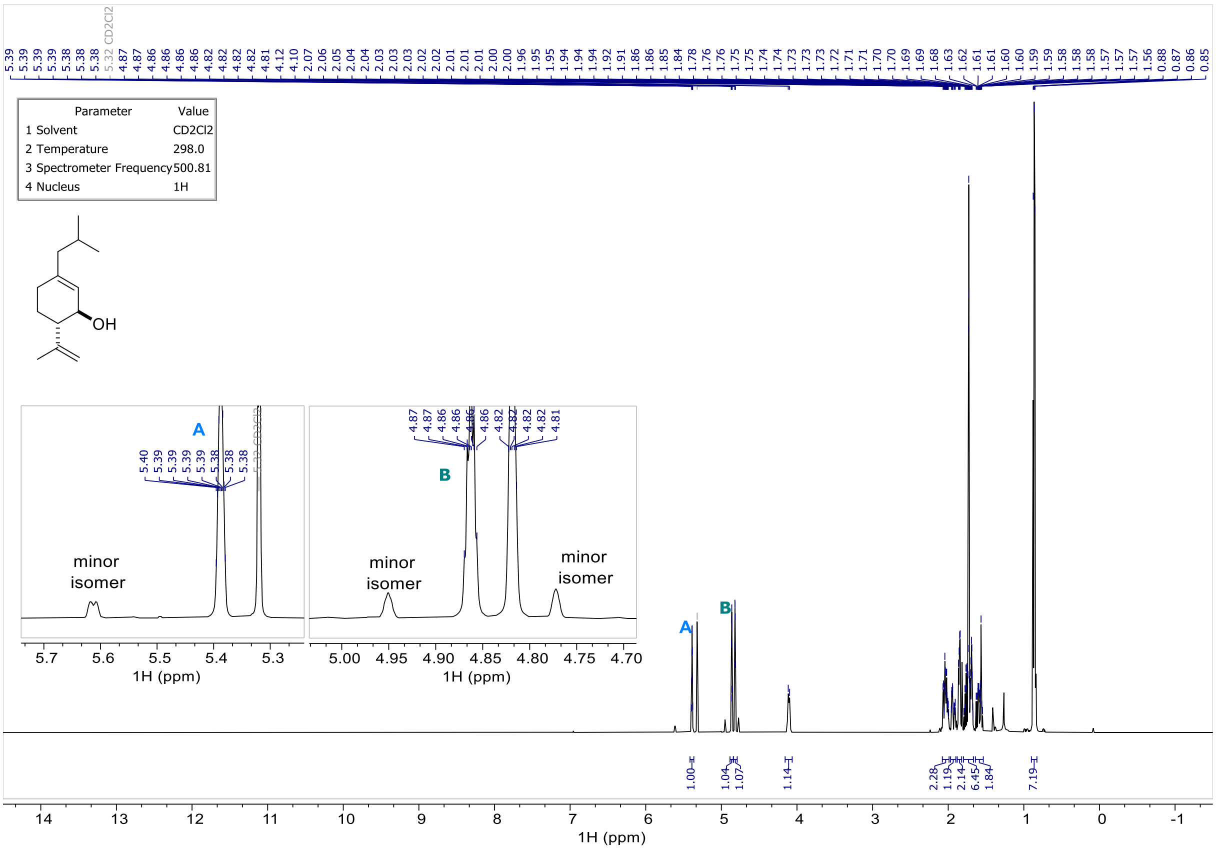

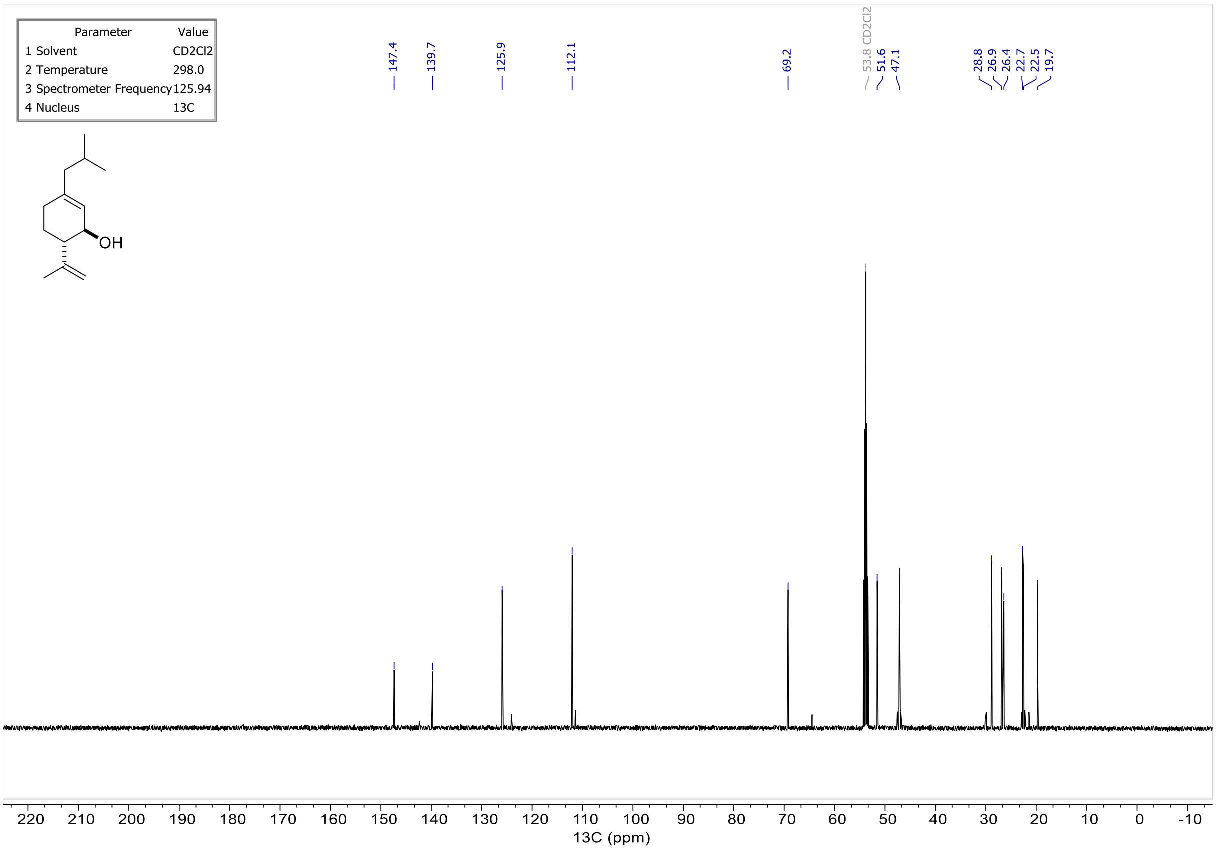

3-benzyl-6-(prop-1-en-2-yl)cyclohex-2-en-1-ol (**19**)

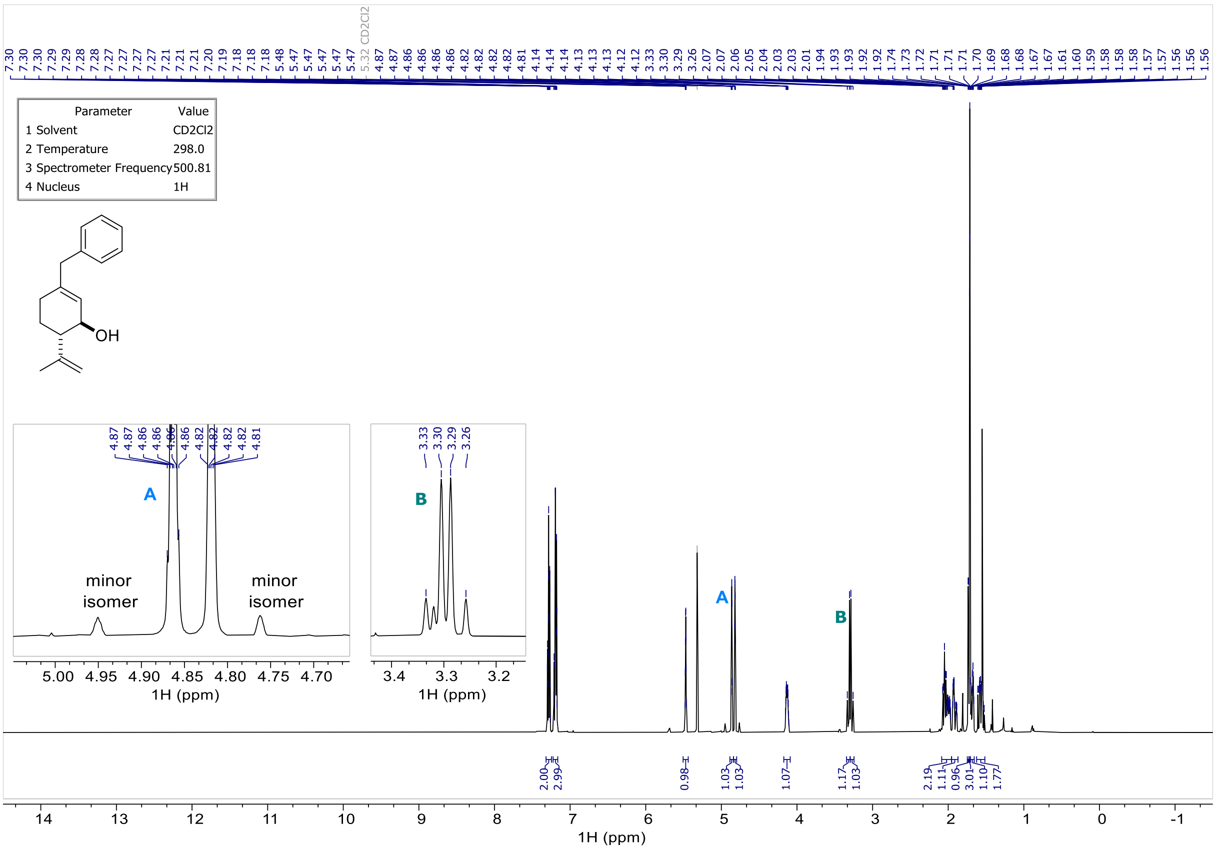

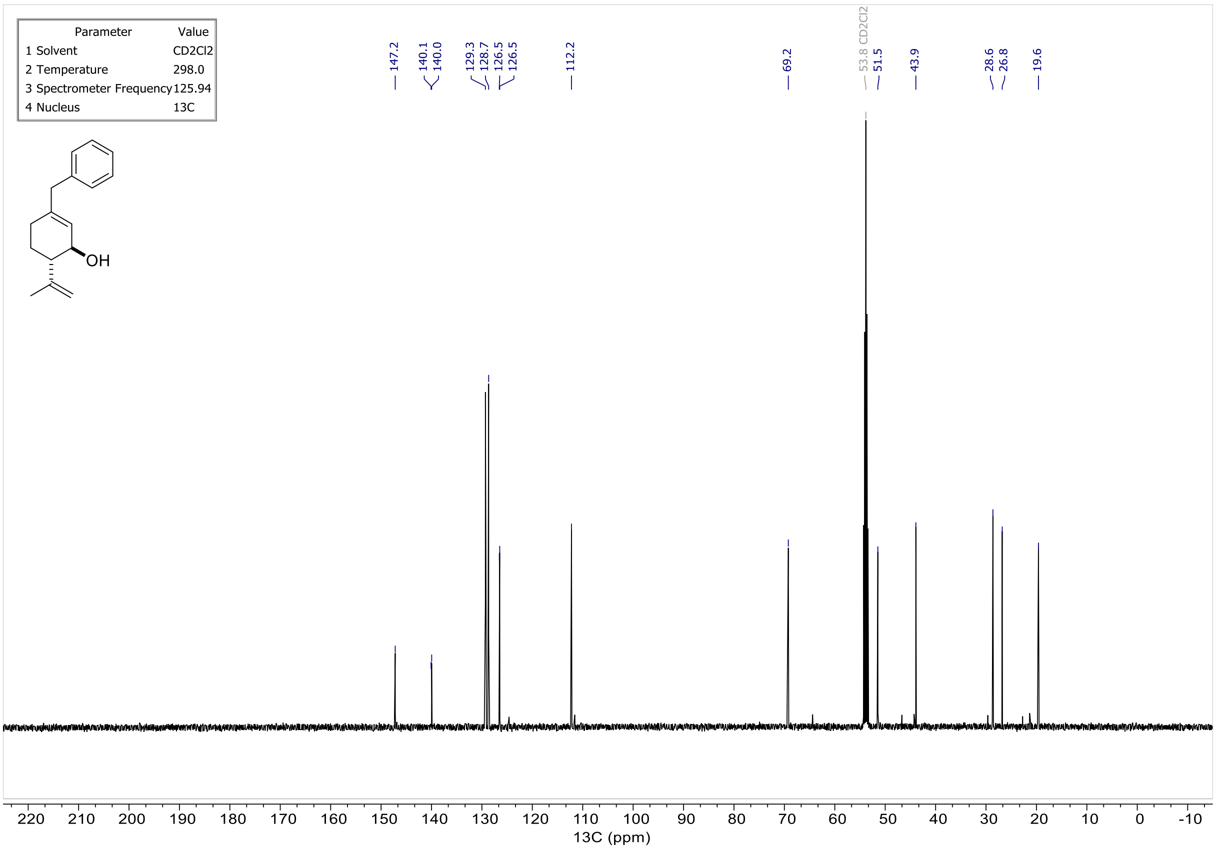

3-phenethyl-6-(prop-1-en-2-yl)cyclohex-2-en-1-ol (**20**)

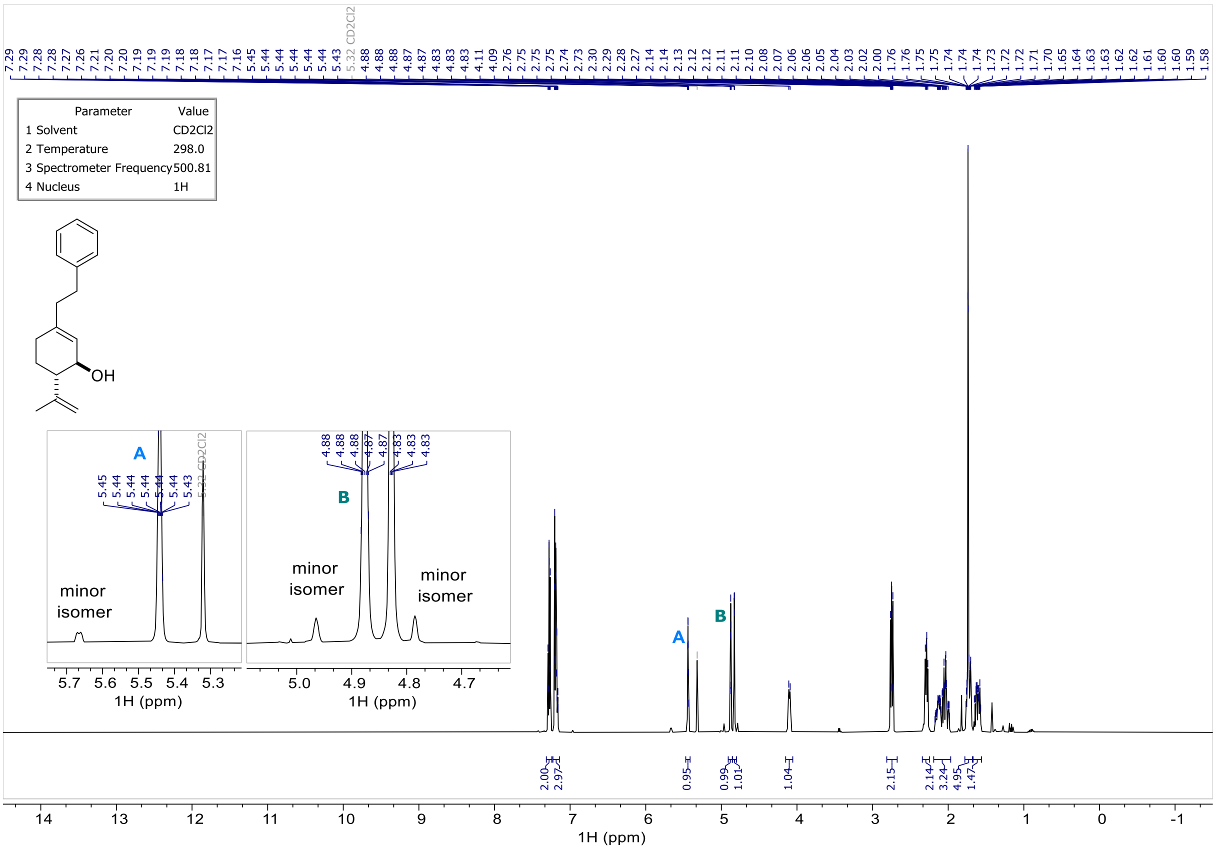

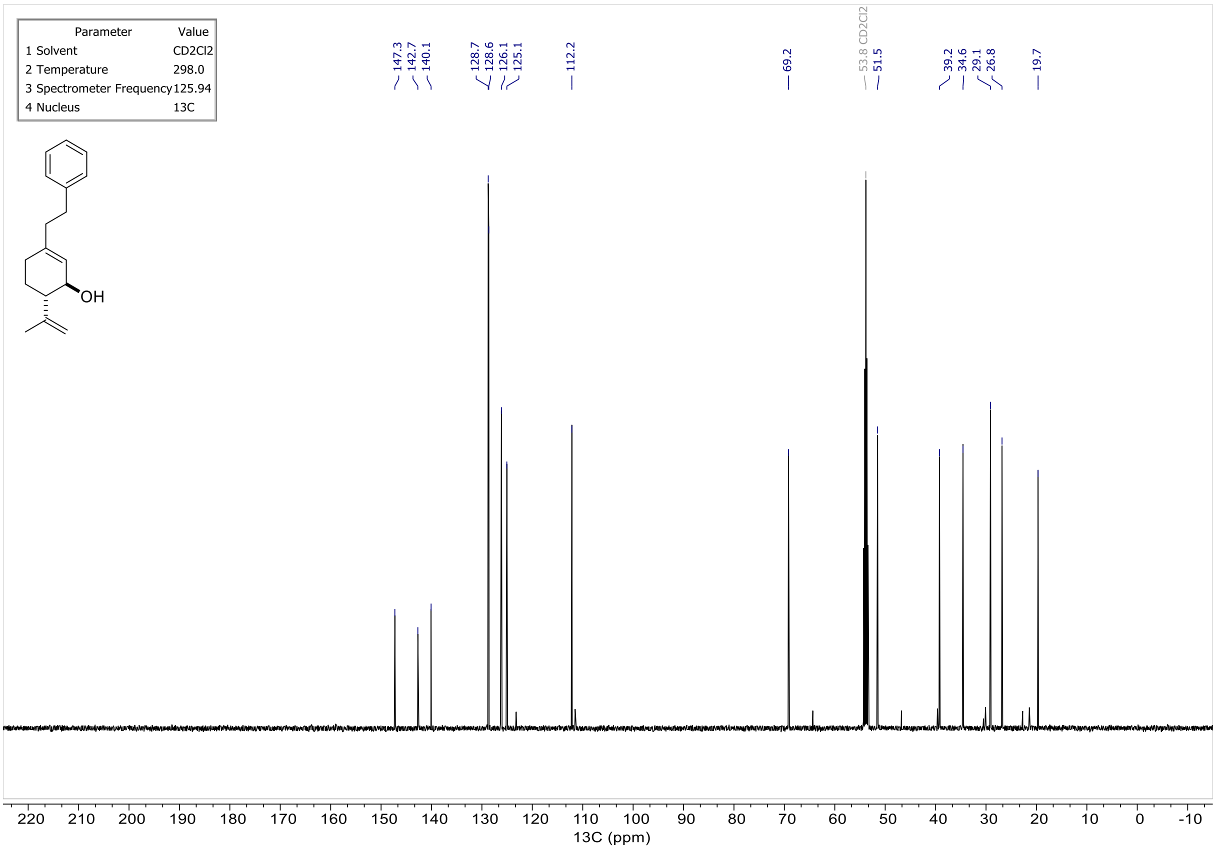

6-(prop-1-en-2-yl)-3-(5-(triethylsilyl)pent-4-yn-1-yl)cyclohex-2-en-1-ol (**21**)

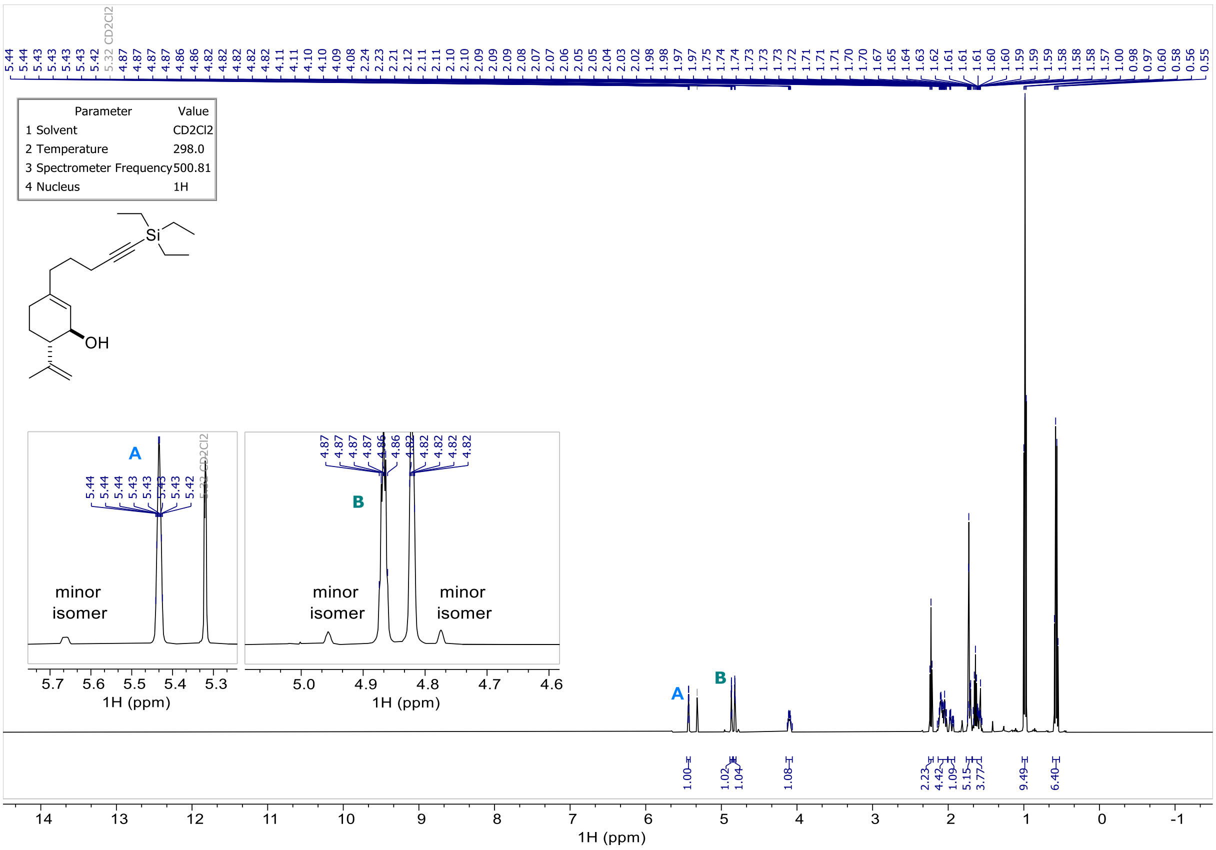

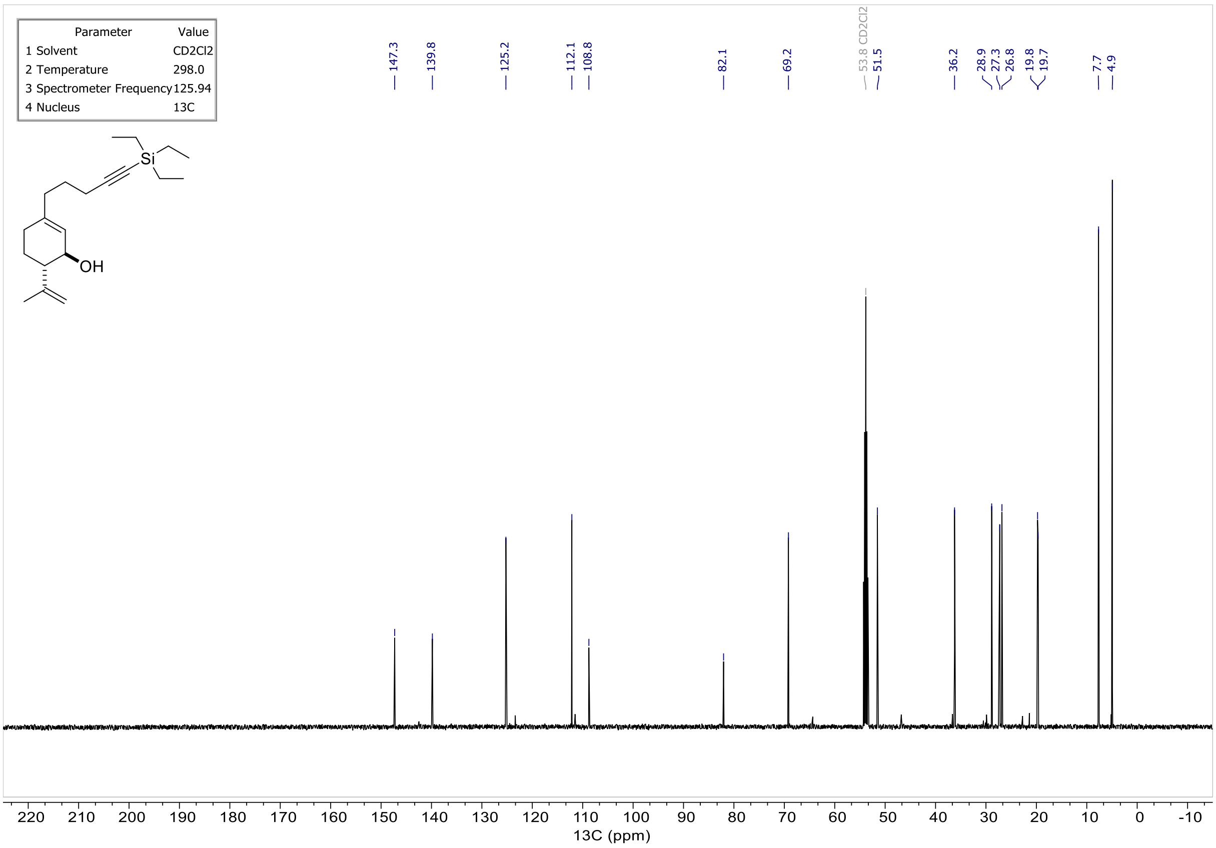

3-cyclopentyl-6-(prop-1-en-2-yl)cyclohex-2-en-1-ol (**22**)

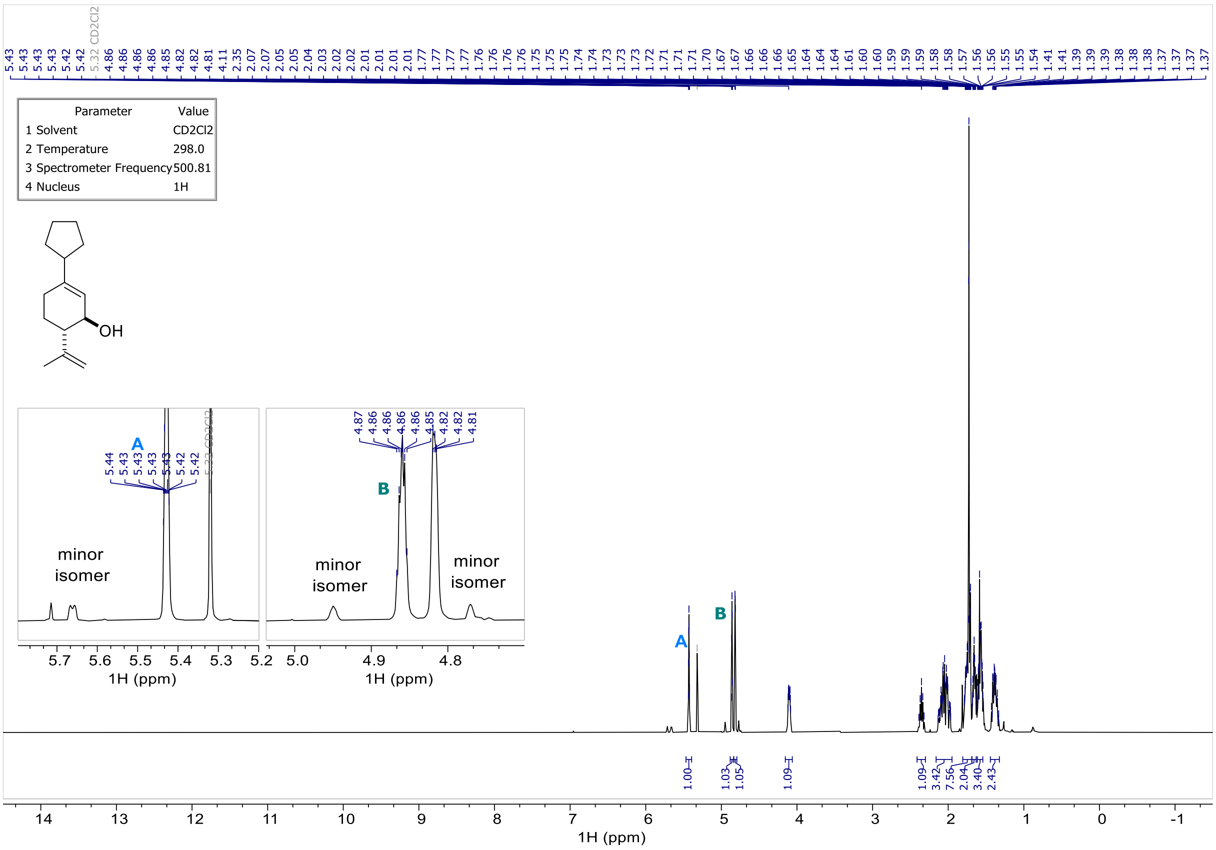

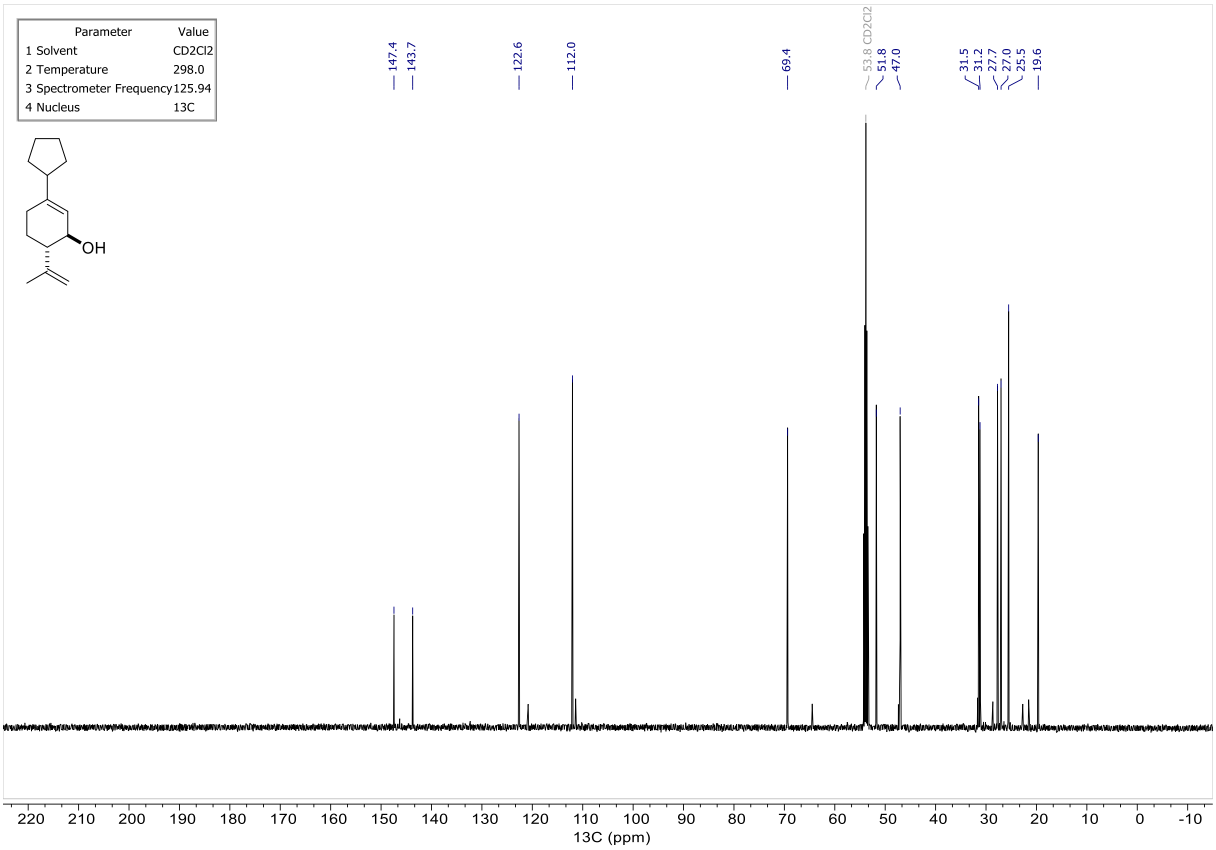

4,4-dimethyl-6-(prop-1-en-2-yl)cyclohex-2-en-1-ol (**23**)

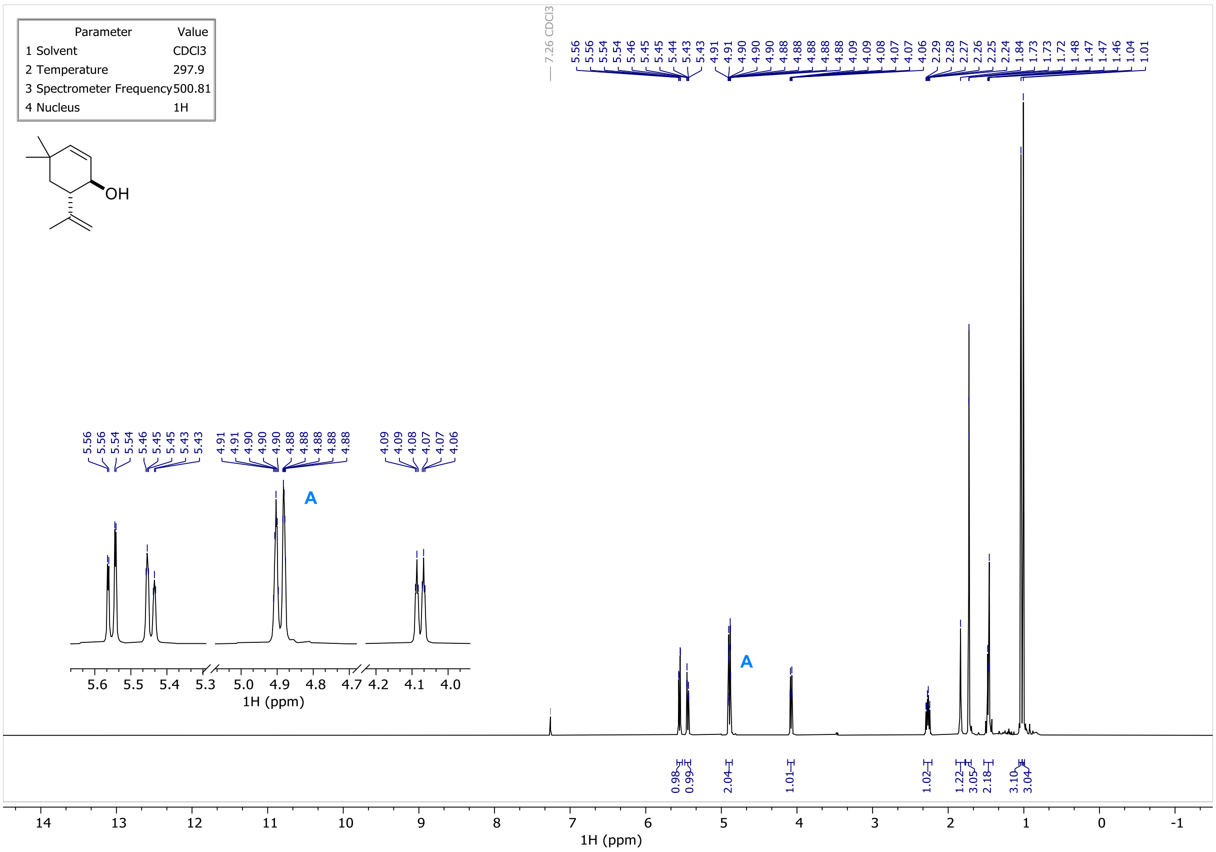

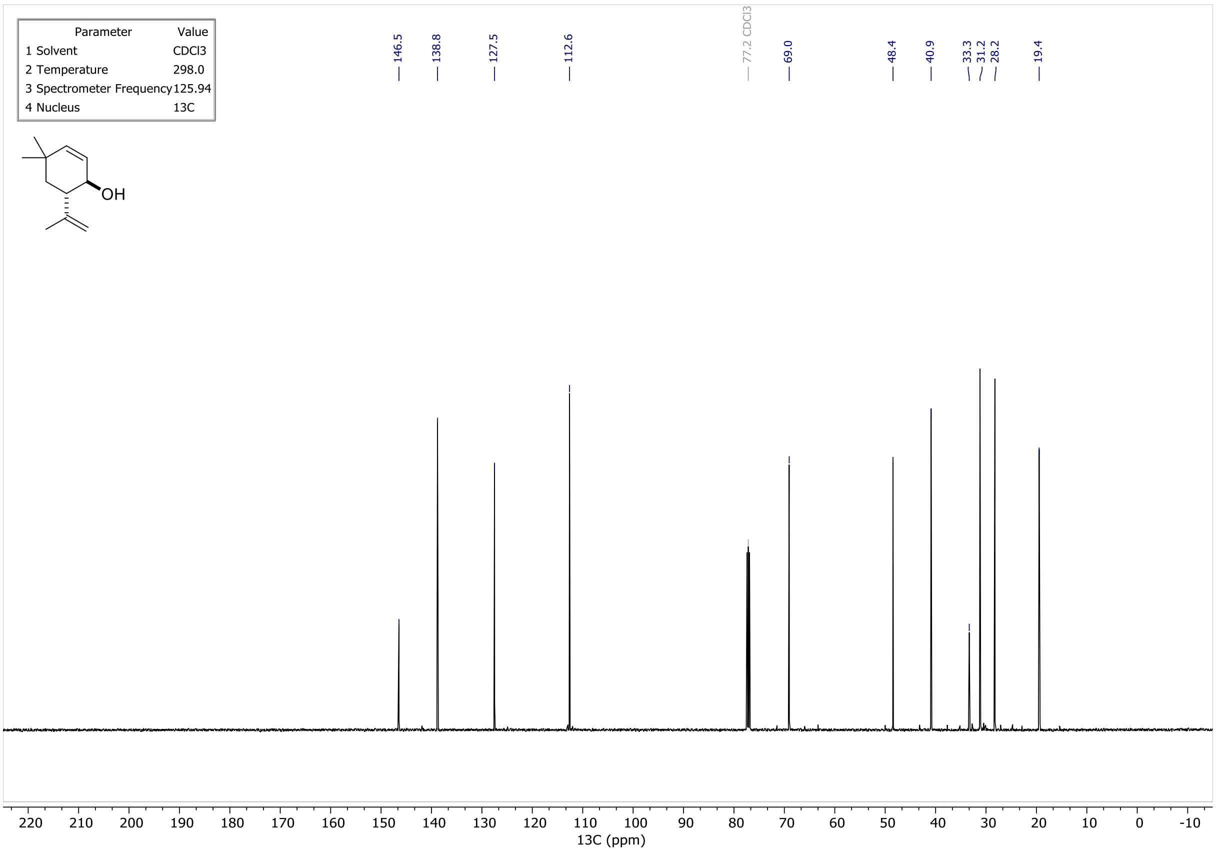

4-methyl-[1,1'-bi(cyclohexane)]-1',3-dien-2-ol (**24**)

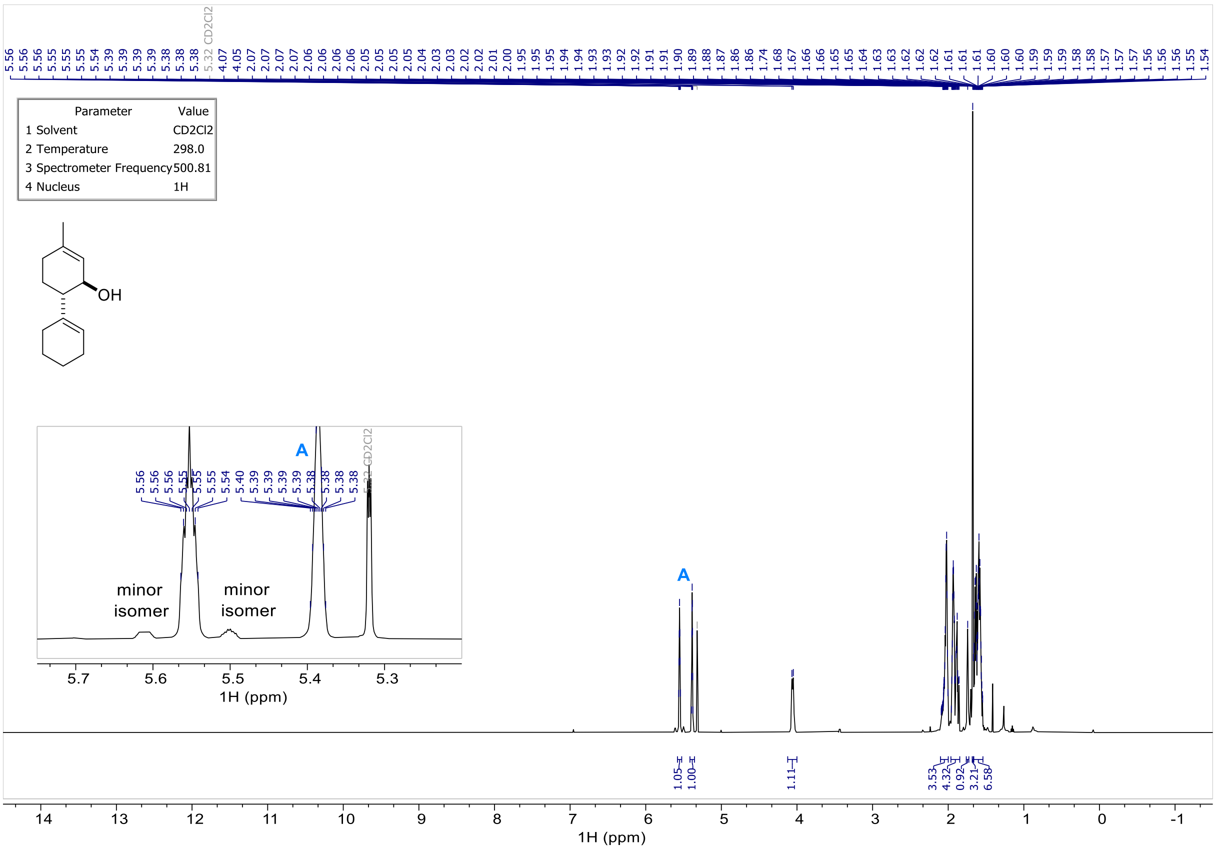

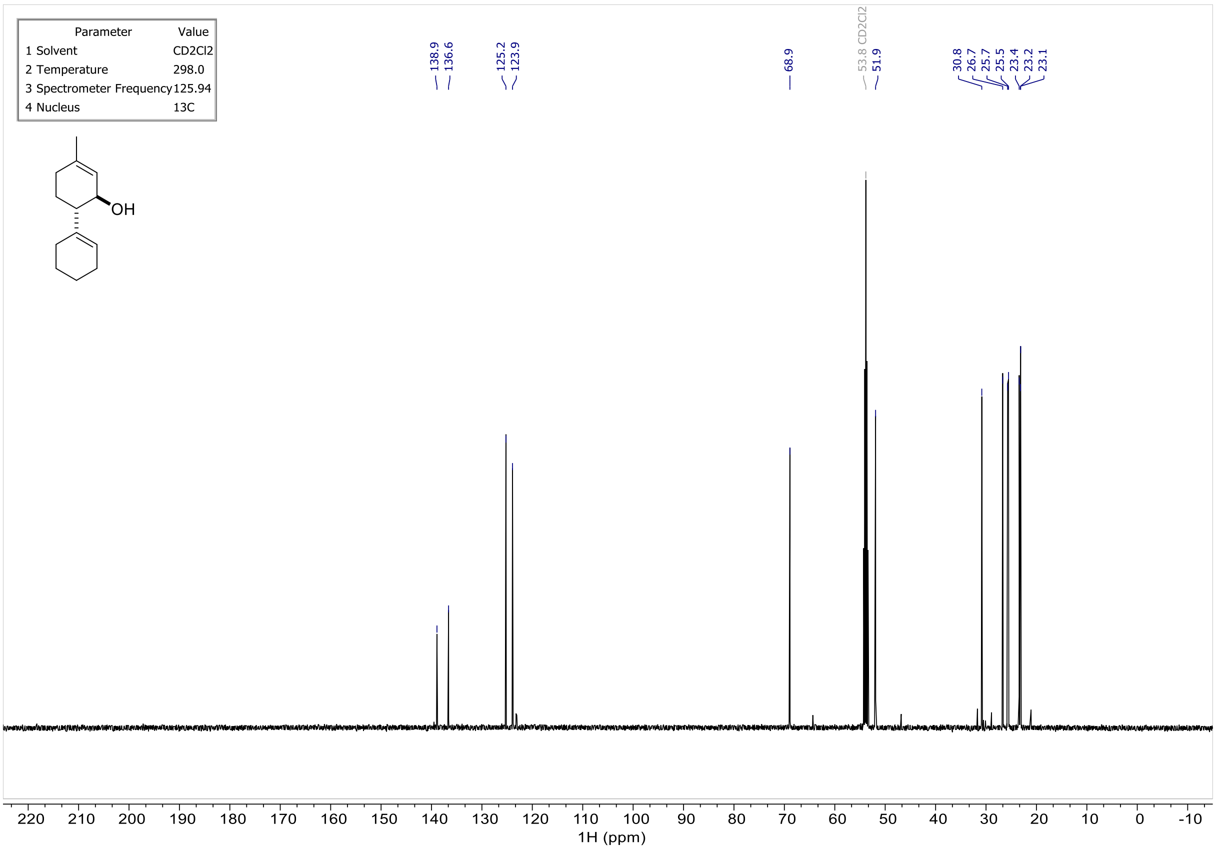

ethyl (2*E*,6*Z*)-8-((*tert*-butyldiphenylsilyl)oxy)-2,6-dimethylocta-2,6-dienoate (**S48**)

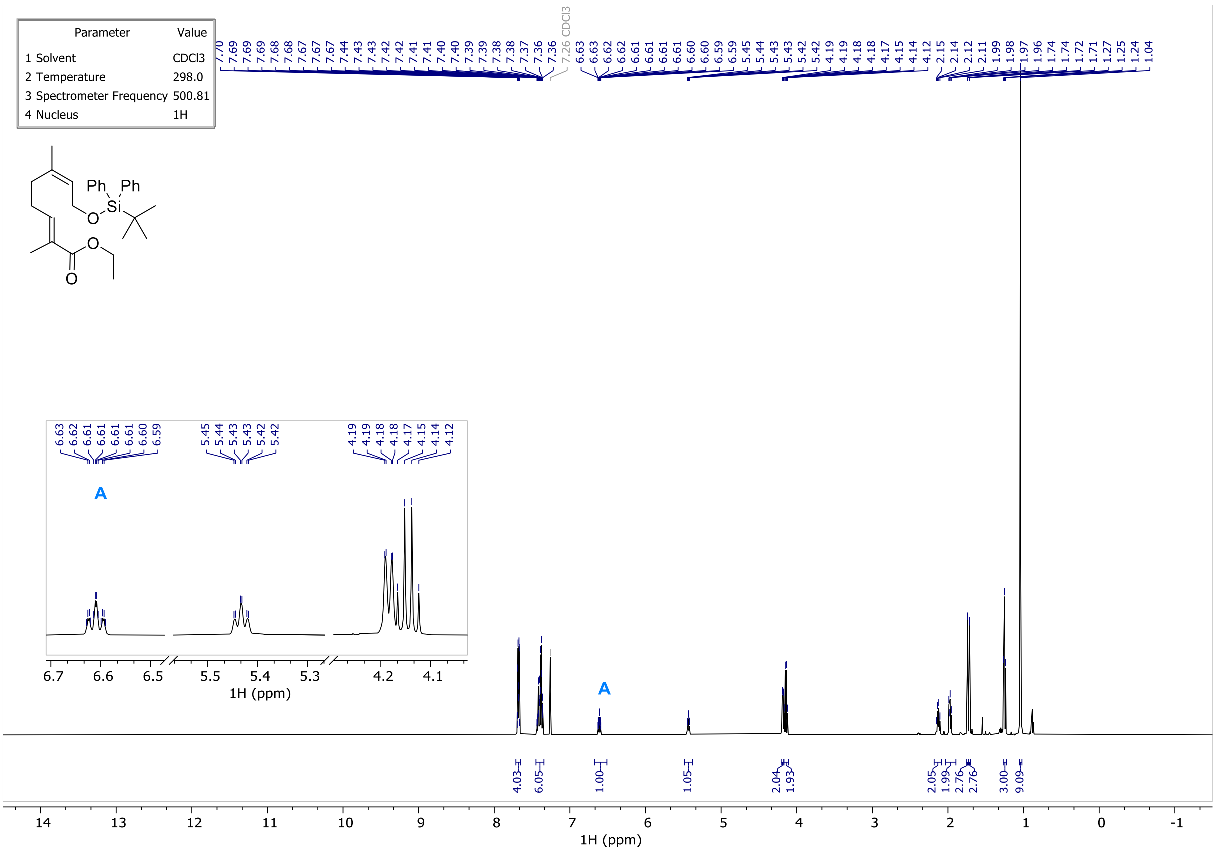

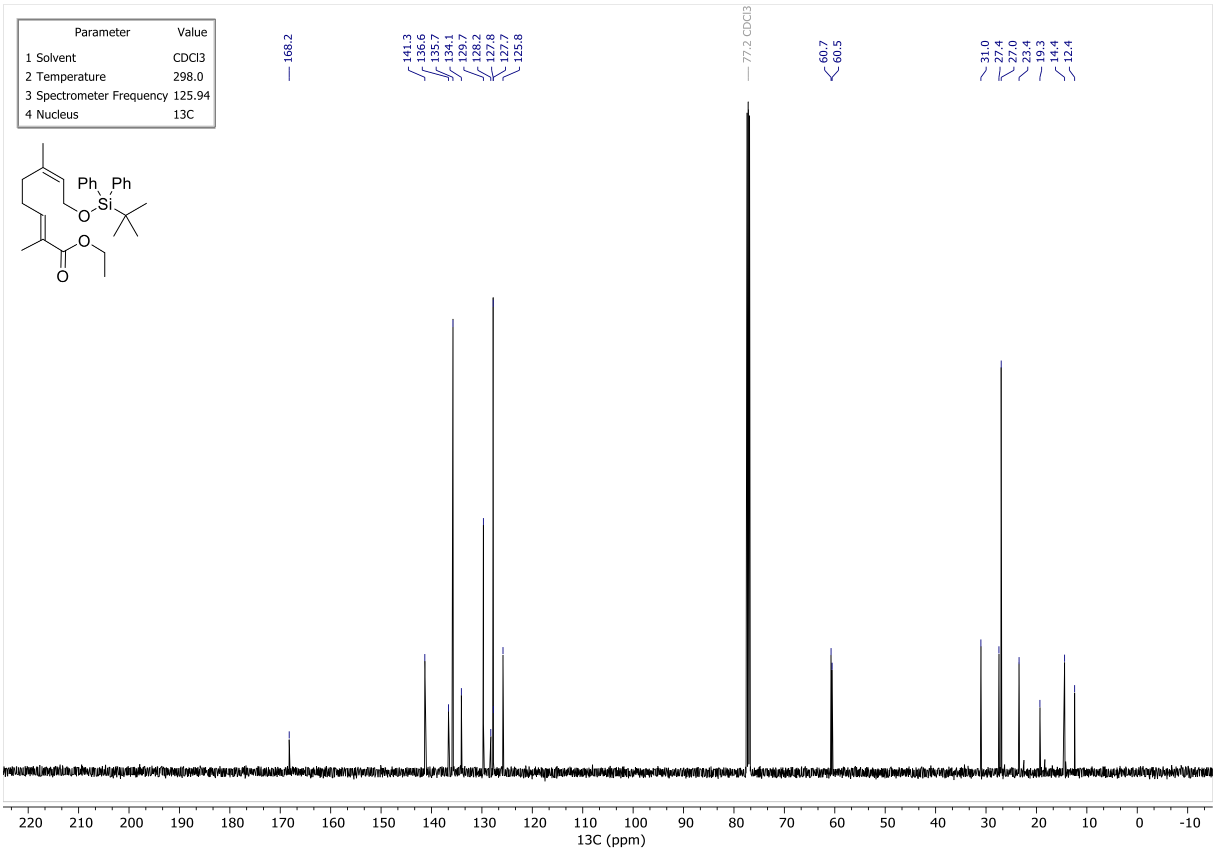

(2*E*,6*Z*)-8-((*tert*-butyldiphenylsilyl)oxy)-2,6-dimethylocta-2,6-dien-1,1-d_2-1-ol (**S49**)

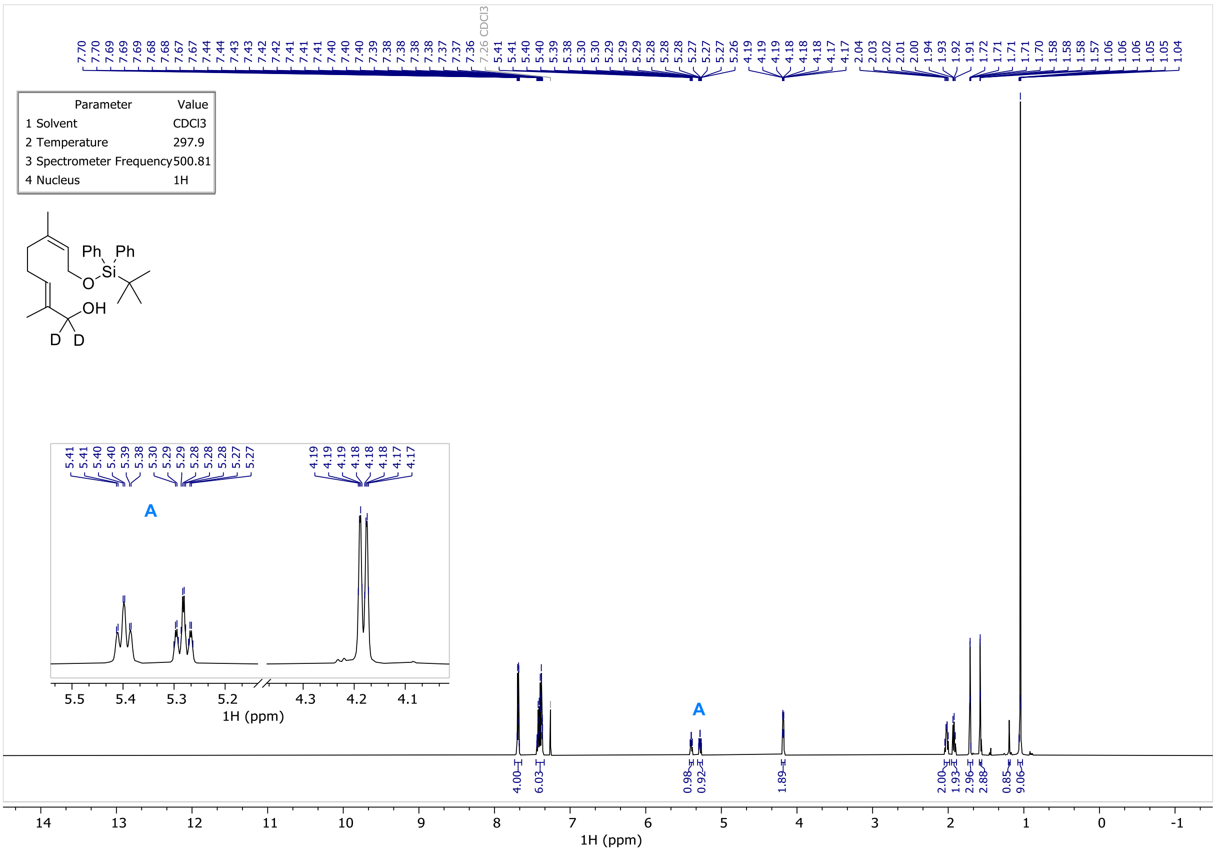

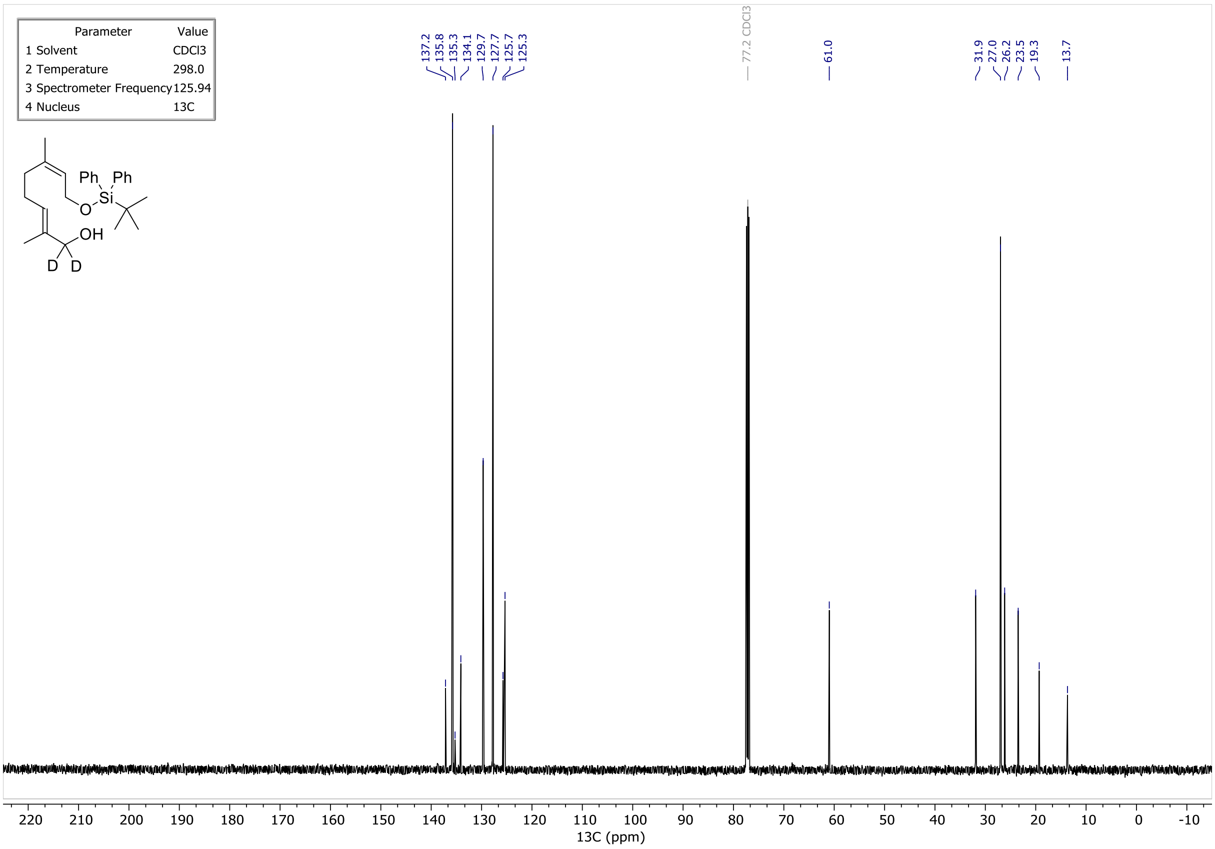

(2*E*,6*Z*)-8-((*tert*-butyldiphenylsilyl)oxy)-2,6-dimethylocta-2,6-dien-1-yl-1,1-d_2 diethyl phosphate (**S50**)

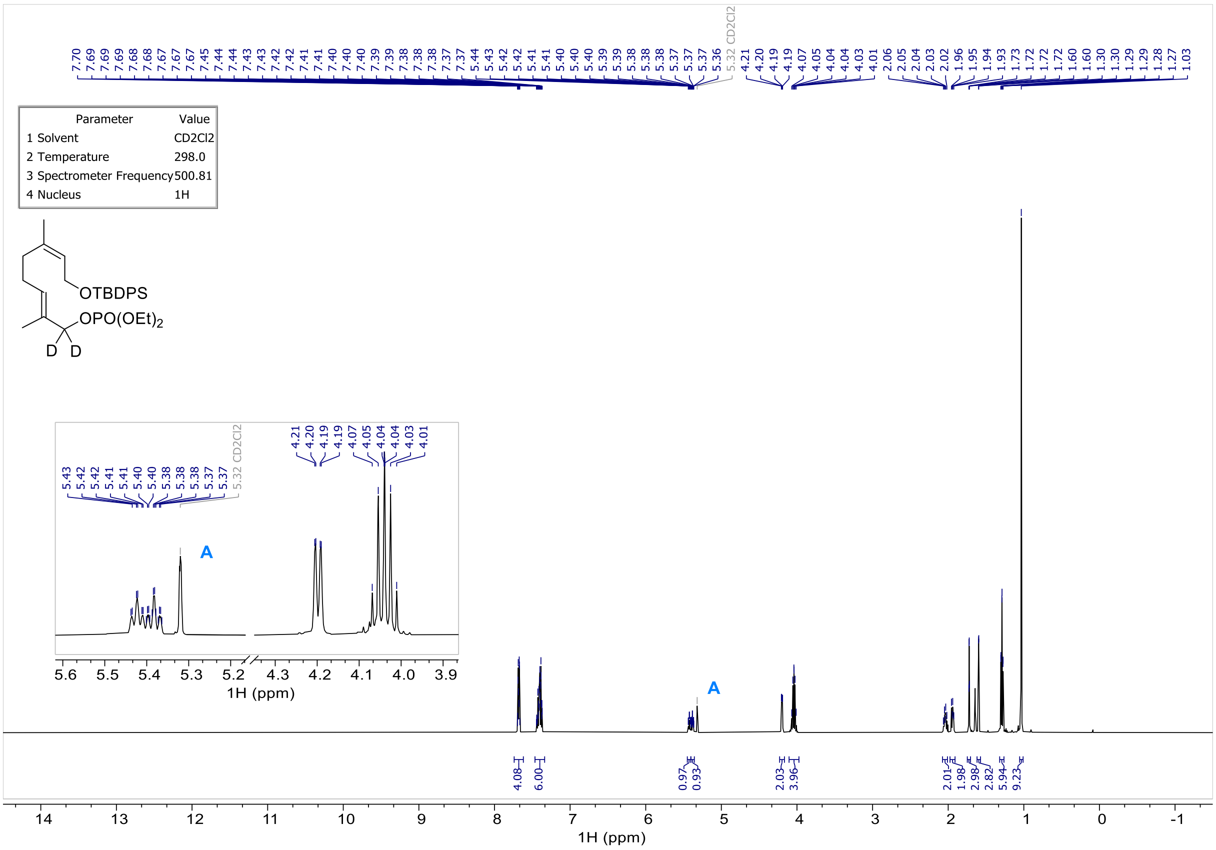

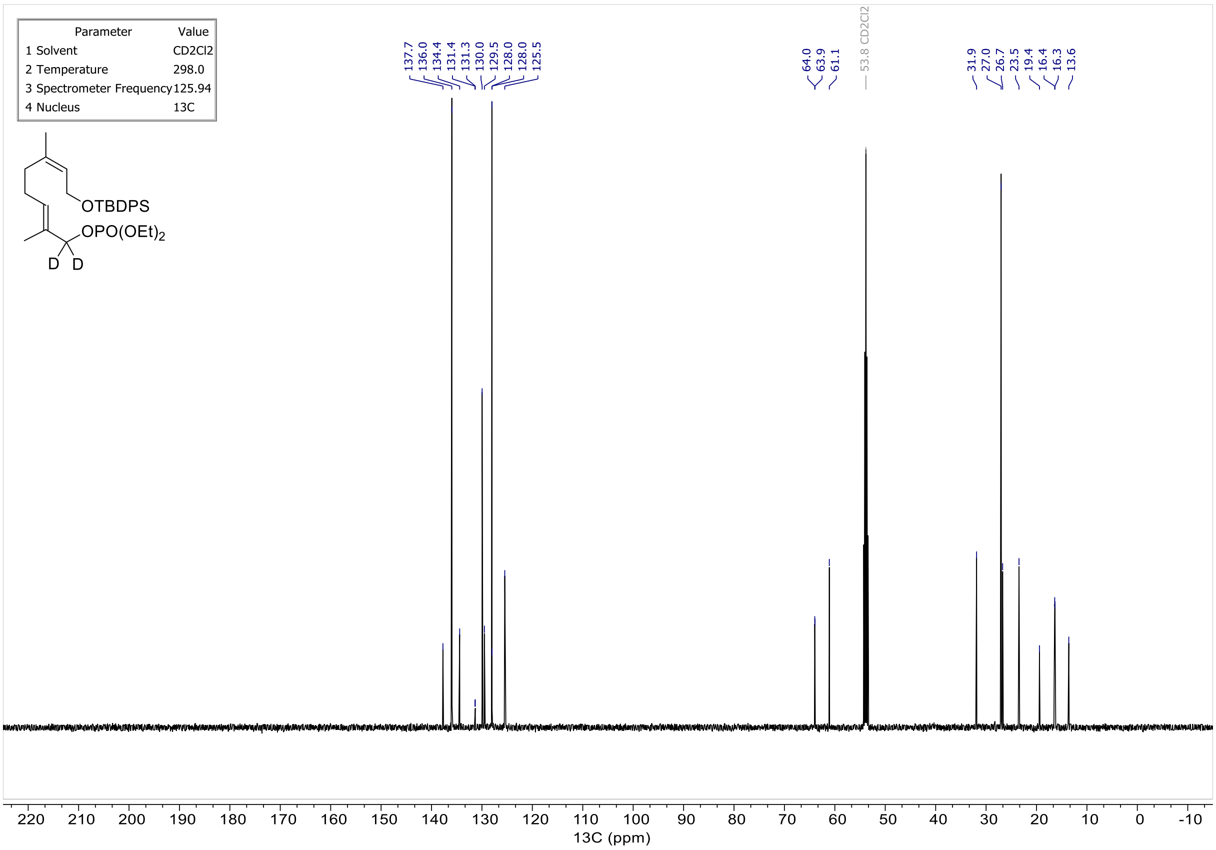

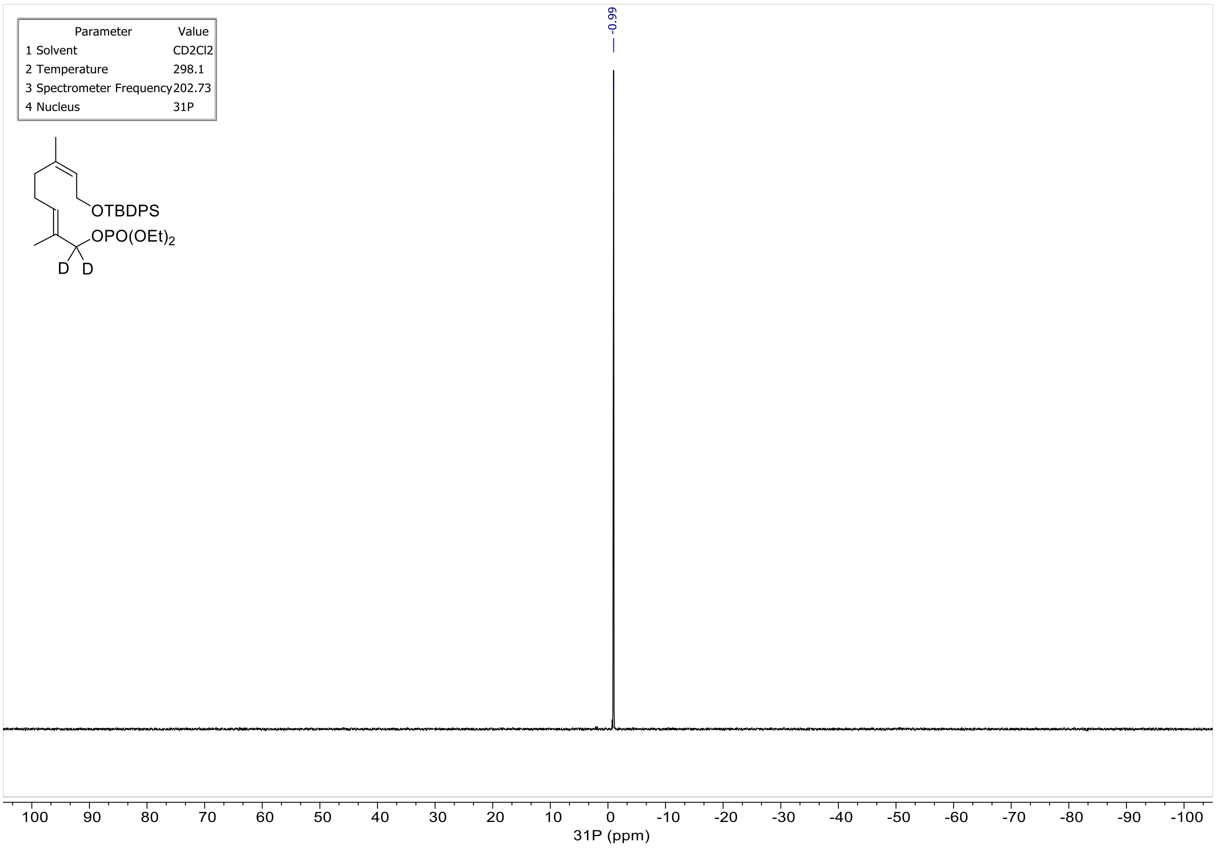

tert-butyl(((2*Z*,6*E*)-3,7-dimethylocta-2,6-dien-1-yl-8,8,8-d_3)oxy)diphenylsilane (**S51**)

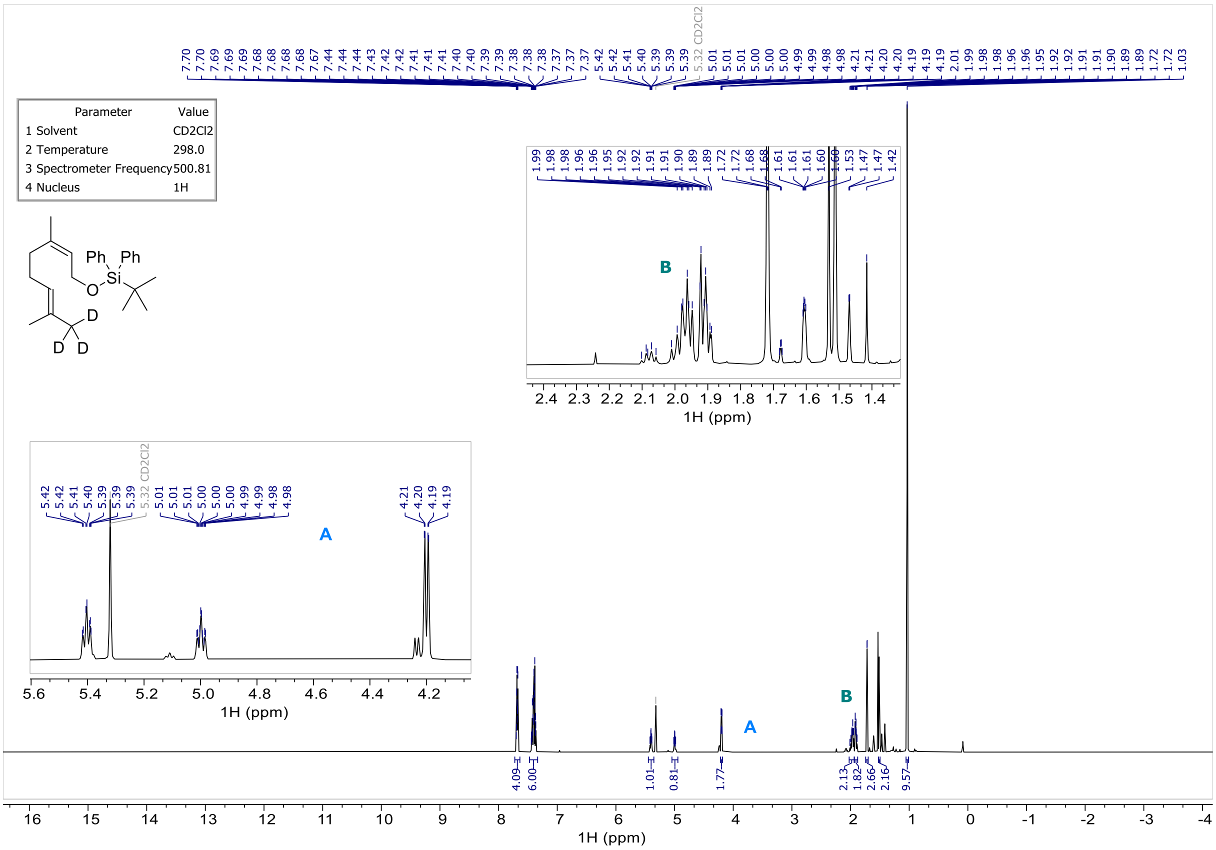

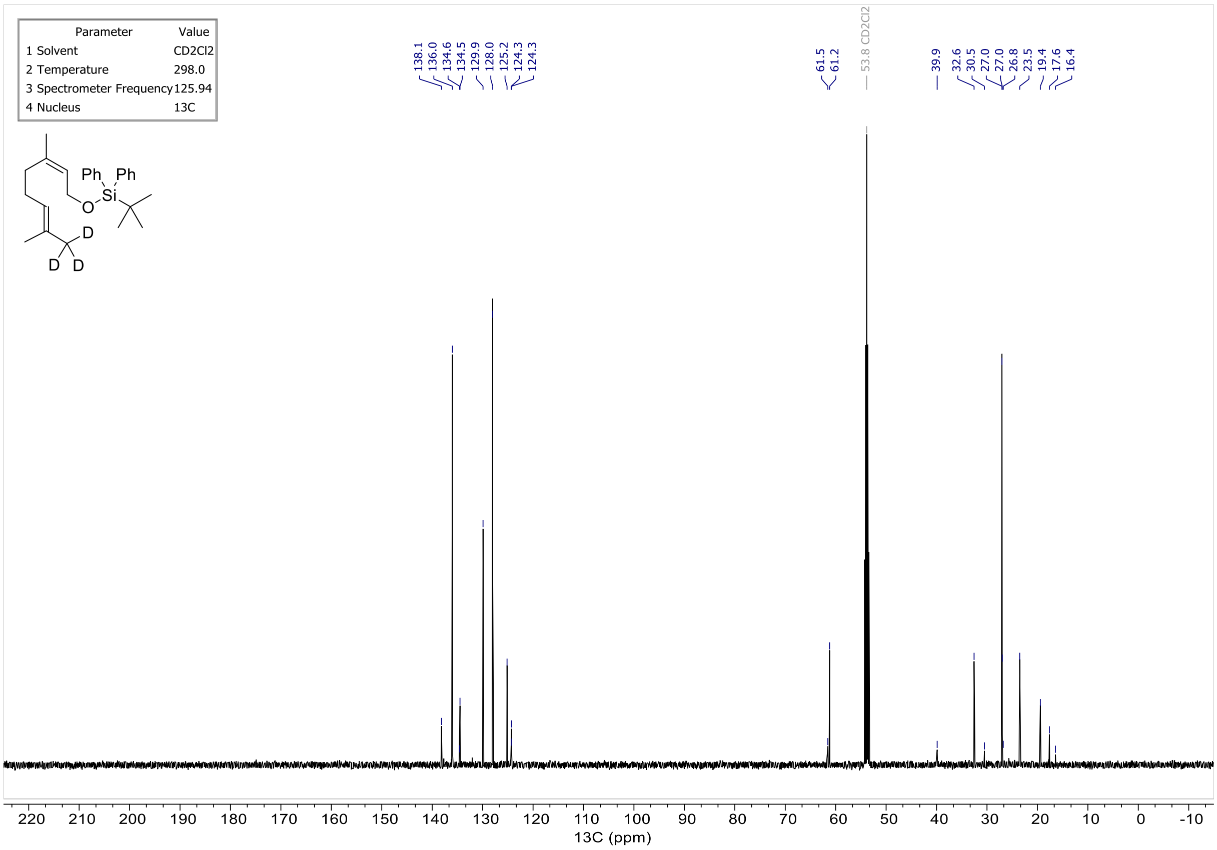

(2*Z*,6*E*)-3,7-dimethylocta-2,6-dienal-8,8,8-d_3 (**25**)

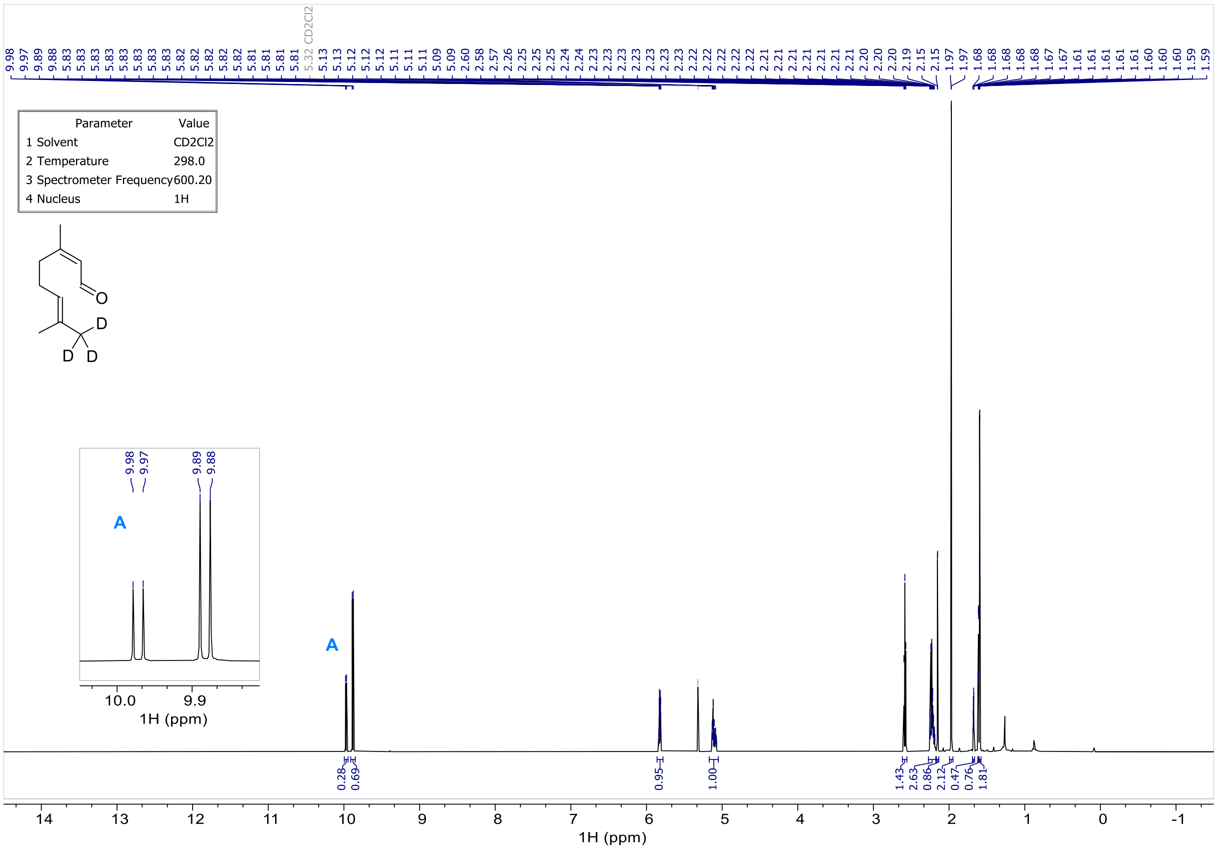

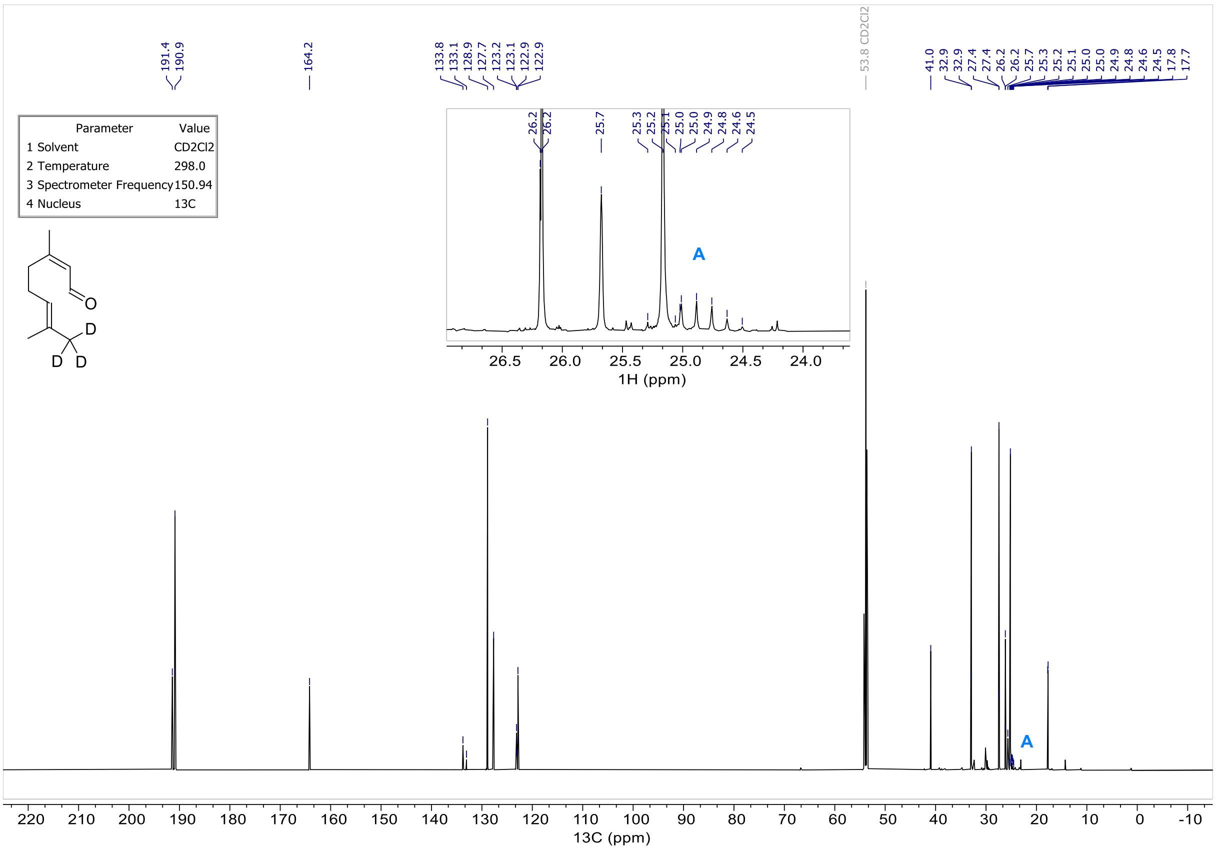
191.4
190.9
164.2
133.8
133.1
128.9
127.7
123.2
123.1
122.9
122.9
53.8 CD2Cl2
41.0
32.9
32.9
27.4
27.4
26.2
26.2
25.7
25.3
25.2
25.1
25.0
25.0
24.9
24.8
24.6
24.5
17.8
17.7
Parameter Value
1 Solvent CD2Cl2
2 Temperature 298.0
3 Spectrometer Frequency 150.94
4 Nucleus 13C
O
D
D D
A
1H (ppm)
13C (ppm)

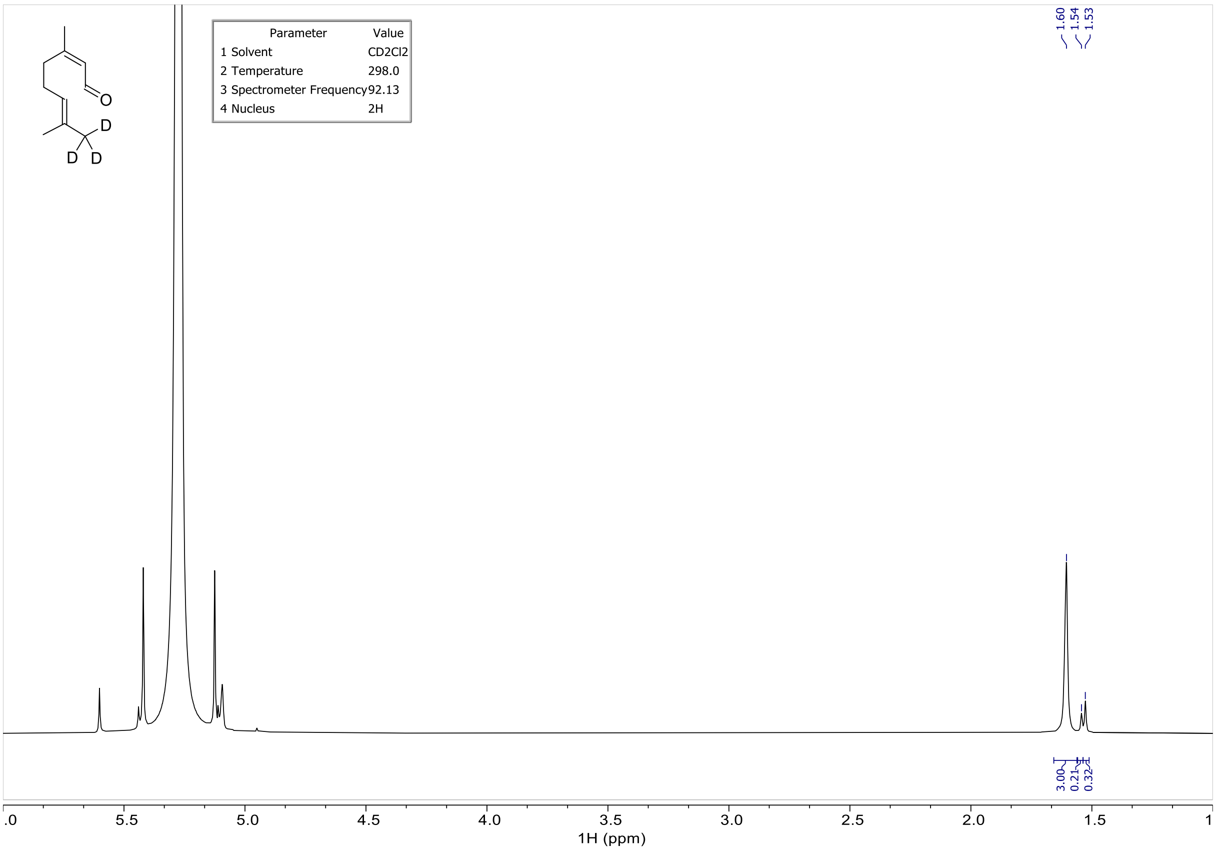
1.60
1.54
1.53
Parameter Value
1 Solvent CD2Cl2
2 Temperature 298.0
3 Spectrometer Frequency 92.13
4 Nucleus 2H
O
D
D D
3.00
0.21
0.32
1H (ppm)

1.4.2. Copies of HPLC traces

(*S*,*S*)-IDP **4**

LC-MS (50 mm Zorbax 300SB-C8, 4.6 mm i.d., MeCN / 1% TFA = 75:25, 1.0 mL/min, 5.9 MPa, 308 K, UV 254 nm): t_R = 5.65 (99.6%); m/z = 1684 ([M–H]$^-$).

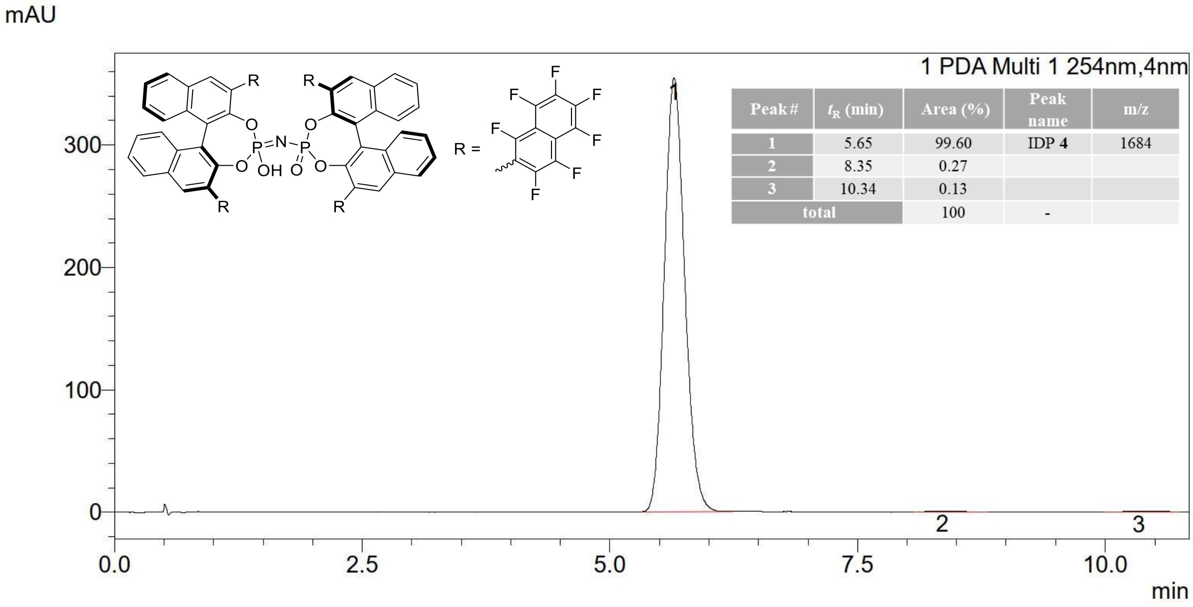

Peak#	t_R (min)	Area (%)	Peak name	m/z
1	5.65	99.60	IDP **4**	1684
2	8.35	0.27		
3	10.34	0.13		
total		100	-	

Note: No separation of the rotamers visible. A detailed explanation on the aspect of rotamers is discussed in chapter 2.1.

(*S*,*S*)-*i*IDP (**S7**)

LC-MS (50 mm Zorbax SB300-C8, 3.5 µm, 4.6 mm i.d., MeCN / 1% TFA = 70:30, 1.0 mL/min, 7.5 MPa, 308 K, UV 254 nm): t_R = 5.62 (97.78%), m/z = 1311 ([M–H]$^-$).

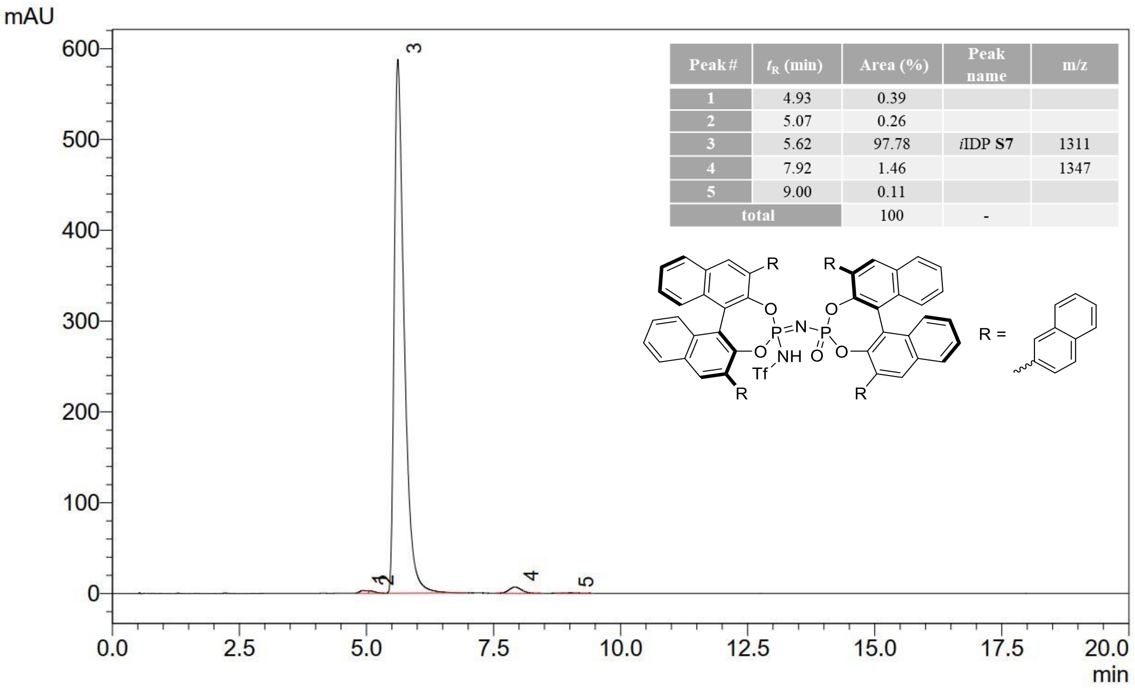

Peak#	t_R (min)	Area (%)	Peak name	m/z
1	4.93	0.39		
2	5.07	0.26		
3	5.62	97.78	*i*IDP **S7**	1311
4	7.92	1.46		1347
5	9.00	0.11		
total		100	-	

(*S*,*S*)-*i*IDP (**5**)

LC–MS (50 mm Zorbax 300SB-C8, 3.5 µm, 4.6 mm i.d.): MeCN / 1% TFA = 80:20, 1.0 mL/min, 5.2 MPa, 308 K, UV 220 nm.

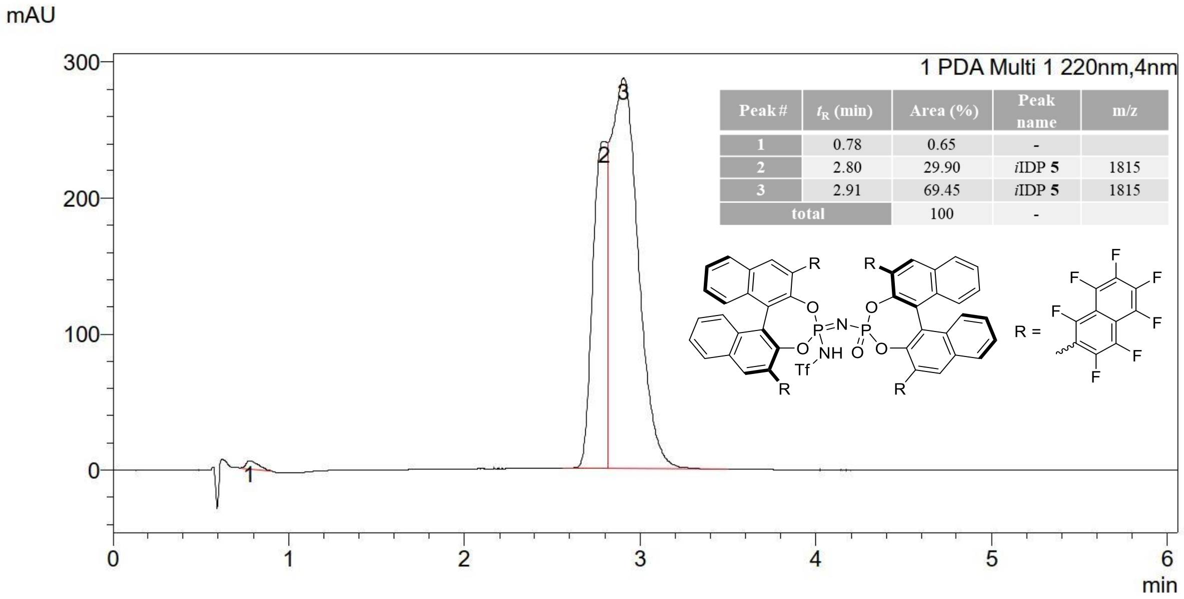

Peak#	t_R (min)	Area (%)	Peak name	m/z
1	0.78	0.65	-	
2	2.80	29.90	*i*IDP **5**	1815
3	2.91	69.45	*i*IDP **5**	1815
total		100	-	

Note: Two main rotamers can be differentiated. A detailed explanation on the aspect of rotamers is discussed in chapter 2.2.

(*S*,*S*)-IDPi (**6**)

LC-MS (50 mm Zorbax 300SB-C8, 4.6 mm i.d.): MeCN / 1% TFA = 80:20, 1.0 mL/min, 5.4 MPa, 308 K, UV 254 nm.

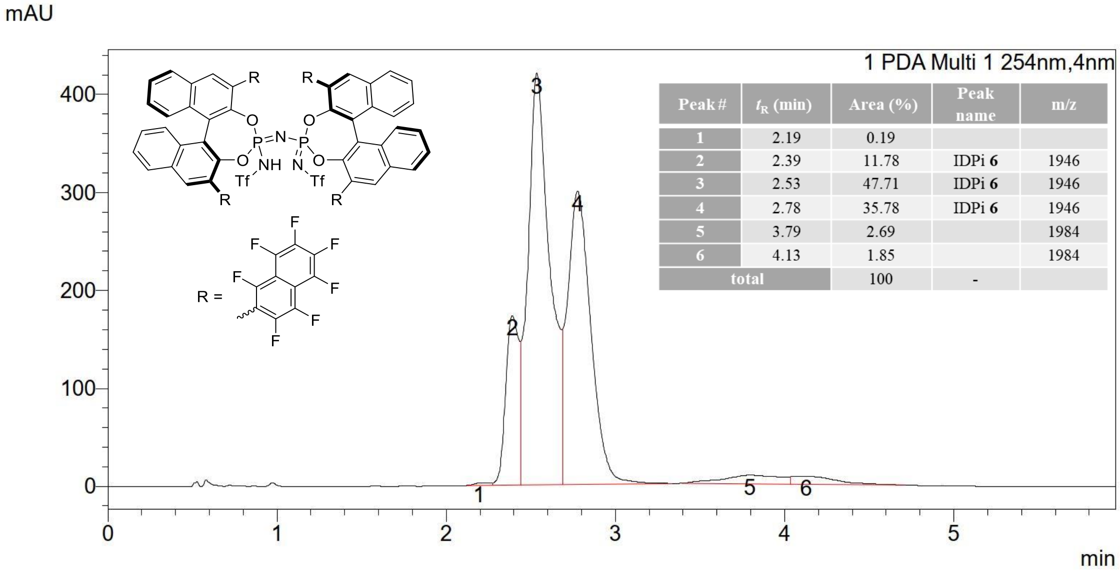

Peak#	t_R (min)	Area (%)	Peak name	m/z
1	2.19	0.19		
2	2.39	11.78	IDPi **6**	1946
3	2.53	47.71	IDPi **6**	1946
4	2.78	35.78	IDPi **6**	1946
5	3.79	2.69		1984
6	4.13	1.85		1984
total		100	-	

Note: Three main rotamers can be differentiated, A detailed explanation on the aspect of rotamers is discussed in chapter 2.2.

Cannabidiol (**10**)

HPLC (IG-3, heptane:*i*PrOH = 99:1, 1.0 mL/min, 298 K, 254 nm): t_{R1} = 15.1 min, t_{R2} = 19.7 min.

Racemate:

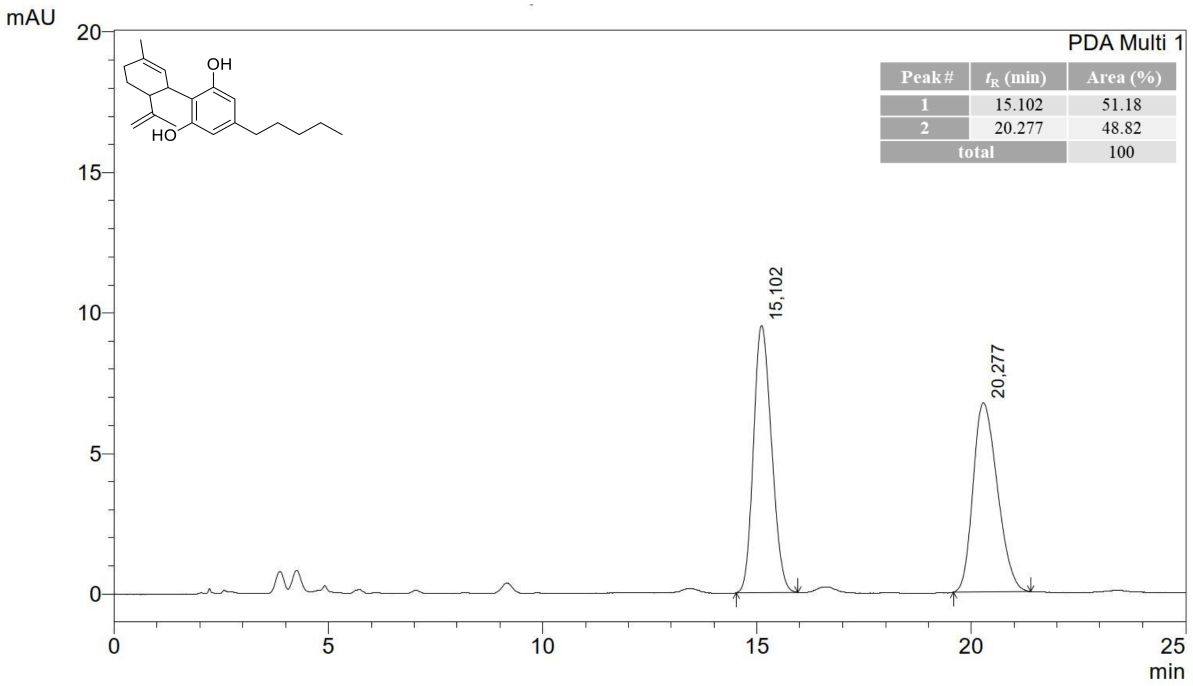

Enantioenriched compound:

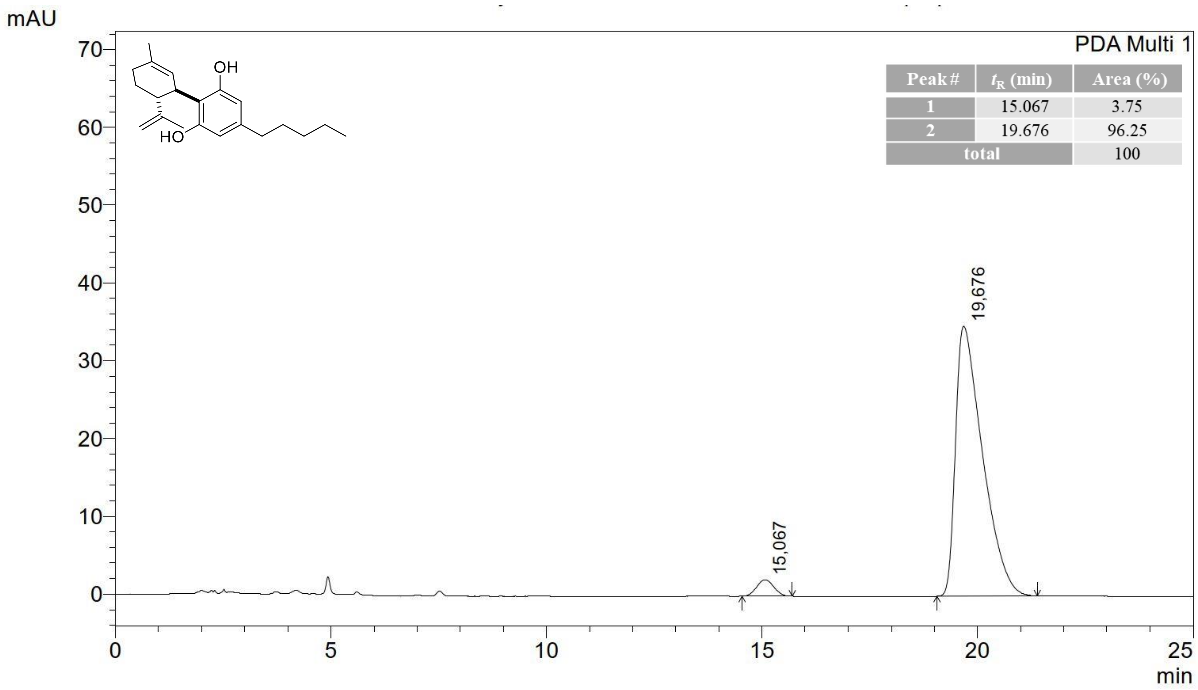

Methyl (1'*R*,2'*R*)-2,6-dihydroxy-5'-methyl-4-pentyl-2'-(prop-1-en-2-yl)-1',2',3',4'-tetrahydro-[1,1'-biphenyl]-3-carboxylate (**11**)

HPLC (AD-3, heptane:*i*PrOH = 99:1, 0.5 mL/min, 298 K, 254 nm): t_{R1} = 7.9 min, t_{R2} = 9.1 min.

Racemate:

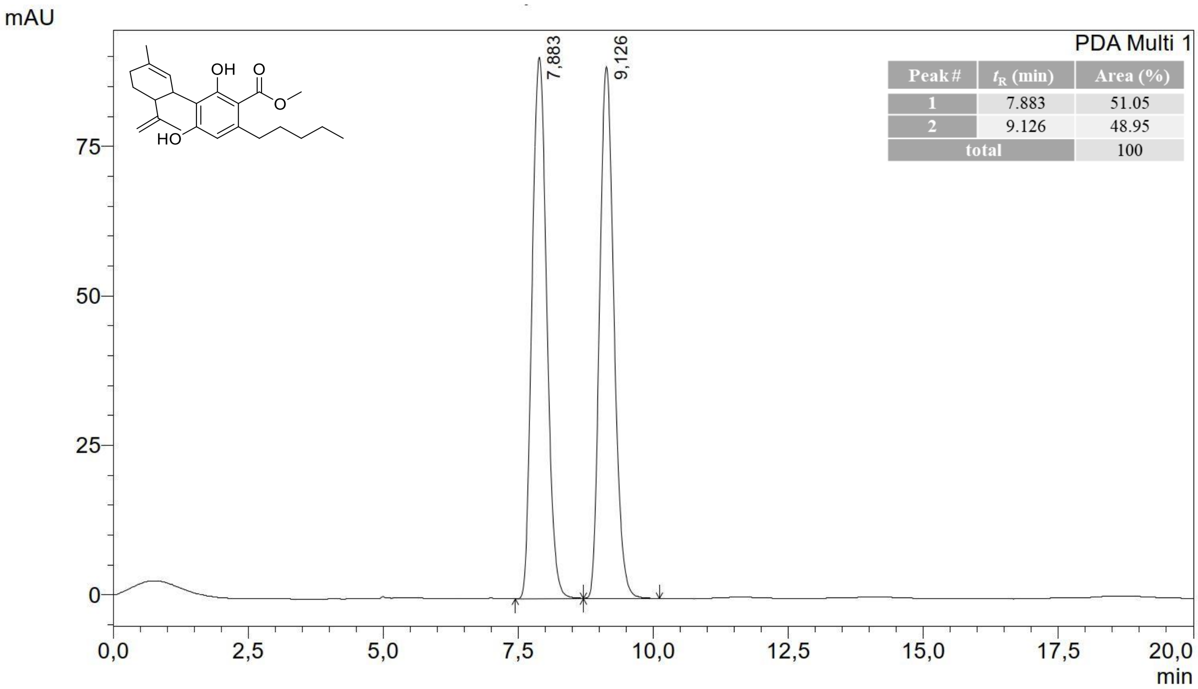

Enantioenriched compound:

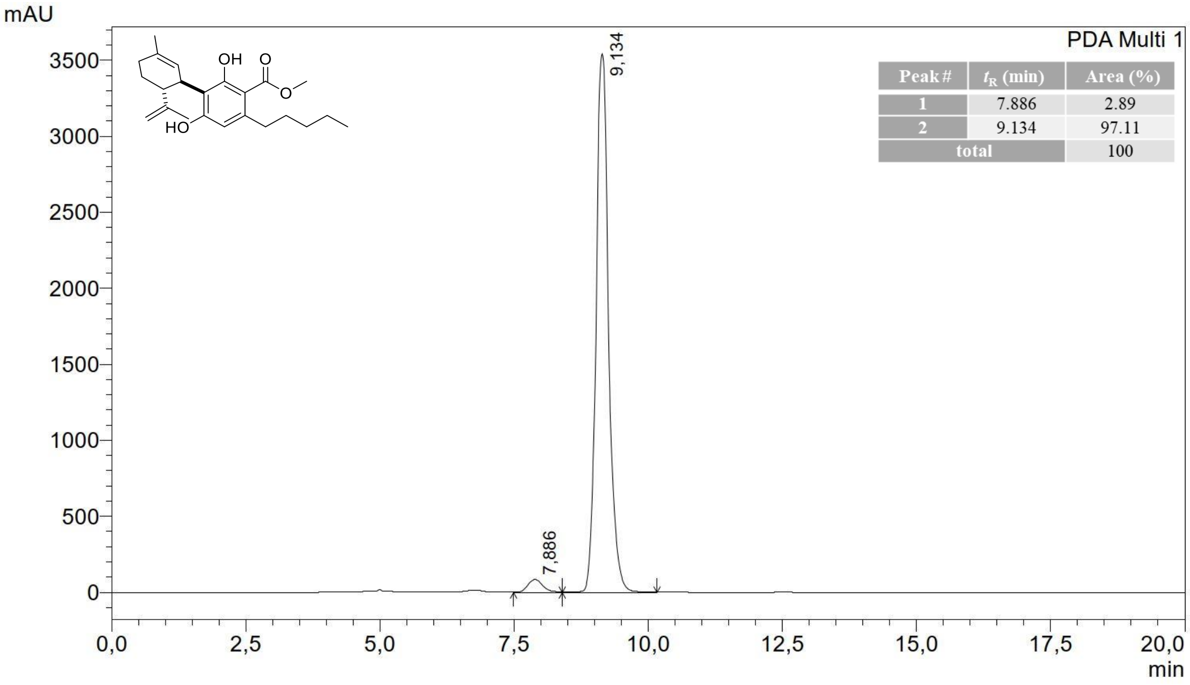

Δ^9-Tetrahydrocannabinol (**13**)

HPLC (OJ-3R, MeCN/H_2O = 60:40, 1 mL/min, 298 K, 210 nm): t_{R1} = 10.6 min, t_{R2} = 11.9 min.

Racemate:

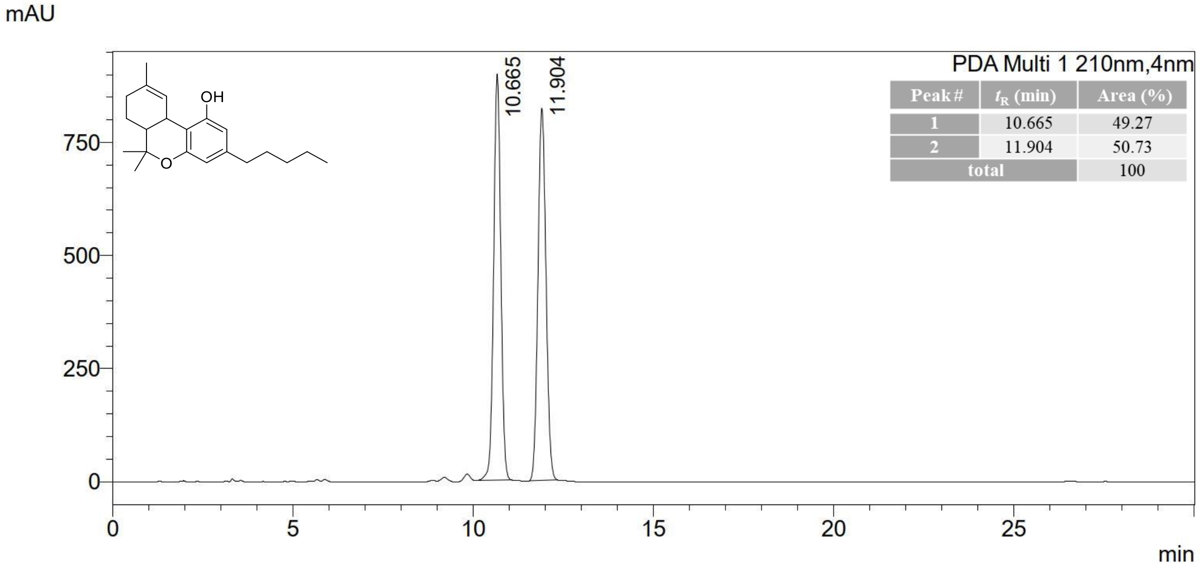

Enantioenriched compound:

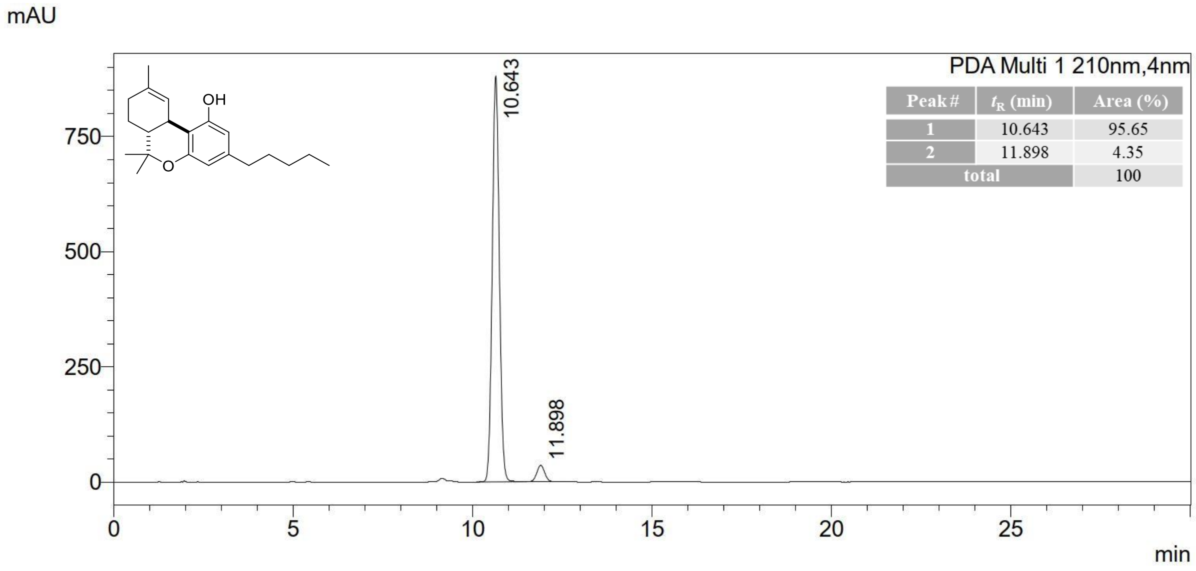

Δ^8-Tetrahydrocannabinol (**14**)

HPLC (AD-3, heptane:*i*PrOH = 98:2, 1 mL/min, 298 K, 254 nm): t_{R1} = 12.4 min, t_{R2} = 15.8 min.

Racemate:

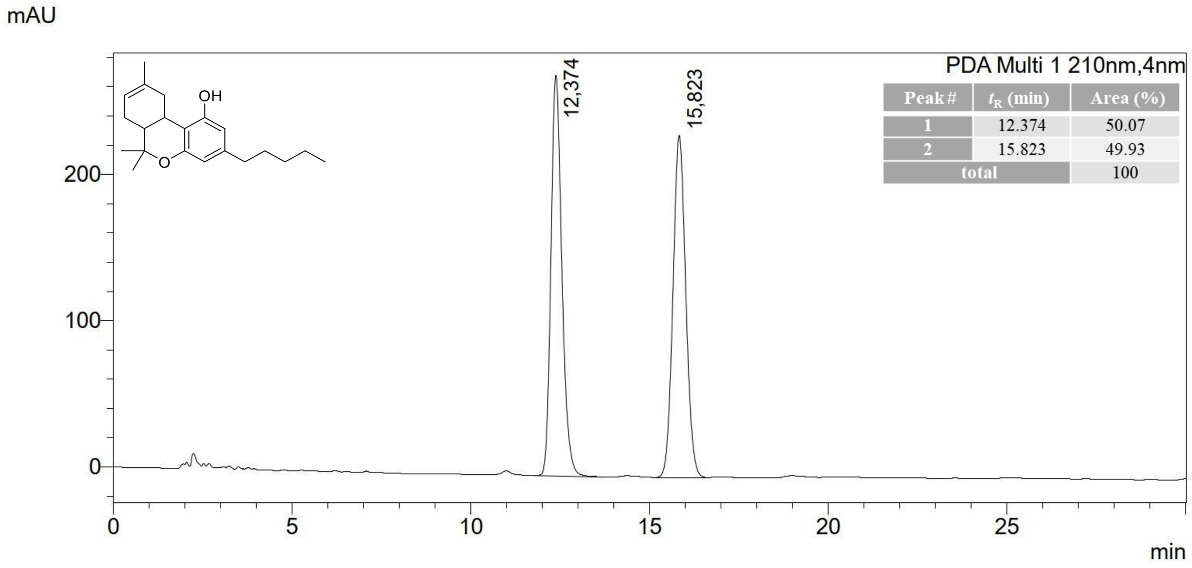

Enantioenriched compound:

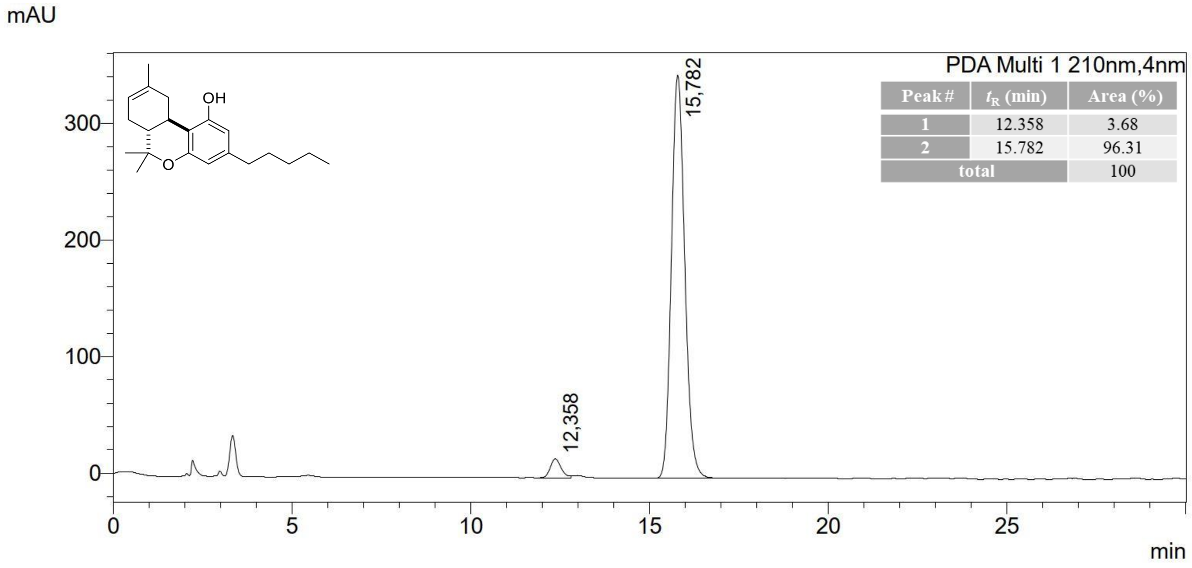

(10a*R*)-6,6,9-trimethyl-3-(2-methyloctan-2-yl)-6a,7,10,10a-tetrahydro-6H-benzo[c]chromen-1-ol (**15**)

HPLC (AD-3, heptane:*i*PrOH = 98:2, 1 mL/min, 298 K, 254 nm): t_{R1} = 6.1 min, t_{R2} = 6.8 min, e.r. = 96:4.

Racemate:

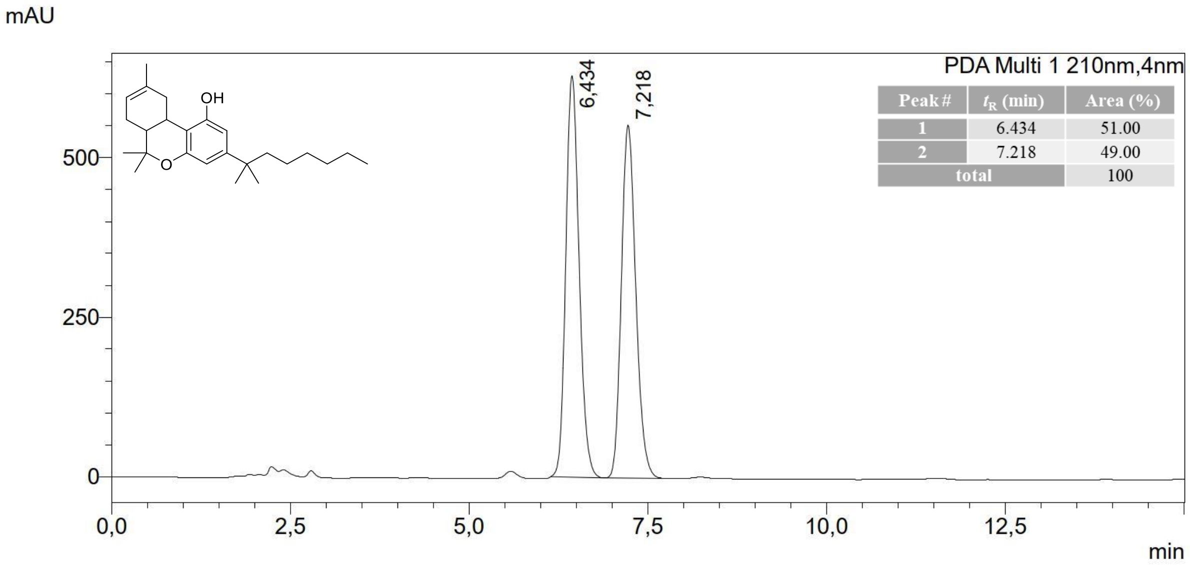

Enantioenriched compound:

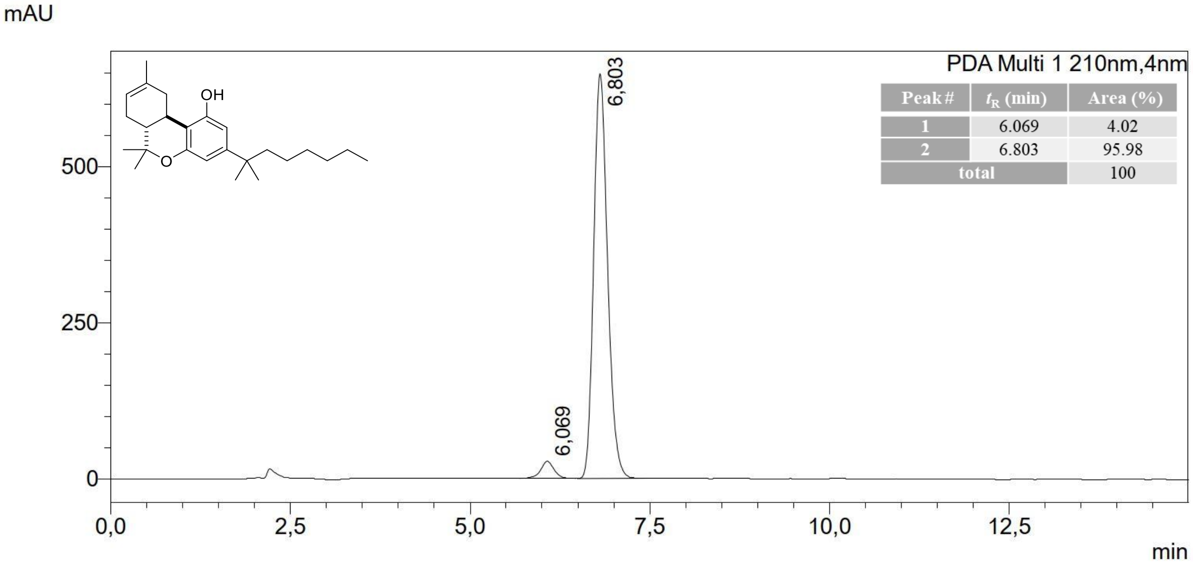

1.4.3. Copies of GC traces

isopiperitenol (*rac*-**2**)

Column: 30.0 m, HP-5 MS 0.25/0.5df; *temperature program*: 220/ 50 5/min 150 12/min 320, 5 min iso/350; *gas*: 0.50 bar (H_2).

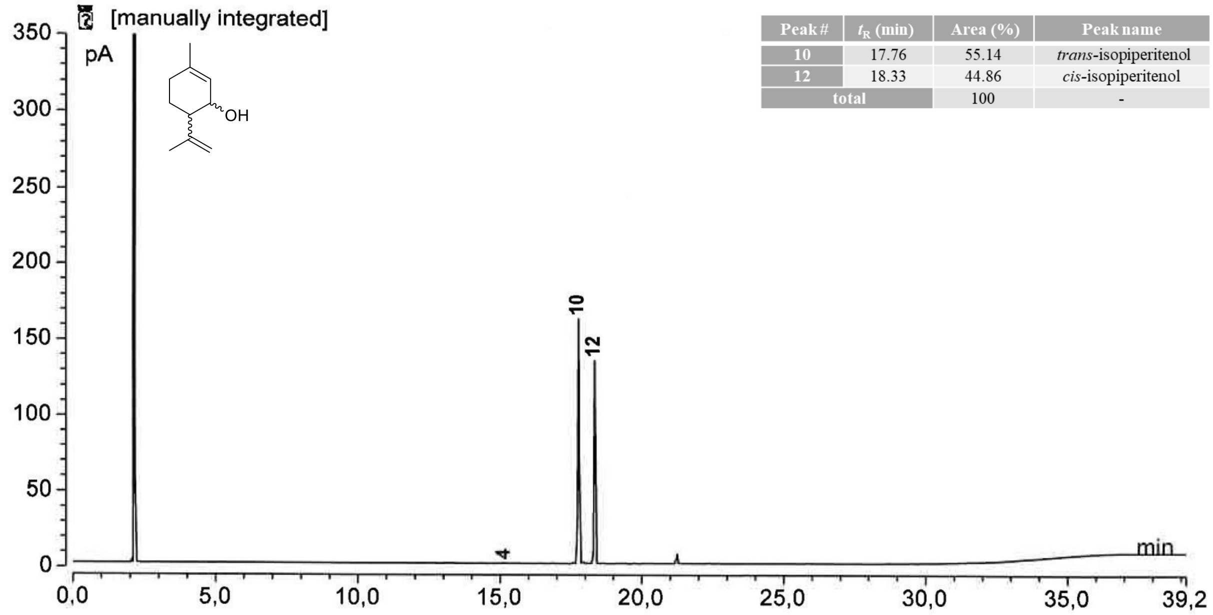

Peak#	t_R (min)	Area (%)	Peak name
10	17.76	55.14	*trans*-isopiperitenol
12	18.33	44.86	*cis*-isopiperitenol
total		100	-

Column: 25.0 m, Hydrodex-beta-TBDAc-CD 0.25/?df; *temperature program*: 220/ 95, 42 min iso 8/min 220, 3 min iso/350; *gas*: 0.40 bar (H_2).

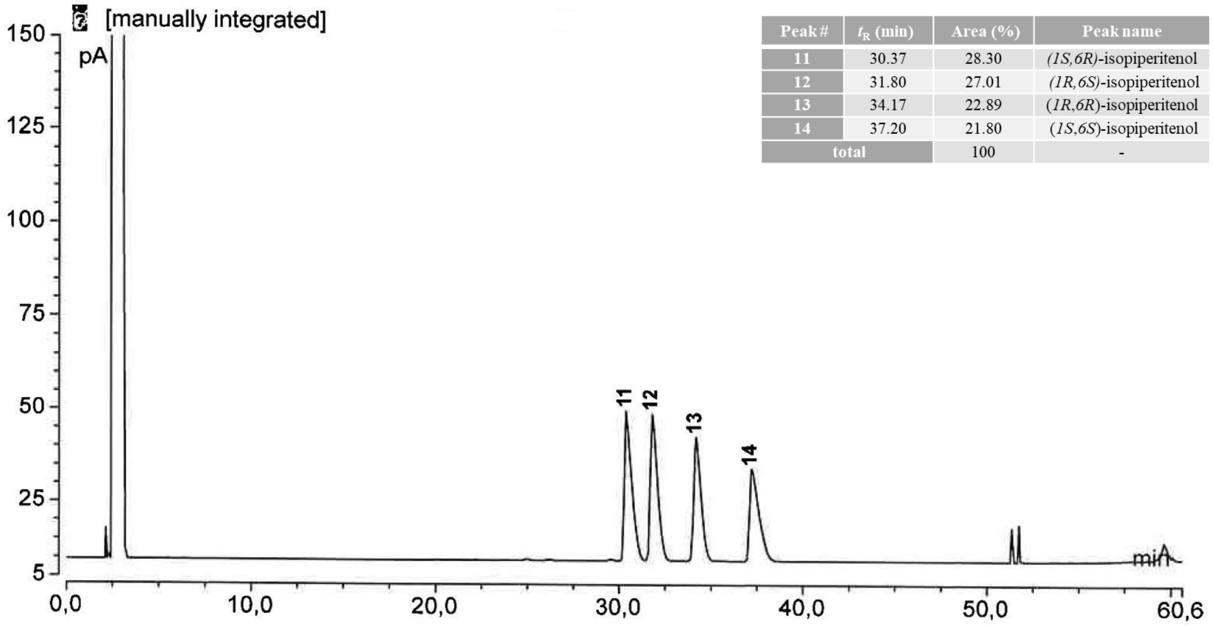

Peak#	t_R (min)	Area (%)	Peak name
11	30.37	28.30	*(1S,6R)*-isopiperitenol
12	31.80	27.01	*(1R,6S)*-isopiperitenol
13	34.17	22.89	(*1R,6R*)-isopiperitenol
14	37.20	21.80	(*1S,6S*)-isopiperitenol
total		100	-

(6*S*)-isopiperitenol ((6*S*)-**2**)

Column: 30.0 m, HP-5 MS 0.25/0.5df; *temperature program*: 220/ 50 5/min 150 12/min 320, 5 min iso/350; *gas*: 0.50 bar (H_2).

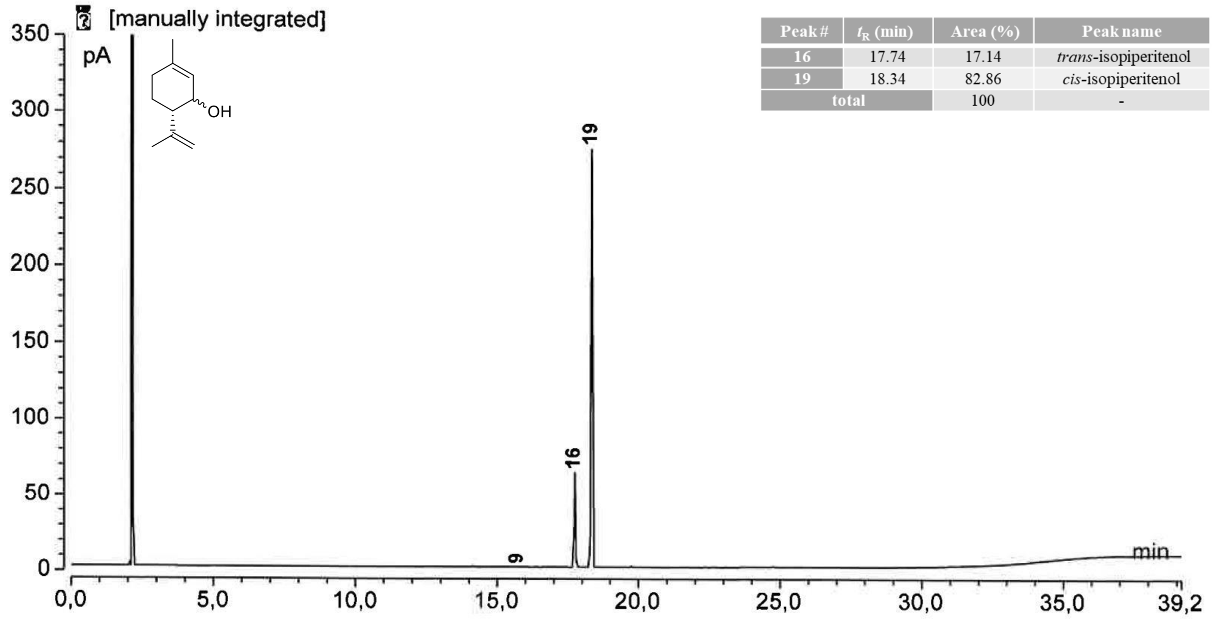

Peak#	t_R (min)	Area (%)	Peak name
16	17.74	17.14	*trans*-isopiperitenol
19	18.34	82.86	*cis*-isopiperitenol
total		100	-

Column: 25.0 m, Hydrodex-beta-TBDAc-CD 0.25/?df; *temperature program*: 220/ 95, 42 min iso 8/min 220, 3 min iso/350; *gas*: 0.40 bar (H_2).

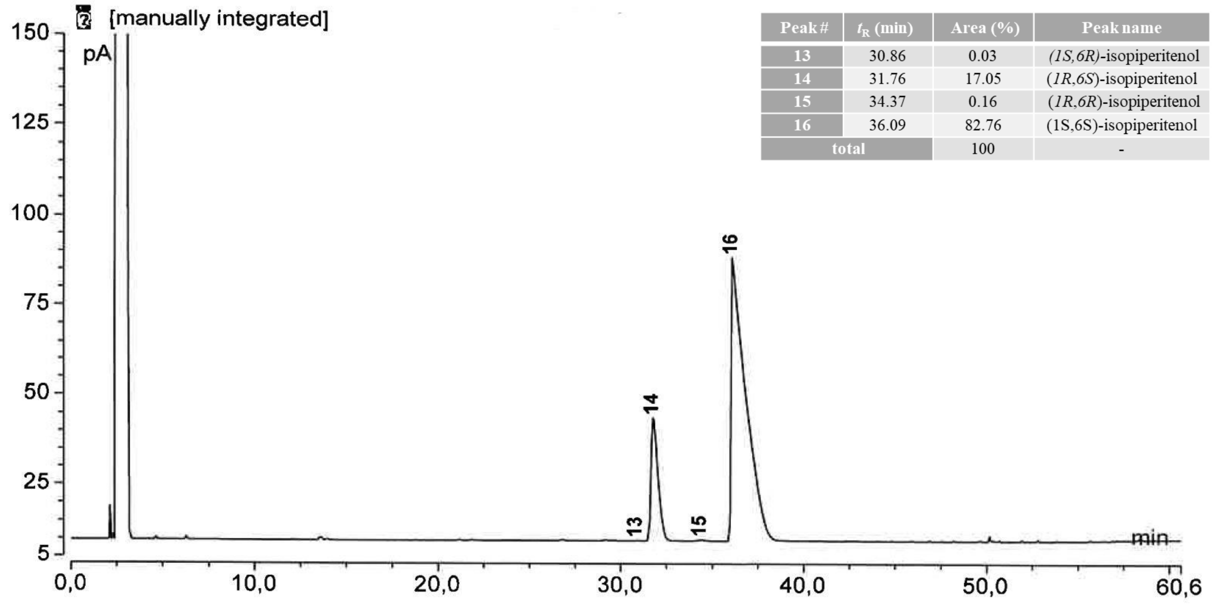

Peak#	t_R (min)	Area (%)	Peak name
13	30.86	0.03	*(1S,6R)*-isopiperitenol
14	31.76	17.05	(*1R,6S*)-isopiperitenol
15	34.37	0.16	(*1R,6R*)-isopiperitenol
16	36.09	82.76	(1S,6S)-isopiperitenol
total		100	-

(6*R*)-isopiperitenol ((6*R*)-**2**)

Column: 30.0 m, HP-5 MS 0.25/0.5df; *temperature program*: 220/ 50 5/min 150 12/min 320, 5 min iso/350; *gas*: 0.50 bar (H_2).

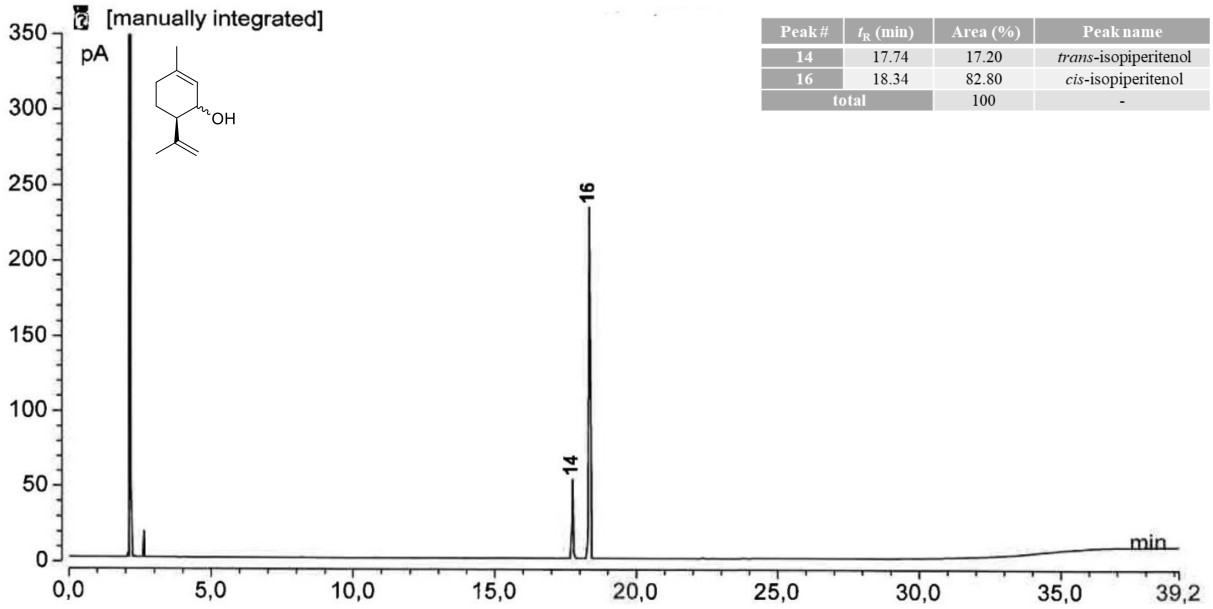

Peak#	t_R (min)	Area (%)	Peak name
14	17.74	17.20	*trans*-isopiperitenol
16	18.34	82.80	*cis*-isopiperitenol
total		100	-

Column: 25.0 m, Hydrodex-beta-TBDAc-CD 0.25/?df; *temperature program*: 220/ 95, 42 min iso 8/min 220, 3 min iso/350; *gas*: 0.40 bar (H_2).

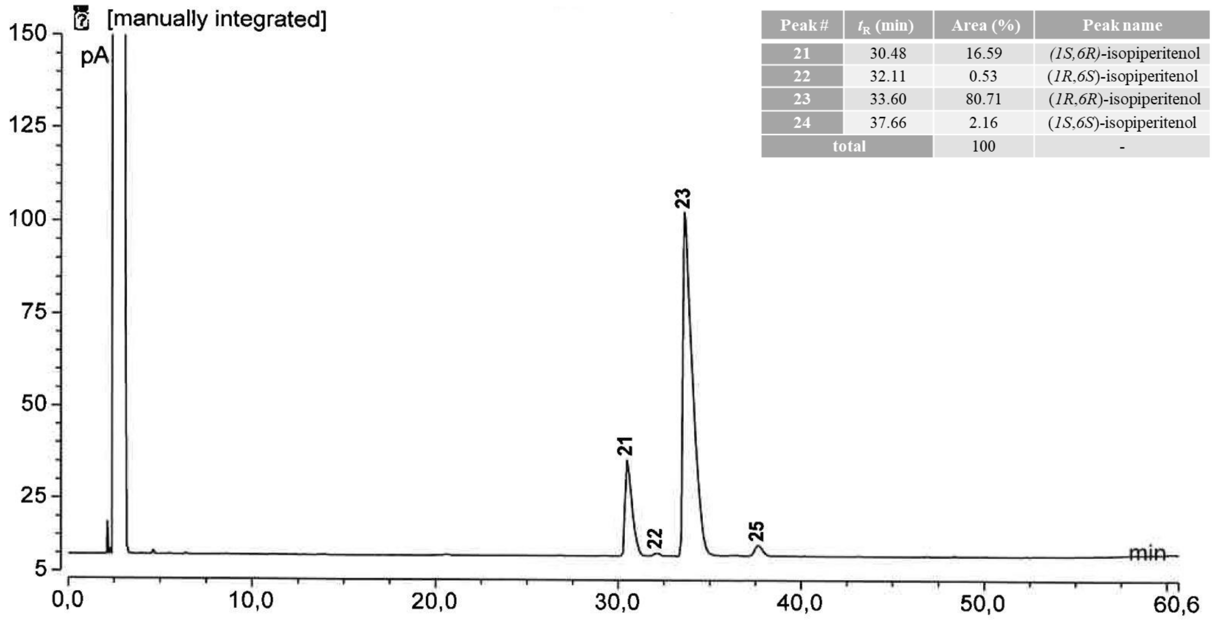

Peak#	t_R (min)	Area (%)	Peak name
21	30.48	16.59	*(1S,6R)*-isopiperitenol
22	32.11	0.53	(*1R,6S*)-isopiperitenol
23	33.60	80.71	(*1R,6R*)-isopiperitenol
24	37.66	2.16	(*1S,6S*)-isopiperitenol
total		100	-

Chiral GC-comparison of rac-isopiperitenol, (6S)-isopiperitenol, (6R)-isopiperitenol and isopiperitenol obtained from catalytic reaction

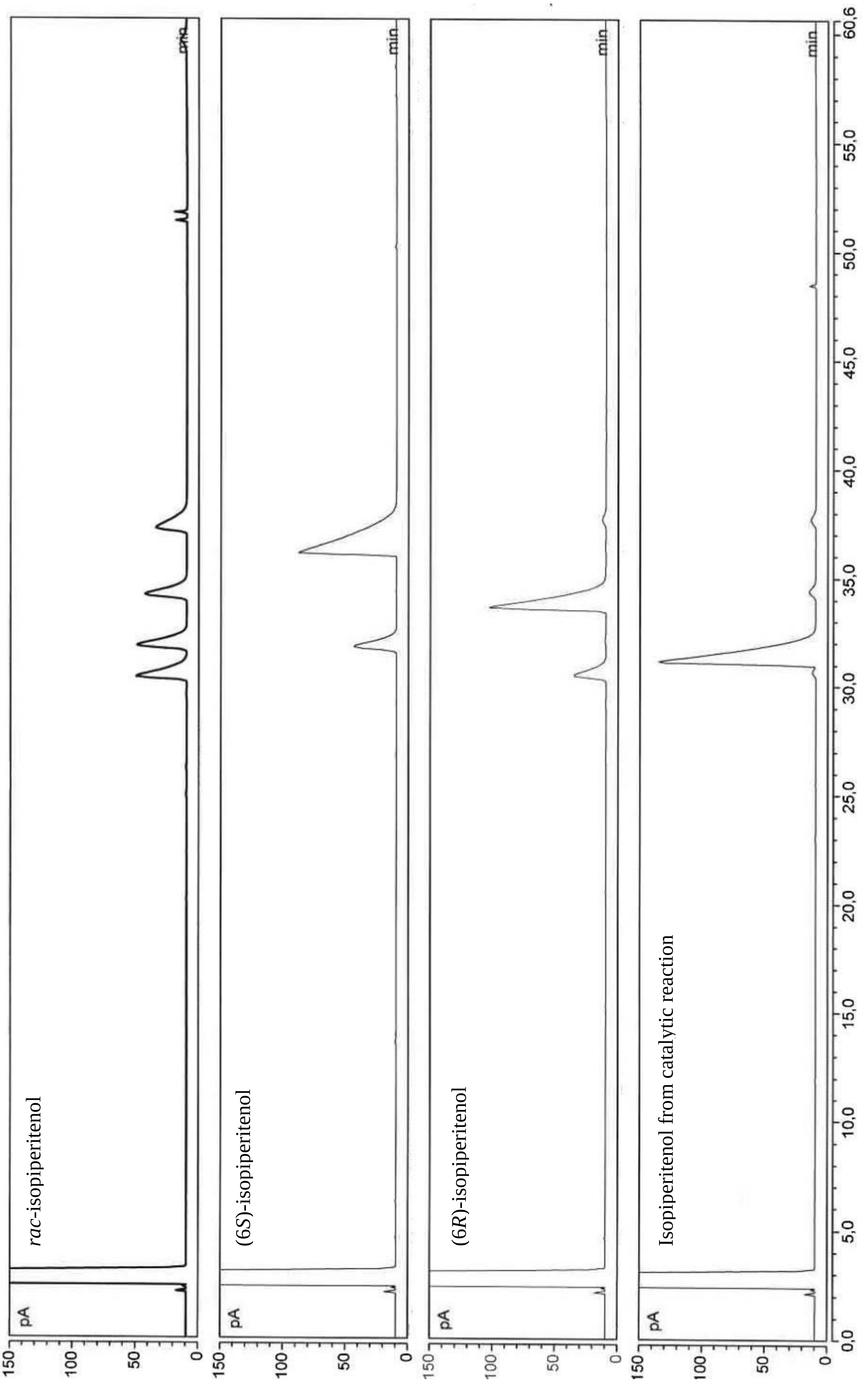

isopiperitenol (**2**)

Note: This sample was obtained from 0.029 mmol scale reaction and the main diastereoisomer and major enantiomer is depicted.

Column: 24.3 m, RTX-5 0.25/0.5df; *temperature program*: 220/ 50 5/min 150 12/min 320, 5 min iso/350; *gas*: 0.40 bar (H_2).

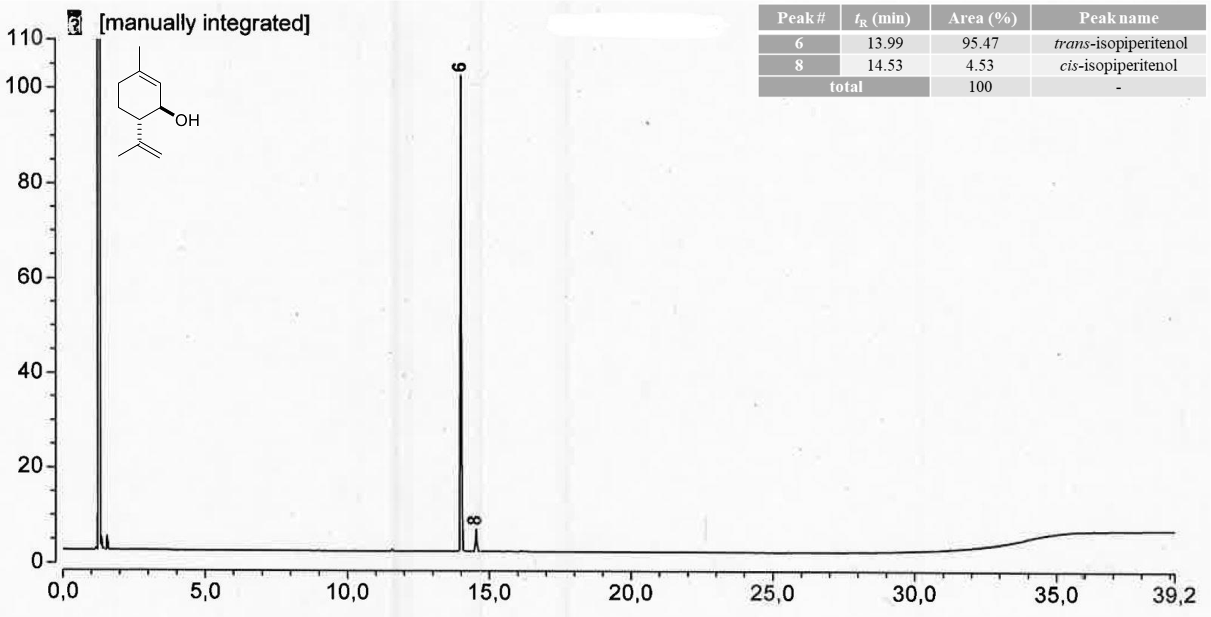

Peak #	t_R (min)	Area (%)	Peak name
6	13.99	95.47	*trans*-isopiperitenol
8	14.53	4.53	*cis*-isopiperitenol
total		100	-

Column: 25.0 m, Hydrodex-beta-TBDAc-CD 0.25/?df; *temperature program*: 220/ 95, 42 min iso 8/min 220, 3 min iso/350; *gas*: 0.40 bar (H_2).

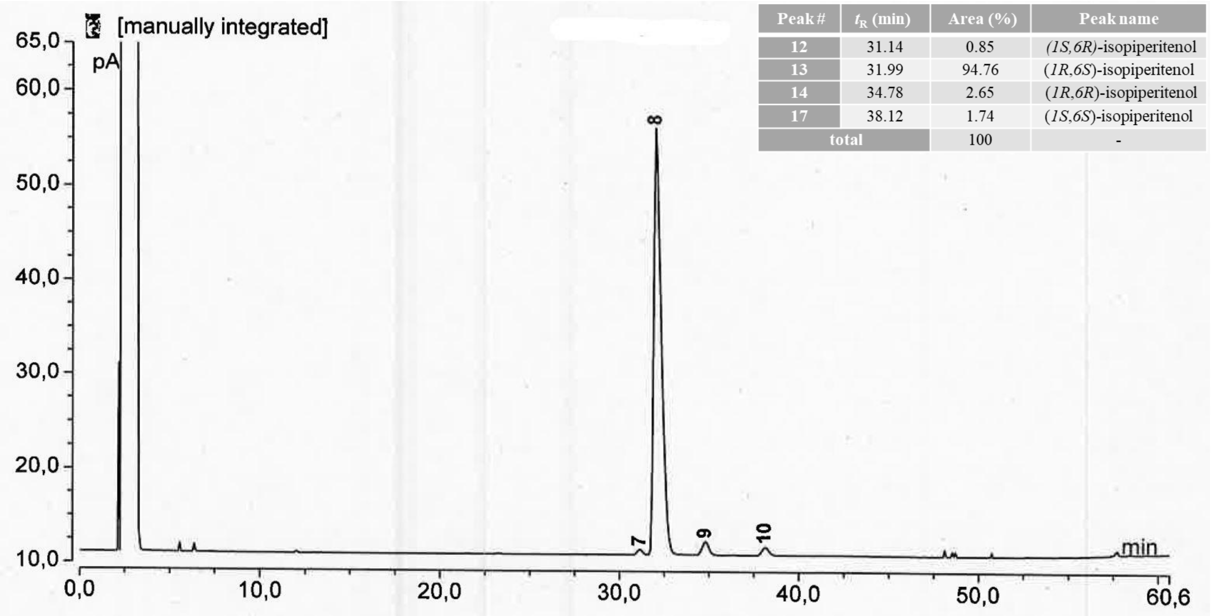

Peak #	t_R (min)	Area (%)	Peak name
12	31.14	0.85	*(1S,6R)*-isopiperitenol
13	31.99	94.76	(*1R,6S*)-isopiperitenol
14	34.78	2.65	(*1R,6R*)-isopiperitenol
17	38.12	1.74	(*1S,6S*)-isopiperitenol
total		100	-

isopiperitenol (**2**)

Note: This sample was obtained from up-scaling experiments in PhMe and the main diastereoisomer and major enantiomer is depicted.

Column: 30.0 m, HP-5 MS 0.25/0.5df; *temperature program*: 220/ 50 5/min 150 12/min 320, 5 min iso/350; *gas*: 0.50 bar (H_2).

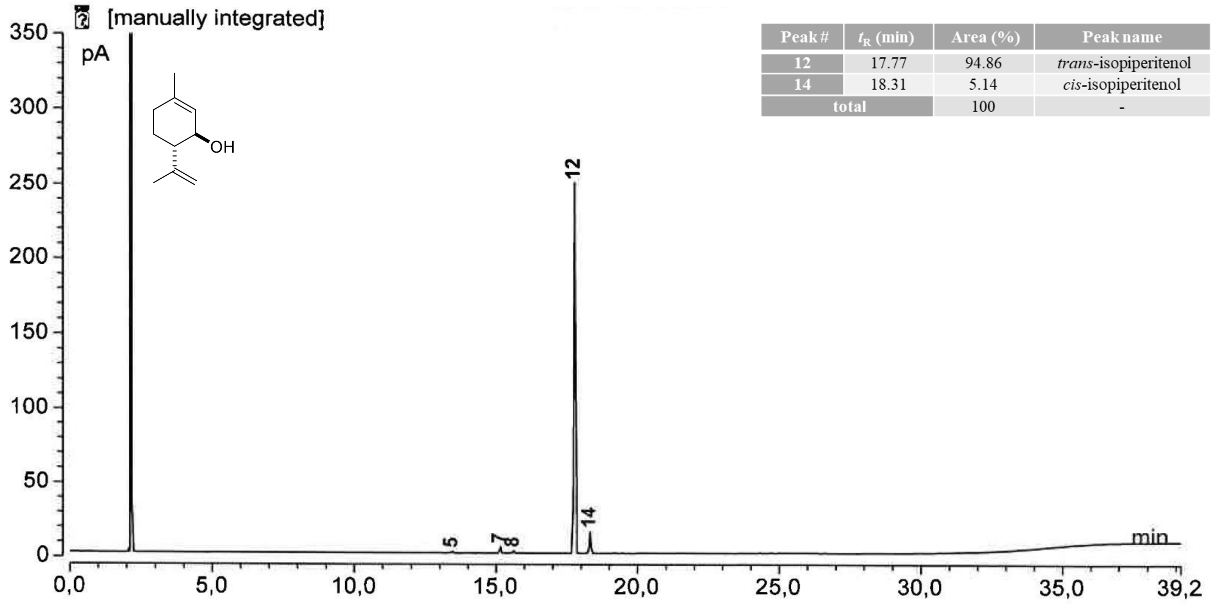

Peak#	t_R (min)	Area (%)	Peak name
12	17.77	94.86	*trans*-isopiperitenol
14	18.31	5.14	*cis*-isopiperitenol
total		100	-

Column: 25.0 m, Hydrodex-beta-TBDAc-CD 0.25/?df; *temperature program*: 220/ 95, 42 min iso 8/min 220, 3 min iso/350; *gas*: 0.40 bar (H_2).

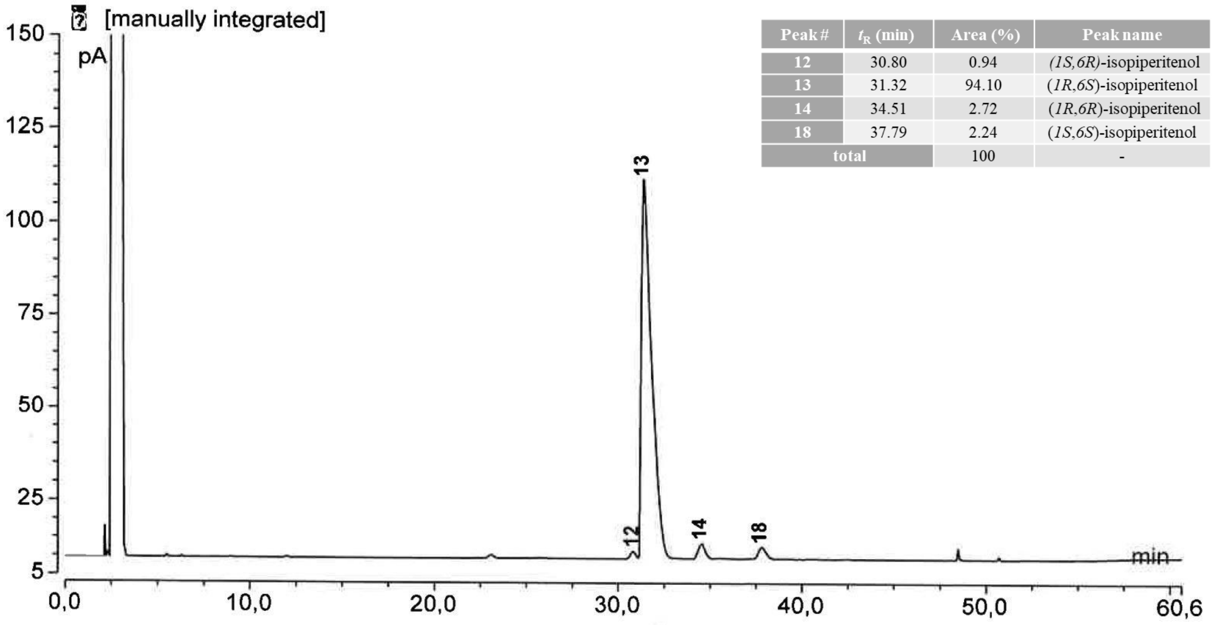

Peak#	t_R (min)	Area (%)	Peak name
12	30.80	0.94	*(1S,6R)*-isopiperitenol
13	31.32	94.10	(*1R,6S*)-isopiperitenol
14	34.51	2.72	(*1R,6R*)-isopiperitenol
18	37.79	2.24	(*1S,6S*)-isopiperitenol
total		100	-

menthol (**7**)

Note: This sample was obtained from hydrogenation of a mixture of (6S)-isopiperitenol and (6R)-isopiperitenol (2:1).

Column: 30.0 m, DB-1 0.25/0.25df; *temperature program*: 220/ 50 2/min 110 12/min 320, 5 min iso/350; *gas*: 0.60 bar (H_2).

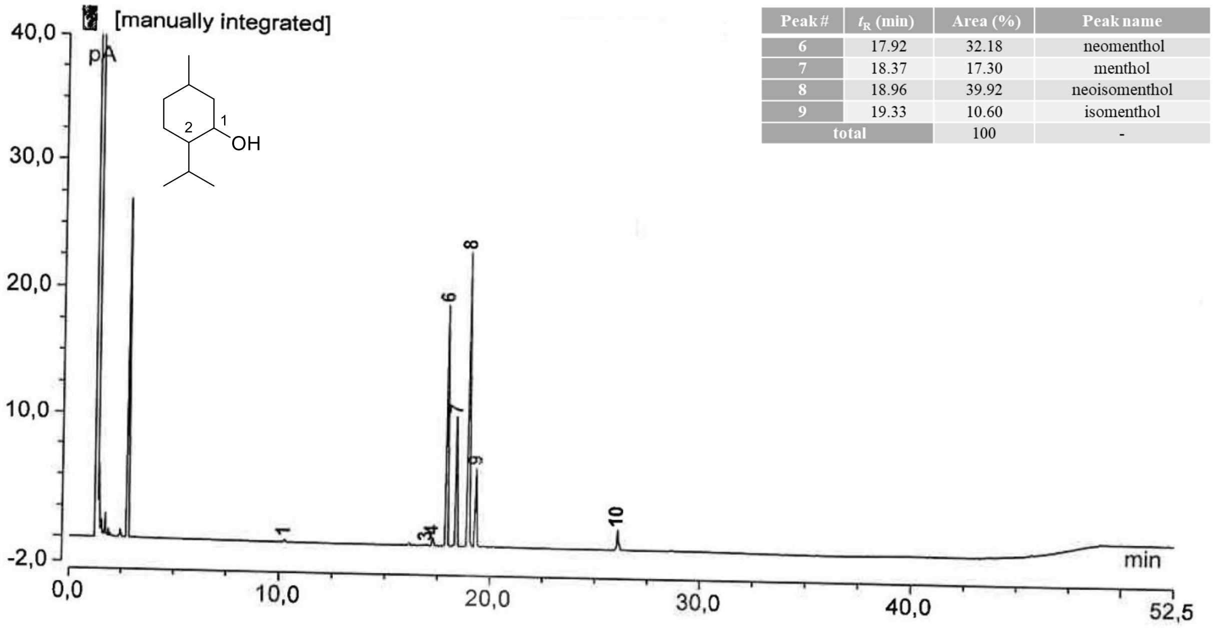

Peak #	t_R (min)	Area (%)	Peak name
6	17.92	32.18	neomenthol
7	18.37	17.30	menthol
8	18.96	39.92	neoisomenthol
9	19.33	10.60	isomenthol
total		100	-

Column: 30.0 m, BGB-176/BGB-15 0.25/0.25df; *temperature program*: 220/ 80 iso/ 350; *gas*: 0.60 bar (H_2).

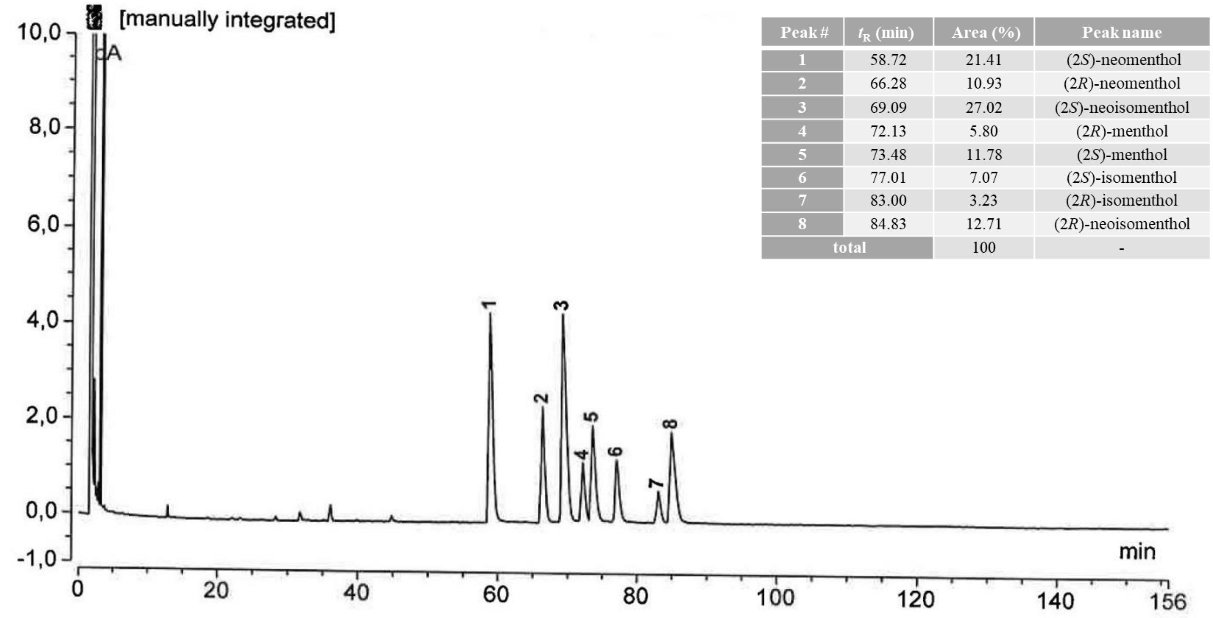

Peak #	t_R (min)	Area (%)	Peak name
1	58.72	21.41	(2*S*)-neomenthol
2	66.28	10.93	(2*R*)-neomenthol
3	69.09	27.02	(2*S*)-neoisomenthol
4	72.13	5.80	(2*R*)-menthol
5	73.48	11.78	(2*S*)-menthol
6	77.01	7.07	(2*S*)-isomenthol
7	83.00	3.23	(2*R*)-isomenthol
8	84.83	12.71	(2*R*)-neoisomenthol
total		100	-

menthol (**7**)

Note: This sample was obtained from selective hydrogenation of (1R,6S)-isopiperitenol obtained from upscaling reaction.

Column: 30.0 m, DB-1 0.25/0.25df; *temperature program*: 220/ 50 2/min 110 12/min 320, 5 min iso/350; *gas*: 0.60 bar (H_2).

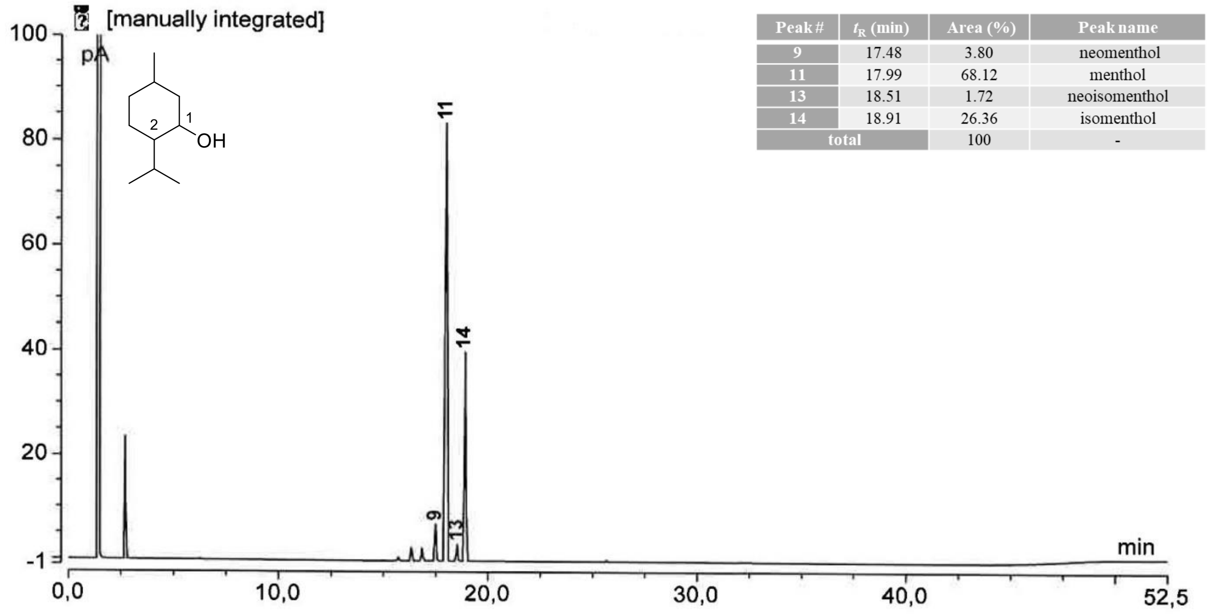

Peak #	t_R (min)	Area (%)	Peak name
9	17.48	3.80	neomenthol
11	17.99	68.12	menthol
13	18.51	1.72	neoisomenthol
14	18.91	26.36	isomenthol
total		100	-

Column: 30.0 m, BGB-176/BGB-15 0.25/0.25df; *temperature program*: 220/ 80 iso/ 350; *gas*: 0.60 bar (H_2).

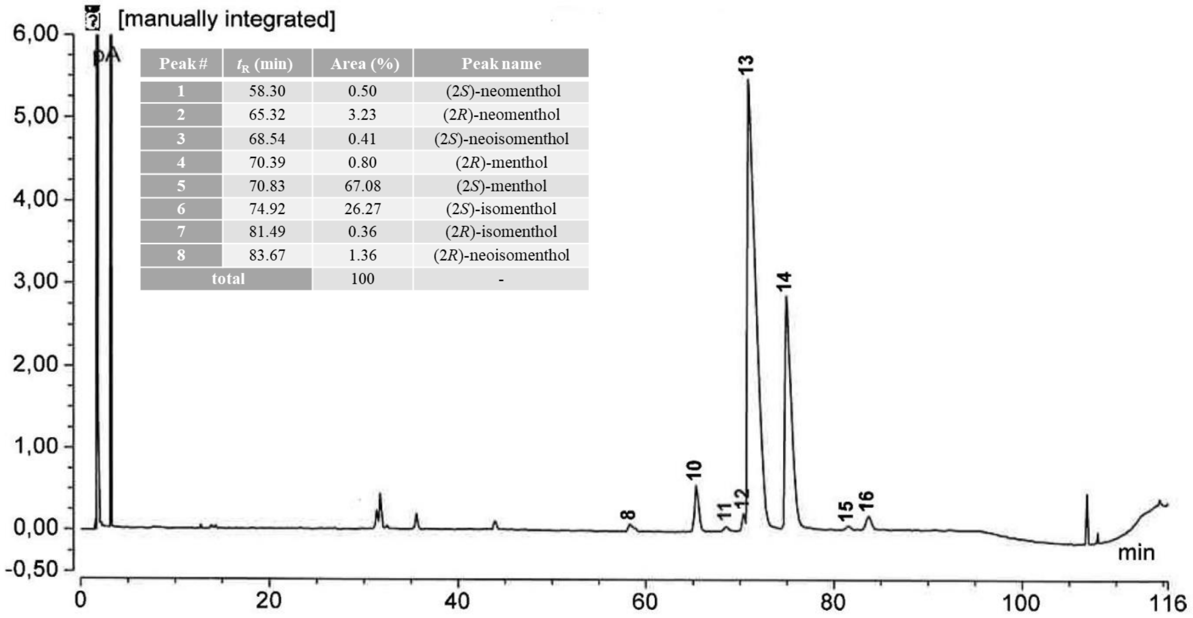

Peak #	t_R (min)	Area (%)	Peak name
1	58.30	0.50	(2*S*)-neomenthol
2	65.32	3.23	(2*R*)-neomenthol
3	68.54	0.41	(2*S*)-neoisomenthol
4	70.39	0.80	(2*R*)-menthol
5	70.83	67.08	(2*S*)-menthol
6	74.92	26.27	(2*S*)-isomenthol
7	81.49	0.36	(2*R*)-isomenthol
8	83.67	1.36	(2*R*)-neoisomenthol
total		100	-

piperitol (**9**)

Note: This sample was obtained from selective hydrogenation of (rac)-isopiperitenol.

Column: 30.0 m, DB-1 0.25/0.25df; *temperature program*: 220/60 3/min 120 12/min 320/350; *gas*: 0.50 bar (H_2).

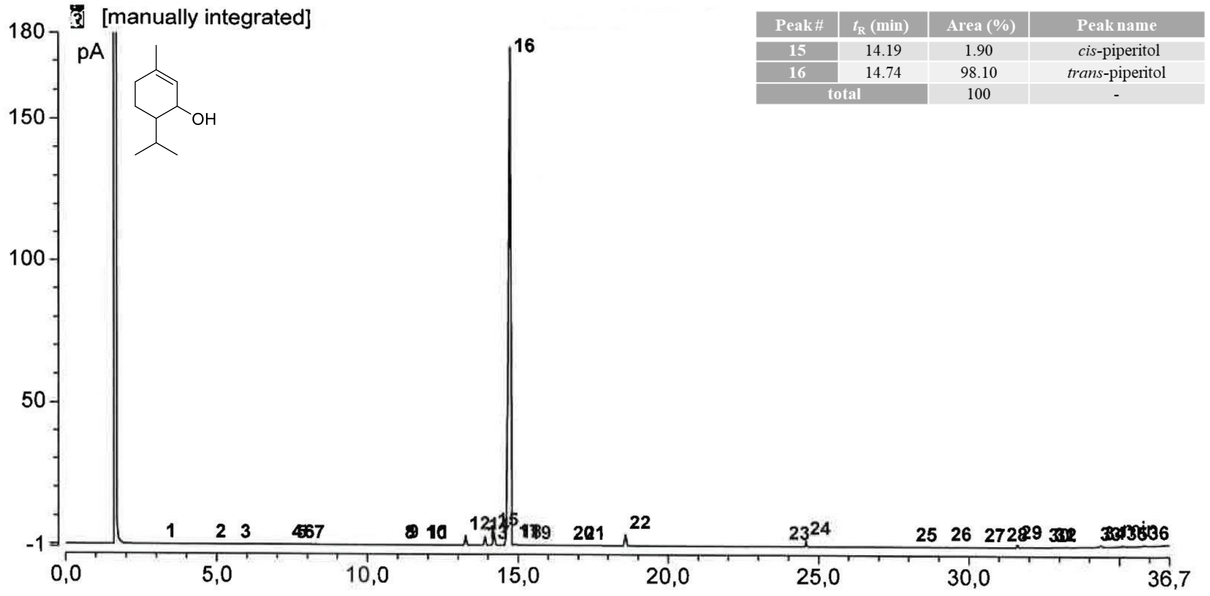

Peak#	t_R (min)	Area (%)	Peak name
15	14.19	1.90	*cis*-piperitol
16	14.74	98.10	*trans*-piperitol
total		100	-

Column: 25.0 m, Hydrodex-gamma-TBDAc-CD 0.25/?df; *temperature program*: 220/90 iso/350; *gas*: 0.50 bar (H_2).

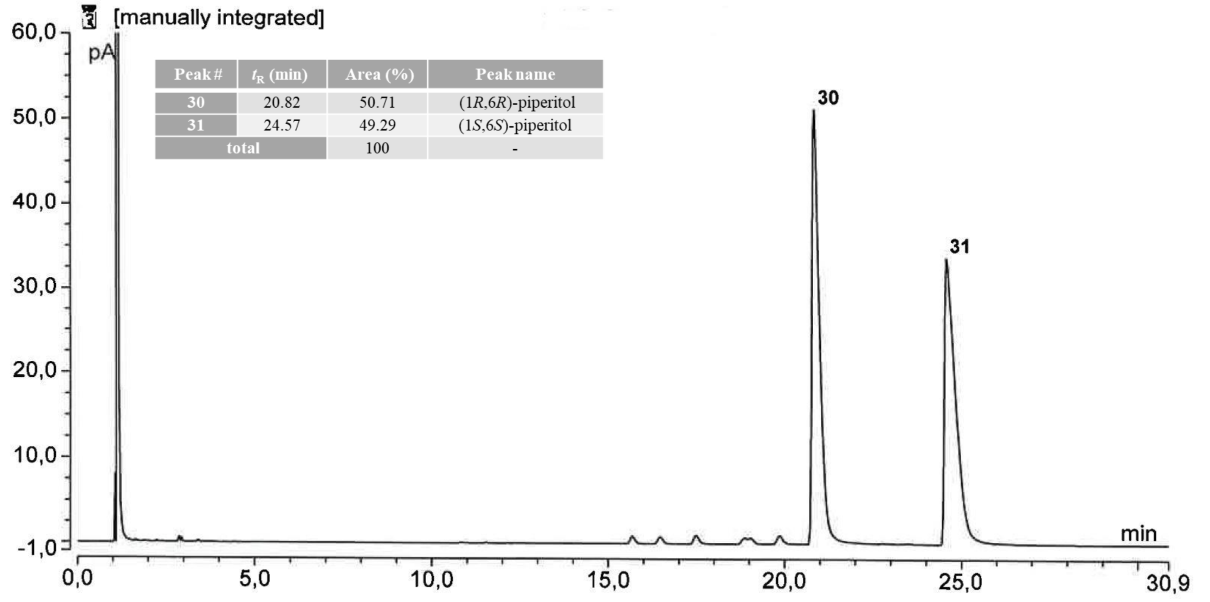

Peak#	t_R (min)	Area (%)	Peak name
30	20.82	50.71	(1*R*,6*R*)-piperitol
31	24.57	49.29	(1*S*,6*S*)-piperitol
total		100	-

piperitol (**9**)

Note: This sample was obtained from selective hydrogenation of (1R,6S)-isopiperitenol obtained from upscaling reaction.

Column: 30.0 m, DB-1 0.25/0.25df; *temperature program*: 220/60 3/min 120 12/min 320/350; *gas*: 0.50 bar (H_2).

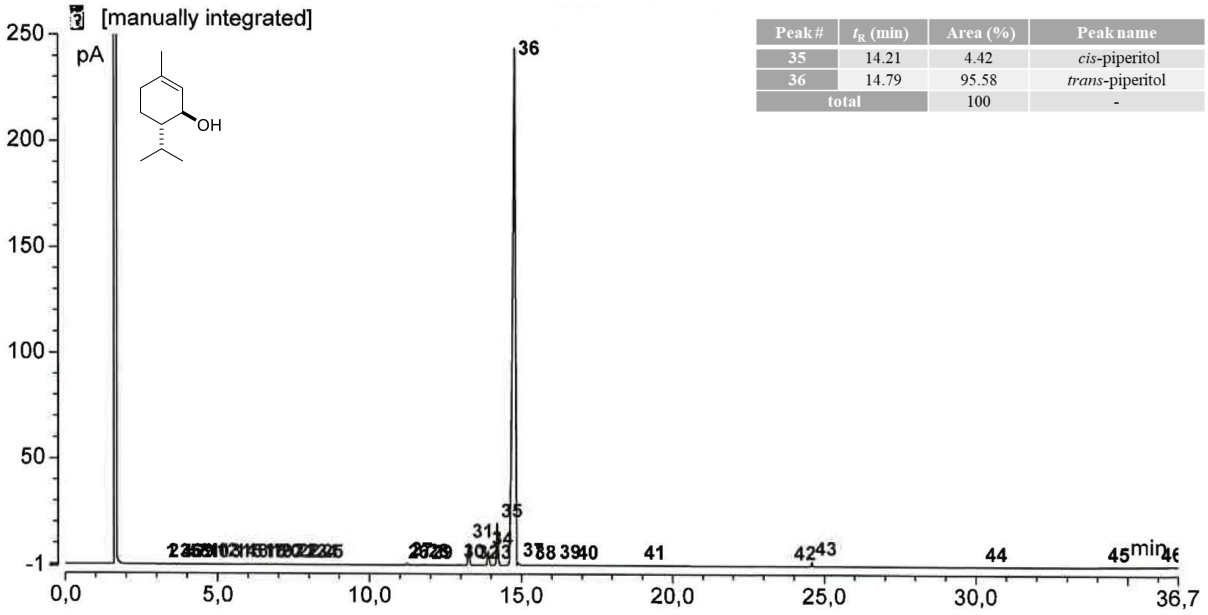

Peak #	t_R (min)	Area (%)	Peak name
35	14.21	4.42	*cis*-piperitol
36	14.79	95.58	*trans*-piperitol
total		100	-

Column: 25.0 m, Hydrodex-gamma-TBDAc-CD 0.25/?df; *temperature program*: 220/90 iso/350; *gas*: 0.50 bar (H_2).

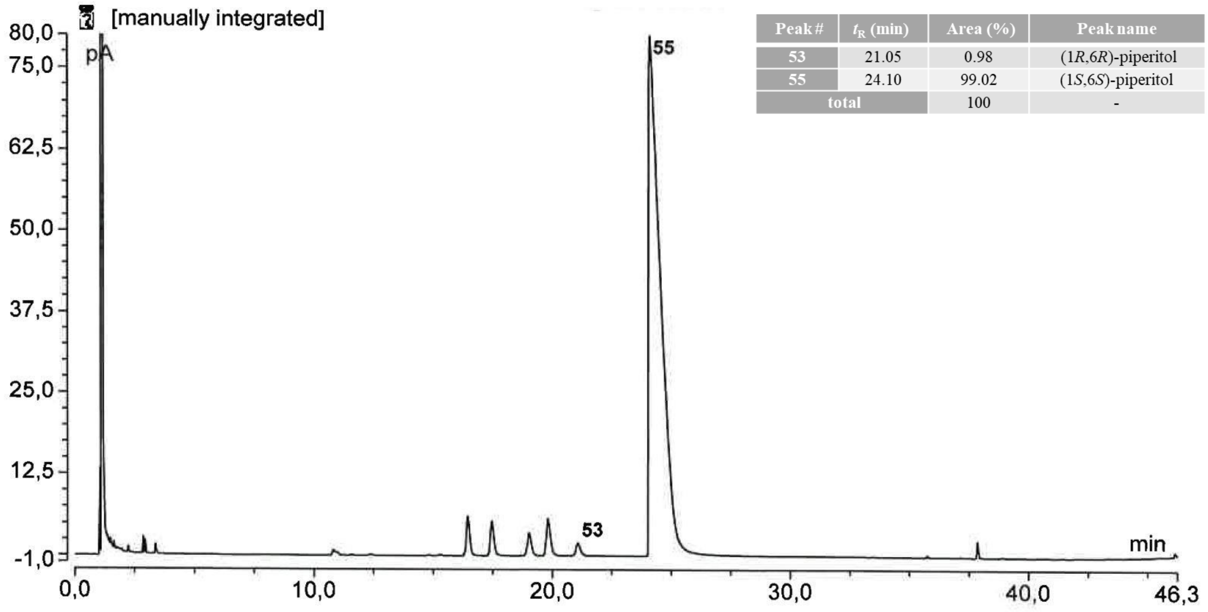

Peak #	t_R (min)	Area (%)	Peak name
53	21.05	0.98	(1*R*,6*R*)-piperitol
55	24.10	99.02	(1*S*,6*S*)-piperitol
total		100	-

3-ethyl-6-(prop-1-en-2-yl)cyclohex-2-en-1-ol (**16)**

Column: 25.0 m, Hydrodex-beta-TBDAc-CD 0,25/?df; *temperature program*: 220/70 iso/350; *gas*: 0.50 bar (H_2).

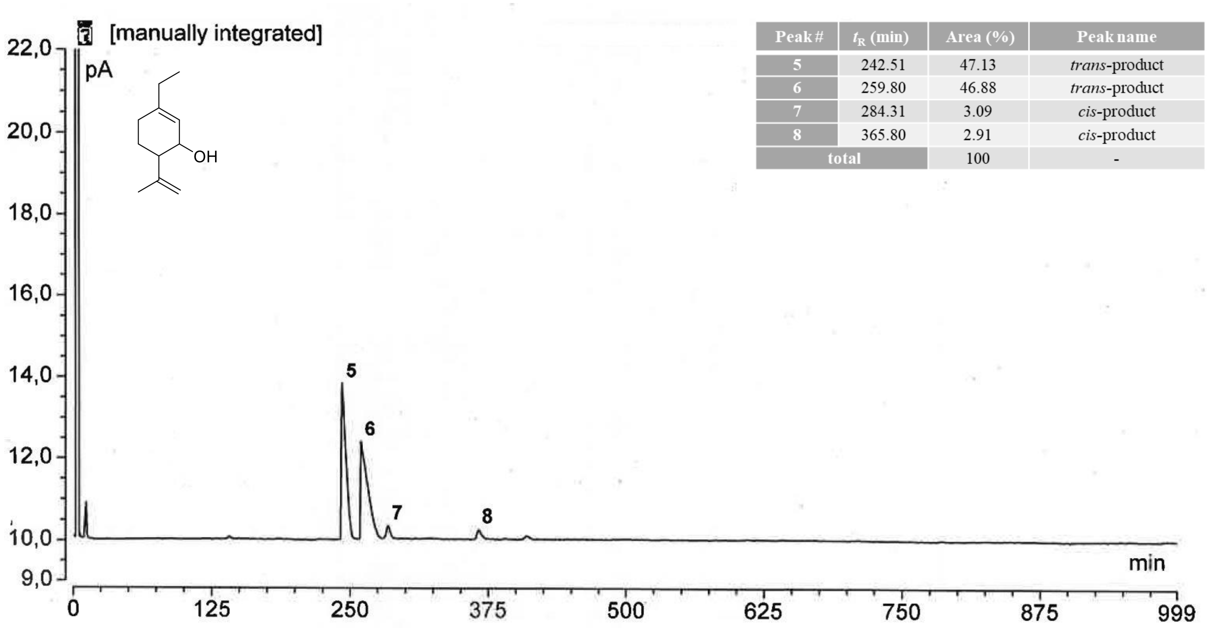

Peak #	t_R (min)	Area (%)	Peak name
5	242.51	47.13	*trans*-product
6	259.80	46.88	*trans*-product
7	284.31	3.09	*cis*-product
8	365.80	2.91	*cis*-product
total		100	-

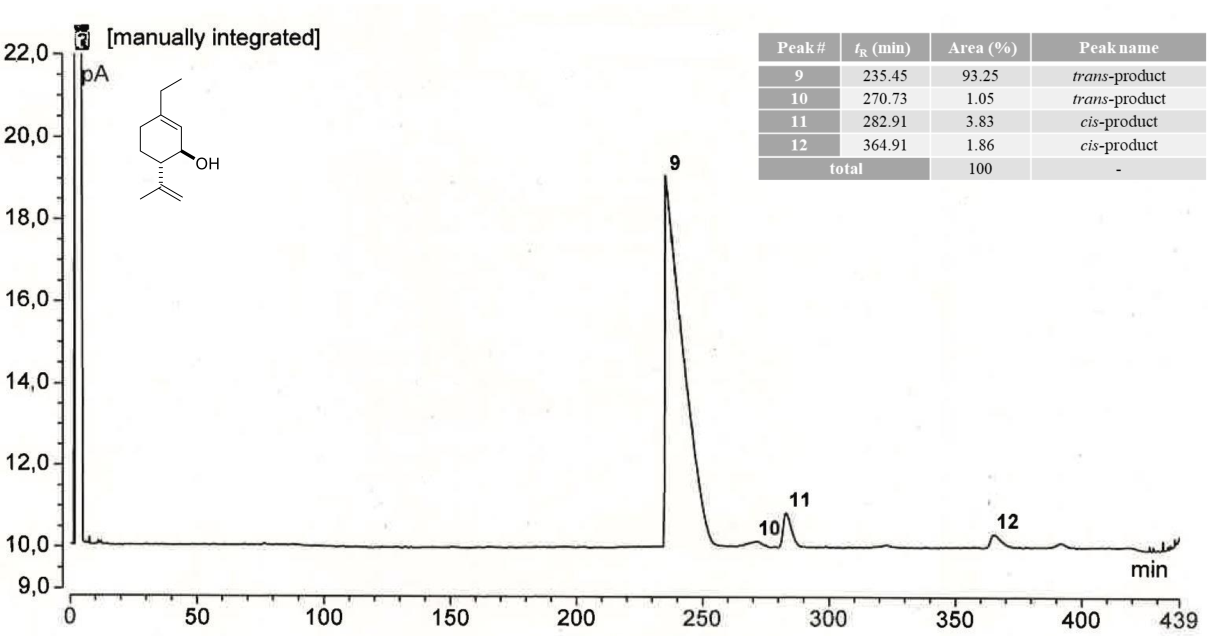

Peak #	t_R (min)	Area (%)	Peak name
9	235.45	93.25	*trans*-product
10	270.73	1.05	*trans*-product
11	282.91	3.83	*cis*-product
12	364.91	1.86	*cis*-product
total		100	-

6-(prop-1-en-2-yl)-3-propylcyclohex-2-en-1-ol (**17**)

Column: 30.0 m, BGB-174/BGB-1701 0.25/0.25df; *temperature program*: 220/100 76 min iso 8/min 240, 3 min iso/ 350; *gas*: 0.60 bar (H_2).

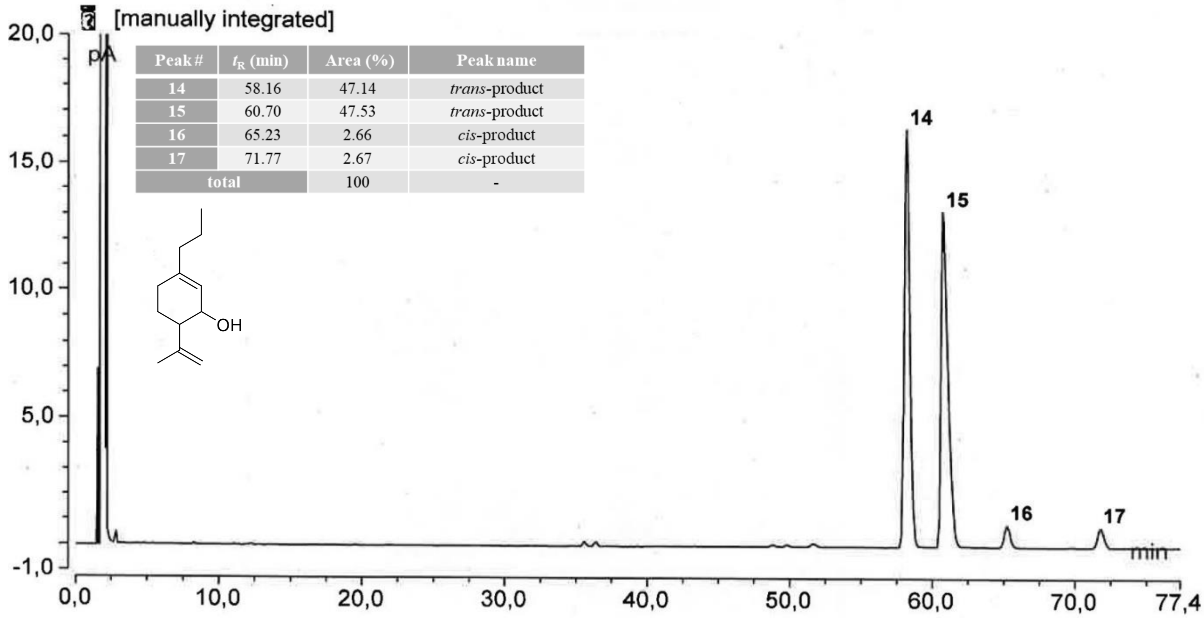

Peak#	t_R (min)	Area (%)	Peak name
14	58.16	47.14	*trans*-product
15	60.70	47.53	*trans*-product
16	65.23	2.66	*cis*-product
17	71.77	2.67	*cis*-product
total		100	-

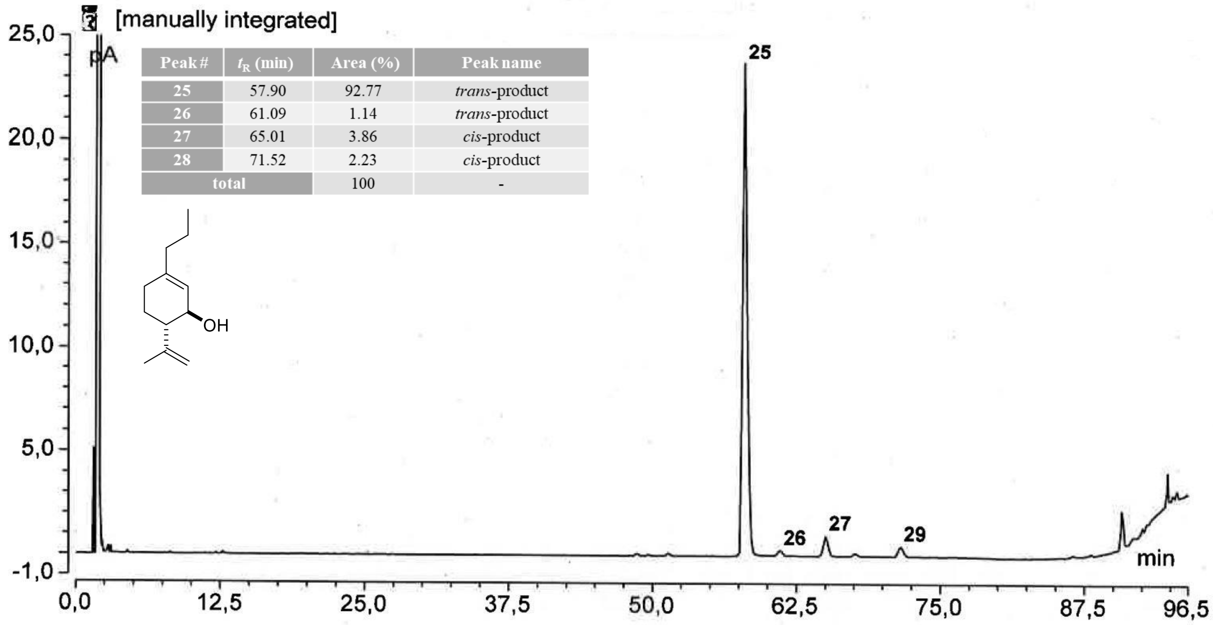

Peak#	t_R (min)	Area (%)	Peak name
25	57.90	92.77	*trans*-product
26	61.09	1.14	*trans*-product
27	65.01	3.86	*cis*-product
28	71.52	2.23	*cis*-product
total		100	-

3-isobutyl-6-(prop-1-en-2-yl)cyclohex-2-en-1-ol (**18**)

Column: 25.0 m, IVADEX-1/PS086 0.25/0.15df; *temperature program*: 220/100 65 min iso 8/min 220/350; g*as*: 0.50 bar (H_2).

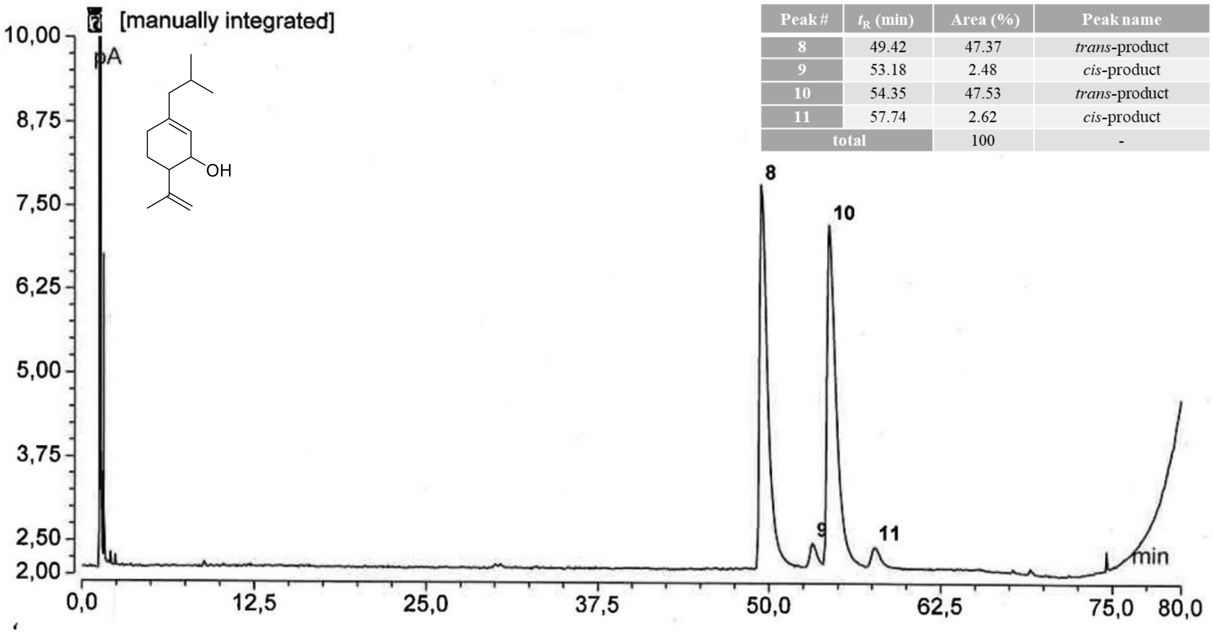

Peak #	t_R (min)	Area (%)	Peak name
8	49.42	47.37	*trans*-product
9	53.18	2.48	*cis*-product
10	54.35	47.53	*trans*-product
11	57.74	2.62	*cis*-product
total		100	-

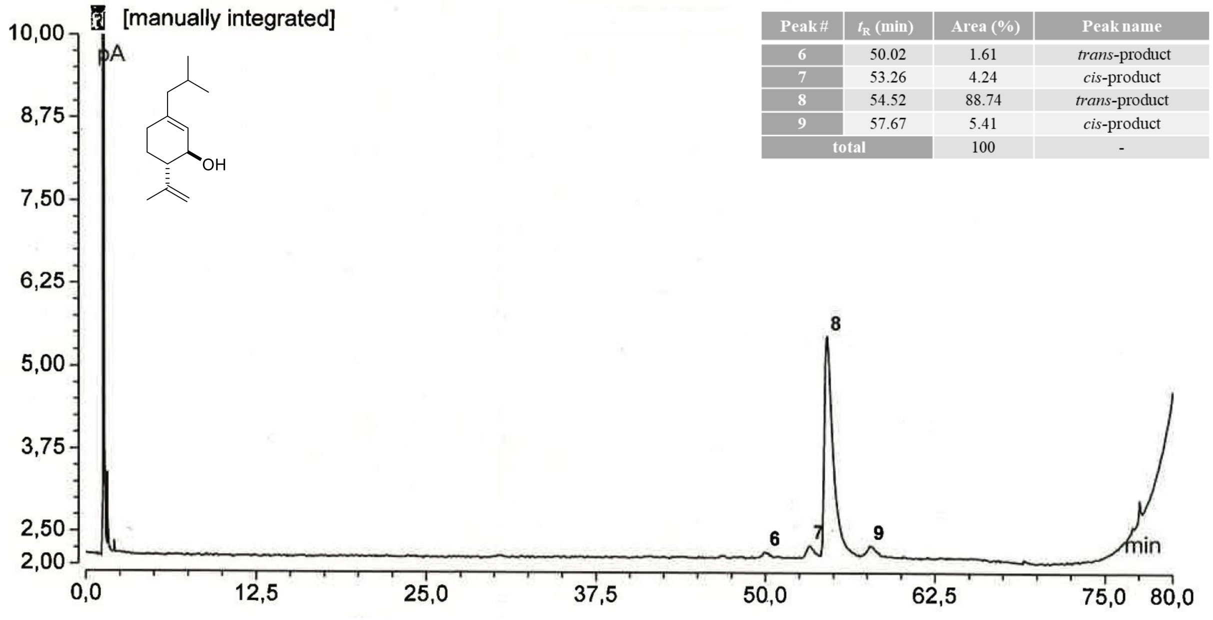

Peak #	t_R (min)	Area (%)	Peak name
6	50.02	1.61	*trans*-product
7	53.26	4.24	*cis*-product
8	54.52	88.74	*trans*-product
9	57.67	5.41	*cis*-product
total		100	-

3-benzyl-6-(prop-1-en-2-yl)cyclohex-2-en-1-ol (**19**)

Column: 30.0 m, BGB-174/BGB-1701 0.25/0.25df; *temperature program*: 220/ 130, 400 min iso 8/min 240/350; *gas*: 0.50 bar (H_2).

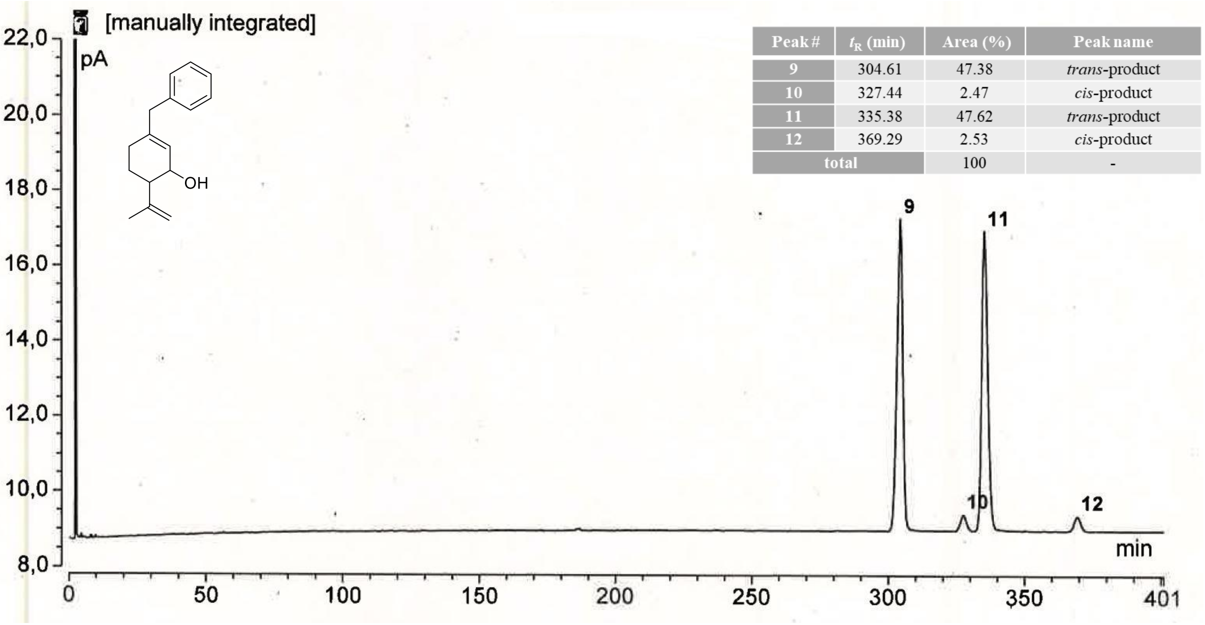

Peak #	t_R (min)	Area (%)	Peak name
9	304.61	47.38	*trans*-product
10	327.44	2.47	*cis*-product
11	335.38	47.62	*trans*-product
12	369.29	2.53	*cis*-product
total		100	-

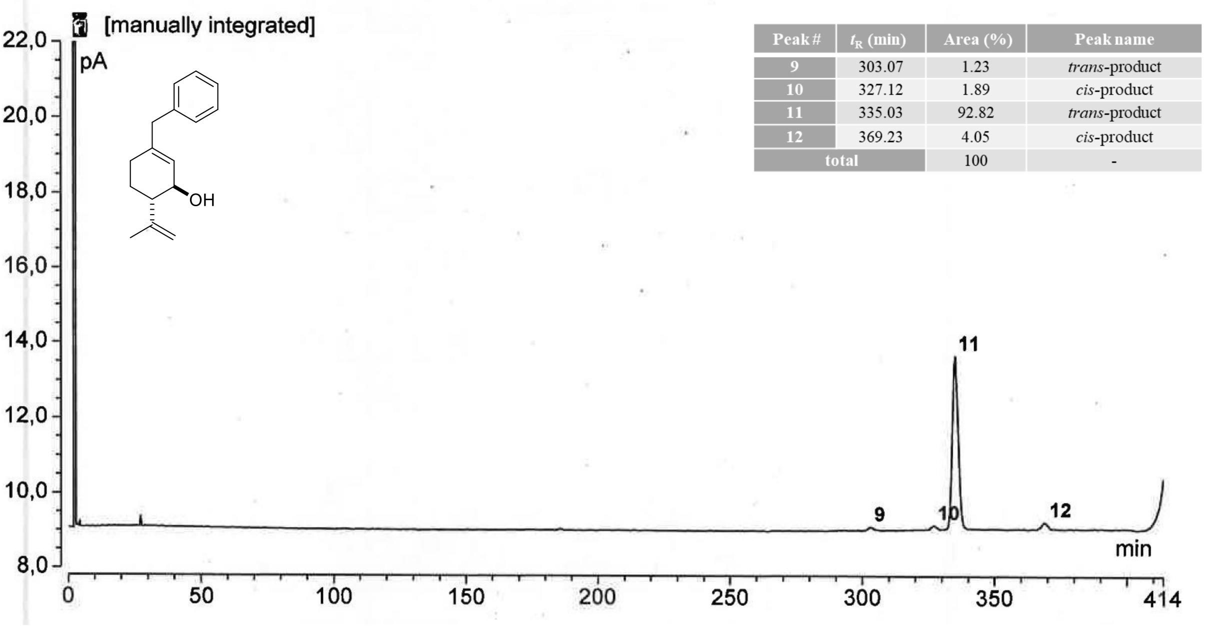

Peak #	t_R (min)	Area (%)	Peak name
9	303.07	1.23	*trans*-product
10	327.12	1.89	*cis*-product
11	335.03	92.82	*trans*-product
12	369.23	4.05	*cis*-product
total		100	-

3-phenethyl-6-(prop-1-en-2-yl)cyclohex-2-en-1-ol (**20**)

Column: 25.0 m, IVADEX-3 0.25/0.25df; *temperature program*: 220/120 iso/350; *gas*: 0.50 bar (H_2).

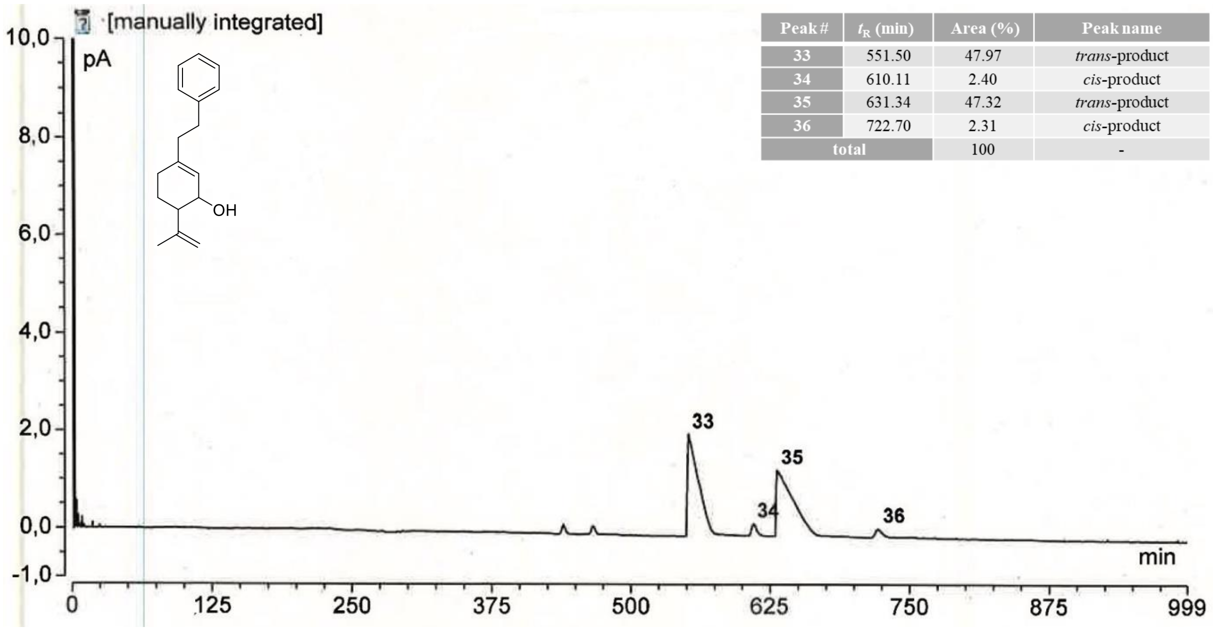

Peak #	t_R (min)	Area (%)	Peak name
33	551.50	47.97	*trans*-product
34	610.11	2.40	*cis*-product
35	631.34	47.32	*trans*-product
36	722.70	2.31	*cis*-product
total		100	-

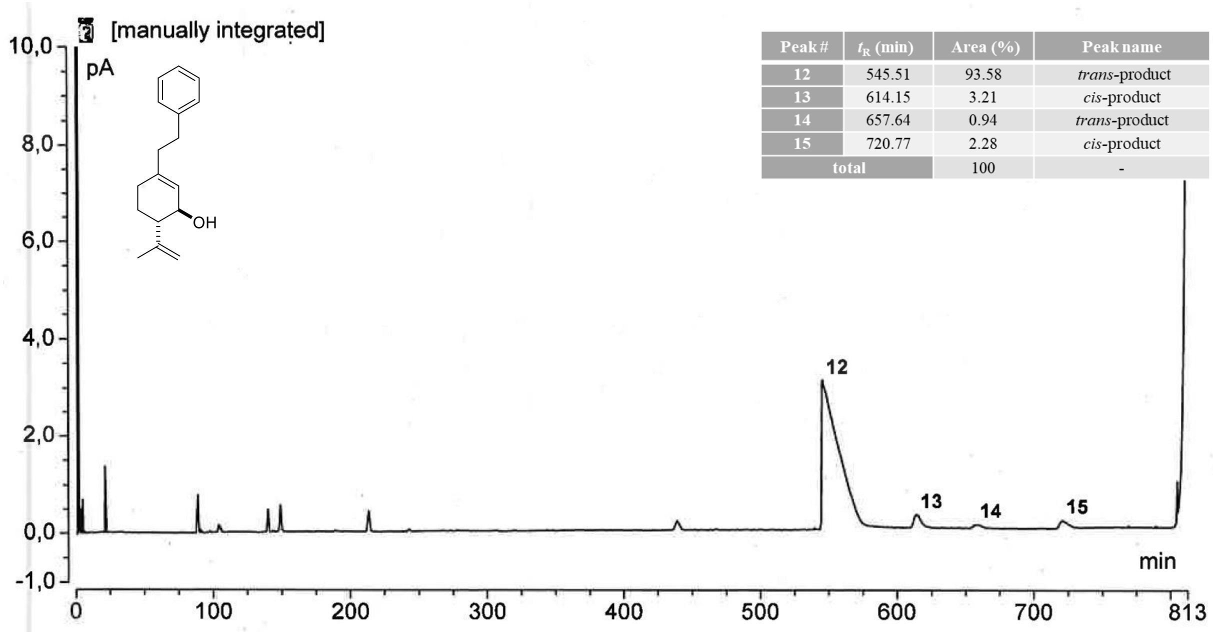

Peak #	t_R (min)	Area (%)	Peak name
12	545.51	93.58	*trans*-product
13	614.15	3.21	*cis*-product
14	657.64	0.94	*trans*-product
15	720.77	2.28	*cis*-product
total		100	-

6-(prop-1-en-2-yl)-3-(5-(triethylsilyl)pent-4-yn-1-yl)cyclohex-2-en-1-ol (**21**)

Column: 30.0 m, BGB-175/BGB-1701 0.25/0.25df; *temperature program*: 220/170 195 min iso 8/min 240, 3 min iso/ 350; *gas*: 0.50 bar (H_2).

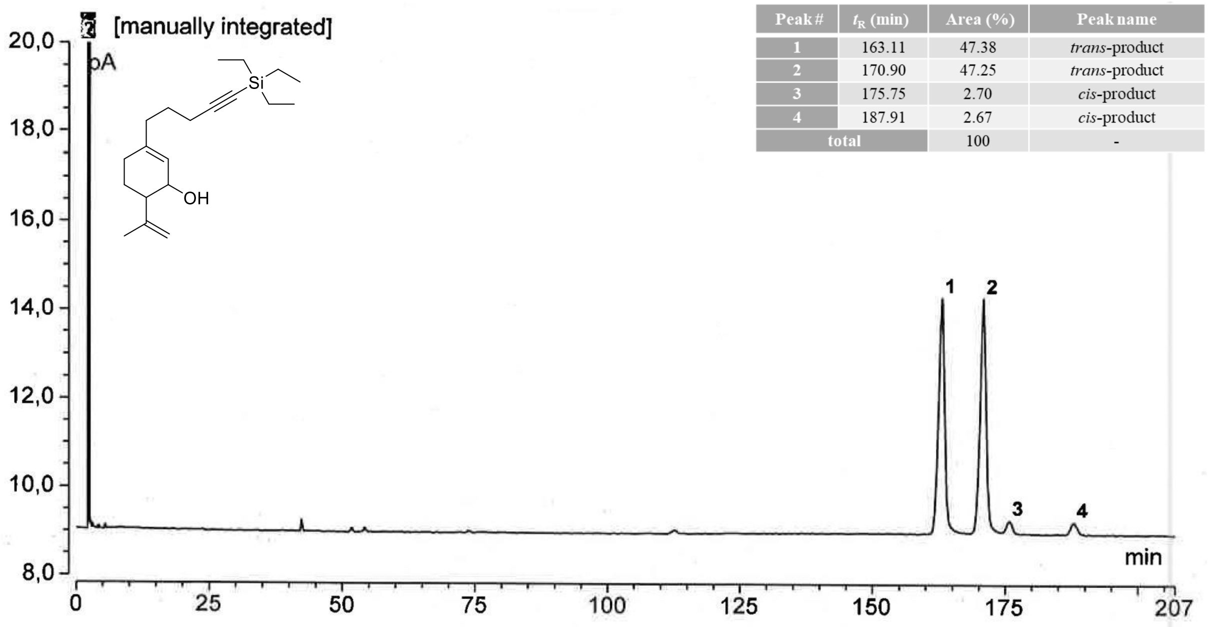

Peak #	t_R (min)	Area (%)	Peak name
1	163.11	47.38	*trans*-product
2	170.90	47.25	*trans*-product
3	175.75	2.70	*cis*-product
4	187.91	2.67	*cis*-product
total		100	-

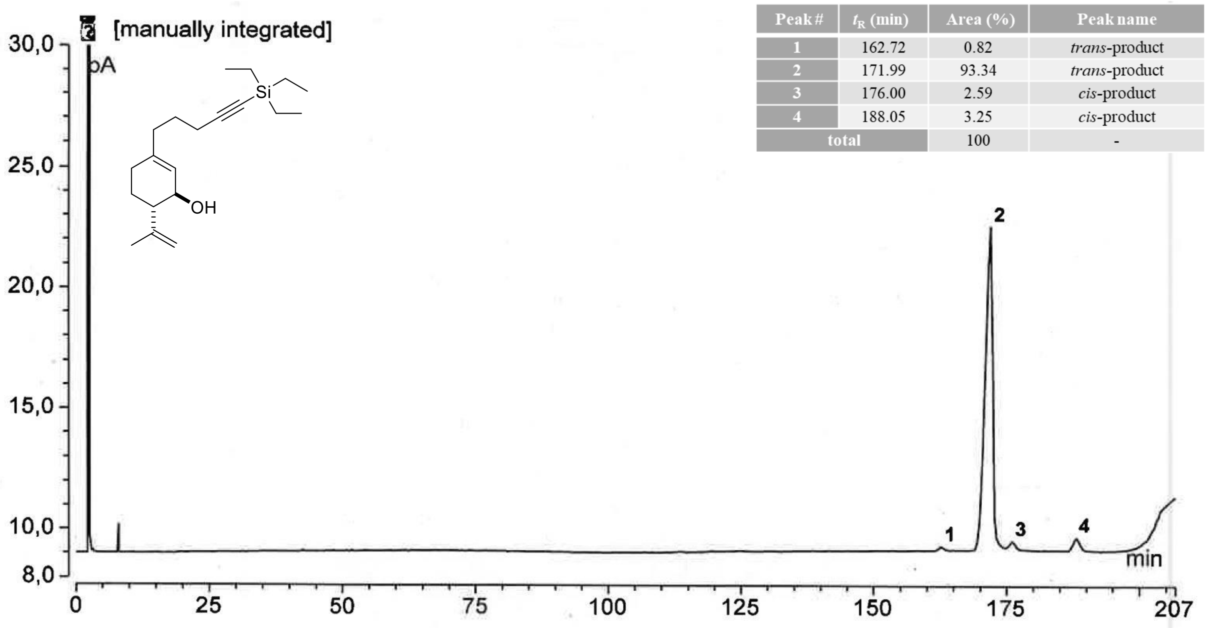

Peak #	t_R (min)	Area (%)	Peak name
1	162.72	0.82	*trans*-product
2	171.99	93.34	*trans*-product
3	176.00	2.59	*cis*-product
4	188.05	3.25	*cis*-product
total		100	-

3-cyclopentyl-6-(prop-1-en-2-yl)cyclohex-2-en-1-ol (**22**)

Column: 30.0 m, BGB-175/BGB-1701 0.25/0.25df; *temperature program*: 220/130 120 min iso 8/min 240/350; *gas*: 0.50 bar (H_2).

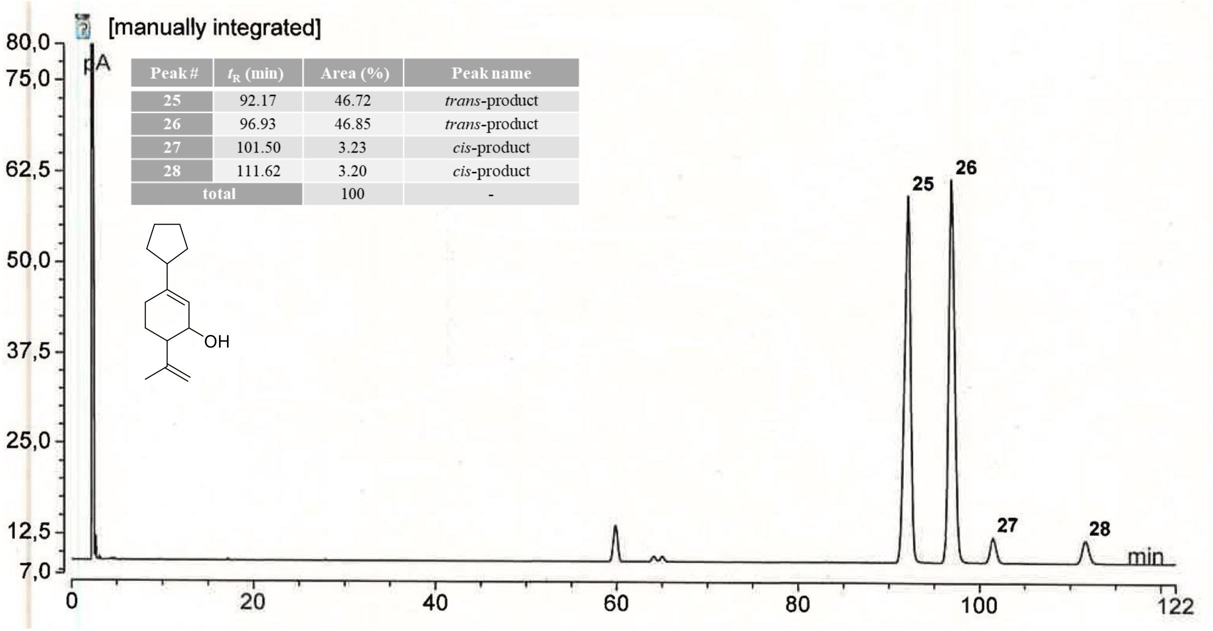

Peak#	t_R (min)	Area (%)	Peak name
25	92.17	46.72	*trans*-product
26	96.93	46.85	*trans*-product
27	101.50	3.23	*cis*-product
28	111.62	3.20	*cis*-product
total		100	-

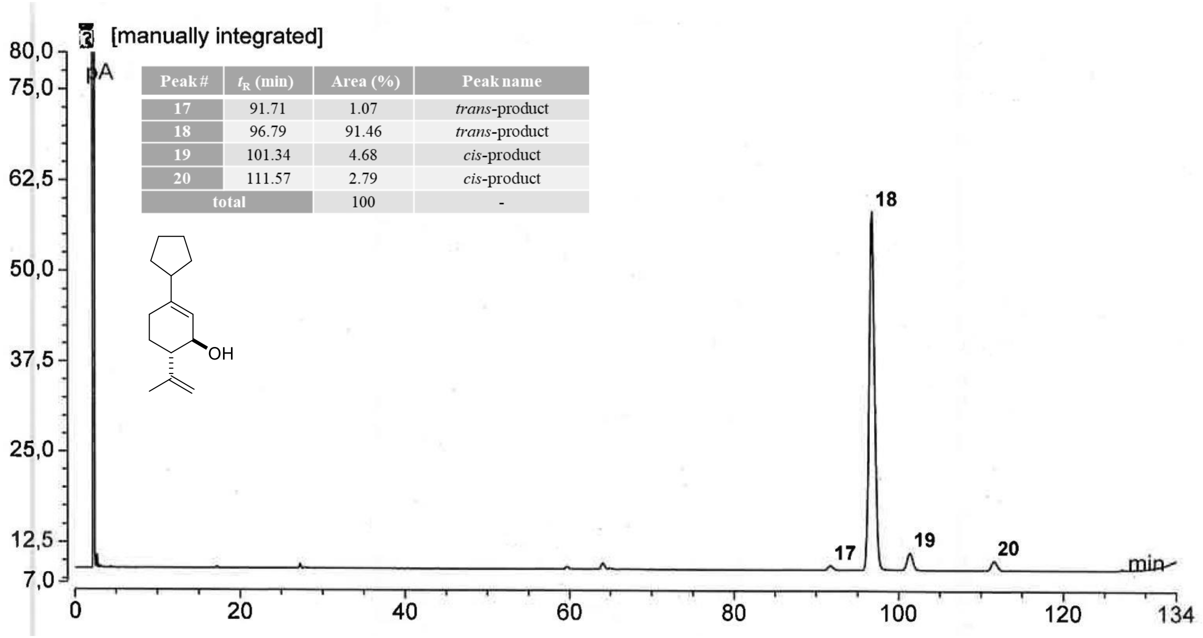

Peak#	t_R (min)	Area (%)	Peak name
17	91.71	1.07	*trans*-product
18	96.79	91.46	*trans*-product
19	101.34	4.68	*cis*-product
20	111.57	2.79	*cis*-product
total		100	-

4,4-dimethyl-6-(prop-1-en-2-yl)cyclohex-2-en-1-ol (**23**)

Column: 30.0 m, BGB-176/BGB-15 0.25/0.25df; *temperature program*: 220/60 iso/350; *gas*: 0.60 bar (H_2).

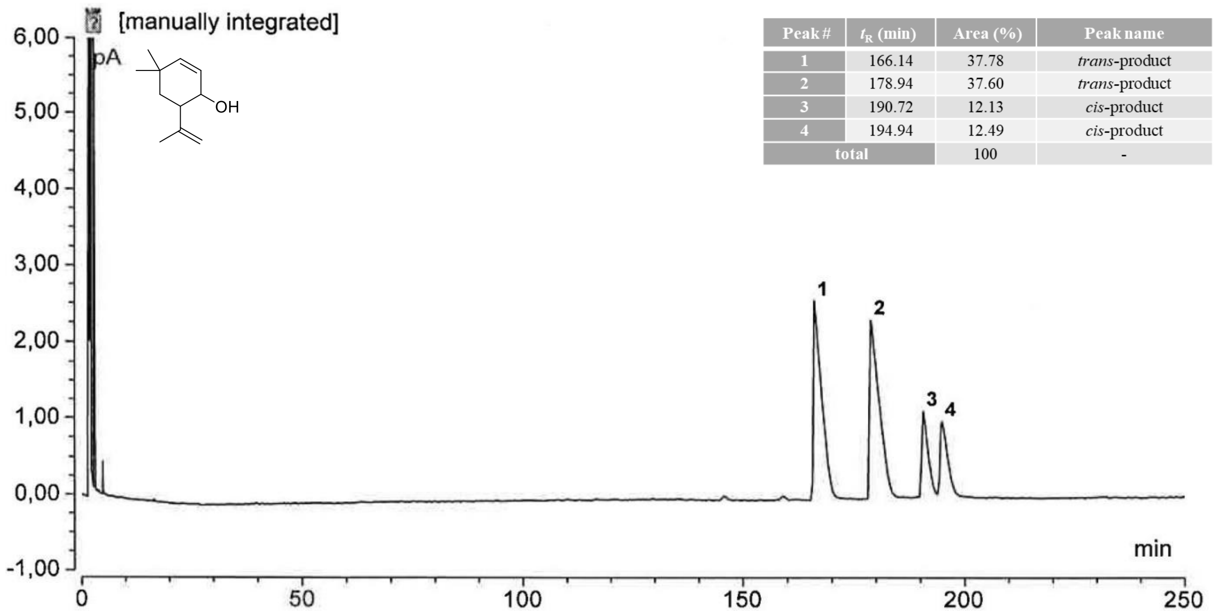

Peak #	t_R (min)	Area (%)	Peak name
1	166.14	37.78	*trans*-product
2	178.94	37.60	*trans*-product
3	190.72	12.13	*cis*-product
4	194.94	12.49	*cis*-product
total		100	-

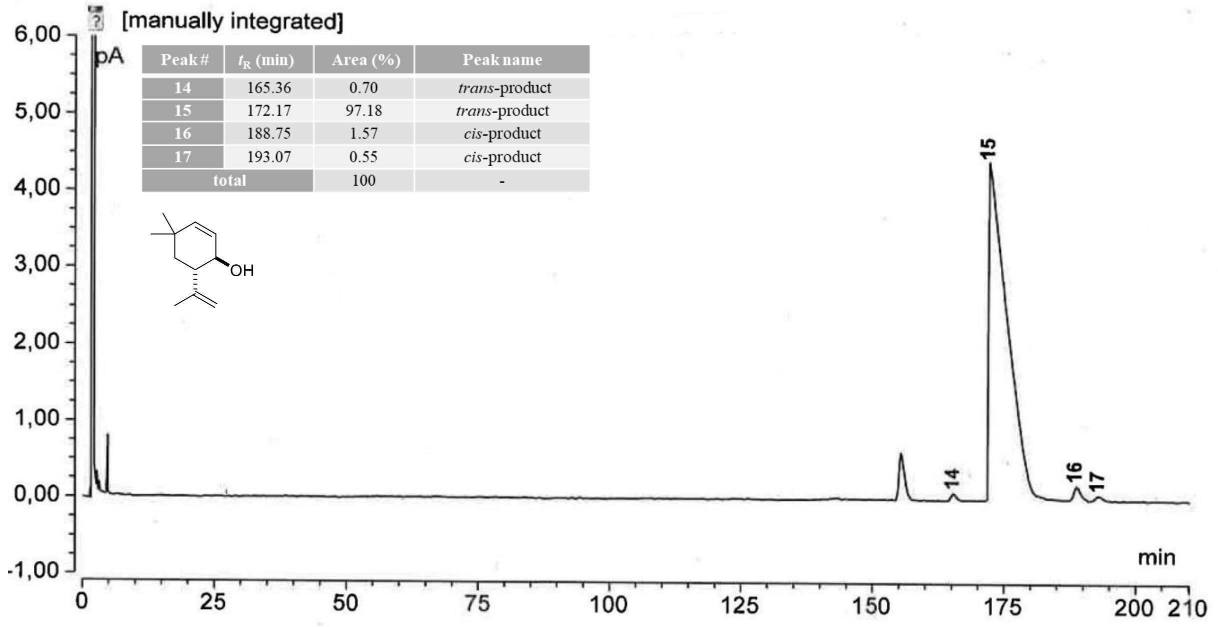

Peak #	t_R (min)	Area (%)	Peak name
14	165.36	0.70	*trans*-product
15	172.17	97.18	*trans*-product
16	188.75	1.57	*cis*-product
17	193.07	0.55	*cis*-product
total		100	-

4-methyl-[1,1'-bi(cyclohexane)]-1',3-dien-2-ol (**24**)

Column: 25.0 m, IVADEX-3 0.25/0.25df; *temperature program*: 220/110, 140 min iso 8/min 220, 3 min iso/ 350; *gas*: 0.50 bar (H_2).

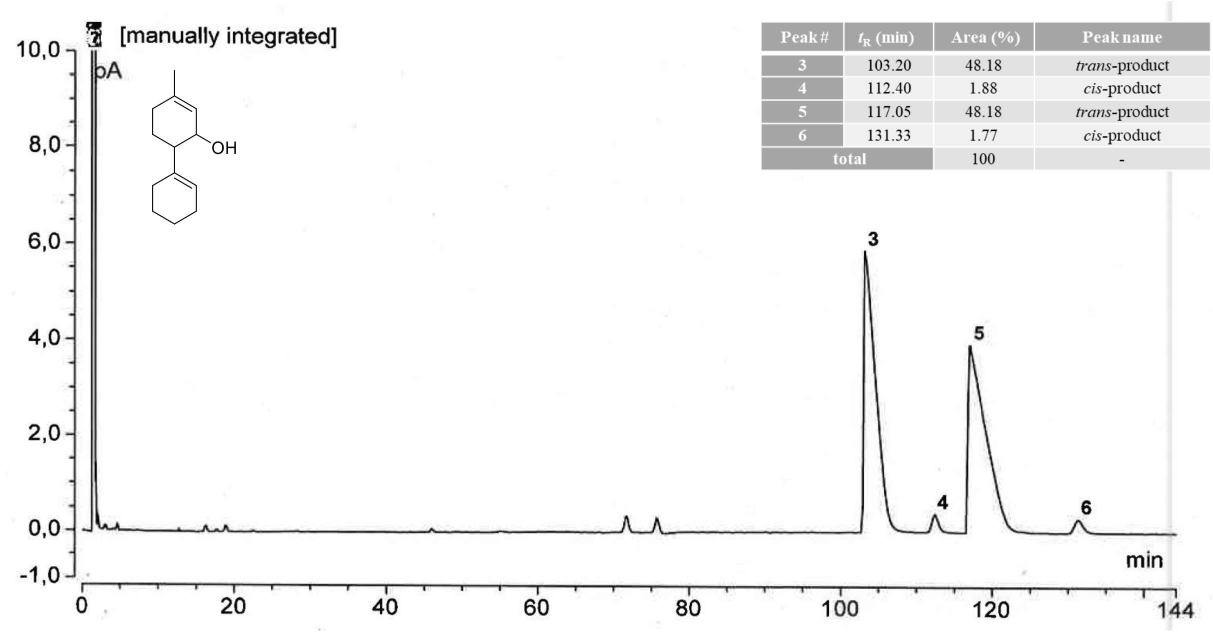

Peak#	t_R (min)	Area (%)	Peak name
3	103.20	48.18	*trans*-product
4	112.40	1.88	*cis*-product
5	117.05	48.18	*trans*-product
6	131.33	1.77	*cis*-product
total		100	-

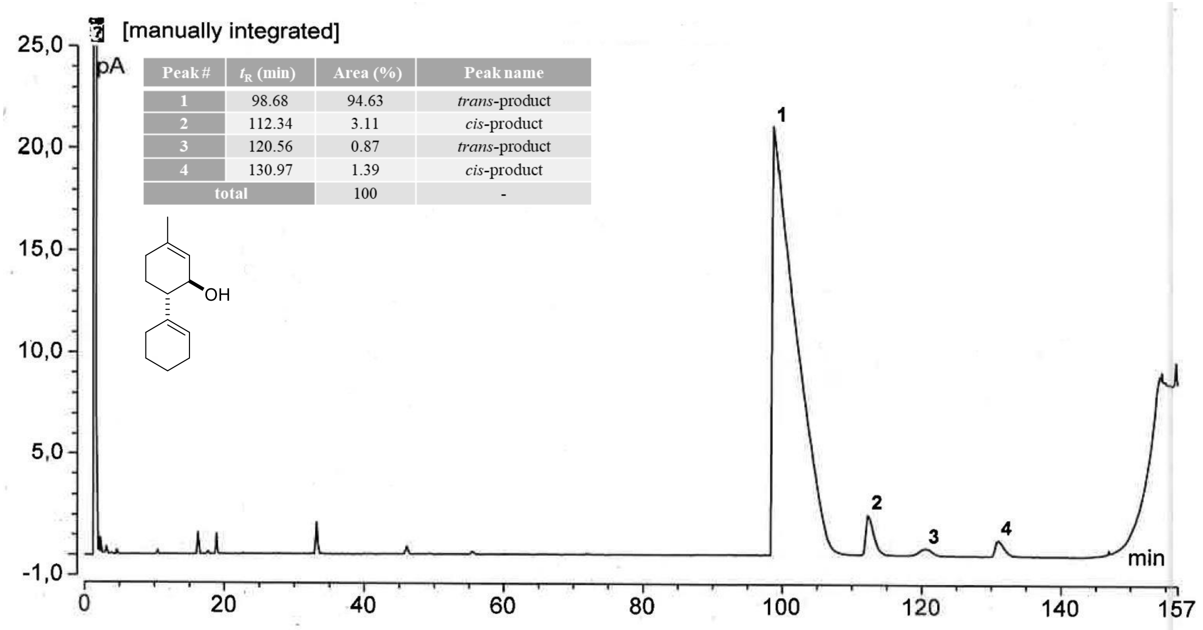

Peak#	t_R (min)	Area (%)	Peak name
1	98.68	94.63	*trans*-product
2	112.34	3.11	*cis*-product
3	120.56	0.87	*trans*-product
4	130.97	1.39	*cis*-product
total		100	-

2. Supplementary text

2.1. NMR spectroscopic kinetic studies

NMR Studies

General reaction procedure for NMR kinetic studies and dataprocessing with MNOVA (GP–8): The reaction progress was monitored by ^{1}H NMR spectroscopy at –15 °C. Generally, catalyst (5 mol%) was dissolved in PhMe-d_8 (0.5 mL) and precooled to –78°C in a dry ice/ethanol bath. After addition of a solution of neral (**1**) (5.0 μL, 1.0 equiv.) or isopiperitenol (**2**) (5.0 μL, 1.0 equiv.) in PhMe-d_8 (0.1 mL), the sample was quickly mixed by shaking and vortexing and was transferred to the pre-cooled NMR probe (–15 °C). After quick shimming, single-scan ^{1}H NMR spectra were acquired every 5 min for approximately 20 h to 24 h. The data was imported with the Reaction Monitoring Plugin into MNOVA 14.2.3 (Mestelab Research S.L., Spain) and was processed therein. NMR concentration plots were generated from ^{1}H NMR spectra at the different time points and were referenced to the first ^{1}H NMR spectrum of the series.

A – Reaction progress with different catalysts

*Note: The neral (**1**) (Z:E >95:5) and isopiperitenol (**2**) (d.r. (trans:cis) = 12:1, e.r. (trans) = 98.5:1.5) concentration for the reaction progress studies: 49.3 mM.*

The cyclization reaction of neral and the conversion/decomposition of isopiperitenol was monitored in the presence of different Brønsted acid catalysts **3**–**6** by ^{1}H NMR spectroscopy. The reaction followed general procedure *GP–8*. The Figures 2 (main text) and S2 show the formation and decomposition of isopiperitenol, respectively. Noteworthy, the differences in the decomposition rates for the two different diastereoisomers (*cis*- and *trans*-isopiperitenol) are neglectable (see Figure S2). The decomposition rates for isopiperitenol depend on the catalyst loading (see table T5).

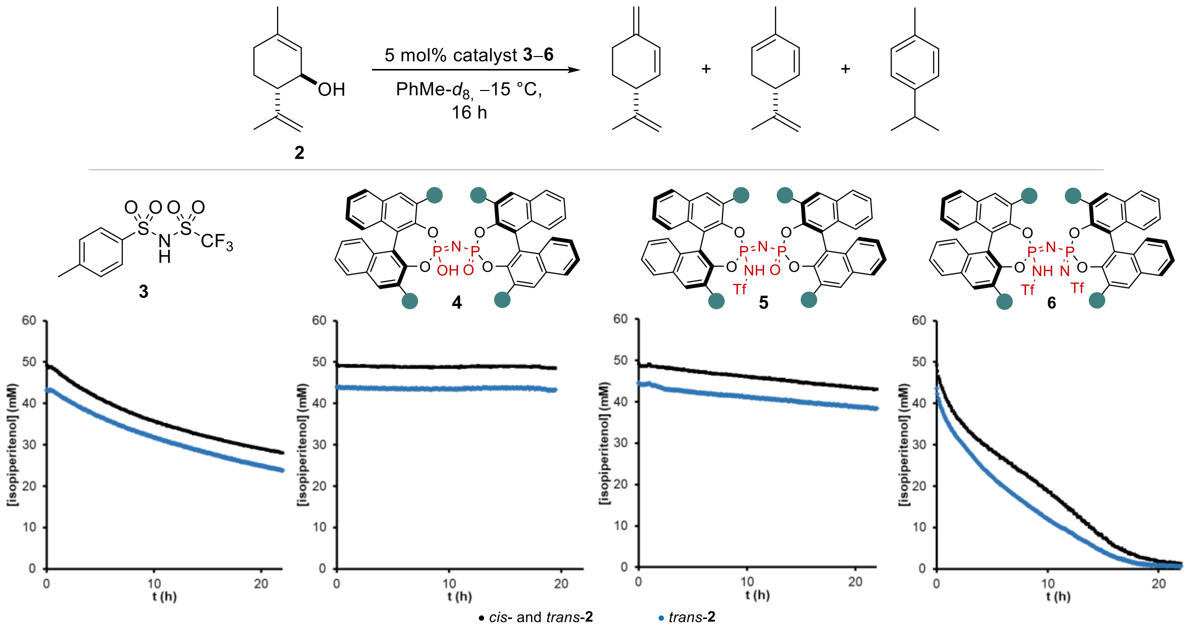

Figure S2: Stability of isopiperitenol (2) under optimized reaction conditions using Brønsted acids 3–6 analyzed by ^{1}H NMR spectroscopy. The *trans* and *cis*-diastereoisomers decompose in similar reaction rates.

B – Reaction profile analysis of the optimal catalyst

*Note: The neral (**1**) concentration for reaction profile analysis: 58 mM*[4]

The reaction from neral (**1**) to isopiperitenol (**2**) followed the general procedure (*GP–8*) using *i*IDP catalyst **5** proceeds smoothly (Figure S3, A) and the product concentration reaches a plateau after approximately 12 h before the concentration slowly decreases due to formation of sideproducts.

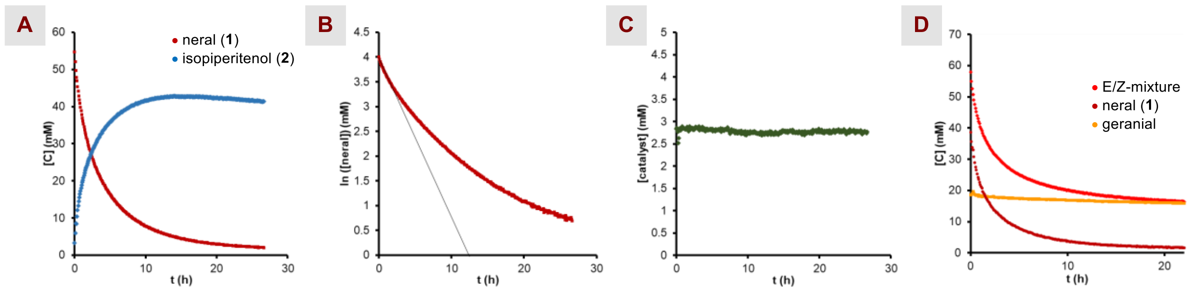

Figure S3: ^{1}H NMR reaction profile analysis. A) Reaction profile of the cyclization reaction using optimal *i*IDP catalyst **5** under optimized reaction conditions. **B**) Plot of ln ([neral]) against t. The decrease of reaction rates of the cyclization reaction indicated reaction inhibition. **C**) Profile of the catalyst concentration at reaction temperature of –15 °C. **D**) Reaction of the diastereoisomeric mixture under optimal reaction conditions. The *E*-isomer, geranial, appeared unreactive under the applied reaction conditions.

[4] The starting material was added (neat) to the precooled solution.

The neral conversion rate drops non-linearly during the reaction and does not follow a first-order kinetic behavior, which would be expected for an intramolecular cyclization reaction.[28] The plot of $\ln(c_{neral})$ against the time *t* (Figure S3, B) shows a non-linear curve, which suggests either catalyst decomposition or product inhibition in the cyclization of neral.

^{31}P NMR analysis of the catalyst during the reaction revealed a constant concentration during the whole reaction time and no additional signals were observed (Figure S6, C). Furthermore, ^{1}H NMR studies on *E*/*Z*-mixture of the starting material employed in the reaction revealed that the corresponding *E*-isomer (Geranial) is not reactive throughout the whole reaction time (Figure S6, D) and a double-bond isomerization is not observed under optimal reaction conditions (–15/–20 °C).

C – Determination of the reaction order in catalyst

As no significant change of the ^{31}P NMR signals or drop of catalyst concentration was detected during the reaction (Figure S3, C), the reaction order of the catalyst was determined with *Variable Time Normalisation Analysis* (VTNA) established by the Burés group[29,30].

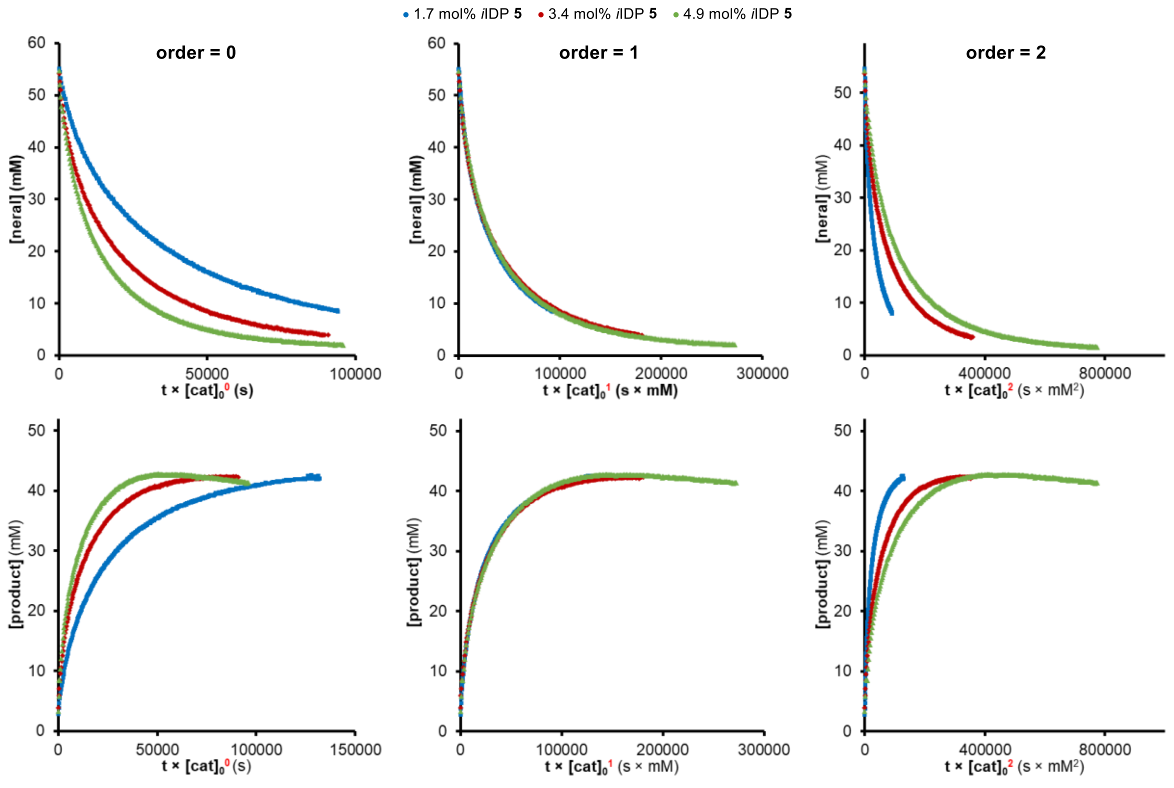

Figure S4: Determination of the reaction order in catalyst by the Burés method for the cyclization of neral (1). Concentration plots of neral conversion (top) and product formation (bottom) with time scales normalized to different reaction orders of the catalyst (0th left, 1st middle, 2nd right).

Therefore, the reaction was carried out in the presence of different catalyst loadings of ideal *i*IDP catalyst **5** (1.7 mol%, 3.4 mol% and 4.9 mol%). The catalyst concentration was extracted as average from the ^{1}H NMR data of the first 2 h of reaction time. The figure S4 shows an overview of different reaction profiles with time scales normalized to different catalyst orders. The best overlap was found when the reaction profiles are normalized to a first-order dependence in catalyst concentration.

The same method was used to determine the reaction order in catalyst of the decomposition of isopiperitenol (**2**). The catalyst concentration was extracted as average from the ^{1}H NMR data of the first 2 h of reaction time. The following figure S5 shows an overview of different reaction profiles with time scales normalized to different catalyst orders.

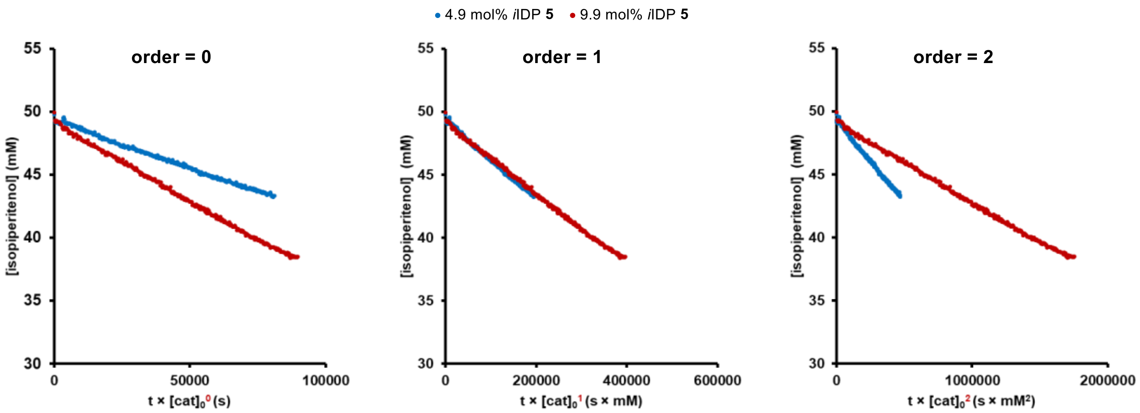

Figure S5: Determination of the reaction order in catalyst by the Burés method for the decomposition of isopiperitenol (2). Concentration plots of neral conversion with time scales normalized to different reaction orders of the catalyst (0th left, 1st middle, 2nd right).

The best overlap was found when the reaction profiles are normalized to a first-order dependence in catalyst concentration.

D – Product Inhibition Studies

To investigate the influence of the product concentration on the neral conversion rate, two different additional experiments were performed and were compared to the standard conditions.

In the first experiment, additional isopiperitenol (**2**) (0.55 equiv.) was added to the reaction. In the second, independent experiment, the reaction was monitored at a lower initial neral concentration (38.4 mM) and the time scale was shifted to match the same neral concentration of the standard condition following a procedure by *Blackmond et. al.*.[31,32] In

absence of product inhibition, an overlap of all the concentration profiles is expected. However, in the current case (Figure S6) the conversion of neral is slower, when a higher excess of isopiperitenol is present in the sample. Both independent experiments strongly support that the product inhibits the cyclization reaction.

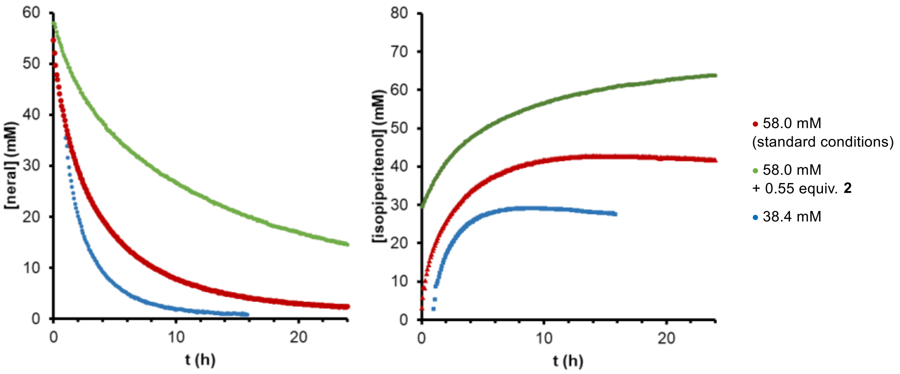

Figure S6: Neral (left) and isopiperitenol (right) concentration profiles at −15 °C with different initial conditions and *c*[*i*IDP] = 2.84 mM. Reaction under standard conditions (red); Reaction with additional 0.55 equiv. of product (**2**) (green); Reaction at a lower concentration of 38.4 mM, where the time t_0 was shifted (1.1 h) to match the same time point under standard conditions (blue).

E – Stoichiometry experiments

To investigate the interaction of the catalyst with the substrate and the product, respectively, stoichiometric NMR experiments were performed at low temperatures. Therefore, *i*IDP catalyst **5** (21.2 mg, 0.012 mmol, 1.0 equiv.) was dissolved in PhMe-d_8 (0.5 mL). Neral (2 µL, 0.012 mmol, 1.0 equiv.) was added at –80 °C and after quick shaking and vortexing the sample and was transferred to the NMR probe precooled to –60 °C. At this temperature a ^{1}H NMR and an *edited*-^{1}H,-^{13}C HSQC were acquired to investigate the changes upon mixing. When mixing the catalyst and the substrate significant shifts are observed for the NMR signals of neral. The aldehyde signal H1 shifts 0.98 ppm towards lower frequencies in the ^{1}H NMR spectrum. The olefinic signals H2 and H6 shift 0.24 ppm and 0.20 ppm also shift to lower frequencies. The origin of these shifts could be due to electronic changes, due to activation from the catalyst or due to anisotropic shielding effect from the aromatic groups of the catalyst. The ^{13}C NMR signal of aldehyde C1 shifts +5.2 ppm, all other protonated carbons are shifting less than ±2 ppm upon coordination (see figure S8). This is in line with the proposed activation of the aldehyde by the catalyst via protonation, which creates a δ^+ at the carbonyl carbon.

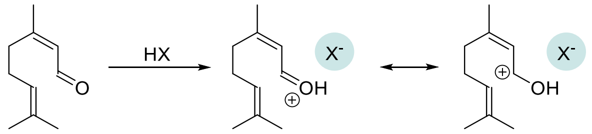

HX = *i*IDP **5**

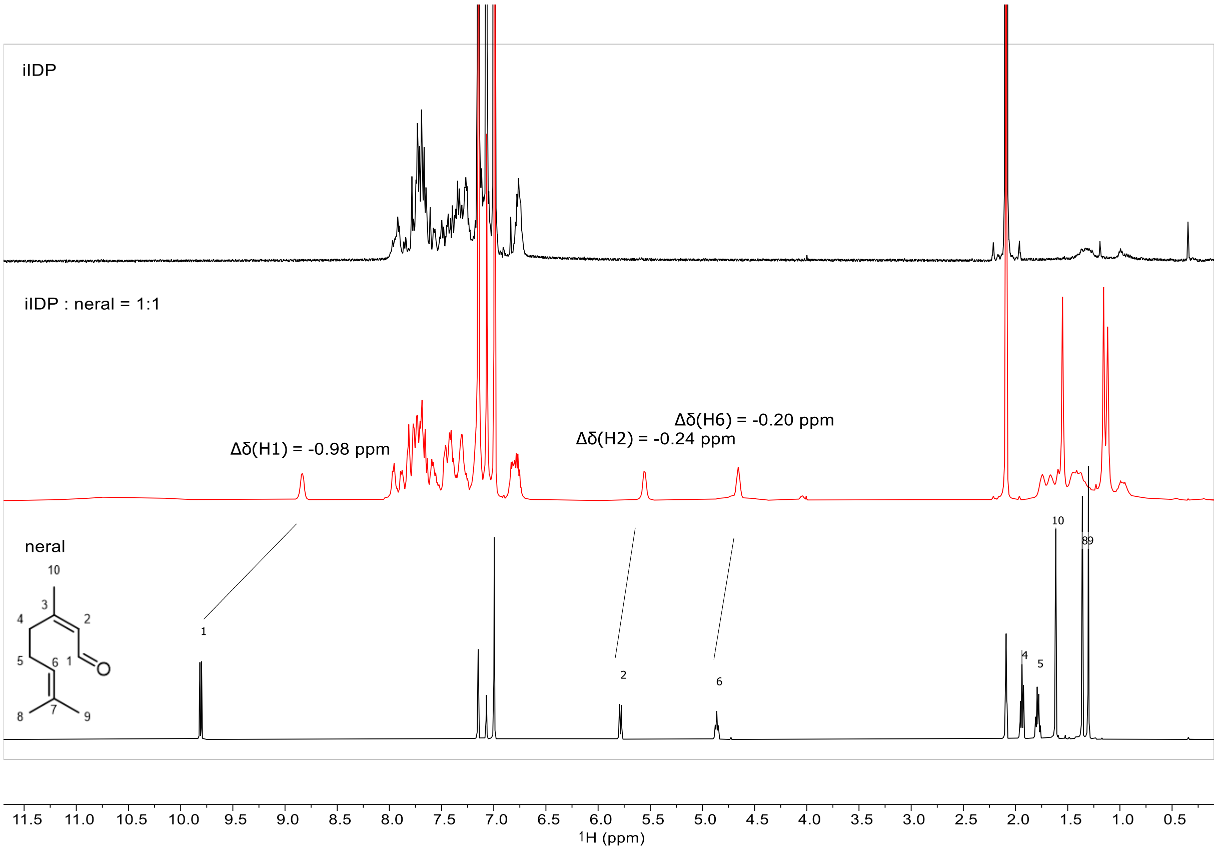

Figure S7: Comparison of different ^{1}H NMR spectra at –60 °C in PhMe-*d*$_8$. Top: *i*IDP catalyst **5**, middle: 1:1 mixture of neral and *i*IDP catalyst **5**; bottom: neral.

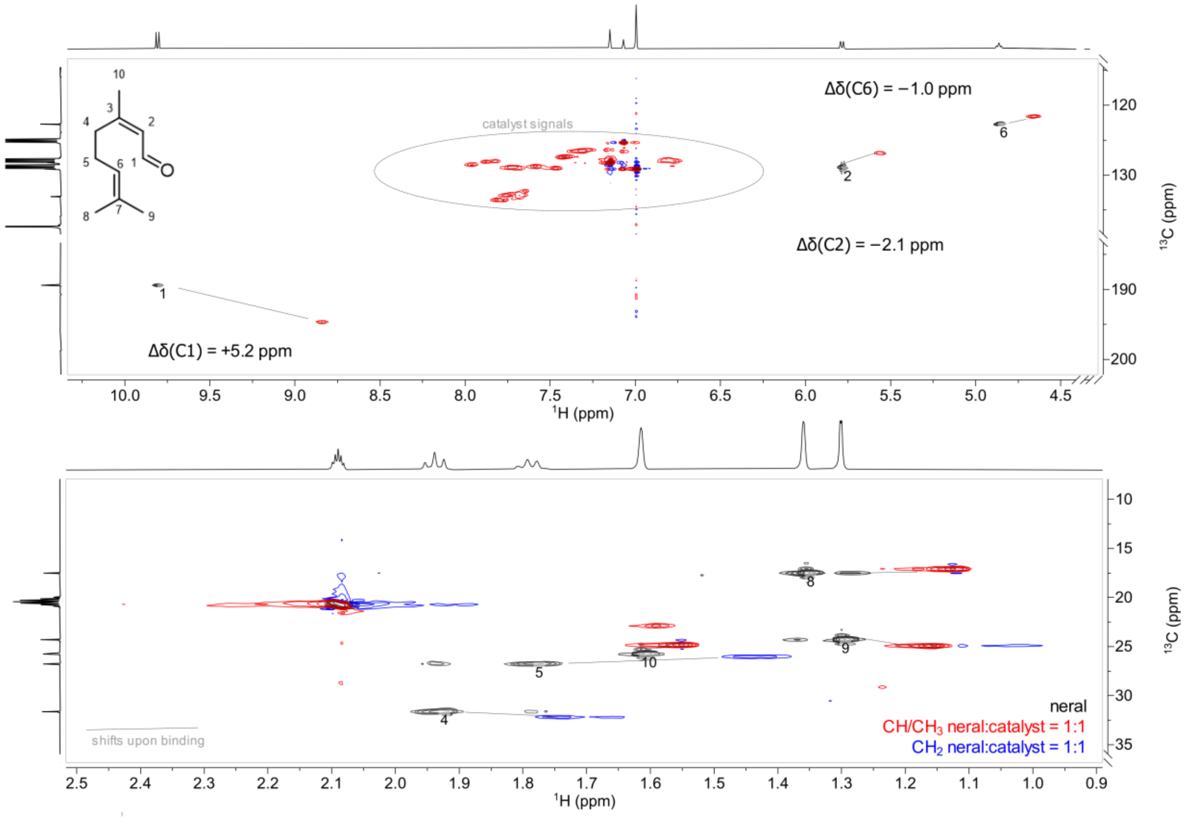

Figure S8: Comparison of an ^{1}H,^{13}C HSQC spectrum of neral (1) (black) with an edited ^{1}H-^{13}C HSQC of a 1:1 mixture of *i*IDP 5 + neral (blue, red) at –60 °C in PhMe-*d*$_8$. Two different regions are depicted the aromatic/olefinic region (top) and the aliphatic region (bottom).

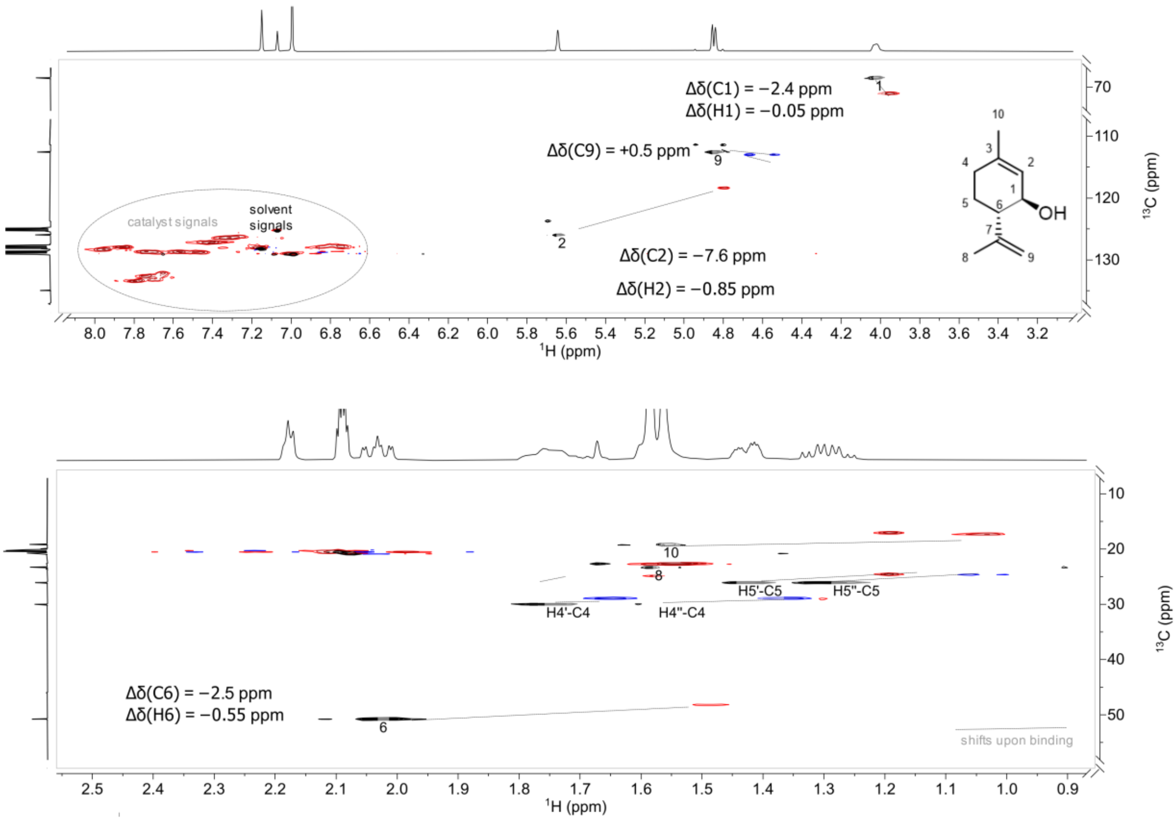

Figure S9: Comparison of an ^{1}H,^{13}C HSQC spectrum of the isopiperitenol (2) (black) with an edited ^{1}H,^{13}C HSQC of a ~1:1 mixture of *i*IDP 5 + isopiperitenol (2) (blue, red) at –60 °C (blue, red) in PhMe-d_8. Two different regions are depicted the aromatic/olefinic region (top) and the aliphatic region (bottom).

After the measurement, the sample was warmed up to –50°C for approximately 9 h until most of neral was converted (>90%) to the desired product **2**. After cooling to –60 °C an *edited*-^{1}H,-^{13}C HSQC of the sample was acquired (see figure S9). The most significant shifts are observed for position 2 ($\Delta\delta^{13}$C = –7.6 ppm, $\Delta\delta^1$H = 0.85 ppm) and position 6 ($\Delta\delta^{13}$C = –2.5 ppm, $\Delta\delta^1$H = 0.55 ppm) in the desired product. These observed significant shifts can be explained by a strong aromatic ring current effect from the perfluorated aryl systems (deshielding C-F···H-interactions).

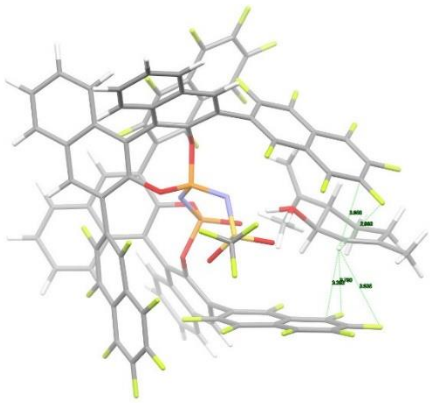

Figure S10: Structure of the protonated product in the cavity of the *i*IDP 5 obtained from computational calculations. Distances of the H2-atom of the protonated product to the perfluoranted naphthyl wings of the catalyst **5** are measured and are displayed in green.

F – Deuterium NMR spectroscopic experiments

The labeling experiment was carried out following the general procedure *GP–1* using deuterium-labelled neral **25**. A mixture of deuterated products **26** and **27** was obtained and characterized by ^{1}H, ^{2}H (see figure S11) and ^{13}C NMR spectroscopy.

The distribution of products **26** and **27** in the sample excludes that the performed cyclization of neral using *i*IDP catalyst **5** proceeds via a concerted carbonyl-ene pathway involving a six-membered transition state to give the homoallylic alcohol. From the obtained results of the deuterium-labeling studies, a stepwise mechanism *via* an isopropenyl cation and a highly asynchronous mechanism are feasible.

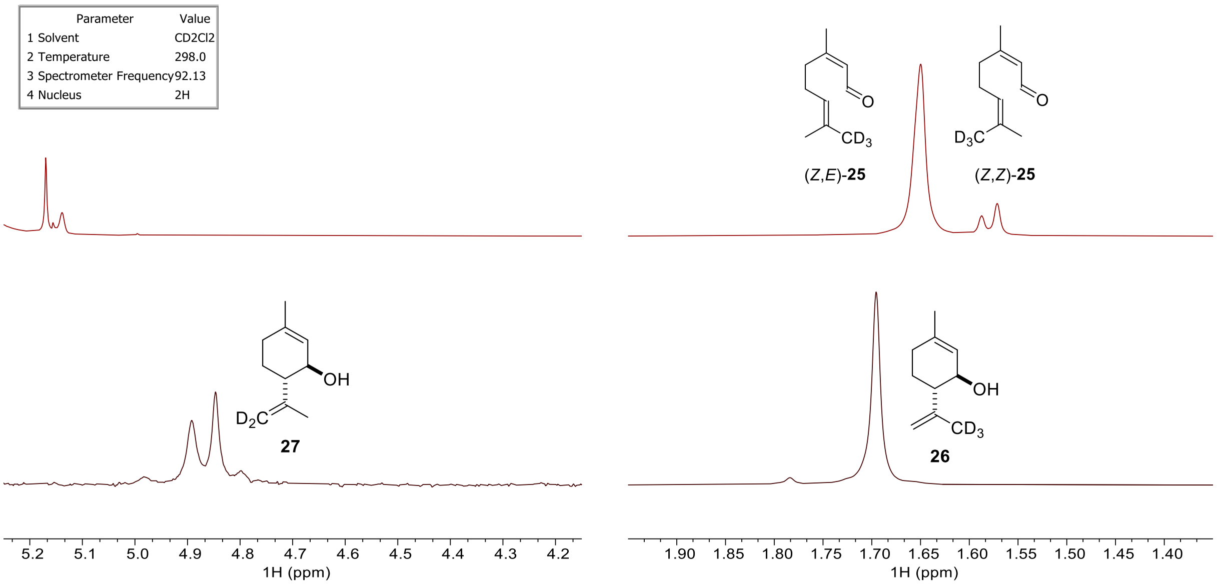

Figure S11: ^{2}H NMR of the deuterated neral 25 (top) and the mixture obtained from the catalytic reaction (bottom) with *i*IDP catalyst 5. The starting material shows a 85:15 distribution of the corresponding (*Z*,*Z*)-**25** and (*Z*,*E*)-**25** isomers.

2.2. Rotameric mixtures of IDP, *i*IDP and IDPi-catalysts

In this work, three novel confined, chiral Brønsted acid catalyst **4**–**6** were introduced. The presented catalysts share the same BINOL (3,3’-substituent: perfluoronaphthyl) and only differ in the inner core constitution (X = Y = O IDP **4**, X = O Y = NTf *i*IDP **5**, X = Y = NTf IDPi **6**). Noteworthy for the upcoming discussion of the NMR analysis, in general IDP and IDPi are C_2-symmetric compounds while *i*IDP **5** is non C_2-symmetric due to its bifunctional innercore. When a free rotation of the C–C-bond between BINOL and 3,3’-substituent is allowed, the NMR spectra for each nuclei show a single set of signals.

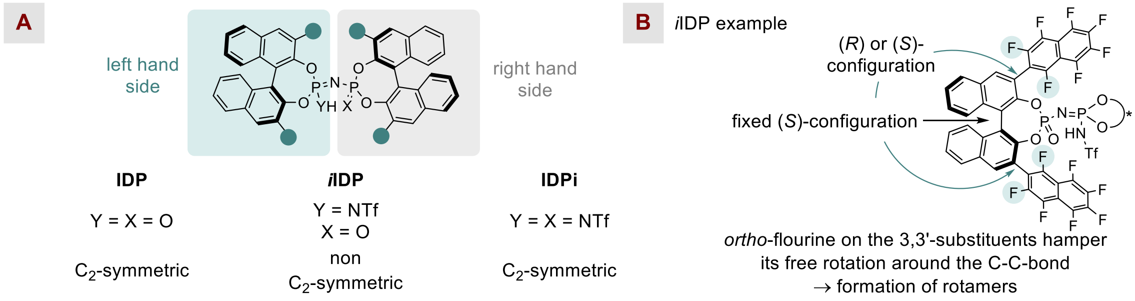

Performing ^{31}P NMR spectroscopy on which the following discussion will focus, the observed signals for IDP and IDPi are singlets and for *i*IDP a set of doublets, respectively (e.g. see *i*IDP **S9**).

However, if the free rotation of this bond is hampered, for example because of large *ortho*-substituents on the 3,3'-substituent or a highly confined environment, a rotameric mixture of catalysts can be obtained. In the specific case of perfluoronaphthyl as 3,3'-substituent, the two F-substituents in the *ortho*-position hinder its free rotation, which ultimately results in the formation of rotamers and a complex NMR spectra of each desired nuclei (^{1}H, ^{13}C, ^{19}F, ^{31}P).

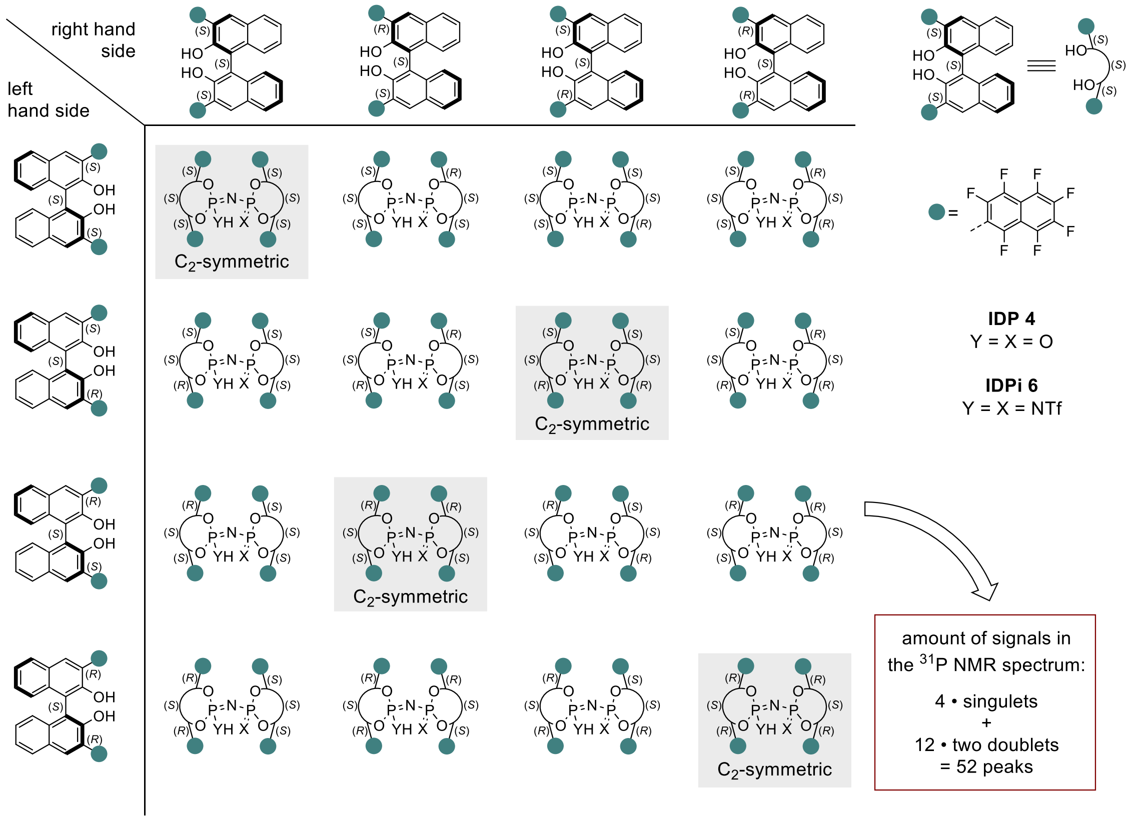

Figure S12: Theoretical analysis on the expected amount of signals in the ^{31}P NMR spectrum of the rotameric mixture of IDP 4 or IDPi 6 based on their symmetry. A total amount of 52 peaks could be rationalized for the catalysts **4** and **6**.

A theoretical analysis on the amount of expected signals in the ^{31}P spectrum of these rotameric mixtures, is shown for C_2-symmetric catalysts **4** and **6** and for non C_2-symmetric catalyst **5**, in the following figures S12 and S13. Since the configuration of the employed BINOL is a (*S*)-configuration and the 3,3'-substituents can adapt both (*R*)- and (*S*)-configurations, four different rotamers can in theory be destinguished (*S,S,S* , *S,S,R*, *R,S,S* and *R,S,R*). In the case of C_2-symmetric catalysts, IDP **4** and IDPi **6**, 16 different rotameric catalysts may be present in the mixture, from which four rotamers are actually C_2-symmetric and 12 rotamers are non C_2-symmetric. Therefore, one could expect 4 singlets and 12 sets of two doublets leading to a total amount of 52 signals in the ^{31}P NMR spectrum.

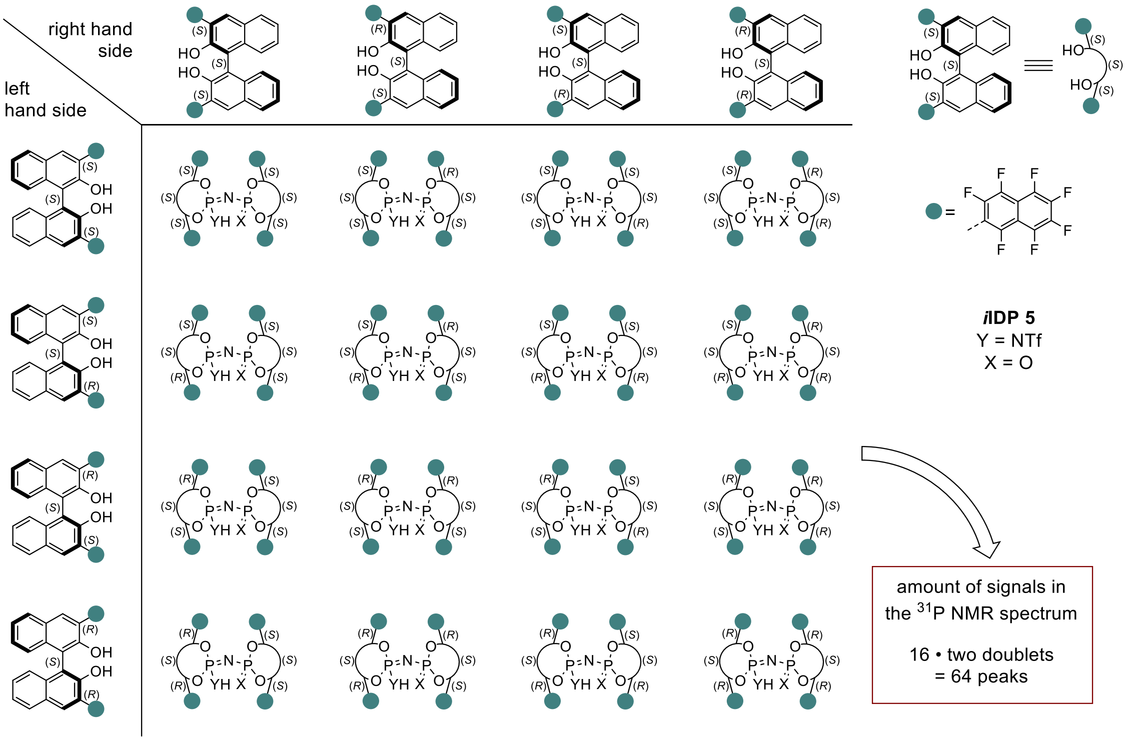

Figure S13: Theoretical analysis on the expected amount of signals in the ^{31}P NMR spectrum of the rotameric mixture of *i*IDP 5 based on its symmetry. A total amount of 64 peaks can be rationalized for the catalyst **5** in the ^{31}P NMR spectrum.

In the case of non C_2-symmetric *i*IDP a total of 16 sets of two doublets could be expected ultimately resulting in 64 peaks.

A detailed analysis of the corresponding NMR spectra, especially ^{31}P spectra, indicates that the observed signals with their corresponding shifts strongly depend on the equilibrium established between different rotameric species, the water content, and concentration and equilibration time (= time of catalyst in solution) of the measured probe. Due to these intrinsic factors and complex spectra, a standardized analysis via NMR spectroscopy for these reported catalysts **4**–**6** is not

representative and we decided to resort on further analytic measurements to guarantee high purity of the desired catalysts.

We carried out LC-MS measurements for all catalysts, for which the NMR analysis was difficult due to the presence of rotamers. Exemplarily, the LC-MS traces of *i*IDP **5** and IDPi **6** are shown in figure S14. These LC-MS traces show two and three peaks, respectively, which represent different rotamers of the desired catalysts indicated by the same corresponding mass.

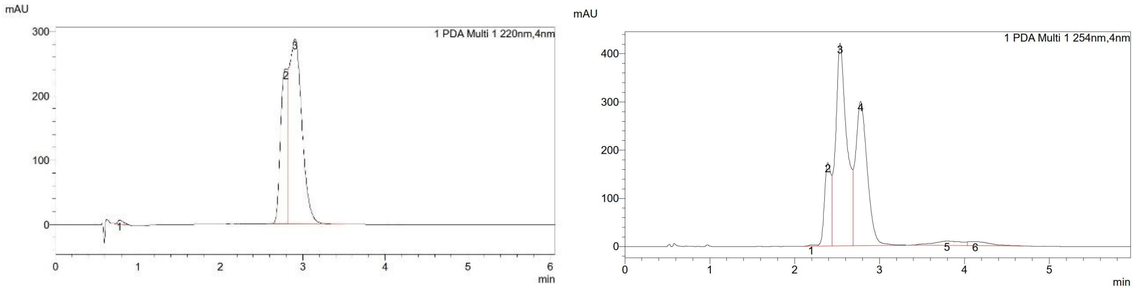

Figure S14: LC–MS traces of catalyst 5 (left) and 6 (right) after acidification. For the integration of both LC-MS traces see chapter 1.4.2. (Left) For catalyst **5** the peaks 2 and 3 show the exact same mass spectrum (m/z = 1815 [**5**–H]$^{-}$). (Right) For catalyst 6 the peaks 2–4 show the exact same mass spectrum (m/z = 1946 [**6**–H]$^{-}$).

2.3. Computational methods

2.3.1. Computational details

Unless otherwise specified, all calculations were carried out with a development version of ORCA based on version 5.0.3.[33]

Initially, a preliminary exploration of the chemical space was carried out at the GFN1-XTB and GFN2-XTB level[34,35], which showed that these schemes provide similar results for the systems investigated in this work. The final structures were computed by means of geometry optimizations at the DFT level using the PBE-D3(BJ) exchange-correlation functional[36–38] in conjunction with the def2-svp basis set[39] This functional/basis set combination has been extensively used in geometry optimizations for a number of different chemical transformations catalyzed by organocatalysts of similar size and nature[40–42]. In the present case, environmental effects were modelled using the implicit solvation model C-PCM[43] (PhMe), together with the gaussian charge scheme[44]. The resolution of identity (RI) approximation[45] was used in the Split-RI-J variant[46] together with the corresponding auxiliary basis set[47]. This level of theory is denoted hereafter as "PBE-D3 + C-PCM(PhMe)/def2-SVP". The nature of the low-energy stationary points was verified by computing vibrational frequencies. All intermediate structures were proven to be true minima, with no imaginary frequencies. A single imaginary frequency was identified for the transition states.

For the key transition states leading to the formation of the stereocenters (**TS$_{AB}$**), a more sophisticated conformational sampling procedure was used.[40] In this case, the PBE-D3 + C-PCM(PhMe)/def2-SVP structures obtained from our preliminary exploration of the chemical space were used as input for the CREST algorithm, as it is implemented in the XTB code (version 6.4.1).[48] Separate CREST calculations were carried out for the transition states leading to the different enantiomeric products. This conformational sampling procedure was carried out at the GFN1-XTB level in the presence of geometrical constraints, as detailed in ref.[40] .In the present case, the forming C-C bonds were selected as geometrical constraints. For the MTD-GC(RMSD)/GFN1-xTB simulations, the default parameters and thresholds were used. The SHAKE algorithm was used to restrict the C-C bonds, with an MD time step *dt* of 5 fs. RMSD was used to evaluate structural similarity. For **TS$_{AB}$ (major)**, this procedure led to the identification of 98 transition state conformers within 6 kcal/mol, 14 of which with unique structural features. For **TS$_{AB}$ (minor)**, 227 transition state conformers within 6 kcal/mol were identified, 77 of them with unique structural features. The unique transition states were then re-

optimized at PBE-D3 + C-PCM(PhMe)/def2-SVP level via a series of constrained/unconstrained optimizations, as detailed in ref.[40] Frequency calculations were carried out only for the low-lying transition state conformers (8 frequency calculations were performed in total for this step).

In all cases, free energy corrections were computed at the same level of theory used for the geometry optimizations at 258 K. Electronic energies were then refined at the M062X +C-PCM(PhMe) level using the def2-TZVP basis set. The RIJCOSX approximation was used for the exchange integrals in this case.

2.3.2. Free Energy Profiles associated with the minium energy pathway – connecting the substrate with the enantiomeric products experimentally detected

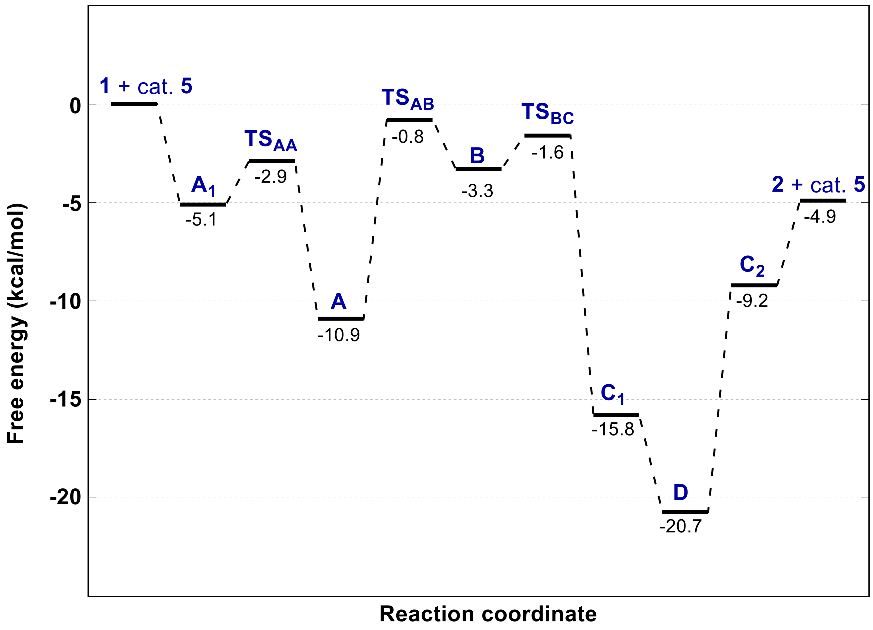

Figure S15: Free energy profile associated with the formation of the minor enantiomeric product. All energies are in kcal/mol.

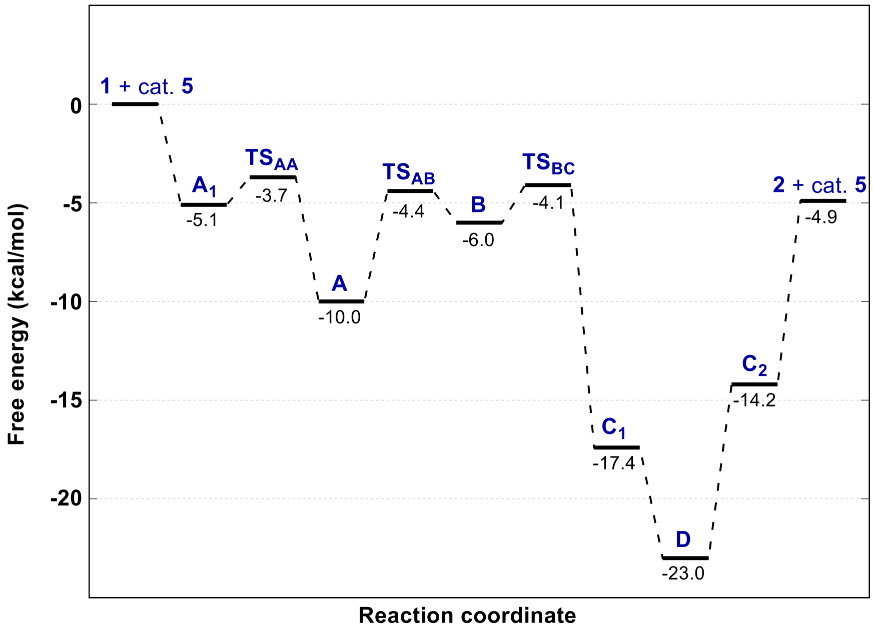

Figure S16: Free energy profile associated with the formation of the major enantiomeric product. All energies are in kcal/mol.

2.3.3. Proposed catalytic cycle of the Brønsted-acid catalyzed cyclization of neral

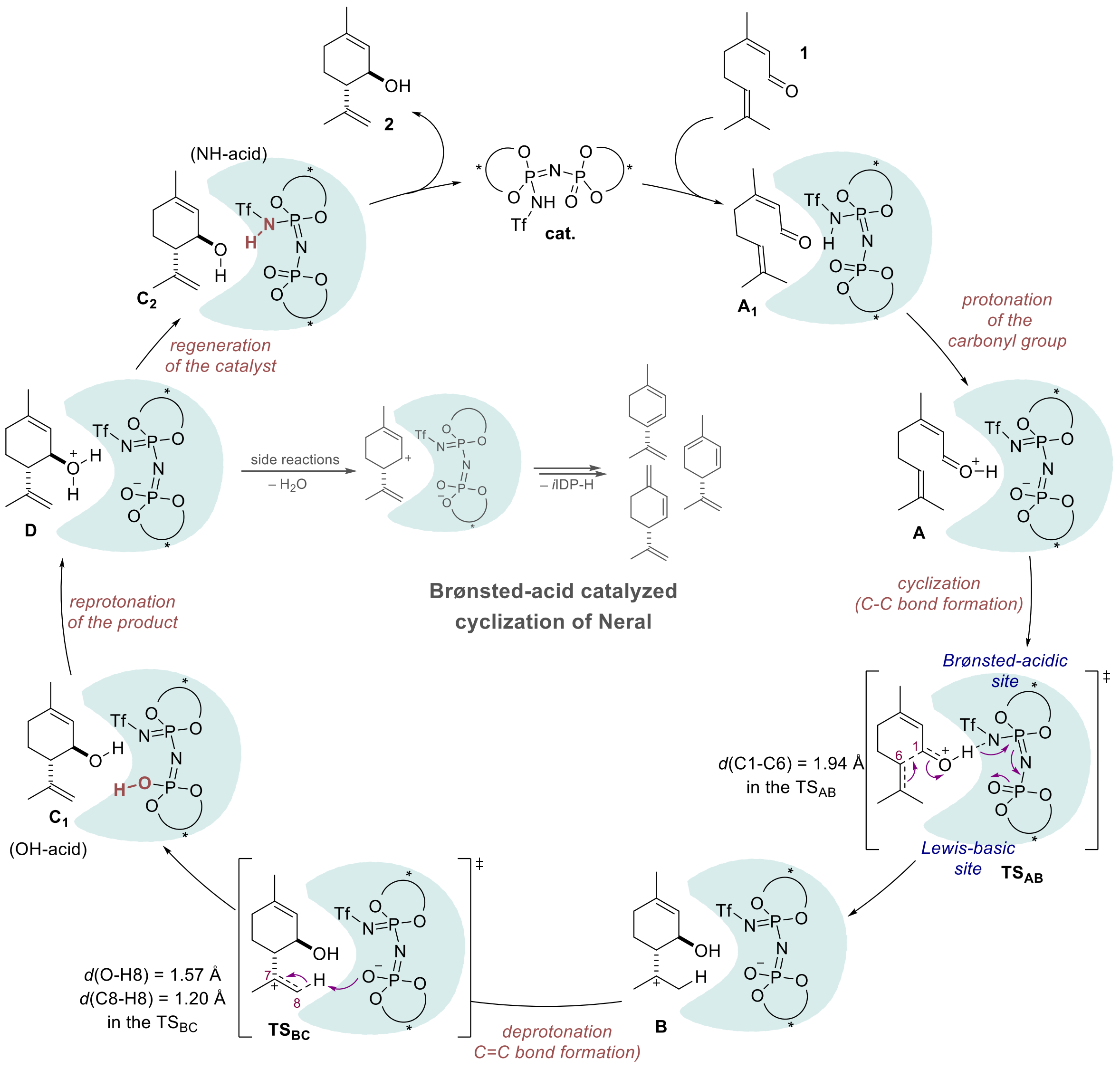

Figure S17: Proposed reaction mechanism for the Brønsted acid-catalyzed cyclization of neral (1) to isopiperitenol (2) using confined *i*IDP catalyst 5 based on experimental and computational data. Structure D is the starting point in the formation of decomposition processes towards shown trienes. Structure C_1 and C_2 are different concerning the position of the acidic proton on the catalyst (OH vs. NHTf-acid). The key bond forming and bond breaking distances are extracted from the corresponding computed structures (for corresponding xyz-coordinates see section 2.3.8)

2.3.4. NCI-Plot

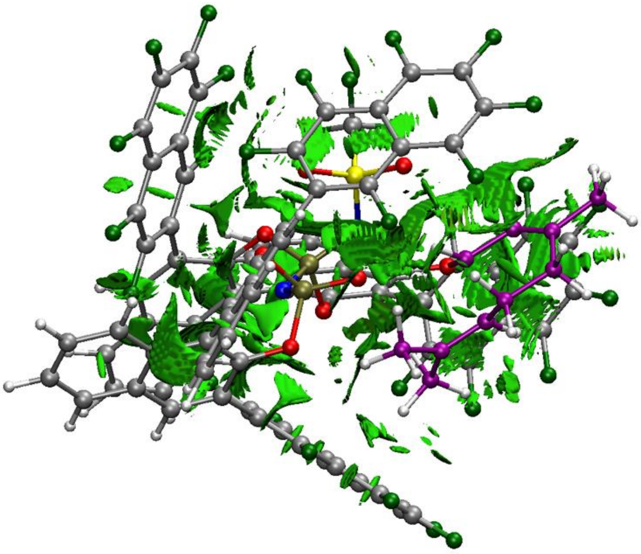

Figure S18: Noncovalent interaction (NCI) plots computed using the NCIPLOT program[49]. The density isovalue for the NCI plots is 0.5 a.u..

As shown in Figure S18, there are strong attractive interactions between the substrate and the perfluorinated naphthyl substituents, which play a major role on the selectivity. Analysis of the dispersion energy at the DFT level indicates that these interactions preferentially stabilize the cyclization transition state leading to the major trans-product by 2.0 kcal/mol with respect to the one leading to the minor trans-product. These calculations were carried out by computing the relative electronic energy between these two transition states at the PBE level of theory with and without Grimme's -D3 dispersion correction. As discussed in ref.[50], the D3 correction associated with the PBE functional provides a lower bound for the dispersion energy at the gold standard CCSD(T) level.

2.3.5. Initial studies on the E1-elimination pathway

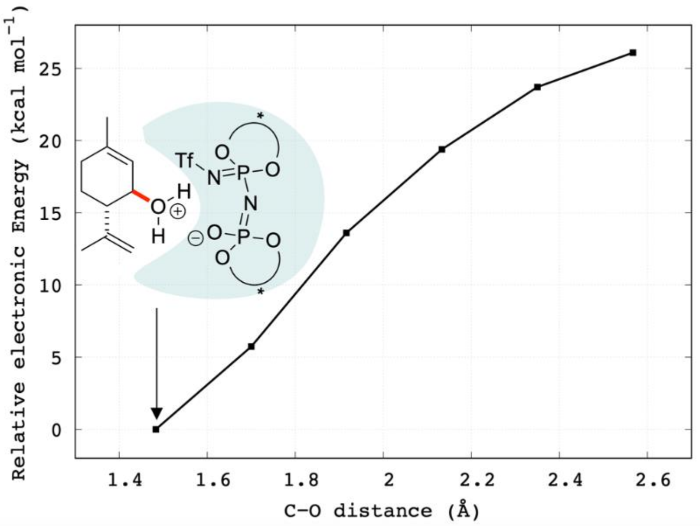

Figure S19: Energy profile associated with the elongation of the C···O distance (emphasized in red in the figure) starting from protonated product in the cavity of the iIDP anion, shown as structure D. All other internal coordinates were allowed to relax during the scan. Calculations were carried out at the PBE-D3/def2-svp level.

As shown in Figure S19, increasing the C-O distance in structure D from the equilibrium value of 1.48 Å to 2.48 Å leads to a steep increase of the electronic energy. These initial results on the decomposition pathway suggest that an E1-elimination pathway via the formation of the allylic cation at C1 atom features a large activation barrier and therefore appear as an energetically unfeasible process.

2.3.6. Alternative pathways

The computed minimum energy pathway describes a stepwise mechanism, in which the carbonyl group of the substrate is initially activated through protonation (**TS$_{AA}$**), leading to the formation of intermediate **A**. In the following cyclization step (**TS$_{AB}$**) the stereocentres are formed. The resulting, highly unstable carbocation intermediate (**B**) quickly loses a proton, leading to the formation of the final product, as described in the main manuscript.

It is important to note here that the carbocation intermediate **B** can also form a covalent adduct with the catalyst upon carbon-oxygen bond formation. The energy of this covalent adduct for the pathway leading to the minor and major enantiomeric product is –19.7 kcal/mol and –19.9 kcal/mol, respectively. This reaction is essentially “barrierless”, *i.e.*, the free energy of the corresponding transition states is nearly equal to that of **B**. However, no low energy pathway was found connecting the covalent adduct and the product. The XYZ coordinates of the covalent adducts and of the corresponding transition states is given below.

Finally, we also explored the possibility of a concerted mechanism in which the cyclization and subsequent proton transfer occurs in a concerted way, without the formation of the carbocation intermediate. However, it was not possible to locate a low-energy transition state for this step.

2.3.7. Calculation of the KIE

The computational estimate for the expected kinetic isotopic effect on the rate constant was computed using the formula:

$$\frac{k_H}{k_D} = e^{-(\Delta G_H^\ddagger - \Delta G_D^\ddagger)/RT}$$

In which $\Delta G_D^\ddagger$ and $\Delta G_H^\ddagger$ denote the free energy barriers for the D- and H-transfer step (TS$_{BC}$), respectively. The computed $\frac{k_H}{k_D}$ ratio amounts to 2.6.

2.3.8. ORCA 5.0.3 input examples

Geometry Optimizations

```
! PBE OPT VeryTightSCF NOSOSCF D3BJ  def2-SVP def2/J  CPCM(Toluene) XYZFile

* xyz 0 1
--Coordinates---
*
```

Transition State Optimizations

```
! PBE VeryTightSCF NOSOSCF D3BJ  CPCM(Toluene)  def2-SVP def2/J   OPTTS XYZFile

%geom
calc_hess true
end

* xyz 0 1
--Coordinates---
*
```

Frequencies and Thermal Corrections

```
! PBE FREQ VeryTightSCF NOSOSCF D3BJ  def2-SVP def2/J  CPCM(Toluene) XYZFile

%freq Temp 258 end

* xyz 0 1
--Coordinates
*
```

Single Point Energy Calculations at the M062X level

! M062X VeryTightSCF RIJCOSX def2-TZVP def2/J CPCM(Toluene) XYZFile

* xyz 0 1

--Coordinates

*

2.3.9. XYZ structures

The labels “major” and “minor” denote structures along the pathway leading to the major and minor enantiomeric product, respectively.

Catalyst

145

C	0.09842501696020	0.94215198361425	4.24415307563880
C	0.66315147695676	2.00984130600578	3.48857877901955
C	-0.11677615325690	3.21979235347547	3.32028908758756
C	-1.39416511888448	3.31237321345354	3.94019391333928
C	-1.91408695756395	2.24595289587965	4.66362600039857
C	-1.16411011384750	1.05289980976260	4.80953770713993
C	0.42001017168496	4.26305129621130	2.51488466838842
C	1.62681817623794	4.09885028135792	1.85460218539903
C	2.39152638231128	2.89748231945450	1.94544023628886
C	1.91967093363673	1.91864495357339	2.81969849805871
C	3.47132154745630	2.67932153352773	0.94981019062197
C	3.37902637560526	1.57910937373033	0.03650116981841
C	4.10227626824547	1.49966794702958	-1.15739297976027
C	4.99562693661259	2.58489187411836	-1.48081607735518
C	5.20758059998136	3.62811708061738	-0.50691711163791
C	4.43214211192048	3.64958714317399	0.68420000882202

C	5.64998521655430	2.69698151809084	-2.74306499612097
C	6.52241409370444	3.74456462229672	-3.00349338009455
C	6.78355752749622	4.73258396452871	-2.01709299266337
C	6.12953089602910	4.67847497897552	-0.79646723183354
C	3.88242125547412	0.34726905148418	-2.07790532496201
C	2.58425946327443	0.00508310499530	-2.46095131130222
C	2.25333348027644	-1.12173805543065	-3.26840431003534
C	3.30365566113514	-1.89879247727790	-3.74235657293842
C	4.65444935527752	-1.60349607039670	-3.41314422957844
C	4.96093116776629	-0.48669570895602	-2.55028582787361
C	5.71472412893078	-2.42759472168785	-3.89434355895022
C	7.02815646555570	-2.18199980212881	-3.52760682770492
C	7.32593048966655	-1.11104832849183	-2.64574523699398
C	6.31872893870343	-0.28653993294398	-2.16662271610007
O	1.50805766118189	0.78014812925380	-1.98937716564957
P	1.04907870340788	0.51331892177324	-0.45752147957924
O	2.46997505028480	0.55810361858811	0.34550196837869
C	0.83222630267074	-1.48299585302284	-3.49032193361927
C	-0.09402273649329	-0.59047481470187	-4.02763764165908
C	-1.47739819700878	-0.89892916706420	-4.17611878366953
C	-1.92963186597441	-2.21814038648482	-3.77961990537169
C	-0.97519288085534	-3.12817936817243	-3.24495923652524
C	0.35514905787463	-2.76602976939341	-3.10334221091845
C	-3.29930322777078	-2.55887854306726	-3.96233559847470
C	-4.18897032200392	-1.65123977165382	-4.52180937082841
C	-3.75375488993976	-0.35485725832412	-4.89225001568103
C	-2.42760669154092	0.01675220067964	-4.71610915148728
F	0.34948097240241	0.60453256552678	-4.44190189674976
F	-1.34168508800416	-4.36230405639756	-2.87228299061388
F	1.20811854236946	-3.65709036335414	-2.57974216850948
F	-3.76774021307063	-3.75764416085372	-3.59007387636151

F	-5.47349169977888	-1.97223037476087	-4.68370974995742
F	-4.64165829725690	0.50680245624224	-5.39073125852368
F	-2.07094385573854	1.25350820803229	-5.07206203580563
F	2.66487767566404	0.81602441935155	2.99668773054512
F	-0.25010605710888	5.40803717912681	2.33926095709000
F	2.03760936492433	5.09008924241425	1.05041644568590
F	-2.12744893255796	4.42690057498665	3.82974225593876
F	-3.11450711826474	2.32709706988110	5.23294009948096
F	-1.69846998244006	0.03211649742836	5.47992788526505
F	0.74069941359361	-0.22110979697542	4.38604036751064
N	0.30646952438927	-0.83803161433113	-0.16537261520481
P	-0.97317860793388	-0.88936132315400	0.85526616609813
O	-1.30589766368903	0.35226538125167	1.64766648180100
O	-0.74773729289550	-2.17728277553416	1.86006008308929
C	-0.65585990099877	-3.43067531503097	1.25455013752046
C	0.64191284411562	-4.01042502323927	1.11429153618417
C	0.74485485500951	-5.21780561868661	0.43075980168196
C	-0.39388236359296	-5.85406387444610	-0.13460228055788
C	-1.70010597897135	-5.26602335778528	0.03558487584421
C	-1.81893705407917	-4.04276340653001	0.78094114630040
C	-0.26541818262791	-7.05476949643722	-0.89424381416996
C	-1.37298598979227	-7.64346369715859	-1.48406431115702
C	-2.65433321301547	-7.04607258530819	-1.34300782019847
C	-2.81551399166504	-5.88541411698391	-0.60068801568367
C	1.82669207024528	-3.31813209922350	1.67382573232736
C	2.96190920693793	-3.05341369393543	0.90836769857737
C	4.10835546419719	-2.38191485262933	1.42357424584729
C	4.05957700052312	-1.89076278860977	2.78449833901806
C	2.91541069194451	-2.19472771055710	3.57167311242452
C	1.84737800799491	-2.89595452138348	3.03267206380193
C	5.30327920512467	-2.18682111514601	0.66964150007321

C	6.36389077855135	-1.45825508727580	1.19244501636043
C	6.28041036054636	-0.90876504435941	2.49708929084726
C	5.15593292851987	-1.13008031830684	3.28091770006481
F	7.30757199795303	-0.19564119767440	2.96374945257551
F	5.13387355888459	-0.61236907839262	4.51398643430129
F	5.44074530910325	-2.72623736991062	-0.54674324116935
F	7.49197618177047	-1.27947323914539	0.49724285880779
F	2.96716848670995	-3.42322354462423	-0.38268046828674
F	0.81693307914744	-3.19943585905415	3.82843423493497
F	2.86023796684551	-1.83132999114902	4.85846952532297
C	-3.12058670399804	-3.35973858552966	1.00725165894071
C	-3.26360096015264	-2.04610926390963	0.56340409729277
C	-4.42885980098595	-1.26209237562537	0.80693386027536
C	-5.48342532853961	-1.84754643072202	1.49494010695655
C	-5.40196925553038	-3.18582046181239	1.97376746247504
C	-4.20041676566800	-3.95541634354765	1.74990252045957
C	-4.11514148101230	-5.26041286800150	2.31945622956747
C	-5.17689160070295	-5.79647734076365	3.03470635701699
C	-6.37319533839423	-5.05365830946386	3.22116222872051
C	-6.47810216296894	-3.77107724253218	2.70550114001493
O	-2.19907943487261	-1.44507554037116	-0.10788739034453
C	-4.44858625449450	0.15267828935286	0.36238915646888
C	-4.36886143370510	0.47267142478204	-1.01593922463969
C	-4.32514702377499	1.77843231650012	-1.47594888059946
C	-4.34659253819895	2.86646015857426	-0.55792593949391
C	-4.42231698263971	2.57301417380070	0.86015118935532
C	-4.47504637016781	1.20789095401252	1.26742493861909
C	-4.43565523629851	3.65773974927497	1.78332556533489
C	-4.31740451320294	4.97074361286225	1.34733759823574
C	-4.23828608299742	5.25457985095566	-0.03875506175591
C	-4.25845377942021	4.22502276055380	-0.97034264482965

F	-4.33572412367700	-0.53360644463138	-1.90262024299301
F	-4.26243054670832	1.99249637054701	-2.79752666890517
F	-4.50959055522480	0.92162720102690	2.57799224277907
F	-4.55629721024290	3.43894002879381	3.09733913006107
F	-4.30388348665405	5.98068814760248	2.21901366717321
F	-4.12746555538675	6.52420869981716	-0.43369920724662
F	-4.16035594987685	4.54513161351192	-2.26577690101516
N	0.22631136765909	1.95088439361045	-0.10190814854311
S	-0.65556024549575	2.90456776119035	-1.21731855771607
O	-1.35620992421628	2.08151793354331	-2.21495200176072
C	0.73211570399352	3.84499197963352	-2.16651186895491
F	0.92780109217580	3.26160669818566	-3.34180398451360
O	-1.33816752535256	3.89942938065414	-0.37521054098335
F	0.28033278787735	5.08504065756232	-2.33497698713092
F	1.86438752565800	3.86446047421519	-1.46482704752545
H	6.56853404524931	0.51706355994550	-1.46257233309424
H	8.36358235865671	-0.94133911991214	-2.32262313949368
H	7.83769539533622	-2.82598153104262	-3.90258363049168
H	5.46332462243674	-3.27047723223626	-4.55621684988948
H	3.08983813150598	-2.77626276890930	-4.36969686915152
H	5.45207856382915	1.94592440423864	-3.51945639561683
H	6.29207698027532	5.45816836278188	-0.03654764356142
H	4.54633326217553	4.50011155808756	1.37110181656670
H	-3.80581913572606	-5.41618809446894	-0.52178222058631
H	-3.52580177753343	-7.50191732737946	-1.83677771373409
H	-1.26099542216138	-8.56566694658597	-2.07391695746067
H	0.73600690636144	-7.49674453256599	-1.01220470108948
H	1.73123394813408	-5.68677462353517	0.30365584300792
H	-3.19123033701715	-5.84091635681060	2.19243125935563
H	-5.08807809641376	-6.80477843792465	3.46663737113401
H	-7.20882660237777	-5.49284501861343	3.78654115274808

H	-7.39135283319259	-3.17617001923591	2.86112219495491
H	-6.39430778191322	-1.26282868032461	1.69175124358297
H	7.48680214226181	5.55072563985531	-2.23312163163355
H	7.01196881482938	3.81195157667210	-3.98669615818179
H	-0.39043156263831	1.78036194196043	0.73765907783574

Neral (1)

27

H	3.71409126621619	-0.88421986478253	0.53210544587663
C	-1.81871875713043	0.31247136203527	0.11574074808181
C	-2.88929974619220	-0.07194045113514	1.09599269105570
H	-3.89195120564187	-0.00246300296858	0.62084105597531
H	-2.88058566119029	0.56037458930670	2.00390224727301
H	-2.77178425476836	-1.13478191967211	1.40251876515175
C	-1.68605565222982	-0.58733722838938	-1.08725301724426
C	-0.96133337675568	1.34078979990454	0.38711772907985
C	-0.65328258195987	-1.73509772373931	-0.83598280210832
H	-1.37355879848142	-0.02254738716348	-1.98848394902117
H	-2.67176618001289	-1.04297880590209	-1.31371304082935
C	0.76799887692519	-1.25662882332897	-0.87561453951015
H	-0.80842514970482	-2.49728662481079	-1.62842506316811
H	-0.89308372359518	-2.23060246334916	0.12744807056891
C	0.14630500898985	1.77731100138539	-0.46374229239494
H	1.17946498610052	-1.09326089099450	-1.88892536797978
C	1.56936644951509	-0.92940578934564	0.17129405260763
O	1.02787989092493	2.55273875138515	-0.09035863890781
H	0.14652749444258	1.37899227883712	-1.51789365208618
H	-1.04981577029760	1.88409844754943	1.34403708645162
C	1.16965965330779	-1.05187450105579	1.61757420939547
C	2.94000357351249	-0.35131216336137	-0.06061550707938

H	0.15392719238920	-1.46418193399944	1.76141803147897
H	1.20397483876727	-0.05331038710133	2.10428778044357
H	1.89030932604346	-1.69398436558501	2.16904520760472
H	2.95771758119554	0.71201257785491	0.26877678277883
H	3.22879985423033	-0.37982345437390	-1.12933509249430

A1

172

C	-6.28122044080753	1.55478675416579	-2.93258021175671
C	-5.14343409306719	1.89830252249086	-3.65060034606686
C	-4.01027250609364	1.03724907787843	-3.68126608457276
C	-4.06130135772144	-0.21433894191719	-2.95316122097578
C	-5.24483781344714	-0.52713240195157	-2.22364676965990
C	-6.33215139109891	0.33504614552677	-2.21350091202528
C	-2.91788927577230	-1.06332785835587	-2.99629758322208
C	-1.76292713593144	-0.76485862534586	-3.71561003920097
C	-1.74548402031354	0.47267744178294	-4.41585923242489
C	-2.82148252623655	1.34840596635250	-4.39734324946613
C	-0.62770820176023	-1.71383055558409	-3.81963174153968
C	0.70480121975244	-1.28798616871060	-3.52748599586940
C	1.82172332376507	-2.09871295467256	-3.75675288882229
C	1.61141960346013	-3.46570268069153	-4.15033271343899
C	0.26444228478676	-3.93119982548365	-4.37382197236569
C	-0.82368823499801	-3.02608623380683	-4.23700582836076
C	0.04611144395327	-5.29822219823112	-4.72086370071546
C	1.10921685302414	-6.18226375156528	-4.82512864118732
C	2.43412520346042	-5.73001065258757	-4.57935328635616
C	2.68022147335006	-4.40449428032087	-4.25117664822151
O	0.87777248766934	-0.02757253442976	-2.97718830951602
P	1.23696869829273	0.03290099501632	-1.34421597958494

N	0.02806893493676	-0.79377392376598	-0.63398984458415
P	-0.70582380638900	-0.63518027379265	0.74117849231154
N	-0.23389242402700	0.54643377777974	1.82186563192975
S	1.34956461241393	0.58816904263856	2.34839985376452
C	1.15661038896411	0.10369089260169	4.16737850951674
C	3.17126636980430	-1.50670916383255	-3.54784036704985
C	3.45875718576377	-0.92054415517034	-2.31415732183065
C	4.66077201248126	-0.18977879164048	-2.06465897929208
C	5.60918827485344	-0.11292468331755	-3.07418620617016
C	5.38617653951372	-0.71333301851147	-4.34627033457829
C	4.14112550952677	-1.39802264840894	-4.60672863978084
C	6.35783299217357	-0.61074111714178	-5.38588258007182
C	6.11216468521242	-1.13589750985627	-6.64520186488067
C	4.87097362573204	-1.77121610156954	-6.91559400063810
C	3.90802338779549	-1.89554907350107	-5.92377991482329
F	-0.66678773521936	0.80804575572857	-5.13294690311338
F	-2.71457814726866	2.50193234176301	-5.07679280558946
F	-2.95397383450417	-2.21672127820211	-2.31211017444983
O	2.52390799136786	-1.02274437490466	-1.29511255559172
C	4.82379855254202	0.49612755052893	-0.75474516967938
C	5.08969373386911	-0.25694310542505	0.41273523985409
C	5.09898023344599	0.30679729876032	1.67776312979766
C	4.87542271832456	1.70063310299285	1.84560523430085
C	4.66647346627137	2.51309100964736	0.66284553024443
C	4.63746162534601	1.86569637219413	-0.60783415426557
C	4.46381330780319	3.91298782762570	0.82800916673889
C	4.40874702196508	4.48567170140534	2.09116193322208
C	4.56239049509777	3.68116586079430	3.24620023389959
C	4.80029898646397	2.31907687492629	3.12559434657314
F	5.31514469435733	-1.57065672459552	0.27814822511472
F	5.31455814716817	-0.48883573343632	2.73799433773491

F	4.38114062055049	2.59501064672169	-1.70303475106273
F	4.92785330651680	1.59824362341194	4.24682549435224
F	4.29649253626943	4.71898842623175	-0.23003255894696
F	4.18539171162775	5.79479769911062	2.23899824121949
F	4.45204080985182	4.25207667412033	4.44910169001300
F	-5.13958174982327	3.06510645138938	-4.30508178239055
F	-5.33680676887420	-1.64818040357103	-1.49537230827678
F	-7.42779241688501	0.04010740513320	-1.50892206466618
F	-7.33593119282786	2.37235910570382	-2.89759571813863
O	1.81721753358211	1.98405654278577	2.37212753532652
O	2.12712253290690	-0.50347517266767	1.72646252861955
O	-0.72581087891018	-2.00881974554232	1.63441390790067
C	-1.12719778105658	-3.12994453447864	0.89696067283701
C	-2.48176158321133	-3.26187989775622	0.59906214557382
C	-2.87737093109687	-4.30823381622394	-0.31153616597793
C	-1.86403815812844	-5.19402693472747	-0.83114360200790
C	-0.51526461138470	-5.05852947232881	-0.40444434118609
C	-0.12160039856156	-4.04119233532699	0.45966481074008
C	-2.22898958318076	-6.20311975791129	-1.77267630854686
C	-3.54133350481200	-6.33519407100852	-2.19807471592470
C	-4.53839051565012	-5.45767325160707	-1.69401291042804
C	-4.21601287537555	-4.47116073853385	-0.77315920530550
C	-3.45948869149664	-2.34254750941327	1.25023942921965
C	-3.33834791552018	-0.94761437075395	1.17589485520358
C	-4.28621539948617	-0.06174102529915	1.78098411181298
C	-5.37169052130435	-0.61756260137124	2.45171740174522
C	-5.51801822317829	-2.01849989387552	2.61263848627954
C	-4.52837891930412	-2.89871717124540	2.04626871189488
C	-6.60197475774609	-2.56210726491183	3.36545756991236
C	-6.69139452818948	-3.92552886830435	3.59343466196147
C	-5.68479067540485	-4.79132117785316	3.08821514372438

C	-4.63120179012282	-4.29309358525740	2.33521883411935
C	1.27890698517318	-3.90514861816804	0.91601391234245
C	1.60987276181268	-3.72973465993608	2.25976963216285
C	2.95120492216680	-3.61635185290638	2.72515612517218
C	4.02506253823029	-3.77954878444705	1.76709802496983
C	3.68260509001779	-3.97392239684031	0.39922487192774
C	2.35795639899518	-4.01150162899802	-0.00260149133770
C	5.37018880319218	-3.72222514553807	2.22787890619070
C	5.65673307431332	-3.46677791808407	3.56207328172810
C	4.60836217504704	-3.27462621954329	4.49553026009013
C	3.28387589439294	-3.35371893599310	4.08630430425957
F	0.61798651321997	-3.70219874240222	3.16176926594026
F	4.63160987264211	-4.09803238363401	-0.53885363681574
F	2.09505136483443	-4.16080974463040	-1.30806679837093
O	-2.29817247585507	-0.35674539386437	0.45480129265624
C	-4.14901821376007	1.41869103020301	1.77328709601447
C	-4.08961351957429	2.18568232553361	0.61381776596537
C	-4.03754017575925	3.60964963713913	0.61166372160027
C	-4.02682708145954	4.29419859379851	1.88946161694912
C	-4.10132359991682	3.51129931886085	3.07339436327570
C	-4.15370223516572	2.12590924991877	3.00894770107839
C	-3.85362554925104	5.70830366376557	1.91071597212349
C	-3.68997914302390	6.42400634474534	0.73170035183788
C	-3.71773306584680	5.75921176824308	-0.52012192668803
C	-3.88775592722191	4.38311181394535	-0.57714099642817
F	-4.08028827408838	1.54716044305655	-0.56558926527970
F	-4.07754905425867	4.09250938017417	4.27827925686027
F	-4.18660591486764	1.44082415288319	4.15776048490896
F	-3.83906048843465	3.80364842985583	-1.78270466223777
F	-3.51027942077493	6.46474384714600	-1.63380593523817
F	-3.45558350271920	7.73657424790085	0.76231379495625

F	-3.77820500066591	6.37958716744567	3.06531038560522
F	6.39448592540971	-3.89152016509232	1.38463531277754
F	2.33261273017507	-3.16838128571660	5.00954551422162
F	4.91353783580507	-3.01674683798967	5.76962014138082
F	6.92031744493987	-3.39010626796283	3.98390954992545
O	1.57482549763966	1.43766278917497	-0.94958020944165
H	-4.99406750107681	-3.78762789671807	-0.41281542546173
H	-5.57740904889728	-5.55381544920952	-2.04381293393201
H	-3.81229317971146	-7.11264038147283	-2.92823728864761
H	-1.44019664585984	-6.86614024481455	-2.15982965522029
H	0.23022656846363	-5.77361482260182	-0.77966016653480
H	-3.85708401200290	-4.97827184643535	1.96619570800537
H	-7.35415786045032	-1.87115040658492	3.77627308604247
H	-6.12364550429986	0.04598988450105	2.90327139084377
H	3.70390311624217	-4.07201789681330	-4.02937155730143
H	3.27237603507679	-6.44062093136328	-4.63948190881953
H	0.92970249185797	-7.23593847019626	-5.08718250868371
H	-0.98753001173590	-5.63738843820838	-4.89045182702692
H	-1.84058701676893	-3.38225858307385	-4.45773260700748
H	2.94794310381150	-2.37737993999115	-6.15285146523906
H	4.66692095478282	-2.16363002060539	-7.92342507710688
H	6.86905604376678	-1.04925662755811	-7.43924176072782
H	7.30539957551767	-0.09391688405030	-5.16815291990890
H	6.54502712221479	0.43849926822062	-2.89872474079468
H	-7.52631230364335	-4.33690929393207	4.18018693798229
H	-5.73527763136064	-5.86957500681079	3.30226200697903
F	0.48258246421727	-1.04137523051024	4.26450222198174
F	2.38266723220946	-0.04254647884320	4.68116967144568
F	0.50480294532612	1.07233662446040	4.81708287114649
H	1.77825145584577	5.68948774695466	3.86504335106738
C	-0.58154285844618	3.91996906816393	-1.41998514029876

C	-0.37501363532395	3.35063546506805	-2.78341787076424
H	-1.12967370981183	3.76893027834291	-3.48423244699803
H	-0.43578043172638	2.24984873643608	-2.79946301189476
H	0.61953592093087	3.65226938484341	-3.17539612497401
C	-0.25225098603623	5.37168521723580	-1.24305651416385
C	-0.91621817899926	3.10550377259798	-0.36520143044236
C	1.18364275329856	5.54222402230360	-0.64065303068615
H	-0.97224752928994	5.88367606273234	-0.57375349911138
H	-0.29685061987090	5.88692529905239	-2.22261055920824
C	1.31130683139903	5.04279826327314	0.76974638603688
H	1.43063686055885	6.62269955038930	-0.70063938968044
H	1.90805000688519	5.01157077018561	-1.28947972849851
C	-1.13501848681758	3.58185853045016	0.97007195655471
H	1.72517916930367	4.03099305259735	0.90806533049160
C	0.91047514948678	5.71148749795420	1.88733785341509
O	-1.20776792117695	2.85698373366419	2.00299280600321
H	-1.33982054246848	4.66294495860575	1.10943303012423
H	-0.99992466265162	2.02524144191830	-0.54387703642986
C	0.24614321591063	7.06186636962818	1.84734065064203
C	1.05747138294908	5.10274729007774	3.25470087599156
H	0.35911930741470	7.58178021912578	0.87732350349693
H	0.63902744322122	7.72275924681011	2.64789451885034
H	-0.84581112158643	6.95723845647708	2.04601484205287
H	1.40409800408227	4.05463063622076	3.20912665422332
H	0.08789249551933	5.12538627511182	3.79842161573936
H	-0.76760555676950	1.61212823343389	1.87696839300336

TS_{AA}(major)

172

C	-5.04626829028855	3.67193128834033	1.12005838011053

C	-4.96082826482288	2.28608805844551	0.80366628713225
C	-5.38001786990687	1.84656167141387	-0.51224228471832
C	-5.88077812536875	2.81539282151297	-1.42810935253613
C	-5.92375363564821	4.16303653170841	-1.09382545332795
C	-5.52355983910371	4.59014711842897	0.19493382993201
C	-5.25739612752979	0.46321902688335	-0.83220654368814
C	-4.68348765646174	-0.41998558781596	0.06837786259756
C	-4.25272022966059	-0.01801793380985	1.36039025046661
C	-4.41953380902031	1.32028888028303	1.70240354776554
C	-3.72929639764845	-1.02720589385658	2.31199659212441
C	-2.56988675739126	-1.79680606731024	1.99520961149659
C	-2.10127581058589	-2.82828441501104	2.80999069327875
C	-2.74559130098905	-3.04497057137022	4.08147417914585
C	-3.90140492064449	-2.25409602509129	4.42744072612568
C	-4.37815982499627	-1.27420689019063	3.51526970608155
C	-2.25349940947751	-3.97726385832828	5.04260027279977
C	-2.89086615426402	-4.14542805587875	6.26394183748434
C	-4.05110270551721	-3.39247575142881	6.58679323469814
C	-4.54184271399141	-2.46101839482017	5.68535053814402
C	-0.90823055283462	-3.62321501451888	2.41125018632101
C	0.29247774944140	-2.95968697505469	2.17322802698240
C	1.53188081477858	-3.62694508345520	1.94683732781945
C	1.51103662494421	-5.01555240239556	1.87135662021091
C	0.29982472227015	-5.75004964289748	2.00286882888377
C	-0.93428551657321	-5.05489328581200	2.27869159542840
C	0.28180110256598	-7.17016765462195	1.86499991089629
C	-0.90437069249405	-7.87972203032962	1.97211373298291
C	-2.12317783815980	-7.19148986901894	2.21638102772270
C	-2.13829931566759	-5.81226363020939	2.36803996619071
O	0.29588612312861	-1.56989804867728	2.18302534934593
P	-0.41542186366521	-0.74580518778192	0.95796283256948

O	-1.88579939915538	-1.48303702427033	0.81707581042946
C	2.78197713674448	-2.84432318417970	1.81627381238759
C	3.16480124166320	-1.91662855145659	2.78540066998849
C	4.35551970575185	-1.14116564741351	2.69463986067687
C	5.24616649795811	-1.38592983662521	1.57831349037397
C	4.86223513175050	-2.35278266629982	0.60748946392949
C	3.66221851481828	-3.03872273819757	0.71839046552995
C	6.46244926972678	-0.65156564515767	1.49671936030242
C	6.78052448183982	0.30401606679804	2.45289475914179
C	5.89722128815565	0.56475344533119	3.52911890486632
C	4.71117706615132	-0.14668486040477	3.65149162000313
F	2.39271017937772	-1.78239873731494	3.87027536871184
F	5.65500576515635	-2.61442129287026	-0.44149570526522
F	3.33356029728259	-3.92095018586298	-0.23701579778104
F	7.32010854517864	-0.84224221843032	0.48530250242174
F	7.91271131420453	1.00588080117009	2.36102508965158
F	6.21727059393936	1.51253281800531	4.41317812373757
F	3.91492641474332	0.13920454608288	4.68416335471157
F	-4.03361275834320	1.71955029532739	2.92058955941766
F	-5.69359929153123	-0.01380812028407	-2.00815900029072
F	-4.57302533307674	-1.71009491499919	-0.26980707782668
F	-6.27582642061277	2.46193135280539	-2.65908774909573
F	-6.32560958287421	5.07499514116915	-1.98237621747976
F	-5.54506035321779	5.89521689455686	0.48339736817986
F	-4.62393061840551	4.14331704776265	2.29797457536450
N	0.30471013976644	-0.93477363666206	-0.42540591654816
P	0.93525196911517	0.15485362944061	-1.44326288744545
O	0.33586610526027	1.51941283041662	-1.62165466946347
O	1.01242851206296	-0.61970920369663	-2.91188189735188
C	1.85926411056522	-1.70826815600276	-3.04502165165670
C	1.27652357643181	-3.01110614555708	-3.04075664065737

C	2.11284978565127	-4.11070355359792	-3.17552229186591
C	3.52070673723237	-3.95764041725202	-3.30891029033691
C	4.10172882202036	-2.63613739489300	-3.30051789830512
C	3.23339337190554	-1.49281909672061	-3.18426494386430
C	4.37330287490094	-5.09555206210230	-3.42027248847426
C	5.74849406060334	-4.94796619019476	-3.51073396331668
C	6.32361364838148	-3.64984581867059	-3.47515503575691
C	5.52167391250336	-2.52294242423280	-3.36998677964782
C	-0.19356365541693	-3.15615133968909	-2.88889725207235
C	-1.06794589802537	-2.69461480148146	-3.86644276365621
C	-2.48390634366866	-2.80140128296395	-3.75972674499461
C	-3.03365465293232	-3.41752316560140	-2.56815812016500
C	-2.13284130645083	-3.86908104301760	-1.56293779717660
C	-0.76012252114639	-3.75474524697822	-1.73423324220958
C	-3.38065023425137	-2.33432455872034	-4.76330445396522
C	-4.75489260352078	-2.43105777635341	-4.59807164893698
C	-5.29183126657608	-3.03986381423508	-3.43615681607951
C	-4.44814536013599	-3.53987522670180	-2.45309927484906
F	-6.61654428195430	-3.13070262156052	-3.30560238865320
F	-5.00352919180643	-4.13062017460586	-1.38925189662629
F	-2.92394122840775	-1.74742793696168	-5.87776427721417
F	-5.58627319430339	-1.94175009926712	-5.52134544214146
F	-0.54123000697020	-2.12136997867210	-4.96113617727174
F	0.05200747736206	-4.19753101426777	-0.76704748954257
F	-2.59673766101513	-4.40285321045920	-0.42306170971877
C	3.73607925195294	-0.09246834351413	-3.13928523583506
C	3.38600579078597	0.70696459284358	-2.04591698315094
C	3.79942645303931	2.06787483302055	-1.91941337707405
C	4.58619613825247	2.61873225483080	-2.92213355018342
C	4.94573431673694	1.86775766426076	-4.07615646400032
C	4.49998607129988	0.50055865070897	-4.20676558194686

C	4.79011996175368	-0.18982680329046	-5.42154450558105
C	5.52401782514149	0.41745018464645	-6.43087573169739
C	6.00324193773415	1.74611879494233	-6.27972159676964
C	5.71122197695774	2.45729258149732	-5.12625570389094
O	2.56282702646058	0.18234447550558	-1.05751946578372
C	3.35047184527695	2.86187338692762	-0.74757108124260
C	3.76675857925474	2.51782849691378	0.56342530058980
C	3.35550505290610	3.22114379194067	1.68519530778659
C	2.44434544762060	4.30683514726330	1.56082855321831
C	1.99992666601299	4.68508065699074	0.23437659268938
C	2.49425043643220	3.94920475167707	-0.88143325393906
C	1.07576684213122	5.76147430788132	0.11322888528873
C	0.54584332527847	6.38632031452906	1.23265229322354
C	0.97559656595068	6.00945022789342	2.52910533476933
C	1.91534802003471	4.99922864238298	2.68676026408446
F	4.60487188670283	1.48406037805678	0.71588153848445
F	3.83018245241831	2.85907306263495	2.88343527942713
F	2.08446050731212	4.29233919358901	-2.11525950200332
F	0.66790228611841	6.19246826929249	-1.09099204225627
F	-0.36755365186488	7.35501775123548	1.09823489574482
F	0.45079679025551	6.62418990994925	3.59070373003035
F	2.28192494266168	4.67048053259791	3.92738246042751
N	-0.64577792596712	0.75547489305640	1.61311608538325
S	0.31726296993432	1.38443979849125	2.81092616252765
O	1.70654480682136	0.89211893152160	2.74939415620471
C	-0.44303564379535	0.70888110878450	4.42908250734639
F	-0.07486278747640	1.54014183460633	5.40979885202540
O	0.02036605577226	2.82882924498605	2.87085692491023
F	-1.77798895757018	0.70531495906050	4.32462251233804
F	-0.01550210196605	-0.52149102870564	4.70913102472730
H	-1.06301726804471	2.88133411582062	-1.02841970496050

C	-1.69207636488686	3.37224043276878	-0.25667920669606
C	-2.22235659429757	4.69777125121184	-0.35605133339800
C	-2.23827768486454	5.58489964587872	-1.41245413996871
C	-1.70883016720280	5.41306367666076	-2.80609726476085
C	-1.24579146693387	4.05326788195910	-3.32126270122334
C	-2.36400632795100	3.04259729826815	-3.41141956086870
H	-3.33119418295833	3.33189813503020	-2.95979362852039
C	-2.90221429056155	6.91150584082109	-1.18032325072963
H	-3.08858795375323	-5.28767352413308	2.53897951510643
H	-3.06545824803077	-7.75661209637394	2.27839035154789
H	-0.90675497782796	-8.97419726347002	1.85818751741169
H	1.23113382452128	-7.68927859233583	1.66143361656004
H	2.44778630708761	-5.56445416235061	1.69658622337418
H	-1.34952934796409	-4.55778549794785	4.81463541016055
H	-5.42888958687987	-1.85608832632864	5.92889299485063
H	-5.27737706920595	-0.69630206360784	3.77483814483302
H	5.98381937439811	-1.52860449916322	-3.31108384626806
H	7.41739370974903	-3.53624493098913	-3.51839995002568
H	6.39673479205763	-5.83322709524931	-3.59474693765656
H	3.91368578456400	-6.09605861776969	-3.42603104936150
H	1.68352243313716	-5.12378304142491	-3.17314144883444
H	4.41543557275606	-1.21337352312535	-5.55866367312754
H	5.73123053719701	-0.13505951011840	-7.35988139754971
H	6.59063480050600	2.21265505392061	-7.08482340752623
H	6.05408939890001	3.49654760809016	-5.00500557264512
H	4.92137638861446	3.66335588354058	-2.83822470496412
H	-4.54908928897945	-3.54066201687018	7.55672291232594
H	-2.48793882201163	-4.86551843587944	6.99206956187807
H	-3.91702592116907	6.88547041400478	-1.63623380898823
H	-2.34835582814704	7.72852599717655	-1.68485432273954
H	-3.01529936354407	7.14550387136243	-0.10670184844377

H	-0.88192007793028	6.15258214328103	-2.89337461119640
H	-2.49472448326313	5.81924795600856	-3.48403383426229
H	-0.39649799026833	3.67235069963460	-2.71397630784701
H	-0.80547219419226	4.21989339029506	-4.32777190234673
C	-2.29583236068593	1.83366542063239	-4.01931160826547
O	-1.93961668173965	2.72866722894571	0.81172485501970
H	-2.68774059209204	5.03633770834456	0.58472931132395
H	-1.35363621771030	1.73846997822848	1.05144229289732
C	-3.48482030868020	0.91336855979538	-4.03646381787102
C	-1.05987465344220	1.31738455066506	-4.70169514049775
H	-4.40500493977051	1.41483745749237	-3.68895610129631
H	-3.66971113075291	0.50439499901425	-5.05138629096826
H	-3.30786458344113	0.03577451744997	-3.37728183982250
H	-0.75815975834627	0.33589836414774	-4.28578450365789
H	-1.26198720246675	1.14314777654989	-5.78147367111976
H	-0.19179905268643	1.99433190981796	-4.60671998734319

TS$_{AA}$(minor)

172

C	6.33416287577672	0.59147622087119	2.24466242698859
C	6.01421991338470	-0.51688093191443	1.47163149016330
C	4.84852436755547	-1.28609876220171	1.74665977797042
C	3.99198620711000	-0.89424438699299	2.84768855613168
C	4.34808795602378	0.25671198102924	3.61048371680315
C	5.49217459951621	0.98551025405742	3.31427734468602
C	2.84386326792188	-1.69344874619916	3.12101723245317
C	2.45452458663035	-2.77602683542693	2.33312522713601
C	3.31840920621530	-3.13122829094626	1.26046444882336
C	4.48558401019473	-2.43315055140461	0.98914889855661
C	1.20617668284554	-3.53634013336375	2.58305001302154

C	-0.05008769693009	-2.85646668412425	2.66782515815345
C	-1.26184368813691	-3.52831119479207	2.86116720940639
C	-1.25662032882350	-4.96655044525799	2.90936999236571
C	0.00397725386636	-5.66287406289305	2.83436969409996
C	1.20802070694165	-4.92351852551493	2.68477412477734
C	0.01846117561086	-7.08913507421293	2.87982829140934
C	-1.16233172492401	-7.80896809696700	2.97028521756897
C	-2.40702530873911	-7.12465382822781	3.00702960139954
C	-2.45382743328138	-5.73875324945168	2.97758473307126
O	-0.05184085767495	-1.47620637779778	2.56649173540443
P	-0.60159620457925	-0.74837800587096	1.17694524746643
N	0.42550569233378	-1.18598731585104	-0.00052943568589
P	0.84368645291796	-0.26121187312625	-1.20748551748524
N	-0.01203034166513	1.08496760039572	-1.62468907241126
S	-1.16050809346476	1.05979352459870	-2.81868902482511
C	-0.15124356136999	1.25326148747547	-4.44162295613492
C	-2.51685252330001	-2.73509685792202	2.96018126565419
C	-2.82812917741261	-1.82967949966162	1.94166479659830
C	-3.99302637935829	-1.00381196474125	1.97044202141927
C	-4.86089063123200	-1.12092689293722	3.04844754030662
C	-4.58242479316251	-1.99925299453307	4.13166952531852
C	-3.38306175213303	-2.80179204574144	4.10994132446608
C	-5.44578789063075	-2.06175176366515	5.26601038420653
C	-5.13065079260334	-2.85672317090469	6.35690999170771
C	-3.92767572336121	-3.61213789619716	6.35659214962862
C	-3.07525658170985	-3.58572712452323	5.26159031167964
F	3.01641494030771	-4.18066545911731	0.48532421307217
F	5.26647053135940	-2.86370413353976	-0.01140988782972
F	2.11022914437665	-1.40455690982978	4.20649998789621
O	-1.95137795925225	-1.68353675541291	0.87635560395319
C	-4.20722615313617	0.00499273394311	0.90400810469657

C	-4.35089471199763	-0.37574382531298	-0.45457984967905
C	-4.54831222103152	0.54872897182192	-1.46945620236959
C	-4.56445879926422	1.94309465381757	-1.18327315518902
C	-4.40224023538217	2.36838828613217	0.19308037509348
C	-4.25440255788302	1.36577455508447	1.19309761754466
C	-4.38397466756998	3.76559880829366	0.47856983228237
C	-4.49455598013680	4.70183166957072	-0.53917603679374
C	-4.65635834577302	4.28505894399275	-1.88390803427630
C	-4.70046711089638	2.93319545957325	-2.19713778448548
F	-4.32933741507565	-1.67868893430964	-0.76245924779815
F	-4.73177897918416	0.10154073611310	-2.71718636121869
F	-4.12927299585451	1.75424500408776	2.47361625080405
F	-4.84906029545840	2.58987062306446	-3.47772513478458
F	-4.24614718289618	4.22146932433198	1.73483678496026
F	-4.44155291068609	6.01232597711951	-0.26807064495448
F	-4.75914332617290	5.21100513654258	-2.83955325549321
F	6.82110313479419	-0.82938446617709	0.44888871168712
F	3.57860826389651	0.68670110805582	4.61858804326484
F	5.80522769054067	2.07701555175306	4.01634791878506
F	7.41786034814873	1.32117175732739	1.97022698275549
O	-1.91098677962360	2.32611124839496	-2.71982794530939
O	-1.86071829565241	-0.23700436443499	-2.91100204404554
O	0.95329528107977	-1.09609335964132	-2.61272879502598
C	1.92751823909310	-2.08614440413289	-2.70106875047357
C	3.25162420240120	-1.68986935140987	-2.87729212022602
C	4.27113170222829	-2.70532871859062	-2.88341450408081
C	3.87309674567113	-4.08778103244021	-2.76451488449859
C	2.49656220063553	-4.42366450441632	-2.63442643572221
C	1.50917975587588	-3.44556744641188	-2.59050368259163
C	4.87771505933508	-5.10074694423170	-2.75453094876916
C	6.22128651330639	-4.77135103575678	-2.84135269083746

C	6.61330284205224	-3.40907649009514	-2.93079887713935
C	5.66192901316952	-2.39987208164335	-2.95042201464265
C	3.55832034819383	-0.23869427767068	-3.01939207036211
C	3.14845870167113	0.66336649961153	-2.03280468884519
C	3.44825927429302	2.05903253526734	-2.08451417883658
C	4.15282837022625	2.53638346354419	-3.18590236319360
C	4.53185069194252	1.68387155778556	-4.25613728319651
C	4.21977719241882	0.27742353997262	-4.19030711823665
C	5.19694154964179	2.20153799579306	-5.40794578826968
C	5.51704365859325	1.37488084264653	-6.47308805272615
C	5.17224997386622	-0.00268842846497	-6.42792487333536
C	4.54043441799288	-0.53938025032023	-5.31499095651332
C	0.07318552602781	-3.75353667131753	-2.38676111154775
C	-0.91271477352103	-3.25854198992395	-3.24053967650052
C	-2.30655956833567	-3.45107654489655	-3.01831222686617
C	-2.71245652887372	-4.25645852919636	-1.88454474268059
C	-1.70003770244855	-4.79118905458149	-1.04046563797455
C	-0.35915931151446	-4.53429669756874	-1.28053557875355
C	-4.09990690137103	-4.48253201018190	-1.66324911386200
C	-5.05335017152302	-3.92524409533228	-2.50483599179869
C	-4.65983638476197	-3.11592981317389	-3.59896469753319
C	-3.31405380902972	-2.89112168582337	-3.85711807999477
F	-0.52265087876473	-2.58524600391281	-4.32946367679783
F	-2.02433070882083	-5.55112321626180	0.01595821825089
F	0.54891641898693	-5.03699101795599	-0.43251206947965
O	2.40754900877113	0.19735752617009	-0.94680133212907
C	3.02675578418486	2.98736613658195	-1.00791786785241
C	2.36961307103938	4.18564039904668	-1.29289211347364
C	2.04985680507414	5.15638314082603	-0.29938308586691
C	2.39326617328013	4.86835213256357	1.07883801706986
C	3.01890929963394	3.62268822614934	1.37106266078339

C	3.33941011198252	2.73340855673464	0.35554234288851
C	2.10297810169035	5.84397488677160	2.07429970333274
C	1.47718521391455	7.03790843189988	1.74006095949688
C	1.10350055816252	7.30482693509085	0.40097115476613
C	1.38101822351772	6.38006524655705	-0.59754695493157
F	2.02874959376004	4.43863437414789	-2.56319215751112
F	3.33828472612364	3.29802107004678	2.63390978134917
F	4.00633911593744	1.61895093804825	0.67338294099309
F	0.98676712793785	6.66962749205328	-1.84039271660202
F	0.44591257524870	8.43328672112929	0.12445866357213
F	1.16814541157164	7.92820373761959	2.68972848969622
F	2.39541368476107	5.62696980205335	3.36247484735700
F	-4.52353955440309	-5.21640240323812	-0.62503247590481
F	-2.99764504789973	-2.13361835771903	-4.90984440667562
F	-5.60222831560261	-2.57433885636267	-4.37472901440764
F	-6.35488696887272	-4.12152296844405	-2.28019007863976
O	-0.89619582775330	0.69691510894374	1.45369209295602
H	5.97888857028074	-1.34883479925181	-2.98862690098594
H	7.68214346772098	-3.15042313889684	-2.97201359176787
H	6.98664568742284	-5.56186667626923	-2.82734676626081
H	4.56152639342699	-6.15157840355913	-2.66570531237572
H	2.21575274791047	-5.48302544370476	-2.54476937141019
H	4.26961073318656	-1.60361351714703	-5.29926782584305
H	5.43738717268559	3.27548440924961	-5.43684127039333
H	4.40940251270110	3.60390026289692	-3.24522411238100
H	-3.42390048710949	-5.22377222695552	2.97688262316812
H	-3.34447804716655	-7.69969694661385	3.04790866974677
H	-1.14018269321489	-8.90882177317934	2.99796070258051
H	0.98934130421602	-7.60594820543603	2.82848370248850
H	2.15951709793502	-5.47089447817488	2.62201162657325
H	-2.14248649344704	-4.16536461726496	5.28089145534814

H	-3.66488457237566	-4.22023046716531	7.23544978780652
H	-5.80081054129637	-2.89217505669198	7.22896118756915
H	-6.36208640488998	-1.45129062781151	5.26209852991654
H	-5.76629080120926	-0.49729051178437	3.08753635504977
H	6.02391321030465	1.78445502429015	-7.35965569281303
H	5.40286638809915	-0.65087820448215	-7.28681130004049
F	0.06252887105979	0.07151726740278	-5.01537839067060
F	-0.86980262851451	2.02378008854080	-5.26447895758526
F	1.02205345565696	1.85057690957924	-4.18985482101318
H	0.58229275421710	0.39799280359343	4.16409092453442
C	-1.48913670218465	5.66313460170188	2.00244058779739
C	-2.15867793690925	6.99547425576164	1.82515119981459
H	-1.57727411861828	7.80137051986059	2.31886170883387
H	-3.14935396710320	6.96717883247907	2.32802432811684
H	-2.31762575078220	7.25188044893168	0.76322346191972
C	-1.06529723724856	5.38594248325197	3.41435134609119
C	-1.26439508826267	4.91386787255418	0.86651036133643
C	-0.41600583104580	4.06660068838562	3.82295494698638
H	-1.94434472356041	5.59160908161714	4.06742851434735
H	-0.37650853079664	6.22700342258802	3.65690123603562
C	-1.39956276354255	2.94903937894489	4.07723497057440
H	0.16939139514934	4.26517486568576	4.74618918588873
H	0.35525348381787	3.77349966494829	3.07619898386167
C	-0.76095458629296	3.58648790947178	0.69061271987331
H	-2.46432209966164	3.16939669152706	3.88709968678876
C	-1.06600573387573	1.72060995310452	4.53873837432569
O	-0.43292462767243	3.26437552573505	-0.49497242093454
H	-0.71105027770509	2.81632101902476	1.48942819972180
H	-1.49465514545396	5.39757112195858	-0.09830553482673
C	-2.10273387230607	0.67041804139659	4.82856151053359
C	0.35862136831501	1.29843133426020	4.76991735614103

H	-1.94069306038433	-0.21932580724879	4.18518523878146
H	-3.13202722103263	1.03456116874720	4.65755027745546
H	-2.02153913450994	0.31235539104857	5.87816435583953
H	1.10389140267158	2.07369188639492	4.51239348153375
H	0.52331606109816	1.00159015692739	5.82886435039718
H	-0.18940741235061	2.19612257788989	-0.82430695054960

A(major)

172

C	-4.42492921245024	3.35875656211324	1.55379468293607
C	-4.48146026198779	1.99897658063218	1.13176341860187
C	-4.85866198580898	1.70997690520719	-0.23885724034027
C	-5.18418631795802	2.79575155584378	-1.09967680132045
C	-5.11854814196478	4.10773159129442	-0.65456047657933
C	-4.76024962896774	4.38877635604364	0.68578811625602
C	-4.90000150445050	0.35058597537382	-0.65812049522870
C	-4.57456697867665	-0.66384350160513	0.22751716007622
C	-4.18818972782145	-0.41308604633134	1.56630444659428
C	-4.15412939412473	0.90956070342983	1.99140358520648
C	-3.78108166321903	-1.54319396357154	2.43519966201934
C	-2.60878192164163	-2.28062137804849	2.09436253144164
C	-2.13530337811163	-3.35091756095774	2.85318026065990
C	-2.84938907208847	-3.70184992448402	4.05652873945145
C	-4.04987843547493	-2.97640556384428	4.40017420297799
C	-4.48967367874309	-1.90673505645858	3.57107178150263
C	-2.38798007051628	-4.70553263170929	4.95953107206597
C	-3.09823813533847	-5.01149483582376	6.11210090102025
C	-4.30245487686103	-4.32687350183164	6.42485424425691
C	-4.76229654254986	-3.32331470381007	5.58658102330990
C	-0.85823082917322	-4.01153880216931	2.47054133662132

C	0.27996812945328	-3.21483454552667	2.32946855419418
C	1.58097788597191	-3.74273416002664	2.06895228182084
C	1.69624008477698	-5.11864906433098	1.89307614609194
C	0.56198889629198	-5.97499904262067	1.94593135801606
C	-0.74146035052325	-5.42361862003857	2.22779216542459
C	0.68655611148225	-7.37594024318230	1.70407124978547
C	-0.42786284358192	-8.20064485369035	1.71013415618382
C	-1.71583506621770	-7.65057727846746	1.95022015735295
C	-1.86939765454629	-6.29482805321661	2.20328726933113
O	0.14923921156373	-1.84078375731722	2.45824542866889
P	-0.53350192644240	-0.98938742753530	1.22114940037055
O	-1.90469386130156	-1.87413509992795	0.95995878457962
C	2.74639784006893	-2.83254740462873	1.98270581476341
C	3.01011166546322	-1.86998167025677	2.95841901326172
C	4.10409090613483	-0.96139449210088	2.88349405768782
C	5.05469261332707	-1.12140702430594	1.80403510245042
C	4.79014599303117	-2.11233784137296	0.81817229316784
C	3.66552714237321	-2.91930380645042	0.90039284371509
C	6.19946984405618	-0.27729500006131	1.76773043572993
C	6.37756088461870	0.71930972853781	2.71829975913057
C	5.41810821312938	0.91444123165418	3.74208091724147
C	4.30260594438548	0.09188850687800	3.82198692329491
F	2.22564817691984	-1.82631485014910	4.04214801572923
F	5.62179090662095	-2.27245300665330	-0.22307078649552
F	3.44884347610548	-3.81574067936833	-0.07484047661878
F	7.11774009811298	-0.39878979506231	0.79939260154557
F	7.44118984101411	1.52559362924223	2.66628939457218
F	5.59429172421208	1.91052831436623	4.61351660234616
F	3.41858283209840	0.32733237059552	4.79643157910057
F	-3.78233028520448	1.16478226128907	3.24994001620366
F	-5.25768625627144	0.02306943605654	-1.91239979741450

F	-4.63754623322506	-1.93591534190417	-0.18415440894808
F	-5.52128656499600	2.58687495529550	-2.38267209123857
F	-5.36684392803906	5.12085239821498	-1.49150941772218
F	-4.67541386536755	5.66543451164713	1.08185768554965
F	-4.04454240053353	3.68658482809212	2.79038649543702
N	0.34693687949710	-1.14942141456696	-0.09840730831994
P	0.83568036067362	-0.03918913123273	-1.13810760669345
O	0.15574645537377	1.31477347245503	-1.27860825062383
O	0.81498520659455	-0.72324913992084	-2.64996674218475
C	1.63228164390280	-1.83280267342245	-2.83609426214692
C	1.01605690654465	-3.11840192963844	-2.83285091257985
C	1.82751077600837	-4.23866396571087	-2.96061211060210
C	3.23884454978546	-4.11985534846170	-3.08640820434073
C	3.84894479989182	-2.81190185679165	-3.09969991419381
C	3.00642289144768	-1.64833567217107	-3.00415271708870
C	4.06733620527514	-5.27785311390661	-3.16913395345405
C	5.44637087597913	-5.16102087461173	-3.24539017815717
C	6.04943538588741	-3.87511564305222	-3.22865100834573
C	5.27080754330521	-2.72907903467064	-3.15916701924678
C	-0.45503132287509	-3.22824167984729	-2.68936026139794
C	-1.32206535803181	-2.64735622548669	-3.61054860982650
C	-2.73911256338060	-2.75125242859266	-3.51715305868342
C	-3.29613255965434	-3.50436371637525	-2.41090516011336
C	-2.40419751872416	-4.05468785124562	-1.44732855875123
C	-1.03071273838744	-3.93424147684434	-1.60146717673732
C	-3.63138857484255	-2.17343714354869	-4.46693672632690
C	-5.00655090860686	-2.29227897136735	-4.32532981134795
C	-5.54857361191293	-3.04130166343239	-3.25092283815367
C	-4.71044309749164	-3.64610709903360	-2.32476102802315
F	-6.87405275297850	-3.15348756010746	-3.14425466878677
F	-5.27133140126046	-4.34677779205766	-1.33355682363223

F	-3.16924369786220	-1.48055598871727	-5.51925087000156
F	-5.83513922506769	-1.72796594421646	-5.20669961882017
F	-0.79018095288441	-1.96699865971832	-4.63953241921706
F	-0.22383098153497	-4.47217094954415	-0.67927782131744
F	-2.87515454762424	-4.68956484471889	-0.36413977642641
C	3.53083928926656	-0.25668109650754	-3.04085651319812
C	3.21864323788285	0.61178204146660	-1.99035638143292
C	3.61944355649557	1.98416954517769	-1.98019952493166
C	4.37484035192224	2.45891422582386	-3.04609972579116
C	4.70772691893586	1.62912760386968	-4.15128759518226
C	4.25861919877392	0.25787021257684	-4.17274561317718
C	4.51237891586955	-0.51554881115250	-5.34496288758859
C	5.21757314187914	0.01872705365528	-6.41423430863773
C	5.70148804228850	1.35368121096940	-6.36985713086559
C	5.44303345032526	2.14379393920320	-5.26087536923570
O	2.46934399610756	0.13530761596776	-0.92086021624393
C	3.18018582064952	2.88789206711000	-0.88867391832050
C	3.50961100500136	2.62675464098767	0.46457786474867
C	3.05816215542532	3.42022488469559	1.50788954015675
C	2.22399004131048	4.54152157456680	1.25428592579275
C	1.90881879479130	4.86973279473737	-0.12196161598507
C	2.40960017893592	4.01817978087304	-1.14607171595452
C	1.09747205791781	6.01326081378068	-0.37684743913121
C	0.54946492240907	6.74890144523266	0.66295940921494
C	0.82006541232053	6.39792428741915	2.00959733287379
C	1.64293403259988	5.31713051882168	2.29766709615378
F	4.27769503659289	1.57012172582993	0.74285485032029
F	3.41468653637313	3.10445494617173	2.75748659019491
F	2.06947697621911	4.28468289824549	-2.42687642036841
F	0.81310683870901	6.39582951614934	-1.63386884800665
F	-0.24312321082698	7.79649587836566	0.40965729534549

F	0.26394056641201	7.11436478571528	2.98820825830923
F	1.86510763362323	5.01922304773279	3.57959960137572
N	-1.00075955469658	0.44865762992007	1.79609336410026
S	0.11844221991396	1.49506264121208	2.31792866413286
O	1.51239682565596	1.05375140481795	2.02410553714977
C	-0.02729380094645	1.39200100456028	4.19673490386391
F	0.88347704007250	2.21956303592843	4.73054994702749
O	-0.28561752012724	2.88721979036888	2.01571912918801
F	-1.25203663443761	1.77559061812184	4.58207529824157
F	0.20035523448398	0.14624485234363	4.62356647868962
H	-0.46924245634271	3.78546057213502	-1.87305514014953
C	-1.34560295973394	3.83434403176917	-1.20622346609400
C	-2.01507626078376	5.07029004464696	-0.91111876273831
C	-2.22697058857786	6.14630755191348	-1.73538180291632
C	-1.94047320269695	6.21164634009211	-3.20956652692754
C	-1.41906737190747	4.97769251043705	-3.95711123709816
C	-2.19281340699993	3.71223426699755	-3.69306294176472
H	-3.24787290701268	3.82302738719708	-3.38577781576821
C	-2.91276757591986	7.35906162891901	-1.17986059422909
H	-2.87107542465370	-5.87538238195401	2.37003195634369
H	-2.60101835355861	-8.30413531173556	1.92776752255739
H	-0.31993340939859	-9.27860563275970	1.51689919141231
H	1.68774016650138	-7.78610409770820	1.49958397826252
H	2.68276549286733	-5.56072437170593	1.69252486069833
H	-1.45095828547235	-5.23466767984268	4.74042480828840
H	-5.68138768687581	-2.76724921118809	5.82807498916754
H	-5.39994477340149	-1.35332068325873	3.84582059884706
H	5.75198393089625	-1.74265423777358	-3.11919772878788
H	7.14582524197023	-3.78611984093616	-3.26099850376796
H	6.07634528398960	-6.06140452106381	-3.30328813375020
H	3.58644116251995	-6.26823478023313	-3.16074330018516

H	1.37477088925429	-5.24133010894578	-2.95606016815846
H	4.13533127627663	-1.54553119720312	-5.39945175095202
H	5.39810372807063	-0.59755426085432	-7.30800444780988
H	6.26627495293158	1.76128619657713	-7.22174369155587
H	5.78898654413886	3.18831396186456	-5.22145630290808
H	4.70392022486021	3.50855791651423	-3.05164969526756
H	-4.85831300685707	-4.58346418315271	7.33918827523813
H	-2.71902433356608	-5.78794083852465	6.79364663150456
H	-3.92293214727950	7.46190235342534	-1.63082043544709
H	-2.34874695998525	8.27474444973320	-1.45288192048303
H	-3.02136858093718	7.31402894864014	-0.08222892869732
H	-1.24135089235290	7.06264604065182	-3.36782556574504
H	-2.89060371151788	6.55799034160538	-3.67794464477810
H	-0.34157404053815	4.82785053060740	-3.73487637767348
H	-1.44774479502080	5.21642794997709	-5.04412562190379
C	-1.71050753699094	2.44656036246968	-3.87373413620200
O	-1.61816311237072	2.79883536555663	-0.50509301273590
H	-2.41245732279280	5.11580771040071	0.11496695647701
H	-0.90383944789442	2.03512268526792	-0.74115741233073
C	-2.55434846229488	1.24428601955976	-3.57417397588629
C	-0.31099909577174	2.17925312567418	-4.36433946632594
H	-3.59864149528964	1.50520944455492	-3.32830087845057
H	-2.55180101215015	0.53239584772547	-4.42448035629101
H	-2.11254424071137	0.69253972629697	-2.71418433603413
H	-0.00300195657473	1.13666754891583	-4.16423655231465
H	-0.25454667781668	2.34770371738278	-5.46241705520596
H	0.44112979556242	2.85283008416308	-3.90514320458972

A(minor)

172

C	6.69406689528637	0.50987193532500	2.39929128430455
C	6.40440823034767	-0.59483461812727	1.60893996629094
C	5.18182963726583	-1.30575486682403	1.76901918838882
C	4.24363237858789	-0.86754538665184	2.78239208101471
C	4.57227167068071	0.27689027348626	3.56635212998638
C	5.76978888913100	0.95304783436549	3.37754020427639
C	3.04210840238628	-1.61366193785317	2.95541977723593
C	2.69283275253279	-2.70119032288846	2.15251563053667
C	3.62572051978410	-3.08767695562535	1.15096410492878
C	4.83654985252735	-2.43459461845905	0.97566176223369
C	1.43394769590146	-3.45807728590664	2.34377291658484
C	0.18458999594236	-2.76998689280610	2.41786588144037
C	-1.02693906166497	-3.41815756532134	2.66613293093058
C	-1.04151044090644	-4.85616723298774	2.70209410531991
C	0.20802928830633	-5.56960514185990	2.59930587631727
C	1.42167134774868	-4.84548265950548	2.44767560863589
C	0.20202991933946	-6.99608879018563	2.64095265384493
C	-0.98760053891546	-7.69862847657438	2.75323051758133
C	-2.22123677073873	-6.99661490865504	2.82019344231376
C	-2.24810147892679	-5.60957307904644	2.79722301918654
O	0.20357271290638	-1.39474147738666	2.28670690842431
P	-0.46388842161865	-0.63928406364337	0.97686903683272
N	0.37177196639154	-0.96677913452307	-0.33595008935239
P	1.05966441872397	-0.10451751653450	-1.48258177174946
N	0.60555706397233	1.36303381728728	-1.95808722720108
S	-0.90244997495696	1.89767459036914	-2.13589327063686
C	-1.43164794988949	1.21555490131078	-3.82010442778620
C	-2.24958919560013	-2.60157759031503	2.89624016962431
C	-2.64701169970561	-1.66026116028741	1.94561950734540
C	-3.80846386510706	-0.84159503467679	2.10097793367543
C	-4.55133531396236	-0.96561972267075	3.26836867451152

C	-4.16233477840673	-1.85980503285222	4.30303303768651
C	-2.99135813025747	-2.68560867580860	4.13046387401298
C	-4.89886270551521	-1.93292279942781	5.52304474203419
C	-4.48758248058270	-2.76447103988616	6.55287543079335
C	-3.31263135820768	-3.54780029341215	6.40090390186393
C	-2.58302763240162	-3.51028573785932	5.22062455924673
F	3.34106294392710	-4.12683093054285	0.35287700529172
F	5.67600144055792	-2.88722496906434	0.03475111957587
F	2.22740806362909	-1.27463471360725	3.96567751827275
O	-1.87472358853935	-1.48449995004047	0.80101956836857
C	-4.18734624863645	0.11592464208829	1.03514350639181
C	-4.39741843694314	-0.33599780541044	-0.29306837843047
C	-4.66828984601844	0.52741197843052	-1.34091662016379
C	-4.78994405370187	1.92607406699557	-1.11225223138369
C	-4.64986561954274	2.41481531667380	0.24526026522153
C	-4.33585378463497	1.47818494093311	1.27175629290502
C	-4.81269630472936	3.80955610609782	0.48316200752285
C	-4.98073662694065	4.70354874541896	-0.56433525053853
C	-5.07410719422257	4.22895451665189	-1.89637603470239
C	-5.00246690962527	2.86565057335810	-2.16028606693042
F	-4.31390074090852	-1.64904351418159	-0.53882412399224
F	-4.79456217113923	0.01803216729906	-2.57462147836517
F	-4.12488374165006	1.93971995392262	2.52210068526168
F	-5.14298821603223	2.46401553732762	-3.42657888294145
F	-4.80501272394122	4.30667936601558	1.73149812110840
F	-5.08346029883425	6.01619752192262	-0.32808357197297
F	-5.25412910011045	5.10595560515447	-2.88437473444135
F	7.29837747306866	-0.95980253722461	0.68150749682318
F	3.72903330720573	0.75012659201702	4.49247968910726
F	6.06036036102580	2.03819047048033	4.09837573244692
F	7.83436461317099	1.18324288427124	2.23315324754733

O	-0.89715065537784	3.36384147679817	-2.31039037580798
O	-1.86964976002676	1.27251208542903	-1.19219304087969
O	1.15261294712372	-1.03397536487979	-2.84661019283339
C	1.98762326024023	-2.14442937793184	-2.82686115872328
C	3.36702663015752	-1.95781552412784	-2.94629862442889
C	4.22313584581752	-3.11380321113732	-2.87762159773964
C	3.62371897000893	-4.41912835730943	-2.73261677774856
C	2.20947559033040	-4.54202507004680	-2.63984998051105
C	1.38169770833200	-3.42774253410106	-2.68351881072324
C	4.46463342287433	-5.56884944996513	-2.65910812250754
C	5.84407496220703	-5.44639025234571	-2.71700529463330
C	6.43563464720107	-4.16056017632084	-2.83309926921630
C	5.64600627654475	-3.02253935541371	-2.90736419830123
C	3.88682516752684	-0.56882195606672	-3.08177563591070
C	3.51654494115958	0.38392748719527	-2.13022737162603
C	3.93244540629663	1.74786578924406	-2.18692139631139
C	4.75274895918831	2.14409354951663	-3.23475871348906
C	5.14070183492676	1.23073003220887	-4.25473284822845
C	4.68769529681642	-0.13934060909050	-4.20034030427518
C	5.94165131553814	1.65867882483892	-5.35544164987831
C	6.26150737498265	0.78640852138327	-6.38419534805032
C	5.77678589337432	-0.54837966492363	-6.35582312463028
C	5.00803079845559	-0.99987759367705	-5.29214313367215
C	-0.09451667874845	-3.52838878015866	-2.57650079752818
C	-0.92616343184981	-3.07810133316240	-3.59800593173188
C	-2.34734585608255	-3.12765362316586	-3.52369116267226
C	-2.94749369346195	-3.70270718296718	-2.33699957217260
C	-2.09129571173866	-4.13846256092655	-1.28677633038984
C	-0.71233689111646	-4.05941648415820	-1.41458063715494
C	-4.36620014510568	-3.78923110735675	-2.26226736373177
C	-5.16651492543385	-3.28136982057125	-3.27574634633552

C	-4.58179085177827	-2.69871171811380	-4.42753129604661
C	-3.20089616319570	-2.62826306626714	-4.55086017861887
F	-0.35321700521166	-2.57739216082222	-4.70176290373919
F	-2.60359425235788	-4.61800353770944	-0.14312281356840
F	0.05811946658976	-4.46979836035461	-0.39755548743279
O	2.66383518711654	0.01282009571440	-1.09113490293567
C	3.43515324464796	2.69766316408599	-1.16268705354072
C	2.64399233086976	3.79286024787800	-1.49920856005165
C	2.09651379343454	4.67761137203365	-0.52525928262957
C	2.39300888714544	4.43177349903498	0.87309018005960
C	3.20346358952802	3.31121209960367	1.20631192272465
C	3.69775031408737	2.48000097348256	0.21299647529582
C	1.86588432630354	5.32395452912832	1.84880293264517
C	1.04090846683254	6.37668343342744	1.48212492151925
C	0.73240654821891	6.60469238635033	0.11945889031619
C	1.24515673222730	5.77032172411549	-0.86468511974405
F	2.36982074451890	4.00549830899722	-2.78996205893871
F	3.50462357532822	3.03824424264836	2.48771501041573
F	4.46810619912665	1.44156795109691	0.56322390787950
F	0.90993491789767	6.02160233030145	-2.13026576275622
F	-0.10793075436400	7.59795950383535	-0.19513284941885
F	0.49421826067572	7.16595801188397	2.41401058308575
F	2.11248785203081	5.15009754668334	3.15748273444552
F	-4.96737334541128	-4.32254251991192	-1.19196977604891
F	-2.69783374321692	-2.07587778238967	-5.66019331407935
F	-5.37849031770859	-2.21822149332193	-5.38531588497771
F	-6.49731558794396	-3.33246530964396	-3.18125384981983
O	-0.68574430808712	0.77309566320691	1.51203295337121
H	6.11926464630561	-2.03303679694121	-2.96315275167489
H	7.53172072671522	-4.06355734486907	-2.85000358217090
H	6.48268948584199	-6.34067514174284	-2.65889481212538

H	3.99217307967350	-6.55725341344967	-2.54938370883954
H	1.76758676068544	-5.54294330294893	-2.52493665758618
H	4.62872600861293	-2.03064900846827	-5.28947968231849
H	6.28836246387049	2.70344317111979	-5.37635722280167
H	5.09104099695787	3.18894517785370	-3.29678719275608
H	-3.20891184213607	-5.07764919940677	2.83030611091952
H	-3.16554804265980	-7.55833635456070	2.88264269391221
H	-0.98100830326053	-8.79874367170624	2.77763052816910
H	1.16391643522672	-7.52715909755567	2.56984489349981
H	2.36761303626589	-5.40347044816976	2.39659092147563
H	-1.66996798520997	-4.11265238175577	5.12416075717068
H	-2.97248753506436	-4.18747789593392	7.22930230123776
H	-5.05978886763261	-2.80949924045175	7.49165336349768
H	-5.79556333815774	-1.30344519257640	5.63204851354730
H	-5.45249133644384	-0.35034638873681	3.40611248749282
H	6.87563868024960	1.12930176948001	-7.23040275344372
H	6.00668782748596	-1.23036154298279	-7.18837332037732
F	-1.67722761346306	-0.10212978820511	-3.70417986351510
F	-2.55496217279136	1.83402759090267	-4.20803370056272
F	-0.48088563682864	1.40625552223760	-4.73830672698273
H	0.84258968303552	1.64937828092601	3.32638675487646
C	-2.35326091086850	5.76425702750944	2.30536336381751
C	-2.81763902917199	7.13255357770502	1.92869637421914
H	-2.25951222899952	7.89244212757905	2.51690072344239
H	-3.88630199048840	7.24690933320755	2.21213817199732
H	-2.70033610677172	7.34590641119731	0.85152734612103
C	-2.56100051610602	5.37995920966511	3.74919780484272
C	-1.77594976088815	4.97242139019316	1.33447495408384
C	-1.43129041984572	4.62386115012691	4.51125365284179
H	-3.49397972929124	4.77234152518155	3.78340945335442
H	-2.81144466101012	6.31061037328607	4.29503998727947

C	-1.68096239901749	3.15115822007225	4.70714409881989
H	-1.37155427413895	5.09649464917680	5.51677511179295
H	-0.44932360143428	4.82582008881680	4.03743965984722
C	-1.37748153313542	3.62616221701025	1.52887914016829
H	-2.72114281746085	2.90099772435590	4.98952263478628
C	-0.80562692733410	2.11438825908868	4.62776932191521
O	-0.64571486502255	3.02723551957447	0.67881426141757
H	-1.71346919651319	3.05338556488931	2.42104123418411
H	-1.61324189026960	5.37903700902099	0.32430123065938
C	-1.26220017952282	0.70728332243328	4.91514143474118
C	0.64366434966196	2.24310670523085	4.24386594919400
H	-1.03730251561928	0.04533152155411	4.05431282650099
H	-2.34706722654292	0.65014617461480	5.12967092367132
H	-0.70963177119386	0.28447655670348	5.78256105734522
H	0.97032213919283	3.28271809477425	4.06692176715614
H	1.29732987341843	1.80860498598730	5.02922206065412
H	-0.59109101320339	1.95066754863443	0.95952784627356

TS$_{AB}$ (major)

172

C	-4.49137030636358	3.36665777088294	1.48777099169616
C	-4.52729557317620	2.00757866462313	1.06146116401479
C	-4.86592364038765	1.72052483955320	-0.31861980180901
C	-5.16398905257488	2.80709559471204	-1.18636465432983
C	-5.12384357031141	4.11851485169171	-0.73661699644268
C	-4.80528464200532	4.39865840802427	0.61398913576619
C	-4.87871427920002	0.36338124582229	-0.74372049078091
C	-4.56223796685321	-0.65203305724943	0.14373237823490
C	-4.20888316190343	-0.40347154319759	1.49184004257660
C	-4.20486133416429	0.91832472748077	1.92286001493543

C	-3.80944584319534	-1.53051146274791	2.36814580663741
C	-2.63388589288803	-2.27314229228550	2.04710418562511
C	-2.17033453954737	-3.33507193889624	2.82508260559446
C	-2.89644086890115	-3.67093361966938	4.02532177508979
C	-4.10087134645851	-2.94237863412550	4.34680768530585
C	-4.53042609306145	-1.88268204613926	3.50032360620278
C	-2.44286610951906	-4.66104044146091	4.94724459646037
C	-3.16509700853778	-4.95266762620310	6.09604797478144
C	-4.37409471923574	-4.26640614431430	6.38615982601767
C	-4.82615188057390	-3.27481941869630	5.52965360264886
C	-0.88857661440005	-4.00000694619038	2.46602965963709
C	0.24908790518622	-3.20184459174152	2.32779973226097
C	1.55193905039099	-3.72880284312354	2.07635283950125
C	1.67249197865173	-5.10629459927490	1.91800533601639
C	0.54139781630890	-5.96604774468449	1.97892258519027
C	-0.76567341633453	-5.41521209284234	2.24464628834309
C	0.67276371719800	-7.37013559777067	1.75992693714880
C	-0.43811673442493	-8.19939857984164	1.77440216883585
C	-1.72943369939489	-7.65085447062780	1.99952606374638
C	-1.88967918185352	-6.29182356586663	2.22927964796270
O	0.11285294264744	-1.82722377551033	2.44679379661264
P	-0.54273970471075	-0.99640709356762	1.18003269227470
O	-1.91533763193075	-1.88249714446655	0.91629212285007
C	2.71531439449653	-2.81740681949894	1.97866217154277
C	2.98238417217993	-1.84729025543938	2.94555298832160
C	4.08152184205805	-0.94548738942796	2.86315708795889
C	5.03035841071987	-1.11836057783907	1.78396632435847
C	4.75704432100289	-2.11133511056908	0.80257015643492
C	3.62894770676968	-2.91232807496450	0.89249554589148
C	6.18165894711451	-0.28350150795485	1.74300601280272
C	6.36771983941954	0.71681523978663	2.68813042286845

C	5.40983628280040	0.92516652722499	3.71067526440779
C	4.28680728776540	0.11327989486625	3.79385525536234
F	2.19752448519314	-1.79036925114432	4.02857449765878
F	5.58203510497305	-2.27796106670553	-0.24302033025327
F	3.40369795892256	-3.81082036084978	-0.07905621224743
F	7.09892915451395	-0.41746134535035	0.77525208778364
F	7.43765943728178	1.51451224368727	2.63185932889650
F	5.59337836173249	1.92513665100856	4.57608208759478
F	3.40214766792863	0.36403216110996	4.76397826562259
F	-3.86225592620226	1.17283331727265	3.19022503226018
F	-5.20112885879364	0.03751868768578	-2.01008906473230
F	-4.60320207817815	-1.92296715779604	-0.27619445815391
F	-5.43793629664972	2.59983060171826	-2.48763966529106
F	-5.35416552242687	5.13080913767235	-1.57923476766349
F	-4.75089612418082	5.67191534455993	1.01893344874416
F	-4.14971730404043	3.69220320409005	2.73676989782829
N	0.36887744539785	-1.18676242451121	-0.11545587650661
P	0.84200550534690	-0.05964697589238	-1.15208202241923
O	0.14377594360343	1.28118172589489	-1.27008253519866
O	0.81765083683976	-0.74715687587249	-2.66876260991689
C	1.64070267894967	-1.84962444913953	-2.85902855874530
C	1.03691322708391	-3.14173021240213	-2.84752189420713
C	1.85873576860331	-4.25461875481011	-2.97758471290571
C	3.26799340239906	-4.12296683390690	-3.11232772042717
C	3.86555665086021	-2.80938323239622	-3.13292829001864
C	3.01240798339079	-1.65420360276273	-3.03578501914700
C	4.10715599140742	-5.27325027080861	-3.19656465828268
C	5.48451612633367	-5.14334527481576	-3.28148024151528
C	6.07532982844963	-3.85156400712645	-3.27246693494992
C	5.28617923665096	-2.71279951441912	-3.20131799109459
C	-0.43086186911035	-3.26752658113499	-2.68852396853505

C	-1.31774978886383	-2.66708495940377	-3.57786502175132
C	-2.73257793238807	-2.78177861312858	-3.46656627412168
C	-3.26688912619480	-3.58875749142134	-2.38815203437999
C	-2.35694300613798	-4.16001756638336	-1.45465152414277
C	-0.98684564928468	-4.01512962914046	-1.61730103768342
C	-3.64453155299675	-2.16441426803233	-4.37177136878020
C	-5.01583913597415	-2.31818963859726	-4.22694460461402
C	-5.53468879359313	-3.12500121579408	-3.18366725429542
C	-4.67757324772334	-3.75503096879680	-2.29238655988001
F	-6.85708571157072	-3.25970743418413	-3.06664049590735
F	-5.21697928724250	-4.49462324835025	-1.31789702687417
F	-3.20410663344385	-1.39420422245663	-5.37947475968794
F	-5.86246207751576	-1.71743624377372	-5.06615834445015
F	-0.80917902623249	-1.95296070402078	-4.59830872888253
F	-0.16577248885801	-4.57192371395361	-0.71922021588226
F	-2.80809714282514	-4.84059196807481	-0.39159386219567
C	3.52269344649561	-0.25732934708216	-3.07882303087538
C	3.21132726774426	0.60750093363071	-2.02441373181760
C	3.59765603370956	1.98529378480631	-2.02000523549892
C	4.33494478107987	2.46613815203783	-3.09652169555614
C	4.66473909933317	1.63946337817737	-4.20482120008461
C	4.23282629989940	0.26267540005599	-4.21886761212642
C	4.48581692521265	-0.51086266282591	-5.39131681659391
C	5.17330122827104	0.02978556251196	-6.46890871741532
C	5.63957652423441	1.37136631805024	-6.43283264258779
C	5.38196042198793	2.16088949553531	-5.32317366719793
O	2.48024408604164	0.11865077158392	-0.94980624149291
C	3.17061791065385	2.88910390237911	-0.92335083811027
C	3.50788675152626	2.62119540167226	0.42666470191176
C	3.07399261718279	3.41764728328576	1.47471412623437
C	2.25591569465561	4.55250693412822	1.23169098332698

C	1.93317391769717	4.88914161466876	-0.14081959991344
C	2.41530158949269	4.03285293765317	-1.16974336463431
C	1.14245055478539	6.04944734752940	-0.38693865331104
C	0.63628386430412	6.80456665949575	0.66009256641760
C	0.91298194747667	6.44520416971038	2.00342657515105
C	1.70630693582070	5.34067887378192	2.28241865798536
F	4.26829169695213	1.55706012034731	0.69773882475089
F	3.43506464985262	3.09223463991631	2.72096529239465
F	2.07366081071250	4.31192956468895	-2.44926147090873
F	0.84812753062876	6.43634555821808	-1.63969576589596
F	-0.11737558156422	7.88284773639319	0.42069296010333
F	0.39525385286685	7.18205809266503	2.98816738490236
F	1.93657229506574	5.03715042928443	3.56239896591886
N	-1.02398289206415	0.45225093029609	1.71654219060038
S	0.08924564624280	1.52963015144697	2.18767304048575
O	1.48666568450742	1.07752246540015	1.92789186315176
C	-0.06449171029145	1.50620571714780	4.06749703530268
F	0.82856297347786	2.37113614892861	4.57158486971774
O	-0.31584535731379	2.90462884448868	1.81696696027394
F	-1.29772368959259	1.88121012214403	4.43368120785385
F	0.18605873642911	0.28240236079155	4.54403827963643
H	-0.36262205582862	3.68319351838307	-1.60752315564671
C	-1.44700356609121	3.70140177501637	-1.38034529608867
C	-2.00857875118615	4.99368172942555	-0.90612369280119
C	-2.26337215139232	6.11756137756650	-1.62085115527222
C	-2.07540928705571	6.18344088546593	-3.11281367562196
C	-1.40375014008290	4.95875253744813	-3.73021186887917
C	-2.02261975650954	3.66381617496940	-3.23031582371421
H	-3.11084065682370	3.70207549984830	-3.04670606961167
C	-2.83417074446517	7.34482301096482	-0.97306907719015
H	-2.89399737127777	-5.87407141979448	2.38367279298471

H	-2.61180597238339	-8.30841507400021	1.98445154871923
H	-0.32480159709113	-9.27987442360630	1.59910273544335
H	1.67667823499838	-7.77937922075186	1.56730572319323
H	2.66115951029962	-5.54716189328576	1.72520780874484
H	-1.50219683649422	-5.19089021518553	4.74634819850487
H	-5.74833574652705	-2.71660533188350	5.75388173690991
H	-5.44285063409214	-1.32533370460354	3.75963801851188
H	5.75780003776348	-1.72142928127773	-3.16761673532934
H	7.17062695953099	-3.75234882744052	-3.31172163631579
H	6.12278861799135	-6.03777300632675	-3.34070106041058
H	3.63594451237831	-6.26822162539784	-3.18182308129564
H	1.41662984468017	-5.26190377107874	-2.96668380805007
H	4.12292644683485	-1.54634045968566	-5.43865077970538
H	5.35444026487187	-0.58682853044359	-7.36235203043700
H	6.19053538503710	1.78410261959528	-7.29129388687595
H	5.71566275335764	3.20961473676701	-5.28935038365816
H	4.65366529624997	3.51890446708813	-3.10676774813499
H	-4.93960420284046	-4.51164950230630	7.29769354462151
H	-2.79178939993199	-5.71872537877985	6.79245572657288
H	-3.85211848108518	7.54417594026834	-1.37136580506708
H	-2.21412916119672	8.23349127838375	-1.21025163851351
H	-2.90355271152561	7.24203327115031	0.12453459571901
H	-1.49190895039460	7.09674691060062	-3.35844023458027
H	-3.07931962172857	6.35231413864109	-3.56687483355988
H	-0.31829584287245	4.97307909049103	-3.50219982455973
H	-1.48727086425419	4.99987134958738	-4.83675798000352
C	-1.54997197061282	2.40558833913313	-3.64921756612147
O	-1.87502842925518	2.65702614071145	-0.67848986038387
H	-2.22438903112549	4.97337032401785	0.17517365405580
H	-1.13562808458449	1.95359129905905	-0.73288523620673
C	-2.36074443315030	1.19815454888857	-3.38235226078058

C	-0.19872745465907	2.25052325916742	-4.24823391294018
H	-3.44839854069433	1.38849274200738	-3.38369077262874
H	-2.09951494559349	0.36007849215615	-4.05357875739759
H	-2.09285902423956	0.86688845551269	-2.34676639453085
H	0.14996146445082	1.20259116363120	-4.23345552897941
H	-0.24807306684650	2.59487061297663	-5.30811991946009
H	0.54986552059599	2.90295208925835	-3.75592115193148

TS_{AB}(minor)

172

C	6.67159965847798	0.55632121438388	2.48710141047608
C	6.36268661581622	-0.51488601045638	1.65901682591221
C	5.13204493355002	-1.21548813041140	1.80145735959589
C	4.21774208276255	-0.81785395506151	2.85229182692077
C	4.55585237199543	0.30577394349304	3.66127165089535
C	5.75680346618377	0.97867165425428	3.48364327984268
C	3.03066440668774	-1.58410340076977	3.03434149441291
C	2.65760921774646	-2.63277668535476	2.19379880848149
C	3.54560709877088	-2.95001260390885	1.12857311021487
C	4.75041934495263	-2.28759053596543	0.94891476686792
C	1.40609587835062	-3.40199633394953	2.38472495225850
C	0.15725864944848	-2.72204392629776	2.51299722203683
C	-1.05996888247055	-3.37963728181326	2.68792836622295
C	-1.07288411077829	-4.82052708193927	2.66332940305824
C	0.18055613421995	-5.52372642080982	2.53282110877260
C	1.39313608078433	-4.79254046471429	2.40828520414697
C	0.18151004325468	-6.95016605125504	2.50263564387147
C	-1.00369179420970	-7.66382847351132	2.58333346054655
C	-2.23991358592242	-6.97199904164659	2.68440075336753
C	-2.27457878026481	-5.58576275404064	2.72262306105392

O	0.16267738873926	-1.33903730363906	2.46610559758933
P	-0.33732481445572	-0.60169831676495	1.07025955742454
N	0.57320768824320	-1.12183574507701	-0.14384125463170
P	1.21560936060378	-0.24067008874587	-1.30645925147599
N	0.74620626915110	1.24406739893351	-1.70206033486379
S	-0.75899587469453	1.79223262710194	-1.77851801882950
C	-1.09991935297398	1.75936920263316	-3.63284469310105
C	-2.30332389090880	-2.57826197346408	2.85492113691217
C	-2.65986147808527	-1.62072879212393	1.89602819190466
C	-3.91593733569499	-0.92845597808959	1.94123517146082
C	-4.76549139790736	-1.16709084428001	3.01611336832499
C	-4.40124273606880	-2.03777511290809	4.07765632616155
C	-3.14785088224074	-2.74743985354918	4.01084098190385
C	-5.24715378085444	-2.21057229626275	5.21421849333640
C	-4.86401041133879	-3.02715445403448	6.26661395089537
C	-3.61061995480014	-3.69486439483540	6.22222861524309
C	-2.77444784964346	-3.56082425491281	5.12266814788387
F	3.21814197707125	-3.92111774134541	0.26538158736381
F	5.54253373047535	-2.66510208480576	-0.06356929955269
F	2.25118720273545	-1.30106558597684	4.09200003441464
O	-1.80677090198557	-1.34747540779510	0.83938155473580
C	-4.28963656835855	0.04886171342874	0.88866606804302
C	-4.31252832019358	-0.31460903028216	-0.48232987530704
C	-4.59579260081748	0.59484218324935	-1.48758840240182
C	-4.85720211548839	1.95836303530887	-1.18144687955650
C	-4.87942173659788	2.35651753013267	0.21075824975378
C	-4.62644361539602	1.36443746046320	1.19796832852917
C	-5.07171837267039	3.73209964112879	0.52771883719408
C	-5.21283412450313	4.68173832099766	-0.47421424563914
C	-5.20662876303723	4.29077450961452	-1.83690370569050
C	-5.04542446634052	2.95453420563282	-2.18229068205263

F	-4.02778895710141	-1.57952062819611	-0.81645287287331
F	-4.55993915207542	0.17587102456157	-2.76105196924998
F	-4.64711639317174	1.73055676407365	2.49452993505823
F	-5.06365216170467	2.63438242472454	-3.48009695254009
F	-5.08084700890007	4.15913719403032	1.79832284619702
F	-5.34477030279013	5.97922475010281	-0.17084057280742
F	-5.36307788222889	5.22366307705572	-2.77803280574522
F	7.24212983141473	-0.85176936093474	0.70785440283402
F	3.71554259209406	0.76199974940006	4.60132335479352
F	6.05922499554917	2.04259830183867	4.23125412218360
F	7.81776239093388	1.22334628573757	2.33597263679029
O	-0.80352134616102	3.22453020961864	-1.40695222097200
O	-1.77765328016224	0.86484628581558	-1.21409146833559
O	1.25034301651576	-1.13756762462101	-2.69419355310420
C	2.09226542122231	-2.23712229149837	-2.76803668068753
C	3.46524644987541	-2.02994862566337	-2.93762047770578
C	4.33876354078793	-3.17310457834999	-2.95358592871095
C	3.76117207709832	-4.49256691669654	-2.87534455100131
C	2.35341531297368	-4.63876747706225	-2.75099534434748
C	1.50687700254069	-3.53678731185428	-2.67870874157860
C	4.61880794647754	-5.63311926409486	-2.89711817670036
C	5.99490757699949	-5.48611312322633	-2.97008394868648
C	6.56634025057950	-4.18571121904403	-3.01110911980382
C	5.75937924056899	-3.05751633866392	-3.00319634526495
C	3.95991507100715	-0.63218029455380	-3.06790465387488
C	3.61187876108591	0.30221754161117	-2.09006678876523
C	3.95681730002597	1.68517707025039	-2.17850240224913
C	4.71234622110204	2.10802429618161	-3.26420737319357
C	5.09665790117058	1.20716619184681	-4.29571092976821
C	4.69277359049311	-0.17690367570048	-4.22294138936113
C	5.83424665630777	1.66318187322112	-5.42896516947253

C	6.13790051328534	0.80337516401932	-6.47283537760496
C	5.69710313770932	-0.54601041948356	-6.42694701123005
C	4.99056278025972	-1.02373639792331	-5.33201297928432
C	0.04796526126412	-3.69537281086169	-2.47466587029047
C	-0.89984888137971	-3.07519179859952	-3.28909947283854
C	-2.30562639310424	-3.21562524290213	-3.09723456714503
C	-2.76954061246277	-4.10993220140778	-2.05611701388980
C	-1.79816067337116	-4.75089792250712	-1.23833310024306
C	-0.44364881301274	-4.52806634274010	-1.43142924597971
C	-4.16859938926603	-4.31508808947981	-1.89783945899917
C	-5.08060765133300	-3.65382957503887	-2.70977408587975
C	-4.63121369268251	-2.75359180673753	-3.70650735520740
C	-3.27266899140823	-2.53445346319707	-3.89282543034825
F	-0.47038080921656	-2.34861653622501	-4.32876533411693
F	-2.17556341369705	-5.57999587868506	-0.25410724711609
F	0.42409688316428	-5.13587303382159	-0.60845985282123
O	2.84108032686613	-0.11203030269499	-1.00389768512690
C	3.44303962681078	2.64168000009454	-1.16812927515134
C	2.57055292126958	3.66989046352046	-1.51868025993916
C	2.02235362415734	4.57684003264128	-0.56677915766545
C	2.40702978719210	4.42729041682759	0.82265720269554
C	3.26857178923488	3.35252071798486	1.17554034895154
C	3.76673784259860	2.50013872730218	0.20282449898898
C	1.93107514064329	5.38019788343415	1.76610832575532
C	1.07073072988751	6.39815197582119	1.38311195178372
C	0.63150868191328	6.49956997104367	0.04036362055971
C	1.10463777002662	5.61191241997172	-0.91711615737800
F	2.21749999041609	3.79370744976978	-2.80193372726626
F	3.62167360857010	3.14931152204083	2.45904613840516
F	4.59740118006425	1.51574578367340	0.57030106283209
F	0.68135640664242	5.76454524869383	-2.17135568838323

F	-0.24219050227243	7.45779366043318	-0.28253235043018
F	0.63498600017303	7.29196899623477	2.27800190160673
F	2.29132721876331	5.30805712220328	3.06082798103549
F	-4.64413599462719	-5.13235152812536	-0.94839718932726
F	-2.90405866357657	-1.66139794353467	-4.83491130223331
F	-5.53315568192189	-2.10297237905632	-4.44446130091920
F	-6.39316537085950	-3.83532442664176	-2.54490862303637
O	-0.38604233724883	0.87333080045870	1.42776228628386
H	6.21562893315814	-2.05828835617411	-3.00838922866830
H	7.66056609073062	-4.07149882418075	-3.04000626744388
H	6.64725771354990	-6.37225523133759	-2.98162812275799
H	4.16189578943391	-6.63345248759550	-2.84364655566551
H	1.93177078303186	-5.65242028951507	-2.68959293658137
H	4.64508111704862	-2.06593584941596	-5.31814769781523
H	6.14382777754104	2.71924483973092	-5.46225660631975
H	4.99551197673429	3.16766987827571	-3.34884731619519
H	-3.24066204498586	-5.06605558014361	2.77112898890075
H	-3.18171268502101	-7.54022320887581	2.71906671729728
H	-0.99134261773132	-8.76377693070584	2.55417699753569
H	1.14615756247083	-7.47153529500459	2.40351497075805
H	2.33696626444714	-5.34790909616001	2.30952147209733
H	-1.80406092687394	-4.07536682513885	5.10759773078192
H	-3.29616828101153	-4.32162729094983	7.07046270650655
H	-5.52065129155183	-3.15012696458973	7.14098561645546
H	-6.20632575865224	-1.67063016509142	5.24070200559795
H	-5.73572983694882	-0.65157446727517	3.06091528693273
H	6.70304788240301	1.16710111520492	-7.34412269780120
H	5.91126407887159	-1.21912396133463	-7.27086990103340
F	-1.06028377963568	0.49561793584075	-4.07609599040825
F	-2.32173469255677	2.26745150083421	-3.86046872477911
F	-0.19075307749442	2.48923910613484	-4.29016699972744

H	0.82309334733427	1.04675735315170	4.10794453173542
C	-2.12652743542728	6.05625953515258	2.54022180989835
C	-2.76735755271440	7.32321331978154	2.05183323267565
H	-2.00340547044878	8.12421797872971	1.96016687023627
H	-3.52601084029884	7.68699772751393	2.77685628871833
H	-3.25526815773732	7.18498688741384	1.06908670827103
C	-1.66216281907507	6.04122676967539	3.97201577669144
C	-2.02693515870576	4.97455749505924	1.73121078106423
C	-0.85876139008038	4.80502698617173	4.36796297435297
H	-2.56624750580057	6.14101878093373	4.61662026336205
H	-1.06600112229421	6.95668215705449	4.16962763094045
C	-1.54938884819490	3.51716549066622	3.95513114247488
H	-0.70885613379278	4.79234924977682	5.46853293461055
H	0.15775025008910	4.85647889792483	3.93015739273493
C	-1.42785057033438	3.64098084196731	1.99639784317272
H	-2.65315664805235	3.53454291769618	4.01747929883006
C	-0.97189511565843	2.25293086918530	4.16699544664395
O	-2.08914392821873	2.66865956028247	1.39341864181066
H	-0.31984959261530	3.56300820906205	1.94900168728152
H	-2.46492236173477	5.01532760794123	0.71868476893943
C	-1.85697808910098	1.06376608115927	4.15080848025563
C	0.49733761836850	2.08033802850404	4.32081859551896
H	-2.39171608891170	1.05298180474802	3.17291080010309
H	-2.66378972114872	1.15235154349063	4.90793866427241
H	-1.31610403238280	0.10904103152158	4.26697768229614
H	1.08053977184259	2.79239023462571	3.70231834929890
H	0.77305015337640	2.31457648163424	5.37625553930318
H	-1.45732864855957	1.85412481026938	1.28546009180776

B(major)

C	-4.53886348206045	3.38459948799787	1.45293825315920

C	-4.55846862040951	2.02942289593625	1.01362744057497
C	-4.86097582383949	1.75462671735250	-0.37665713780738
C	-5.13707110653326	2.84726603919859	-1.24273658031549
C	-5.11515175456270	4.15443435671759	-0.77995101112608
C	-4.82974014044821	4.42401067668878	0.58017564864211
C	-4.83631049079995	0.40412988458077	-0.81938165671176
C	-4.52280906213145	-0.61860738085654	0.06094507935703
C	-4.21291186913121	-0.38328809603371	1.42242213406294
C	-4.24332624930494	0.93381667308004	1.86908119159140
C	-3.82746728245457	-1.51192084269297	2.30313507829268
C	-2.64627841571207	-2.25640919922860	2.00641690306431
C	-2.19684085008830	-3.31558169886491	2.79807940147042
C	-2.94042697635120	-3.64039868583360	3.99069190094425
C	-4.15097227623458	-2.91136746501829	4.28653378600033
C	-4.56751565498069	-1.85905800934794	3.42498250397012
C	-2.49833079258545	-4.61890174700980	4.93053691271530
C	-3.23758646284469	-4.90062268823109	6.07089563684795
C	-4.45353786004478	-4.21580922699107	6.33432442399624
C	-4.89461159947041	-3.23436144124997	5.46068730060519
C	-0.91333453436016	-3.98986920049227	2.46137928765459
C	0.22877225136165	-3.19750853179374	2.32633802086316
C	1.53059336582575	-3.73005113681178	2.08271927020959
C	1.64854733744361	-5.10906726370441	1.93903451328722
C	0.51549465078147	-5.96557116786013	2.00817501925811
C	-0.79190451737755	-5.40876249319129	2.26006626639555
C	0.64595320975364	-7.37287537537596	1.81034113853631
C	-0.46554036183505	-8.20095869698880	1.83444817892797
C	-1.75678025811483	-7.64770912904832	2.04766821252786
C	-1.91622707580269	-6.28520630596346	2.25560113761604
O	0.09810232042559	-1.82216263421207	2.44178881656818
P	-0.53503622727514	-0.99134727826873	1.16445826074198

O	-1.90912676254133	-1.87159030197621	0.88590592131956
C	2.69539997219397	-2.82141385730507	1.97612288175633
C	2.96947039043274	-1.84953855086644	2.93887930119344
C	4.06960769492430	-0.94992363891414	2.84762457406569
C	5.01037115787020	-1.12580574554857	1.76177545959459
C	4.72744070805022	-2.11825278105627	0.78248031955179
C	3.59920539185775	-2.91783707720816	0.88210442967544
C	6.16373119594269	-0.29423099654093	1.71278090999018
C	6.35900783716646	0.70586106706780	2.65626109123668
C	5.40818466465674	0.91798087395514	3.68460103839080
C	4.28329975510653	0.10956403206521	3.77548976761820
F	2.18933956351541	-1.78781220875546	4.02513183371476
F	5.54246600283725	-2.28538453331995	-0.27086674694426
F	3.36360311093653	-3.81514403815235	-0.08815974663975
F	7.07446863721541	-0.43150404740403	0.73920895675228
F	7.43057069630454	1.50090015387562	2.59228811837432
F	5.60026181616294	1.91808910081912	4.54806565850108
F	3.40493629586571	0.36431488057544	4.75028826025741
F	-3.93594236079209	1.17785632750104	3.14799739119344
F	-5.11656525075465	0.09113493810376	-2.09981707067036
F	-4.52497880771349	-1.88217817620115	-0.38316441684584
F	-5.36231698971428	2.65054327007865	-2.55565922053001
F	-5.33199040828233	5.17305506402247	-1.61830661609401
F	-4.79224651611641	5.69284546920694	0.99816951226950
F	-4.23048443297387	3.69934912470270	2.71364139352261
N	0.39399170048327	-1.18864934715195	-0.11724297281206
P	0.85039030423797	-0.05541243650068	-1.15756036852687
O	0.14575816816054	1.28062900256224	-1.26489236600254
O	0.81474950774876	-0.74960928483245	-2.67371301880077
C	1.64160585429681	-1.84713967472099	-2.87108152869525
C	1.04958255950664	-3.14541956579513	-2.85116839643863

C	1.88173602676021	-4.25021790537538	-2.99137758976534
C	3.28796452151492	-4.10669783945880	-3.14011046743811
C	3.87349553867417	-2.78810207039664	-3.16567436097772
C	3.01042466293213	-1.64119182971515	-3.06173667497946
C	4.13630054537217	-5.24975482564505	-3.23247866579050
C	5.51155872034408	-5.10763340992251	-3.32980522994502
C	6.09098910030462	-3.81053738012850	-3.32564821780701
C	5.29261497007112	-2.67873340869149	-3.24684390450762
C	-0.41330977225220	-3.29676709221394	-2.67114897109209
C	-1.32940523225133	-2.68705530170423	-3.52408999696875
C	-2.73988454771772	-2.83278141614574	-3.39357535559926
C	-3.23886380299811	-3.70509697510724	-2.35019246649074
C	-2.30142942125535	-4.29291364179377	-1.45576852936937
C	-0.93817784642003	-4.09818976669461	-1.62211948030344
C	-3.68051848269628	-2.18091466945328	-4.24333912055544
C	-5.04493959247841	-2.38324530245204	-4.09190882825953
C	-5.52817558857090	-3.26096798122388	-3.08994910820697
C	-4.64277785849726	-3.91511317926902	-2.24445937500582
F	-6.84496717248957	-3.43684032367216	-2.96422256215546
F	-5.14890810529549	-4.71743420090145	-1.30181961558561
F	-3.27619864968789	-1.33177340618629	-5.20076872597588
F	-5.91892330993782	-1.75087884448723	-4.87827875362702
F	-0.85526380091063	-1.93280860170653	-4.53359229997261
F	-0.09508581455601	-4.66564875564990	-0.75108308950015
F	-2.72155383434850	-5.03732356665181	-0.42376668962659
C	3.51125976717279	-0.24077674215757	-3.10926074833663
C	3.20882525086702	0.61973651172726	-2.04846219331918
C	3.59076751691941	1.99948456662339	-2.04443372743973
C	4.31445359754639	2.48474266891042	-3.12860440752552
C	4.63365763381789	1.66239292321889	-4.24315648763027
C	4.20617408925801	0.28421083329465	-4.25609577218351

C	4.44853472993964	-0.48565273246040	-5.43325458697690
C	5.12197531277361	0.06004364738252	-6.51714591894123
C	5.58447163616000	1.40304628659966	-6.48265539650057
C	5.33678272940804	2.18892460476847	-5.36813711092612
O	2.49214011048946	0.12282978023390	-0.96932800475660
C	3.17107896871647	2.90153774368097	-0.94330945278972
C	3.50752936791211	2.62596849360506	0.40578612123965
C	3.08098887524248	3.42239773894958	1.45683923102047
C	2.27380379275036	4.56620611289323	1.21903565556597
C	1.95255464457063	4.91109466239792	-0.15170208068842
C	2.42737798649949	4.05461256262043	-1.18387908063086
C	1.17329662466843	6.08043028108787	-0.39293589751389
C	0.68203239590507	6.84030894327973	0.65787127917207
C	0.95745866066787	6.47353417548516	1.99938223740893
C	1.73636086484228	5.35756868829376	2.27346677150765
F	4.26330514151438	1.55748466480374	0.67243638029423
F	3.44311946301254	3.09160049967167	2.70138397037070
F	2.09223660603164	4.34595784136548	-2.46274677000243
F	0.87700625520548	6.47332388205510	-1.64289524042648
F	-0.05675599297843	7.92974345284547	0.42425399521454
F	0.45313896180128	7.21521695143846	2.98748565187639
F	1.96519909622456	5.04703687260798	3.55217861419680
N	-1.01341775061186	0.46273837871413	1.69114800011536
S	0.10712597443761	1.53167607327913	2.16683179718375
O	1.50283421609088	1.06318692474261	1.92844233364088
C	-0.07240835400435	1.52281052506181	4.04401639358405
F	0.80788897335399	2.39720138663522	4.55398078600136
O	-0.27701448802377	2.90854567959063	1.78067263794229
F	-1.31333727425325	1.89201794290423	4.38971044992012
F	0.17956876709622	0.30416165222127	4.53305562848847
H	-0.34870476000736	3.63526445961459	-1.41803675123514

C	-1.45839446727923	3.66935164636253	-1.38738407720128
C	-1.96887787458195	4.97568999893830	-0.83996642214675
C	-2.28293191624555	6.08599953792048	-1.54481360987177
C	-2.16368863381168	6.11567429648245	-3.04804039267995
C	-1.40166867811051	4.92321488594695	-3.62043357492041
C	-1.90917639779281	3.61411513488766	-3.00807834476555
H	-3.01398892410653	3.58396842542727	-2.93327127374602
C	-2.80671055756470	7.33109253819746	-0.89022694844227
H	-2.92094418822993	-5.86483821062652	2.39913266897519
H	-2.63990144233133	-8.30438914002268	2.04058302807916
H	-0.35274683528334	-9.28407350414272	1.67591165803100
H	1.65014725717672	-7.78582524362567	1.62739194467216
H	2.63665809503730	-5.55394318203965	1.75235061178090
H	-1.55258464128854	-5.14723328063764	4.75083866224050
H	-5.82145427503111	-2.67606674186265	5.66462210701028
H	-5.48435109811265	-1.30077424715123	3.66607981336430
H	5.75578571827279	-1.68324261565992	-3.21687724845321
H	7.18499282857308	-3.70179843563101	-3.37467182291836
H	6.15719919851201	-5.99630688340021	-3.39534055622402
H	3.67395264819091	-6.24880080945364	-3.21309129046281
H	1.45007442525162	-5.26187358137449	-2.97590781814257
H	4.08849257868476	-1.52225032935479	-5.47889850863782
H	5.29597505676234	-0.55393132569950	-7.41382890789000
H	6.12447840792530	1.81975089879489	-7.34614553203357
H	5.66829288796404	3.23838045266849	-5.33504698826287
H	4.63040181637647	3.53828040615084	-3.13999109138947
H	-5.03290071404642	-4.45346213916474	7.23914445234923
H	-2.87255059951990	-5.65748476215438	6.78159025805165
H	-3.83659100938945	7.54411247940946	-1.24857826897789
H	-2.18106662053037	8.20691557912185	-1.15941631548687
H	-2.83408865927280	7.24070559051970	0.21070352688237

H	-1.66403146768186	7.06050176138449	-3.35183682454997
H	-3.18990989355220	6.17271578003852	-3.47921695167860
H	-0.32003456101864	5.03518604692715	-3.40589139369006
H	-1.51196450564000	4.88483557802515	-4.72325211367542
C	-1.40403552610070	2.36666427353723	-3.53389994132522
O	-1.96174952430383	2.61473697088245	-0.66503120448883
H	-2.07197909213010	4.95866069366027	0.25759747123340
H	-1.21256792139223	1.94691150635129	-0.61305673923726
C	-2.20705849540577	1.16519433224814	-3.30755228249563
C	-0.08373270703942	2.29340270361387	-4.18313423200550
H	-3.25421694168955	1.28813173160969	-3.65297217649773
H	-1.75266000964961	0.23676743697417	-3.68740681925824
H	-2.31857805829546	1.12376376039895	-2.18696717312772
H	0.30059389526616	1.26115276057695	-4.25698321817146
H	-0.20403658226513	2.70922037737422	-5.21400721826384
H	0.65518551264955	2.95729152546172	-3.69127683086415

B(minor)

C	6.64864561133893	0.76448137838366	2.66797417895430
C	6.41944776959684	-0.32039371789892	1.83181919506499
C	5.21191152631442	-1.06850028215470	1.92276194132244
C	4.23884492662818	-0.70781892275117	2.93306927075644
C	4.49835074106471	0.42834307711527	3.75310439132013
C	5.67564243869650	1.15143083633962	3.62264429212283
C	3.07568458717361	-1.51941124540301	3.06634430201851
C	2.77139757461158	-2.57361377896245	2.20377186294216
C	3.71655631389820	-2.85347898351297	1.17750577695779
C	4.90429960403014	-2.14872565512106	1.05088789299716
C	1.54482212933781	-3.39214670330241	2.34251869217274
C	0.27152906371935	-2.76056413229076	2.48248767588463
C	-0.91788291454832	-3.46771688532006	2.65515311247866

C	-0.88242118566005	-4.90407715034045	2.56665466636960
C	0.39377587942259	-5.55970218278995	2.41368847245850
C	1.58120322515293	-4.78319715695742	2.32507652152376
C	0.44062214607306	-6.98326594738498	2.32693180922987
C	-0.72268870995433	-7.73615274598117	2.36244859233060
C	-1.98195560381065	-7.08825398754044	2.47647324243655
C	-2.06037140307341	-5.70653795325150	2.57778396953698
O	0.22548128587987	-1.37941635891387	2.45758657141013
P	-0.35347313273030	-0.63475263183551	1.09227116133682
N	0.48946822044037	-1.14099073606283	-0.17706413602272
P	1.13881141926823	-0.22956135391007	-1.31226591671115
N	0.65175583670583	1.25562006943404	-1.69040136452759
S	-0.87129561505426	1.76444000114876	-1.75101867829925
C	-1.26402033545719	1.67054335573727	-3.59381426983428
C	-2.17417287648858	-2.71445359984324	2.91961148742796
C	-2.60224835143833	-1.71727330167512	2.03499294526277
C	-3.85489343776177	-1.03463964931003	2.20363117863577
C	-4.62337044892291	-1.32736131402563	3.32478910701196
C	-4.18768307126066	-2.25619713730528	4.30755236495756
C	-2.94233403088902	-2.95823170972046	4.11522633925643
C	-4.95739752855268	-2.49925462941480	5.48460726482088
C	-4.50924108218837	-3.37895500597472	6.45739719549483
C	-3.26335836569609	-4.04092960488142	6.29009240745224
C	-2.49990562779156	-3.83747857460439	5.14902797400870
F	3.46057676393020	-3.83382901833142	0.30058427213510
F	5.75326193983846	-2.49240809451932	0.07285646285009
F	2.25285136505738	-1.27311927386377	4.09952032021108
O	-1.82654640145040	-1.39561542302358	0.93391047485296
C	-4.31094861491734	-0.03842520710661	1.20397414340777
C	-4.40336625106417	-0.38933338401830	-0.16710941003921
C	-4.72176749015604	0.52761806015202	-1.15276879298871

C	-4.96704522862843	1.88865177837987	-0.82003106270481
C	-4.94818650511728	2.26864669189916	0.57738715793933
C	-4.64822725662558	1.26800605777324	1.54392262275027
C	-5.11992355038988	3.64123383185607	0.91532801907015
C	-5.29548295012613	4.60266720398951	-0.06928262595377
C	-5.32541393695310	4.22956448650775	-1.43630951018699
C	-5.17419266058223	2.89788049352633	-1.80317298757233
F	-4.15635704945234	-1.65796705079612	-0.51376317802518
F	-4.75065443217370	0.11486101412064	-2.42850132647552
F	-4.61380568502723	1.61858311618336	2.84528108418201
F	-5.21766304566547	2.59446778953418	-3.10486296353020
F	-5.07076299022063	4.05586513510679	2.18995696586416
F	-5.41888061545000	5.89571432046419	0.25289922513120
F	-5.50221694939837	5.17542924447636	-2.36123408782083
F	7.35340881730813	-0.62388643189712	0.92282496346025
F	3.60105932154411	0.85331171157763	4.65580142107497
F	5.90274398888079	2.22718306841328	4.37972317992338
F	7.77255794396479	1.47680817490055	2.56551588916232
O	-0.93408227439458	3.20650922435228	-1.42248707033651
O	-1.86082443091783	0.83282072328309	-1.14306035391759
O	1.20864102043314	-1.09137815513368	-2.72198270260007
C	1.99459646095569	-2.23570177599388	-2.73300402237867
C	3.38222234058566	-2.10958412467662	-2.83482740381206
C	4.18167614692055	-3.30499582225089	-2.77438563176644
C	3.52048966106632	-4.58441264124846	-2.66862687573794
C	2.10011249587383	-4.64525195336406	-2.60817961919013
C	1.32686087392058	-3.49182999515028	-2.63804442487360
C	4.30763442121337	-5.77218377967900	-2.60180866765950
C	5.69240965447364	-5.71091621551419	-2.62205695001587
C	6.34367656575155	-4.45083380417308	-2.69621548949322
C	5.60705890413918	-3.27764174839953	-2.77037159829425

C	3.94932908869452	-0.74007468018116	-2.97464082669716
C	3.58573382106273	0.23850214135358	-2.04570639569946
C	3.97930310342117	1.60563704987298	-2.16060871805868
C	4.79908246947660	1.97102282553020	-3.21952090820482
C	5.20592041808598	1.02327376284503	-4.19976421223973
C	4.75431697833768	-0.34512644107932	-4.10371785109858
C	6.01416649736497	1.41789272094141	-5.30756312685999
C	6.34183727844684	0.51516625457394	-6.30698308270611
C	5.85449151096588	-0.81722629205115	-6.24184440775350
C	5.07833374595145	-1.23608194033025	-5.17022518974069
C	-0.15374529707664	-3.51334433267575	-2.57279732675898
C	-0.92909285793136	-2.93923877089964	-3.57742278723507
C	-2.35196225242081	-2.91239030511576	-3.54260156196858
C	-3.01781215967406	-3.55880343720183	-2.43022289328420
C	-2.21866021886545	-4.10640765072342	-1.38678908063091
C	-0.83395557526920	-4.08762880842027	-1.46796978106521
C	-4.44054099924557	-3.59348280637556	-2.41403343455776
C	-5.18111095124614	-2.97089908971349	-3.40869898601434
C	-4.53118332669141	-2.31332396278301	-4.48244671966219
C	-3.14500595657718	-2.28826200457604	-4.55009161800926
F	-0.30057125950553	-2.40054074415230	-4.63162511968445
F	-2.79087394290178	-4.63808469257247	-0.29495281527414
F	-0.11655463422431	-4.60495919100493	-0.46071809538053
O	2.75959798955811	-0.10830669932811	-0.97891125433757
C	3.44642125292525	2.59926864845111	-1.19570179251203
C	2.57287305607441	3.60748041224332	-1.59520206377758
C	2.00176255154379	4.54084603426006	-0.68237927593966
C	2.36204377783152	4.43874724636710	0.71778986089478
C	3.22750734438559	3.38470733231942	1.11997843279420
C	3.74629039436445	2.50381952616575	0.18458173917554
C	1.86030284780048	5.41639531525357	1.62075833863092

C	0.99863917568996	6.41477358625479	1.19243439351878
C	0.59003534352905	6.47414462805426	-0.16242343644978
C	1.08342304323984	5.55696925726580	-1.08118171675602
F	2.24379797926089	3.68702301479392	-2.88823559333233
F	3.56289926600062	3.22759239746318	2.41548380696401
F	4.57278227209499	1.53403540579988	0.59810655214284
F	0.68030359818219	5.66462610451665	-2.34681258273417
F	-0.28117647150737	7.41591541098434	-0.53507637058634
F	0.53841450646453	7.32736556012239	2.05427646231422
F	2.19194613533401	5.38348600744369	2.92624355936931
F	-5.10253002661956	-4.19047874509282	-1.41542418999460
F	-2.57867422625522	-1.66465331980610	-5.58882481710280
F	-5.27142875801057	-1.71900317529390	-5.42132723153716
F	-6.51574710587167	-2.97705094413275	-3.36705091852309
O	-0.39959804228263	0.83405224437709	1.46628284673818
H	6.12275814966343	-2.30834865197697	-2.79945212461258
H	7.44309074662888	-4.40288975176637	-2.68499772474102
H	6.28865996277933	-6.63419654310624	-2.56699869719959
H	3.78927831250344	-6.74059404153075	-2.52589003145629
H	1.61083343595964	-5.62762203362407	-2.53161073224690
H	4.69732526294588	-2.26559765578613	-5.14303372335320
H	6.35977068161904	2.46203996827167	-5.35814053567669
H	5.11895844345217	3.01848674091458	-3.32346458335927
H	-3.04066519695474	-5.21559162746032	2.64492802456150
H	-2.90542270994190	-7.68686712330946	2.47574362956781
H	-0.67531622879307	-8.83296588271633	2.28726277483169
H	1.42211997224647	-7.46996233188856	2.21692352014521
H	2.54480435806320	-5.30279800470689	2.22314171727744
H	-1.53435618627241	-4.34947564599614	5.04042917737710
H	-2.89664758371862	-4.71961072685829	7.07505462606267
H	-5.10838969672576	-3.55658477948950	7.36312619128393

H	-5.91203961308255	-1.96417547484709	5.60610654648318
H	-5.59004369868597	-0.82162530286266	3.46346798757857
H	6.96198569593624	0.83215425493160	-7.15888591018692
H	6.08788913644599	-1.52411745231282	-7.05239275713149
F	-1.20156005522272	0.39419221452317	-4.00649128204757
F	-2.50635783513254	2.13489965071494	-3.79641051488870
F	-0.39647325828325	2.40498867714228	-4.29924182846082
H	0.94962549506588	1.29201857981894	3.99382234609742
C	-2.19041563244622	6.05967625075574	2.25506499869290
C	-2.78749768102488	7.29875211817729	1.65387307039287
H	-2.08880965939524	8.15561501036945	1.75452361403211
H	-3.72045537620790	7.57696397380252	2.18959270249186
H	-3.02968558684341	7.16214929540550	0.58400028561701
C	-1.86904254010950	6.09712313472375	3.72802855081322
C	-1.97020455266737	4.94631900590687	1.52373237371388
C	-0.98274805325146	4.93922772619416	4.17700014121681
H	-2.82619689464758	6.10413265348784	4.29925434112253
H	-1.37369644509194	7.06097558700622	3.97064180976114
C	-1.54765243565226	3.60160464133597	3.67932169319330
H	-0.91358626743393	4.91121921216072	5.28321295422169
H	0.04745756489141	5.08574150175895	3.79852680654482
C	-1.43568169684156	3.61802917816582	1.99203991764611
H	-2.64409240285639	3.53882612593536	3.82345820309889
C	-0.89417423721199	2.38406501901975	4.08942965988484
O	-2.17988537744605	2.61610725602636	1.43767773331443
H	-0.34950761118071	3.50468365117495	1.76863176391560
H	-2.22933092368858	4.91551323246636	0.45168688039377
C	-1.72050383740512	1.18154217054174	4.22188163028567
C	0.56770356648934	2.29673981996950	4.25662969595530
H	-2.59579778133536	1.35461816350484	4.88107336646754
H	-1.16595641023400	0.26580713474182	4.48861587550438

H	-2.18211632461506	1.05293674988362	3.20800534575701
H	1.13295712190409	3.09272447754212	3.73763853596955
H	0.77109715452438	2.42855722285234	5.34860299617656
H	-1.56213359985119	1.82739887019427	1.27720421033399

TS$_{BC}$(major)

172

C	-4.43042428470031	3.38548275617504	1.62692026117095
C	-4.55186696543612	2.04365637653604	1.16321659325247
C	-5.00174489910598	1.81629587901097	-0.19549497640167
C	-5.33509035204071	2.94073430591572	-0.99829197680706
C	-5.22488613735343	4.23323751088952	-0.50711061060272
C	-4.77568837835799	4.45743574619374	0.81572432912860
C	-5.05316703099894	0.47894145707499	-0.67638845520680
C	-4.66959895369771	-0.57641313345317	0.13564988409269
C	-4.22513390510545	-0.38734389854182	1.46521472402544
C	-4.18778831257101	0.91475170970851	1.95286551920688
C	-3.80864954461069	-1.55110130256091	2.28554854260692
C	-2.63250661423753	-2.28328213691662	1.94294682897913
C	-2.16351858234016	-3.35870508723302	2.70070403816312
C	-2.88809329513347	-3.72660205112393	3.89296590273476
C	-4.09944919502829	-3.01676420009922	4.22714700524669
C	-4.53010688872578	-1.93867631744306	3.40622980034319
C	-2.42719564163451	-4.73044486864279	4.79667971361933
C	-3.15073171251783	-5.05337310252313	5.93617450948664
C	-4.36833179433593	-4.38661489765077	6.23588714757202
C	-4.82670531685277	-3.38128260013782	5.39927615797521
C	-0.87153415291541	-4.00454218091067	2.34419725511852
C	0.25966813582014	-3.19248690396330	2.23786928830776
C	1.57379248691773	-3.70133742323293	2.01056462768136

C	1.71173115070491	-5.07681222321895	1.84341148619764
C	0.58978892323667	-5.94950465303467	1.87085122311857
C	-0.72882853221081	-5.41598309333924	2.11219848169383
C	0.74199039757141	-7.34973848459485	1.64064009486031
C	-0.35962736890756	-8.19103848837280	1.62252748087803
C	-1.66170179861270	-7.65915907733602	1.82506363358644
C	-1.84242659228110	-6.30443850470533	2.06439740387079
O	0.10070541346347	-1.81926500757307	2.36615735454319
P	-0.53942672113418	-0.99879005841342	1.08722869850431
O	-1.91582297609798	-1.87077192490657	0.81452422212021
C	2.73315886314792	-2.78201673454668	1.94486586445630
C	2.96913897470607	-1.80434284558793	2.91276961224085
C	4.07396910956663	-0.90659392199478	2.86191301783624
C	5.06258382483191	-1.09461971260964	1.82178300899688
C	4.81886341381437	-2.09165830212107	0.83694806382169
C	3.68387074293059	-2.88551680346738	0.89110871346039
C	6.22107189606397	-0.26896680191129	1.81933718584964
C	6.37729261052558	0.73873494269709	2.76209671937542
C	5.38084362901641	0.96403077520804	3.74340986634963
C	4.24946841189521	0.16056617652695	3.78943726443272
F	2.14989905100216	-1.73639874935660	3.96986314633955
F	5.67857544828579	-2.26718244609367	-0.17874915209534
F	3.48686415624802	-3.78598880890691	-0.08465657511126
F	7.17390039340847	-0.41770541796389	0.88930097194308
F	7.45439418335213	1.52784662347290	2.74051289448126
F	5.53753312226790	1.97036172440133	4.60632099302654
F	3.32861066857738	0.42616905578978	4.72165291481696
F	-3.76850105778432	1.10886550823747	3.20882877272064
F	-5.46622157016273	0.21036427863610	-1.93089498368906
F	-4.74114336704202	-1.82803313359811	-0.33714693739526
F	-5.70707756330244	2.78747454055911	-2.28259685878504

F	-5.50927630540046	5.27923405618876	-1.28896261372464
F	-4.63802016161668	5.71325781042329	1.25244315425610
F	-3.96309991496916	3.65397585871543	2.84979922043226
N	0.39624613078359	-1.17336592403347	-0.18784290816253
P	0.97478076315788	-0.08639949332975	-1.21257576266508
O	0.35006247329291	1.29331622155876	-1.35884830360465
O	0.96327960165086	-0.79255660842433	-2.72032053198739
C	1.75420938158894	-1.92182747413658	-2.88882013889478
C	1.10920893841732	-3.19449077451359	-2.89510612166047
C	1.89949339082050	-4.33295882311784	-3.00663252555456
C	3.31526701501013	-4.24751772125620	-3.09928769006326
C	3.95488306565354	-2.95396872290444	-3.09780731940790
C	3.13725850057297	-1.77271274948316	-3.02559354624542
C	4.11965833241575	-5.42406360185498	-3.15941412013675
C	5.50253708518061	-5.33782961404250	-3.19704230171726
C	6.13379219729948	-4.06551913039246	-3.16468083751069
C	5.37937510782424	-2.90230731144322	-3.11771636639123
C	-0.36329627341715	-3.27791569785367	-2.75716588226557
C	-1.22672605338741	-2.61398400769648	-3.62480178708978
C	-2.64519382451957	-2.67149010242828	-3.51088705917595
C	-3.21163186498067	-3.49947513315901	-2.46524108636075
C	-2.32460697268402	-4.14490102684440	-1.55867070604843
C	-0.95022710255518	-4.04340454157909	-1.71468566297223
C	-3.53172810505003	-1.96249126205161	-4.37342487896764
C	-4.90810533609465	-2.05591913105801	-4.22187761504884
C	-5.45901518626810	-2.89678231962431	-3.22326918030003
C	-4.62735670521737	-3.61182344832106	-2.37275123904058
F	-6.78504267107871	-2.97303582649328	-3.10019998275343
F	-5.19564951913454	-4.37569081130210	-1.43422905292050
F	-3.06101055999655	-1.14965883779289	-5.33151719464832
F	-5.72869193993180	-1.35818103100692	-5.01041003355998

F	-0.69378905020682	-1.88578374860266	-4.62191586629653
F	-0.15332392324866	-4.66186068931836	-0.83449713678558
F	-2.80249306376891	-4.85005302444372	-0.52358660882981
C	3.68913540161149	-0.39180955401247	-3.06277262772812
C	3.36658690043096	0.48758889892784	-2.02409225611391
C	3.73926806103830	1.86861355447321	-2.04264693221622
C	4.50359181497887	2.32861672918892	-3.10935692931944
C	4.87017700106198	1.47959116843499	-4.18871928810312
C	4.43311517304253	0.10437932905793	-4.19160416312360
C	4.71353278521669	-0.68895720071164	-5.34449181719451
C	5.43518041028618	-0.16977595890056	-6.41019857263104
C	5.90815619434191	1.16962133110813	-6.38236634083121
C	5.62206690450409	1.97899572846258	-5.29425192114861
O	2.61323408592110	0.00695572537452	-0.95652990743412
C	3.26668753134073	2.80507784167658	-0.99244552156460
C	3.59531948612158	2.61070876993382	0.37034909172593
C	3.13055753886475	3.44332640648454	1.37572738571561
C	2.29217483252540	4.54818648861406	1.07050080963092
C	1.97088630446998	4.80679611971890	-0.31970504318094
C	2.47414239967924	3.90801806472544	-1.30209656854193
C	1.16255609312133	5.93926302055593	-0.63127994892279
C	0.64498922911475	6.74611459359832	0.37088215505117
C	0.91997162474160	6.46217006966612	1.73245964668178
C	1.72603570733408	5.38485321028410	2.07424387536673
F	4.36954184572247	1.57191218937856	0.69939871401917
F	3.47430168275458	3.17340497280431	2.64148417739541
F	2.12550418331012	4.10443066755540	-2.59056941141273
F	0.86240486826588	6.24927306574501	-1.90264995288243
F	-0.11580697986193	7.80289000163926	0.07238191968628
F	0.38960786385942	7.24511418251960	2.67439133849151
F	1.95802620152508	5.15702762711321	3.37076061889805

N	-1.02696319953377	0.44744656365926	1.62876001972613
S	0.06286092107309	1.56103901150784	2.01859272263030
O	1.47456704812907	1.15994845535957	1.77656722079276
C	-0.07580212217610	1.68117891416890	3.89526115968243
F	0.83785070603029	2.55781914550122	4.33210129688018
O	-0.40513533530537	2.90088555580973	1.54798615650809
F	-1.29742210011063	2.10090569462586	4.24167881690967
F	0.15783487250603	0.48278143116717	4.43833839858257
H	-0.54470143701250	3.79364908957316	-1.35492382529659
C	-1.65663080006226	3.86797810782925	-1.32885107258988
C	-2.08258008964022	5.23094127034137	-0.83034719355562
C	-2.51005512825728	6.26148144382719	-1.59272474301858
C	-2.54647100494066	6.14241756517896	-3.09774277728438
C	-1.79190618480893	4.91999975148355	-3.62233038497986
C	-2.17974654206215	3.67402403897295	-2.81052714924621
H	-3.28363555134104	3.61298308144090	-2.71183675370265
C	-3.00909778799466	7.54939015649652	-1.00374613992992
H	-2.85444620580981	-5.89938689534139	2.20109958203586
H	-2.53591227975372	-8.32644012126303	1.78443425022620
H	-0.23050893271040	-9.26833844113801	1.43890482129171
H	1.75435358551345	-7.74587822968207	1.46577322707677
H	2.70886345261594	-5.50594132401593	1.66929694689622
H	-1.48008986728086	-5.24601195530027	4.59002503278579
H	-5.75466848343167	-2.83623653535280	5.63170446177362
H	-5.44372175632346	-1.38939190210442	3.67829363529406
H	5.87996410133839	-1.92592945280235	-3.06778802900247
H	7.23238803632119	-4.00135336804818	-3.16697834857977
H	6.11367393375035	-6.25204166072200	-3.23740773667047
H	3.61705028937971	-6.40364152123991	-3.16340813741830
H	1.42556255921330	-5.32561539016783	-3.00967336938610
H	4.34558161174113	-1.72275127709124	-5.38651310894822

H	5.63813250012454	-0.80181715253240	-7.28802383066076
H	6.48599169859524	1.56528061234271	-7.23113678187594
H	5.95891406016457	3.02692249923148	-5.26872257895063
H	4.81282949662901	3.38405283961978	-3.13577305778025
H	-4.93466662832238	-4.65739569068264	7.13962931375686
H	-2.77143665158740	-5.82949805883127	6.61803347349585
H	-4.06665479122032	7.72018312081339	-1.29925005699806
H	-2.42450276251781	8.41273266523118	-1.38592605841056
H	-2.95376474441928	7.54654364638410	0.10033446054624
H	-2.12684643065478	7.06754781182171	-3.54897277916505
H	-3.61241842426494	6.11317934141458	-3.42231252075193
H	-0.70009680619487	5.09278629978938	-3.53140412733777
H	-2.01216569805545	4.75599259436888	-4.69599276027437
C	-1.68856086301659	2.37173138418113	-3.32064071409885
O	-2.18542976337469	2.85745474491287	-0.50625439818693
H	-2.06228507469594	5.32379189174185	0.26852712304684
H	-1.54308182342287	2.72588424969092	0.24021319543116
C	-2.48071376471380	1.18365657401962	-2.97679313971303
C	-0.37778089466751	2.21475642756765	-3.85084492107405
H	-3.54659530396317	1.29873946073259	-3.25706915266613
H	-2.06017087952096	0.24069941032569	-3.36027735701532
H	-2.47813652917660	1.20031317298473	-1.85215251555793
H	0.07813075406821	1.88133896517976	-2.78780520329791
H	-0.20074295383660	1.29847946543124	-4.44260480046527
H	0.16586087140360	3.11332605084541	-4.18208132782901

TS$_{BC}$(minor)

172

C	6.50780189120070	0.80585343776920	2.81824832322420
C	6.31241828874450	-0.25200360158153	1.94001725757821

C	5.12925946696790	-1.04135217645264	2.00044860722788
C	4.14592784587360	-0.75249297022429	3.02379759929402
C	4.37639384936469	0.35054469062451	3.89595555895581
C	5.52619343203089	1.12052088492013	3.79047190549306
C	3.00422164036186	-1.59944869836222	3.11888053063801
C	2.73563311923824	-2.62878385180345	2.21504865904076
C	3.69144432814463	-2.84254036200798	1.18356817717487
C	4.85401817178722	-2.09258607936359	1.08329803811495
C	1.52860804176473	-3.48190429952081	2.31787370883572
C	0.24096436808342	-2.88306748406871	2.46104388717030
C	-0.93622144740042	-3.61442110837846	2.61406315051878
C	-0.86914482558637	-5.04754076478271	2.49912514447879
C	0.42135920038032	-5.67274779477455	2.33653619539672
C	1.59391033880966	-4.87099891831131	2.26967280417255
C	0.49756300265639	-7.09292762317875	2.22079390839275
C	-0.65026681558092	-7.86994249141217	2.23777145127260
C	-1.92304649939604	-7.25105746436130	2.36188004581438
C	-2.03042207804316	-5.87353339125900	2.49040407026072
O	0.17042769327188	-1.49851803053791	2.45533232933430
P	-0.42756531957396	-0.75244154577162	1.10901580202139
N	0.43967615303879	-1.12120387584627	-0.17165881023826
P	1.09994726914030	-0.21531593192505	-1.30704975809320
N	0.61741096957475	1.26667220134225	-1.69000523166099
S	-0.89883424337030	1.79915422547368	-1.74192162059944
C	-1.36240853027499	1.59043997902519	-3.55940431061963
C	-2.20452216541802	-2.88488591272512	2.88944059890649
C	-2.63695642183173	-1.87080238765762	2.02927630143331
C	-3.86592453549657	-1.16075063471528	2.22771700385118
C	-4.62937692956025	-1.46757219385300	3.34799732101681
C	-4.20566462380900	-2.43677529895967	4.29744594373453
C	-2.97129566896816	-3.15170326791974	4.08156991275295

C	-4.97476258466294	-2.70195888846221	5.46994363672936
C	-4.53598142609448	-3.61470299063178	6.41621233217392
C	-3.29947083996611	-4.28775179385842	6.22749940723728
C	-2.53618579716750	-4.06258958170606	5.09036809535966
F	3.46617921415904	-3.79798598250670	0.27159652909013
F	5.71068578995264	-2.36477889464837	0.08951431322336
F	2.16402490860458	-1.41218966887296	4.14945997470511
O	-1.86573226690122	-1.54142602894846	0.91783783137479
C	-4.29918318827086	-0.12679629807986	1.25662278632852
C	-4.44201737733667	-0.45569132403863	-0.11557066227000
C	-4.75953287702257	0.48374087960656	-1.07952821225247
C	-4.95965095751453	1.84607959701109	-0.72079760405469
C	-4.88487365574791	2.20678269175520	0.67972931574351
C	-4.57455336176299	1.18656652442541	1.62315518531825
C	-5.01958541423044	3.57782235293853	1.04040658401487
C	-5.19989632700505	4.55603152478862	0.07423179932388
C	-5.28075862909085	4.20225686708662	-1.29574298308324
C	-5.17270282856819	2.87279611553373	-1.68407027231917
F	-4.25132159732152	-1.72964921456559	-0.48314107987848
F	-4.83823239038183	0.08828066329742	-2.35953215432364
F	-4.49145501736165	1.51602716715379	2.92812722792494
F	-5.26727529195472	2.58703414120254	-2.98782881777260
F	-4.92220392992166	3.97507417532503	2.31654016253728
F	-5.28382534539953	5.84661904864700	0.41605271541751
F	-5.46274262094984	5.16427069063992	-2.20300298243117
F	7.25300408095673	-0.48667319504817	1.01800776690694
F	3.47938614220250	0.69444136784690	4.83338668018040
F	5.72383341413419	2.16387982699136	4.59857610804071
F	7.60677368486482	1.55864057017178	2.74313877517858
O	-0.91904470421521	3.25839646525386	-1.50531460929435
O	-1.87669731398363	0.92643094888283	-1.03397061250894

O	1.16724034248173	-1.08693581252260	-2.70765309282135
C	1.95236358272803	-2.23384984257285	-2.71818872563051
C	3.34074252093587	-2.10894012244908	-2.80898181279889
C	4.13839919499693	-3.30573258759618	-2.75099315309158
C	3.47503533229152	-4.58511746813434	-2.66229856728851
C	2.05441815490193	-4.64424249799224	-2.61479927365673
C	1.28231762332736	-3.48971440909438	-2.63994616169562
C	4.25986330750286	-5.77454984072455	-2.59753625338503
C	5.64475577524080	-5.71458786735254	-2.60244182474979
C	6.29807224294820	-4.45461565570905	-2.65959150162261
C	5.56363074428432	-3.27994772259476	-2.73195814879163
C	3.91291615566930	-0.74110942894155	-2.94319824848560
C	3.55039857129615	0.24016396746011	-2.01678343034836
C	3.95246742335950	1.60447308898142	-2.12946855138627
C	4.78151062451484	1.96278118515646	-3.18409890208862
C	5.18680564441787	1.01317446245244	-4.16266678903529
C	4.72533893395824	-0.35182469570134	-4.06907932494431
C	6.00229873019176	1.40312868772654	-5.26684869170832
C	6.32728053484701	0.49873775018107	-6.26552652049621
C	5.83022507454530	-0.83020479980555	-6.20315876695352
C	5.04717860633848	-1.24450056567179	-5.13484482345613
C	-0.19866313445156	-3.51562393833508	-2.58421149015846
C	-0.97222911084049	-2.94311519962547	-3.59088888470923
C	-2.39560773364410	-2.92339748251576	-3.56172738758853
C	-3.06296044278773	-3.57148390818823	-2.45099806561633
C	-2.26532792312955	-4.11900007036944	-1.40622942344645
C	-0.88076501954931	-4.09746786492915	-1.48468525803621
C	-4.48564215915479	-3.60955421789605	-2.43813991809286
C	-5.22549820886751	-2.98556773209767	-3.43252165288260
C	-4.57435481054414	-2.32603726820812	-4.50440833560869
C	-3.18800517881459	-2.30187753880719	-4.57133514051381

F	-0.34091378089583	-2.39656167521725	-4.63924540867849
F	-2.83837681988761	-4.65009068331330	-0.31461690263391
F	-0.16350406503399	-4.61685891389740	-0.47805967657826
O	2.71314339707152	-0.10309499135339	-0.95408545981707
C	3.42738085265853	2.61169129774645	-1.17400998068611
C	2.57582744493968	3.63255890376302	-1.58905621743124
C	2.03274151753875	4.59856605057159	-0.69308334433061
C	2.40253261864906	4.52110947658603	0.70645239469701
C	3.24850212463376	3.45653845331829	1.12420532268217
C	3.73203244353854	2.53748784252683	0.20655735592039
C	1.92616837069924	5.52849525875615	1.59098657951479
C	1.07552983514097	6.53052920717239	1.14876654830243
C	0.66388486677282	6.57079621538564	-0.20590293791283
C	1.13364702114500	5.62525529593393	-1.10797305889306
F	2.24463162036952	3.69552054981425	-2.88259579541487
F	3.59272336127655	3.31911227518283	2.41987075767123
F	4.53304788166914	1.55355918178966	0.63792294166184
F	0.72389787146670	5.71416885735455	-2.37324562838685
F	-0.19207830816091	7.51891718667392	-0.59485441196067
F	0.63048362552069	7.46253256286565	1.99570238143996
F	2.26446131581589	5.51571510854133	2.89443512773241
F	-5.14811122154571	-4.21287097244188	-1.44408438351379
F	-2.62081289154490	-1.68009623430245	-5.61057337613233
F	-5.31335055141836	-1.73263881959810	-5.44447853224912
F	-6.55977503333063	-2.99435127051444	-3.39362483749679
O	-0.54999401890952	0.71137997879594	1.56790257735433
H	6.08070367960571	-2.31117094685774	-2.74927048000365
H	7.39735063791338	-4.40806131017319	-2.63696648005738
H	6.23952147864270	-6.63889796439530	-2.54899476166194
H	3.73962651185630	-6.74287742122592	-2.53492164563220
H	1.56293442272926	-5.62647751048986	-2.55191619502643

H	4.65900103109962	-2.27133488479278	-5.11033046800296
H	6.35501237206218	2.44494683072207	-5.31549479937779
H	5.10920950096115	3.00800328782996	-3.28610648522203
H	-3.02047500507456	-5.40368794208535	2.56548644719882
H	-2.83371180649203	-7.86872889198303	2.34719442724970
H	-0.57998870198572	-8.96381749061597	2.14104998215677
H	1.48897559370947	-7.55723920927807	2.10361837456507
H	2.56860133883698	-5.36854640725645	2.16223903294678
H	-1.57722314352355	-4.58315789713765	4.96637852654766
H	-2.93916176521406	-4.99170767880417	6.99287795192023
H	-5.13498156595223	-3.80899027760525	7.31858346263411
H	-5.92067954687862	-2.15632980987881	5.61064649044795
H	-5.58156039985552	-0.94250060248381	3.51314646635844
H	6.95285117881394	0.81181600697358	-7.11488548909686
H	6.06141879856048	-1.53805764096433	-7.01347772026338
F	-1.32176658945987	0.28925489650068	-3.88999829185975
F	-2.60792501271370	2.04992261893924	-3.74350928453785
F	-0.51737243068754	2.27071372456460	-4.34035568875586
H	1.20995201673482	1.69706123286109	4.42147429822145
C	-2.14694994949749	6.22490946391267	2.14119317487001
C	-2.71508681843532	7.47146979407154	1.52595994019430
H	-2.02403223358337	8.32899643581327	1.67089506707465
H	-3.67405112586716	7.74382841245903	2.01677962747521
H	-2.90197921503588	7.34857336317190	0.44303421962130
C	-1.91291467402976	6.24475799183469	3.63301961018664
C	-1.85757652336311	5.12678521329924	1.41097124292730
C	-1.00164736777621	5.11338438499597	4.10733442816252
H	-2.89924309986489	6.19535213227722	4.15045295978529
H	-1.47575011805231	7.22439911331530	3.92180432725093
C	-1.46690154385520	3.76570876917822	3.52995244110673
H	-0.99096982471184	5.06447694779058	5.21545562003935

H	0.03851205319059	5.32134152482833	3.78677944080758
C	-1.37093822500399	3.79818502981976	1.93690376570306
H	-2.55163501033636	3.62967411655296	3.71484224198076
C	-0.73009256188072	2.57688999161025	4.03045742020296
O	-2.18470812043088	2.80266081678258	1.38266374162833
H	-0.30537097714480	3.64342963274235	1.64094388513048
H	-2.02952122001394	5.11263003425191	0.32156289339682
C	-1.35220616459995	1.31654410575844	4.05582485337087
C	0.72095433877471	2.68375039857589	4.33095406151115
H	-2.45182555884746	1.26825103166502	4.02218756743694
H	-0.83303844761204	0.49651555911043	4.58395349043838
H	-0.98679393062526	1.03995907153418	2.87530708542212
H	1.25760812986139	3.31010774146502	3.59215919936471
H	0.83188722163524	3.21788226856179	5.30195204975980
H	-1.64035496192290	2.02190117940338	1.12403831911562

C_1(major)

172

C	-4.45682698816800	3.38330553227768	1.51691715145006
C	-4.55410867065561	2.03052927690315	1.08198838333432
C	-4.96897179514818	1.76838226361619	-0.28167280471503
C	-5.27999004566461	2.87111834959868	-1.12278159862795
C	-5.19179485969610	4.17545004984059	-0.65862978209325
C	-4.78957113692455	4.43299632930057	0.67287124947560
C	-5.01295521765461	0.41887473515618	-0.72946081340657
C	-4.64934642172591	-0.61369072462327	0.11942607051895
C	-4.22996640898195	-0.39012624322955	1.45229974401488
C	-4.20164573784792	0.92378306784744	1.90720525701945
C	-3.81903810641645	-1.53543902240149	2.30088367989704
C	-2.64251734651690	-2.27351486846025	1.97612176202891

C	-2.17660334591604	-3.34317275464382	2.74209192807466
C	-2.90630957981801	-3.69820574686684	3.93480446471862
C	-4.11518613247857	-2.97904160597267	4.25885590749597
C	-4.54307971231728	-1.90961815277207	3.42446410152998
C	-2.45227534961787	-4.69705990882463	4.84708027603859
C	-3.17924927267505	-5.00560194017810	5.98838980893569
C	-4.39317741585764	-4.32878880062360	6.28010534828953
C	-4.84548294348167	-3.32898060540199	5.43351072410233
C	-0.88771810505674	-3.99790077651112	2.38851006326666
C	0.24953922928847	-3.19623048109761	2.27675065433876
C	1.55802934920222	-3.71168539305255	2.03575672426166
C	1.68569027367531	-5.08788751013498	1.86749529403443
C	0.55808742881784	-5.95296073799220	1.90608127040014
C	-0.75544359255399	-5.41054812458893	2.15628316233151
C	0.69956182406564	-7.35468267425252	1.67816293035259
C	-0.40775264891839	-8.18855000890460	1.66958338130327
C	-1.70490553780123	-7.64772078597278	1.87956054128042
C	-1.87527475612265	-6.29143210647094	2.11747906868623
O	0.10401713346857	-1.81913601028226	2.41219143939481
P	-0.55489260422818	-0.98705528228978	1.15912292966105
O	-1.91022615408716	-1.86462667556870	0.85123742377517
C	2.72050516801690	-2.79726039500674	1.95489495297687
C	2.97650295535484	-1.82642617232723	2.92438789211106
C	4.08045039866006	-0.92860667186109	2.85757039306577
C	5.04825157557536	-1.11040252493972	1.79685199110020
C	4.78491230521903	-2.10194866659023	0.81126819139387
C	3.65015884901870	-2.89502383715158	0.88208813906127
C	6.20618185498001	-0.28426921929233	1.77586869866770
C	6.38083274292761	0.71820183908054	2.72116041728744
C	5.40398715335463	0.93763442774245	3.72339690553727
C	4.27450261912658	0.13268351558679	3.78802538483078

F	2.17688531278583	-1.76576778753067	3.99655728954278
F	5.62398735320045	-2.27150727984294	-0.22252703604320
F	3.43261767811526	-3.78861377170302	-0.09624094198260
F	7.14001301606452	-0.42749085670395	0.82609240000029
F	7.45660060917851	1.50774129346709	2.68271327865638
F	5.57762321041750	1.93826760098694	4.58893084005251
F	3.37297367472755	0.39143171262010	4.74054907217024
F	-3.79944772613222	1.15098449767416	3.16462219295312
F	-5.40314800455109	0.11576650921241	-1.98150075493682
F	-4.70957932874365	-1.87928843700526	-0.32068817075765
F	-5.61462285327647	2.68482239092102	-2.41076274037771
F	-5.45590797026010	5.20195152867550	-1.47221395137477
F	-4.68592079000897	5.69823537490715	1.09023582729577
F	-4.02835632212599	3.68552836132238	2.74738813955149
N	0.39130820275474	-1.14789887926162	-0.12423207693084
P	0.98403042598730	-0.14459365435281	-1.18171284338243
O	0.32955102549533	1.28221007694293	-1.30086865161685
O	0.92150508191144	-0.77846603602582	-2.69329296553850
C	1.72966760818839	-1.90854769844908	-2.89131001944539
C	1.09353080361524	-3.18276826045574	-2.89281888063783
C	1.89896823969814	-4.30950396078619	-3.01431698261909
C	3.31265476962170	-4.20447758171551	-3.12163270002806
C	3.93752489057763	-2.90352524406250	-3.12507074809946
C	3.10574234049606	-1.73259587460700	-3.03852009801659
C	4.13069115307917	-5.37072552488184	-3.19485953666000
C	5.51157228689184	-5.26644844599513	-3.25153266903726
C	6.12777659132104	-3.98676193282330	-3.22582749938939
C	5.36031201258447	-2.83278074440476	-3.16582079669090
C	-0.37771767473738	-3.28103773104571	-2.74691613311135
C	-1.24711590331091	-2.64571969149504	-3.62963647642016
C	-2.66460617246969	-2.72488651697248	-3.52178088428499

C	-3.22106415152505	-3.53251303012889	-2.45504329862356
C	-2.32758987190617	-4.14327565052375	-1.53004894605098
C	-0.95434258262655	-4.03159663727753	-1.68811403897941
C	-3.55739980660225	-2.06204128127496	-4.41396160473380
C	-4.93278195580162	-2.16918400566560	-4.26089780441144
C	-5.47402231170848	-2.98234379510312	-3.23442997842874
C	-4.63517269502781	-3.66012738345756	-2.36066174734658
F	-6.79866944301572	-3.07429954319883	-3.11239433991245
F	-5.19515570487747	-4.40536198919133	-1.40240573821285
F	-3.09547777362987	-1.29028038339515	-5.40676543677400
F	-5.76145048080935	-1.51851741466912	-5.07906719190184
F	-0.71633553211988	-1.92517958851300	-4.63298032176261
F	-0.14784020437282	-4.62034257039399	-0.79541553315131
F	-2.79815864227897	-4.82694388427258	-0.47747555839164
C	3.64090915428446	-0.34429899457374	-3.07517955956372
C	3.33568224211784	0.53213364286264	-2.03083562798760
C	3.70806627002279	1.91190440418354	-2.03738804937661
C	4.45524556990539	2.37615835601323	-3.11519644960239
C	4.79913423786438	1.53598698650813	-4.20823434271005
C	4.36297057017854	0.16078797990573	-4.21455806427601
C	4.62278462945228	-0.62502398592256	-5.37684539300237
C	5.32335036267318	-0.09642055469096	-6.45182957343606
C	5.79492750313436	1.24337127832290	-6.42280447244276
C	5.52942616880617	2.04478495127796	-5.32387598953142
O	2.59258693096211	0.04235874696695	-0.94483168095706
C	3.25498784172696	2.84231496583607	-0.97360893959162
C	3.57399452710366	2.61941110994306	0.38789458578154
C	3.11728662744594	3.44200303669408	1.40443706565384
C	2.29131786597942	4.56077942202959	1.11436171852598
C	1.98695628893587	4.85269331455840	-0.27293523964145
C	2.48898819485338	3.96886719591292	-1.26974880548070

C	1.19180068015807	5.99854394783948	-0.56975615399113
C	0.66823034754589	6.78574331658603	0.44519770928296
C	0.92666958832895	6.46975921801274	1.80286782012364
C	1.72091051633817	5.37895466158345	2.13016601953404
F	4.33078926973242	1.56254890977254	0.70262576059163
F	3.45341982669054	3.14740112874157	2.66655919977251
F	2.16703432215924	4.20321275595551	-2.55772176413853
F	0.90826364569441	6.33669320744639	-1.83524163704862
F	-0.08347436114541	7.85183958955552	0.16252146165304
F	0.39007866593602	7.23352030705257	2.75613773450833
F	1.93541744459802	5.11924064168002	3.42371975845155
N	-1.03253811354074	0.45611728528255	1.69494096396847
S	0.06465994113206	1.56480592232292	2.09934821800757
O	1.46357975383540	1.16371495029831	1.78558607813979
C	-0.02028423723834	1.60632690408615	3.98378049261757
F	0.88476747892914	2.48713149199219	4.42710437512313
O	-0.41498281526022	2.91668903532412	1.69476522476271
F	-1.24189306118523	1.98314459943382	4.37377871490014
F	0.25421716916094	0.39448234397339	4.47416644755401
H	-0.47394598222020	3.80012036057321	-1.29879550199556
C	-1.59141011600738	3.85184608852056	-1.30487200254935
C	-2.02675466585023	5.20123982828085	-0.78020172351484
C	-2.44381606051741	6.23347532291341	-1.54796182767907
C	-2.45521347864023	6.11719717415041	-3.05551640448225
C	-1.69129892928897	4.89594533692177	-3.57640083240874
C	-2.05519376045558	3.64364149878452	-2.76526541091437
H	-3.16302122717749	3.56778346777069	-2.72321069962982
C	-2.95502909426675	7.52275579654765	-0.97025630659595
H	-2.88371133918928	-5.87957667587253	2.25995262834579
H	-2.58366099001313	-8.30935598768414	1.84654144513339
H	-0.28702167990306	-9.26715673904451	1.48809206181343

H	1.70801202747072	-7.75801619756141	1.49744667823476
H	2.67852608119615	-5.52311676306281	1.68394603537768
H	-1.50812706772284	-5.22032454920299	4.64570675802974
H	-5.77128371730497	-2.77763885218799	5.65945853237411
H	-5.45780392209186	-1.35758023638627	3.68692429254417
H	5.84991614063368	-1.85061483899464	-3.12164571860188
H	7.22527331076576	-3.90936108669212	-3.24442304013934
H	6.13343338112231	-6.17278020724495	-3.30261517346831
H	3.64053869032847	-6.35644580563141	-3.19462930578381
H	1.43755396744016	-5.30796109509859	-3.01496236193967
H	4.25546587050691	-1.65892616373175	-5.42021163179322
H	5.51030425050001	-0.72128757552036	-7.33817648453156
H	6.35517498622538	1.64605977746923	-7.27991396992879
H	5.86518647657839	3.09285766391299	-5.29725341976050
H	4.76699226503344	3.43062146150973	-3.13872292767732
H	-4.96223868909837	-4.58788099694049	7.18554222009437
H	-2.80588081007342	-5.77798810645362	6.67768891969140
H	-4.01298532347269	7.68388766139693	-1.27074377251516
H	-2.37648291194987	8.38875956582447	-1.35644577592128
H	-2.90439861613919	7.52997789649792	0.13423732392877
H	-2.03769342607579	7.04863350192479	-3.49694052444789
H	-3.51726666860423	6.08274698188788	-3.39290362545168
H	-0.60040543005868	5.08379321217787	-3.49270218469638
H	-1.90851421436848	4.72849787046203	-4.65075453888797
C	-1.54339385885927	2.33311287952719	-3.33817556713390
O	-2.11537792820210	2.80291053017550	-0.50513791991765
H	-2.01656196412199	5.30054748319484	0.31910422028211
H	-1.58879556589983	2.79248147465389	0.32926297870788
C	-2.43577087747189	1.13855252827664	-3.16177299741805
C	-0.31682349900985	2.22474017424201	-3.91949558249936
H	-3.43671416745976	1.32370041402496	-3.60200668038136

H	-2.00386187717329	0.22929592365483	-3.61606990252890
H	-2.59739132101470	0.97797911940527	-2.07511061811605
H	0.02300613683457	1.61509431220797	-2.23115913476157
H	0.01645539500952	1.26738392854815	-4.35326049193510
H	0.35119879352712	3.09159533089729	-4.03422129208535

C_1(minor)

172

C	-5.01217777809577	-3.88343343961002	-2.74987711544509
C	-3.72340499495763	-4.39570790466137	-2.67580351910313
C	-2.91815349457849	-4.19075723269714	-1.52055263624912
C	-3.46848505171685	-3.44950591753352	-0.40306672302970
C	-4.79205227363883	-2.94138513520816	-0.52133232042185
C	-5.54849323404269	-3.14540836068989	-1.66687465982044
C	-2.65544984267184	-3.25195921303887	0.75429697505931
C	-1.36009349296551	-3.74867015450913	0.85361086346745
C	-0.85213409639644	-4.47267925317005	-0.25981442095828
C	-1.58497663373518	-4.67648921630031	-1.41630807547703
C	-0.47686561301320	-3.51776417123926	2.02364274518265
C	0.76635133738704	-2.85788171892581	1.80213746889913
C	1.74980629082492	-2.69101538883114	2.77651743168122
C	1.43516545027796	-3.10961453628159	4.11951999105914
C	0.16342427441910	-3.74561232979130	4.37529231229723
C	-0.76008227487608	-3.95274108231788	3.31059409158177
C	-0.15849122070519	-4.14717494174821	5.70555175422605
C	0.72141726571383	-3.91646280501223	6.75172084909796
C	1.95734443453066	-3.26196691280178	6.50565268600250
C	2.30562216642780	-2.86796701715041	5.22153210950261
O	0.98224554260289	-2.35331617752874	0.51842258347113
P	0.80908176957216	-0.72428204125836	0.35232577499606

N	-0.63386778615918	-0.24284455596772	0.79120462408173
P	-1.62011217758093	0.82781136647100	0.12013023219149
N	-1.13450857706146	1.82321242455784	-1.03696618749153
S	0.22834847902812	2.68093977254790	-0.96737217723122
C	-0.42864447728559	4.41195120046584	-0.61166149671911
C	3.05187578490985	-2.08550976671485	2.37806361787551
C	3.07124522078000	-0.86310552625691	1.69796597464277
C	4.27340378518497	-0.24709114113718	1.23233441272108
C	5.48027784368169	-0.88349628594307	1.47973245095558
C	5.52971793791230	-2.14277984114484	2.14042477996900
C	4.30561335521822	-2.77070926978343	2.57864009776284
C	6.77275129823472	-2.81631694269449	2.33193717295825
C	6.81843371968312	-4.07470805441673	2.91116487773719
C	5.61312057992949	-4.71049444148017	3.31060513846303
C	4.38958253019406	-4.07666264058570	3.14770799472218
F	0.38810688919843	-4.97668299237031	-0.19395216425772
F	-1.00872925303774	-5.34403831881836	-2.42649004623973
F	-3.14245578302012	-2.53910723049573	1.78142873986822
O	1.87603855966692	-0.18163099882503	1.47648833056724
C	4.19090041037457	1.03577085075886	0.47897308839648
C	4.12783801454880	2.27505276609733	1.15878790229922
C	3.92369953596936	3.47356922322865	0.49351850124928
C	3.78718728780378	3.50402595715172	-0.92327621899579
C	3.89718843143522	2.25781279808637	-1.65441589297233
C	4.11746297494315	1.07138815186896	-0.90456342564161
C	3.74717690270231	2.27361952052076	-3.06968623574395
C	3.51929790266851	3.46028597148431	-3.75048411110535
C	3.40234430835273	4.67704500292420	-3.03457392735852
C	3.53202766619927	4.69903545733275	-1.65175596919172
F	4.26925146702897	2.27343696776904	2.49132884081162
F	3.84848114505729	4.60152247947515	1.21641079917927

F	4.26558189333646	-0.09979833349527	-1.56857185351558
F	3.40668971999225	5.87391780017487	-1.02460167286431
F	3.79045856441100	1.13603432156587	-3.78418418067690
F	3.37803274725715	3.47352082009529	-5.07592795046422
F	3.15748350750361	5.80105535894098	-3.70930447168597
F	-3.25402669464797	-5.07469650205128	-3.72860005370225
F	-5.35026614821000	-2.24141478698659	0.47205175704781
F	-6.78336024690548	-2.64683115946491	-1.76314149884682
F	-5.75515198516759	-4.06711777665417	-3.84253546549624
O	0.83718518286209	2.78239298647001	-2.31095501616074
O	1.08504294157014	2.35960010171410	0.20837646708896
O	-2.37069484515732	1.72157668620121	1.28289587964857
C	-3.18526579786360	1.05663246947895	2.19197638315893
C	-4.45998040054427	0.64056479491817	1.79911560033489
C	-5.24812222276467	-0.10296897628093	2.75107543959134
C	-4.70652766646919	-0.35084255054768	4.06746056207902
C	-3.41543072556228	0.13925149353829	4.41187346413469
C	-2.64851563179648	0.84655131033603	3.49608124324258
C	-5.47825730957982	-1.08971528199937	5.01379138777712
C	-6.73175173697339	-1.57996375523464	4.68376647252850
C	-7.25601620144797	-1.35747642560152	3.38268438118593
C	-6.53299057629908	-0.63925321490981	2.44136723017435
C	-4.91693745516559	0.98511590138196	0.42241320101386
C	-4.09543241767359	0.68123913767804	-0.66740920021908
C	-4.37933318555767	1.09813428499179	-2.00772281801315
C	-5.57397900142783	1.76330409012860	-2.24597202008259
C	-6.46598061337405	2.08608293630997	-1.18548378949554
C	-6.11898564076391	1.74393840519913	0.17420593479653
C	-7.66789156878643	2.81179881066111	-1.44058839271292
C	-8.48374367585229	3.22626603937091	-0.39970240219530
C	-8.11154800680939	2.94676623392576	0.94188401474583

C	-6.95776191880837	2.22802908334731	1.22217205596927
C	-1.29903101046893	1.37755029857262	3.79938280841081
C	-0.98980960467374	2.72380621167053	3.61191826157935
C	0.31423396968313	3.25795749933249	3.81138009071097
C	1.34535682150303	2.36636427589060	4.30241219234993
C	1.02762862503752	0.98931905100136	4.47077875859697
C	-0.25399737883806	0.51963224085851	4.22993024179580
C	2.63173436028340	2.90181949723068	4.58547753658367
C	2.92632051825084	4.23159353639381	4.31645904400387
C	1.93417819204137	5.09310532857871	3.78790933096151
C	0.64919929411549	4.61991670221583	3.55538616858609
F	-1.97189543994091	3.55248066280548	3.23273767759641
F	1.97770950255676	0.11426817199574	4.83839036190123
F	-0.50653898568208	-0.78931367703170	4.37583627972084
O	-2.90916091477658	-0.02386977350430	-0.44873552207569
C	-3.30591533983673	0.96459769211118	-3.02445632189895
C	-2.70876500165574	-0.25579690645286	-3.31330634839147
C	-1.47355188181906	-0.37635213260318	-4.00638500705289
C	-0.81368532074175	0.83982783671900	-4.43883073362479
C	-1.48138269298315	2.07957876485094	-4.23570807100944
C	-2.68739837174687	2.13048332864837	-3.55207431541603
C	0.47823888810894	0.74348467921396	-5.03290669387457
C	1.07692025294056	-0.49298996726136	-5.24017605755086
C	0.39132150256471	-1.68321162074994	-4.89422978682295
C	-0.84114921658847	-1.62634684007306	-4.26477648066305
F	-3.30749360204822	-1.37155176591203	-2.86026109938081
F	-0.94315871927070	3.22595789526646	-4.66501888817514
F	-3.23612174558647	3.32743562139519	-3.31089464901085
F	-1.41240862780259	-2.78817571215046	-3.91256889648271
F	0.95968287866124	-2.86803532034892	-5.14899927582212
F	2.30181543582597	-0.58320367299833	-5.76621729980656

F	1.15652078533718	1.83879830091043	-5.38841272980943
F	3.60415836355945	2.12961229390830	5.08434043677011
F	-0.25817183305090	5.47913448630442	3.07974628826782
F	2.25095052840495	6.36258860323775	3.52618087949070
F	4.15064544545286	4.71192859158059	4.53711676059673
O	1.26323329014530	-0.44861888223453	-1.11065632088995
H	-6.93878192168936	-0.50077704133522	1.43122252150686
H	-8.24248108835676	-1.76479820375869	3.11412166442210
H	-7.31693776389447	-2.14874088925063	5.42197226117662
H	-5.05192378778121	-1.26410693878717	6.01385271446870
H	-3.02695986510159	-0.04692793457886	5.42399390612376
H	-6.67781377331575	2.03605628192370	2.26623780106605
H	-7.92297621160630	3.05150520796341	-2.48440727078665
H	-5.81886975534567	2.09187846282418	-3.26661083980242
H	3.25266821755969	-2.33985921129461	5.04826065896812
H	2.64170265480501	-3.05513315097380	7.34220853040772
H	0.45937119269171	-4.22692902679169	7.77433773876421
H	-1.12839122244531	-4.63627636819983	5.88487911983487
H	-1.71050777168434	-4.46669691424185	3.51714684643803
H	3.46802164878189	-4.59413629531396	3.44389317409589
H	5.64676474786985	-5.72078387278862	3.74547744598444
H	7.78229368084865	-4.58681868660253	3.05023836407093
H	7.69546388836709	-2.31767570803708	1.99739097854424
H	6.41724149266090	-0.42104356735436	1.13527980826642
H	-9.40571772136732	3.78959409849129	-0.60808509241584
H	-8.73900165196897	3.31047939544228	1.76963218974458
F	-1.10968976026710	4.41532964127010	0.54183771171736
F	0.60888539483885	5.25868830331296	-0.51159094821252
F	-1.23220275172698	4.81094841841832	-1.60435469409209
H	1.79984881735351	-1.18401220445899	-1.72534375215029
C	5.05133817855840	-3.82540703357156	-3.79530867818149

C	5.91288869794656	-4.03115338342526	-5.00817807814501
H	6.34897777865093	-5.05391751676414	-5.00668570771316
H	6.77442434151250	-3.32901634545419	-5.00458089113972
H	5.34788156843783	-3.89314505492665	-5.95006279327061
C	5.70759908533925	-4.03601909401338	-2.45250138980614
C	3.73797618759821	-3.50390094752045	-3.90077316087053
C	4.91129374196325	-3.43040727851756	-1.29767290013470
H	6.73898665486028	-3.62318072994963	-2.47723572607810
H	5.83329311170303	-5.13377000285869	-2.30254305257581
C	3.41755430561795	-3.81168339137026	-1.39332327939557
H	4.99731925688903	-2.32515787588976	-1.32125677113487
H	5.32966751274062	-3.74757266048243	-0.32236022271532
C	2.83173372031690	-3.30432962915655	-2.72113638512266
H	2.87235033312185	-3.29083641273006	-0.57880387267007
C	3.20749911294524	-5.30545826611978	-1.17531733573186
O	2.49710068508980	-1.86803569585042	-2.66637198614438
H	1.84522338153420	-3.78034187170809	-2.89274252430731
H	3.29153023231173	-3.34278528579242	-4.89432392143658
C	3.40665880910663	-5.77260011572242	0.24376503058653
C	2.86556494690214	-6.16905046534461	-2.15518908544483
H	2.72875407708167	-5.22930267471239	0.93508976255300
H	4.43692950703865	-5.56689664499710	0.60210644756556
H	3.21655936045982	-6.85813538057991	0.34639231034910
H	2.72405391316949	-5.85644840614146	-3.20062903986011
H	2.71721452398528	-7.23761860954140	-1.93141247590214
H	3.29569507628294	-1.34090912391309	-2.87745843018849

D(major)

172

C	-4.26653271318104	3.38095957992181	1.60447627461087

C	-4.57468516446231	1.91796012881215	1.25541362051173
C	-4.91877254287206	1.72365858368901	-0.19144207272898
C	-5.38067695924719	2.72171254783152	-0.98152648484109
C	-5.55519697758379	4.12320128444099	-0.44362730926030
C	-5.45257950769865	4.21230378836231	1.08164670415926
O	-3.38909499954752	1.09063764260398	1.58551073796657
C	-5.71387909750083	2.51083895891941	-2.43032814746049
C	-3.95111641763579	3.58559037260878	3.07802991128493
C	-5.09321926069454	3.52707668822045	4.05910185797597
C	-2.67821293555240	3.79588614382072	3.47588497276016
O	-3.81164415419836	-1.35983448460302	0.98356041697791
S	-3.21528224287854	-2.27001731456525	2.03303623957527
C	-2.79326004533203	-3.81806731036309	1.03518047763122
F	-2.03077431531215	-4.61807662124490	1.78373419220137
N	-1.81271981315969	-1.66389049350875	2.49804703956638
P	-1.34501355681091	-1.74076054852112	4.05514276002230
N	-2.19538111164538	-1.08105811244945	5.22314789882484
P	-3.50044697801978	-0.15321360399406	5.16115611948960
O	-4.75665322421484	-1.05553412855663	5.74659012023078
C	-5.84442188658537	-0.38268292574525	6.29641989601601
C	-5.72195471829453	0.15964751601602	7.57991774688218
C	-6.83061633981776	0.89497460022833	8.13300285810748
C	-8.05840859844831	0.97771948891941	7.38050910667115
C	-8.12833914053139	0.39198751369910	6.08817736573039
C	-7.03984830941472	-0.26110568539478	5.51918137176442
C	-6.75431907317703	1.59163716164089	9.37609397235500
C	-7.84862343715380	2.28474597296796	9.87409261988097
C	-9.07310733685222	2.32161357664455	9.15461105571245
C	-9.17061207380773	1.68570313704394	7.92718857113564
C	-4.42004913670894	0.05252647191844	8.29181205953400
C	-3.27064703869301	0.51744301071705	7.64932822761897

C	-1.98225386143127	0.51433634570025	8.25984564768093
C	-1.86479628546693	-0.00746700293682	9.54263401171096
C	-2.98775730621363	-0.54200541398336	10.23140988734712
C	-4.28611080731835	-0.52948882767741	9.60184451051460
C	-5.37648661353630	-1.14172031904344	10.28616005306562
C	-5.20192115549935	-1.71192534738388	11.53869374596395
C	-3.93034586686298	-1.69522089187395	12.17151924882700
C	-2.84465980037880	-1.12511436786304	11.52538913236350
O	-3.36754724828711	0.99235188568585	6.34448522793675
C	-0.81123042713601	1.03033797787047	7.51298813090507
C	-0.76785038690335	2.32862376940041	7.01115621888415
C	0.34210096654032	2.84474959500250	6.28253626197707
C	1.48956818653330	1.98124970238672	6.08538279535736
C	1.42201778175664	0.64416501705332	6.56818614227084
C	0.31325909824223	0.19804998058850	7.27105731412728
C	2.62466752234036	2.49790017212358	5.40019095647540
C	2.62272371722416	3.78570450801543	4.88248293348555
C	1.49436410618790	4.62542335439800	5.05496149565600
C	0.38708700855618	4.16945587779078	5.75765984272216
F	-1.82065805984040	3.13139119706993	7.23477353387774
F	2.42470405310045	-0.21660140514601	6.33635024863652
F	0.28707684192416	-1.06906744053352	7.70416368893651
F	-0.64475248362826	5.01016686526392	5.91164152294397
F	1.52008646136198	5.85983211056584	4.54849012357121
F	3.67740381955627	4.24814993763542	4.20854658202993
F	3.70966707729509	1.74188416469781	5.19943345078502
C	-7.10659250265013	-0.75617376669097	4.12149883537088
C	-6.84584738419026	-2.11189298776066	3.80077650030074
C	-6.86765424474167	-2.58910662888184	2.49960045979760
C	-7.15929323784340	-1.72121234166476	1.41343503486499
C	-7.49051904400263	-0.34013196481493	1.70626132757678

C	-7.44634651152944	0.08635052065530	3.06423883073158
C	-7.83930240887928	0.52020396272728	0.62400799558381
C	-7.84693193361685	0.05510620481164	-0.68266367663246
C	-7.46817057297695	-1.27937323671669	-0.97257956855189
C	-7.12564618008129	-2.14876050175580	0.05521536956420
F	-6.57060204759863	-2.96242247877411	4.79266438838444
F	-6.61002141376471	-3.88532168089053	2.28622135780182
F	-7.70768995967040	1.38140537372211	3.33283144580817
F	-8.14934048927567	1.80815812186513	0.83063178392447
F	-8.18280219754551	0.86479899574952	-1.69048583617523
F	-7.43921838486639	-1.67925920994202	-2.24543577854006
F	-6.77046980229204	-3.39709248417939	-0.26460037921488
O	-1.02086713159821	-3.31820385316267	4.38903656688734
C	-0.28678697672595	-3.59349173829522	5.53919757166126
C	1.09003182453512	-3.35879066325359	5.52497471588304
C	1.82770170134759	-3.53536246503458	6.74701722451152
C	1.13603222089054	-4.03715183434301	7.90939587134849
C	-0.25421762240816	-4.32417800055919	7.83617556883381
C	-0.98778992865325	-4.09951534987919	6.67433868028145
C	3.20336830913714	-3.18422598950558	6.87318380656454
C	3.87348023063490	-3.35481712988257	8.07613390716386
C	3.20207200427359	-3.88492473202958	9.21071520050307
C	1.85818557467041	-4.21419245870317	9.12754006077736
C	1.71934801793136	-2.93325800291992	4.24553872288336
C	1.21010799941018	-1.83164619270973	3.55636564936949
C	1.68803065363740	-1.42588508325978	2.27592022232071
C	2.73687293116000	-2.13079049133038	1.70407396441775
C	3.30245406041546	-3.26378156888878	2.35237533973302
C	2.77267126704492	-3.69860470235094	3.62335457901188
C	4.35657409190716	-4.00546355547124	1.74060288459304
C	4.85532665181805	-5.15407999576693	2.33440858218486

C	4.30055352999586	-5.61321508709691	3.55846995492400
C	3.28381543001035	-4.90660073314450	4.18542466240827
C	1.00604262054858	-0.31167032499806	1.57234377542026
C	1.10527799029055	1.01538034082023	2.05186330821443
C	0.42430387446294	2.07014267289518	1.46463478471850
C	-0.41786368563170	1.84550002221501	0.33939857050411
C	-0.55198816394584	0.49321018402599	-0.16614864020411
C	0.19042085189475	-0.54198273585258	0.47108487999429
C	-1.12441762107847	2.88906330291318	-0.31996439030259
C	-1.95115612925347	2.62283655654371	-1.40197831640655
C	-2.11450721291062	1.29608040793766	-1.87020243590379
C	-1.43721130936366	0.25103485739864	-1.25688319266665
F	-1.63562164394198	-0.98422573189255	-1.72602334229243
F	-1.04318703304404	4.15706359879065	0.11152258743362
F	-2.61619402490993	3.61389051172372	-2.00174139763777
F	-2.94517173451180	1.06105239023016	-2.88933232959737
F	0.57945101332789	3.30340290820972	1.97718700377694
F	1.90063133189954	1.25240985178446	3.10230440088935
F	0.08398340794662	-1.79486930570350	0.01313608613868
O	0.16473526744726	-1.08808318025364	4.11434085975911
C	-2.44593294856034	-4.34944886081472	6.60671029685971
C	-3.03667291422836	-5.07271712197507	5.56937907865758
C	-4.43826094763639	-5.31219504766538	5.48456035605013
C	-5.27413293599291	-4.88518732939590	6.58641565310887
C	-4.66918579563723	-4.14677168358817	7.64104388621196
C	-3.30994165442223	-3.87467364571803	7.63300757833501
C	-6.66191821998752	-5.19688477858518	6.55597997974087
C	-7.21941990256449	-5.85029973438924	5.46472063413479
C	-6.41326033678398	-6.21585207088440	4.35858630965803
C	-5.05067107046547	-5.94965255967398	4.36714622243656
F	-2.24683631991708	-5.60857049946998	4.62944014739071

F	-5.41251946352557	-3.68031437886217	8.65594311407427
F	-2.79851498193267	-3.14154158209299	8.63359096523502
F	-7.47298781819496	-4.83799796450911	7.55958508217869
F	-8.52742107398431	-6.11619807360567	5.42831986463372
F	-6.98765534338856	-6.79767735183089	3.30351265482594
F	-4.33786069421871	-6.29313735785059	3.28895437081210
O	-3.91651975575090	0.56850133449750	3.89058595587696
O	-4.09267162983087	-2.75338062352592	3.12717707438639
F	-3.93710810095068	-4.43845561579819	0.72812799377887
F	-2.14530613496964	-3.48867490940269	-0.08340265440707
H	-5.38962133964820	1.53526924173945	1.90738693328030
H	-3.36367953588057	3.66017254462179	1.02257941460778
H	3.72696070430855	-2.75546355250156	6.00803585466643
H	4.93338962008594	-3.06905800892150	8.15390299792221
H	3.74784598191112	-4.02002402203488	10.15664246397260
H	1.32007359582408	-4.60670407007425	10.00422516895413
H	-0.75949802035133	-4.71538108653318	8.73081462336588
H	2.85766041313787	-5.28430515401303	5.12414304262801
H	4.75637563169595	-3.65129064169484	0.77791433595311
H	3.12309047709140	-1.82689907865768	0.71983741792959
H	-6.35882144437709	-1.18327382173747	9.79694886917135
H	-6.05565530039114	-2.19048562714374	12.04180406493290
H	-3.80752367638621	-2.14813285237652	13.16685502544983
H	-1.84872282644826	-1.12293787673432	11.99472678764520
H	-0.88119042356017	-0.02373317828451	10.03483400009011
H	-5.81125480506634	1.58289306298227	9.93846544646524
H	-7.76383182053110	2.81641051845828	10.83392185489301
H	-9.93477654365989	2.86881704441822	9.56552138800459
H	-10.10377788105887	1.72743182314059	7.34440136026367
H	-9.06305728018855	0.48379678626330	5.51594446697224
H	5.66621648880308	-5.72051397591720	1.85234989094665

H	4.67492385981436	-6.54298332139995	4.01284943455724
H	-5.04074935021255	3.12348930846542	-3.06653596776204
H	-6.75086569048816	2.84060866138952	-2.64553582637584
H	-5.60963840686104	1.45215988008526	-2.73176869932672
H	-6.53272515388362	4.52274531616843	-0.79057302118953
H	-4.78589862181902	4.76953723512233	-0.92480359535284
H	-6.39173288988863	3.83503696530421	1.53931574393091
H	-5.33741302768883	5.26689332357375	1.40275925520713
H	-4.82674070496282	0.69932523199077	-0.59100327751204
H	-3.53084935044302	0.12533598744097	1.22561280019802
H	-1.84809426744567	3.85223419906747	2.75467437468590
H	-2.43271186590121	3.92449993392826	4.54101350745535
H	-4.72186473026420	3.49203680122253	5.10076819311162
H	-5.75601348279280	4.41287340072803	3.95333655420684
H	-5.73087487157383	2.63607696370412	3.89581556742433
H	-3.45341397217429	0.90929291180316	2.65634005699107

D(minor)

172

C	6.41253794885561	0.87635346195575	2.96717206043582
C	6.29276759511380	-0.17437059154485	2.06683798457797
C	5.15531812390680	-1.02962946199249	2.09161010555436
C	4.14364835115480	-0.81720689717816	3.10524011922184
C	4.29434957717280	0.27976558955992	4.00328335082234
C	5.40059580586765	1.11564135646603	3.92999333600417
C	3.04105910441707	-1.71820329517787	3.15666233801671
C	2.82307519340864	-2.71791427122029	2.20584411929369
C	3.81551780813346	-2.87009740616850	1.19828956303879
C	4.94874987201842	-2.07149341568979	1.14578195272443
C	1.63088766826668	-3.59780988539182	2.24353890104183

C	0.32982926727007	-3.02650035287209	2.40022066064477
C	-0.83333066785416	-3.78813965621595	2.53140643583484
C	-0.73653308853234	-5.21339398689907	2.34453895994412
C	0.56460245116401	-5.80631149410472	2.15114615822694
C	1.72239480989481	-4.98142626059187	2.13039897613162
C	0.66798106173478	-7.21706494987460	1.96310614285351
C	-0.46471388422045	-8.01565974335914	1.93684366780317
C	-1.74866991589647	-7.42692031293795	2.08795059593499
C	-1.88166935950835	-6.06005294227223	2.28742754893730
O	0.24860377658143	-1.64734657811986	2.46931497367531
P	-0.44519940618587	-0.78177032792983	1.25721618017232
N	0.33436443359174	-0.97097744893315	-0.11984038800957
P	1.04896633577325	-0.13013165049305	-1.24926541649440
N	0.65831248170598	1.36921331302898	-1.69882832439309
S	-0.70385799090157	2.12430211641412	-1.95749680767027
C	-1.36748591670767	1.47808753611755	-3.60523937797116
C	-2.11976333919261	-3.11621958519466	2.86817565625728
C	-2.59340147886767	-2.05534804342713	2.09192867496987
C	-3.83709015307088	-1.39392441525362	2.35159666192424
C	-4.59295295655634	-1.81862724354086	3.43784213708993
C	-4.13658053059260	-2.84957005769935	4.30269065563542
C	-2.87434540416062	-3.49685583497779	4.03878239847526
C	-4.89738031302512	-3.23362405028256	5.44742088121060
C	-4.42397684491557	-4.19577437718679	6.32527806877645
C	-3.15910240508185	-4.79911342146521	6.09480342046382
C	-2.40331158646058	-4.45885869065051	4.98140943367756
F	3.65659531390224	-3.81784039771404	0.26349407171404
F	5.84420786058600	-2.28769144708898	0.17057406906310
F	2.19006468057224	-1.61362868022921	4.18561786359896
O	-1.84669869663772	-1.61758919180544	1.00363396554905
C	-4.26472077007862	-0.25767300682969	1.50115000315997

C	-4.30461633818478	-0.37713965185901	0.08632768164153
C	-4.56008804851499	0.69664549997381	-0.74934449862950
C	-4.80966757908410	1.98900421721470	-0.20747181239646
C	-4.87363702080942	2.12878503099978	1.23334052823721
C	-4.60124979510715	0.98385516948756	2.03660699161554
C	-5.10064689158187	3.42343610078536	1.78100530333282
C	-5.20440593403387	4.53831943943471	0.96281475459210
C	-5.10538142592630	4.40654848446844	-0.44420752038334
C	-4.92087131425974	3.15441634295899	-1.01803184525564
F	-4.09093880102102	-1.57841233851041	-0.46302028531240
F	-4.54456675819451	0.49283160091502	-2.07391752919762
F	-4.59491431368102	1.12707334700738	3.37339711712402
F	-4.82497491742088	3.07721738614284	-2.34917874590834
F	-5.15340077393353	3.61947167066597	3.10743338406517
F	-5.37217239641620	5.75391071114429	1.49379091440378
F	-5.16480018774187	5.50352812880579	-1.20277692333322
F	7.25912785048572	-0.33703725582204	1.15499352057962
F	3.36382710170888	0.55195257044236	4.92736281996745
F	5.52575972683946	2.15417437655755	4.75842963468955
F	7.46795068256779	1.69198689867032	2.92225690097737
O	-0.47476260157977	3.57246324823242	-2.12791362009142
O	-1.83179484119132	1.72449508395035	-1.02873335512983
O	1.08120736949730	-1.00722597900253	-2.64706078279722
C	1.84082919679944	-2.17411270310165	-2.67077690814521
C	3.23334284116944	-2.08015697770434	-2.74323445057754
C	4.00486910895731	-3.29470270109960	-2.69042742356305
C	3.31470655351971	-4.56087102887975	-2.63734661385265
C	1.89367650982712	-4.58885035221427	-2.61875134843230
C	1.14484321373154	-3.41808692775998	-2.62986472099154
C	4.07238911947297	-5.76770052302911	-2.57199713902010
C	5.45765434918441	-5.73578862084862	-2.53894754339229

C	6.13727923535199	-4.48867716899206	-2.55957357257597
C	5.42921775281524	-3.29823938354792	-2.63418795469943
C	3.85338703526526	-0.73187217161388	-2.85820907386362
C	3.52475230713316	0.25704273961328	-1.92758788380095
C	4.00107154652383	1.59962514634886	-2.01754963003624
C	4.87650001792449	1.91796067920844	-3.04864550559999
C	5.24757658280302	0.95997296009884	-4.03123321780356
C	4.70433477730673	-0.37556008124875	-3.96669508090827
C	6.10762966923021	1.31615977670749	-5.11302233598033
C	6.39767311466194	0.40799577009733	-6.11880308898271
C	5.82014592217204	-0.88923773799518	-6.08642758973379
C	4.99310477593194	-1.27113263503582	-5.03944976221816
C	-0.33634317529709	-3.43493224205211	-2.58215674620924
C	-1.11248231820614	-2.83694876602742	-3.57129741292263
C	-2.53650544956601	-2.81690007188475	-3.53513521144782
C	-3.20098631987012	-3.48474484651841	-2.43434283582805
C	-2.39998550889154	-4.07501246159535	-1.41639733254701
C	-1.01633745488648	-4.05741854495835	-1.50376711744144
C	-4.62399927537038	-3.48656905644675	-2.39362493191105
C	-5.36624924062283	-2.82424966906557	-3.36145420001942
C	-4.71786543513989	-2.15550578525377	-4.42986489655148
C	-3.33250387829189	-2.16064654002832	-4.51966046923192
F	-0.48350785023623	-2.25966503393516	-4.60474224521287
F	-2.96822984942310	-4.64022683466798	-0.34018322888796
F	-0.29584984254625	-4.62370692988478	-0.52657183971206
O	2.65642327528548	-0.06076047526424	-0.88083654017682
C	3.49544921373691	2.64139228056484	-1.09054256205863
C	2.78988624308908	3.74343542528845	-1.56928996659458
C	2.24891163402921	4.74801259442494	-0.71685499449253
C	2.46512009141173	4.62743225901482	0.71212368190900
C	3.18526702570261	3.49724537738342	1.19240560312467

C	3.66846149038152	2.54125059524397	0.31166819185331
C	1.95841269669520	5.64839627475468	1.56532025819819
C	1.22283457935116	6.70808675291592	1.05289886332949
C	0.99348634512582	6.81531705175317	-0.34049003768154
C	1.49688783022176	5.85602359958521	-1.20798188495417
F	2.59048050954535	3.84429126202401	-2.88828170295678
F	3.41766113101667	3.33280058409430	2.50649434983034
F	4.34534569976924	1.49659388321268	0.80552674687339
F	1.26000314538157	6.00937851455922	-2.51122270235356
F	0.27659582417216	7.84384518033312	-0.80238875817714
F	0.71309858439740	7.63733072818664	1.86652450947105
F	2.15047116517418	5.59780984158952	2.89348626138757
F	-5.28278752820142	-4.09467868778083	-1.40037467885079
F	-2.76894431484213	-1.52230286185682	-5.55135586937548
F	-5.45851869593016	-1.52676034904000	-5.34541459591799
F	-6.69960544320644	-2.80586199535300	-3.30132049173990
O	-0.62738589111322	0.58538595920045	1.89988362361810
H	5.96611909841645	-2.34067857360893	-2.62331604581582
H	7.23611357423461	-4.46477186337827	-2.50509170744566
H	6.03227769182160	-6.67258531589083	-2.48299550111897
H	3.53084578177005	-6.72559878225138	-2.53640637023656
H	1.38043746820636	-5.56121630070510	-2.58407615518819
H	4.54493210457863	-2.27348149129497	-5.03762037861455
H	6.52283150965212	2.33547476542122	-5.13868677392352
H	5.26599830563750	2.94329788530961	-3.13157906524982
H	-2.88084552362911	-5.61407487461046	2.38105425980673
H	-2.64811572649233	-8.05901405992323	2.03731666123991
H	-0.37379082301056	-9.10171980500515	1.78486556806457
H	1.66852033811958	-7.65553612711031	1.82534572085791
H	2.70701957354423	-5.45571877463092	2.01044544478664
H	-1.42184667572888	-4.92627536562039	4.82683139243228

H	-2.76952509407188	-5.53970770321099	6.80958281144504
H	-5.01600037883486	-4.48085281882852	7.20787118755043
H	-5.86411930648743	-2.73748214593374	5.62490618878349
H	-5.55699997555088	-1.33477918496630	3.65098375201267
H	7.05797079154858	0.69453299290774	-6.95104095126351
H	6.02439791314751	-1.59798183025718	-6.90315766577058
F	-1.67682128365385	0.18059973948755	-3.46173847047972
F	-2.46365582948223	2.16508706595218	-3.93148044373482
F	-0.44347680026583	1.61662724663133	-4.55572515537063
H	0.96439270637288	2.02000167879318	4.79895229436275
C	-2.16280457528831	6.09554014725764	1.40391821666521
C	-2.63203241848067	7.20474003302469	0.50706919400637
H	-1.94611504545494	8.07464089166520	0.57918589442926
H	-3.63595534395567	7.55837129156719	0.82159602656469
H	-2.68792228197367	6.88767410668594	-0.55164653903992
C	-2.14730487170602	6.37402363884132	2.88989889972442
C	-1.76605427922579	4.89786098910691	0.91378032683468
C	-1.27391278957376	5.39236907187023	3.67661618202089
H	-3.20038972677340	6.33426871879262	3.25319644485403
H	-1.81228770534608	7.41869104148639	3.06184212439445
C	-1.58393965138053	3.94167758480276	3.26524708947217
H	-1.44425937116863	5.50301625618412	4.76624826300287
H	-0.20387789791316	5.62063120706403	3.49648374367901
C	-1.27572445025980	3.77179321054725	1.77183326107234
H	-2.68115002927167	3.80381678339300	3.36105892401268
C	-0.91285212749919	2.88424833092086	4.12976105484998
O	-1.94887863746091	2.52810466793158	1.34556080809833
H	-0.18711678319420	3.60983383235109	1.59968087823079
H	-1.74804437685816	4.72428505858283	-0.17608981501084
C	-1.66889092606124	2.04693081515424	4.87051434990633
C	0.59016738263874	2.79231375744315	4.10066959334300

H	-2.76846486618602	2.10093079420841	4.85145907585157
H	-1.20712584546786	1.27006967899998	5.50104026715309
H	-1.38695799953622	1.63399440178987	1.66676177630133
H	0.95393714780234	2.51009322278049	3.09032094103167
H	1.07479259217124	3.75824551935580	4.34956476117467
H	-1.92512981010816	2.38228343550433	0.32111225737288

C_2(major)

172

C	-1.51921008207641	-3.60270503539501	0.75516295457506
C	-1.86275854492962	-4.51902146759496	-0.38175870619130
C	-2.42713162565280	-5.73835225654058	-0.22037255196633
C	-2.84448725652227	-6.23523953305204	1.14659149550689
C	-2.25377553920428	-5.42277606251575	2.30370884178901
C	-2.33189988578028	-3.91059791043273	2.03429180978075
C	-2.66118364125842	-6.66838423042691	-1.37714862862170
C	-1.90244818747104	-3.03565774711952	3.20298833435895
C	-2.60963666027637	-1.93351263978521	3.53717015269925
O	-1.72898380780810	-2.22070248748583	0.37499593017598
C	-0.64213153704374	-3.40853037291717	3.93680706886942
O	0.44895027515540	-1.44573126563738	1.60498395540421
P	0.93627034087627	-0.02612059664439	1.48239539516046
O	2.54916801622917	0.10967870266939	1.12381483196059
C	3.44981906791414	-0.16660619614383	2.15016821689892
C	3.67275900522779	0.79605666526326	3.14039664894369
C	4.58161415081224	0.47637489390411	4.21259763916340
C	5.27433176315666	-0.79040737826877	4.19136118711404
C	4.99070767453120	-1.73154802768258	3.16285122231361
C	4.07022777529141	-1.45278170670558	2.16134909623177
C	4.78903794517772	1.33714424061461	5.33192578580216

C	5.67741251648764	0.99560132508888	6.34188621675351
C	6.39907155649033	-0.22660371243439	6.29019536113940
C	6.19148573629640	-1.10501998825047	5.23842874603078
C	2.87356427346862	2.05292450082797	3.11999001451658
C	1.48248136320559	1.94903106599736	3.05798988981618
C	0.60677321600868	3.07395506765845	3.05530990367612
C	1.17054727264614	4.34278151624721	3.08626600752837
C	2.58225551597480	4.52129354998684	3.11015769161247
C	3.45465974778769	3.37038332247447	3.12420466998708
C	3.15341257454496	5.82842122235835	3.08887179391132
C	4.52823749250040	6.00415821921556	3.06120126429585
C	5.38575935456117	4.87222579624860	3.04478207337862
C	4.86221482786279	3.58803043749769	3.07621647451629
C	-0.85789227505687	2.84221331587744	3.01877782682853
C	-1.66511291791540	3.33659752486370	1.99803298308886
C	-3.07384417007988	3.11891900117276	1.94874392392782
C	-3.68822941652479	2.34560295232829	3.00964843732550
C	-2.85024342087927	1.81796993746955	4.03114163964765
C	-1.48654240449970	2.06709788088176	4.03166114365434
C	-3.90870143834791	3.62273613152533	0.91166256714074
C	-5.28062379842702	3.41514531791566	0.92264771988894
C	-5.87984920132070	2.67709749150811	1.97253502543346
C	-5.09907815228177	2.15700367905688	2.99596071375581
F	-7.19869421544865	2.47593386155399	1.95443317487695
F	-5.70912456979252	1.46064138722769	3.96128619958909
F	-3.39270720736950	4.30737171084359	-0.11720162366643
F	-6.04734160077359	3.88306635248810	-0.06601233274036
F	-1.08278104860257	4.03445057150363	1.00897687326755
F	-0.74641963817439	1.56656668396535	5.02791214679641
F	-3.36234126365658	1.06961564155204	5.01998366463908
O	0.89496699098717	0.69200986450242	2.96490673037936

C	3.63521853318520	-2.48628720344170	1.18541713309423
C	3.87695820848444	-2.35323784973998	-0.20366219912374
C	3.42297983907320	-3.27907904154399	-1.13103556000042
C	2.63530536605491	-4.39004207090943	-0.71672734058844
C	2.37003355886173	-4.55752150945187	0.69826231522206
C	2.91280029539706	-3.59995558611768	1.60291198095920
C	1.55708079606766	-5.65216835892593	1.11435277745797
C	0.98257396912074	-6.51043331421692	0.18814980223554
C	1.23249260380129	-6.33746655496069	-1.19561773835253
C	2.05565570335676	-5.31034754614969	-1.63698003890943
F	4.57905173558266	-1.29655156212541	-0.63102516164022
F	3.72879211788576	-3.09715300231192	-2.42278084668531
F	2.67028183847844	-3.74392094056809	2.91628252980589
F	1.28621172874474	-5.86014724527650	2.41197735983191
F	0.18175009457750	-7.50458263979496	0.58560346693307
F	0.65529427214941	-7.16976439833663	-2.06589882363385
F	2.26626892928574	-5.19302832567377	-2.95069952129865
N	0.19949171058247	0.98748644106026	0.45070384130335
P	-0.48126191732251	0.73499724719225	-0.93689941031758
O	-1.98984366323502	1.36365461617042	-0.87955753367235
C	-2.61569467093716	1.66663180603223	-2.09753304941615
C	-2.18292094169077	2.77918591232602	-2.82427188285149
C	-2.80313974900721	3.05053522801108	-4.09762091267254
C	-3.89552601147733	2.21572947935900	-4.53484775697536
C	-4.28292566037682	1.10048337955197	-3.74679703415089
C	-3.64423575158960	0.78981291823450	-2.55047485751749
C	-2.35087941699257	4.07870303559926	-4.97826609618814
C	-2.98320047733199	4.30835627241608	-6.19193088648383
C	-4.09556646775891	3.52109911845073	-6.59278773691218
C	-4.53528624779332	2.48811214599983	-5.78119807395029
C	-1.00779027479271	3.57241818062998	-2.36514827721858

C	0.19899221040359	2.90639014345777	-2.14914274992720
C	1.43712751416612	3.56361171304244	-1.88802061947327
C	1.40253299986225	4.94693050366847	-1.73332585772629
C	0.19013698342180	5.68091959834486	-1.84385275918552
C	-1.03683107508187	4.99926570723298	-2.17977787391989
C	0.17106250823613	7.09413680531067	-1.64379343023659
C	-1.00876222506493	7.81168500500960	-1.76012053752070
C	-2.21912359661355	7.13869171215450	-2.07776362173695
C	-2.23340952378329	5.76698871458478	-2.28411376904167
O	0.20494498488152	1.50667389760046	-2.19985307156308
C	2.70029437011297	2.79441487586044	-1.81389693032084
C	3.05898958814803	1.86659503442985	-2.79330357387206
C	4.26554229081622	1.11146367897172	-2.74919775641738
C	5.20377008298309	1.38241287163173	-1.67998430689174
C	4.84354036217632	2.34422647104037	-0.69517271917061
C	3.63163533965084	3.01403289929790	-0.76157577079180
C	6.42955414222514	0.66067457003611	-1.64166730187860
C	6.70784799609678	-0.31716074095645	-2.58795138897699
C	5.77587723197524	-0.60859757162197	-3.61414379207228
C	4.58440121081376	0.09935391222886	-3.70058816310731
C	-4.00277512202739	-0.46651248882965	-1.84375093191059
C	-4.52232930548870	-0.49743529658035	-0.55455446835687
C	-5.01867108295678	-1.68432727716844	0.05866326561995
C	-4.95763133754938	-2.91990581028355	-0.69387107615629
C	-4.36187467481348	-2.89661516135725	-1.98538580120659
C	-3.90677429148751	-1.70769829838134	-2.53368479429774
C	-5.49933652431219	-4.10150800304848	-0.11610217727126
C	-6.04385795525274	-4.09121695210329	1.16136615734424
C	-6.08797168337754	-2.88848200255839	1.90957643364179
C	-5.59234917571830	-1.71257289124195	1.36132716605670
F	2.24207519262436	1.70161274468360	-3.84275197272995

F	5.67197134077505	2.61177253115719	0.32578752781759
F	3.33285753427937	3.90224421570978	0.19729090932093
F	7.33168888193486	0.87846925729629	-0.67647750296423
F	7.84697630705994	-1.01034290480872	-2.53323491183160
F	6.06126973081906	-1.57091941955929	-4.49398581435586
F	3.74755377962155	-0.20807477722041	-4.69505669241694
F	-4.60789322989362	0.66254386493968	0.11613554413324
F	-4.22818236806411	-4.02187563872562	-2.69912985661144
F	-3.36013961498082	-1.73828338830179	-3.75730889706255
F	-5.49263117145530	-5.26176179119659	-0.78287838146822
F	-6.51536135303021	-5.21550203947447	1.70391898833194
F	-6.59446659657659	-2.90372379060256	3.14197028852818
F	-5.64719132997645	-0.60780254610407	2.11753366515853
N	-0.64492723411064	-0.82954251088451	-1.53969621357707
S	0.52201119151446	-1.55974291756430	-2.50864084737573
O	1.83392758186626	-0.91588917657489	-2.32545298965993
C	-0.07143377460235	-1.11331563937702	-4.28510147353975
F	1.00857923847599	-0.84042247559399	-5.01182076236944
O	0.33122295282911	-3.01280046814740	-2.37270042200574
F	-0.70538759386892	-2.17264418079364	-4.78442419859670
F	-0.88970320151951	-0.06063260406588	-4.27409697256224
H	-3.17938910936357	5.25517091520932	-2.50471973935187
H	-3.15692964176613	7.70946163018642	-2.15323503726717
H	-1.01266644188737	8.90046175965566	-1.60102853011475
H	1.11545245679919	7.60024370803833	-1.39086884937756
H	2.33274152114886	5.49361118033376	-1.52307401684759
H	-1.48450790699731	4.69037720919959	-4.69515465615019
H	-5.37616247369657	1.84831172863283	-6.09025372593546
H	-5.08950860576601	0.44795046225856	-4.11244741927612
H	5.53504340587970	2.72159170285584	3.03395871859071
H	6.47602778230777	5.01339719595672	2.99567945242647

H	4.95668238784508	7.01752247370610	3.03899066157139
H	2.47567677119707	6.69621288406014	3.08396463761549
H	0.51976804435280	5.22970820064924	3.08144674758699
H	4.22891328922683	2.27953134148825	5.39491492198560
H	5.81826471962404	1.67678575711139	7.19471304384976
H	7.10587816080676	-0.48156506829728	7.09404946219934
H	6.72014658096504	-2.06993468751042	5.19915615106116
H	5.48611947393634	-2.71356876058234	3.18615303833710
H	-4.59160988868000	3.72093991583057	-7.55436423033245
H	-2.61285926805590	5.10590016420497	-6.85357368821137
H	-2.02162621342824	-7.57136185083540	-1.27634454664156
H	-2.43229689807936	-6.18742905828488	-2.34703215415603
H	-3.71390505257801	-7.01936169799936	-1.39659303562778
H	-2.56254424908480	-7.30541480106181	1.24719982909951
H	-3.95804793812889	-6.22508895781168	1.19249603583833
H	-1.19212667557760	-5.71283142827496	2.44502248929678
H	-2.77653732772807	-5.66473622752059	3.25190387440788
H	-3.38092432477061	-3.64935708610849	1.78456739042131
H	-0.43168199355568	-3.71037870610959	0.97907661408396
H	-1.55214421090327	-4.18198268504885	-1.38424113598943
H	-0.38316152616080	-2.64404309203194	4.69392125676539
H	0.21876122109241	-3.50091488554488	3.24653271972621
H	-0.74705772251806	-4.38762914096954	4.45048713934247
H	-3.53689338207055	-1.65646771873270	3.01441130308708
H	-2.28469116189071	-1.26782192068904	4.35299927572045
H	-1.12481213893250	-1.74211048623176	1.01583450203098
H	-1.13608700972921	-1.55188252504043	-0.84026078450914

C_2(minor)

172

C	-0.88032598028912	-4.25093780952986	0.01817087099417
C	-0.77565446033904	-4.54992761949150	-1.45283199724749
C	0.21852545394717	-5.26889636654353	-2.01763322565836
C	1.34343119511469	-5.81217897267934	-1.17447634359101
C	0.98914822220018	-5.91093053814113	0.30909033271019
C	0.37525537679811	-4.59654467109464	0.83278073965201
C	0.30849282759747	-5.50794829407858	-3.49686302940676
C	0.12656734936465	-4.66352495842325	2.33953214493055
C	-0.94587909677888	-5.28989083114527	2.86991436412374
O	-1.23700366129292	-2.84136415519262	0.16637113383251
C	1.19524993058586	-4.04595984878735	3.19701880747469
C	-4.25138566803453	-3.89462594159764	0.83389054376342
C	-4.37763672780051	-2.50541376668840	1.21967062781972
C	-4.36376116105252	-1.51399437024907	0.19525475977627
C	-4.15614734808169	-1.81171420559716	-1.15072867729859
C	-4.04003250273456	-3.18854383254557	-1.49149929690029
C	-4.11264762282038	-4.19862196766202	-0.54815501549711
C	-4.22945437447376	-4.88934674553318	1.84917606383927
C	-4.29803987437238	-4.54524643966258	3.19277824715791
C	-4.43535678261843	-3.18834644510308	3.57458175190324
C	-4.48176803685411	-2.19365519360698	2.60524015980822
C	-4.08356430433702	-0.77989411253693	-2.20971997115686
C	-3.24759395819519	0.37451874332771	-2.08679865790338
C	-3.20127500943356	1.37901489795294	-3.05940985831380
C	-3.87959758147987	1.15374027085528	-4.31195237202886
C	-4.71814021634853	-0.00836754697528	-4.45651519587337
C	-4.82238610812635	-0.92832365450552	-3.38162729471151
C	-3.72578954549200	2.02080352363203	-5.43440959444250
C	-4.39945634879380	1.77317642478192	-6.62197924308022
C	-5.26478306680185	0.65289419243721	-6.74576748000126
C	-5.41554797320201	-0.22365701579746	-5.68327939068741

C	-2.48014916541535	2.65476935984399	-2.80778569387849
C	-1.14130223158010	2.63549288681653	-2.42551899740631
C	-0.36580339800336	3.80838329851639	-2.19518618731374
C	-1.00311455905421	5.03686792148221	-2.33718479820562
C	-2.37809798603066	5.13028179499990	-2.68383817949595
C	-3.14419493391297	3.92991728062499	-2.91536464308209
C	-3.02213330843976	6.39955923422245	-2.78367039512327
C	-4.37355969079711	6.48929766587487	-3.07703319388023
C	-5.13623931169919	5.30682466403093	-3.26721558170823
C	-4.53759404449830	4.05831348108122	-3.18944758636116
O	-0.51457892820624	1.39491008889268	-2.27753124695038
P	-0.85293185692998	0.50315866312463	-0.95871084836728
O	-2.48945603417093	0.50022348057357	-0.91428886998788
C	1.06108586223771	3.70743562530314	-1.81444573809542
C	1.97615028248190	2.97435966122560	-2.56938363267452
C	3.35413153889965	2.86199122973921	-2.22969694409877
C	3.83259439860784	3.58368679056804	-1.06845586934794
C	2.90173230009929	4.36575528733927	-0.32894050190596
C	1.56100934677821	4.40138959452747	-0.67883434776898
C	5.20999028741314	3.49344050794172	-0.72251302233095
C	6.08628148929555	2.73516434357207	-1.48830613244713
C	5.61939472858769	2.02702279430690	-2.62310546951752
C	4.27874673111938	2.07977985378189	-2.97958898805542
F	1.54106493277669	2.38072307712481	-3.68862435962199
F	3.30472316224408	5.09122123358992	0.72245993350873
F	0.72341972874312	5.13598533092613	0.06788693419690
F	5.69396127619143	4.11987661338779	0.35887066091618
F	7.37539777533066	2.64333237379810	-1.15383908142743
F	6.48075545713561	1.28214757751766	-3.31940596082465
F	3.88352810110899	1.36145510794360	-4.03474382686596
F	-4.57749521291833	-0.23387703846792	0.53845757204115

F	-3.96637084913931	-5.46689465902632	-0.95835305832817
F	-3.81680791411890	-3.53587679620528	-2.77063273697890
F	-4.06791033967969	-6.18427378817729	1.54016096226589
F	-4.20577339194796	-5.48184829295719	4.13640768761205
F	-4.50448867965750	-2.88081211016256	4.86968397165686
F	-4.60316936863107	-0.92954013745142	3.01950620038809
N	-0.29631077573007	1.08594199461717	0.39307978469531
P	0.45843399798135	0.25578981071132	1.57628344756616
O	0.20255221531788	-1.22149095882600	1.74718168646967
O	0.11412217767590	1.02610912157409	2.99477334651768
C	0.54348873207753	2.34050857204064	3.14968687803015
C	-0.43271370986836	3.37696559948262	3.07005393863256
C	-0.00596458598980	4.69423601196462	3.17986939305575
C	1.36661489083861	5.01700920990070	3.36251211106135
C	2.34132839026408	3.95851562481201	3.46613640795117
C	1.89384359241007	2.59052443396183	3.38466235730637
C	1.79848283506314	6.37426317164149	3.43693943001758
C	3.13957220261274	6.68533130101019	3.59685071610164
C	4.10316591981467	5.64500224845035	3.67118053242777
C	3.71543729503547	4.31492191738244	3.60296586061403
C	-1.86171927606601	3.02813592884264	2.89887348997621
C	-2.61065279314181	3.48002218257735	1.81794135901866
C	-3.98553736548334	3.14934592115032	1.64109076170803
C	-4.61785443880284	2.28072704932316	2.61270037577029
C	-3.85484261456441	1.85960490680749	3.73846944210107
C	-2.52144380194607	2.21996383809418	3.86348542308595
C	-4.76454868561689	3.64084821531539	0.55549825833980
C	-6.06866008096130	3.21019065570798	0.35450967255999
C	-6.67125153348015	2.32060423271091	1.27976179799863
C	-5.96938567434441	1.88687582859556	2.39793790839735
F	-7.92804009891965	1.92241202468728	1.07027289911775

F	-6.59518409141802	1.07390187523829	3.25618152720536
F	-4.25901175678959	4.54404031188245	-0.28968213526179
F	-6.78145968768738	3.64739815736602	-0.68888074781902
F	-1.99921050395334	4.23036940866273	0.88713387762756
F	-1.84115352145907	1.81112435510317	4.94088434179151
F	-4.41242307559669	1.12522788973587	4.70918696555758
C	2.84210268868688	1.45207375289636	3.49222889832663
C	2.94493006713430	0.53868486668831	2.43799220466683
C	3.96542092321909	-0.46567476961647	2.40399392306752
C	4.80662952624370	-0.58991158805561	3.50636214286993
C	4.66184025642030	0.22926681817230	4.65697147056385
C	3.66334058176505	1.26815300954512	4.66045160749930
C	3.50339328173531	2.05001779006445	5.84311883890030
C	4.31062744546948	1.83661947951372	6.95155631879838
C	5.31926234431123	0.83568096210493	6.93283897641726
C	5.48680584270025	0.04501204206588	5.80688651617553
O	2.06544900216823	0.63543701468405	1.36588511317963
C	4.17112889245049	-1.32640001472517	1.21489767634156
C	4.37235250078909	-0.75372909019922	-0.06947637977094
C	4.62077015341039	-1.52521346967107	-1.19381784015467
C	4.66896900683278	-2.94330006580415	-1.10853160721570
C	4.50329154423646	-3.55998549282367	0.19151307900087
C	4.28529172752549	-2.71136454744727	1.31396658437912
C	4.54736819560986	-4.98139112772465	0.28369455035280
C	4.70173884647532	-5.76679231486291	-0.84994067507785
C	4.84139319737052	-5.16228854407497	-2.12454425443728
C	4.81724093742663	-3.77903925737055	-2.25137586289558
F	4.35865916751015	0.57822899579264	-0.18611594697808
F	4.80305357589452	-0.91134742833036	-2.37141069543413
F	4.17562194918310	-3.27394959136342	2.53001450964230
F	4.36980477531973	-5.60727471503899	1.45686924351870

F	4.66462012965036	-7.09883333510890	-0.76503216570660
F	4.94920578953556	-5.94319853562324	-3.20126433597773
F	4.92466813980882	-3.25733353098885	-3.47854235971629
N	-0.37521682835935	-1.03940411403164	-1.43931946554055
S	1.21284850411444	-1.27459331509633	-1.93225501890939
O	1.98098400914319	-0.02373459314716	-1.79273285871141
C	1.00039937803903	-1.55612531333940	-3.79543982887926
F	-0.04531144350870	-2.35659337150548	-4.02136833618322
O	1.69776987889905	-2.54978524238703	-1.38200779978879
F	0.81455304849390	-0.38755279945320	-4.40377531302369
F	2.11865738616703	-2.13595191989885	-4.24326263070213
H	-5.15043835526821	3.15527549218138	-3.30703667633873
H	-6.21685381218865	5.37896827848437	-3.46138238207538
H	-4.86093315348308	7.47342724119400	-3.14598865493491
H	-2.42267827163607	7.30649677800173	-2.61018808555643
H	-0.43567483468198	5.96362861087295	-2.16960966187819
H	-3.05242105259865	2.88490193465250	-5.35618751189776
H	-6.06304157107562	-1.10960387655786	-5.77107001298275
H	-5.48873598463115	-1.79505702203018	-3.49430867761026
H	4.47592178990147	3.52329403569457	3.63036568666051
H	5.17048709047348	5.89538437874312	3.76685538165657
H	3.46177707558724	7.73614238472803	3.65105849311318
H	1.04204573275564	7.17029972388508	3.35788560576676
H	-0.74210893720064	5.50932997847328	3.12053754310695
H	2.71986859385648	2.81952594842676	5.87324459950030
H	4.16377599671869	2.44549506743551	7.85647509313622
H	5.95605170471005	0.68274308340030	7.81709156095731
H	6.25186429822171	-0.74637661149530	5.78328194404519
H	5.60384451883872	-1.34681889374292	3.49324237084600
H	-5.79988223814207	0.47548475590553	-7.69067560056536
H	-4.25720770983309	2.44941460868477	-7.47835666406911

H	-0.53019756072518	-5.04438157099911	-4.05011943771862
H	0.32882820079855	-6.59557407487916	-3.72504627550581
H	1.26035566710973	-5.09045910429198	-3.89357310881477
H	2.20485141894891	-5.12386718820709	-1.31480502556194
H	1.67967248302975	-6.79724326837074	-1.56354696848238
H	1.88962359397803	-6.16094929431480	0.90549677045554
H	0.26116007420588	-6.73526101022439	0.47124768633874
H	1.10969745818431	-3.78733943061565	0.63243486424682
H	-1.73866052509853	-4.82340377853553	0.43219204035681
H	-1.58023535744551	-4.14678880361935	-2.08806883676401
H	2.18391688146320	-4.49653541376430	2.97347481672508
H	0.98776905178390	-4.17516622203807	4.27689191905040
H	1.28511997408658	-2.96214175856691	2.97334619738936
H	-1.71290499416028	-5.77862263864046	2.25048404483527
H	-1.08425549100420	-5.35444219967085	3.96106368878812
H	-0.78626759091132	-2.43106359556684	0.96047981834362
H	-0.77515084068705	-1.90930791924474	-0.81054712900443

Covalent adduct (major)

172

C	-4.89409662562892	3.51078833608691	1.47265529986110
C	-4.94009702770711	2.15051923360399	1.05480272005655
C	-5.23531050475689	1.86122846267897	-0.33333407112729
C	-5.43018604209523	2.94526402938964	-1.23176656056438
C	-5.33534534397519	4.25929580921638	-0.79628403674955
C	-5.08831425348744	4.54428887427871	0.56772208431376
C	-5.20427580684383	0.50596504690826	-0.76251575984383
C	-4.78585904097771	-0.49506194136665	0.09643217665876
C	-4.41668228544528	-0.23604116173125	1.44025270405572
C	-4.54168505779870	1.07301696900535	1.89765551220775

C	-3.90047931586509	-1.33167695231548	2.29504928677928
C	-2.71742980056784	-2.04109165643485	1.92286288966666
C	-2.19698090052777	-3.10152949571286	2.66682571670524
C	-2.83684634419009	-3.43449893937578	3.91833089135265
C	-4.03296276408549	-2.72679687421696	4.30664818781098
C	-4.54316637941632	-1.69683893333492	3.47067390357188
C	-2.30516178557331	-4.40076869776234	4.82378433491466
C	-2.94512957334171	-4.68629630240866	6.02185946184081
C	-4.14787088792061	-4.02127307349742	6.37901294572585
C	-4.67518366176829	-3.05434852502201	5.53794261881280
C	-0.95152787735247	-3.79937992753883	2.24002341031443
C	0.20927250942054	-3.05120622942278	2.04567627266999
C	1.50078114432855	-3.62859376634983	1.85499045608815
C	1.57622165505390	-5.01417524221639	1.75132845567813
C	0.41349536321632	-5.83003685927808	1.82413393042223
C	-0.87377709777430	-5.22653103466012	2.07148254911067
C	0.49742482704927	-7.24456388233740	1.65599775161761
C	-0.63990591325467	-8.03548427632891	1.70786985813811
C	-1.91039437804305	-7.43737075924501	1.92435668980442
C	-2.02468866605227	-6.06634563032898	2.10444938780068
O	0.12950640187285	-1.66683653924815	2.09708034601974
P	-0.64262153501728	-0.81687621896724	0.93281961334794
O	-2.05649621560283	-1.63381234234267	0.75439197990915
C	2.69815140232472	-2.75761693204978	1.80531585924863
C	2.93686389167871	-1.78260967597334	2.77605458919112
C	4.05223864255269	-0.89884092476371	2.73888105418406
C	5.03980706305626	-1.08732194118890	1.69806439010294
C	4.82004354802736	-2.11696066408980	0.74054719845481
C	3.67831944399823	-2.90287128645782	0.78532376356791
C	6.17424908783152	-0.22927955896272	1.66512574481993
C	6.31768579370567	0.79377185924143	2.59338218448244

C	5.33677781887174	0.99775579369879	3.59476752923179
C	4.22703493344481	0.16678023610364	3.66833909855057
F	2.09279206572989	-1.69357960133531	3.81070547704455
F	5.71330482812076	-2.33516414301096	-0.23700306082889
F	3.50339779049799	-3.83697950858368	-0.16366279420570
F	7.11731132088693	-0.36504441447185	0.72271411620771
F	7.37223399350716	1.61123876534451	2.54450360285937
F	5.48834856752537	2.00890699184803	4.45241069134228
F	3.32533984397622	0.41040605603606	4.62252445898281
F	-4.19890198227880	1.33404749219887	3.16792313702800
F	-5.51310911686606	0.17440029906059	-2.03104014058431
F	-4.72056343486574	-1.75735451077870	-0.35294283589447
F	-5.61439426202512	2.72792952525328	-2.54439323090523
F	-5.44530576558378	5.26999622796337	-1.66371565035320
F	-4.98294432708898	5.81822604207321	0.95693074767400
F	-4.59723431405895	3.83780203711332	2.73498960295857
N	0.08590993636560	-0.98452127013411	-0.48250294766436
P	0.71153095521292	-0.02829034943213	-1.56614642915406
O	0.06040227102424	1.36687190791302	-1.92932670706935
O	0.82968796923477	-0.80011643966199	-3.00024372911807
C	1.71862358815373	-1.87638264822212	-3.07175761791905
C	1.16472043791966	-3.18943099364728	-3.05796499074472
C	2.04589459231227	-4.26160736443467	-3.11167327264390
C	3.45306202738688	-4.05993665734270	-3.16909417999353
C	3.99305466217166	-2.72017214188393	-3.17009177900881
C	3.08222874339666	-1.60271046197516	-3.15079776883494
C	4.34640420486070	-5.17137825534727	-3.19084571734510
C	5.71903402209374	-4.97982101133038	-3.19966181733463
C	6.25012048818211	-3.66348759071709	-3.16960803272054
C	5.40880908617492	-2.56053137718127	-3.15504201921923
C	-0.30470664520643	-3.34691035035733	-2.94460317640992

C	-1.17432156394046	-2.75224485975248	-3.85496123736338
C	-2.58974036123688	-2.76292203944344	-3.70330083015262
C	-3.14889603735190	-3.50301764624061	-2.59045524775450
C	-2.25522288953853	-4.11896589668084	-1.66904986392797
C	-0.88181503146336	-4.04066647684290	-1.84827774703465
C	-3.47322424899341	-2.05108827593188	-4.56604592955730
C	-4.84593823362246	-2.07647304075099	-4.35998796864120
C	-5.39396160275763	-2.83920879776710	-3.29855864663432
C	-4.56348966256207	-3.55047738028744	-2.44320526740683
F	-6.71544466695680	-2.84932848723213	-3.12446967948946
F	-5.12901907121293	-4.24374725594402	-1.45020152529362
F	-3.00122883700685	-1.29838244934666	-5.56843774924065
F	-5.66552634780221	-1.37872308043413	-5.14788591196909
F	-0.64292813977606	-2.11316373161136	-4.91144734603069
F	-0.07522586995811	-4.60254291006783	-0.93891457617990
F	-2.72641302360000	-4.76008323162163	-0.59098999413542
C	3.53497626827737	-0.18272171133640	-3.19009425409208
C	3.13418858398953	0.71675507563207	-2.19685897461291
C	3.56287791102341	2.07708485927199	-2.14832068334808
C	4.39724605298013	2.53354205490574	-3.16206986997699
C	4.78426651366296	1.69430241126285	-4.24207836846120
C	4.33756996966503	0.32268501597003	-4.27703043368108
C	4.66786592933798	-0.46523964068795	-5.41969548390152
C	5.43801694253980	0.05814567084418	-6.44872058854842
C	5.91738274140600	1.39387868376351	-6.38900778595980
C	5.58923255489391	2.19662068108449	-5.30805091744233
O	2.26513745253562	0.28706335775732	-1.18232771959714
C	3.11938827155966	2.97273149693425	-1.05095981828879
C	3.40317317600969	2.66214026591631	0.30554441342666
C	3.01887305767915	3.48889912952234	1.34869592957386
C	2.28381668233946	4.68054470037841	1.09762207262183

C	1.98969981106414	5.03753796998597	-0.27583241571968
C	2.44696646419349	4.16502167311832	-1.30492803498060
C	1.26770830055662	6.24042136010615	-0.52858068688046
C	0.83296794238214	7.04345788692715	0.51606828925572
C	1.09403196917229	6.67607004133492	1.85918306294242
C	1.80333158940871	5.51637722874285	2.14416214713449
F	4.09180980556677	1.54694005464412	0.57604667716710
F	3.35951854610322	3.15303948650775	2.59821218634494
F	2.19962375841478	4.49220675468223	-2.58823691002314
F	0.97310231337961	6.62366476169872	-1.77840436961413
F	0.15488941381549	8.16922873082290	0.27539550087679
F	0.64509723887558	7.45885710149703	2.84166936944449
F	2.01958078046433	5.21026431931944	3.42631570327333
N	-0.94014680009778	0.65387781136507	1.53652318229082
S	0.20415099226756	1.49943590985030	2.33888169730445
O	1.56953942985373	0.94936979811495	2.15646537892467
C	-0.26559493917148	1.19687550464505	4.14744467441584
F	0.63483875474855	1.80824695100817	4.92540051134685
O	-0.04269952336880	2.94423131993698	2.14113954802268
F	-1.47942215244688	1.71338074606141	4.37337290594979
F	-0.28273286981458	-0.10959483160388	4.42741881598156
H	-0.48371132873462	3.14079366185235	-0.49249617128600
C	-1.54778170223643	3.31648243074287	-0.76998451785630
C	-1.90209211707823	4.74725756051764	-0.43505054242180
C	-2.12238803803260	5.73624214085890	-1.32920446396275
C	-1.93161641461739	5.47999096984566	-2.80473381088009
C	-1.15941970941396	4.18986957275180	-3.09544747748390
C	-1.72395790301054	3.01937016541109	-2.27759657700207
H	-2.82255813056219	2.99589994878887	-2.45615382690431
C	-2.58877996725456	7.10819742381663	-0.93137809875518
H	-3.01318890201248	-5.61052540054715	2.25356380091626

H	-2.81345027534990	-8.06601979444011	1.94238329250477
H	-0.56340948486777	-9.12472737704140	1.57157919153989
H	1.48632805458902	-7.69336434052241	1.47468020307733
H	2.55338571499663	-5.49668530476978	1.60564343395488
H	-1.36777108495596	-4.91432233332270	4.57338924840788
H	-5.59308971063398	-2.51128763315293	5.81085794910205
H	-5.46002788730502	-1.16881294139421	3.77099329746376
H	5.83384343587379	-1.54964437204201	-3.10098177975806
H	7.34015943208353	-3.51530348449352	-3.14498355475529
H	6.39879688717405	-5.84496580382497	-3.21354671209299
H	3.92007747887613	-6.18634801694097	-3.19120268258586
H	1.65495322432599	-5.28959236555023	-3.10076496053493
H	4.29488641709079	-1.49590692564855	-5.48754803706496
H	5.67393919064545	-0.56772980718428	-7.32257475215632
H	6.53406734135221	1.79249446790150	-7.20843717596674
H	5.93213848902427	3.24151116090612	-5.25832289254240
H	4.75320927735655	3.57390859242041	-3.14242576065110
H	-4.64789159408940	-4.26299768826395	7.32888210367073
H	-2.50974000719696	-5.43126510778838	6.70489366542508
H	-3.58440930316862	7.31874039244249	-1.37836101205872
H	-1.89435677665006	7.88904382098303	-1.30566285419271
H	-2.67607717436423	7.21289636418562	0.16664350325981
H	-1.40842424051595	6.34715189240275	-3.26236611393396
H	-2.93486161993411	5.44888964693038	-3.29227849618206
H	-0.08749728216141	4.33338346838439	-2.85322086492020
H	-1.21356093195902	3.97695384695845	-4.17975287250624
C	-1.24334128765947	1.60103050627976	-2.69871565355699
O	-2.38403186126006	2.46794472857414	0.00963046123769
H	-2.02877807133814	4.93449228535740	0.64513087212636
H	-1.83407688302346	1.89224931498885	0.59657793588991
C	-2.25470922693315	0.53211799108581	-2.30058754381730

C	-0.88595023580123	1.48468825535689	-4.17693656487524
H	-3.17609407679835	0.68432243005327	-2.89348379365942
H	-1.88569232201469	-0.48704880384800	-2.52032172285230
H	-2.51849678378029	0.62645295139309	-1.23474590425864
H	-0.68791385297876	0.43302844711113	-4.45131900416307
H	-1.74330954593913	1.83810330838831	-4.78315137806632
H	0.00064645663233	2.09484102381793	-4.43129997018059

Covalent adduct (minor)

172

C	6.11527431857980	1.19296821586626	3.22299896134458
C	5.97152942427839	0.19659077519217	2.26722003420815
C	4.85887888295775	-0.69018216784537	2.29613511082507
C	3.91803868405329	-0.58676608646458	3.39261566308846
C	4.09175579634952	0.45380779094996	4.35109993667477
C	5.16299915497895	1.33192633136881	4.26383367470293
C	2.85175566621020	-1.52709009398873	3.44664167580985
C	2.61036704863572	-2.46747100249319	2.44849973711235
C	3.52290727281709	-2.51306629331476	1.36270215669200
C	4.61294755118956	-1.65813404800916	1.28138434571896
C	1.41845262486848	-3.34630191107902	2.49029072635101
C	0.12693835079949	-2.75111185029123	2.58545233919761
C	-1.05799012877089	-3.47634426339495	2.67710346538700
C	-0.98109214965541	-4.91213054225624	2.58010768516931
C	0.31707027957232	-5.53564606816556	2.47248575205440
C	1.49121588839886	-4.73264827106797	2.43881192858683
C	0.40179938186709	-6.95605389287913	2.37269382724154
C	-0.74321073357182	-7.73725010091387	2.35800963716391
C	-2.02100928088397	-7.12170919478064	2.43084661349981
C	-2.13751291421587	-5.74337651919356	2.53963148990414

O	0.06034110328450	-1.36068598062457	2.62661999647589
P	-0.49324773304744	-0.54601438710992	1.31883879699950
N	0.54305175705812	-0.67322030555652	0.14392741314116
P	1.12842256067776	0.02187534677989	-1.16619915174115
N	0.63144611365516	1.44501224374732	-1.72163638870502
S	-0.89452477489236	1.92757213194120	-1.80298239922640
C	-1.47145029674032	1.39272593846613	-3.52060701252273
C	-2.34128407024865	-2.74417763263759	2.86395260774388
C	-2.72183919554502	-1.73428147663405	1.97565127050470
C	-3.97333493896961	-1.05108201867216	2.05733100414222
C	-4.81672826395944	-1.35882174476420	3.11831782920548
C	-4.44829162082532	-2.30512689163361	4.11284385824887
C	-3.19327908367717	-3.00764496618246	3.99831952914297
C	-5.29744737512338	-2.56362272508146	5.23019203128163
C	-4.91706890432410	-3.45629657120497	6.21973577661120
C	-3.66227510747976	-4.11584324321160	6.13168825972125
C	-2.82144557617334	-3.89831638463597	5.04914346452277
F	3.30915441530453	-3.39130974041459	0.37485338567283
F	5.41590570332606	-1.74068606929559	0.21173360839180
F	2.02883022067152	-1.50655058651548	4.50975883414142
O	-1.85370096971139	-1.36294538549652	0.93471053204354
C	-4.36905598735072	-0.07282796912212	1.01666709520301
C	-4.41712565198343	-0.46643639117372	-0.34500645433348
C	-4.76603439822283	0.40371992720266	-1.36144779601840
C	-5.08721029997152	1.75963452494859	-1.07184230082465
C	-5.07620499970526	2.18909382614191	0.31172265636030
C	-4.71814472456176	1.24084369171037	1.31387395394386
C	-5.31997718843250	3.56130410663203	0.60512586589347
C	-5.57833007186005	4.47249850259838	-0.40886305016634
C	-5.61481523979881	4.04672424574309	-1.76003102103988
C	-5.38282371246993	2.71599234856846	-2.08421722634163

F	-4.11387573536855	-1.73684171019028	-0.65130607878873
F	-4.76529996357651	-0.05257452998309	-2.62535486206314
F	-4.68826762385000	1.62763634005143	2.59874568462569
F	-5.43360206517086	2.36231194870624	-3.37479849211553
F	-5.27537965573611	4.01993818548385	1.85835729333111
F	-5.77715014658023	5.76452675240071	-0.13044521321975
F	-5.87480555785503	4.94269866321831	-2.71448411785727
F	6.88509025370818	0.11991924281744	1.29449460860588
F	3.20328126461230	0.63790224251726	5.33750768996710
F	5.30571443404405	2.32273355270703	5.14530953739221
F	7.13671516264068	2.04750275166493	3.16315585087184
O	-0.96625832695078	3.39942580455141	-1.80644595261417
O	-1.79830896042345	1.14942226568486	-0.89110782917221
O	1.08544972914400	-1.01344200602341	-2.44817825994082
C	1.90517407764054	-2.13685029503081	-2.47745406736958
C	3.28298683176616	-1.97938984366550	-2.64328234046888
C	4.11692032688231	-3.14927999715016	-2.56701036622857
C	3.49648798847451	-4.44410683161633	-2.42290695002739
C	2.07883471650915	-4.54304861199790	-2.36114980906718
C	1.27249254843362	-3.41128884317204	-2.38770257553838
C	4.31982866723196	-5.60596008948860	-2.33875345672652
C	5.70177811793199	-5.50157616263806	-2.37404853845883
C	6.31334634158587	-4.22410309700584	-2.48626393744239
C	5.54037125029245	-3.07578895523990	-2.58128842372636
C	3.81702747916338	-0.61758579643990	-2.91558149751000
C	3.51535807290878	0.42811345215483	-2.04480844422648
C	3.90848032126796	1.78005479626992	-2.27721485419963
C	4.63775253103100	2.06582677230724	-3.42227716034065
C	4.96413931653065	1.04925926164294	-4.36327746544668
C	4.53717344463335	-0.30980228442850	-4.12773821389763
C	5.68146383098277	1.36152824075184	-5.55623820812923

C	5.94669464879120	0.38432392766473	-6.50280170328151
C	5.48652403819975	-0.94284186894811	-6.29533018739365
C	4.79723032581679	-1.28172176002545	-5.13918149637923
C	-0.20764581742027	-3.49761235685235	-2.38224534763830
C	-0.96389975521576	-2.97785301173439	-3.43078062957175
C	-2.38488004701651	-3.05707377626539	-3.48024339801090
C	-3.06572123578505	-3.75100469561694	-2.40659419425317
C	-2.28844340903801	-4.26158472357338	-1.32898042250692
C	-0.90655182724595	-4.14091658806493	-1.32970761914922
C	-4.48447783437562	-3.85637496137727	-2.45131331569206
C	-5.20984540099858	-3.27751212511851	-3.48337178576107
C	-4.54629172812478	-2.59044400565242	-4.53020239221657
C	-3.16223108522661	-2.48521244140625	-4.53011220934770
F	-0.31435187553040	-2.39014749894892	-4.44505395077288
F	-2.88174088090379	-4.85844336552339	-0.28416421781626
F	-0.20650073615596	-4.63228839034984	-0.29690909432039
O	2.74774855768263	0.15745350259358	-0.90289339561097
C	3.52669102496660	2.82449807458203	-1.29551294321654
C	2.61782881518515	3.83809533512825	-1.58393655110368
C	2.18075818825738	4.77944933082962	-0.60401915873043
C	2.74449808863943	4.69230177321717	0.72963624092763
C	3.69523456147686	3.66944051318002	1.00174979359219
C	4.05941460682417	2.76975209364489	0.01552428403557
C	2.30839526330208	5.61719783725646	1.71725948891371
C	1.34953691660386	6.57619425710168	1.42652540702938
C	0.78164894411236	6.65240897316169	0.13179935767014
C	1.19804693282127	5.78038345485621	-0.86716200206911
F	2.11458291423133	3.90691547066048	-2.81993411056757
F	4.23440525150152	3.54006674761341	2.22827600967517
F	4.94903166723788	1.80750828006081	0.29888306141734
F	0.64643678355075	5.90589856824590	-2.07443210079413

F	-0.15979163883697	7.56684051948202	-0.10880094981749
F	0.93666302809822	7.41953601170478	2.37429434300826
F	2.78247137123744	5.55999902571699	2.97203796694877
F	-5.15937632921620	-4.48238068895109	-1.47992246414968
F	-2.58268516734971	-1.82875825544133	-5.54054851880526
F	-5.27186062459779	-2.04271251329792	-5.50674640784591
F	-6.54183122101390	-3.35456552273410	-3.50355585032008
O	-0.94990670650578	0.84666866963080	1.92471399855996
H	6.02392056679219	-2.09138800323470	-2.64685319459850
H	7.41084747976822	-4.14311806943140	-2.48948467421122
H	6.32731259717847	-6.40416543252097	-2.30397126946445
H	3.83270240720633	-6.58799938220894	-2.23614266817907
H	1.61550195329570	-5.53841920786307	-2.29193056587014
H	4.43644722773543	-2.30963545979201	-5.00257289259903
H	6.00919093412759	2.40064796718420	-5.71376371253183
H	4.95828007093230	3.09993133836874	-3.61713573010827
H	-3.13191229508520	-5.27882863058618	2.57241136355600
H	-2.92883068426540	-7.74232979448780	2.39125564999843
H	-0.66582422648224	-8.83182788954221	2.27620227828448
H	1.39820680197920	-7.41798694133532	2.29617202949709
H	2.47209459070629	-5.22488020660327	2.36541730864813
H	-1.84902061624391	-4.40647439415542	5.00390786519655
H	-3.34890382099448	-4.80191206142884	6.93301192307902
H	-5.57700108512451	-3.64470371735834	7.07978594258979
H	-6.25672172654926	-2.02734575442568	5.29380891266955
H	-5.79061543487657	-0.85408513527331	3.19679237535131
H	6.49728434298669	0.63722685243076	-7.42129595323480
H	5.67161623004345	-1.71065216494767	-7.06166535485752
F	-1.54286056815582	0.05419306189550	-3.56862648193732
F	-2.68602340775427	1.90766079277951	-3.74313612387417
F	-0.62172303080737	1.82744896352612	-4.45282827379516

H	1.74603105858110	0.94171210083688	2.34130143697800
C	-1.73627087201890	5.90560894485353	2.67377378581936
C	-2.26188081734076	7.31309031584951	2.65041553629508
H	-1.47910032959388	8.03805172544168	2.95827318928522
H	-3.10150676330012	7.42878449480334	3.37039463742917
H	-2.62570431549493	7.59717553095692	1.64430761069267
C	-1.22541594126473	5.37274353001120	3.99080151847856
C	-1.71291426676286	5.12247604376639	1.57433358630441
C	-0.34714384900074	4.13252025628491	3.81652530995608
H	-2.09081268948750	5.14964181741250	4.65792926923142
H	-0.64810852166106	6.16797150533564	4.51012535564208
C	-1.02067940011917	3.07120732832383	2.92485525402524
H	-0.09940373078662	3.71120386482790	4.81054190055865
H	0.61547130944925	4.44257456190979	3.36249734808615
C	-1.35381053166328	3.65786702698097	1.53126831872063
H	-2.00931341353633	2.82012529597037	3.36744212763181
C	-0.20216960829572	1.75271700000931	2.90648137331861
O	-2.49356329954555	3.02110677736224	0.97392532325495
H	-0.47862212753787	3.53312000803481	0.84638028311976
H	-2.08093577517415	5.51208453772446	0.61015128963462
C	-0.24951295959854	1.07585076771663	4.27317204676850
C	1.21776299684388	1.91402142568029	2.37732095746446
H	-1.29811739634847	0.87267183132417	4.56542787961460
H	0.20035580228424	1.74783822828213	5.02782825498909
H	0.32344637526845	0.13512398929526	4.28871815344446
H	1.22023998126053	2.35075773171100	1.36087160770706
H	1.80105829154318	2.57450643738468	3.04559018951833
H	-2.20888160035769	2.23451898385845	0.45661388836364

Transition State connecting B (major) and the covalent adduct (major)

172

C	-4.79538423804587	3.44621310649579	1.45532731644251
C	-4.78527234326371	2.09106400193773	1.01832811542963
C	-5.08162628336524	1.80717947517287	-0.37107180558205
C	-5.35198453347686	2.89364785721523	-1.24653797488506
C	-5.33751090681254	4.20356388604485	-0.78895627416594
C	-5.07927186840685	4.48065896014242	0.57473281905830
C	-5.01513753192425	0.45817522091685	-0.81491277516198
C	-4.62663377572142	-0.54928316088235	0.05237896061001
C	-4.29551137651744	-0.30012814600171	1.40684951119663
C	-4.39937385303405	1.01020120137974	1.86261111146778
C	-3.85013570834235	-1.41260437785113	2.27856259899194
C	-2.66057036663711	-2.13613515655493	1.96127437496565
C	-2.19322213070344	-3.19831487176839	2.73904856893385
C	-2.91210296632810	-3.53632230721240	3.94347287259339
C	-4.12359974246008	-2.82127706701570	4.26476855293308
C	-4.56678416054761	-1.77437077777190	3.41167018002339
C	-2.44499085547921	-4.51673959964100	4.86930759101294
C	-3.16120492056482	-4.81059776391783	6.02123615286913
C	-4.37802936712991	-4.13778200837430	6.31065343903279
C	-4.84361282018928	-3.15613116623049	5.45026678969156
C	-0.91723236053171	-3.87523936255047	2.38176974282795
C	0.22817566279117	-3.08981045145526	2.24625694710702
C	1.52812140686431	-3.62842581656890	2.00877155858316
C	1.63664919882664	-5.00824132461741	1.86143639837757
C	0.49791811856983	-5.85762497092525	1.92131605731743
C	-0.80604560650986	-5.29378693371592	2.17388399941084
C	0.61905498905846	-7.26446093554335	1.71480361776437
C	-0.49852790775265	-8.08452129039039	1.73147887804222
C	-1.78629037035524	-7.52383113242665	1.94658530277481
C	-1.93674344225670	-6.16160037191533	2.16319969655447

O	0.09728290063042	-1.71106303791228	2.35364415704031
P	-0.53302857626434	-0.90933639106677	1.06041054964452
O	-1.94438359309204	-1.73941129562770	0.82852298540542
C	2.70345133242838	-2.73211778619816	1.92083461303094
C	2.97405954369788	-1.76709345488898	2.89114739553970
C	4.09225046221001	-0.88817991337881	2.82606622971611
C	5.05316405895890	-1.07962901597171	1.76100882293584
C	4.77465686818925	-2.06618328716185	0.77440918760995
C	3.63000012983681	-2.84466858895075	0.84754685694212
C	6.21896348807351	-0.26464510523900	1.73511402067958
C	6.40821673616058	0.73445736206907	2.68090420524185
C	5.44067709633943	0.95949254182763	3.69069808264299
C	4.30267446282811	0.16740266333794	3.75876811635860
F	2.17165338829899	-1.69332860893343	3.96029171760060
F	5.61049731857681	-2.24714734158129	-0.26018637587858
F	3.39936781495145	-3.73793557892383	-0.12762756326372
F	7.14714728561217	-0.41532276699398	0.78035462851190
F	7.49196949203894	1.51406599637726	2.63839098004465
F	5.62912869706686	1.95746128403362	4.55734376696576
F	3.40841850222500	0.43474235726019	4.71567764780535
F	-4.08416476156017	1.26432196268569	3.13921600358924
F	-5.30770270067513	0.13458812599783	-2.08973138774041
F	-4.57431375282737	-1.81118113066275	-0.39379984957857
F	-5.55099217643980	2.68829682775831	-2.56095644587150
F	-5.53348691083504	5.21884853067641	-1.63601798900341
F	-5.05637635036294	5.75066991237515	0.98803894696904
F	-4.50057049830821	3.76911792720479	2.71801588644438
N	0.36455174324623	-1.15790021202120	-0.23106531037392
P	0.88067343112967	-0.03862845493314	-1.26539856153473
O	0.20619940670084	1.30940217568339	-1.38998974646696
O	0.88271754350147	-0.76447811270482	-2.76818776295007

C	1.70517583124023	-1.87056028810524	-2.93255657854435
C	1.09942796565549	-3.16257648479746	-2.92187681231262
C	1.92220850893961	-4.27691513786810	-3.03395341879145
C	3.33331409502663	-4.14748467831259	-3.14997185101421
C	3.93268158665226	-2.83461477542181	-3.16869096257917
C	3.07987507773343	-1.67828814917085	-3.08965840908844
C	4.17232189176964	-5.29903624530297	-3.21745587042014
C	5.55087075169370	-5.17085844704023	-3.28399024660428
C	6.14296713938466	-3.87961965873485	-3.27297775388705
C	5.35419269099095	-2.73969445208154	-3.21787985426795
C	-0.37036059109030	-3.28435575067262	-2.77976260376847
C	-1.24528282311185	-2.66985286395001	-3.67127384597407
C	-2.66231473642800	-2.74677793436834	-3.55539974256738
C	-3.21421801714152	-3.55268126073261	-2.48552792107955
C	-2.31606376174637	-4.15669987082982	-1.56140955446181
C	-0.94296279031198	-4.02906007156325	-1.71508838397166
C	-3.55941293035249	-2.06136160948798	-4.42584810957009
C	-4.93384030508540	-2.17567240171286	-4.27261824268037
C	-5.47111903879135	-2.99723588814866	-3.25055129317705
C	-4.62840893685730	-3.68080996495091	-2.38521720186632
F	-6.79597922809580	-3.08773164030750	-3.12273415976953
F	-5.18431262180688	-4.42424730156554	-1.42303318686242
F	-3.10178139058051	-1.25447239616610	-5.39495622878685
F	-5.76610182925918	-1.50541809823890	-5.07263462128833
F	-0.72247210558548	-1.96493075422175	-4.69093289158243
F	-0.13587648684288	-4.60332174800473	-0.81525245297609
F	-2.78255262267064	-4.84466855396829	-0.51010590661137
C	3.59054276709617	-0.28132991354000	-3.13484925383556
C	3.26533623282529	0.58866312448151	-2.08853863805490
C	3.63821944343331	1.96986682849606	-2.09202315756122
C	4.37869446236863	2.44891548210960	-3.16716906457177

C	4.72708549293443	1.61577586190548	-4.26507665105054
C	4.30911542737062	0.23448876122790	-4.27123350561468
C	4.58197013435318	-0.54692899377788	-5.43402863372715
C	5.27527331408173	-0.00907016033844	-6.50926324199124
C	5.72737792199103	1.33752186123629	-6.48058672045148
C	5.45025630995656	2.13437141802906	-5.38088438620613
O	2.52097937836057	0.10125012129628	-1.02195210690475
C	3.20747159312247	2.87866743811761	-1.00040339546218
C	3.57579680812384	2.63090377650566	0.34549563669975
C	3.17313560512204	3.44856405583911	1.38891126297407
C	2.36201571418517	4.58847101222015	1.14548202324442
C	1.99072476900560	4.89310793733877	-0.22219638431286
C	2.44082387452018	4.01461356150296	-1.24830227262517
C	1.19284339789568	6.04919679384464	-0.46603650649173
C	0.73526611035141	6.83581104911508	0.58050013866303
C	1.06376301823239	6.51018950549693	1.92024404884863
C	1.85822956534547	5.40599848094147	2.19613918248563
F	4.34134541183371	1.56895423437387	0.61441437521345
F	3.55986086858969	3.14083922309582	2.63301234196002
F	2.06714769001473	4.26724661262348	-2.52270404720689
F	0.84511257138216	6.40558590737241	-1.71319478953086
F	-0.01883356211972	7.91401349983197	0.34385992728370
F	0.59195496293296	7.27692515470770	2.90556148771021
F	2.13232429086520	5.13032426935965	3.47465964015772
N	-0.94008459491660	0.58960298495963	1.54653268870223
S	0.21720087023866	1.57349184472999	2.12735582140758
O	1.59488127397089	1.03720429215599	1.95681987005217
C	-0.08604451192198	1.55163970324768	3.98915530613788
F	0.76750154774558	2.41479226788558	4.55789808652207
O	-0.08343030802676	2.97124479215337	1.73839492900426
F	-1.34360560001549	1.93205459309428	4.24921029480508

F	0.11978138072729	0.32890148519530	4.48593808791997
H	-0.45928973888764	3.37616817094699	-0.95851100313392
C	-1.55886369434789	3.47639998179742	-1.05005948530478
C	-2.02214486106029	4.83436591718196	-0.58344407196177
C	-2.34034974459428	5.89600240603168	-1.35661637194955
C	-2.24528465944825	5.81258133364400	-2.86019706922039
C	-1.44716594216847	4.60447245630270	-3.34496895564470
C	-1.90595478047632	3.32857787069701	-2.63038663905181
H	-3.01543889563334	3.26521548056652	-2.60510824289204
C	-2.83860680484727	7.19267432534496	-0.78571676602731
H	-2.93838262780043	-5.73480990930517	2.30970309743024
H	-2.67373827926605	-8.17454295009959	1.93423957971420
H	-0.39319447082298	-9.16735708584897	1.56612400010032
H	1.62054620026279	-7.68331294373344	1.53062524674645
H	2.62287206944956	-5.45923895751348	1.67986834450427
H	-1.49834138374809	-5.03625852694282	4.67016047384504
H	-5.77191681995633	-2.60761664109131	5.67302371589787
H	-5.48999852837603	-1.23235502117685	3.66477415992165
H	5.82609139745142	-1.74845519230117	-3.18309002643792
H	7.23880220071243	-3.78179369577630	-3.29806021193166
H	6.18894702406906	-6.06617074464544	-3.33052835860386
H	3.69996493077403	-6.29350428124299	-3.20487576145062
H	1.47916106381542	-5.28376106094536	-3.02373445436938
H	4.22997470171620	-1.58639454700374	-5.47586902204226
H	5.47214874059822	-0.63183388308999	-7.39507854712460
H	6.28295693282111	1.74818842625851	-7.33705696024777
H	5.77299532171588	3.18669298002697	-5.35326230077346
H	4.68708007479944	3.50475259049396	-3.18352625102360
H	-4.93847429319228	-4.38540205028307	7.22464510674581
H	-2.77676061329191	-5.56802940733251	6.72098738640413
H	-3.86531695932610	7.40228874052757	-1.15471533717570

H	-2.19659397378793	8.03661707186093	-1.11253815833127
H	-2.86440244728493	7.17560985525765	0.31939422696222
H	-1.78431923954943	6.74608398735992	-3.24808020156911
H	-3.27819633496178	5.79570630251376	-3.27937397954687
H	-0.36919579147077	4.76785010933379	-3.14870710724495
H	-1.56361197913998	4.48591541705287	-4.44134633350094
C	-1.40651721532360	2.05808194303716	-3.16020936762669
O	-2.20340190045940	2.48589888120514	-0.31148599282774
H	-2.09831429950810	4.89942806140460	0.51507898342661
H	-1.54215572200977	1.85790740678157	0.07046186550573
C	-2.20614828565175	0.85810457656371	-2.88171138871989
C	-0.26378172420205	2.02258414519350	-4.08790426256639
H	-3.17695086413899	0.93586288776111	-3.42159278089162
H	-1.69907924852279	-0.08056999388235	-3.15261914599890
H	-2.49297921440377	0.88959119487207	-1.80343420942977
H	0.12609764660501	1.00375385090166	-4.25061000616407
H	-0.64553195985833	2.42225168141443	-5.06044228483091
H	0.53923671533137	2.71901409865984	-3.77838355355175

Transition State connecting B (minor) and the covalent adduct (minor)

172

C	6.67930553388207	0.55551553251565	2.50440709386433
C	6.35634490178518	-0.51206121920732	1.67693611113059
C	5.11758370902371	-1.19787822493435	1.82105968448964
C	4.20680450438753	-0.78687361429802	2.86984736592910
C	4.56123709599523	0.33102470123742	3.67919065228152
C	5.77042222660116	0.98879976778028	3.50154907745685
C	3.01073674931787	-1.53901304738882	3.05209283127521
C	2.62820734325753	-2.58841083756669	2.21794832734935
C	3.51401312003522	-2.91888682227242	1.15467636425000

C	4.72536946359893	-2.26931903902497	0.97257984270365
C	1.37989635818946	-3.36045894897252	2.42039719117631
C	0.12294265661739	-2.69403028695771	2.54199416776060
C	-1.08538153762742	-3.36899672475710	2.72155374710350
C	-1.07860037907143	-4.80995564795426	2.71325449452330
C	0.18420353942727	-5.49782714547605	2.59659678537506
C	1.38550252409191	-4.75084493968334	2.46338760694450
C	0.20530136523926	-6.92443306436023	2.58604063317889
C	-0.97041579298792	-7.65297237114978	2.67115471067277
C	-2.21647172242166	-6.97660168435962	2.75672284499699
C	-2.27021248232943	-5.59066489651954	2.77678970975898
O	0.11123275252405	-1.31068716925832	2.49159101998324
P	-0.42108206300326	-0.56341115906235	1.10853317016347
N	0.52571366881103	-1.01390203645368	-0.09995734735420
P	1.17782411136440	-0.17379654713794	-1.28416213149680
N	0.73326736831092	1.30513387056948	-1.72335866759503
S	-0.76450663134118	1.85922061781337	-1.88004786644268
C	-1.09089439595535	1.67643853553055	-3.72859677616155
C	-2.34144822875364	-2.58559793026794	2.87539687159022
C	-2.70312328574249	-1.64339341230140	1.90359328556250
C	-3.95601433859911	-0.94752750068264	1.94347064921062
C	-4.80790897598937	-1.17930234009378	3.01820875515606
C	-4.44619818048141	-2.04497502096578	4.08523527889924
C	-3.18900818138292	-2.74941018113187	4.02972540012490
C	-5.29656166329231	-2.21396518881505	5.21905651308950
C	-4.91432485980769	-3.02089661191965	6.27919183855018
C	-3.65681797890359	-3.68153393396228	6.24655822826716
C	-2.81590185592259	-3.55110299029322	5.15021217647924
F	3.17935660245833	-3.89302845159771	0.29818687852296
F	5.51530031195830	-2.66165482743804	-0.03535269559979
F	2.23085798017846	-1.24293493734218	4.10784749336940

O	-1.84553937889851	-1.38412298725358	0.84597077054139
C	-4.31577553793146	0.03345993997629	0.89030572977057
C	-4.34898939881929	-0.33065868773214	-0.48039686098885
C	-4.61697829373052	0.58426732327339	-1.48409655495974
C	-4.85123120706197	1.95336206528392	-1.17609517296141
C	-4.86383241741863	2.35181877441919	0.21505413128173
C	-4.62038030821927	1.35575312923835	1.20019183472593
C	-4.99989553321164	3.73323873333867	0.53268403080273
C	-5.09424707195666	4.68878358263348	-0.46766104953315
C	-5.10600014030086	4.29743423813665	-1.83024641365929
C	-4.99826307281048	2.95614343243576	-2.17684014463569
F	-4.09069185637374	-1.60304368519891	-0.81289361555396
F	-4.59131512699413	0.16457917739133	-2.75809544374653
F	-4.60989089114037	1.72215997263487	2.49931157712765
F	-5.02533994433128	2.63731135513487	-3.47559018984461
F	-4.98024947504176	4.15938509281991	1.80264982607700
F	-5.16278674789701	5.99053139736691	-0.16391411240143
F	-5.22404862373672	5.23611927044993	-2.77169527678155
F	7.23098970945521	-0.86104796810208	0.72610457310072
F	3.72627942693462	0.79872446602774	4.61903344393563
F	6.08551403414702	2.04853648894315	4.24928471313635
F	7.83312044298732	1.20837509179057	2.35222755238686
O	-0.78564791864975	3.31776580542458	-1.64143287336787
O	-1.79600776640694	0.98685190086908	-1.25184399469376
O	1.20412251006864	-1.11018766491558	-2.64674264953690
C	2.05528209385497	-2.20196451246972	-2.71723991822844
C	3.42512537951426	-1.98478714129419	-2.89902643417244
C	4.30798940678642	-3.12091544010552	-2.91607248839539
C	3.74245707388971	-4.44468491777617	-2.82457066637112
C	2.33673474498370	-4.60174971517007	-2.69073054429949
C	1.48166288552658	-3.50651418807092	-2.62012820942938

C	4.60949661132596	-5.57804145434765	-2.84560619266853
C	5.98361078121565	-5.41994659539686	-2.93187690458217
C	6.54349408178071	-4.11511221857894	-2.98789648277447
C	5.72708945239333	-2.99374652818688	-2.98025996272875
C	3.91069067412195	-0.58489482231942	-3.04218815831004
C	3.57208906635541	0.35329382546681	-2.06507209453942
C	3.92299888464295	1.73484860662165	-2.15787209391088
C	4.66558951070333	2.15352366167940	-3.25403546902944
C	5.03294409602615	1.25017005532580	-4.28956865664071
C	4.63025715035864	-0.13360452822336	-4.20724739972149
C	5.75558097721221	1.70264348160368	-5.43378370594181
C	6.04619450227780	0.83933426110024	-6.47850776731580
C	5.60726385720955	-0.51021823628823	-6.42209518772226
C	4.91515524797665	-0.98453885103127	-5.31647277029062
C	0.02348770614026	-3.67790599999384	-2.41962549862464
C	-0.92405326493212	-3.08244996562901	-3.25231756602752
C	-2.32979292977200	-3.23540857124380	-3.07045843389538
C	-2.79226173784477	-4.11962573701815	-2.02010252264266
C	-1.82070106750029	-4.73729097400200	-1.18475002057537
C	-0.46701422200617	-4.50214408151036	-1.36962571327769
C	-4.19033970000249	-4.33678603923413	-1.86929475839434
C	-5.10298228393898	-3.69456972821050	-2.69568714478461
C	-4.65546117479019	-2.80282238256515	-3.70092645092123
C	-3.29762508897865	-2.57613604689934	-3.88341536213431
F	-0.49156011010514	-2.36873661733480	-4.29945644491427
F	-2.19737860561982	-5.55629402680165	-0.19164791470906
F	0.40143187928358	-5.08862246096678	-0.53195569837734
O	2.80250943631417	-0.05632584017260	-0.97477992121928
C	3.43970217590046	2.69146187416033	-1.13290959889468
C	2.58410316446980	3.74292087593012	-1.45665940295191
C	2.06377305788936	4.64457127833995	-0.48289017525123

C	2.46184969373698	4.46575500565309	0.89973447469402
C	3.31984546937135	3.37835202999260	1.22073451784893
C	3.78697761561052	2.52985762077963	0.23026632832230
C	1.99149773739852	5.39399932409236	1.87016474785925
C	1.13365640623421	6.42526988793985	1.51937803999415
C	0.69962023281877	6.56968850345674	0.17908557716140
C	1.16016854825153	5.70191847909429	-0.80293354645472
F	2.21856833519967	3.89337702093446	-2.73306175182346
F	3.69090337330911	3.15237536365568	2.49627565756169
F	4.60950924250356	1.52896755503016	0.57210491736938
F	0.73377360753030	5.89258035974289	-2.05086779524447
F	-0.16832218093654	7.54040882877860	-0.11795462036157
F	0.69087342571844	7.28045468807077	2.44613557880588
F	2.34059011269279	5.27512137851918	3.16446912177368
F	-4.66464783103888	-5.14627444703478	-0.91259107306306
F	-2.93046447204196	-1.71652718773239	-4.83817024393662
F	-5.55824559657699	-2.17102955879279	-4.45378660990993
F	-6.41473530902801	-3.88625878824729	-2.53695306831675
O	-0.58764484143800	0.89492170814156	1.50420700721475
H	6.17479397991704	-1.99080471978148	-2.99718093643574
H	7.63638163170235	-3.99189676281616	-3.02822159192542
H	6.64336548607639	-6.30059447053476	-2.94302049231226
H	4.16155080089218	-6.58180533671364	-2.78151656482589
H	1.92333371943058	-5.61845043120418	-2.62277833187774
H	4.57075539669269	-2.02698353586678	-5.29443240506316
H	6.06435334287904	2.75866836663863	-5.47463027530931
H	4.95323053229090	3.21159062356359	-3.34271636816992
H	-3.24353414147664	-5.08371653916389	2.81311516265418
H	-3.15066196156712	-7.55705652973300	2.79408162591881
H	-0.94312004526189	-8.75293326131638	2.65693399557256
H	1.17740033219629	-7.43377945470257	2.49784277470242

H	2.33722049032699	-5.29395963316020	2.37226991828218
H	-1.84281572495303	-4.06072253599364	5.14364248201531
H	-3.34329750783911	-4.30012269458977	7.10111161435446
H	-5.57492296833729	-3.14128916055198	7.15093580028214
H	-6.25857422191097	-1.67879012290951	5.23723514292434
H	-5.77712589484468	-0.66136136592792	3.05889690324222
H	6.59971008362454	1.20039846298354	-7.35832090615529
H	5.81125496461265	-1.18620880284793	-7.26623274348000
F	-1.07627725476412	0.38092064971003	-4.06890249088744
F	-2.29839281101822	2.19108466270929	-4.00827688042591
F	-0.15881966299116	2.33070987596843	-4.43233525300970
H	0.97546093775677	0.92018590561623	3.86361945863902
C	-2.07532512515703	6.02987445016107	2.49404782065402
C	-2.65169058469543	7.37060694214310	2.14142209581398
H	-1.90973070830322	8.17723273164080	2.32169630098906
H	-3.53313510395010	7.59750043835944	2.77910519740843
H	-2.96872967356106	7.41137324602947	1.08246625498072
C	-1.76025276382278	5.77004124221208	3.94496692877369
C	-1.85275317613718	5.07776758286740	1.56374950427657
C	-0.91010370056154	4.52217819059858	4.16302120704595
H	-2.71543418071751	5.70399350048795	4.51556654239837
H	-1.22500410709924	6.64814527394206	4.36699977002909
C	-1.49496895749123	3.31471339295774	3.37944082174874
H	-0.86281908527812	4.27324446100561	5.24254181133683
H	0.12955595274057	4.71006096190326	3.83175145955922
C	-1.37755904511361	3.67020972438589	1.79927808222796
H	-2.58845682950318	3.24137290989962	3.54410117932129
C	-0.85426711390792	2.04934312787138	3.73923793444947
O	-2.17921827483844	2.84153517720258	1.04294399759475
H	-0.29722504487813	3.55157489192040	1.53825530434914
H	-2.10342569285107	5.26444834305459	0.50631214799448

C	-1.70973385150433	0.94167418607931	4.19765693972695
C	0.60914850159446	1.96039209353373	3.89548419288767
H	-2.64477015126709	0.89070872977654	3.60510057812722
H	-2.04003119512969	1.21555156157189	5.23054721086178
H	-1.19350197934734	-0.03285339710257	4.23658282689902
H	1.15889728332600	2.60537935449092	3.18505485039405
H	0.84352805447279	2.36753471819314	4.91005584654702
H	-1.69370497853534	1.97627638775026	0.94373488330639

References

1. Bougioukou, D. J., Walton, A. Z. & Stewart, J. D. Towards preparative-scale, biocatalytic alkene reductions. *Chemical Communications* **46**, 8558 (2010).

2. Kaib, P. S. J., Schreyer, L., Lee, S., Properzi, R. & List, B. Extremely Active Organocatalysts Enable a Highly Enantioselective Addition of Allyltrimethylsilane to Aldehydes. *Angewandte Chemie International Edition* **55**, 13200–13203 (2016).

3. Schwengers, S. A. *et al.* Unified Approach to Imidodiphosphate-Type Brønsted Acids with Tunable Confinement and Acidity. *Journal of the American Chemical Society* **143**, 14835–14844 (2021).

4. He, Y., Bian, Z., Kang, C. & Gao, L. Stereoselective and hierarchical self-assembly from nanotubular homochiral helical coordination polymers to supramolecular gels. *Chemical Communications* **46**, 5695 (2010).

5. Simonsen, K. B., Gothelf, K. V. & Jørgensen, K. A. A Simple Synthetic Approach to 3,3‘-Diaryl BINOLs. *The Journal of Organic Chemistry* **63**, 7536–7538 (1998).

6. Kim, J. H., Čorič, I., Vellalath, S. & List, B. The catalytic asymmetric acetalization. *Angewandte Chemie International Edition* **52**, 4474–4477 (2013).

7. Díaz-Oviedo, C. D., Maji, R. & List, B. The Catalytic Asymmetric Intermolecular Prins Reaction. *Journal of the American Chemical Society* **143**, 20598–20604 (2021).

8. Matsumoto, M. *et al.* Color composition for making color filter used in liquid crystal display. *JP 2015 118267* (2015).

9. Armarego, W. L. F. & Chai, C. L. L. *Purification of Laboratory Chemicals*. (Oxford, 2003).

10. Fulmer, G. R. *et al.* NMR Chemical Shifts of Trace Impurities: Common Laboratory Solvents, Organics, and Gases in Deuterated Solvents Relevant to the Organometallic Chemist. *Organometallics* **29**, 2176–2179 (2010).

11. Gottlieb, H. E., Kotlyar, V. & Nudelman, A. NMR Chemical Shifts of Common Laboratory Solvents as Trace Impurities. *The Journal of Organic Chemistry* **62**, 7512–7515 (1997).

12. Harris, R. K. *et al.* Further conventions for NMR shielding and chemical shifts (IUPAC Recommendations 2008). *Pure and Applied Chemistry* **80**, 59–84 (2008).

13. Mohr, J., Durmaz, M., Irran, E. & Oestreich, M. Tris(5,6,7,8-tetrafluoronaphthalen-2-yl)borane, a Partially Fluorinated Boron Lewis Acid with Fluorination Distal to the Boron Atom. *Organometallics* **33**, 1108–1111 (2014).

14. Shevchenko, G., Pupo, G. & List, B. Direct Asymmetric α-Hydroxylation of Cyclic α-Branched Ketones through Enol Catalysis. *Synlett* **30**, 49–53 (2019).

15. Lee, S., Bae, H. Y. & List, B. Can a Ketone Be More Reactive than an Aldehyde? Catalytic Asymmetric Synthesis of Substituted Tetrahydrofurans. *Angewandte Chemie*

International Edition **57**, 12162–12166 (2018).

16. Golliher, A. E. *et al.* Using (+)-carvone to access novel derivatives of (+)-ent-cannabidiol: The first asymmetric syntheses of (+)-ent-CBDP and (+)-ent-CBDV. *Tetrahedron Letters* **67**, 152891 (2021).

17. Cheallaigh, A. N. *et al.* Chemoenzymatic Synthesis of the Intermediates in the Peppermint Monoterpenoid Biosynthetic Pathway. *Journal of Natural Products* **81**, 1546–1552 (2018).

18. Subrahmanyam, G., Joshi, S., Bonde, N. L., Bhatt, J. G. & Sitaram, G. M. Process and Compounds for Preparation of Cannabinoids. *WO 2020/099942 A1* (2020).

19. Dethe, D. H., Erande, R. D., Mahapatra, S., Das, S. & Kumar B., V. Protecting group free enantiospecific total syntheses of structurally diverse natural products of the tetrahydrocannabinoid family. *Chemical Communications* **51**, 2871–2873 (2015).

20. Selifonov, S. A. Methods for Making (-)-Menthol and Oxygenated Menthane Compounds. *WO 2004/013339 A1* (2004).

21. Cantín, Á., Lull, C., Primo, J., Miranda, M. A. & Primo-Yúfera, E. Isolation, structural assignment and insecticidal activity of (−)-(1S,2R,3R,4S)-1,2-epoxy-1-methyl-4-(1-methylethyl)-cyclohex-3-yl acetate, a natural product from Minthostachys tomentosa. *Tetrahedron: Asymmetry* **12**, 677–683 (2001).

22. Chiurchiù, E. *et al.* A Novel and Practical Continuous Flow Chemical Synthesis of Cannabidiol (CBD) and its CBDV and CBDB Analogues. *European Journal of Organic Chemistry* **2021**, 1286–1289 (2021).

23. Schafroth, M. A., Zuccarello, G., Krautwald, S., Sarlah, D. & Carreira, E. M. Stereodivergent Total Synthesis of Δ 9 -Tetrahydrocannabinols. *Angewandte Chemie International Edition* **53**, 13898–13901 (2014).

24. Kutateladze, D. A., Strassfeld, D. A. & Jacobsen, E. N. Enantioselective Tail-to-Head Cyclizations Catalyzed by Dual-Hydrogen-Bond Donors. *Journal of the American Chemical Society* **142**, 6951–6956 (2020).

25. Ludwig, J. R. *et al.* Interrupted carbonyl-olefin metathesis via oxygen atom transfer. *Science* **361**, 1363–1369 (2018).

26. Liu, F. *et al.* Rationally Designed Short Polyisoprenol-Linked PglB Substrates for Engineered Polypeptide and Protein N-Glycosylation. *Journal of the American Chemical Society* **136**, 566–569 (2014).

27. Wise, M. L. *et al.* Stereochemical disposition of the geminal dimethyl groups in the enzymatic cyclization of geranyl diphosphate to (+)-bornyl diphosphate by recombinant (+)-bornyl diphosphate synthase from Salvia officinalis. *Tetrahedron* **57**, 5327–5334 (2001).

28. Ouyang, J., Kennemur, J. L., De, C. K., Farès, C. & List, B. Strong and Confined Acids Enable a Catalytic Asymmetric Nazarov Cyclization of Simple Divinyl Ketones. *Journal of the American Chemical Society* **141**, 3414–3418 (2019).

29. Nielsen, C. D. T. & Burés, J. Visual kinetic analysis. *Chemical Science* **10**, 348–353 (2019).

30. Burés, J. A Simple Graphical Method to Determine the Order in Catalyst. *Angewandte Chemie* **128**, 2068–2071 (2016).

31. Baxter, R. D., Sale, D., Engle, K. M., Yu, J.-Q. & Blackmond, D. G. Mechanistic Rationalization of Unusual Kinetics in Pd-Catalyzed C–H Olefination. *Journal of the American Chemical Society* **134**, 4600–4606 (2012).

32. Blackmond, D. G. Kinetic Profiling of Catalytic Organic Reactions as a Mechanistic Tool. *Journal of the American Chemical Society* **137**, 10852–10866 (2015).

33. Neese, F. Software update: The <scp>ORCA</scp> program system—Version 5.0. *WIREs Computational Molecular Science* **12**, 1–15 (2022).

34. Grimme, S., Bannwarth, C. & Shushkov, P. A Robust and Accurate Tight-Binding Quantum Chemical Method for Structures, Vibrational Frequencies, and Noncovalent Interactions of Large Molecular Systems Parametrized for All spd-Block Elements (*Z* = 1–86). *Journal of Chemical Theory and Computation* **13**, 1989–2009 (2017).

35. Bannwarth, C., Ehlert, S. & Grimme, S. GFN2-xTB—An Accurate and Broadly Parametrized Self-Consistent Tight-Binding Quantum Chemical Method with Multipole Electrostatics and Density-Dependent Dispersion Contributions. *Journal of Chemical Theory and Computation* **15**, 1652–1671 (2019).

36. Perdew, J. P., Burke, K. & Ernzerhof, M. Generalized Gradient Approximation Made Simple. *Physical Review Letters* **77**, 3865–3868 (1996).

37. Grimme, S., Antony, J., Ehrlich, S. & Krieg, H. A consistent and accurate *ab initio* parametrization of density functional dispersion correction (DFT-D) for the 94 elements H-Pu. *The Journal of Chemical Physics* **132**, 154104 (2010).

38. Grimme, S., Ehrlich, S. & Goerigk, L. Effect of the damping function in dispersion corrected density functional theory. *Journal of Computational Chemistry* **32**, 1456–1465 (2011).

39. Weigend, F. & Ahlrichs, R. Balanced basis sets of split valence, triple zeta valence and quadruple zeta valence quality for H to Rn: Design and assessment of accuracy. *Physical Chemistry Chemical Physics* **7**, 3297 (2005).

40. Yepes, D., Neese, F., List, B. & Bistoni, G. Unveiling the Delicate Balance of Steric and Dispersion Interactions in Organocatalysis Using High-Level Computational Methods. *Journal of the American Chemical Society* **142**, 3613–3625 (2020).

41. Gatzenmeier, T. *et al.* Scalable and Highly Diastereo- and Enantioselective Catalytic Diels–Alder Reaction of α,β-Unsaturated Methyl Esters. *Journal of the American Chemical Society* **140**, 12671–12676 (2018).

42. Ghosh, S. *et al.* Strong and Confined Acids Control Five Stereogenic Centers in Catalytic Asymmetric Diels–Alder Reactions of Cyclohexadienones with Cyclopentadiene. *Angewandte Chemie International Edition* **59**, 12347–12351 (2020).

43. Barone, V. & Cossi, M. Quantum Calculation of Molecular Energies and Energy Gradients in Solution by a Conductor Solvent Model. *The Journal of Physical Chemistry A* **102**, 1995–2001 (1998).

44. Garcia-Ratés, M. & Neese, F. Effect of the Solute Cavity on the Solvation Energy and its Derivatives within the Framework of the Gaussian Charge Scheme. *Journal of Computational Chemistry* **41**, 922–939 (2020).

45. Eichkorn, K., Treutler, O., Öhm, H., Häser, M. & Ahlrichs, R. Auxiliary basis sets to approximate Coulomb potentials. *Chemical Physics Letters* **240**, 283–290 (1995).

46. Neese, F. *An Improvement of the Resolution of the Identity Approximation for the Formation of the Coulomb Matrix*. *J Comput Chem* **24**, (2003).

47. Weigend, F. Accurate Coulomb-fitting basis sets for H to Rn. *Physical Chemistry Chemical Physics* **8**, 1057 (2006).

48. Pracht, P., Bohle, F. & Grimme, S. Automated exploration of the low-energy chemical space with fast quantum chemical methods. *Physical Chemistry Chemical Physics* **22**, 7169–7192 (2020).

49. Contreras-García, J. *et al.* NCIPLOT: A Program for Plotting Noncovalent Interaction Regions. *Journal of Chemical Theory and Computation* **7**, 625–632 (2011).

50. Bistoni, G. Finding chemical concepts in the Hilbert space: Coupled cluster analyses of noncovalent interactions. *WIREs Computational Molecular Science* **10**, 1–22 (2020).

51. McKeown, G. R. *et al.* Synthesis of Macrocyclic Poly(3-hexylthiophene) and Poly(3-heptylselenophene) by Alkyne Homocoupling. *ACS Macro Letters* **5**, 1075–1079 (2016).
